# Mek1 Down Regulates Rad51 Activity during Yeast Meiosis by Phosphorylation of Hed1

**DOI:** 10.1371/journal.pgen.1006226

**Published:** 2016-08-02

**Authors:** Tracy L. Callender, Raphaelle Laureau, Lihong Wan, Xiangyu Chen, Rima Sandhu, Saif Laljee, Sai Zhou, Ray T. Suhandynata, Evelyn Prugar, William A. Gaines, YoungHo Kwon, G. Valentin Börner, Alain Nicolas, Aaron M. Neiman, Nancy M. Hollingsworth

**Affiliations:** 1 Department of Biochemistry and Cell Biology, Stony Brook University, Stony Brook, New York, United States of America; 2 Institut Curie, PSL Research University, CNRS, UMR 3244, Paris, France; 3 Sorbonne Universités, UPMC Univ Paris 06, CNRS, UMR 3244, Paris, France; 4 Center for Gene Regulation in Health and Disease and Department of Biological Sciences, Cleveland State University, Cleveland, Ohio, United States of America; 5 Department of Molecular Biophysics and Biochemistry, Yale University School of Medicine, New Haven, Connecticut, United States of America; National Cancer Institute, UNITED STATES

## Abstract

During meiosis, programmed double strand breaks (DSBs) are repaired preferentially between homologs to generate crossovers that promote proper chromosome segregation at Meiosis I. In many organisms, there are two strand exchange proteins, Rad51 and the meiosis-specific Dmc1, required for interhomolog (IH) bias. This bias requires the presence, but not the strand exchange activity of Rad51, while Dmc1 is responsible for the bulk of meiotic recombination. How these activities are regulated is less well established. In *dmc1Δ* mutants, Rad51 is actively inhibited, thereby resulting in prophase arrest due to unrepaired DSBs triggering the meiotic recombination checkpoint. This inhibition is dependent upon the meiosis-specific kinase Mek1 and occurs through two different mechanisms that prevent complex formation with the Rad51 accessory factor Rad54: (i) phosphorylation of Rad54 by Mek1 and (ii) binding of Rad51 by the meiosis-specific protein Hed1. An open question has been why inhibition of Mek1 affects Hed1 repression of Rad51. This work shows that Hed1 is a direct substrate of Mek1. Phosphorylation of Hed1 at threonine 40 helps suppress Rad51 activity in *dmc1Δ* mutants by promoting Hed1 protein stability. Rad51-mediated recombination occurring in the absence of Hed1 phosphorylation results in a significant increase in non-exchange chromosomes despite wild-type levels of crossovers, confirming previous results indicating a defect in crossover assurance. We propose that Rad51 function in meiosis is regulated in part by the coordinated phosphorylation of Rad54 and Hed1 by Mek1.

## Author Summary

Sexual reproduction requires the formation of haploid gametes by a highly conserved, specialized cell division called meiosis. Failures in meiotic chromosome segregation lead to chromosomally imbalanced gametes that cause infertility and birth defects such as Trisomy 21 in humans. Meiotic crossovers, initiated by programmed double strand breaks (DSBs), are critical for proper chromosome segregation. Interhomolog strand invasion requires the presence of Rad51, and the strand invasion activity of the meiosis-specific recombinase Dmc1. The meiosis-specific kinase, Mek1, is a key regulator of meiotic recombination, promoting interhomolog strand invasion and recombination pathway choice. Rad51 activity during meiosis is inhibited by preventing the Rad51 protein from forming complexes with an accessory factor, Rad54, in two ways: (1) Mek1 phosphorylation of Rad54 and (2) binding of Rad51 by a meiosis-specific protein, Hed1. Why inactivation of Mek1 affects Hed1-mediated repression of Rad51 was previously unknown. This work demonstrates that Mek1 regulates the ability of Hed1 to inhibit Rad51 by direct phosphorylation of Hed1. Therefore in meiosis, Rad51 activity is regulated in part by the coordinated phosphorylation of both Rad54 and Hed1 by Mek1.

## Introduction

In mitotically dividing cells, DNA damage such as double strand breaks (DSBs) involves potentially lethal events that must be repaired to maintain the integrity of the genome. The most accurate and conservative way to repair such breaks is by homologous recombination, in which the conserved recombinase Rad51 binds to resected single stranded ends on either side of a break and then preferentially utilizes the sister chromatid as the template for repair [1–3]. In meiosis, DSBs are programmed to occur primarily in preferred regions of the genome called “hotspots” using a highly conserved meiosis-specific, topoisomerase-like protein, Spo11 [4, 5]. These breaks are then used to create crossovers (COs) between the non-sister chromatids of homologous chromosomes. Such COs, in combination with sister chromatid cohesion, serve to physically connect homologs, thereby allowing their proper orientation and segregation at the first meiotic division [6]. Changing the bias for repair template from sister chromatids to homologs requires meiosis-specific changes to chromosome structure, the DNA damage response and recombination proteins.

Sister chromatids condense during meiosis by forming loops of chromatin that are tethered at their bases by a structure called an axial element (AE) [6–8]. In yeast, AEs are comprised of the meiosis-specific proteins, Hop1 and Red1, as well as cohesin complexes containing the meiosis-specific kleisin subunit, Rec8 [8–11]. The “tethered loop axis model” proposes that hotspot sequences are brought to the axes where Spo11-mediated DSB cleavage occurs [7, 8, 12–14]. DSB formation and resection activate the Mec1/Tel1 checkpoint kinases, resulting in recruitment of the meiosis-specific kinase, Mek1, to the axes where it is activated by autophosphorylation [15–17]. Mek1 kinase activity is required for the meiotic recombination checkpoint that monitors the progression of DSB repair and prevents entry into the meiotic divisions until repair is complete [17, 18], as well as for the preferential repair of DSBs using homologs [19–21]. Recently, Mek1 was found to regulate the IH CO/non-crossover (NCO) decision by promoting the phosphorylation of the C-terminus of the transverse filament protein, Zip1, by the conserved cell cycle kinase, Cdc7-Dbf4 (DDK)[22].

Many organisms such as yeast and mammals use recombination to form stable associations between the AEs of homologous chromosomes, resulting in the insertion of a meiosis-specific transverse filament protein to create a tripartite structure called the synaptonemal complex (SC) [6]. IH bias in these organisms requires both Rad51 and the meiosis-specific recombinase, Dmc1 [23]. Rad51 and Dmc1 bind to the single stranded 3’ ends created by resection of DSBs to form nucleoprotein filaments. Loading of Dmc1 onto the ends of DSBs is promoted by Rad51, but the organization of the proteins at each end of a DSB may vary in different organisms [24, 25]. Whereas in plants asymmetric loading of Dmc1 and Rad51 to different ends of a DSB has been observed, in yeast, high resolution microscopy has revealed that both ends of the break contains short tracts of Dmc1 and Rad51 [25, 26]. The latter result is consistent with biochemical experiments showing that Rad51 is an accessory factor for the strand exchange activity of Dmc1 [27]. In both plants and yeast, the presence of the Rad51 protein, but not its strand exchange activity is necessary for IH bias. Deletion of *RAD51*, as well as mutations in genes encoding proteins important for forming Rad51-ssDNA filaments such as *RAD52* and the Shu complex, are defective in IH bias [28–30]. Furthermore *rad51* mutants in yeast and plants specifically defective in strand exchange exhibit wild-type (WT) levels of both IH and intersister (IS) recombination [27, 31]. In contrast, yeast cells lacking *DMC1* arrest with unrepaired, resected DSBs as a result of triggering the meiotic recombination checkpoint [32, 33].

Rad51 and Dmc1 strand exchange activity is stimulated by the paralogous co-factors, Rad54 and Rdh54/Tid1, respectively [23, 34–37]. Some functional redundancy can occur during meiosis, however, as *rad54Δ rdh54Δ/tid1Δ* diploids exhibit a more severe phenotype than either single mutant [38]. The fact that Rad51 is localized to DSBs in *dmc1Δ* mutants, but there is no repair, indicates that Rad51 activity is inhibited [24]. One way of downregulating Rad51 is to interfere with Rad51-Rad54 complex formation. The primary way this is accomplished is by binding of the meiosis-specific Hed1 protein to Rad51 [39, 40]. In addition, phosphorylation of Rad54 threonine 132 by Mek1 helps prevent Rad51-mediated DSB repair in the absence of *DMC1* [41]. Although the bulk of repair in *dmc1Δ hed1Δ* diploids occurs using sister chromatids, Mek1 kinase activity promotes some IH repair, resulting in the formation of crossovers and some viable spores [41, 42]. In contrast, removing one or both of these constraints on Rad51 has very little effect in diploids containing *DMC1* [40–43].

Inhibition of Mek1 kinase activity results in IS recombination in both *DMC1* and *dmc1Δ* strains, suggesting that *MEK1* is required for down regulation of Rad51 as well as promoting Dmc1-mediated IH strand invasion [19, 20, 44]. It was not clear, however, why inactivation of Mek1 should affect Hed1 repression of Rad51. This work resolves this conundrum by showing that Hed1 is a direct substrate of Mek1 and that phosphorylation of Hed1 contributes to the down regulation of Rad51 activity in *dmc1Δ* diploids by stabilizing the Hed1 protein. We propose that Mek1 inhibits Rad51 by coordinately phosphorylating Rad54 and Hed1, thereby decreasing the formation Rad51-Rad54 complexes.

## Results

### Hed1 is phosphorylated during meiosis

**Fig 1:**
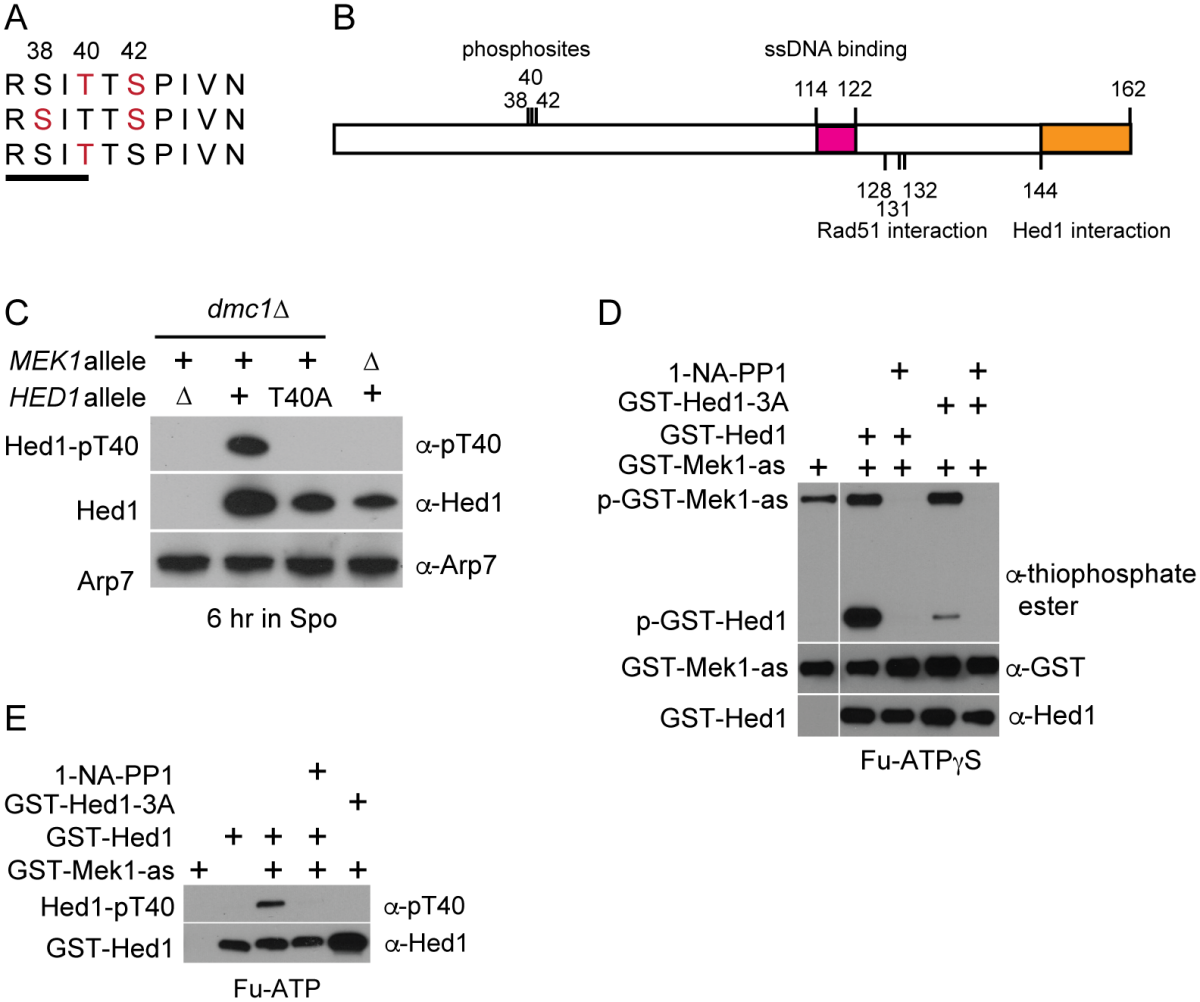
Hed1 T40 is a direct target of Mek1 phosphorylation. (A) Alignment of Hed1 phosphorylated amino acids (indicated in red) detected by MS analysis of phosphopeptides isolated after induction of *NDT80* in ySZ07. Numbers indicate amino acid positions. The line indicates the Mek1 consensus, RXXT. (B) Relationship of phosphosites to functional domains of Hed1 determined by [48]. The pink and orange boxes indicate domains required for ssDNA binding and Hed1 interaction, respectively. (C) Specificity of the Hed1 α-pT40 antibody for Hed1 phosphorylated on threonine 40. The *dmc1Δ hed1Δ* (NH942), *dmc1Δ* (NH942::pNH302^2^), *dmc1Δ hed1-T40A* (NH942::pNH302-T40A^2^) and *mek1Δ* (NH729) diploids were incubated in Spo medium for 6 hr at 30°C and probed with α-Hed1 antibodies to detect total Hed1 protein or α-pT40 antibodies to detect Hed1 phosphorylated on T40. Arp7 was used as loading control [49]. (D) Kinase reactions containing GST-Mek1-as, furfuryl (Fu)-ATPγS and recombinant GST-Hed1 or GST-Hed1-3A (both purified from *E*. *coli*) were fractionated by sodium dodecyl-sulfate polyacrylamide gel electrophoresis (SDS-PAGE). Phosphorylation was detected using the semi-synthetic epitope system [50]. In this assay, GST-Mek1-as specifically transfers thiophosphates onto its substrates using Fu-ATPγS. Reaction with *p*-nitrobenzylmesylate (PNBM) converts the thiophosphates into epitopes that are recognized by a thiophosphate ester monoclonal antibody. The vertical white line indicates the juxtaposition of non-adjacent lanes from the same gel. The horizontal white lines indicate the same samples fractionated on different gels and probed with the indicated antibodies. GST-Mek1-as and GST-Hed1 were detected using α-GST and α-Hed1 antibodies, respectively. (E) *In vitro* phosphorylation of Hed1 T40 by GST-Mek1-as. Kinase reactions were performed as in Panel D except that Fu-ATP was used and Hed1 phospho-T40 was detected using the α-pT40 antibodies.

To identify proteins phosphorylated during meiosis, diploid cells were arrested in pachytene and then synchronously induced to proceed through the meiotic divisions using a conditional allele of the meiosis-specific transcription factor, *NDT80* [45, 46]. Whole cell extracts were generated from cells taken at timepoints indicative of either Meiosis I or Meiosis II and the proteins digested with trypsin. Phosphopeptides were enriched using immobilized metal affinity chromatography and analyzed by mass spectrometry (MS) as described in [47]. Given the timing, an unexpected phosphoprotein detected in this experiment was Hed1, which down-regulates Rad51 during meiotic prophase. Multiple phosphorylated species of the same peptide were detected which identified three phosphosites on Hed1, S38, T40 and S42 (Fig 1A). This cluster of phosphosites is located in the N-terminus of Hed1 and does not overlap at the primary sequence level with Hed1 domains that are required for Rad51 interaction, Hed1 self assembly or single strand (ss) DNA binding (Fig 1B)[48].

### Hed1 is a direct substrate of Mek1

**Fig 2:**
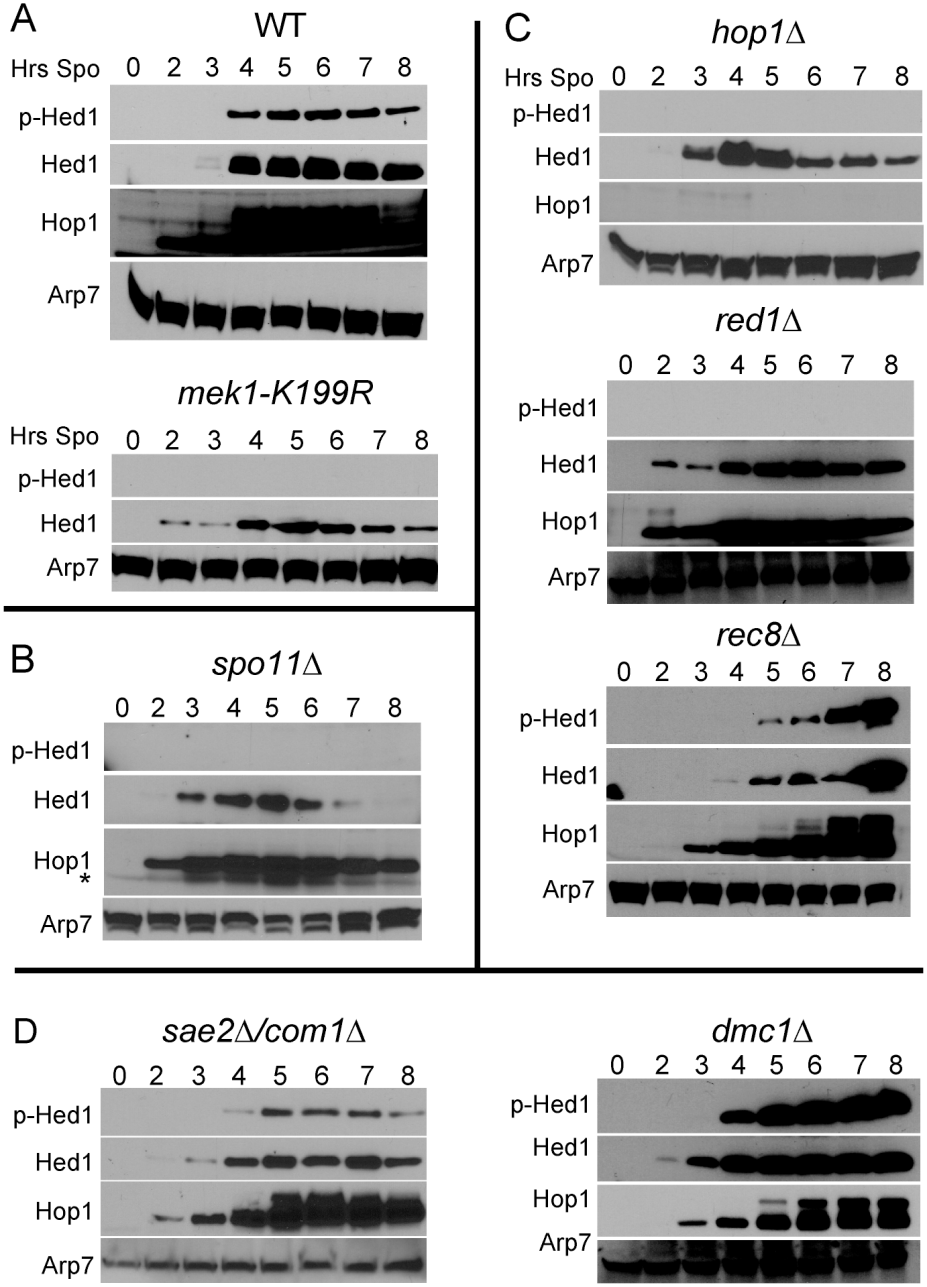
Genetic requirements for Hed1 T40 phosphorylation. (A) Hed1 T40 phosphorylation in WT and *mek1-K199R* diploids. Meiotic timecourses from WT (NH144) and *mek1-K199R* (YTS1::pLP36) diploids were probed with α-Hed1 and α-pT40 antibodies. The WT extract was also probed with α-Hop1 antibodies to detect mobility shifts indicative of DSB formation [20]. (B) Hed1 phosphorylation in the absence of DSBs. A timecourse from a *spo11Δ* diploid (NH2303) was probed as in Panel A. (C) Hed1 phosphorylation in mutants defective in various AE components. Timecourses from *hop1Δ* (DW10::pRS306^2^), *red1Δ* (YTS3) and *rec8Δ* (NH746) diploids were probed as in Panel A. (D) Hed1 phosphorylation in mutants defective in different steps of meiotic recombination. Meiotic timecourses from diploids containing *sae2/com1Δ* (NH1054) and *dmc1Δ* (NH792) diploids were probed as in Panel A.

Hed1 T40 is contained within the Mek1 consensus site, RXXT, defined both by screening peptide libraries and examination of *in vivo* substrates of the kinase (Mek1 T327, Rad54 T132 and Histone H3 T11), raising the possibility that Mek1 is the kinase that directly phosphorylates Hed1 [16, 20, 51, 52]. To create a biochemical probe specific for Hed1 T40 phosphorylation, phosphospecific antibodies (called α-pT40) were generated using a peptide from Hed1 containing phosphorylated T40 (See Materials and Methods). The α-pT40 antibodies produced a signal when used to probe WT Hed1, but not Hed1-T40A, despite the fact that more Hed1-T40A protein was present compared to WT (Fig 1C). Hed1 T40 phosphorylation was eliminated in strains homozygous either for *mek1Δ* or a catalytically inactive version of *MEK1*, *mek1-K199R* (Figs 1C and 2A) [20, 53]. Phosphorylation of Hed1 T40 is therefore dependent upon Mek1 kinase activity.

Analog sensitive (*as*) kinases have enlarged ATP binding pockets that allow both the specific inhibition of a kinase *in vivo* using purine analogs, as well as the detection of direct kinase substrates *in vitro* using ATP analogs [54]. The *mek1-as* allele encodes an analog-sensitive version of Mek1 that can be inhibited by addition of 1-NA-PP1 to the sporulation medium [55]. The semi-synthetic epitope system combines partially purified GST-mek1-as with Furfuryl-(Fu)-ATPγS to test whether phosphorylation of a substrate is direct [50, 56]. Thiophosphorylation of substrate proteins by GST-mek1-as is converted to an affinity tag by a chemical reaction that creates an epitope that can be detected by a commercially available antibody. This approach was previously used to show that Mek1 and Rad54 are both directly phosphorylated by Mek1 [41].

To test whether Mek1 phosphorylation of Hed1 is direct, GST-Hed1 was purified out of *E*. *coli* and added to kinase reactions containing GST-Mek1-as and Fu-ATPγS. GST-Mek1-as autophosphorylation was observed, as well as phosphorylation of GST-Hed1 (Fig 1D). Phosphorylation of both proteins was dependent upon Mek1 kinase activity, as addition of 1-NA-PP1 eliminated the signals. The hypothesis that Mek1 phosphorylates a region on Hed1 containing T40, T41 and S42 was tested using GST-Hed1-3A, in which T40, T41 and S42 were all substituted with alanine. GST-Hed1-3A behaved similarly to GST-Hed1 in biochemical assays measuring Hed1’s ability to interact with Rad51, and to inhibit Rad54-stimulated ATP hydrolysis and D-loop formation by Rad51, indicating that the mutant protein was properly folded (S1 Fig) [39]. Phosphorylation of GST-Hed1-3A was reduced compared to GST-Hed1 (Fig 1D). The residual phosphorylation was eliminated by addition of inhibitor, indicating that Mek1 can phosphorylate other amino acids on Hed1 (or GST) *in vitro*, although to a lesser extent. Taken together, these data show that Mek1 directly phosphorylates a region on Hed1 that includes T40.

To test whether Hed1 T40 specifically is a direct target of the kinase (as predicted based on the consensus), the kinase assays were repeated using GST-Mek1-as and Fu-ATP, in which a phosphate, rather than a thiophosphate, was transferred to the substrate. Phosphorylation of Hed1 T40 was then assayed using the α-pT40 antibodies. A signal was observed with GST-Hed1, but not GST-Hed1-3A, and phosphorylation was abolished by the addition of inhibitor (Fig 1E). Therefore, Hed1 T40 joins the list of *bona fide in vivo* Mek1 substrates.

### Hed1 phosphorylation is dependent upon DSBs and Mek1 activation

As expected given that Mek1 is activated by DSBs, the appearance of Hed1 T40 phosphorylation coincided with DSB-formation (indirectly indicated by phosphorylation of Hop1) and was dependent upon *SPO11* (Fig 2A and 2B) [20]. In addition, Hed1 T40 phosphorylation required *HOP1* and *RED1*, genes that encode AE proteins necessary for Mek1 activation [15, 16] (Fig 2C). In contrast, Hed1 T40 phosphorylation was not dependent upon *REC8*, consistent with the fact that Mek1 is active in *rec8Δ* mutants [19, 44] (Fig 2C). DSB resection and strand invasion were not required for Hed1 phosphorylation, as phosphorylation of Hed1 T40 was observed in an *sae2Δ/com1Δ* mutant, which makes breaks that are not resected [57, 58] and a *dmc1Δ* mutant, in which DSBs are resected but fail to undergo strand invasion [32, 59] (Fig 2D).

### Phosphorylation of Hed1 T40 promotes the meiotic checkpoint arrest triggered by *dmc1*Δ

To determine whether Mek1-mediated phosphorylation of Hed1 is functionally important, various phosphosite mutants were created. In addition to the *hed1-3A* mutant, a T40 to alanine substitution (*hed1-T40A*) was used to create a Hed1 protein that cannot be phosphorylated at this site, while a glutamic acid substitution (*hed1-T40E*) was used to mimic the negative charge conferred by phosphorylation.

Assaying *hed1* mutants for complementation of *hed1Δ* is challenging, because *hed1Δ* exhibits only a two-fold reduction in IH bias with little to no effect on spore viability [42, 43]. A more robust assay is to look at *hed1* phenotypes in the absence of *DMC1*. Because *HED1* is required to prevent Rad51-mediated repair of DSBs in *dmc1Δ* diploids [40], the prophase arrest in SK1 strains is dependent upon *HED1*. Therefore a sensitive assay for *HED1* function is meiotic progression in *dmc1Δ* diploids.

**Fig 3:**
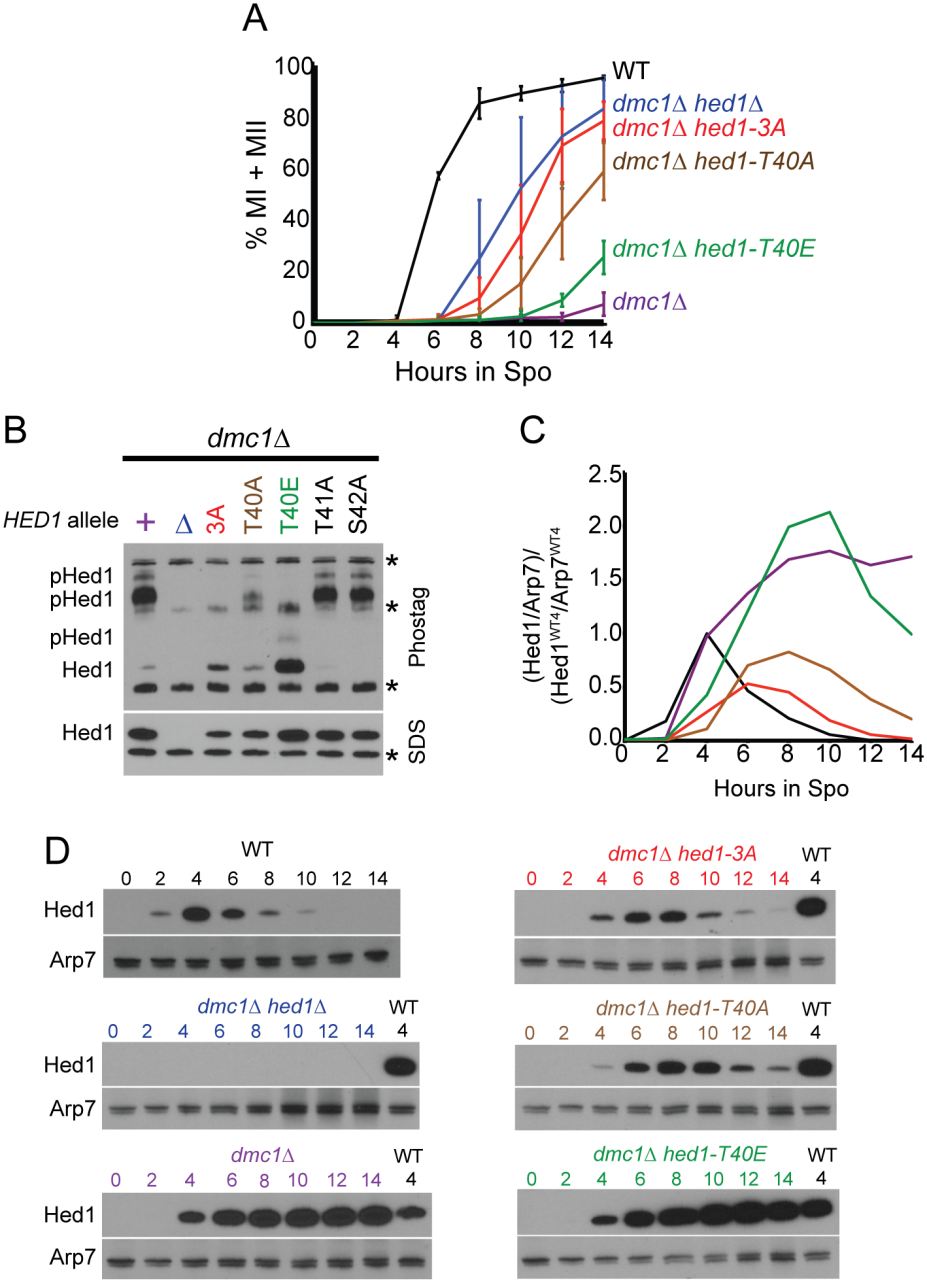
Negative charges in the Hed1 T40 region promote Hed1 function in *the* absence of *DMC1*. (A) Meiotic progression. Wild-type (NH716), *dmc1Δ* (NH942::pNH302^2^), *dmc1Δ hed1Δ* (NH942/pRS316), *dmc1Δ hed1-3A* (NH942::pNH302-3A^2^), *dmc1Δ hed1-T40A* (NH942::pNH302-T40A^2^) and *dmc1Δ hed1-T40E* (NH942::pNH302-T40E^2^) diploids were transferred to Spo medium at 30°C, fixed at various time points, stained with DAPI and the number of nuclei determined using fluorescence microscopy. Bi-nucleate cells have completed MI and tetra-nucleate cells have completed MII. Meiotic progression was plotted as the average % MI + MII from multiple timecourses. Error bars indicate the standard deviations between experiments. For WT, n = 3; *dmc1Δ*, n = 8, *dmc1Δ hed1Δ*, n = 10; *dmc1Δ hed1-3A*, n = 8; *dmc1Δ hed1-T40A*, n = 9, *dmc1Δ hed1-T40E*, n = 5. The color code shown in Panel A is used throughout the paper. The data used for all of the graphs in this paper are contained in S1 Data. (B) Mobility shift of different Hed1 mutant proteins. The *dmc1Δ hed1Δ* diploid, NH942, carrying either vector alone (*Δ*) or two copies of pNH302 (*URA3 HED1*) or its indicated derivatives was transferred to Spo medium for 4 hr. Extracts were fractionated on 10% SDS-polyacrylamide gels with 75 μM Phostag and 75 μM MnCl_2_ (top) or without Phostag (bottom), transferred to membranes and probed with α-Hed1 antibodies. “Hed1” indicates the non-phosphorylated protein, while “p-Hed1” indicates phosphorylated Hed1. Asterisks indicate non-specific bands. (C) Quantification of Hed1 protein levels the timecourses shown in Panel D. After the proteins were transferred, the membrane was cut horizontally and the bottom half was probed with α-Hed1 while the top was probed with Arp7. For each sample, the Hed1 protein was normalized to the Arp7 from that lane. For comparison between different blots, extract from the 4 hr WT timecourse (Hed1^WT4^) was included with each timecourse and each Hed1/Arp7 ratio was then divided by the Hed1^WT4^/Arp7^WT4^ ratio from that gel. (D) Protein extracts from one of the set of timecourses shown in Panel A were probed with α-Hed1 or α-Arp7 antibodies. This experiment was repeated twice with similar results.

Consistent with the literature, nearly all *dmc1Δ* cells arrested as mononucleate cells in prophase (Fig 3A) [32]. In contrast, the *dmc1Δ hed1Δ* diploid exhibited robust meiotic progression, with greater than 80% of cells completing either MI or MII. Progression was delayed four hours compared to WT, however, indicating that Rad51-mediated repair is less efficient than Dmc1-mediated repair (Fig 3A)[42]. The *dmc1Δ hed1-3A* mutant was delayed approximately 1.5 hrs compared to *dmc1Δ hed1Δ*, but ultimately reached the same level of progression (Fig 3A). These results are consistent with phosphorylation of Hed1 suppressing Rad51-mediated DSB repair during meiosis.

To look specifically at the function of T40 phosphorylation, meiotic progression of *dmc1Δ hed1-T40A* was compared to the phosphomimetic allele, *hed1-T40E*. The *dmc1Δ hed1-T40A* mutant exhibited a significant level of meiotic progression, demonstrating that the inability to phosphorylate T40 creates a defect in Hed1 function (Fig 3A). This mutant was delayed approximately 2 hours longer than *dmc1Δ hed1-3A*, indicating a more WT phenotype, but > 60% of the cells still proceeded through either MI or MII by 14 hrs in contrast to the *dmc1Δ*. The residual activity observed for *hed1-T40A* compared to *hed1-3A* is likely due to phosphorylation at other positions that can be detected by mobility shift experiments in the presence of Phostag. Phostag is a commercially available reagent that exacerbates the mobility shift of phosphorylated proteins using SDS-PAGE [60]. The Hed1-T40A mutant protein exhibited a mobility shift, while the Hed1-3A mutant did not, indicating that phosphorylation of T41 and/or S42 can occur in the absence of T40 phosphorylation (and that S38 phosphorylation does not contribute to the shift) (Fig 3B). The mutant with a phenotype closest to *HED1* is *hed1-T40E*. In this mutant, meiotic progression was delayed over three hours compared to *dmc1Δ hed1Δ*, with only ~20% of the cells having entered into the meiotic divisions by 14 hrs (Fig 3A). The fact that *dmc1Δ hed1-T40E* is more similar to WT than *dmc1Δ hed1-T40A* provides genetic evidence that a negative charge at the T40 position is important for Hed1 function. However, approximately 50% of the *dmc1Δ hed1-T40E* cells eventually sporulated, compared to 0% sporulation for *dmc1Δ* (S1 Table), indicating that the *hed1-T40E* phosphomimic is still not as functional as *HED1*.

One explanation for the leaky phenotype of *hed1-T40E* is that glutamic acid only provides one negative charge, in contrast to the two negative charges provided by phosphorylation. A smaller fraction of the Hed1-T40E protein was shifted in the Phostag gel and the shift was not a large as was observed for Hed1-T40A (Fig 3B) suggesting that glutamic acid may inhibit phosphorylation of T41 and/or S42. Furthermore, although no phosphorylation defect was observed for the Hed1-T41A or S42A proteins, a low level of sporulation was observed for these mutants in the *dmc1Δ* background, indicating a partially mutant phenotype (Fig 3B)(S1 Table). This was also true for *dmc1Δ hed1-T38A* diploids, even though phosphorylation of T38 does not contribute to the shift observed on Phostag gels (given that no mobility shift is observed in for Hed1-3A) (Fig 3B) (S1 Table). We conclude that Hed1 T40 is the primary functional phosphorylation site on Hed1, but that the ability to fully inhibit Rad51 requires phosphorylation of nearby amino acids to make a negatively charged patch.

### Phosphorylation of Hed1 T40 promotes Hed1 protein stability

One way that Mek1 could regulate Hed1’s ability to repress Rad51 activity would be if phosphorylation inhibited Hed1 degradation. In fact, total Hed1 protein levels appear reduced in *mek1-K199R* and *spo11Δ* compared to WT (Fig 2A and 2B). To test this idea more quantitatively, timecourses were performed in which steady state protein levels were analyzed for the different *hed1* mutants. This experiment was done in the *dmc1Δ* background so that *HED1* function (i.e. its ability to prevent meiotic progression) could be correlated with the amount of Hed1 protein. The amount of Hed1 in each lane was normalized to the amount of Arp7 in the extract. To normalize between gels, the same amount of extract from a 4 hr WT timecourse was included on every gel. In the WT strain, Hed1 protein peaked at 4 hrs and was gone by 12 hours after transfer to Spo medium (Fig 3C and 3D). In contrast, Hed1 protein levels remained high in the *dmc1Δ* mutant, a situation in which Hed1 T40 phosphorylation persisted (Figs 2D, 3C and 3D). There was an excellent correlation between the amount of meiotic progression, the presence of a negative charge and protein stability (Fig 3). In the *dmc1Δ* background, the Hed1-3A protein was the least abundant, followed by Hed1-T40A. While the Hed1-T40E protein reached peak levels higher than the WT protein, the levels began to drop after 10 hours, after which a small fraction of cells progressed through the meiotic divisions (Fig 3). These results support the idea that Mek1 phosphorylation promotes Hed1 repression of Rad51 activity by inhibiting degradation of Hed1.

### Phosphorylation of Hed1 T40 delays DSB repair at the *HIS4-LEU2* hotspot in *dmc1*Δ diploids

**Fig 4:**
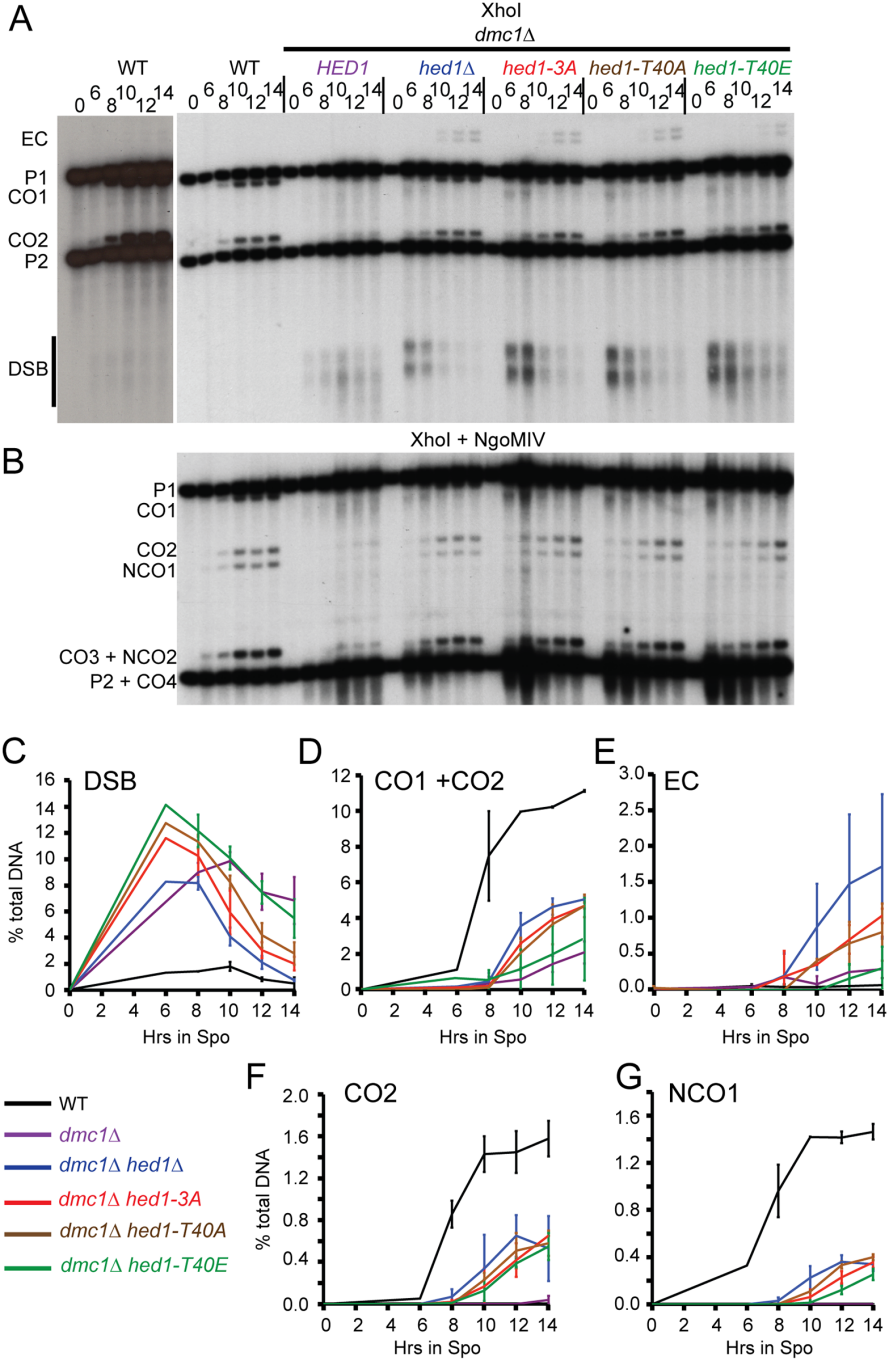
Physical analysis of recombination at the *HIS4-LEU2* hotspot. (A) DNA was isolated at the indicated timepoints, digested with XhoI and probed as described in [59] to detect DSBs and COs. For the WT, a darker exposure is shown on the left to show the DSBs. “EC” indicates bands resulting from ectopic recombination [42]. (B) DNA from the same time course shown in A was cut with XhoI and NgoMIV to detect CO and NCO recombinants at the *HIS4-LEU2* hotspot. (C) Quantification of DSBs from two independent timecourses, one of which is shown in A. Error bars indicate the range. There was no 6 hr timepoint in the second timecourse. (D) Quantification of CO1 +CO2 as in Panel C. (E) Quantification of ECs as in Panel C. (F) Quantification of CO2 from two independent timecourses, one of which is shown in B. (G) Quantification of NCO1 as in Panel F.

The delay in meiotic progression in the various *dmc1Δ hed1* mutants correlates well with the kinetics of DSB repair at the *HIS4-LEU2* hotspot. This hotspot, located on chromosome III, is flanked by XhoI restriction sites [59]. Both DSBs and CO bands can be detected by Southern blot analysis of one-dimensional agarose gels using XhoI-digested DNA and a probe for this region as described in [61]. As expected, DSBs in the *dmc1Δ* strain accumulated to higher than WT levels with very little repair (i.e. little disappearance at the later timepoints) (Fig 4A and 4C) [32]. The peak DSB levels of all of the *dmc1Δ hed1* mutants were greater than WT and went in increasing order from *hed1Δ*, *hed1-3A*, *hed1-T40A* and *hed1-T40E* (Fig 4A and 4C). The high levels of DSBs could be due to inefficient repair and/or the creation of new DSBs due to a lack of IH engagement [62]. In addition, there was a qualitative difference between the *dmc1Δ* DSBs and those in the *dmc1Δ hed1* mutants. At later timepoints the *dmc1Δ* breaks were highly resected, which was not the case for the *dmc1Δ hed1* mutants (Fig 4A). This may be because DSBs in these mutants are turning over and therefore are not present long enough to become hyper-resected. Despite the differences in the kinetics of DSB repair, all of the mutants exhibited approximately 70% viable spores (S1 Table).

Previous studies have shown that Rad51-mediated recombination is able to generate IH COs in *dmc1Δ hed1Δ* mutants [40, 42]. All of the *dmc1Δ hed1* mutants exhibited delayed and reduced levels of COs at the *HIS4-LEU2* hotspot, with the *hed1-T40E* mutant exhibiting the biggest delay (Fig 4A, 4B, 4D and 4F). In addition, ectopic recombinants (EC) were observed in *dmc1Δ hed1* but not WT (Fig 4A and 4E) [42]. Digestion of the DNA with XhoI and NgoMIV allows the detection of NCO products as well as COs [63]. Rad51-mediated recombination resulted in reduced levels of NCOs, again with *dmc1Δ hed1-T40E* exhibiting the greatest delay. We conclude phosphorylation of Hed1 T40 is required, but not sufficient, for the down-regulation of Rad51-mediated repair in *dmc1Δ* diploids.

### Rad51-mediated recombination exhibits reduced IH bias regardless of Hed1 phosphorylation

**Fig 5:**
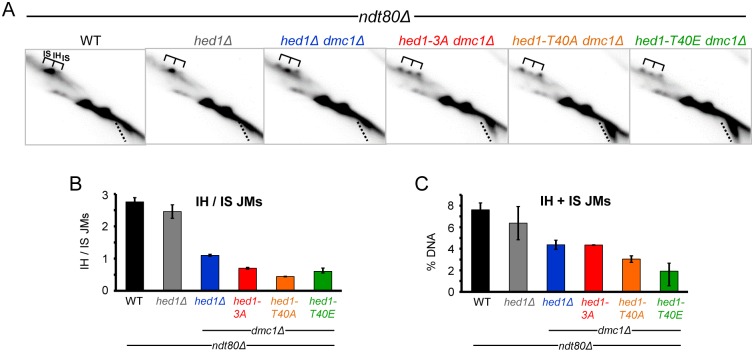
JM formation in *dmc1*Δ *ndt80*Δ strains containing different alleles of *HED1*. *ndt80Δ*, (NH2188), *hed1Δ ndt80Δ* (NH2223), *dmc1Δ hed1Δ ndt80Δ* (NH2166), *dmc1Δ hed1-3A ndt80Δ* (NH2166::pNH302-3A^2^) and *dmc1Δ hed1-T40A ndt80Δ* (NH2166::pNH302-T40A^2^) and *dmc1*Δ *hed1-T40E ndt80*Δ (NH2166::pNH302-T40E^2^) diploids were incubated in Spo medium for seven hours. The DNA was crosslinked using psoralen and digested with XhoI. Southern blots of two-dimensional gels were probed as described in [61]. A. Two-D gels. Brackets indicate the positions of the IH and two IS JMs. Dotted lines indicate DSBs. B. Quantification of JMs from two separate experiments, one of which is shown in Panel A. Error bars indicate the range. C. Quantification of the percentage of JMs (IH + IS) in two separate experiments, one of which is shown in Panel A.

The relative amounts of IH and IS joint molecules (JMs) at the *HIS4-LEU2* hotspot can be determined by probing Southern blots of XhoI-digested DNA that has been fractionated on two-dimensional gels, thereby separating different species based on their size and shape [59]. JM analysis was performed at the *HIS4-LEU2* hotspot in diploids deleted for the meiosis-specific transcription factor *NDT80*. *NDT80* is required for the induction of the polo-like kinase, *CDC5*, which in turn is sufficient to trigger HJ resolution [49, 64]. After transfer to Spo medium for seven hours, DNA was cross-linked with psoralen to prevent branch migration of the JMs, digested with XhoI and fractionated in two dimensions to resolve IH JMs from the two IS JMs. Previous work showed that Rad51-mediated recombination in *dmc1Δ hed1Δ* diploids is defective in partner choice, exhibiting a 25-fold decrease in the IH/IS JM ratio compared to WT [42]. In our hands the IH:IS ratio was also reduced in *hed1Δ dmc1Δ* diploids compared to WT and *hed1Δ*, but to a lesser extent than previously reported (Fig 5A and 5B).

Rad51-mediated recombination exhibits a bias for intersister recombination both in vegetative and *dmc1Δ hed1Δ* meiotic cells [1, 42]. A decrease in IH bias was observed for the *hed1-3A*, *T40A* and *T40E* mutants, regardless of charge (Fig 5A and 5B). The absolute number of JMs reflects the ability of the Hed1 mutant protein to inhibit Rad51 activity. The *hed1-3A* mutant exhibited the most JMs of the point mutants while *hed1-T40E* had the least (Fig 5C). Because the phosphomimetic *hed1-T40E* mutant did not increase the IH:IS ratio relative to the *T40A* mutant, but instead simply reduced the total number of JMs, we conclude that phosphorylation of Hed1 decreases IS recombination by down-regulating Rad51 strand invasion activity, (which is more likely to occur between sister chromatids) but does not play a direct role in promoting IH bias during meiosis.

### Rad51-mediated recombination is inefficient in highly polymorphic hybrid yeast diploids

Chromosome III, where the *HIS4-LEU2* hotspot is located and which was analyzed in detail by Lao et al. (2013), is one of the smallest chromosomes and may not be representative of the genome as a whole. The phenotypic characterization of Rad51-mediated recombination in *dmc1Δ hed1Δ* and *dmc1Δ hed1-3A* diploids was therefore performed on a global scale using Next Generation Sequencing (NGS) of tetrads. A hybrid diploid containing >62,000 single nucleotide polymorphisms (SNPs) was generated by mating an S288c strain to an SK1 strain [65]. The SNPs in this hybrid are distributed such that there is approximately one SNP every 200 nucleotides and can be used to determine the parental origin of DNA sequences within the four haploid chromosomes resulting from meiosis.

**Fig 6:**
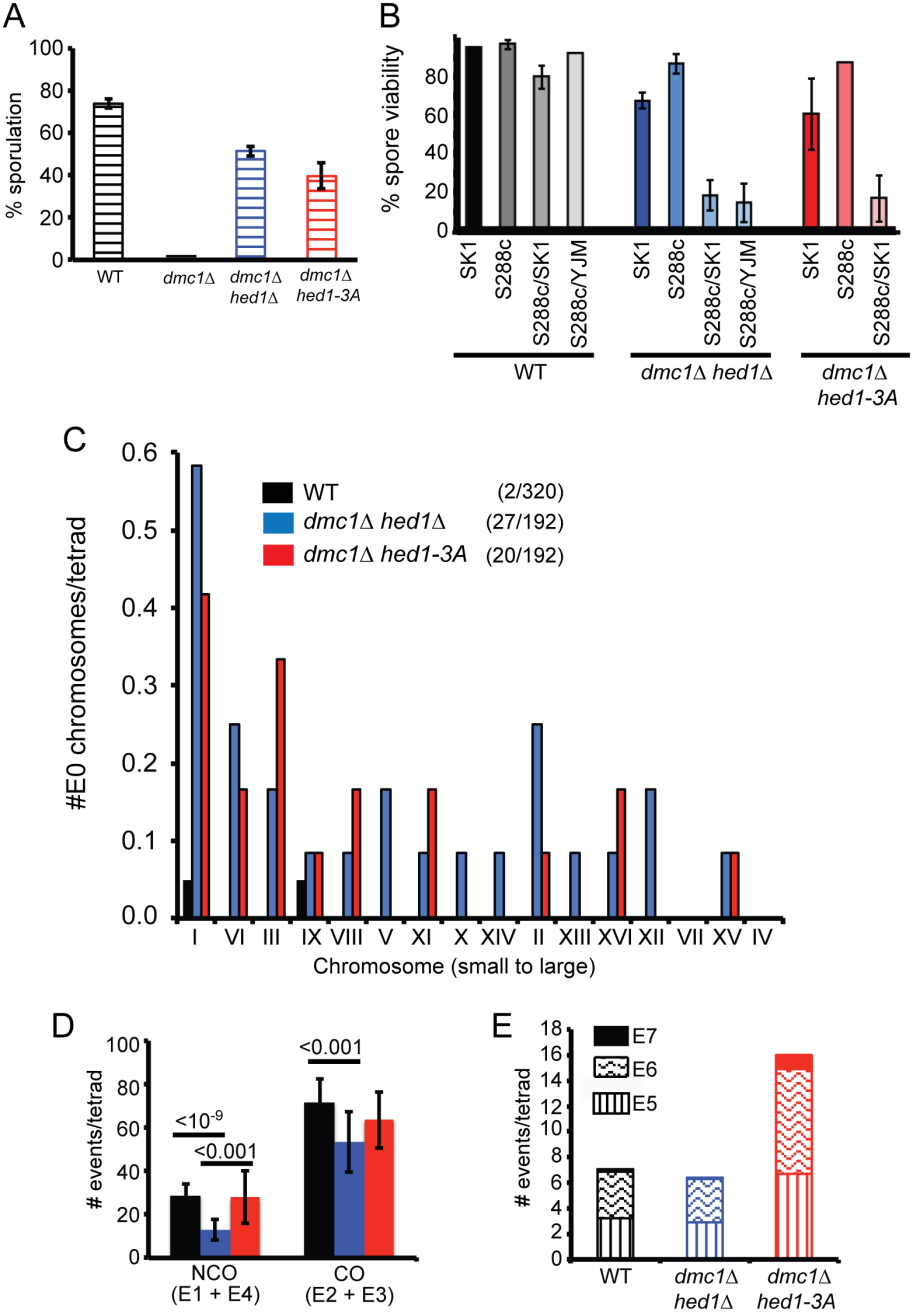
Rad51-mediated recombination in hybrid strains determined by Next Generation Sequencing of tetrads. (A) WT (AND1702), *dmc1Δ* (NH2310), *dmc1Δ hed1Δ* (NH2294) and *dmc1Δ hed1-3A* (NH2294::pNH302-3A^2^) SK1/S288c hybrid diploids were sporulated on plates for three days at 30°C and five independent colonies were assayed for the percent asci measured by light microscopy. 200 cells were counted for each colony. Error bars represent the standard deviations. (B) Spore viability was measured by dissecting a total of 93–104 tetrads. SK1 (NH716), S288c (NH2315/pRS316); SK1/S288c (AND1702), YJM789/S288c (NH1053); *dmc1Δ hed1Δ*: SK1 (NH942), S288c (NH2320/pRS316), SK1/S288c (NH2294), YJM789/S288c (NH2032) and *dmc1Δ hed1-3A*: SK1 (NH942::pNH302-3A^2^), S288c (NH2320::pNH302-3A^2^), SK1/S288c (NH2294::pNH302-3A^2^). Error bars represent the standard deviations obtained by dissection of different colonies (n = 4). (C) Distribution of non-exchange (E0) chromosomes as a function of chromosome size as determined by NGS of SK1/S288c diploids (20 tetrads for WT and 12 tetrads each for *dmc1Δ hed1Δ* and *dmc1Δ hed1-3A*). The absence of a bar indicates that all of the chromosomes for that strain had at least one CO. Numbers in parentheses indicate the number of E0 chromosomes/total number of chromosomes. Dot plots showing the distribution of E0 chromosomes in each tetrad can be found in S3 Fig. (D) Comparison of NCO and CO events/tetrad. The total number of COs (E2 + E3)/tetrad or NCOs (E1+E4)/tetrad was plotted for each genotype. Error bars indicate standard deviation between tetrads. Lines indicate statistically significant differences with the *p* values calculated using a two-tailed t-test. (E) Comparison of minority event types. The average number of E5, E6 and E7 events per tetrad for each genotype is indicated. Asterisks indicate values that are statistically significantly different from both WT and *dmc1Δ hed1Δ* (calculated using a two-tailed t-test). No significant differences were observed for any minority event type between WT and *dmc1Δ hed1Δ*. *p* values can be found in S1 Data.

The *dmc1*Δ SK1/S288c hybrid behaved similarly to a *dmc1Δ* SK1 diploid in that cells remained mononucleate due to a prophase arrest and therefore failed to sporulate (Figs 6A and S2). Both the *hed1Δ* and *hed1-3A* mutants partially relieved the *dmc1Δ* arrest, indicating that Rad51-mediated repair can occur in the hybrid. The *dmc1Δ hed1-3A* hybrid exhibited a small decrease in sporulation and meiotic progression was delayed approximately three hours compared to *dmc1Δ hed1Δ* (Figs 6A and S2). Therefore, similar to the SK1 strain background, phosphorylation of Hed1 downregulates Rad51 in the hybrid while the unphosphorylated protein retains some ability to inhibit Rad51.

Rad51-mediated recombination generated some IH COs, as evidenced by the production of viable spores in the *dmc1Δ hed1Δ* and *dmc1Δ hed1-3A* hybrids (18.3 and 16.7% respectively (Fig 6B). Notably however, spore viability in hybrid strains was significantly lower (5-fold) than that observed in *dmc1Δ hed1Δ* and *dmc1Δ hed1-3A* diploids in which both parents were derived either from the SK1 or S288c backgrounds. A similar decrease in *dmc1Δ hed1Δ* spore viability was observed with a different hybrid created by mating an S288c strain to the YJM789 background (Fig 6B). In contrast, WT hybrids exhibited high levels of viable spores. We conclude that in hybrid strains a high level of polymorphism or unknown genetic interactions is deleterious when meiotic recombination is mediated by Rad51, but not Dmc1.

### Rad51-mediated recombination does not support crossover assurance

The genomic DNA from 20 WT, 12 *dmc1Δ hed1Δ* and 12 *dmc1Δ hed-3A* tetrads obtained from the SK1/S288c hybrids was analyzed by whole genome sequencing, using an Illumina HiSeq 2500 instrument with paired-end reads of 150 X 150 bp. The recombination profiles were generated using the CrossOver (v6.3) algorithm from ReCombine (v2.1) [66] and were further refined using the GroupEvents program, kindly provided by J. Fung (University of California, San Francisco) [67]. One potential caveat with this analysis is that because spore viability was reduced in the mutants and only tetrads in which all four spores were viable were used, crossover values could be overestimated due to selection bias. Schematics of the chromosomes from all the sequenced tetrads can be found in S4 Fig.

A major difference between the tetrads derived from Rad51-mediated recombination (*dmc1Δ hed1Δ* and *dmc1Δ hed1-3A*) from those in which COs were generated by Dmc1 (WT) is the increased number of non-exchange (E0) chromosomes. For the *dmc1Δ hed1Δ* diploid, there were 16 pairs of homologs in the 12 tetrads that were sequenced, resulting in a total of 192 homolog pairs. Of these, 27 failed to sustain a CO, a significant increase over the two E0 homologs observed out of the 320 homolog pairs assayed for the WT (Figs 6C and S3) (χ^2^, *p* <0.0001). The increase in E0 chromosomes observed for the *dmc1Δ hed1Δ* mutant strain was not due simply to the high number of SNPs present in the hybrid, as a significant increase in non-exchange chromosome IIIs was previously observed using a genetically marked homozygous SK1 *dmc1Δ hed1Δ* diploid [42]. A significant increase in E0 chromosomes was also observed for the *dmc1Δ hed1-3A* diploid (Figs 6C and S3) (χ^2^, *p* <0.0001). In both mutants, several tetrads exhibited more than one pair of E0 chromosomes (S3 Fig). While this could represent distributive segregation of the non-exchange chromosomes, the decreased spore viability of the mutants suggests that selection bias is occurring for those tetrads in which the randomly segregating non-exchange chromosomes happened to segregate to opposite poles.

Genetic interference is the phenomenon by which a crossover in one interval inhibits the formation of a crossover in an adjacent interval [68]. Interference values can be calculated from genome wide sequencing data by measuring the distance between COs to generate a value called γ [69]. A γ value of 1 indicates no interference, while values >1 indicate positive interference. Both *dmc1Δ hed1Δ* and *dmc1Δ hed1-3A* exhibited γ values lower than that observed in WT (1.25 and 1.46 vs 1.99), but higher than 1, indicating a partial defect in interference, consistent with published results based on genetic analysis of chromosome III [42].

Although a reduction in interference could explain the increased frequency of small E0 chromosomes, it is notable that large chromosomes without COs were also observed in both the *dmc1Δ hed1Δ* and *dmc1Δ hed1-3A* tetrads (Figs 6C and S3). Chromosomes that lacked COs in the *dmc1Δ hed1Δ* and *dmc1Δ hed1-3A* diploids also exhibited a significant reduction in NCOs. Out of 27 E0 chromosomes from the *dmc1Δ hed1Δ* mutant, 26 also lacked NCOs, while 17 out of 20 E0 chromosomes from *dmc1Δ hed1-3A* exhibited no NCOs. The percentage of E0 chromosomes without NCOs in *dmc1Δ hed1Δ* and *dmc1Δ hed1-3A* was higher than that observed for WT (96%, 85% and 50%, respectively), but the low number of E0 chromosomes precludes a definitive conclusion for the WT. These results suggest that some chromosomes have little to no stable IH interactions when meiotic recombination is mediated by solely by Rad51.

The GroupEvents software developed by [67] breaks down recombination events into seven different categories. There are two types of NCOs: E1 and E4 designate simple and discontinuous NCOs, respectively. COs are also divided into two classes: E2 and E3 which indicate simple COs without and with discontinuous gene conversion, respectively. In addition there are three “minority classes” in which there are at least two COs, NCOs or COs and NCOs within a 5 kb region. The minority class in which all of the events occur between the same two chromatids is called E5, while tetrads with events involving either three or four chromatids are labeled E6 and E7, respectively. Schematics of the minority events from all WT, *dmc1Δ hed1Δ* and *dmc1Δ hed1-3A* can be found in S5, S6 and S7 Figs, respectively.

No effect on the total number of NCOs and COs was observed between *dmc1Δ hed1-3A* and WT, while both types of recombination events were reduced in *dmc1Δ hed1Δ* (Fig 6D). The fact that the *dmc1Δ hed1-3A* diploid exhibited an increased number of non-exchange chromosomes, despite having WT or higher levels of COs, indicates that the crossover assurance defect previously observed by Lao et al. (2013) in their genetic analysis of Chromosome III is true for the entire genome.

In a previous study analyzing WT cells, multichromatid E6 and E7 events were relatively rare, representing only 4.4% of the total events [67]. This was true for our WT and *dmc1Δ hed1Δ* tetrads as well, which exhibited 3.5% and 4.8% E6 + E7 events, respectively (Fig 6E). For *dmc1Δ hed1-3A*, a statistically significant increase in all three minority events was observed compared to WT and *dmc1Δ hed1Δ* (E6 + E7 = 8.6%) (Fig 6E). One explanation for the increased number of multichromatid events is that the necessity of removing or inactivating the Hed1 protein for Rad51 to function allows more time for IH recombination intermediates to become established which may require multiple rounds of strand invasion. Another possibility is that the delay in repair and the consequent lack of IH engagement results in an increased number of closely spaced DSBs [62]. In contrast, in *dmc1Δ hed1Δ* diploids, the major impediment to Rad51 activity, Hed1, has been removed so that breaks are repaired more rapidly.

## Discussion

### Hed1 T40 is a direct substrate of Mek1

Because unrepaired DSBs are potentially lethal to a cell, the deliberate introduction of ~160 DSBs during meiosis [70] requires that repair of the breaks be carefully monitored, with the added complication that repair occur preferentially between homologs. Towards this end, hotspot sequences are recruited to the chromosome axes, where Mek1, instead of Rad53, is locally activated to mediate the meiotic recombination checkpoint, IH bias and the formation of IH crossovers distributed by interference [17–19, 22]. How Mek1 mediates these various processes requires the identification and characterization of its substrates.

**Fig 7:**
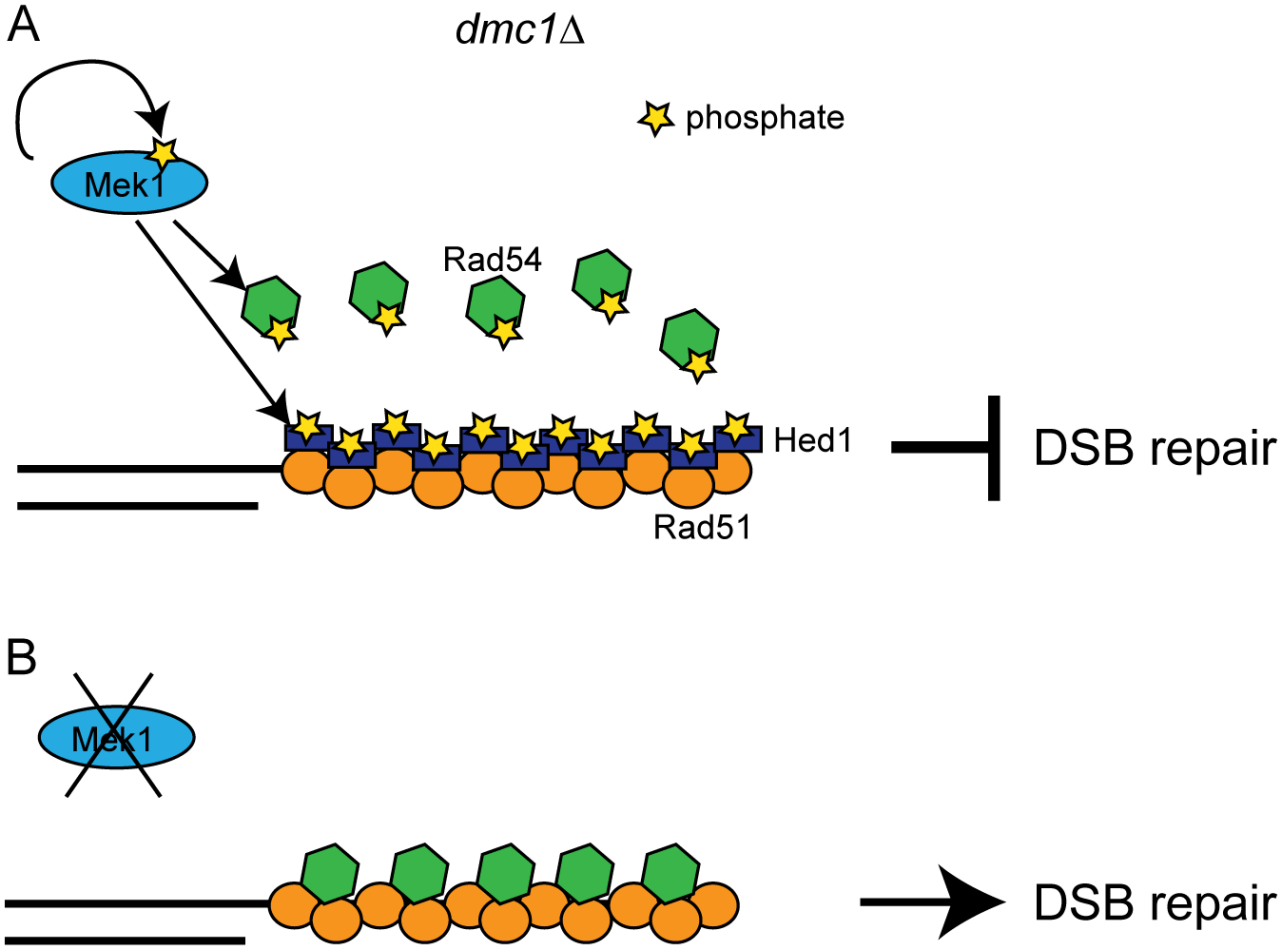
Mek1 inhibits Rad51 activity in *dmc1Δ* diploids by phosphorylation of both Rad54 and Hed1. (A) In the absence of *DMC1*, active Mek1 phosphorylates both Rad54 and Hed1, as well as itself. Rad54-Rad51 complex formation and strand invasion activity are prevented because phosphorylated Rad54 has decreased affinity for Rad51 and Hed1 bound to Rad51 excludes Rad54. Mek1 remains constitutively active by autophosphorylation. This is a cartoon and not meant to indicate any particular stoichometries between the proteins. (B) Inactivation of Mek1 results in degradation of Hed1 and dephosphorylation of Rad54, thereby allowing Rad51-Rad54 complex formation and DSB repair.

Prior to this work, three *in vivo* substrates of Mek1 were known. First, Mek1 is activated by autophosphorylation of threonine 327 in the activation loop of the kinase [16] (Fig 7A). Second, threonine 11 of Histone H3 is phosphorylated by Mek1, but the function of this modification has yet to be determined [51]. Third, Mek1 phosphorylation of Rad54 T132 reduces the affinity of Rad54 for Rad51, helping to downregulate the recombinase during meiosis [41] (Fig 7A). In addition, Mek1 kinase activity is required to allow DDK phosphorylation of Zip1 and generation of COs that are distributed throughout the genome by interference but the mechanism for how this occurs is yet unknown. Finally, Mek1 has been proposed to counteract sister chromatid cohesion nearby DSBs by phosphorylation of an unknown substrate(s) [19].

This work demonstrates that Hed1 is also a direct substrate of Mek1 (Fig 7A). Mek1 directly phosphorylates T40, consistent with T40 being part of the RXXT Mek1 consensus phosphorylation sequence. A single negative charge provided by substituting glutamic acid for T40 substantially represses Rad51 activity in *dmc1*Δ diploids, demonstrating that phosphorylation of this site makes a major contribution to Hed1 function. However, the fact that peptides containing multiple phosphorylation sites were observed by MS, and that the *hed1-T40A* mutant is not as defective as the *hed1-3A* triple mutant, indicates that a negatively charged “patch” is likely used *in vivo* to most effectively down regulate Rad51. While the Hed1 mobility shift is completely eliminated by inhibition of Mek1, whether Mek1 is the direct kinase for these other sites has not yet been established.

### Mek1 regulates Rad51 activity by maintaining steady state levels of Hed1 protein

How does Mek1 phosphorylation of Hed1 promote inhibition of Rad51 function? Phosphorylation by Mek1 *per se* is not required for Hed1 to suppress Rad51, as indicated by the fact that ectopic expression of *HED1* in vegetative cells (where there is no Mek1) makes cells sensitive to the DNA damaging agent, MMS by excluding binding of Rad54 to DSBs [39, 40]. Furthermore, IS recombination is increased to a greater extent in *hed1Δ mek1Δ* diploids compared to *mek1Δ* alone [42] and *dmc1Δ hed1-3A* mutants take longer to repair DSBs than *dmc1Δ hed1*. These results indicate that Hed1 is able to interfere with Rad51-Rad54 complex formation in the absence of Mek1 and is consistent with *in vitro* experiments showing Hed1 purified from *E*. *coli* prevents Rad54 from binding to Rad51 [39]. There was an excellent correlation between the amount of Hed1 phosphorylation, Hed1 protein stability and the ability of Rad51 to mediate DSB repair and allow meiotic progression. Therefore, the most likely mechanism is that Mek1 phosphorylation of the N terminal region of Hed1 promotes protein stability. How Hed1 degradation is impeded by phosphorylation is an interesting question that warrants further study.

### Mek1 down regulates Rad51 by two distinct mechanisms

Degradation of Hed1 after inactivation of Mek1 eliminates one obstacle to Rad51-Rad54 complex formation thereby allowing DSB repair (Fig 7B). Another obstacle to Rad51-mediated recombination in *dmc1Δ* cells is the negative charge conferred by Mek1 phosphorylation of Rad54 T132, which reduces the affinity of Rad54 for Rad51 [41] (Fig 7A). The contribution of Rad54 T132 phosphorylation to Rad51 down regulation is relatively minor compared to Hed1, however. In the *dmc1Δ* background, the *RAD54-T132A* mutant increased sporulation from 1.1 to 22%. In contrast, *hed1Δ* allowed 89% of the *dmc1Δ* cells to sporulate, indicating more efficient DNA repair, similar to a *mek1Δ dmc1Δ* diploid [41]. Both mechanisms contribute to Rad51 down-regulation, however, as combining *hed1Δ* and *RAD54-T132A* results in more extreme phenotypes than the single mutants both in *dmc1Δ* or the presence of a hypomorphic *dmc1* mutant [41, 43]. Removing both *MEK1*-dependent impediments to Rad51-Rad54 complex formation in the *dmc1Δ* background resulted in ~12% viable spores, compared to < 2% for *dmc1Δ mek1Δ*, indicating that Mek1 phosphorylation of other substrates enables some IH recombination by Rad51 [41].

In *DMC1* diploids, *RAD54-T132A hed1Δ* exhibits only a two-fold decrease in IH bias, indicating that down-regulation of Rad51 through Mek1-dependent mechanisms is not as important in the presence of Dmc1 [28, 42, 43]. However, the observations that (1) Rad54 T132 and Hed1 T40 are phosphorylated in WT cells, (2) a decrease in IH bias (albeit small) is observed in *RAD54-T132A hed1Δ* diploids and (3) Hed1 co-localizes with Rad51 during WT meiosis suggest that *MEK1*-dependent regulation of Rad51 occurs during normal meiosis [40, 41, 43, 71]. In this way, Rad54 and Hed1 phosphorylation can contribute to the inhibition of Rad51 while IH recombination is occurring via Dmc1, but then can be coordinately removed by inactivating Mek1 to allow for repair any residual DSBs (Fig 7B).

### Dmc1 is a better recombinase than Rad51 for making stable IH connections

Most organisms that utilize meiotic recombination to make stable connections between homologs contain Dmc1 [23]. In contrast, nematodes and fruit flies form SCs independently of recombination and utilize only Rad51. This has led to the suggestion that Dmc1 itself, perhaps with its accessory factors, is better at making stable IH connections than Rad51 [23, 72]. Support for this idea comes from the comparison of recombination mediated by Dmc1 in WT diploids (where the presence of Rad51 is important for IH bias but its strand exchange activity is repressed) to that of Rad51 alone in *dmc1Δ hed1Δ* strains. [42] proposed that the decrease in IH bias exhibited by Rad51 results in a delay in pairing and synapsis. As a result of the failure in interhomolog engagement, Spo11 activity is not downregulated and DSBs continue to be generated disproportionately on large chromosomes [62]. Strand invasion of these breaks continues to occur until synapsis is achieved. However, because of a defect in CO assurance, there is a fraction of chromosomes that fail to get any crossovers at all.

Our sequencing analysis of *dmc1Δ hed1-3A* tetrads supports and extends this model. In contrast to the *dmc1Δ hed1Δ* diploid, which exhibited a decrease in both COs and NCOs compared to WT, the absolute number of COs and NCOs in the *dmc1Δ hed1-3A* hybrid was equivalent to WT. One explanation is that the need to degrade unphosphorylated Hed1-3A protein gives cells more time to establish IH connections than in *dmc1Δ hed1Δ*, resulting in more NCOs and COs. The Rad51-mediated IH events were distributed in an “all or none” manner, suggesting that making one stable IH connection increases the likelihood of additional IH events. Despite having WT levels of COs, non-exchange chromosomes were significantly increased in the *dmc1Δ hed1-3A* mutant, indicating that Rad51-mediated recombination is defective in CO assurance not only on chromosome III as observed by Lao et al (2013) but throughout the genome.

We propose that a major reason why Rad51 is worse at mediating stable IH interactions compared to Dmc1 is because Rad51 does not handle basepair mismatches well. This would not be surprising given that Rad51 normally invades sister chromatids, which have identical DNA sequences as the invading strand. This hypothesis is based on the observation that hybrid strains containing >60,000 SNPs exhibited a dramatic decrease in spore viability (~10%) when Rad51, rather than Dmc1, was the recombinase. This decrease was dependent upon the high number of mismatches, as spore viability in *dmc1Δ hed1Δ* and *dmc1Δ hed1-3A* mutants is ~70% in diploids when both parents were derived from the same background (although the possibility of genetic interactions has not been ruled out). Recent work has shown that Dmc1 is able to tolerate a low level of mismatches [73] and that Dmc1 is intrinsically able to stabilize mismatches, while the RecA and Rad51 recombinases cannot [74]. Lee et al. (2015) propose the inability of Rad51 to stabilize mismatches could contribute to IH bias by making it more difficult to generate stable IH connections. In contrast, the stabilization of mismatches in Dmc1-generated heteroduplexes could mask them from the mismatch repair machinery until after strand invasion is complete and Dmc1 is removed [74].

## Methods

### Strains

Complete genotypes are listed in S2 Table as well as the strain backgrounds from which the diploids were derived. Sporulation was carried out at 30°C as described in [50]. Genes were deleted by polymerase chain reaction (PCR)-based methods using the *kanMX6*, *natMX4*, *hphMX4*, markers that confer resistance to G418, nourseothricin and Hygromycin B, respectively. In addition, the *S*. *kluyveri HIS3* gene was used as a knockout marker [75–77]. Both the absence of the WT gene and the presence of the deletions were confirmed by PCR. To make diploids homozygous for different alleles of *HED1*, pNH302 and its derivatives were digested with BmgBI, integrated 400 bp upstream of the *hed1Δ* in each haploid parent, which were then mated to make diploids. The *NDT80-IN* diploid, ySZ207, used for the phosphoproteomic experiments, was created by deleting *ARG4* and *LYS2* from the A14154 and A14155 haploids and mating them to make the diploid [46].

DNA sequencing was performed using a hybrid diploid constructed by mating the SK1 strain, ORT7237 to the S288c strain, ORT7235, to create the diploid, AND1702 [65]. The second exon of *DMC1* was deleted with *kanMX6* in each haploid, creating a null allele of *DMC1* [44]. Mating of these haploids created the *dmc1Δ* diploid, NH2310. Subsequent to this, *HED1* was deleted with *hphMX4* and the double mutant haploids transformed with pNH302-3A and mated to make the *dmc1Δ hed1-3A* homozygous diploid, NH2294::pNH302-3A^2^.

### Plasmids

The *URA3 HED1* integrating plasmid, pNH302, was constructed using the polymerase chain reaction (PCR) to amplify a 1.1 kb fragment containing *HED1* flanked by NotI and XhoI restriction sites. After digestion, the NotI/XhoI fragment was ligated to pRS306 cut with NotI and XhoI to make pNH302. This plasmid can be targeted to integrate at *URA3* using StuI or 400 bp upstream of *HED1* using BmgBI. The *URA3-*integrating plasmids, pLP37 and pJR2, contain *mek1-K199R* and *mek1-as*, respectively [44, 78]. Mutations were introduced by site-directed mutagenesis using the QuikChange II Site-Directed Mutagenesis kit from Agilent Technologies. Sequencing of the entire *HED1* gene was performed for each allele at the Stony Brook University DNA Sequencing Facility to confirm that no unexpected mutations were present. The plasmids, pLT11 and pRS304 are *HOP1 URA3* and *ADE2* integrating vectors, respectively [20, 79].

### Sporulation and timecourses

*NDT80* diploids were sporulated as described in [50]. Liquid Spo medium is 2% potassium acetate (KOAc). Meiotic progression was analyzed by fixing cells in 37% formaldehyde, staining the nuclei with 4,6-diamidino-2-phenylindole (DAPI) and counting the number of bi-nucleate (Meiosis I) and tetranucleate (Meiosis II) cells using fluorescence microscopy. For the *NDT80-IN* phosphoproteomic experiments, a two ml YPD overnight culture of ySZ207 was diluted 1:2000 into 1.2 L YPA in a 2.8 L Fernbach flask and placed on a 30°C shaker until the optical density at wavelength 660 nm was 1.5. The cells were pelleted by centrifugation and resuspended in 700 ml Spo medium at a density of 3 X10^7^ cells/ml. After six hours, β-estradiol was added to a final concentration of 1 μM to induce transcription of *NDT80*. Aliquots of 50 ml of cells were collected at 6.0, 8.5, 9.0, 9.5, 10.0 and 10.5 hours in Spo medium, pelleted and resuspended in one ml water. The cells were transferred to a 1.5 ml microfuge tube, pelleted, the supernatant was removed and the pellet flash frozen in liquid nitrogen. Based on meiotic progression analysis, the 8.5 and 10.0 hr timepoints were chosen as representative of Meiosis I and Meiosis II, respectively.

### Phosphopeptide purification and mass spectrometry analysis

Frozen cell pellets were thawed and resuspended with an equal volume of lysis buffer [1 Mini EDTA-free protease inhibitor cocktail (Roche) per 5 ml, 5 mM EDTA, 5 mM NaF, 5 mM β-glycerophosphate in TBS (50 mM Tris-HCl, pH 8.0, 150 mM NaCl)]. Glass beads (Biospec) were added to a level just below the level of the liquid and the cells were lysed using a FastPrep-24 machine (MP Biomedicals) four times for 25 second intervals at setting 4.5 with one minute intervals on ice. Samples were examined by light microscopy to confirm >95% lysis.

To collect the lysates, the bottom of each tube was punctured with a needle and the tubes were placed within a larger tube containing a quantity of solid urea sufficient to give a final concentration of 8 M urea based on twice the volume of lysis buffer that was added to the pellet. The lysate was transferred into the tube with urea by a 10 sec spin in a microfuge. The lysates were incubated with the urea at 37°C for 30 min with rotation and then spun at 13,000 rpm for 10 min. The supernatants were transferred to 15 ml conical tubes. One ml of 8 M urea was added to the remaining pellet, the incubation and centrifugation repeated and the two supernatants pooled together.

The protein concentration of the denatured lysates was determined using the BioRad QuickStart Bradford protein assay. The proteins were reduced by addition of dithiolthreitol to a final concentration of 0.1 M for 30 min at 42°C, and alkylated using a final concentration of 0.3 M iodoacetamide in the dark for 30 min at room temperature. The reactions were terminated by incubating the samples in a final concentration of 14 μM 2-mercaptoethanol for 30 min at 42°C.

The samples were diluted five-fold with TBS to bring the urea concentration below 2 M. To digest the proteins into peptides, TPCK-treated trypsin (1 mg/ml TRTPCK, Worthington) was added to an amount equal to 1/100 the total protein and incubated with rotation for 15 hr at 37°C. The resulting peptides were acidified by addition of 10% trifluoroacetic acid to a final concentration of 0.2% and then spun in a microfuge at 13,000 rpm to remove insoluble material. The peptides were desalted using C18 columns and phosphopeptides enriched using immobilized metal ion chromatography as described in [47].

Phosphopeptides were fractionated by the MuDPIT method using an LTQ Orbitrap XL ion trap mass spectrometer (Thermo Fisher, San Jose, CA) equipped with a nano-liquid chromatography electrospray ionization source at the Stony Brook Proteomics Facility. The MS data were searched using SEQUEST as described in [47].

### Immunoblots and antibodies

Protein extracts for were prepared from five ml sporulating culture as described in [80]. Phostag gels (10% acrylamide/bis, 29:1) contain 37.5 μM Phostag (Wako Pure Chemical Industries, #AAL-107) and 75 μM MnCl_2_ (Sigma, #M3634) and were run as described in [81] with the following modifications: a Mini-Protean tetra Cell Electrophoresis Chamber (BioRad #165–8004) was used and samples were run at 100 V for 150–210 min. Proteins were transferred to polyvinylidine fluoride (PVDF) membranes using a Criteron Blotter with Plate Electrodes (BioRad #170–4070). For analysis of proteins using SDS-polyacrylamide gels without Phostag, extracts were made using the trichloroacetic acid method described by [82]. α-Hed1 [39] and α-Hop1 antibodies were used at 1:20,000 and 1:10,000 dilutions, respectively, and detected with a 1:10,000 dilution of goat anti-rabbit secondary antibodies coupled to horseradish peroxidase. Arp7 polyclonal goat antibodies (Santa Cruz, SC-8960) were used at a dilution of 1:10,000 as a loading control. The secondary antibody was a 1:10,000 dilution of donkey anti-goat IgG-HRP (Santa Cruz, SC-2020). α-GST antibodies were generously provided by D. Kellogg (University of California, Santa Cruz) and used as described in [20].

Antibodies specific to Hed1 phospho-T40 were generated by Covance. A rabbit was injected with the peptide, Ac-CKNKRSI(pT)TSPI-amide. After several months, the serum was tested for specificity to Hed1 p-T40. Such specificity was observed using a 1:20,000 dilution without further purification.

### *In vitro* kinase assays

GST-Mek1-as was partially purified from NH520/pLW6 and kinase assays were performed using the semi-synthetic epitope system [50]. Kinase reactions contained 6 pmoles of GST-Mek1-as and 5.5 pmol of GST-Hed1/GST-Hed1-3A or 1 pmol Rad54 as indicated. GST-Hed1 and Rad54 proteins were purified as previously described [39, 83] The inhibitor, 1-(1,1-Dimethylethyl)-3-(1-napthalenyl)-1H-pyrazolo[3,4-d]pyrimidin-4-amine (1-NA-PP1)(Tocris Bioscience) was used at a final concentration of 10 μM. After alkylation with *p*-nitrobenzyl mesylate (PNBM), phosphorylated proteins were detected by immunoblot analysis using the thiophosphate ester rabbit monoclonal antibody from Epitomics (Cat. # 2686–1). To examine whether Hed1 T40 was specifically phosphorylated, the kinase reactions were carried out as described above except that Fu-ATP was used in place of Fu-ATPγS and the PNBM step was omitted. The proteins were diluted 1:100 prior to fractionation by SDS-PAGE and the membranes were probed with the α-pT40 antibodies.

### Whole genome sequencing, reading mapping and coverage analysis

Genomic DNA was prepared from overnight single colony cultures as described in [84]. Libraries were constructed for paired-end sequencing (150 bp X 150 bp) and sequenced using a HiSeq 25 instrument (Illumina) following the manufacturer’s standard protocols at the Next Generation Sequencing platform of the Institut Curie. Sequencing data were aligned onto the Saccharomyces Genome Database (SGD) S288c reference genome (R64 from 2011-02-03 SGD website) using BWA (v0.6.2) [85], with options “aln–n 0.04 –I 22 –k 1 –t 12 –R 10”. PCR duplicates were filtered out from mapped sequencing reads using the MarkDuplicates tool from Picard [http://picard.sourceforge.net/]. Mapped sequencing read counts and coverage depth were calculated before and after PCR duplicate removal to estimate the level of PCR duplicates for each sample. The raw sequence data can be found at the National Center for Biotechnology Information Sequence Read Archive with the Accession number SRP068581.

### SNP genotyping and recombination analysis

The sequenced strains were systematically genotyped at 62,218 polymorphic positions as described [65]. Recombination events were detected with the CrossOver (v6.3) algorithm from ReCombine (v2.1) [66]. The genotype data were formatted according to the author description and the program was run with a 0 bp threshold (i.e, without grouping closely spaced events). The output of the CrossOver program was manually corrected (as some events were attributed to no chromatid). The output data were then processed using the GroupEvents program, kindly provided by J. Fung (UCSF) to merge closely spaced events into single classes [67]. Complex events were manually verified and reclassified when necessary. The genotype and output files from the Recombine and Group Events analyses can be accessed using the Dryad Digital Repository at http://dx.doi.org/10.5061/dryad.g6s2k.

## Supporting Information

S1 Data This file contains the data used to make the graphs in all of the figures. (XLSX)

S1 Table Sporulation and spore viability in various *dmc1Δ hed1* mutant diploids. (DOCX)

S2 Table *S*. *cerevisiae* strains. (DOCX)

S1 Fig Recombinant GST-Hed1-3A and Hed1-3A proteins are functional in *in vitro* assays. (A) Rad51 interaction was examined with purified GST-Hed1 or GST-Hed1-3A proteins. GST-Hed1 WT or 3A (0.6 μg) were incubated with Rad51 (0.6 μg) and affinity pull-down was performed with Glutathione Sepharose beads (GE Healthcare). The supernatant (S), wash (W), and SDS-eluate (E) fractions were analyzed by SDS-PAGE and Coomassie staining. (B) ATP hydrolysis was examined with 1 mM ATP, Rad54 (23 nM), and Rad51 (460 nM) in conjunction with Hed1 or Hed1-3A (330, 650, 980 nM) after a 10-min incubation at 30°C. The mean values and standard deviations of three experiments were plotted. (C) D-loop formation by Rad51 (1.3 μM)-Rad54 (210 nM) in the presence of Hed1 or Hed1-3A (120, 230, 350 nM) was examined with an 8-min incubation at 30°C. Schematic of the assay (i), a representative gel (ii), and the quantified result of three experiments (iii) are shown. Detailed procedures for the pull-down, ATPase, and D-loop assays are described in [39].
(TIF)

S2 Fig Meiotic progression in different *dmc1Δ hed1* hybrid strains. WT (AND1702::pRS306), *dmc1Δ* (NH2310::pRS306), *dmc1Δ hed1Δ* (NH2294::pRS306) and *dmc1Δ hed1-3A* (NH2294::pNH302-3A^2^) diploids were transferred to Spo medium and incubated at 30°C. At the indicated time points cells were fixed with formaldehyde and stained with DAPI to monitor meiotic progression by fluorescence microscopy. The average values from independent timecourses are plotted (n = 6 for WT and *dmc1Δ*, n = 7 for *dmc1Δ hed1Δ* and *dmc1Δ hed1-3A*). Error bars represent the standard deviation.
(TIF)

S3 Fig Distribution of E0 chromosomes in individual tetrads from *dmc1*Δ *hed1*Δ and *dmc1*Δ *hed1-3A* hybrid strains. (A) E0 chromosomes from *dmc1Δ hed1Δ* (NH2294::pRS306) tetrads. (B) E0 chromosomes from *dmc1Δ hed1-3A* (NH2294:: pNH302-3A^2^) tetrads.
(TIF)

S4 Fig Schematics of the chromosomes from WT, *dmc1Δ hed1Δ* and *dmc1Δ hed1-3A* tetrads determined by Next Generation Sequencing. Blue indicates sequences derived from the SK1 parent, while red indicates sequence from S288c. Gaps indicate regions where that was no SNP genotype information. Black circles indicate centromeres. Chromosomes (indicated by Roman numerals) are arranged by chromosome number (I to XVI) from top to bottom. The scale at the bottom indicates the number of kilobases.
(PDF)

S5 Fig Schematics of minority events from WT tetrads. E5: events between two chromatids; E6: events between three chromatids; E7, events between four chromatids. Blacks indicate the region of repair.
(PDF)

S6 Fig Schematics of minority events from *dmc1 Δ hed1Δ* tetrads. (PDF)

S7 Fig Schematics of minority events from *dmc1Δ hed1-3A* tetrads. (PDF)
