# Correction: Mek1 Down Regulates Rad51 Activity during Yeast Meiosis by Phosphorylation of Hed1

**DOI:** 10.1371/journal.pgen.1006283

**Published:** 2016-08-24

**Authors:** Tracy L. Callender, Raphaelle Laureau, Lihong Wan, Xiangyu Chen, Rima Sandhu, Saif Laljee, Sai Zhou, Ray T. Suhandynata, Evelyn Prugar, William A. Gaines, YoungHo Kwon, G. Valentin Börner, Alain Nicolas, Aaron M. Neiman, Nancy M. Hollingsworth

In the Data Availability statement, the link for accessing the processed recombination analysis files in Dryad is incorrect. The correct link is http://dx.doi.org/10.5061/dryad.g6s2k.
