# Supplementary material for: Mek1 Down Regulates Rad51 Activity during Yeast Meiosis by Phosphorylation of Hed1
Source: PLoS Genet. 2016 Aug 2;12(8):e1006226. doi: 10.1371/journal.pgen.1006226 (PMC4970670; doi:10.1371/journal.pgen.1006226)
Supplement: S1 Table — (DOCX) [file pgen.1006226.s002.docx]

**S1 Table. Sporulation and spore viability in various *dmc1∆ hed1* mutant diploids**

| Strain | Relevant genotype | % sporulation^a^ | % spore viability  (# tetrads)^b^ |
| --- | --- | --- | --- |
| NH716 | Wild type | 92.2 ± 0.4 | 86 ± 2.9 (75) |
| NH942::pNH302^2^ | *dmc1∆* | 0.0 ± 0.0 | ND^c^ |
| NH942/pRS316 | *dmc1∆ hed1∆* | 82.0 ±1.4 | 71.4 ± 8.9 (81) |
| NH942::pNH302-3A^2^ | *dmc1∆ hed1-3A* | 85.5 ± 3.5 | 71.4 ± 8.4 (100) |
| NH942::pNH302-T40A^2^ | *dmc1∆ hed1-T40A* | 81.0 ± 11.3 | 74.4 ± 5.1 (100) |
| NH942::pNH302-T40E^2^ | *dmc1∆ hed1-T40E* | 51.0 ± 12.0 | 70.9 ± 3.4 (98) |
| NH942::pNH302-S38A^2^ | *dmc1∆ hed1-S38A* | 34.5 ± 4.2 | 61.5 ± 2.6 (78) |
| NH942::pNH302-T41A^2^ | *dmc1∆ hed1-T41A* | 16.0 ± 5.7 | 58.6 ± 4.5 (78) |
| NH942::pNH302-S42A^2^ | *dmc1∆ hed1-S42A* | 44.7 ± 19.4 | 68.7 ± 6.7 (78) |

^a^Numbers are the averages and standard deviations from counting 200 cells from two independent colonies.

^b^Numbers are the averages and standard deviations from dissection of at least three independent colonies. The total number of tetrads dissected is indicated in parentheses.

^c^ND=No data
