# Supplementary material for: Mek1 Down Regulates Rad51 Activity during Yeast Meiosis by Phosphorylation of Hed1
Source: PLoS Genet. 2016 Aug 2;12(8):e1006226. doi: 10.1371/journal.pgen.1006226 (PMC4970670; doi:10.1371/journal.pgen.1006226)
Supplement: S2 Table — (DOCX) [file pgen.1006226.s003.docx]

**S2 Table. *S. cerevisiae* strains**

| Strain | Genotype | Source |
| --- | --- | --- |
| ySZ07^a^ | *MATα leu2::hisG ho::LYS2 lys2 trp1::hisG trp1::hisG::P_GAL_-NDT80::TRP1 lys4∆::Sphis5^+^*  *MAT***a** *leu2::hisG ho::LYS2 lys2 trp1::hisG trp1::hisG::P_GAL_-NDT80::TRP1 lys4∆::Sphis5^+^*  *ura3::P_GPD1_-GAL4(848).ER::URA3 arg4::kanMX6*  *ura3::P_GPD1_-GAL4(848).ER::URA3 arg4::kanMX6* | this work |
| NH716^a^ | *MATα leu2::hisG his4-X::LEU2(NgoMIV+ ori) ho∆::hisG ura3(∆pst-sma)*  *MAT***a** *leu2::hisG HIS4::LEU2(BamH + oriI) ho∆::hisG ura3(∆pst-sma)* | (Callender and Hollingsworth, 2010) |
| NH729^a^ | same as NH716 only *mek1∆::natMX4* | (Callender and Hollingsworth, 2010) |
| NH792^a^ | NH716 only *dmc1∆::kanMX6* | (Liu et al., 2014) |
| NH942^a^ | same as NH716 only *dmc1∆::kanX6 hed1∆::natMX4* | (Liu et al., 2014) |
| NH942::pNH302 ^a^ | same as NH942 only *HED1*  *HED1* | This work |
| NH942::pNH302-T40A^2 a^ | same as NH942 only *hed1-T40A*  *hed1-T40A* | This work |
| NH942::pNH302-T40E^2 a^ | same as NH942 only *hed1-T40E*  *hed1-T40E* | This work |
| NH1054^a^ | same as NH716 only *sae2/com1∆::kanMX6* | Chen et al 2015 |
| NH2166^a^ | same as NH716 only *dmc1∆::kanMX6 hed1∆::natMX4 ndt80∆::hphMX4* | this work |
| NH2166:: pNH302-3A^2 a^ | same as NH716 only *dmc1∆::kanMX6 hed1∆::natMX4::URA3-hed1-3A ndt80∆::hphMX4* | this work |
| NH2166:: pNH302-T40A^2 a^ | same as NH716 only *dmc1∆::kanMX6 hed1∆::natMX4::URA3-hed1-T40A ndt80∆::hphMX4* | this work |
| NH2166:: pNH302-T40E^2 a^ | same as NH716 only *dmc1∆::kanMX6 hed1∆::natMX4::URA3-hed1-T40E ndt80∆::hphMX4* | this work |
| NH2188^a^ | same as NH716 only *ndt80∆::natMX4* | (Liu et al., 2014) |
| NH2223^a^ | same as NH716 only *hed1∆::natMX4 ndt80∆::hphMX4* | (Liu et al., 2014) |
| NH144^a^ | *MATα leu2-K HIS4 lys2 ura3 ho∆::LYS2 arg4-Nsp*  *MAT***a** *leu2∆::hisG his4-x lys2 ura3 ho∆::LYS2 ARG4* | (Hollingsworth et al., 1995) |
| YTS1::pLP36^a^ | same as NH144 only *mek1∆::LEU2 ura3::mek1-K199R::URA3* | (de los Santos and Hollingsworth, 1999) |
| YTS3^a^ | same as NH144 only *red1::LEU2* | (de los Santos and Hollingsworth, 1999) |
| DW10::pRS306^2a^ | same as NH144 only *hop1::LEU2 ura3::URA3* | (Niu et al., 2005) |
| NH2303^a^ | same as NH144 only *spo11∆::natMX4* | this work |
| NH746^a^ | same as NH144 only *rec8∆::kanMX6* | (Callender and Hollingsworth, 2010) |
| NH2212::pJR2^a^ | *MATα leu2-K arg4-Nsp ura3 lys2 ho∆::LYS2 HIS3 TRP1*  *MATa leu2::hisG ARG4 ura3::pGPD-GAL4(848).ER::URA3 lys2 ho∆::LYS2 his3::hisG trp1::hisG*  *ndt80∆::kanMX6 mek1∆::natMX4::URA3-mek1-as*  *ndt80∆::TRP1-PGAL-NDT80 mek1∆::natMX4* | this work |
| NH2315/pRS316^b^ | *MATα leu2∆0 lys2∆0 ura3∆0 arg4-bg his3∆200 met15∆0 ho trp1∆63/CEN ARS URA3*  *MAT***a** *leu2∆0 LYS2 ura3∆0 ARG4 his3∆1 met15∆0 ho TRP1* | this work |
| NH2320/pRS316^b^ | same as NH2315/pRS316 only  *dmc1∆::kanMX6 hed1∆::hphMX4* | this work |
| NH2320::  pNH302-3A^2b^ | same as NH2315 only *dmc1∆::kanMX6 hed1∆::hphMX4::URA3::hed1-3A* | this work |
| AND1702^c^ | *MATα leu2∆0 HIS4 ho lys2∆0 ura3∆0 arg4-bgl met15∆0 trp1∆63*  *MAT***a** *leu2::hisG his4B::LEU2 ho lys2 ura3(Pst1-Sma1)::hisG arg4-RV MET15 trp1::hisG*  *his3∆200*  *HIS3* | (Laureau et al., 2016) |
| NH2310^c^ | same as AND1702 only *dmc1∆::kanMX6 hed1∆::hphMX4* | this work |
| NH2294^c^ | same as AND1702 only *dmc1∆::kanMX6 hed1∆::hphMX4* | this work |
| NH2294::  pNH302-3A^2c^ | same as AND1702 only *dmc1∆kanMX6 hed1∆::hphMX4::URA3-hed1-3A* | this work |
| NH1053^d^ | *MATα ho::hisG lys2 cyh^R^ LYS5 URA3*  *MAT***a** *ho::hisG LYS2 CYH lys5 ura3∆::natMX4* | (Liu et al., 2014) |
| NH2032^d^ | same as NH1053 only *dmc1∆::kanMX6 hed1∆::hphMX4* | this work |

^a^Both parents are derived from the SK1 background.

^b^Both parents are derived from the S288c background.

^c^Hybrid strains in which the haploid parents are derived from the SK1 and S288c backgrounds, respectively.

^d^Hybrid strains in which the haploid parents are derived from the YJM789 and S288c backgrounds, respectively.

Callender, T.L., and Hollingsworth, N.M. (2010). Mek1 suppression of meiotic double-strand break repair is specific to sister chromatids, chromosome autonomous and independent of Rec8 cohesin complexes. Genetics *185*, 771-782.

de los Santos, T., and Hollingsworth, N.M. (1999). Red1p: A *MEK1*-dependent phosphoprotein that physically interacts with Hop1p during meiosis in yeast. J Biol Chem *274*, 1783-1790.

Hollingsworth, N.M., Ponte, L., and Halsey, C. (1995). *MSH5*, a novel MutS homolog, facilitates meiotic reciprocal recombination between homologs in *Saccharomyces cerevisiae* but not mismatch repair. Genes Dev *9*, 1728-1739.

Laureau, R., Loeillet, S., Salinas, F., Bergstrom, A., Legoix-Ne, P., Liti, G., and Nicolas, A. (2016). Extensive Recombination of a Yeast Diploid Hybrid through Meiotic Reversion. PLoS Genet *12*, e1005781.

Liu, Y., Gaines, W.A., Callender, T., Busygina, V., Oke, A., Sung, P., Fung, J.C., and Hollingsworth, N.M. (2014). Down-regulation of Rad51 activity during meiosis in yeast prevents competition with Dmc1 for repair of double-strand breaks. PLoS Genet *10*, e1004005.

Niu, H., Wan, L., Baumgartner, B., Schaefer, D., Loidl, J., and Hollingsworth, N.M. (2005). Partner choice during meiosis is regulated by Hop1-promoted dimerization of Mek1. Mol Biol Cell *16*, 5804-5818.
