# Supplementary figures and images for: Mek1 Down Regulates Rad51 Activity during Yeast Meiosis by Phosphorylation of Hed1
Source: PLoS Genet. 2016 Aug 2;12(8):e1006226. doi: 10.1371/journal.pgen.1006226 (PMC4970670; doi:10.1371/journal.pgen.1006226)

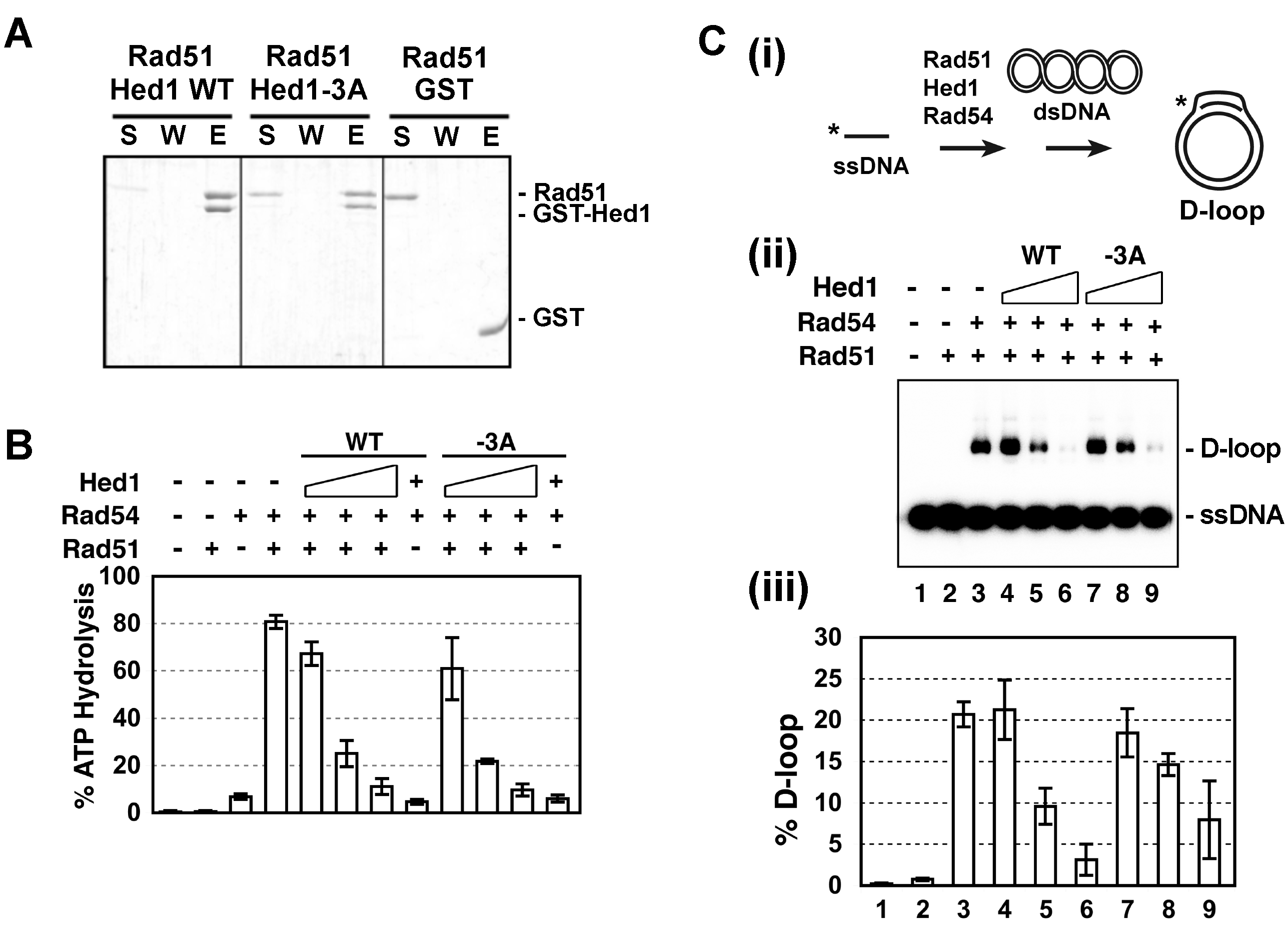

Supplement: S1 Fig — (A) Rad51 interaction was examined with purified GST-Hed1 or GST-Hed1-3A proteins. GST-Hed1 WT or 3A (0.6 μg) were incubated with Rad51 (0.6 μg) and affinity pull-down was performed with Glutathione Sepharose beads (GE Healthcare). The supernatant (S), wash (W), and SDS-eluate (E) fractions were analyzed by SDS-PAGE and Coomassie staining. (B) ATP hydrolysis was examined with 1 mM ATP, Rad54 (23 nM), and Rad51 (460 nM) in conjunction with Hed1 or Hed1-3A (330, 650, 980 nM) after a 10-min incubation at 30°C. The mean values and standard deviations of three experiments were plotted. (C) D-loop formation by Rad51 (1.3 μM)-Rad54 (210 nM) in the presence of Hed1 or Hed1-3A (120, 230, 350 nM) was examined with an 8-min incubation at 30°C. Schematic of the assay (i), a representative gel (ii), and the quantified result of three experiments (iii) are shown. Detailed procedures for the pull-down, ATPase, and D-loop assays are described in [39]. (TIF) [file pgen.1006226.s004.tif]

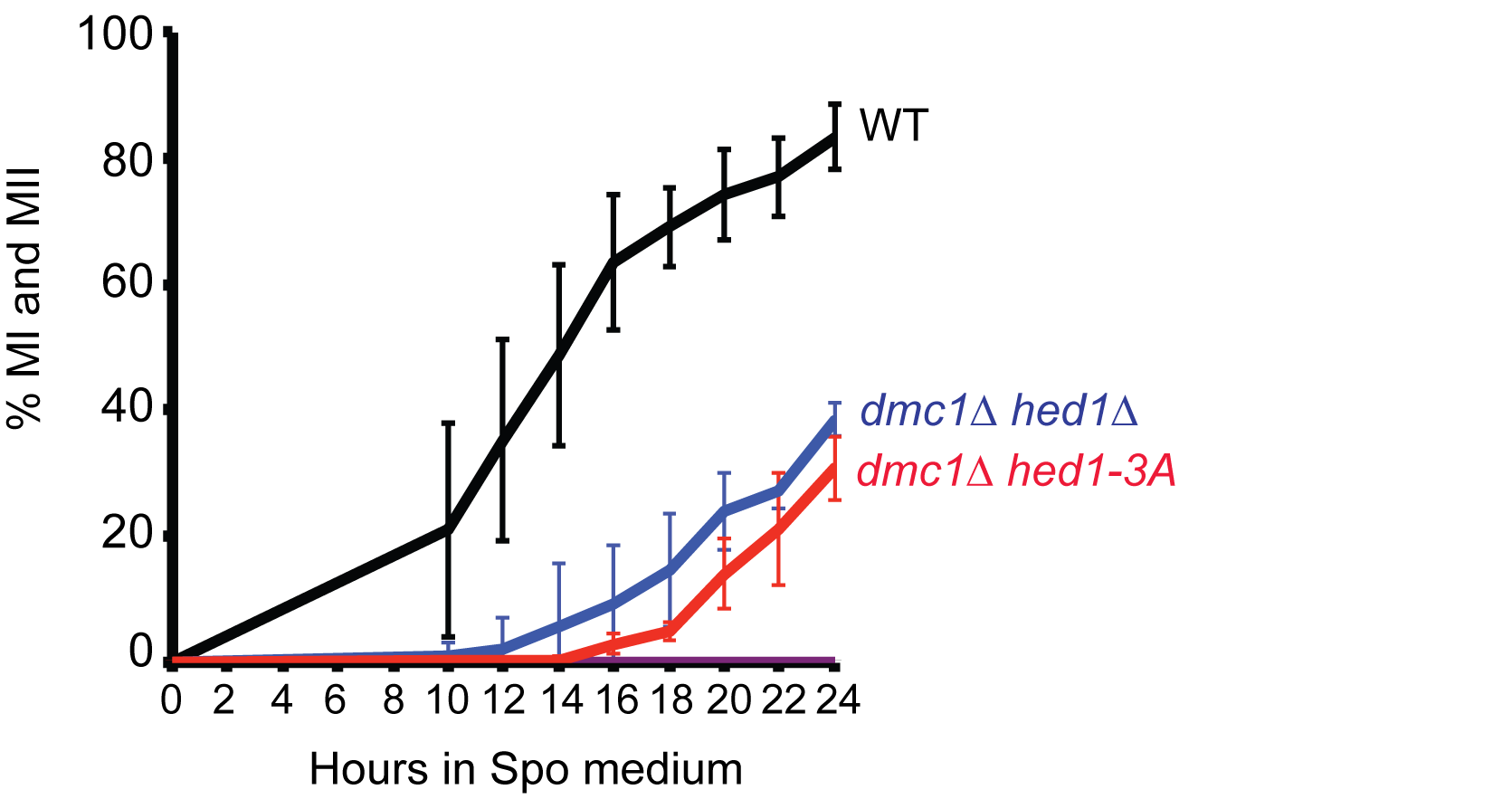

Supplement: S2 Fig — WT (AND1702::pRS306), dmc1Δ (NH2310::pRS306), dmc1Δ hed1Δ (NH2294::pRS306) and dmc1Δ hed1-3A (NH2294::pNH302-3A2) diploids were transferred to Spo medium and incubated at 30°C. At the indicated time points cells were fixed with formaldehyde and stained with DAPI to monitor meiotic progression by fluorescence microscopy. The average values from independent timecourses are plotted (n = 6 for WT and dmc1Δ, n = 7 for dmc1Δ hed1Δ and dmc1Δ hed1-3A). Error bars represent the standard deviation. (TIF) [file pgen.1006226.s005.tif]

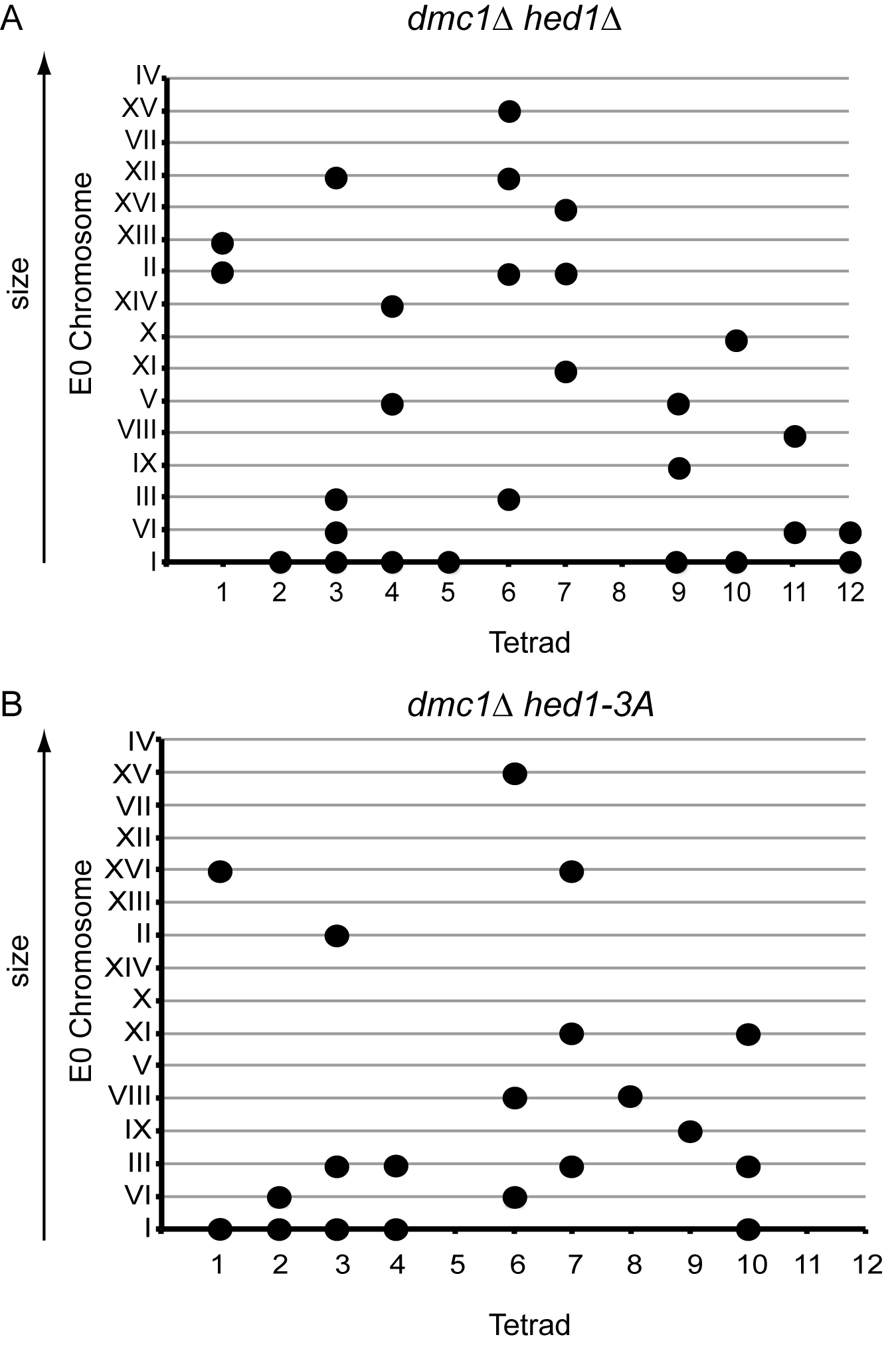

Supplement: S3 Fig — (A) E0 chromosomes from dmc1Δ hed1Δ (NH2294::pRS306) tetrads. (B) E0 chromosomes from dmc1Δ hed1-3A (NH2294:: pNH302-3A2) tetrads. (TIF) [file pgen.1006226.s006.tif]
