# Supplementary material for: Mek1 Down Regulates Rad51 Activity during Yeast Meiosis by Phosphorylation of Hed1
Source: PLoS Genet. 2016 Aug 2;12(8):e1006226. doi: 10.1371/journal.pgen.1006226 (PMC4970670; doi:10.1371/journal.pgen.1006226)

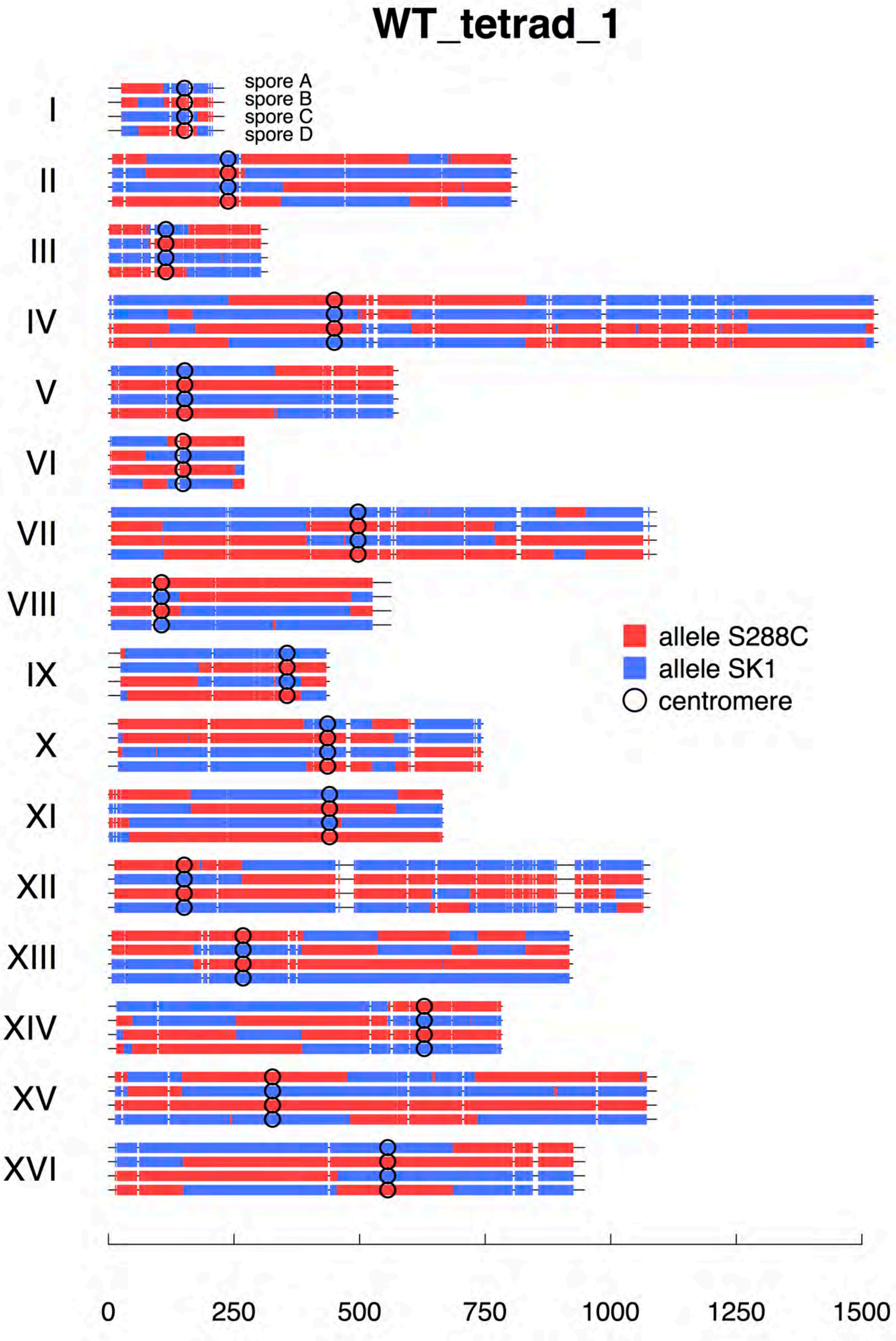
WT_tetrad_1
spore A
spore B
spore C
spore D
I
II
III
IV
V
VI
VII
VIII
IX
X
XI
XII
XIII
XIV
XV
XVI
allele S288C
allele SK1
centromere
0
250
500
750
1000
1250
1500

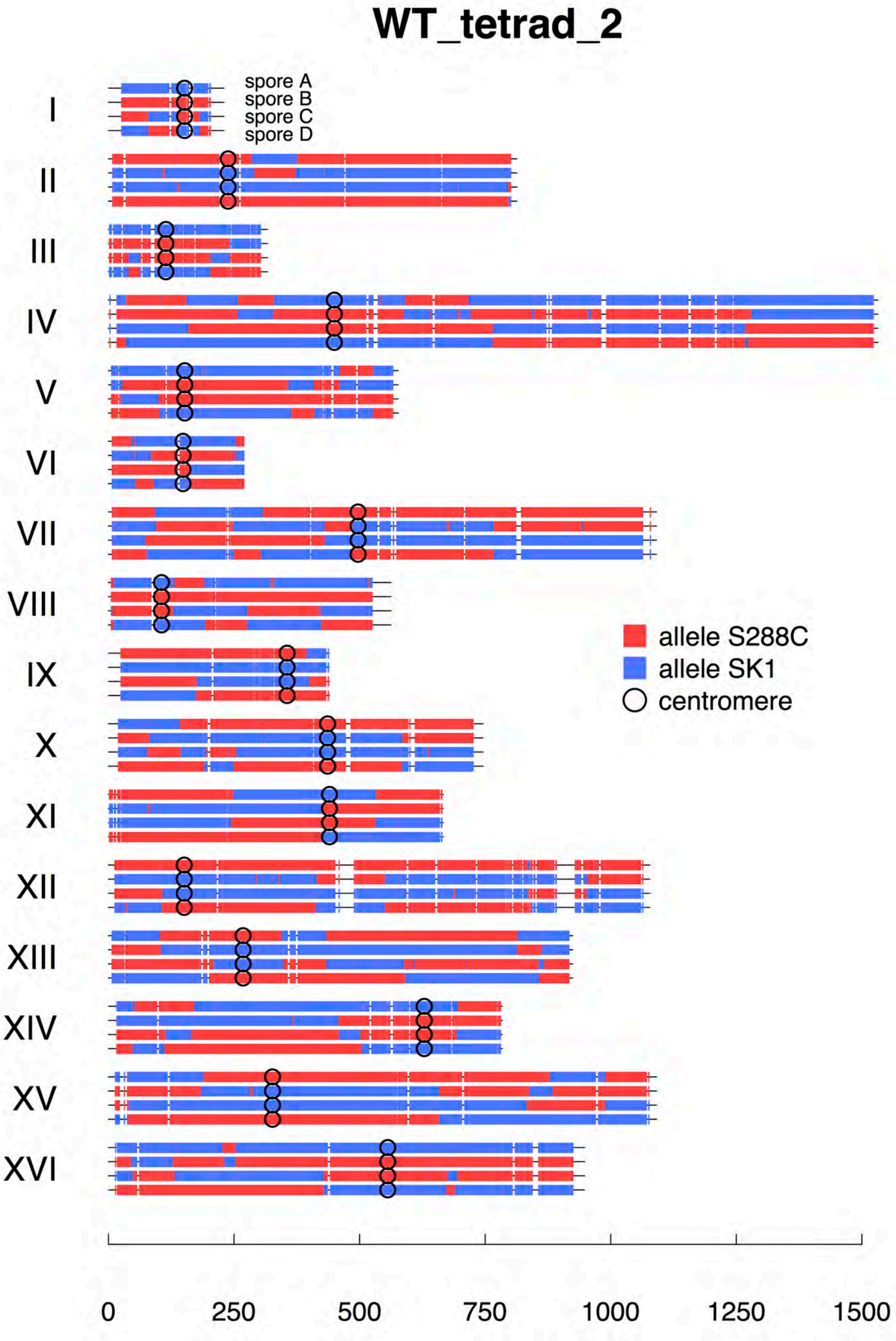
WT_tetrad_2
spore A
spore B
spore C
spore D
I
II
III
IV
V
VI
VII
VIII
IX
X
XI
XII
XIII
XIV
XV
XVI
allele S288C
allele SK1
centromere
0
250
500
750
1000
1250
1500

WT_tetrad_3

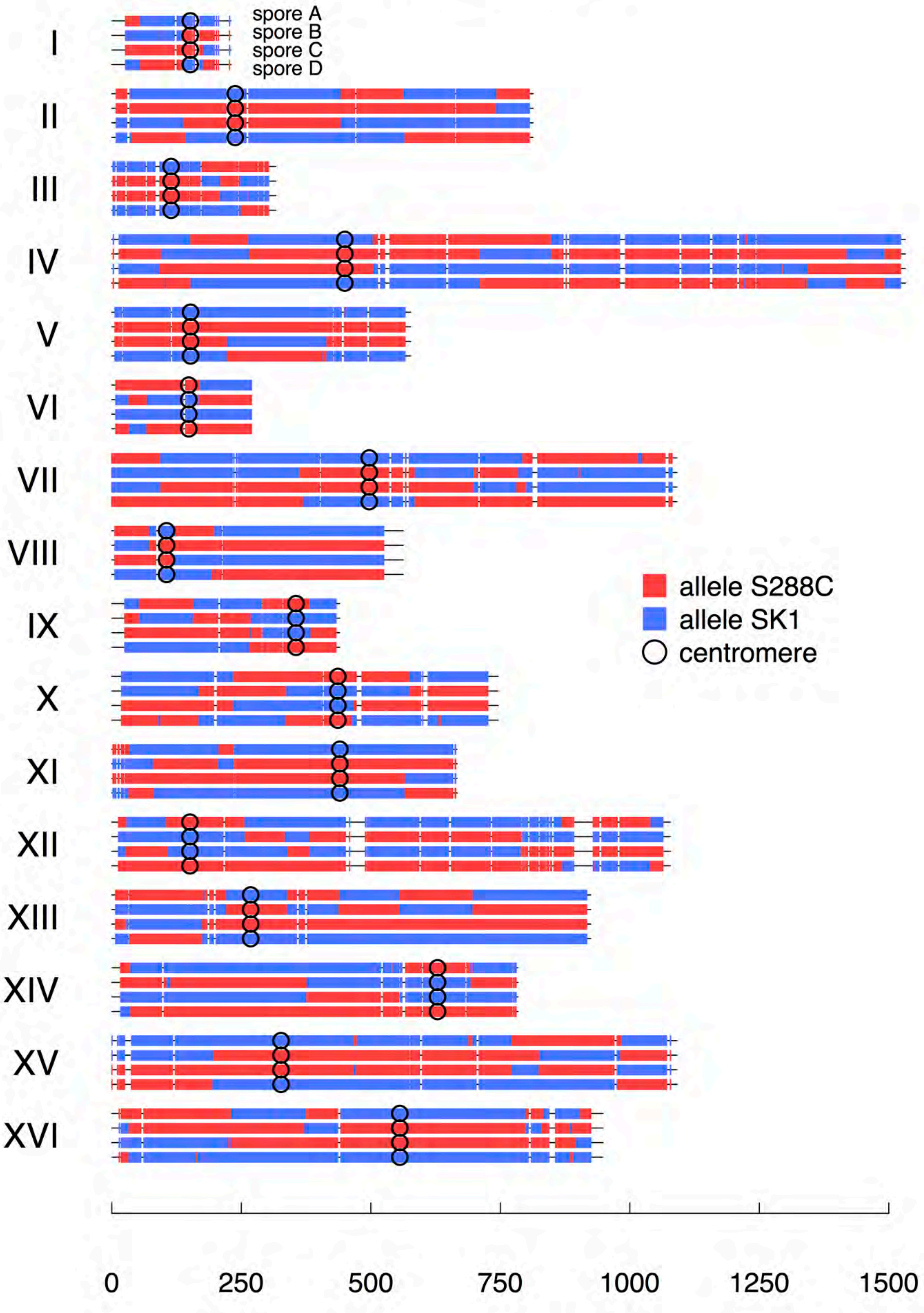

I
II
III
IV
V
VI
VII
VIII
IX
X
XI
XII
XIII
XIV
XV
XVI
spore A
spore B
spore C
spore D
allele S288C
allele SK1
centromere
0
250
500
750
1000
1250
1500

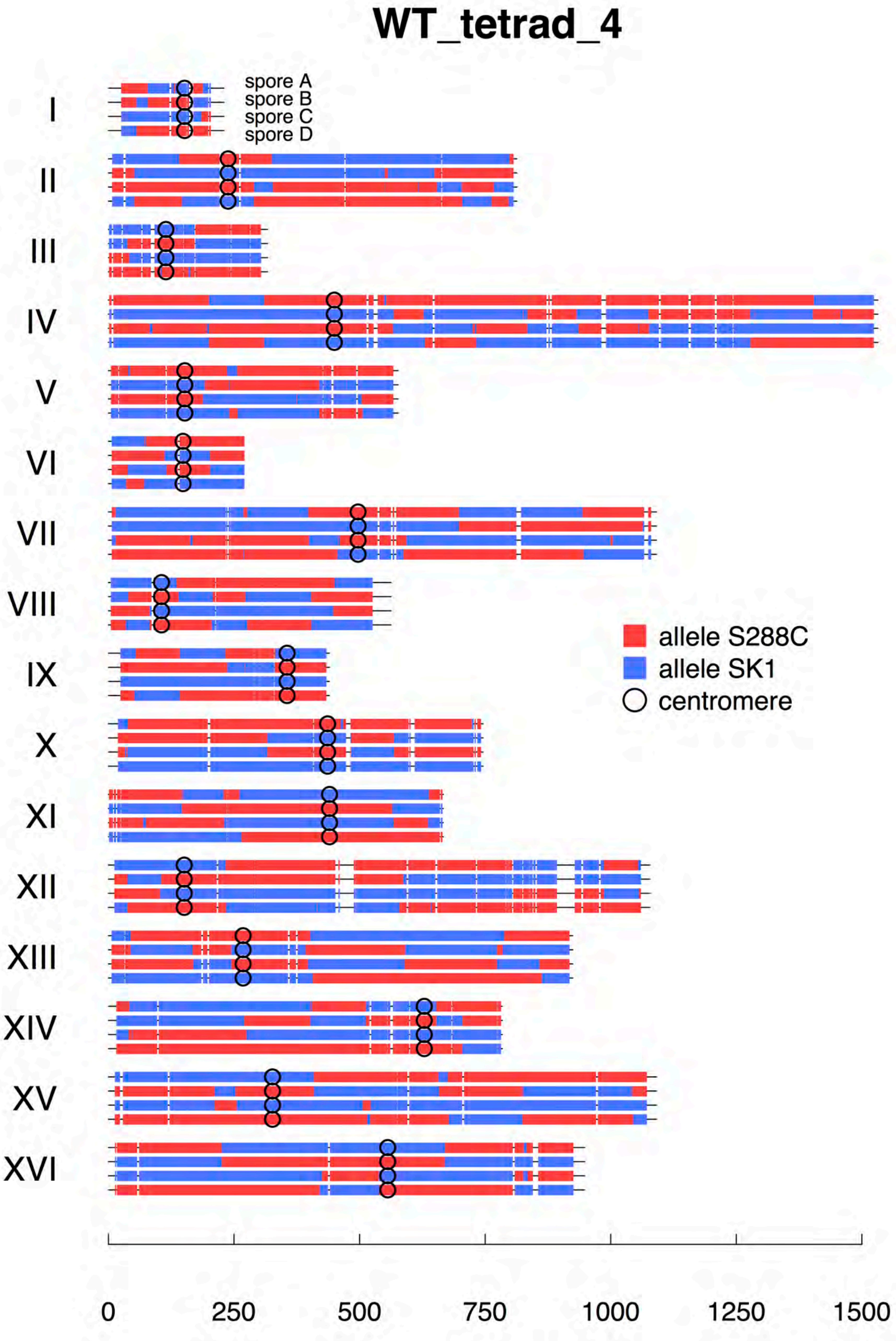
WT_tetrad_4
I
II
III
IV
V
VI
VII
VIII
IX
X
XI
XII
XIII
XIV
XV
XVI
spore A
spore B
spore C
spore D
allele S288C
allele SK1
centromere
0
250
500
750
1000
1250
1500

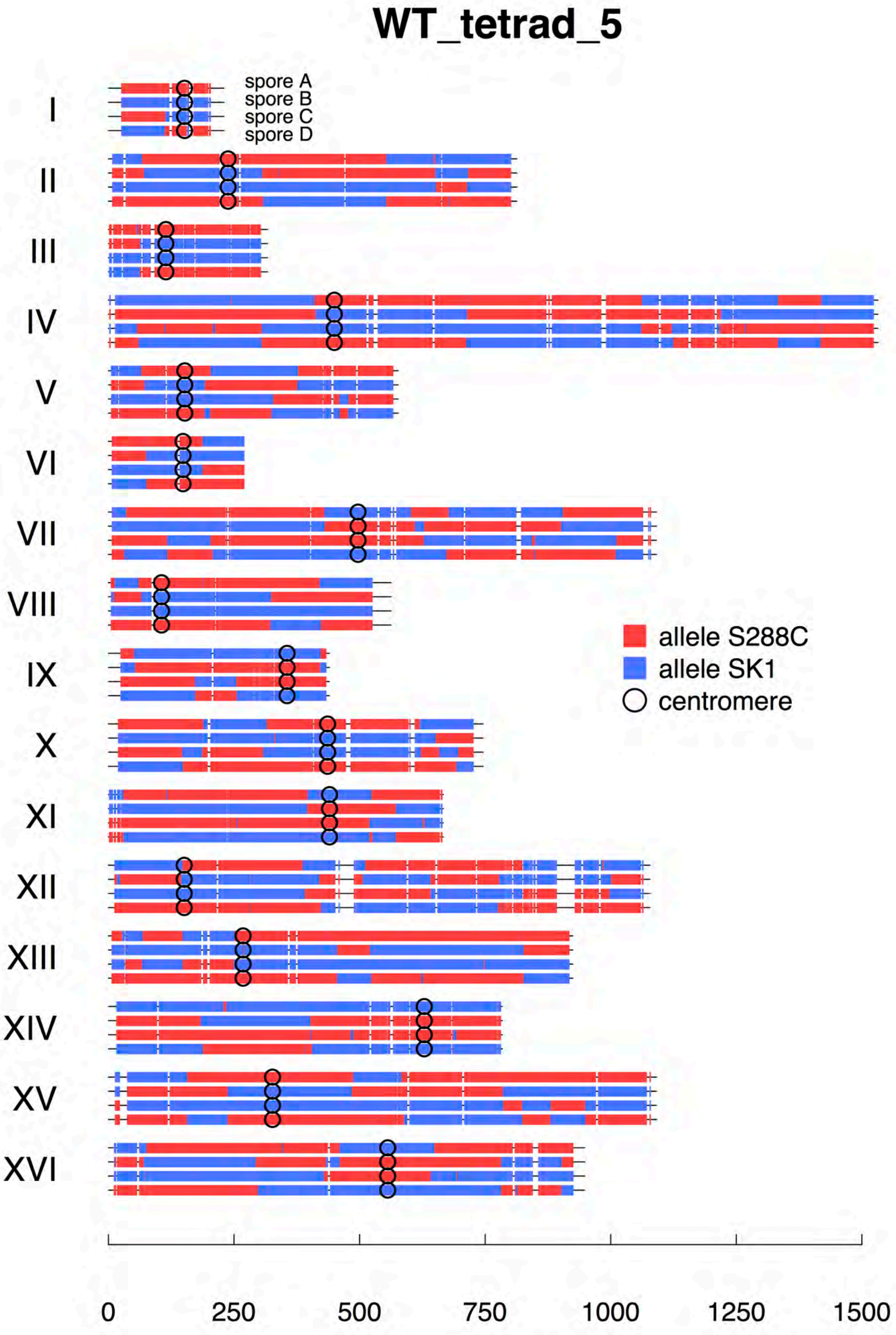
WT_tetrad_5
I
II
III
IV
V
VI
VII
VIII
IX
X
XI
XII
XIII
XIV
XV
XVI
spore A
spore B
spore C
spore D
allele S288C
allele SK1
centromere
0
250
500
750
1000
1250
1500

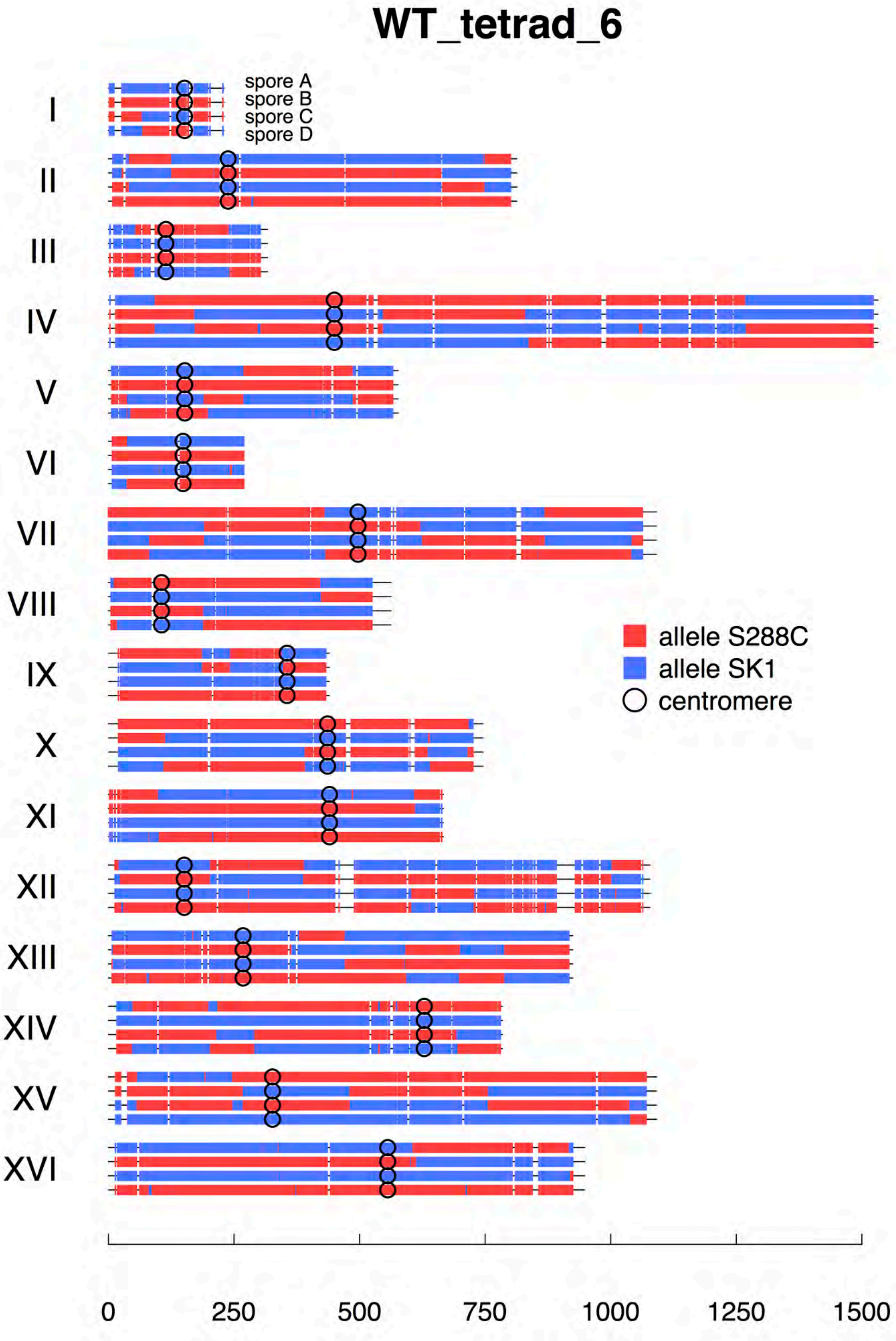
WT_tetrad_6
spore A
spore B
spore C
spore D
I
II
III
IV
V
VI
VII
VIII
IX
X
XI
XII
XIII
XIV
XV
XVI
allele S288C
allele SK1
centromere
0
250
500
750
1000
1250
1500

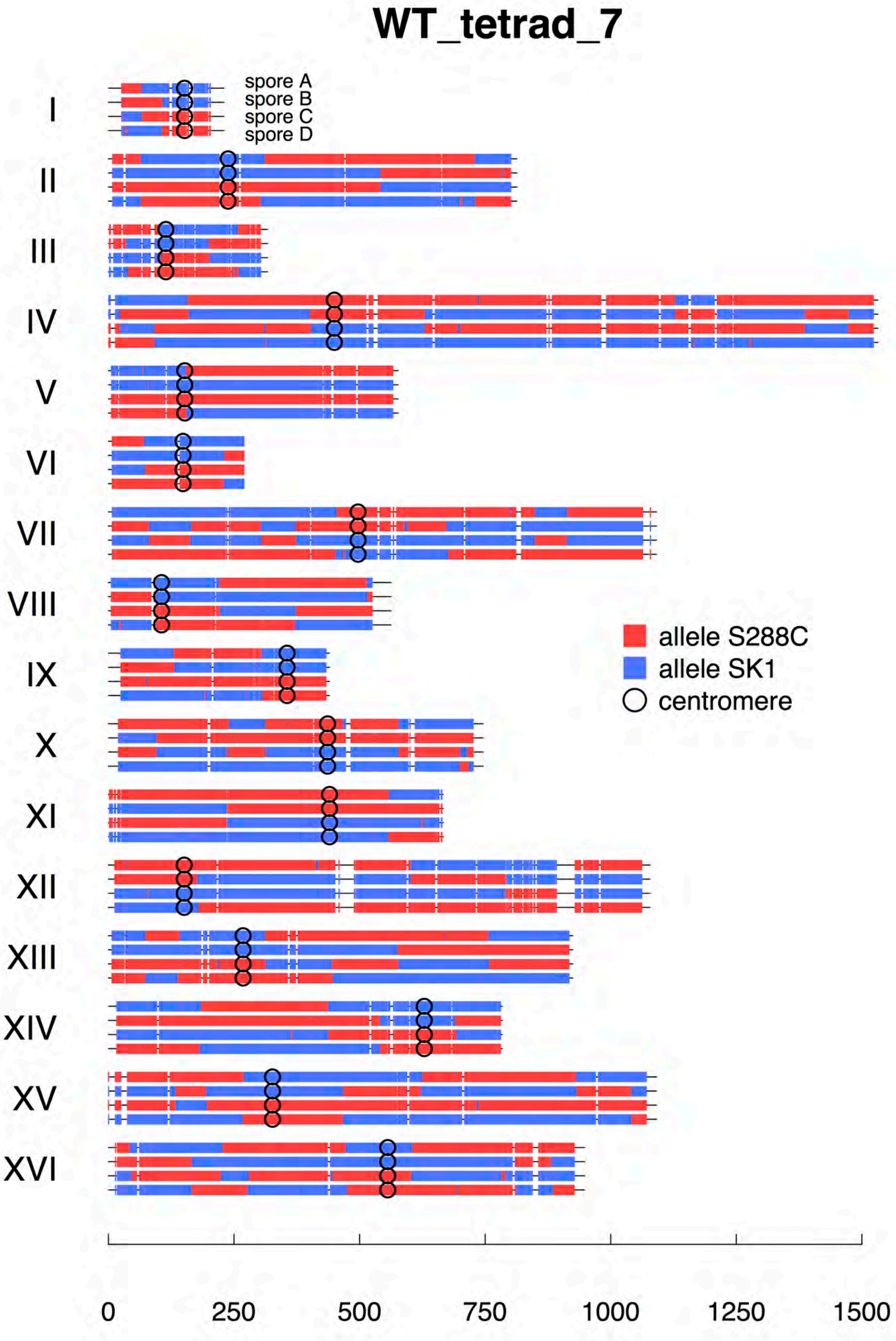
WT_tetrad_7
spore A
spore B
spore C
spore D
I
II
III
IV
V
VI
VII
VIII
IX
X
XI
XII
XIII
XIV
XV
XVI
allele S288C
allele SK1
centromere
0
250
500
750
1000
1250
1500

WT_tetrad_8

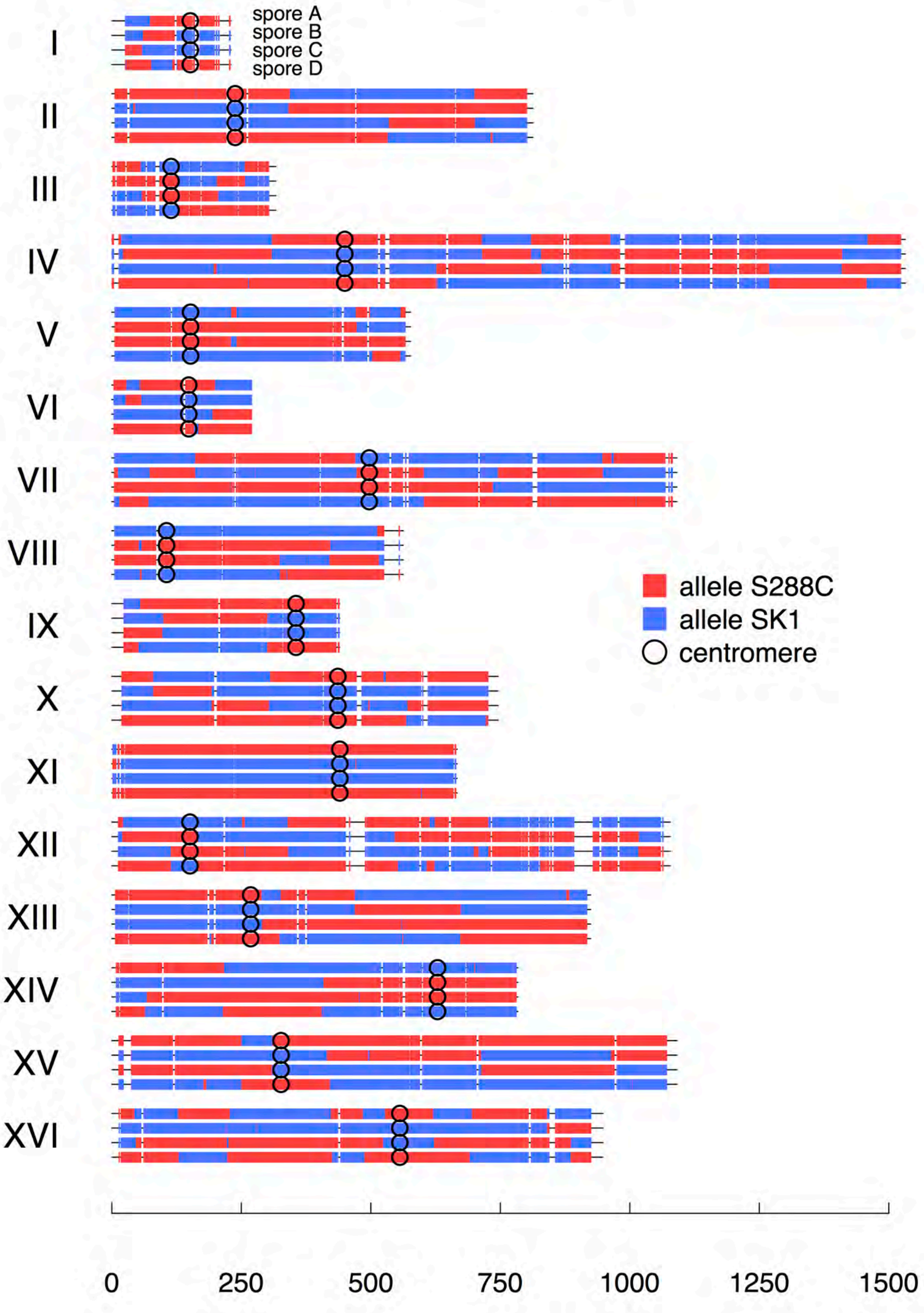

spore A
spore B
spore C
spore D
I
II
III
IV
V
VI
VII
VIII
IX
X
XI
XII
XIII
XIV
XV
XVI
allele S288C
allele SK1
centromere
0
250
500
750
1000
1250
1500

WT_tetrad_9

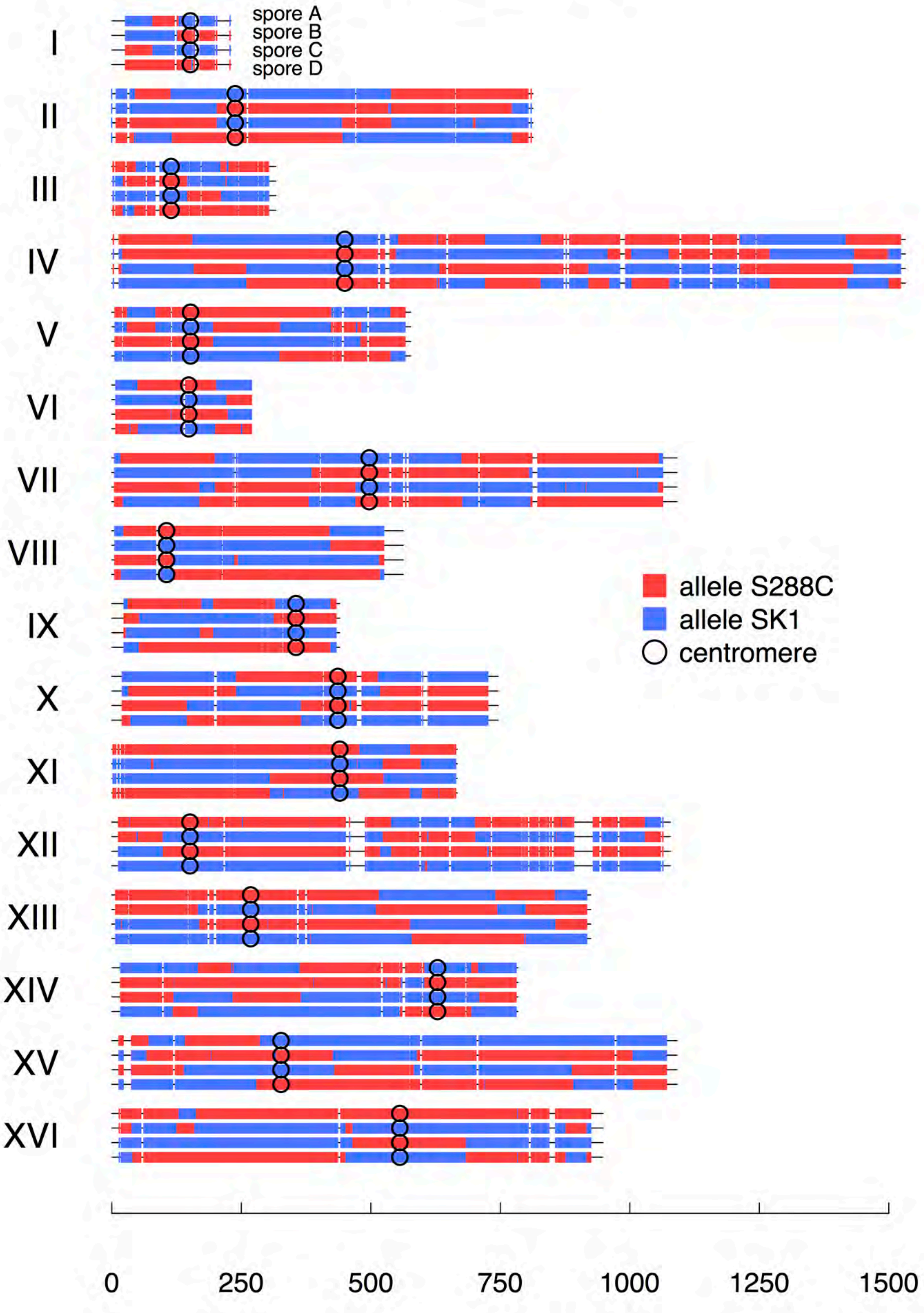

spore A
spore B
spore C
spore D
I
II
III
IV
V
VI
VII
VIII
IX
X
XI
XII
XIII
XIV
XV
XVI
allele S288C
allele SK1
centromere
0
250
500
750
1000
1250
1500

WT_tetrad_10

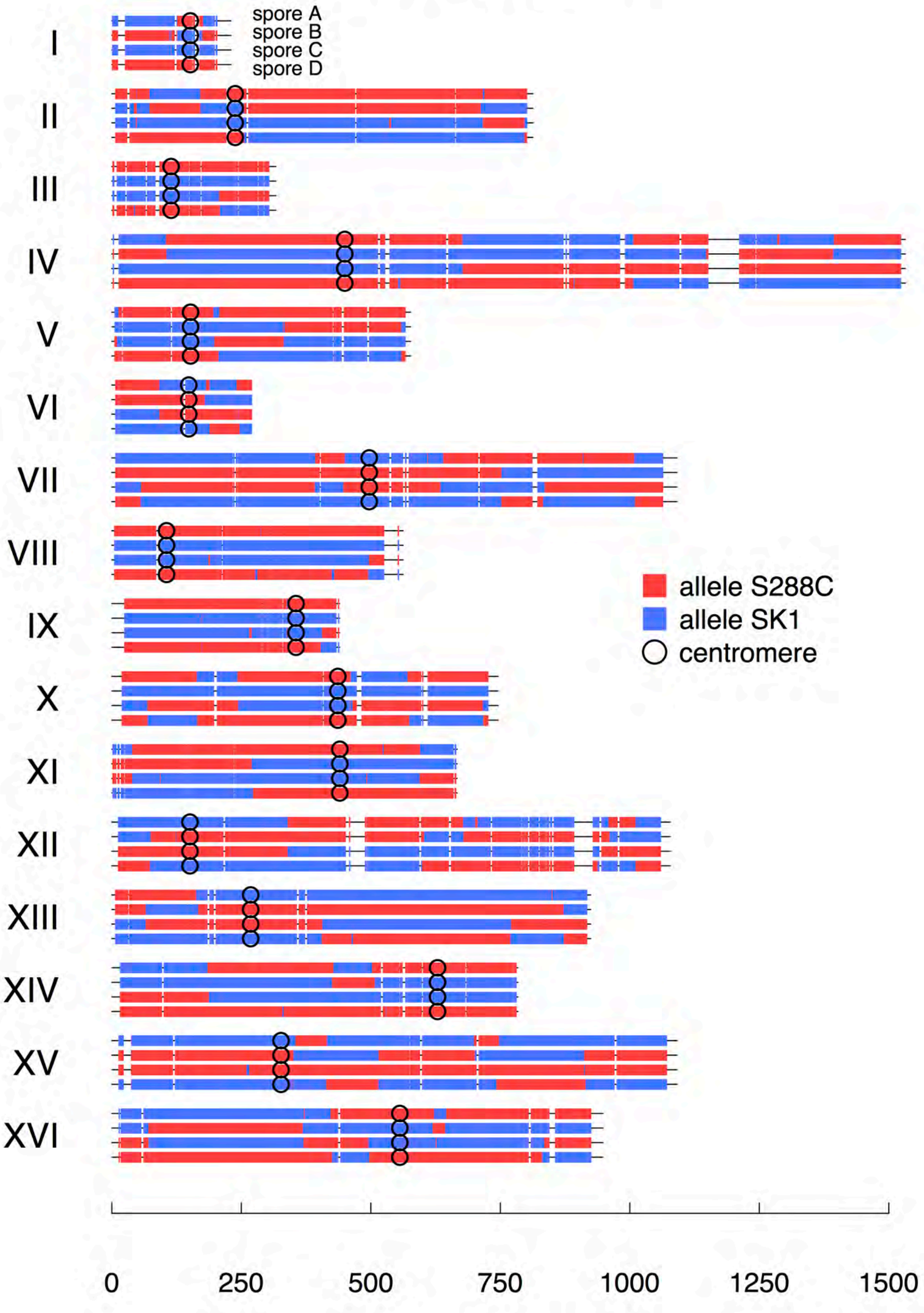

spore A
spore B
spore C
spore D
I
II
III
IV
V
VI
VII
VIII
IX
X
XI
XII
XIII
XIV
XV
XVI
allele S288C
allele SK1
centromere
0
250
500
750
1000
1250
1500

WT_tetrad_11

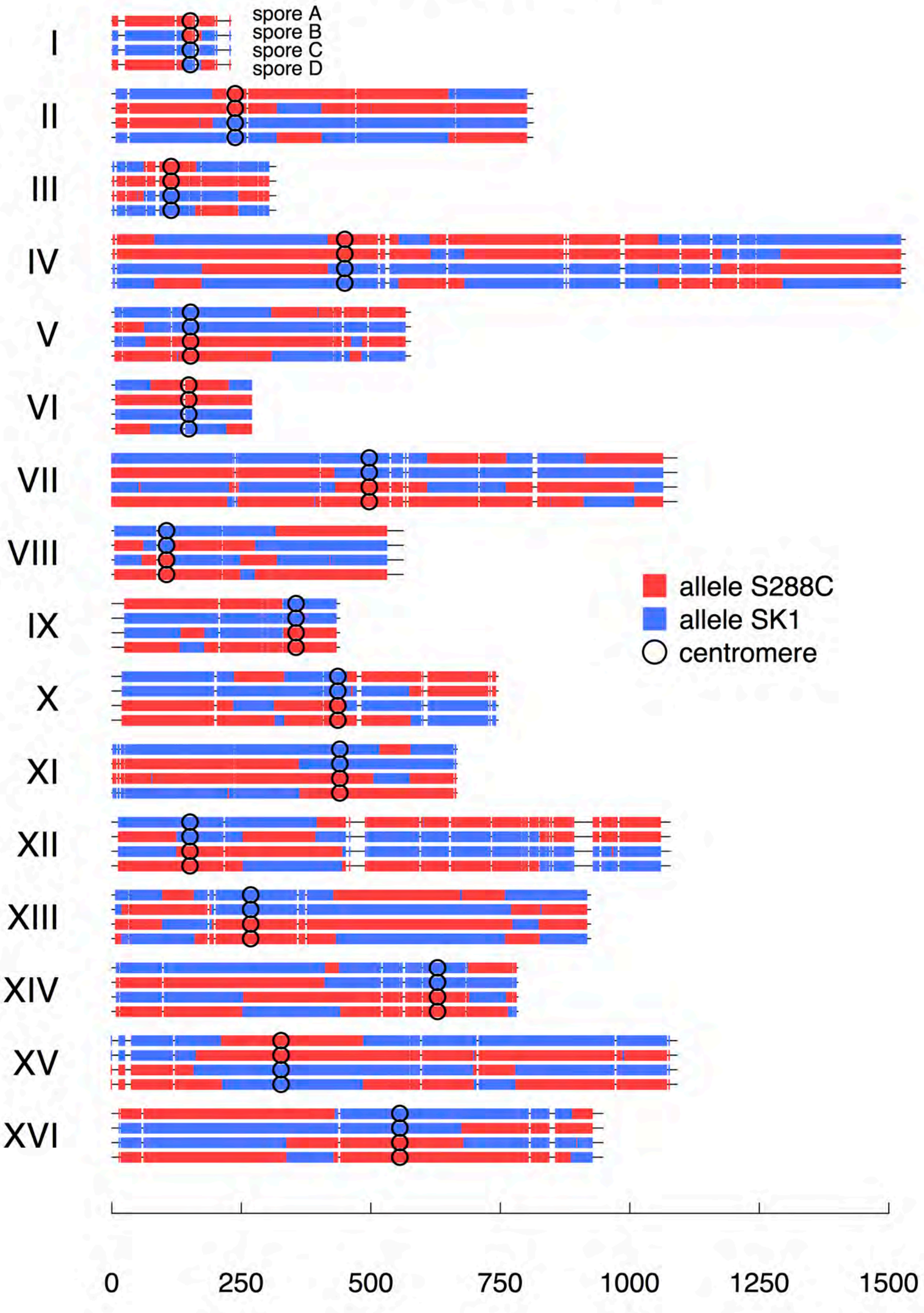
spore A
spore B
spore C
spore D
I
II
III
IV
V
VI
VII
VIII
IX
X
XI
XII
XIII
XIV
XV
XVI
allele S288C
allele SK1
centromere
0
250
500
750
1000
1250
1500

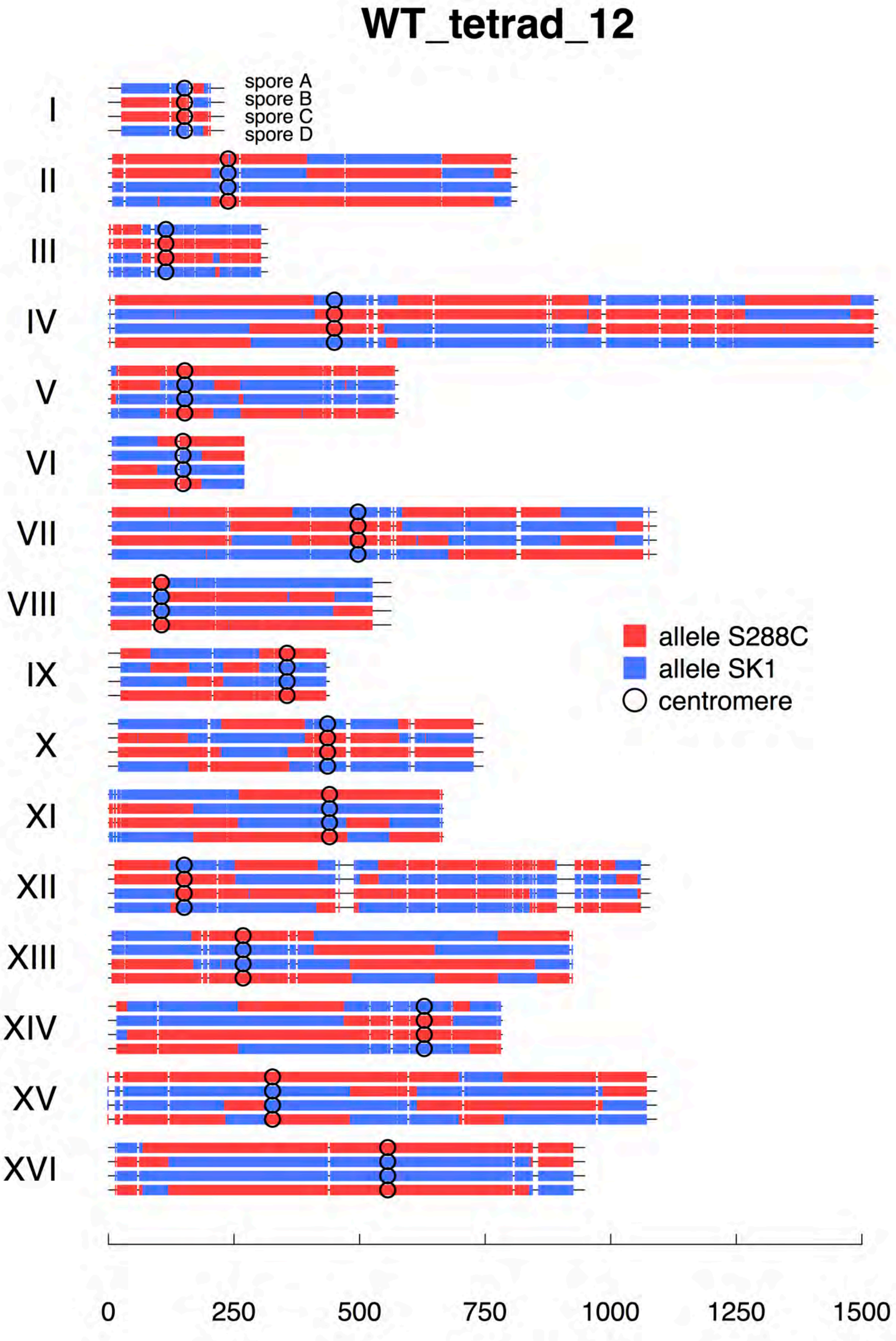
WT_tetrad_12
I
II
III
IV
V
VI
VII
VIII
IX
X
XI
XII
XIII
XIV
XV
XVI
spore A
spore B
spore C
spore D
allele S288C
allele SK1
centromere
0
250
500
750
1000
1250
1500

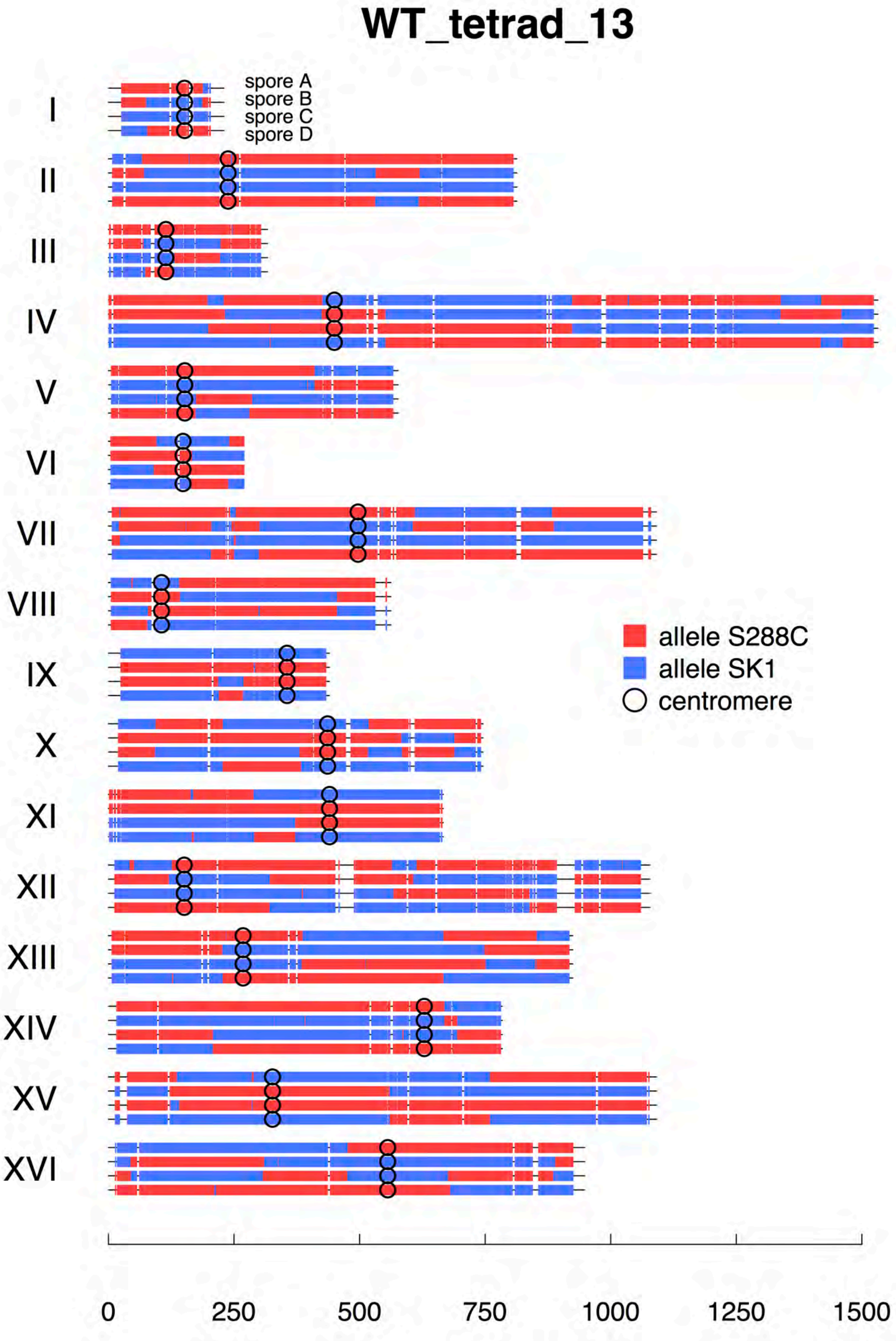

WT_tetrad_13
I
II
III
IV
V
VI
VII
VIII
IX
X
XI
XII
XIII
XIV
XV
XVI
spore A
spore B
spore C
spore D
allele S288C
allele SK1
centromere
0
250
500
750
1000
1250
1500

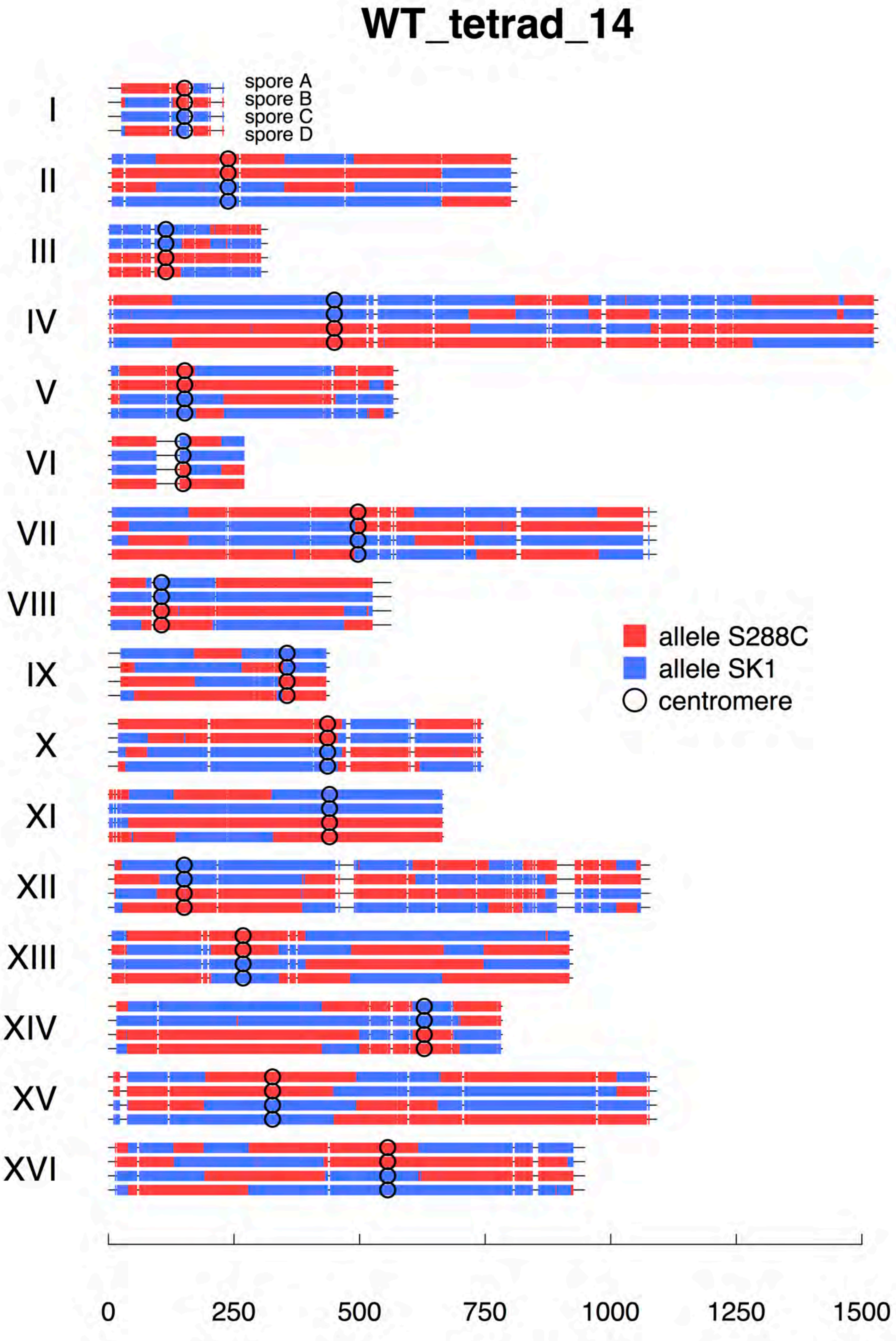
WT_tetrad_14
I
II
III
IV
V
VI
VII
VIII
IX
X
XI
XII
XIII
XIV
XV
XVI
spore A
spore B
spore C
spore D
allele S288C
allele SK1
centromere
0
250
500
750
1000
1250
1500

WT_tetrad_15

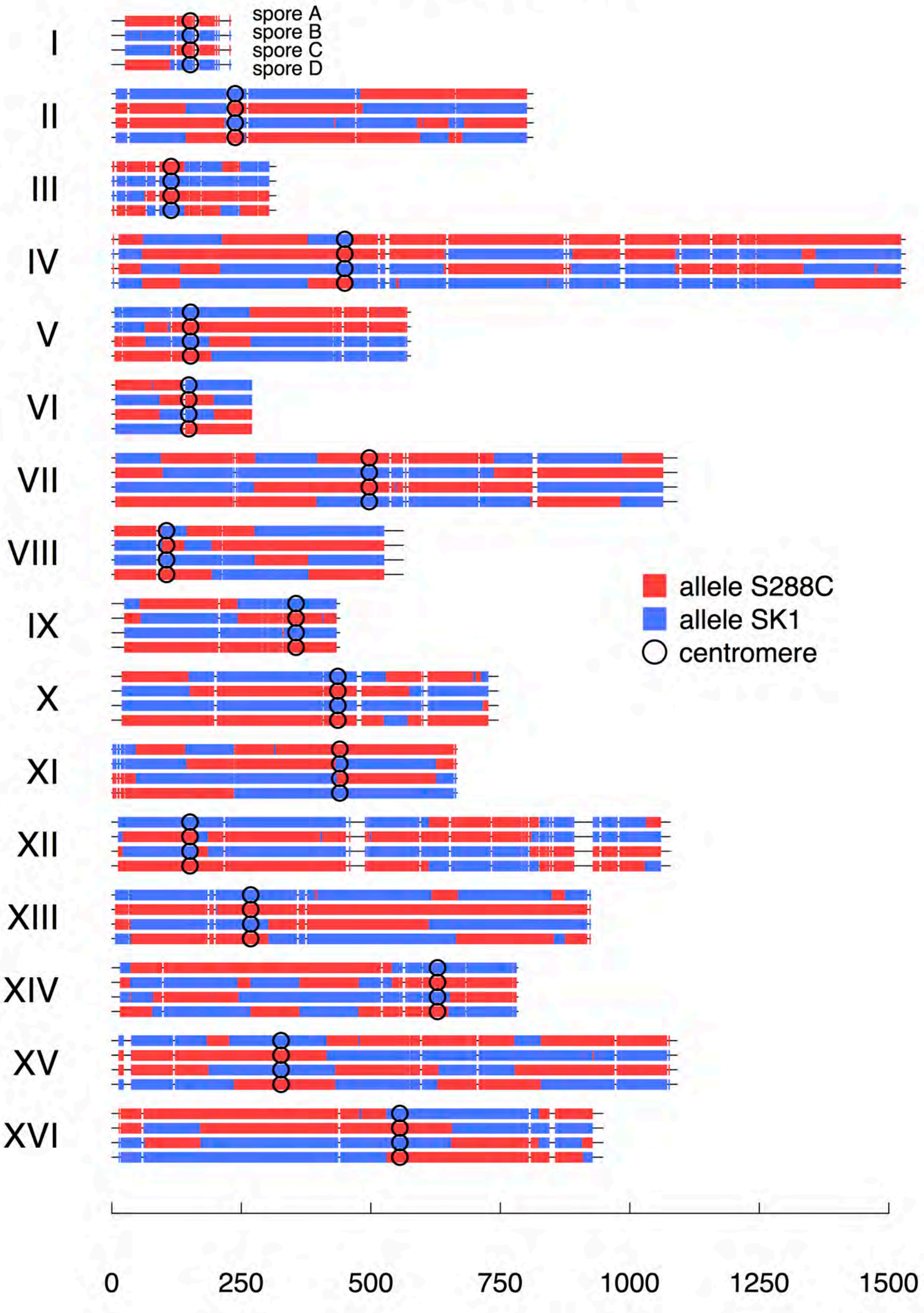

I
II
III
IV
V
VI
VII
VIII
IX
X
XI
XII
XIII
XIV
XV
XVI
spore A
spore B
spore C
spore D
allele S288C
allele SK1
centromere
0
250
500
750
1000
1250
1500

WT_tetrad_16

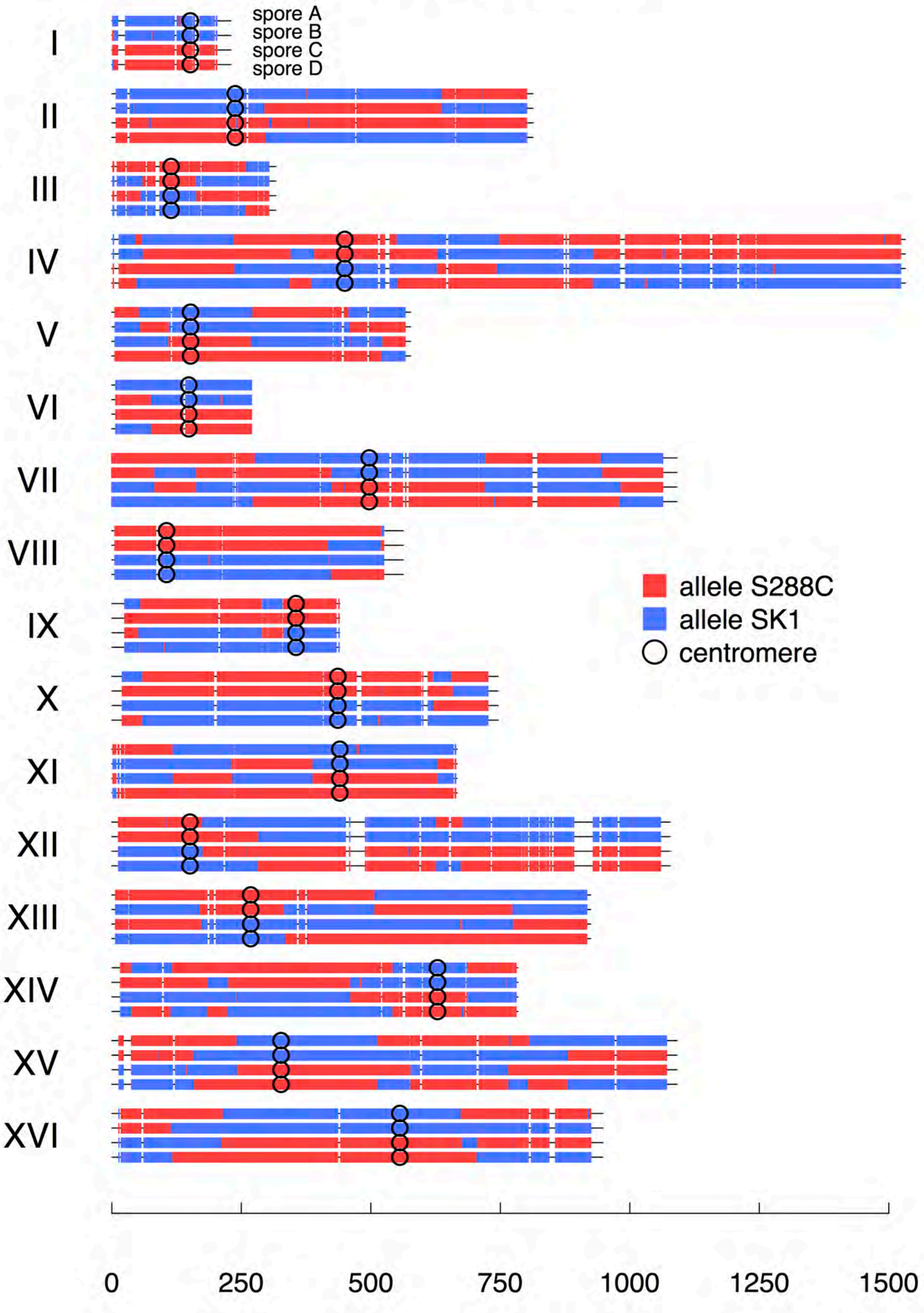

I
II
III
IV
V
VI
VII
VIII
IX
X
XI
XII
XIII
XIV
XV
XVI
spore A
spore B
spore C
spore D
allele S288C
allele SK1
centromere
0
250
500
750
1000
1250
1500

WT_tetrad_17

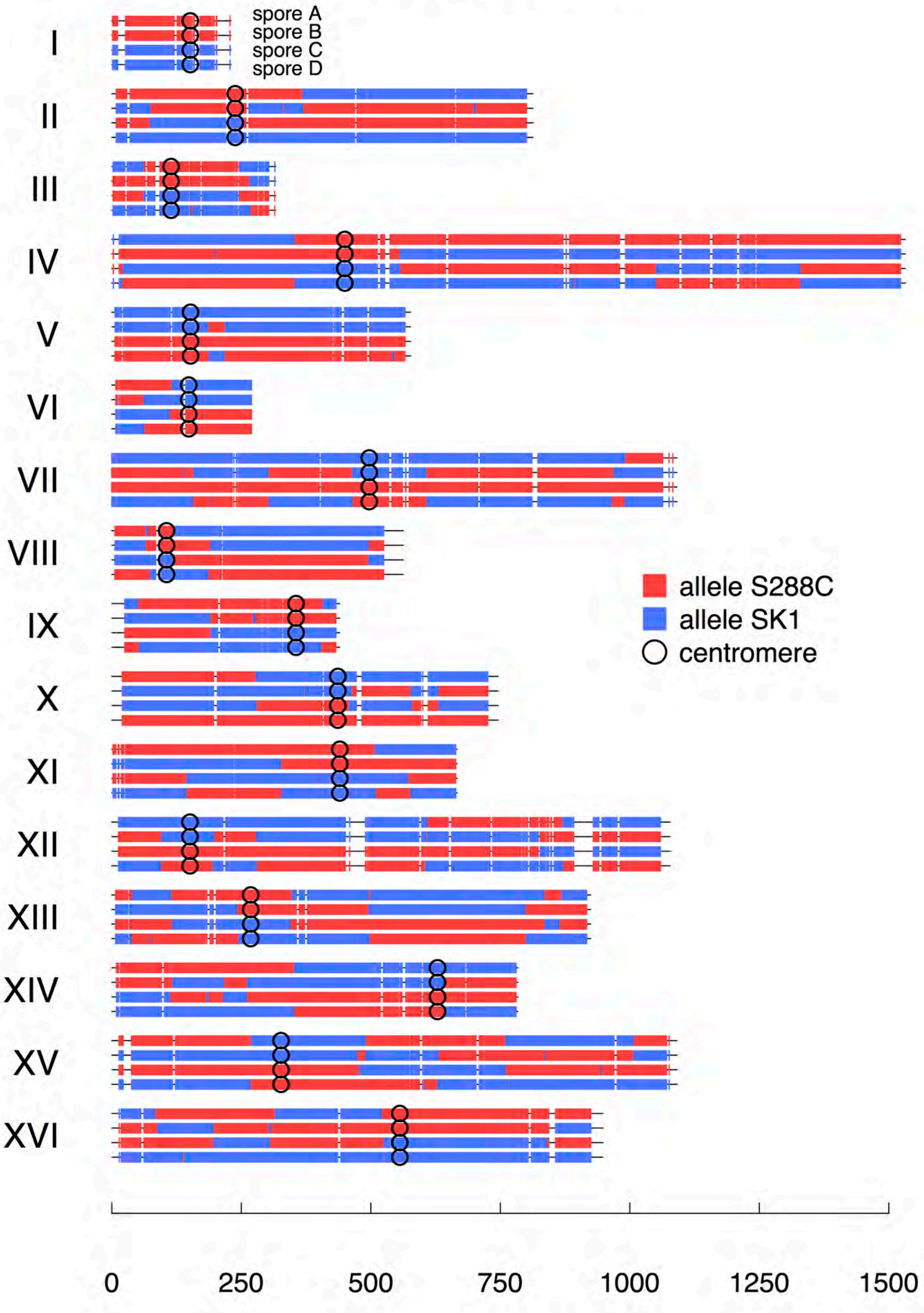
spore A
spore B
spore C
spore D
I
II
III
IV
V
VI
VII
VIII
IX
X
XI
XII
XIII
XIV
XV
XVI
allele S288C
allele SK1
centromere
0
250
500
750
1000
1250
1500

WT_tetrad_18

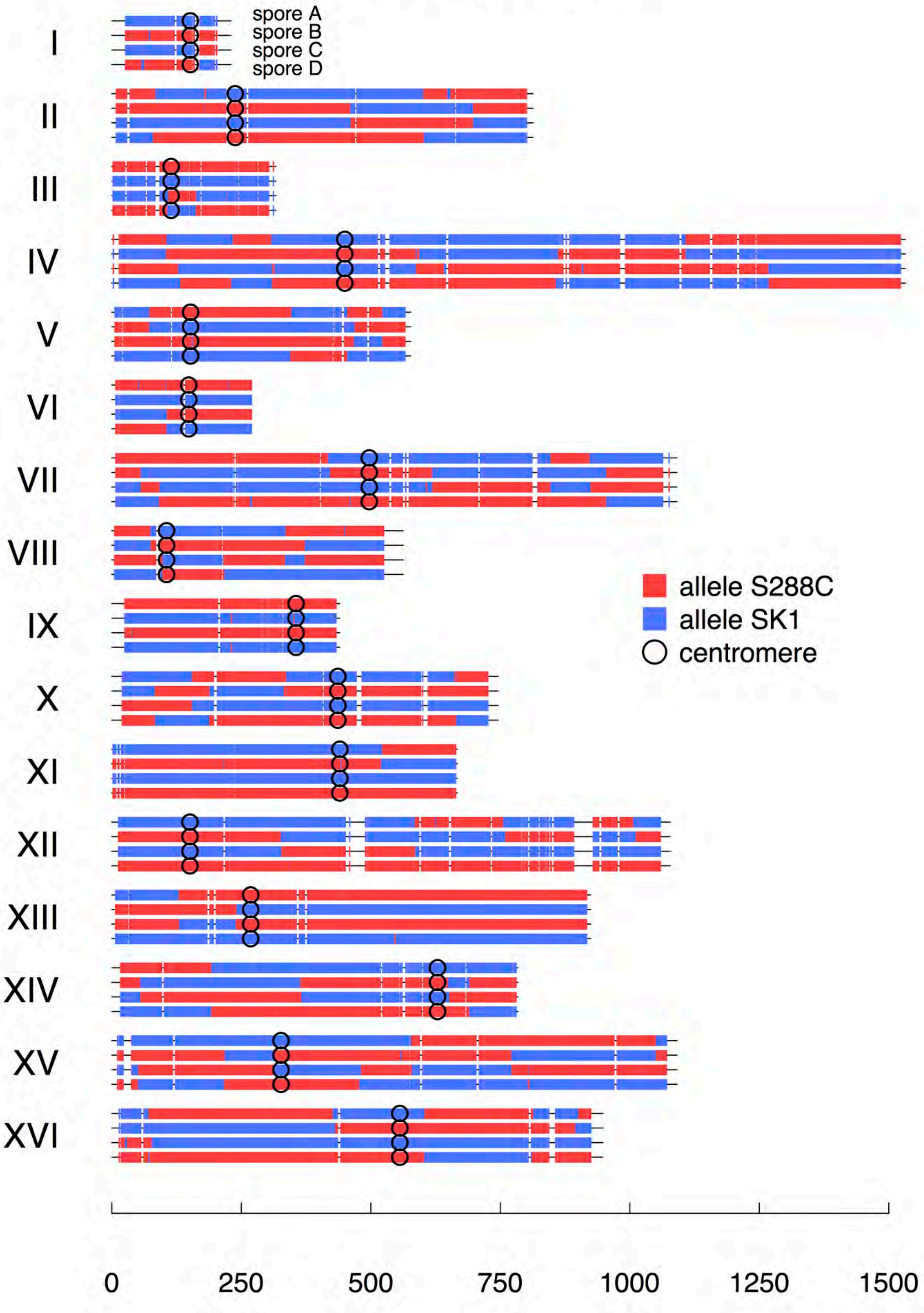

spore A
spore B
spore C
spore D
I
II
III
IV
V
VI
VII
VIII
IX
X
XI
XII
XIII
XIV
XV
XVI
allele S288C
allele SK1
centromere
0
250
500
750
1000
1250
1500

WT_tetrad_19

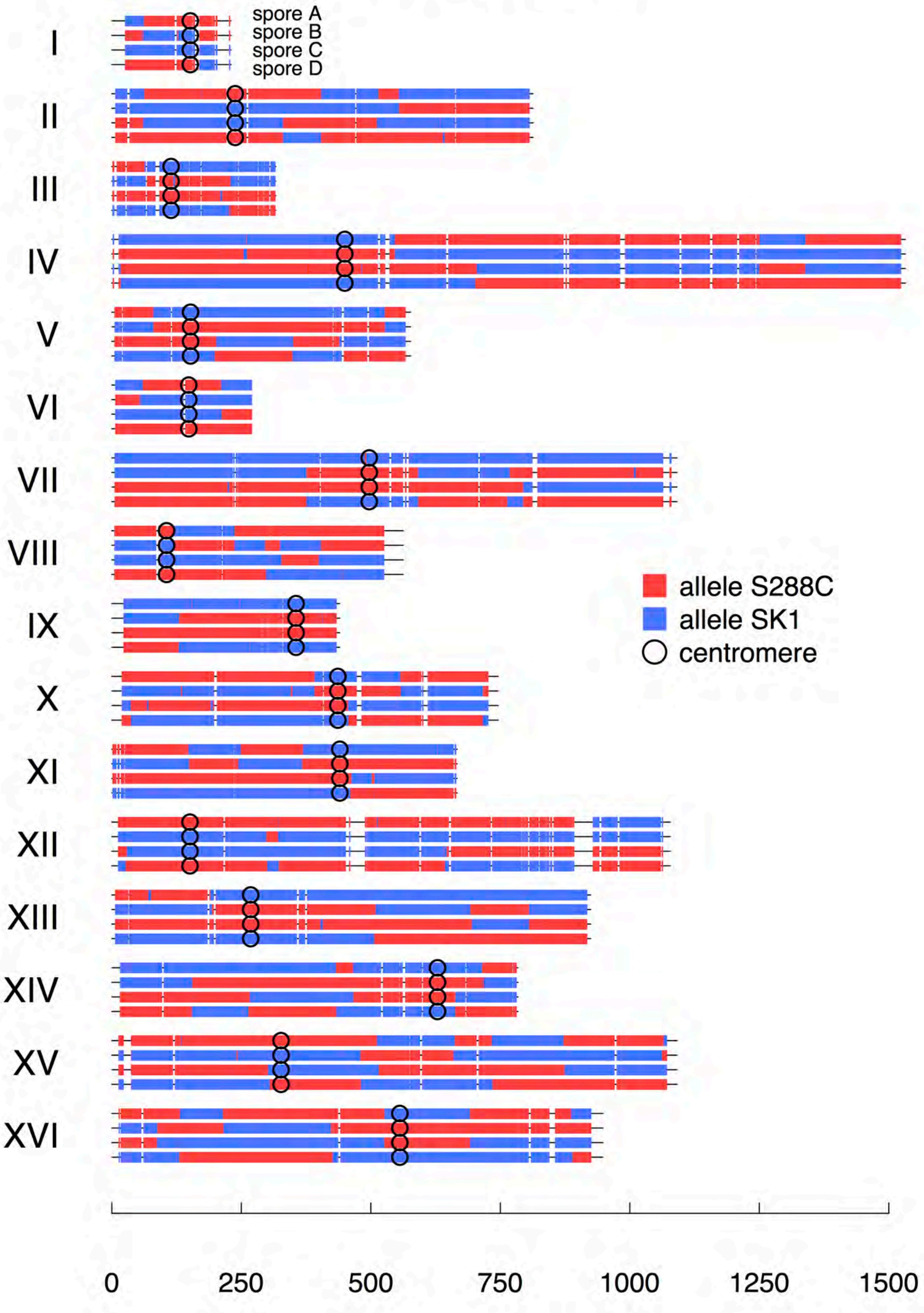

I
II
III
IV
V
VI
VII
VIII
IX
X
XI
XII
XIII
XIV
XV
XVI
spore A
spore B
spore C
spore D
allele S288C
allele SK1
centromere
0
250
500
750
1000
1250
1500

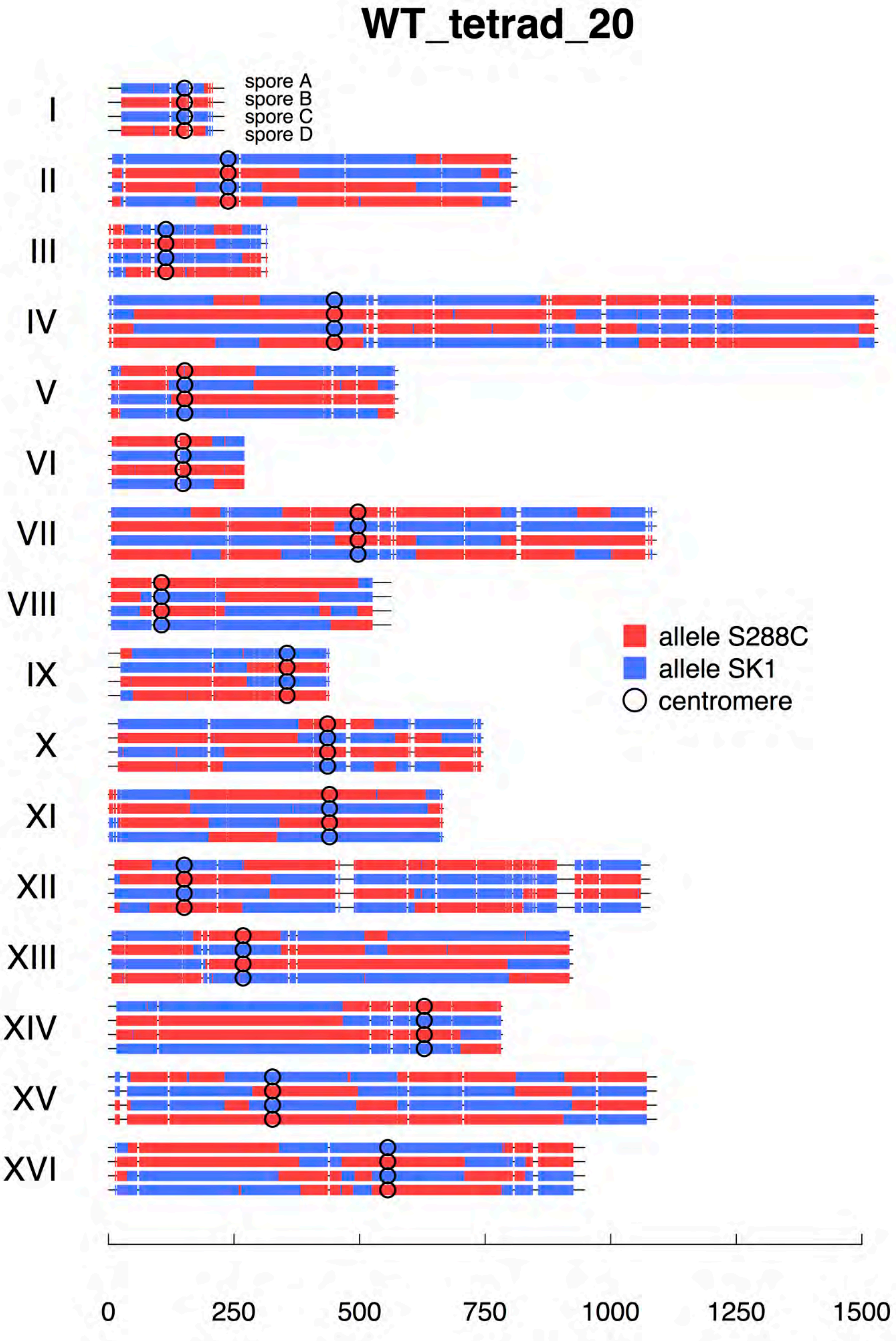
WT_tetrad_20
spore A
spore B
spore C
spore D
I
II
III
IV
V
VI
VII
VIII
IX
X
XI
XII
XIII
XIV
XV
XVI
allele S288C
allele SK1
centromere
0
250
500
750
1000
1250
1500

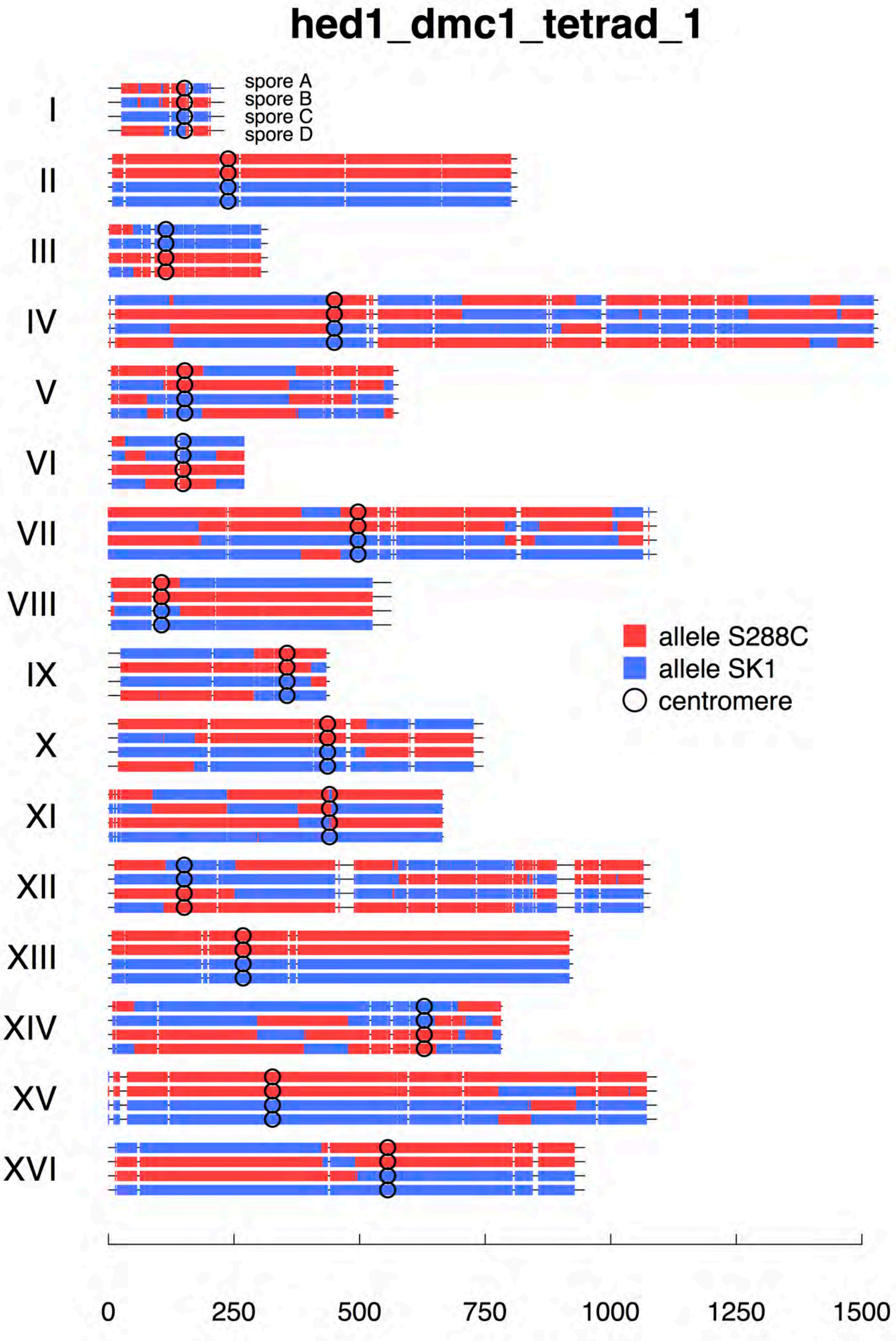
hed1_dmc1_tetrad_1
spore A
spore B
spore C
spore D
I
II
III
IV
V
VI
VII
VIII
IX
X
XI
XII
XIII
XIV
XV
XVI
allele S288C
allele SK1
centromere
0
250
500
750
1000
1250
1500

hed1_dmc1_tetrad_2

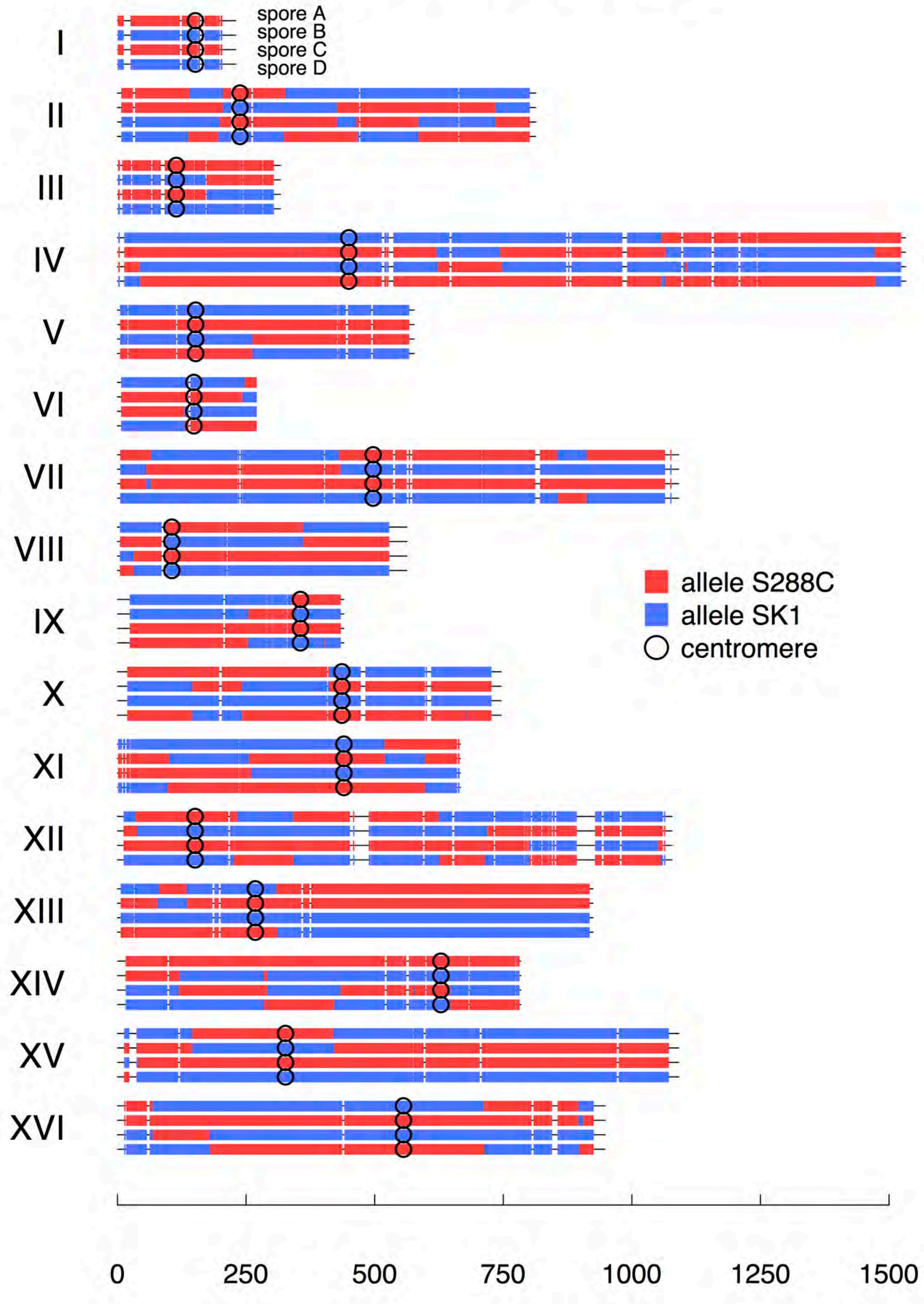

hed1_dmc1_tetrad_3

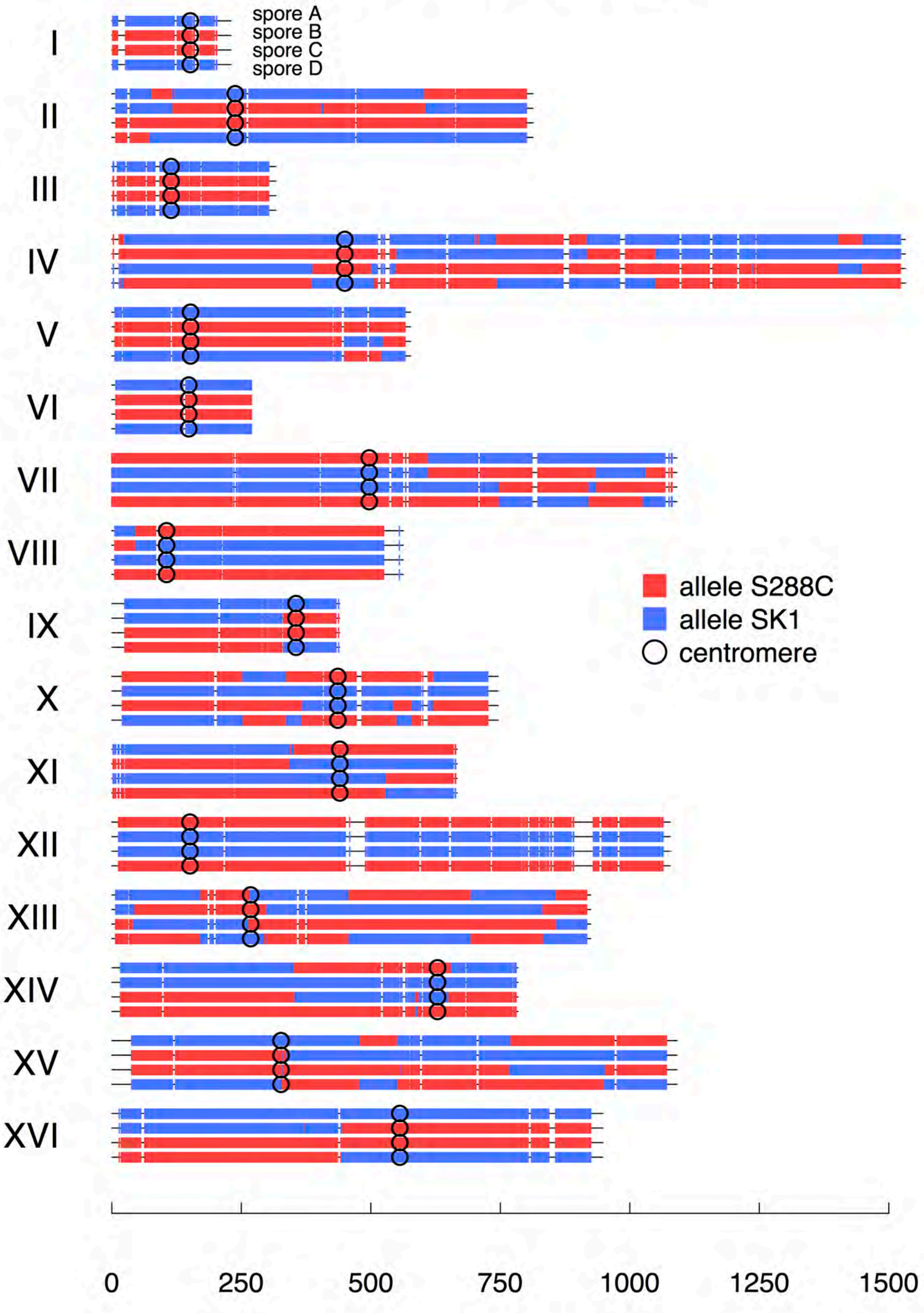

hed1_dmc1_tetrad_4

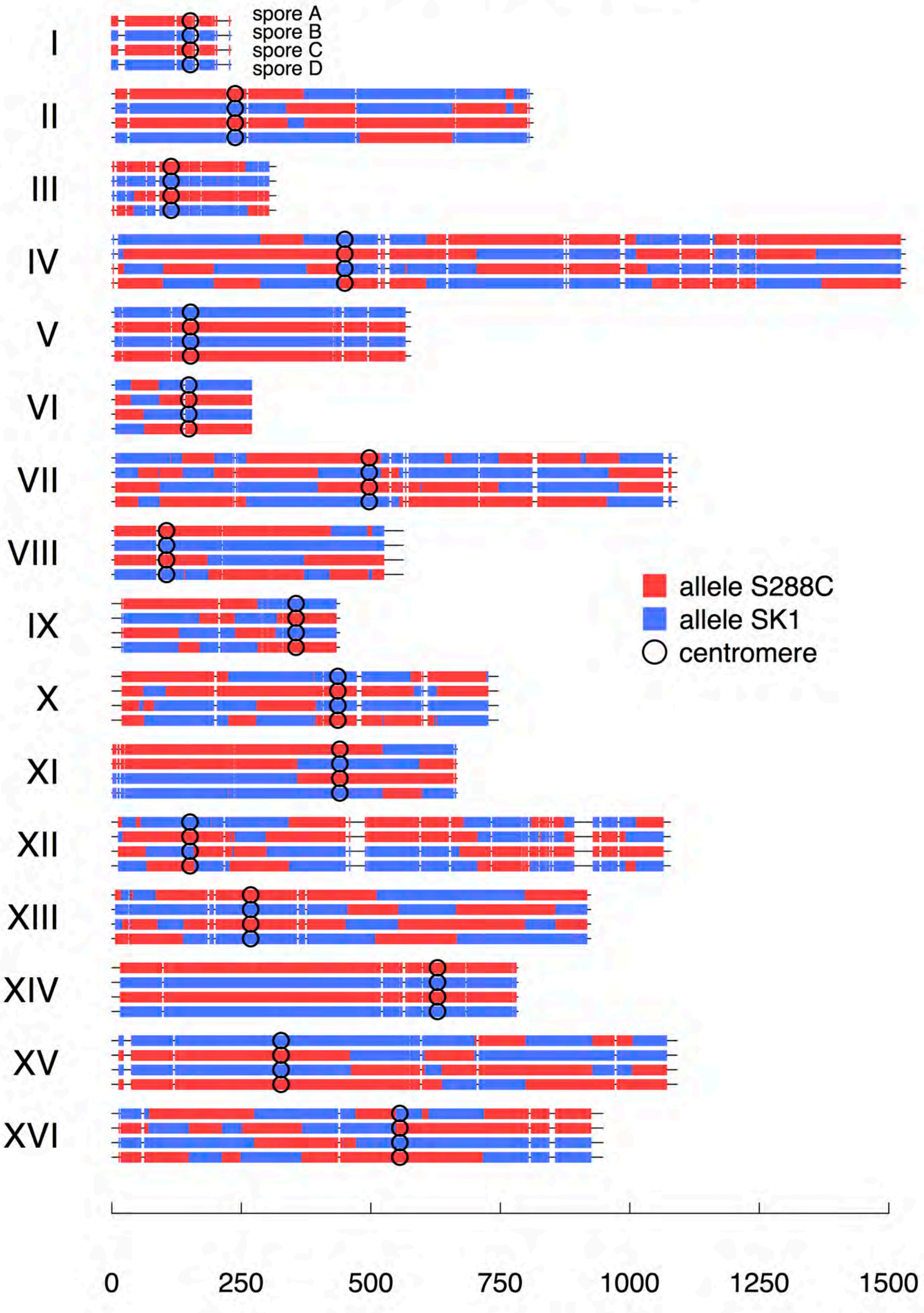

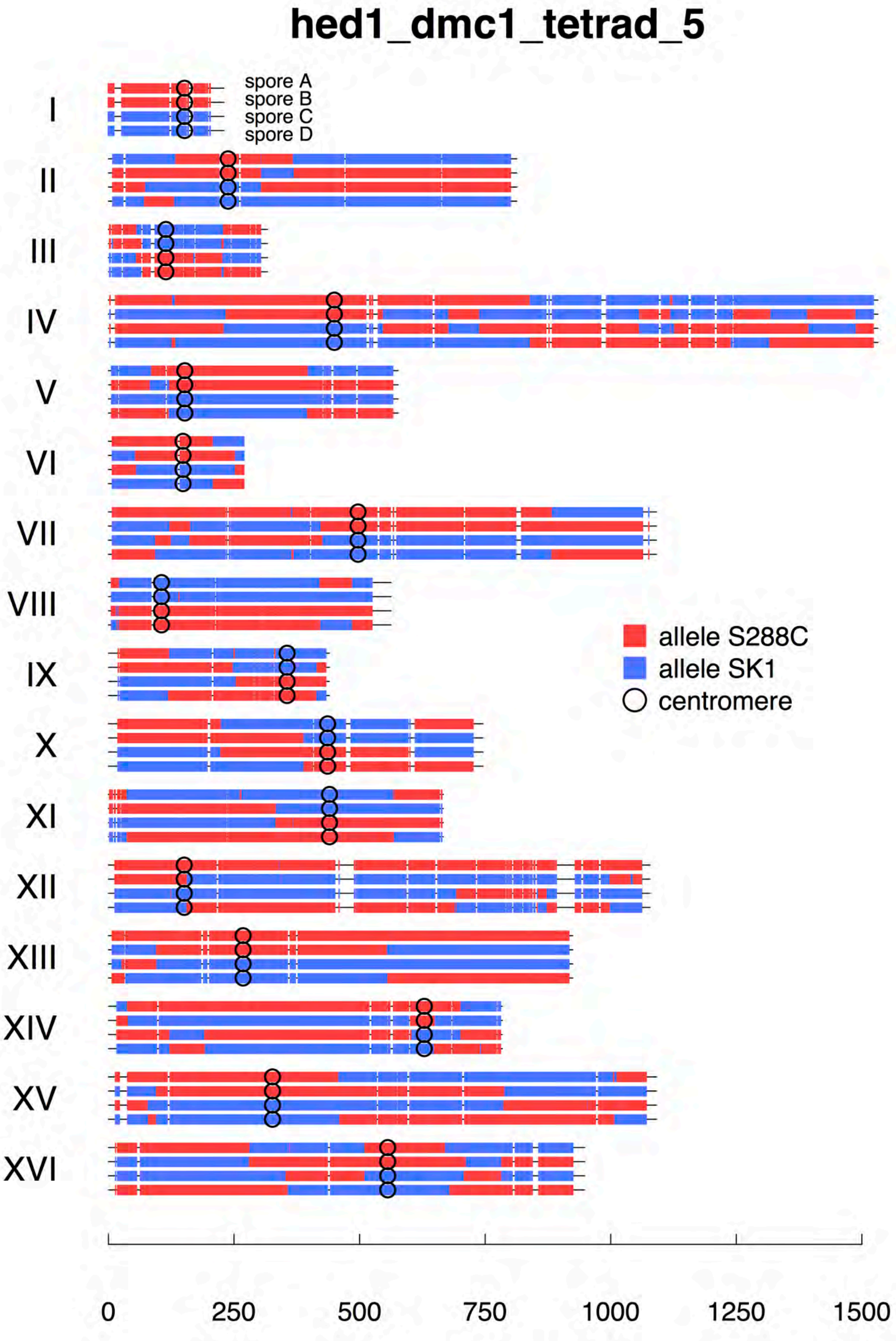

hed1_dmc1_tetrad_5
spore A
spore B
spore C
spore D
I
II
III
IV
V
VI
VII
VIII
IX
X
XI
XII
XIII
XIV
XV
XVI
allele S288C
allele SK1
centromere
0
250
500
750
1000
1250
1500

hed1_dmc1_tetrad_6

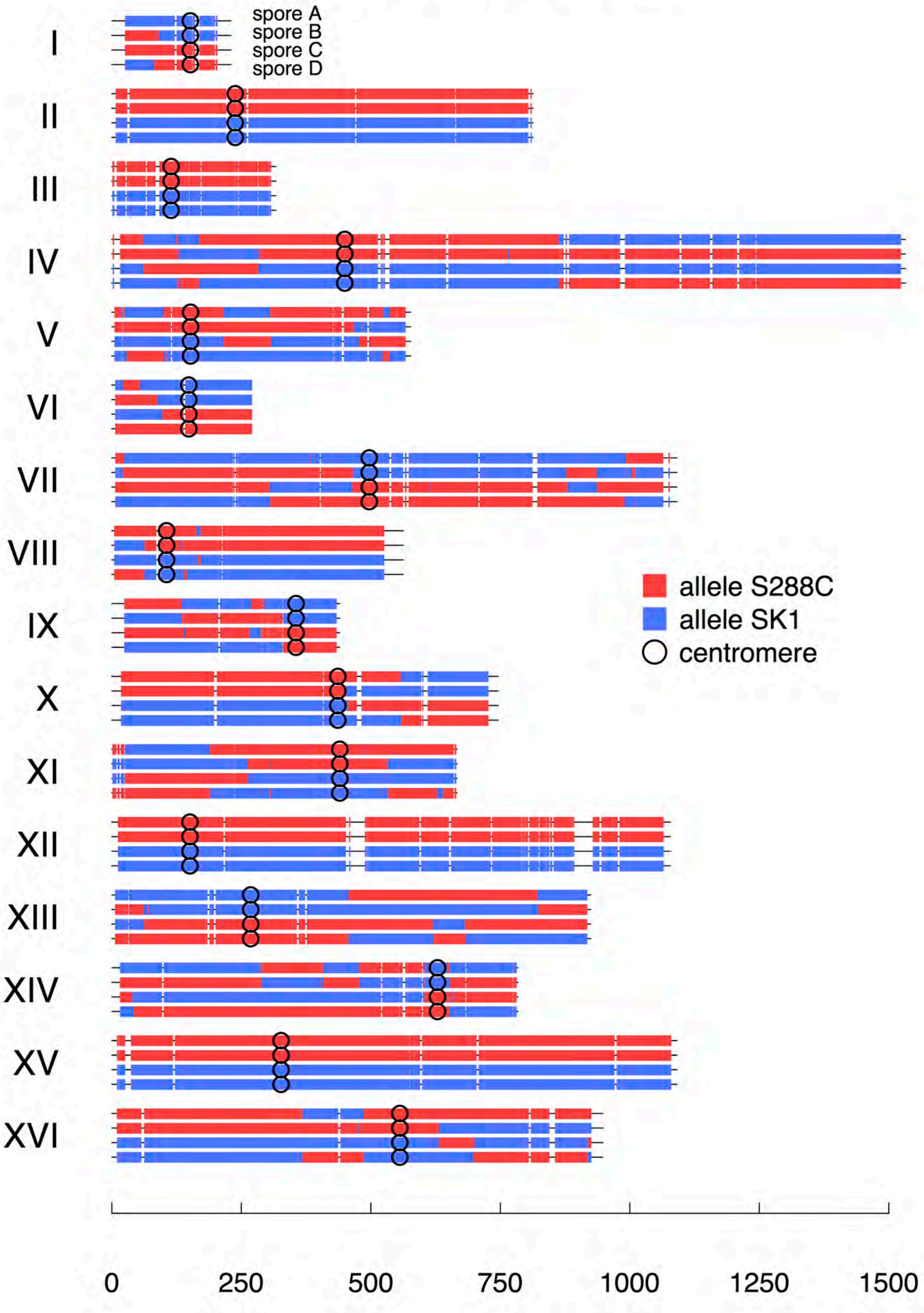

hed1_dmc1_tetrad_7

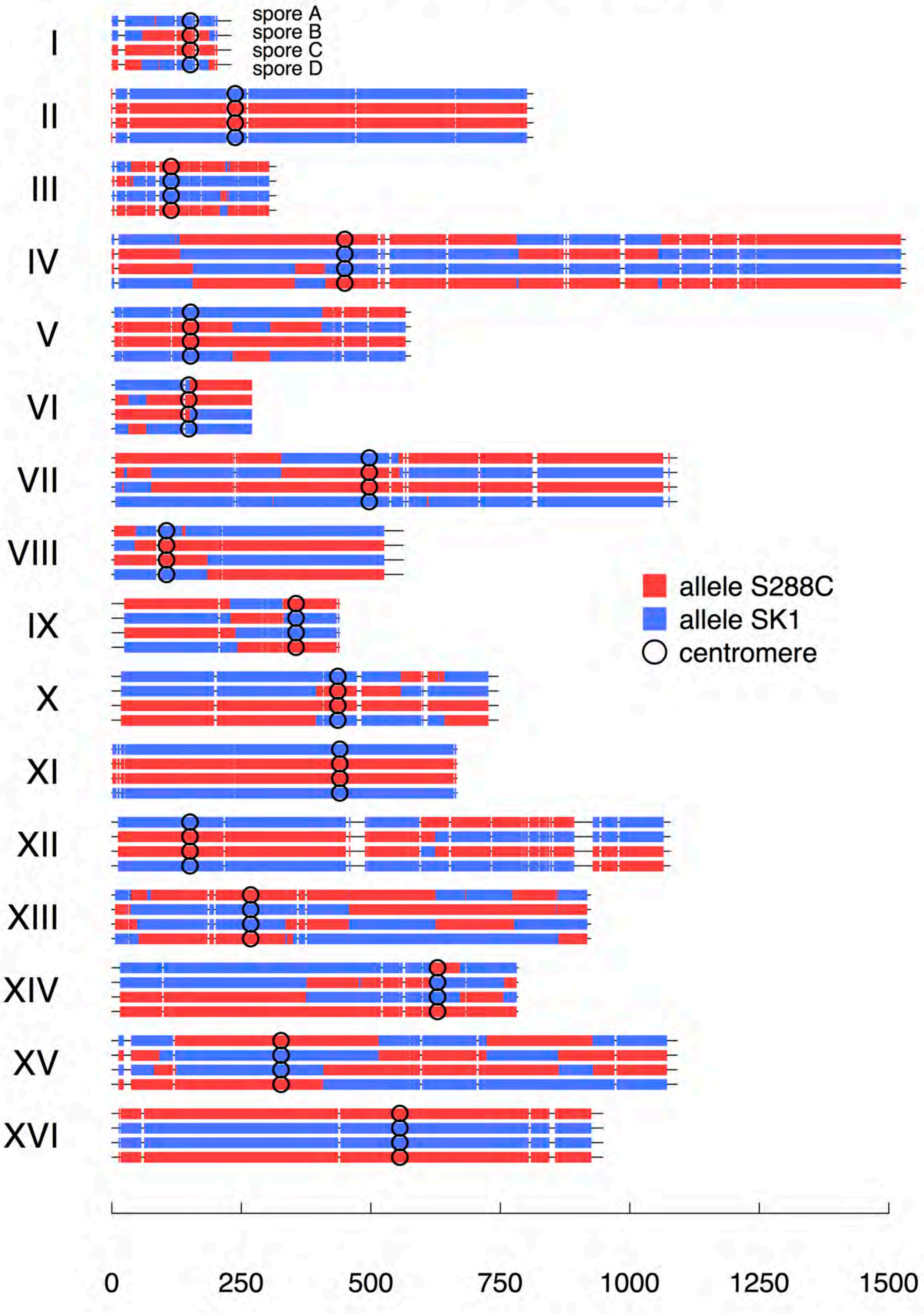

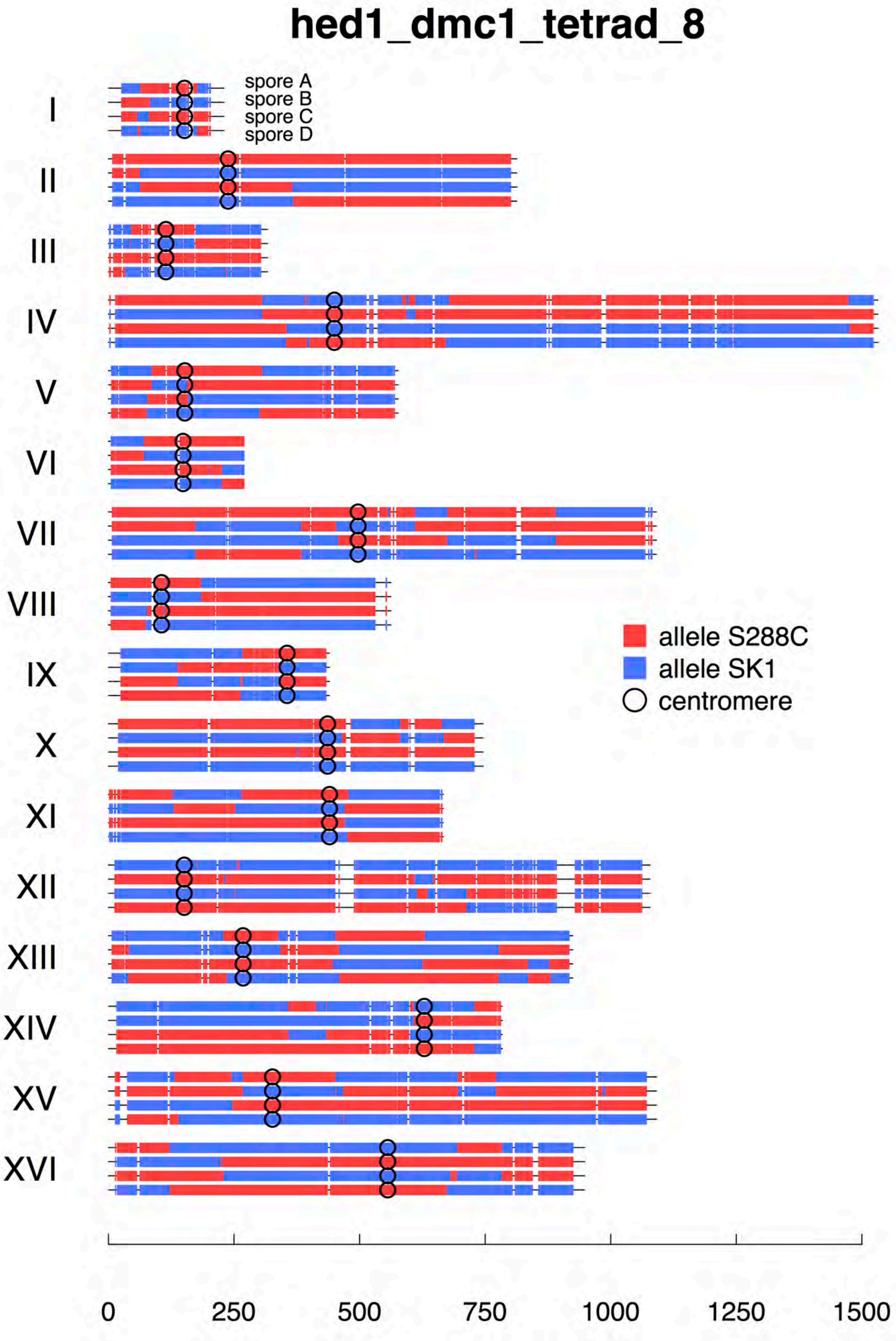

hed1_dmc1_tetrad_8
I
II
III
IV
V
VI
VII
VIII
IX
X
XI
XII
XIII
XIV
XV
XVI
spore A
spore B
spore C
spore D
allele S288C
allele SK1
centromere
0
250
500
750
1000
1250
1500

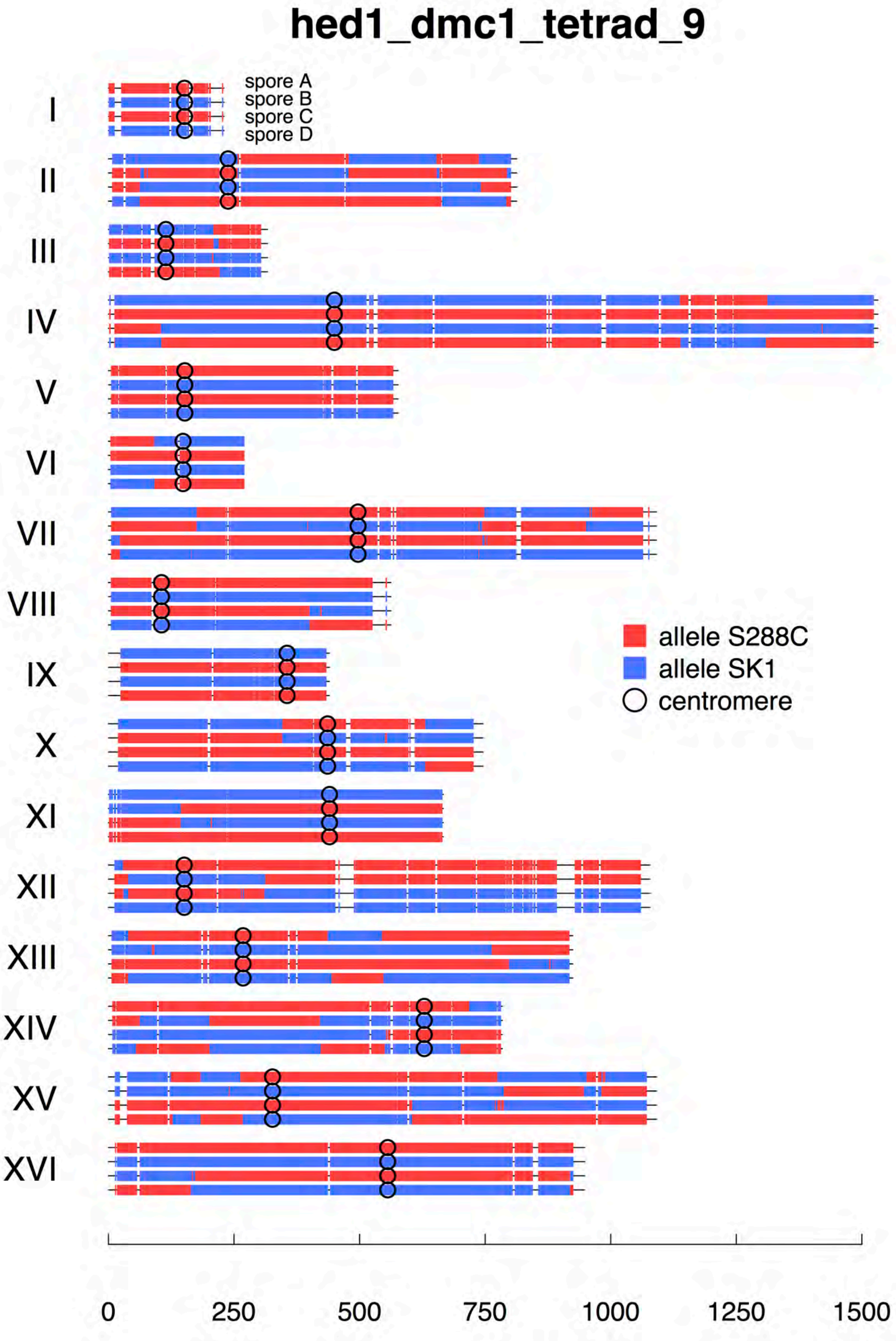
hed1_dmc1_tetrad_9
spore A
spore B
spore C
spore D
I
II
III
IV
V
VI
VII
VIII
IX
X
XI
XII
XIII
XIV
XV
XVI
allele S288C
allele SK1
centromere
0
250
500
750
1000
1250
1500

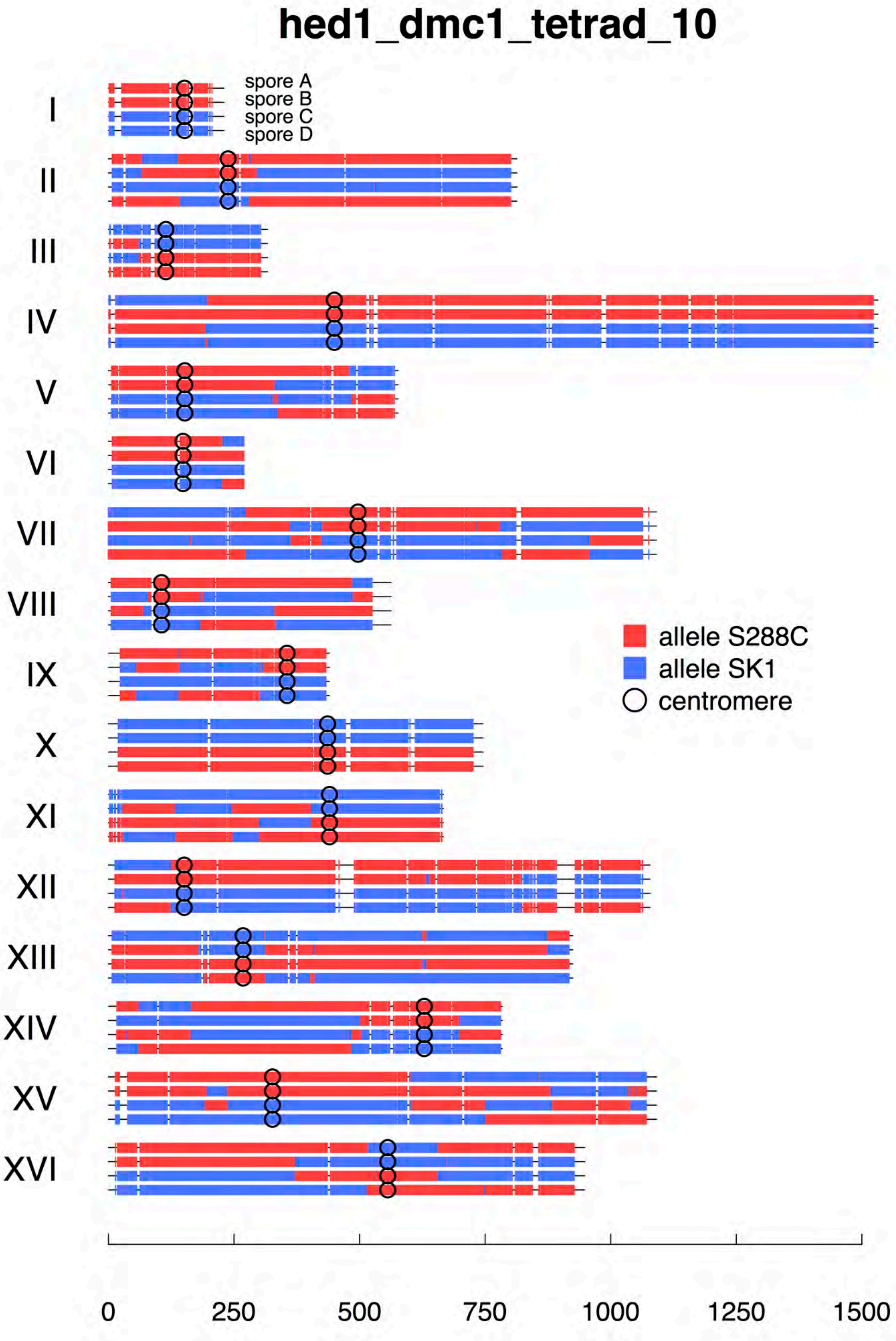
hed1_dmc1_tetrad_10
spore A
spore B
spore C
spore D
I
II
III
IV
V
VI
VII
VIII
IX
X
XI
XII
XIII
XIV
XV
XVI
allele S288C
allele SK1
centromere
0
250
500
750
1000
1250
1500

hed1_dmc1_tetrad_11

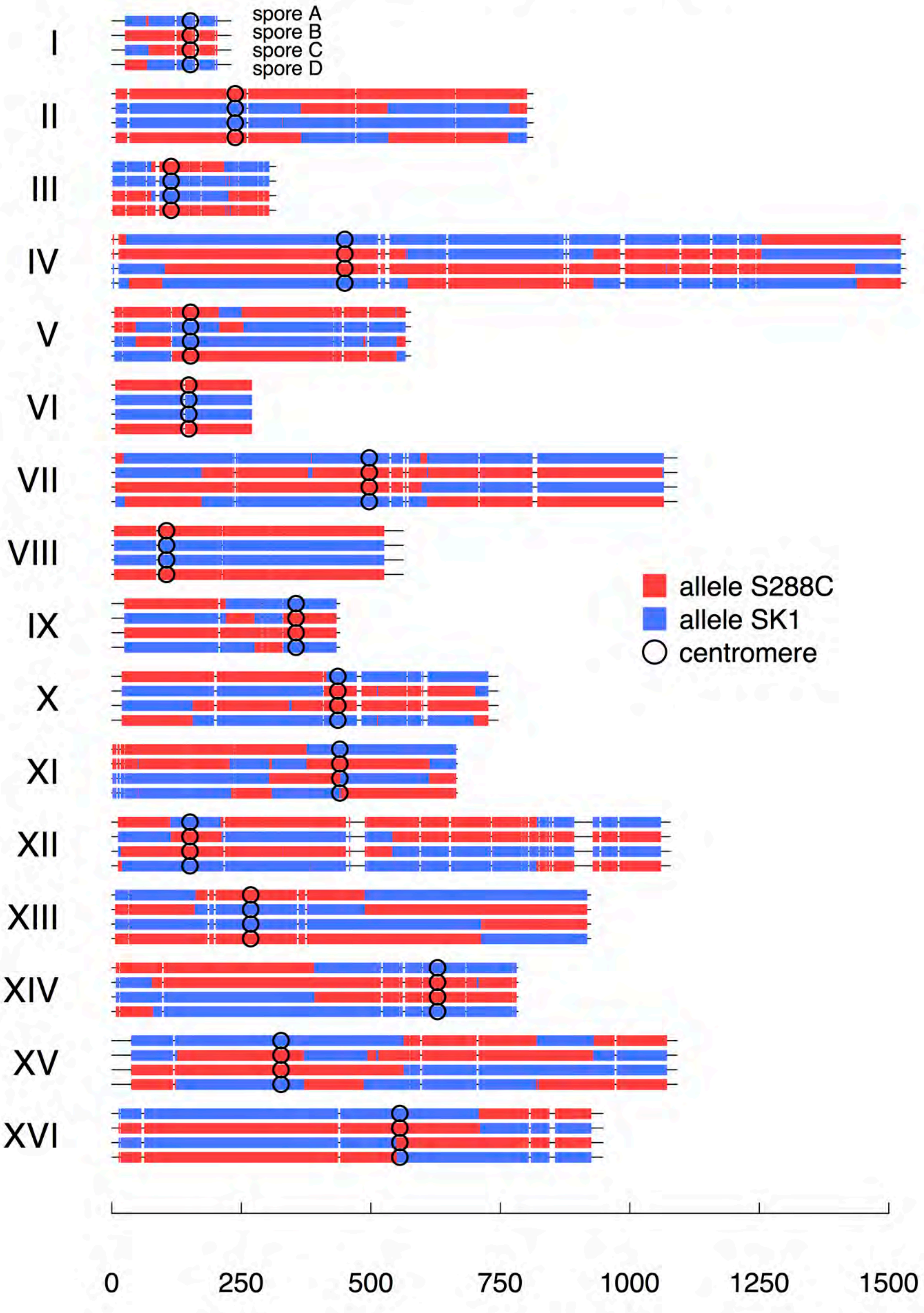

hed1_dmc1_tetrad_12

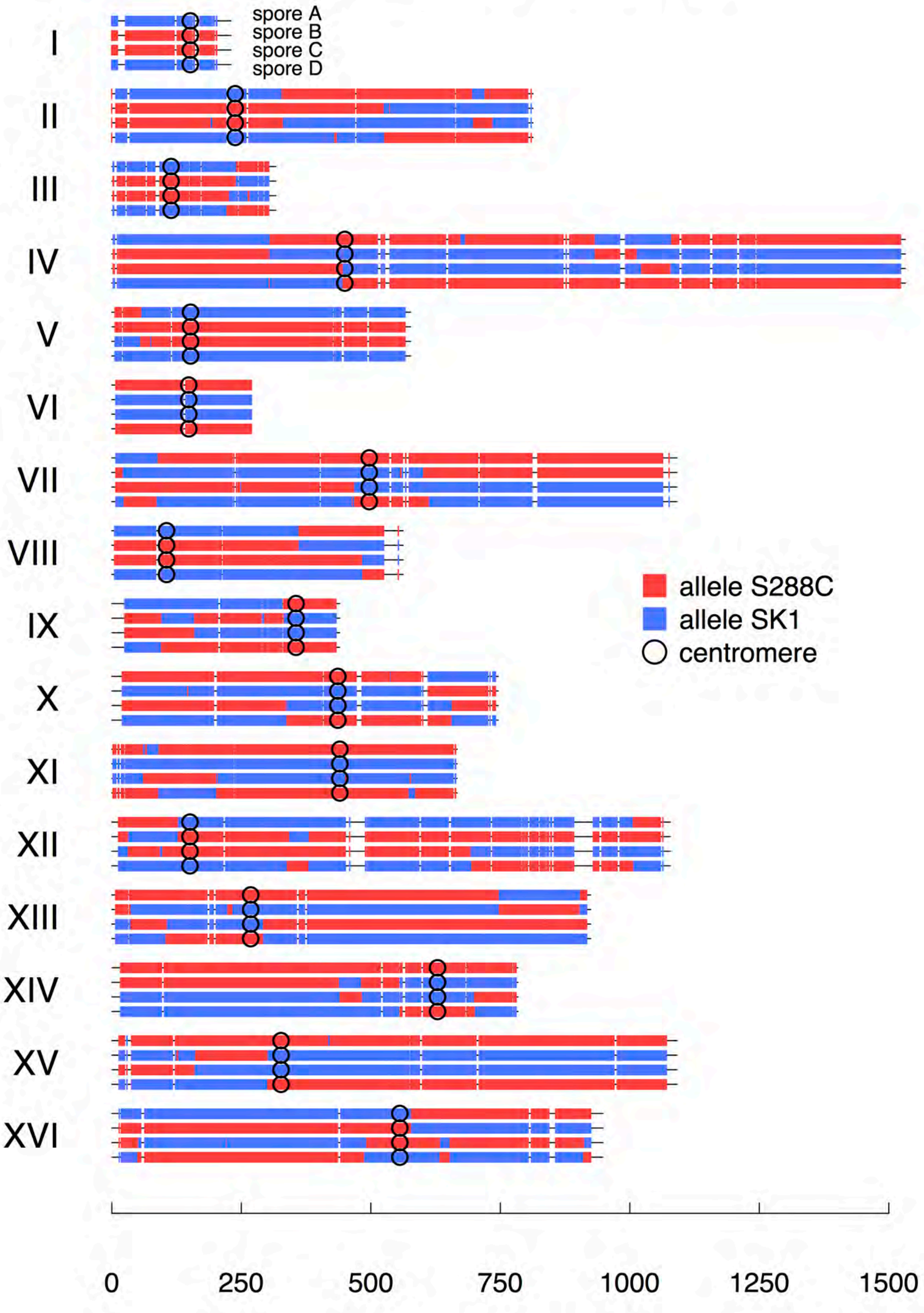

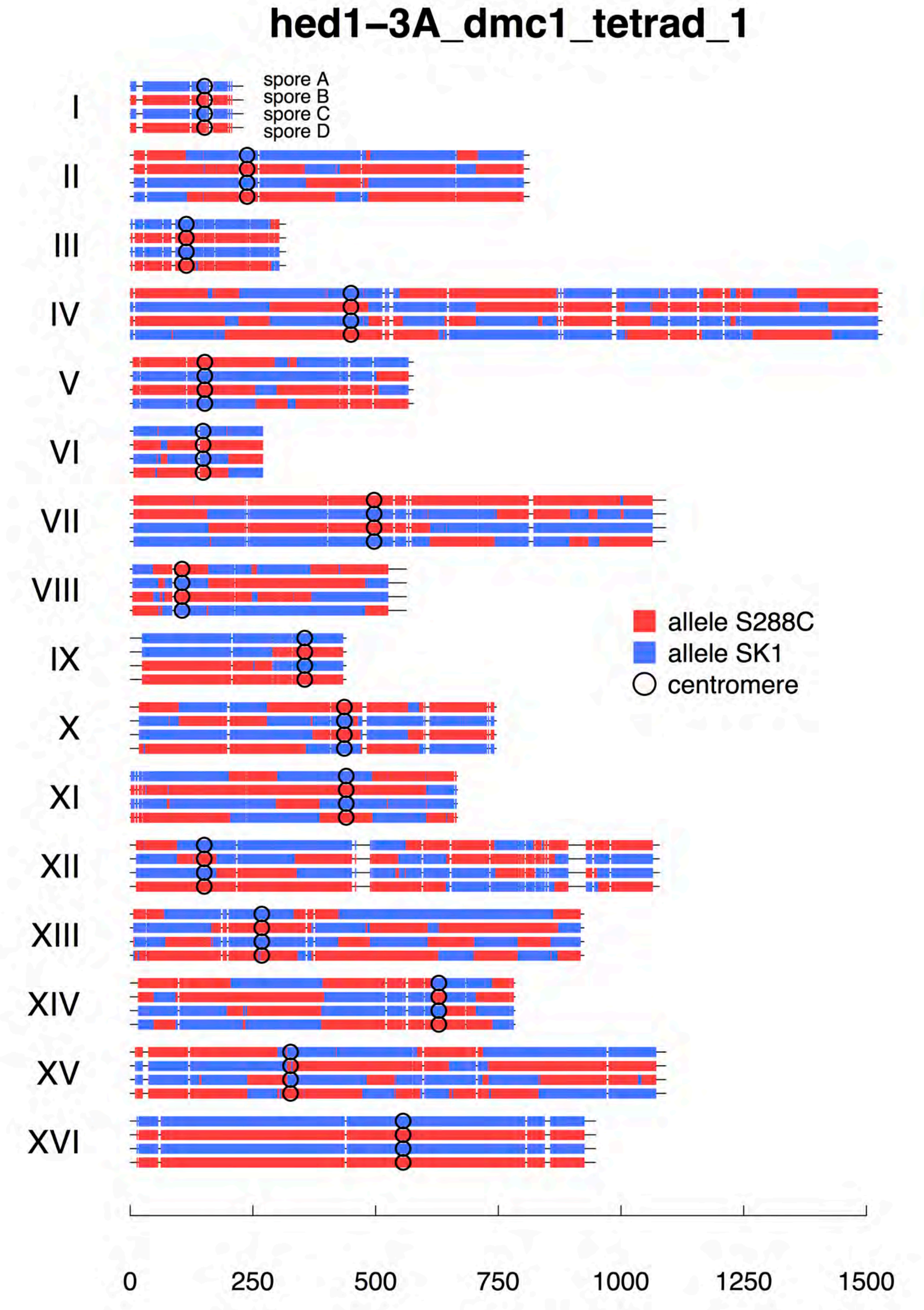
hed1−3A_dmc1_tetrad_1
spore A
spore B
spore C
spore D
I
II
III
IV
V
VI
VII
VIII
IX
X
XI
XII
XIII
XIV
XV
XVI
allele S288C
allele SK1
centromere
0
250
500
750
1000
1250
1500

hed1–3A_dmc1_tetrad_2

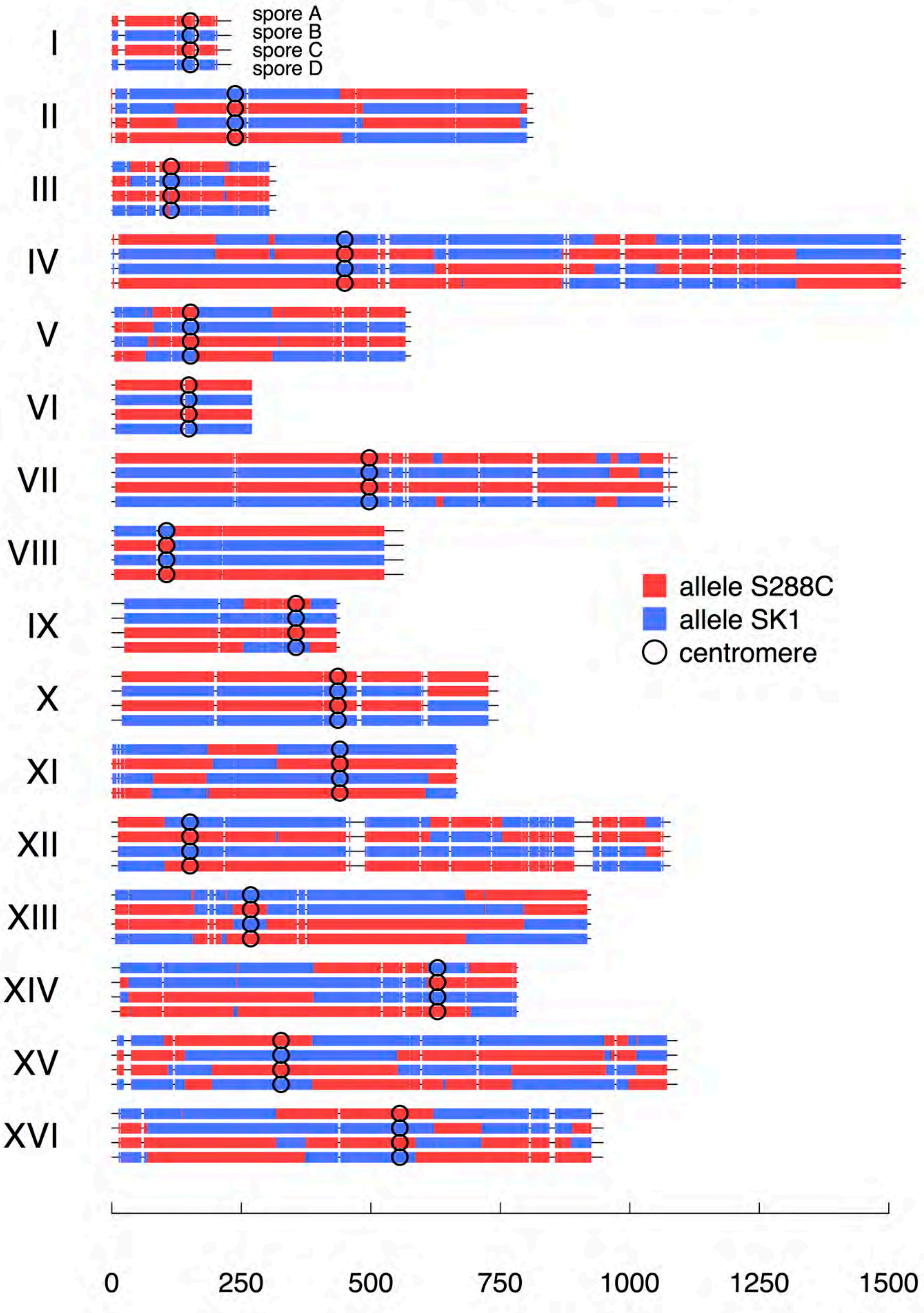

hed1-3A_dmc1_tetrad_3

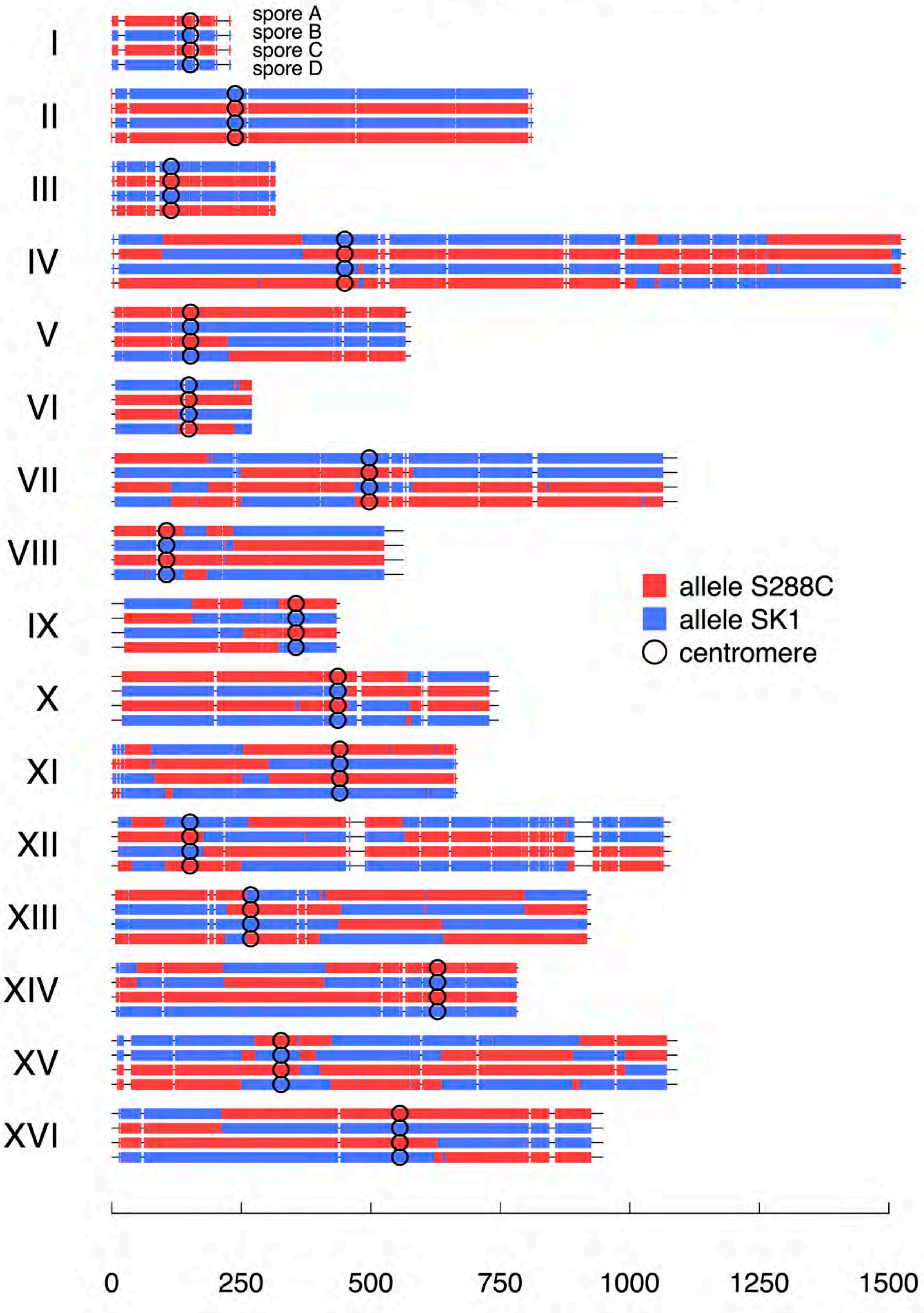

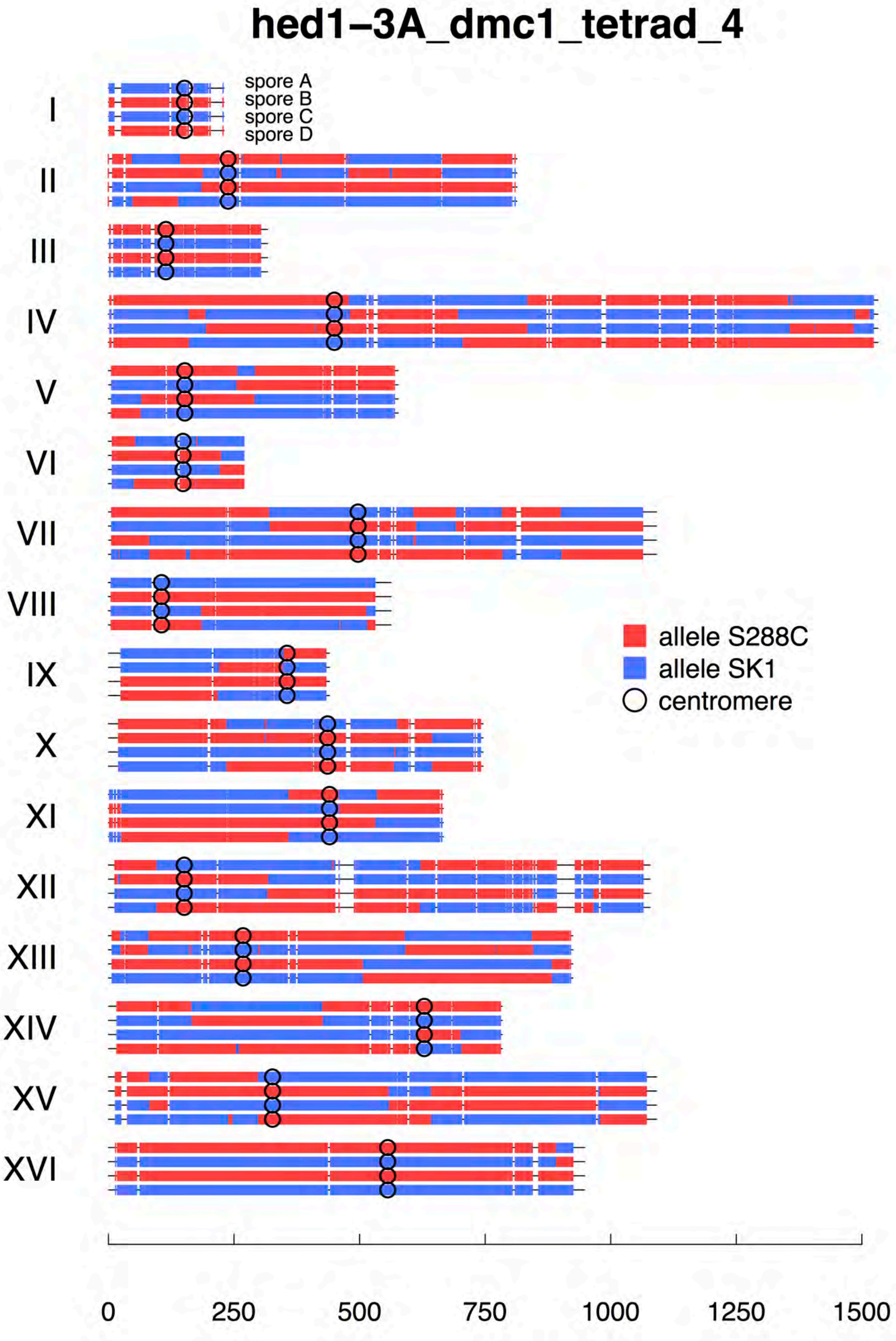
hed1–3A_dmc1_tetrad_4
spore A
spore B
spore C
spore D
I
II
III
IV
V
VI
VII
VIII
IX
X
XI
XII
XIII
XIV
XV
XVI
allele S288C
allele SK1
centromere
0
250
500
750
1000
1250
1500

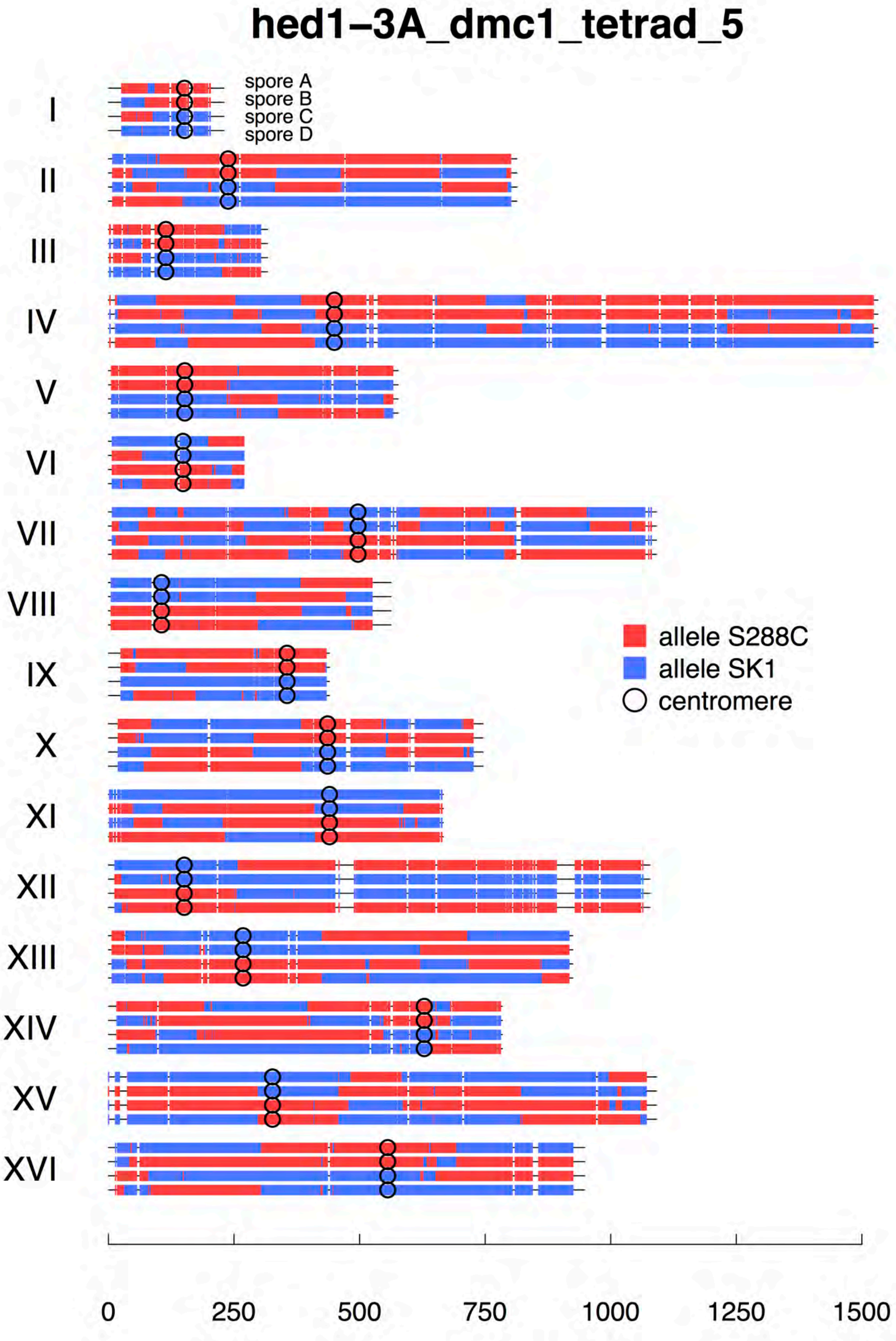
hed1–3A_dmc1_tetrad_5
spore A
spore B
spore C
spore D
I
II
III
IV
V
VI
VII
VIII
IX
X
XI
XII
XIII
XIV
XV
XVI
allele S288C
allele SK1
centromere
0
250
500
750
1000
1250
1500

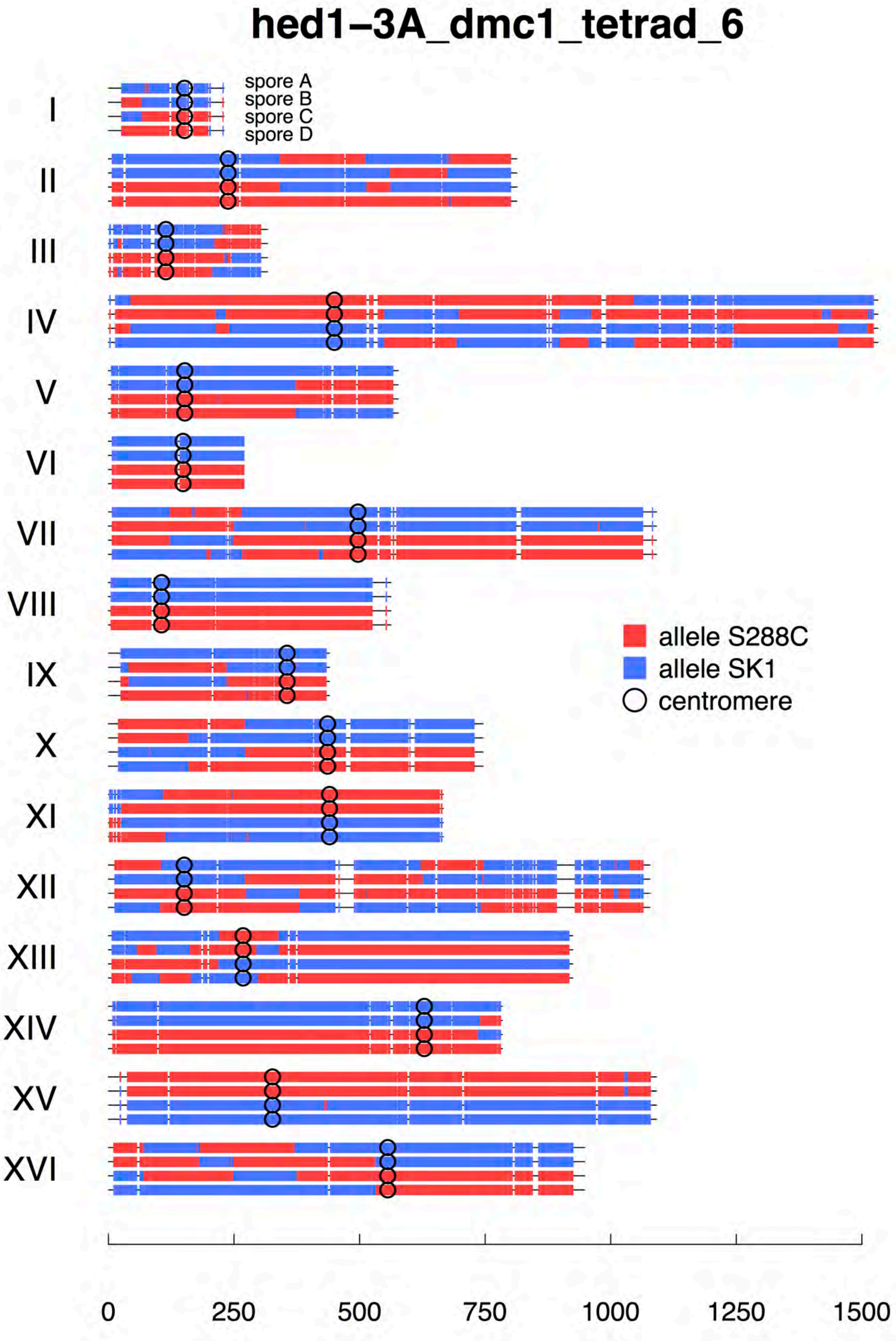
hed1−3A_dmc1_tetrad_6
spore A
spore B
spore C
spore D
I
II
III
IV
V
VI
VII
VIII
IX
X
XI
XII
XIII
XIV
XV
XVI
allele S288C
allele SK1
centromere
0
250
500
750
1000
1250
1500

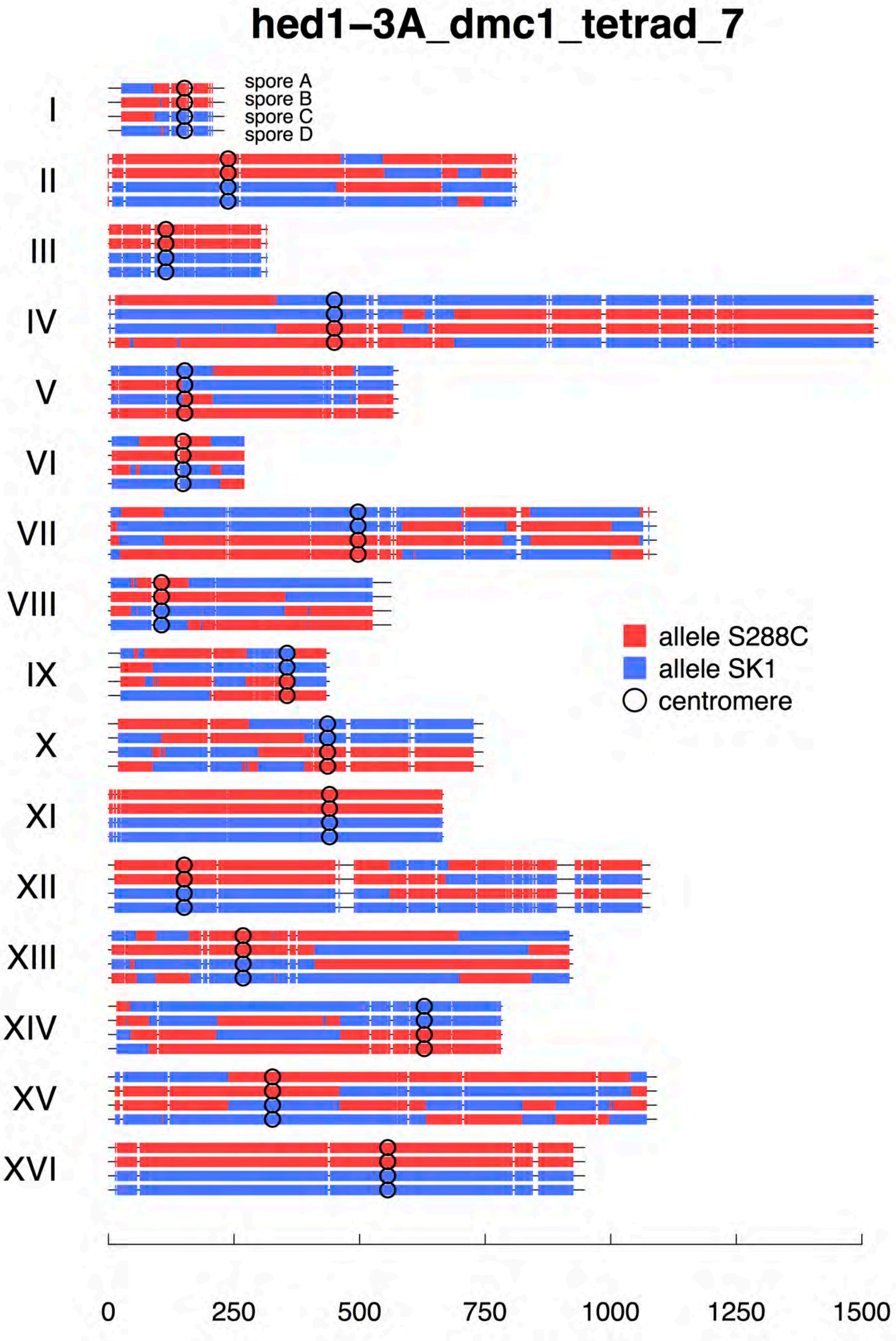
hed1–3A_dmc1_tetrad_7
spore A
spore B
spore C
spore D
I
II
III
IV
V
VI
VII
VIII
IX
X
XI
XII
XIII
XIV
XV
XVI
allele S288C
allele SK1
centromere
0
250
500
750
1000
1250
1500

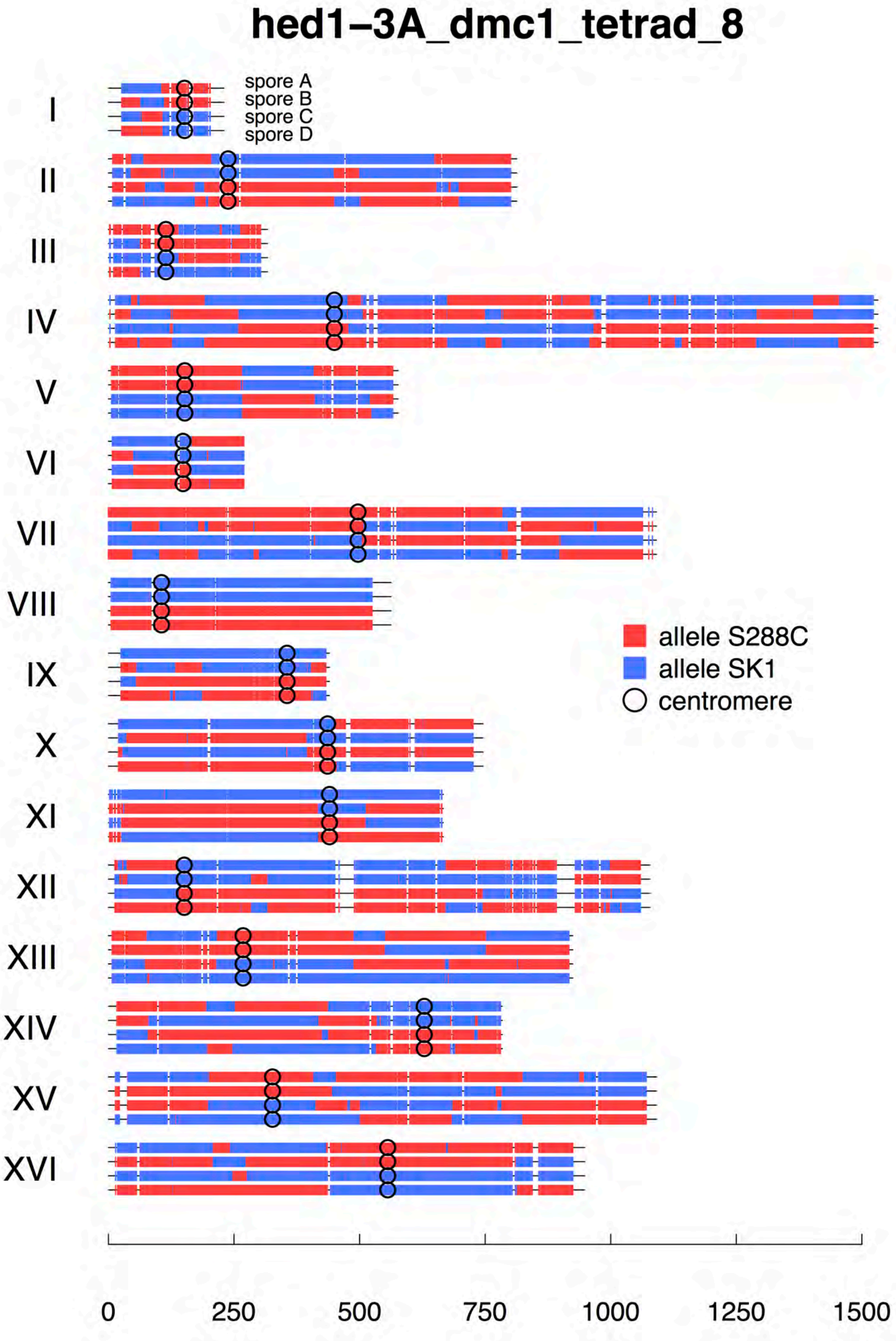

hed1–3A_dmc1_tetrad_8
spore A
spore B
spore C
spore D
I
II
III
IV
V
VI
VII
VIII
IX
X
XI
XII
XIII
XIV
XV
XVI
allele S288C
allele SK1
centromere
0
250
500
750
1000
1250
1500

hed1-3A_dmc1_tetrad_9

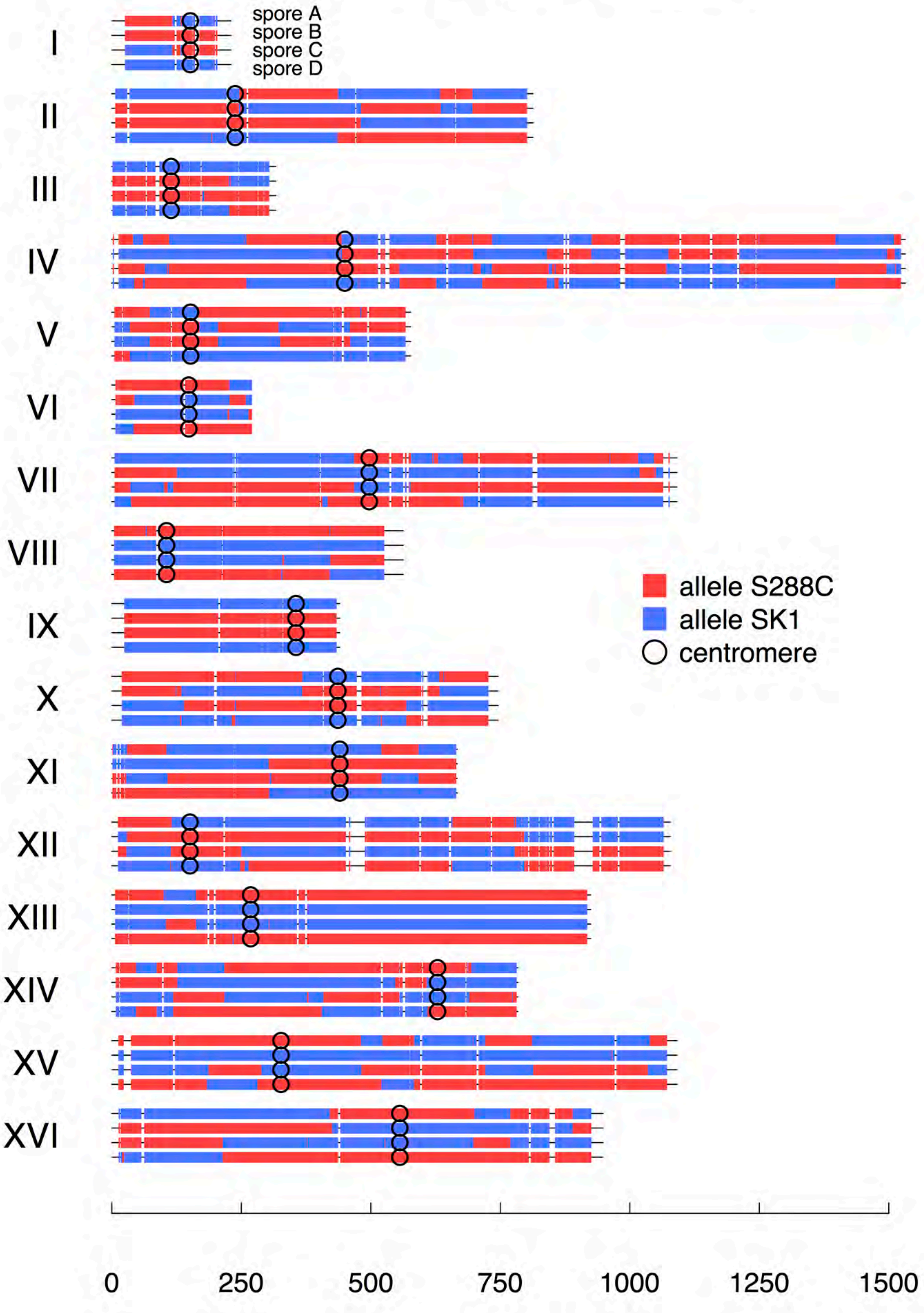

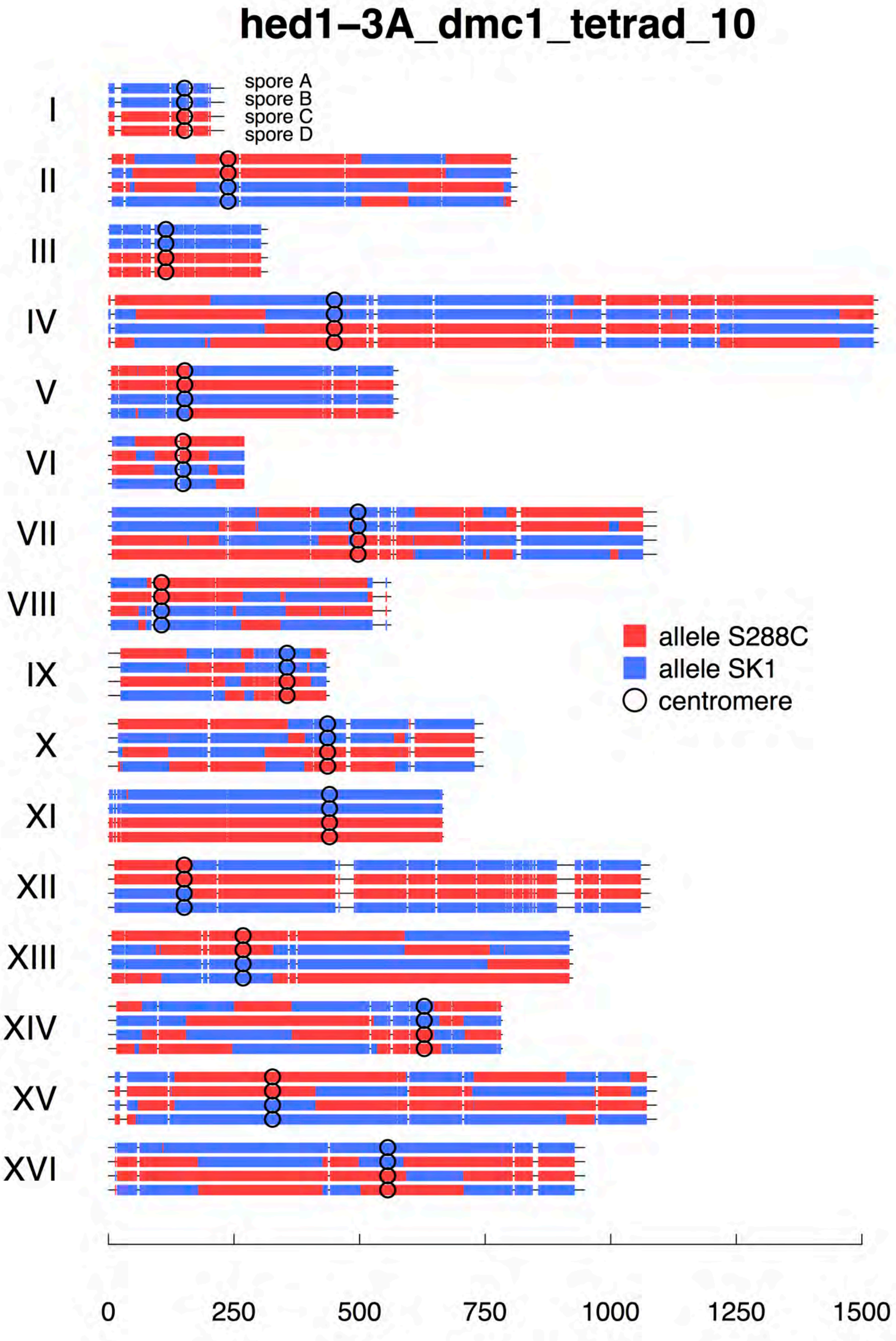

hed1–3A_dmc1_tetrad_10
spore A
spore B
spore C
spore D
I
II
III
IV
V
VI
VII
VIII
IX
X
XI
XII
XIII
XIV
XV
XVI
allele S288C
allele SK1
centromere
0
250
500
750
1000
1250
1500

hed1-3A_dmc1_tetrad_11

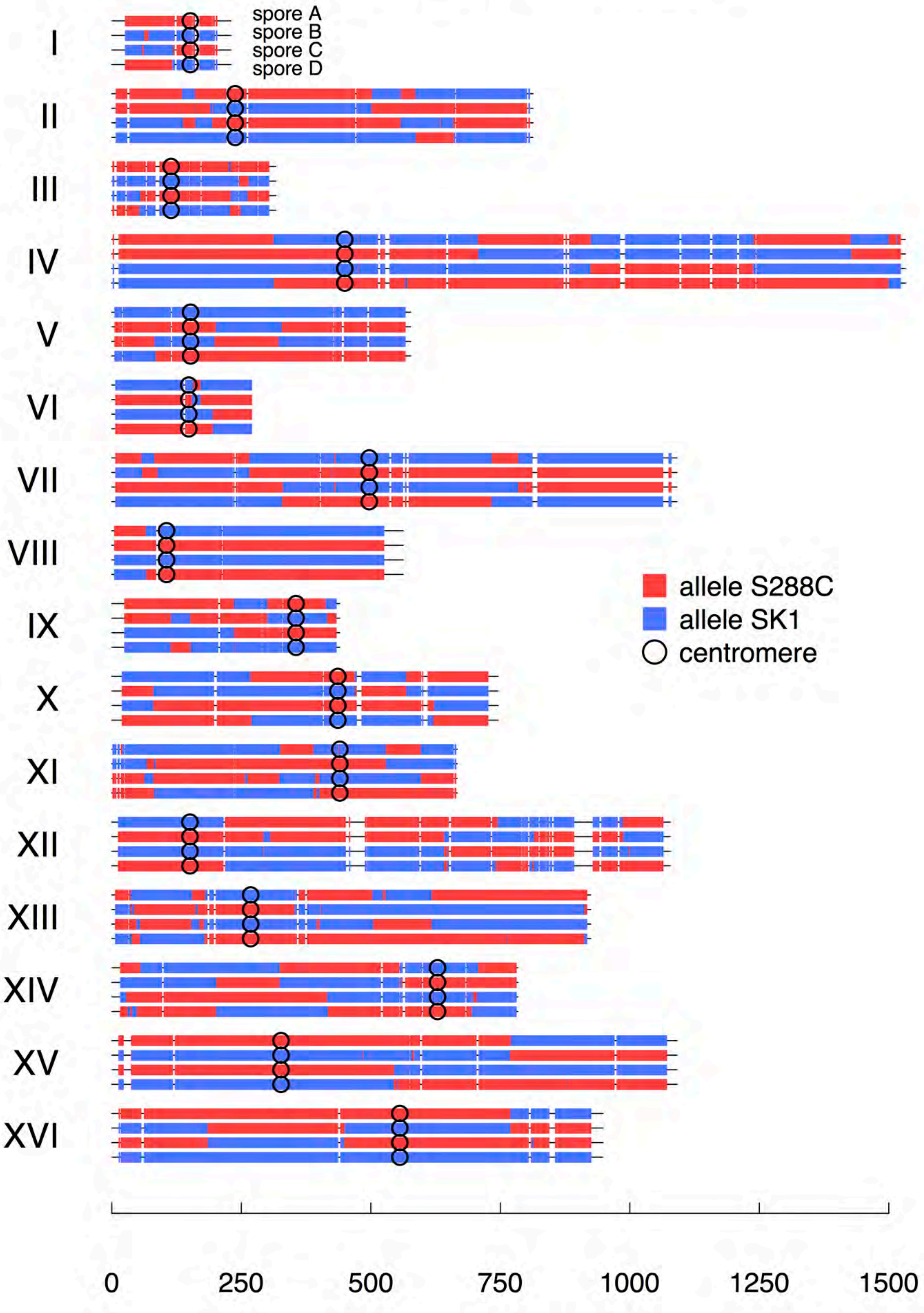

hed1–3A_dmc1_tetrad_12

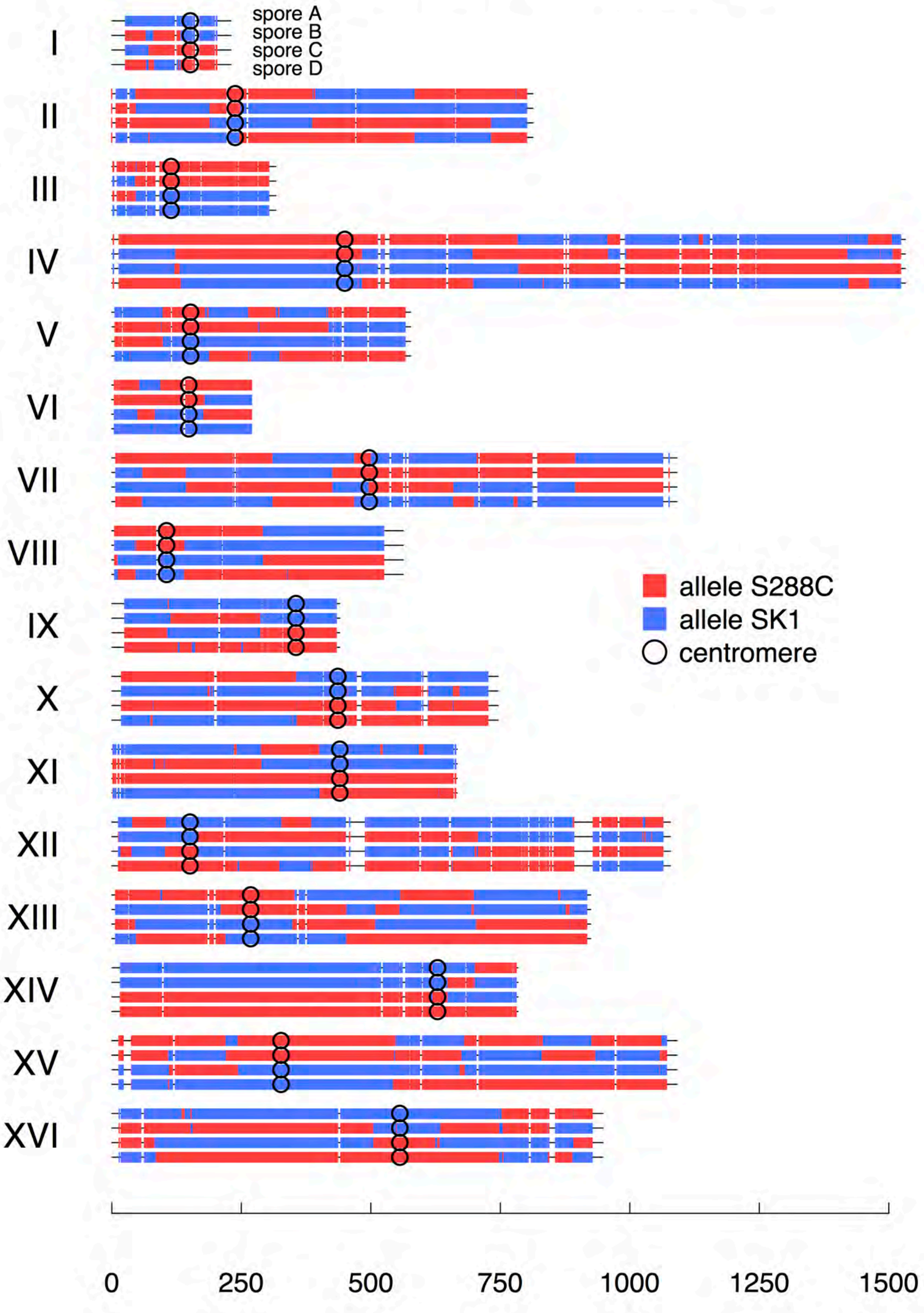

Supplement: S4 Fig — Blue indicates sequences derived from the SK1 parent, while red indicates sequence from S288c. Gaps indicate regions where that was no SNP genotype information. Black circles indicate centromeres. Chromosomes (indicated by Roman numerals) are arranged by chromosome number (I to XVI) from top to bottom. The scale at the bottom indicates the number of kilobases. (PDF) [file pgen.1006226.s007.pdf]
