# Supplementary material for: Mek1 Down Regulates Rad51 Activity during Yeast Meiosis by Phosphorylation of Hed1
Source: PLoS Genet. 2016 Aug 2;12(8):e1006226. doi: 10.1371/journal.pgen.1006226 (PMC4970670; doi:10.1371/journal.pgen.1006226)
Supplement: S5 Fig — E5: events between two chromatids; E6: events between three chromatids; E7, events between four chromatids. Blacks indicate the region of repair. (PDF) [file pgen.1006226.s008.pdf]

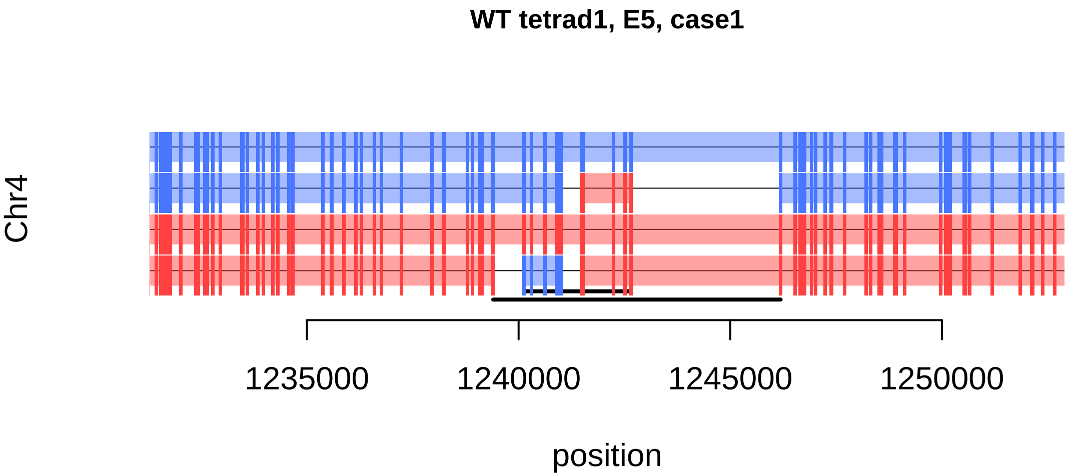
WT tetrad1, E5, case1
Chr4
1235000
1240000
1245000
1250000
position

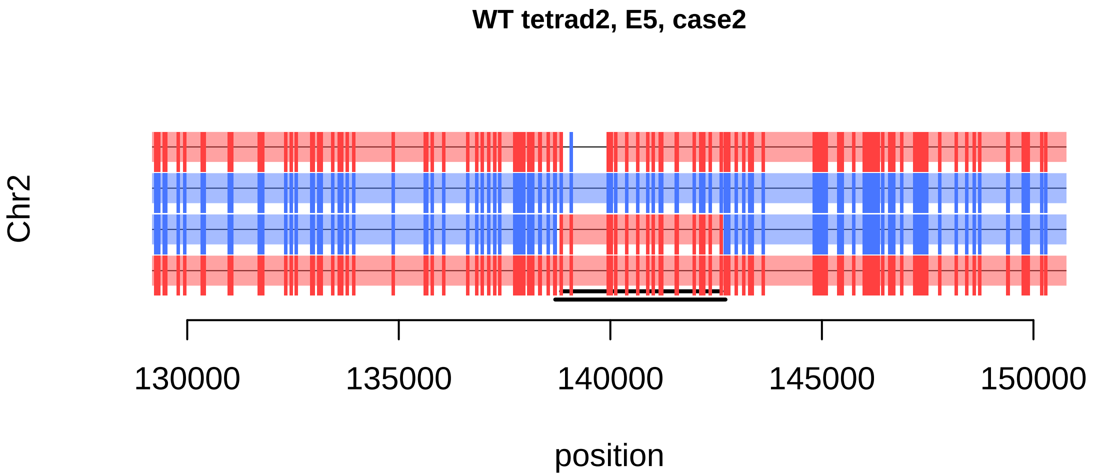
WT tetrad2, E5, case2
Chr2
130000
135000
140000
145000
150000
position

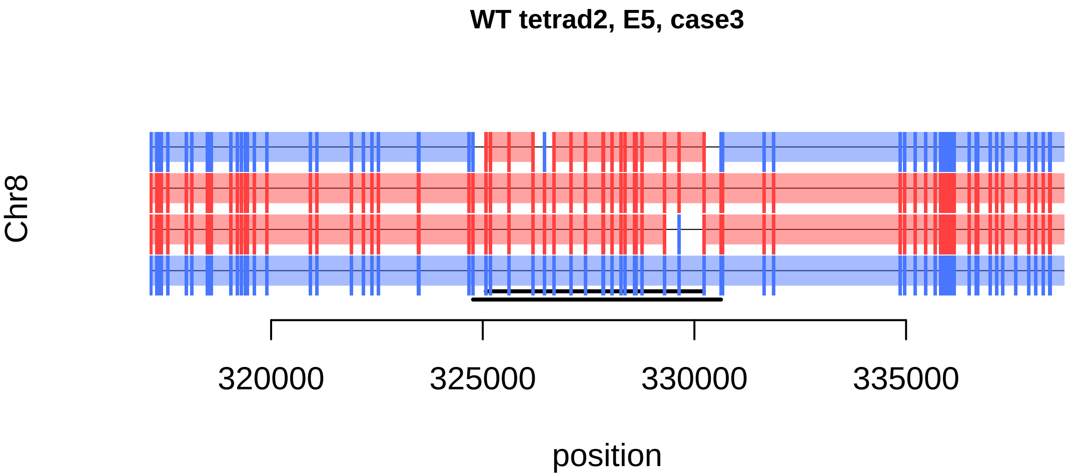

WT tetrad2, E5, case3
Chr8
320000
325000
330000
335000
position

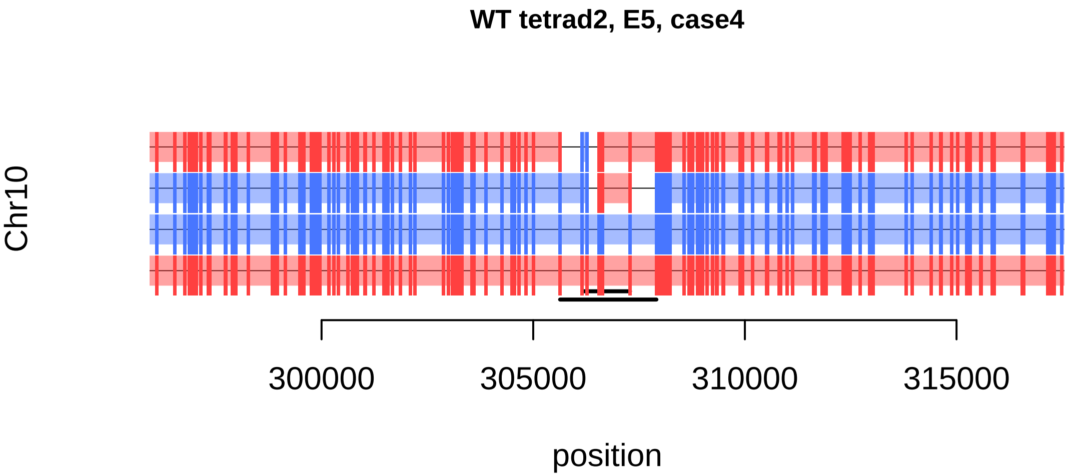

WT tetrad2, E5, case4
Chr10
300000
305000
310000
315000
position

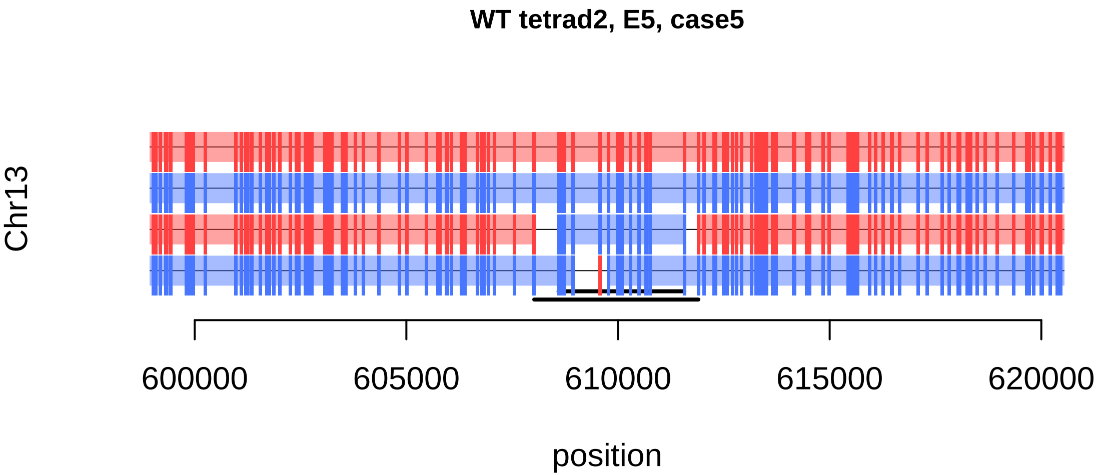
WT tetrad2, E5, case5
Chr13
600000
605000
610000
615000
620000
position

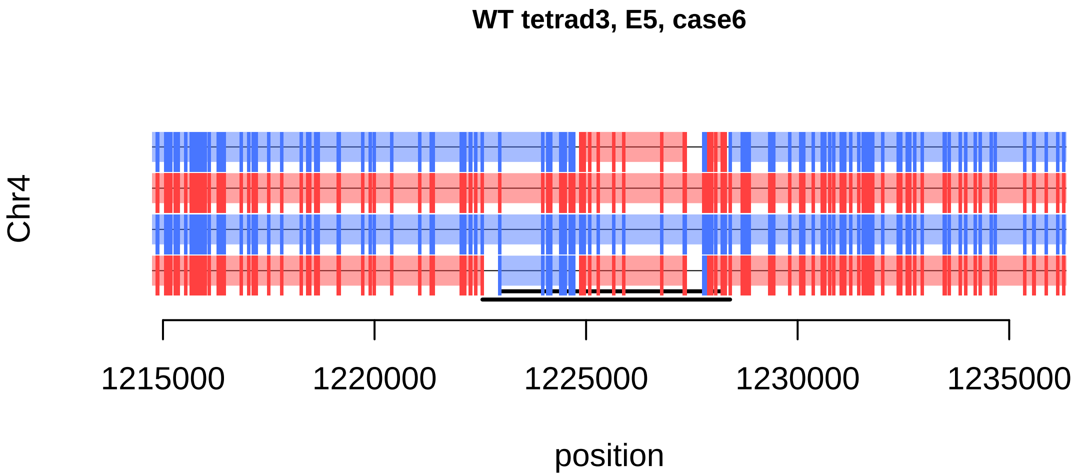
WT tetrad3, E5, case6
Chr4
1215000
1220000
1225000
1230000
1235000
position

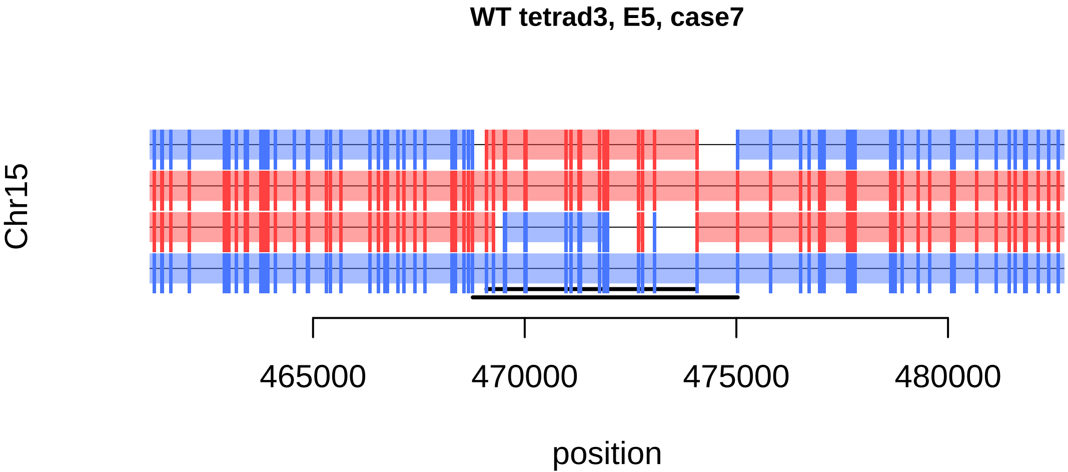

WT tetrad3, E5, case7
Chr15
465000
470000
475000
480000
position

## WT tetrad3, E5, case8

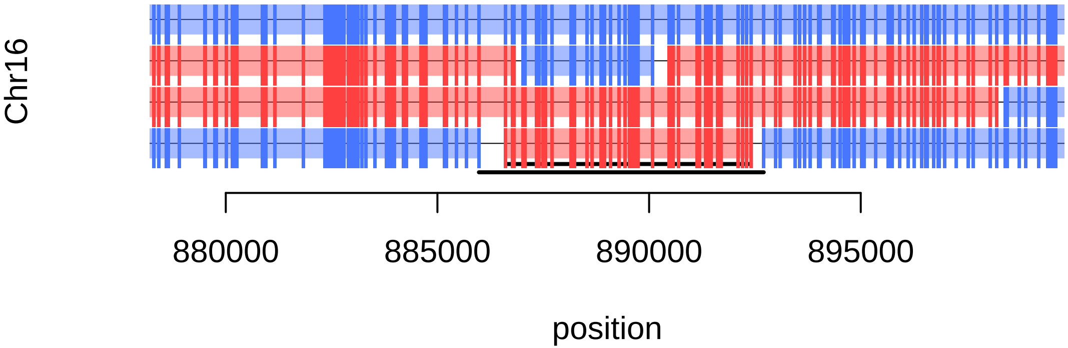

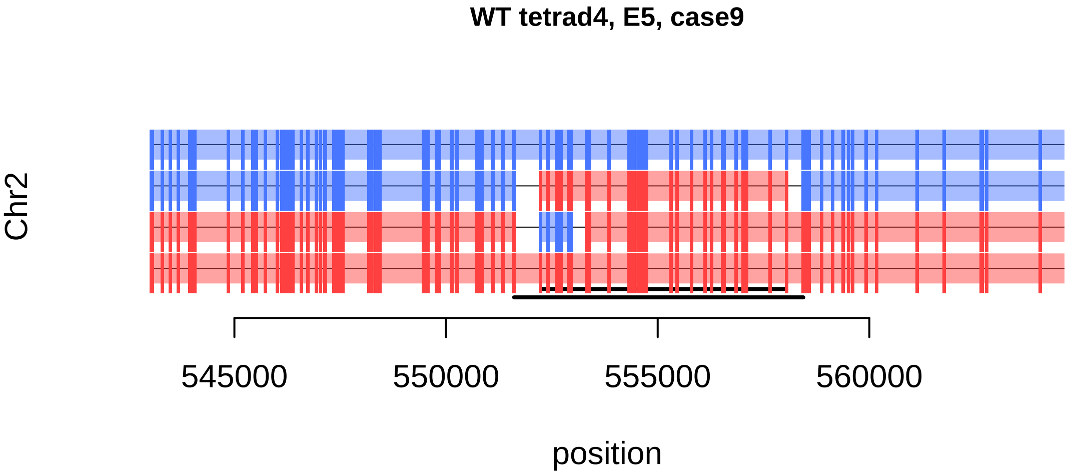
WT tetrad4, E5, case9
Chr2
545000
550000
555000
560000
position

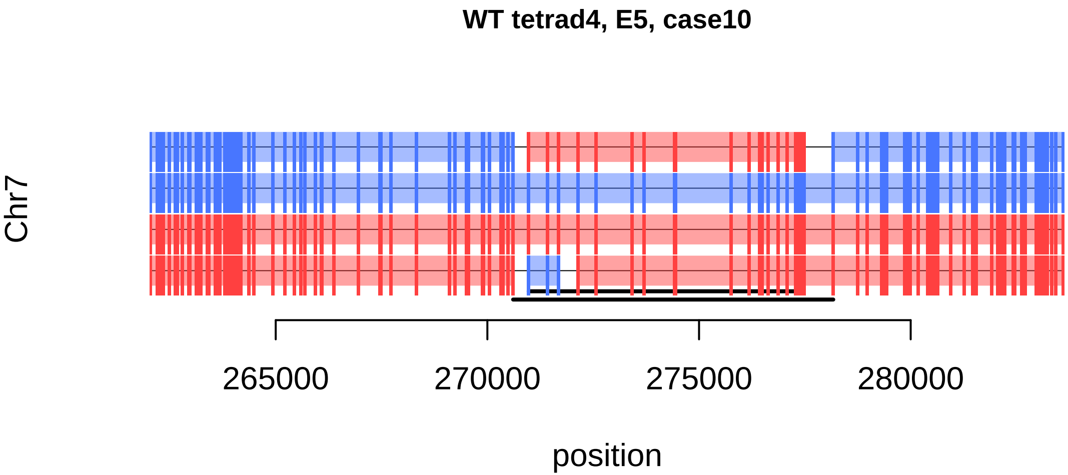
WT tetrad4, E5, case10
Chr7
265000
270000
275000
280000
position

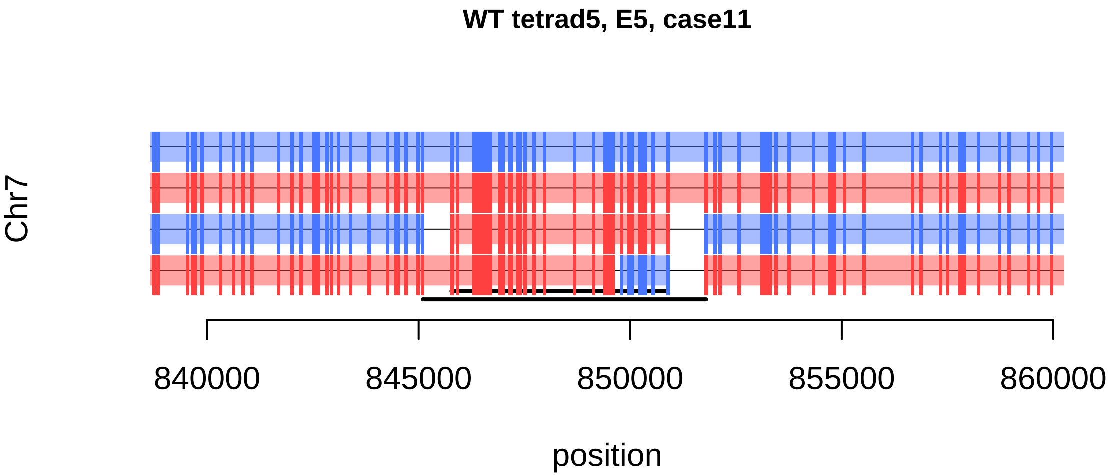

WT tetrad5, E5, case11
Chr7
840000
845000
850000
855000
860000
position

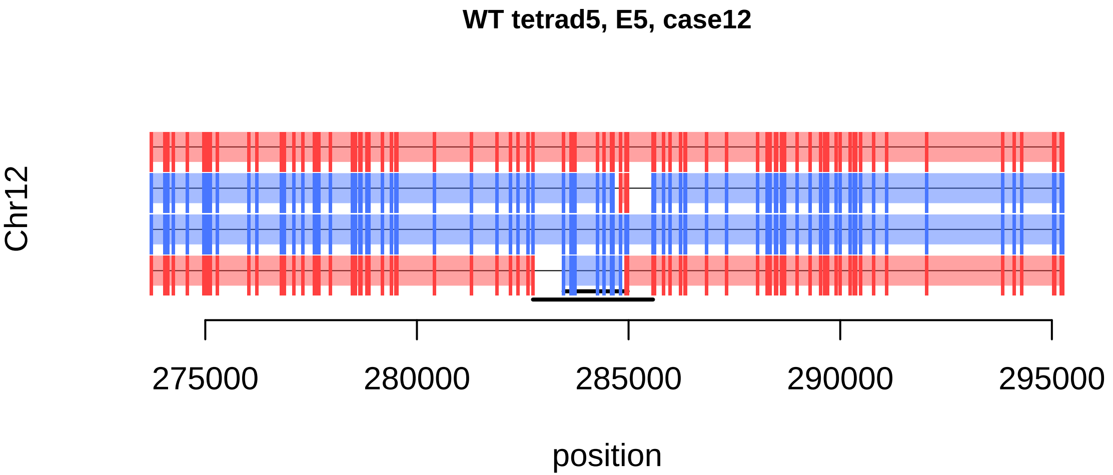
WT tetrad5, E5, case12
Chr12
275000
280000
285000
290000
295000
position

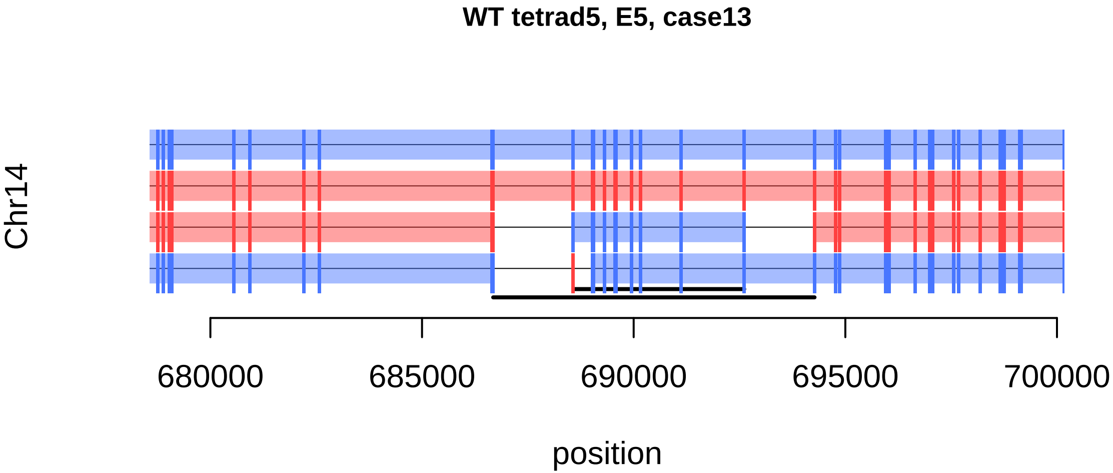
WT tetrad5, E5, case13
Chr14
680000
685000
690000
695000
700000
position

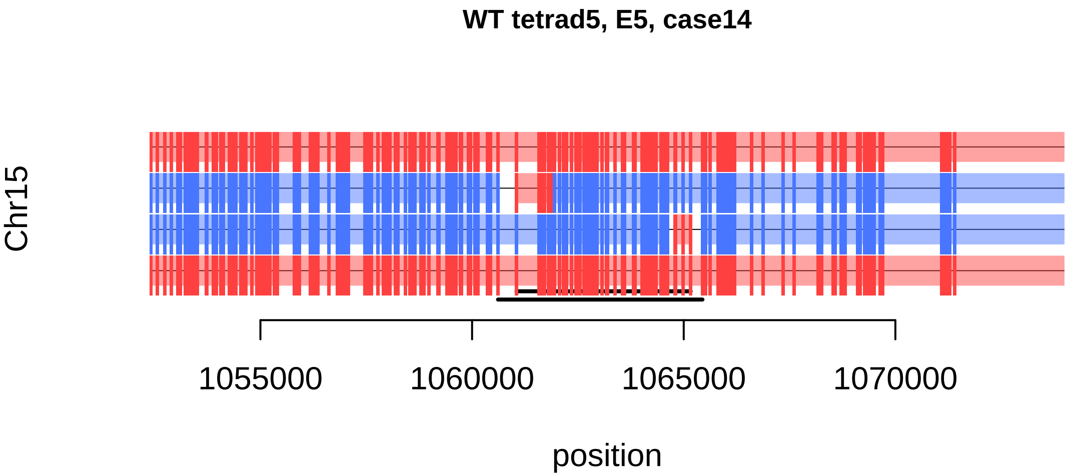

WT tetrad5, E5, case14
Chr15
1055000
1060000
1065000
1070000
position

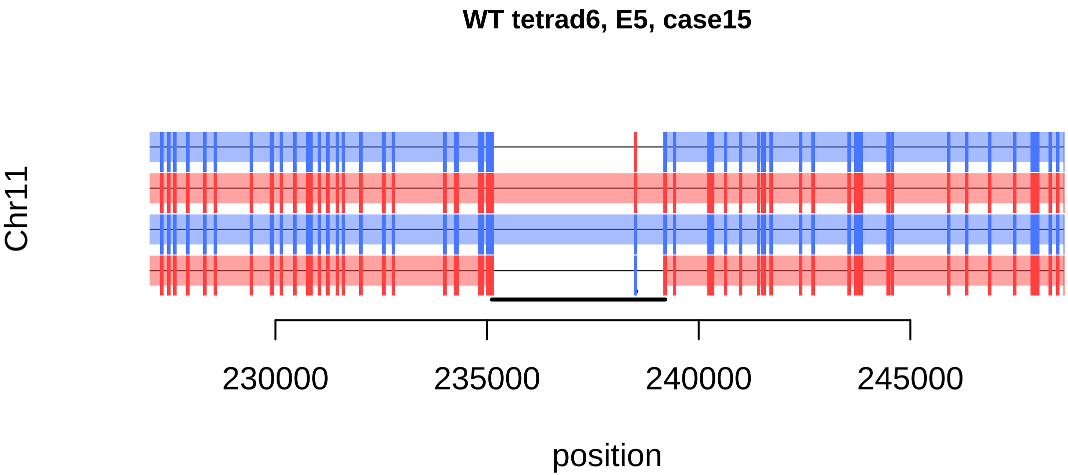
WT tetrad6, E5, case15
Chr11
230000
235000
240000
245000
position

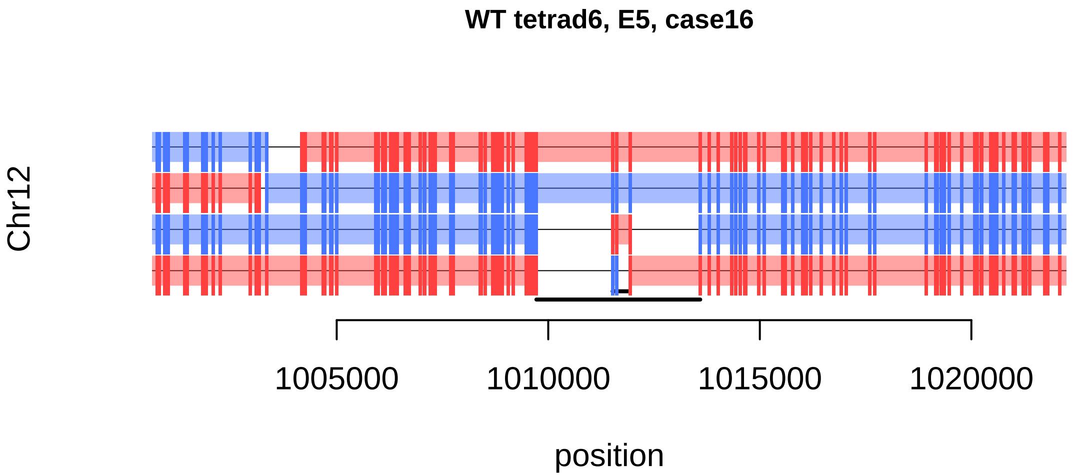
WT tetrad6, E5, case16
Chr12
1005000
1010000
1015000
1020000
position

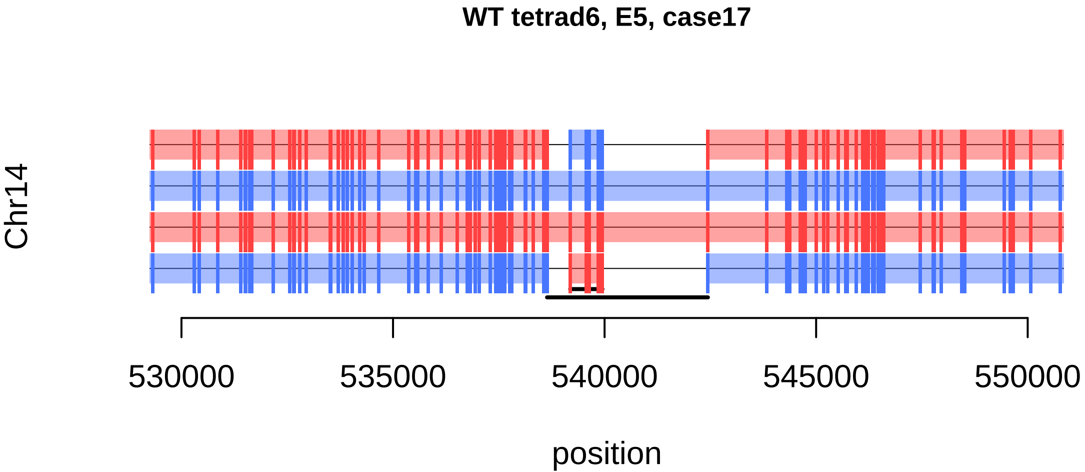

WT tetrad6, E5, case17
Chr14
530000
535000
540000
545000
550000
position

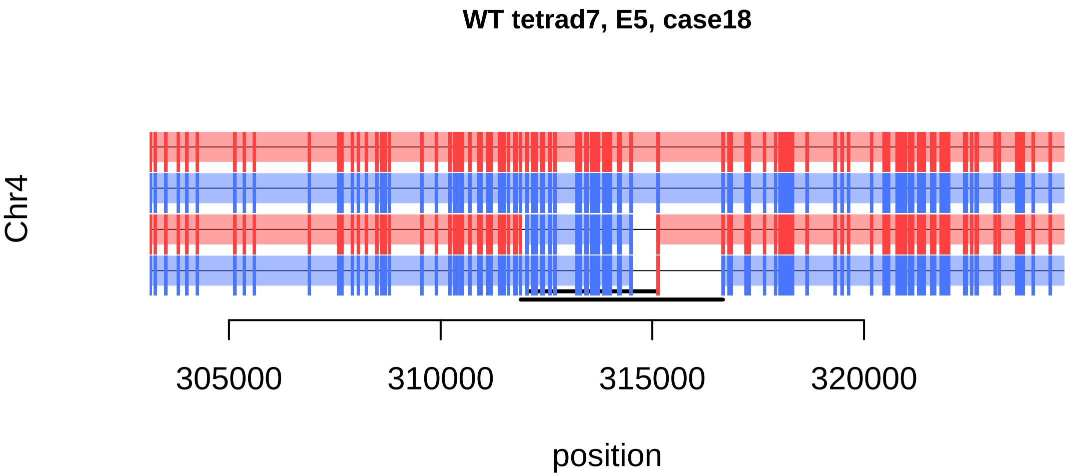
WT tetrad7, E5, case18
Chr4
305000
310000
315000
320000
position

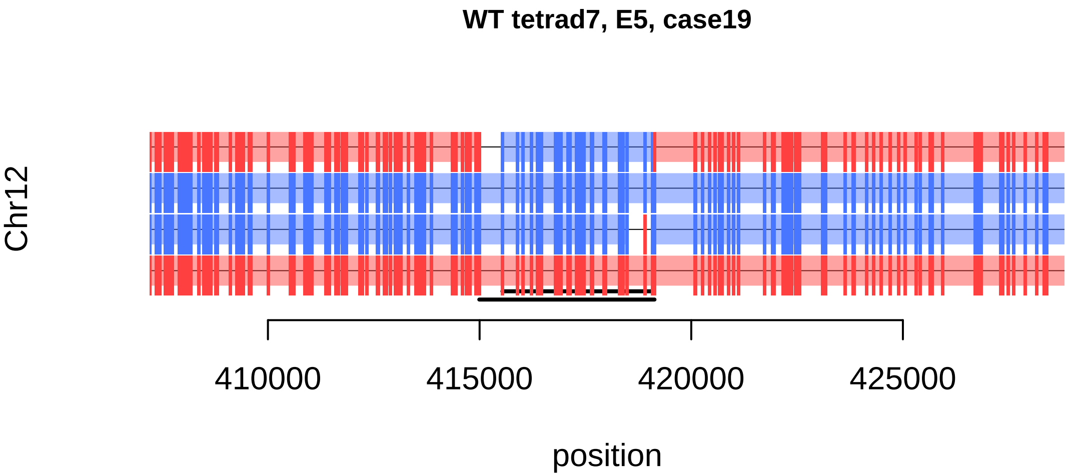
WT tetrad7, E5, case19
Chr12
410000
415000
420000
425000
position

### WT tetrad8, E5, case20

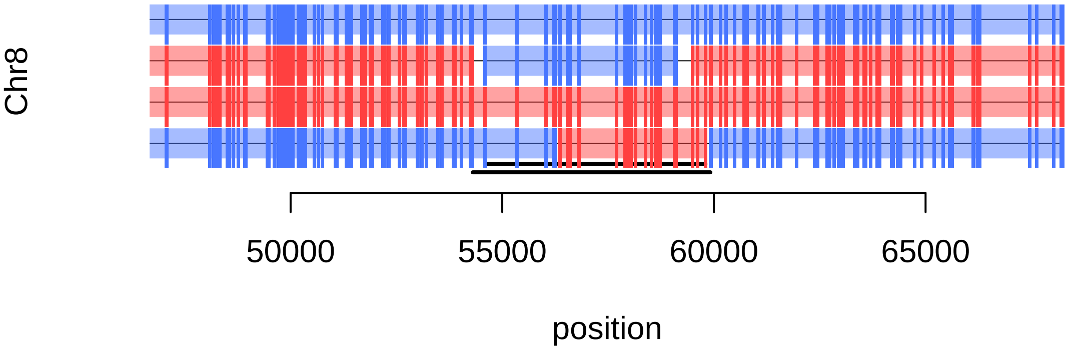

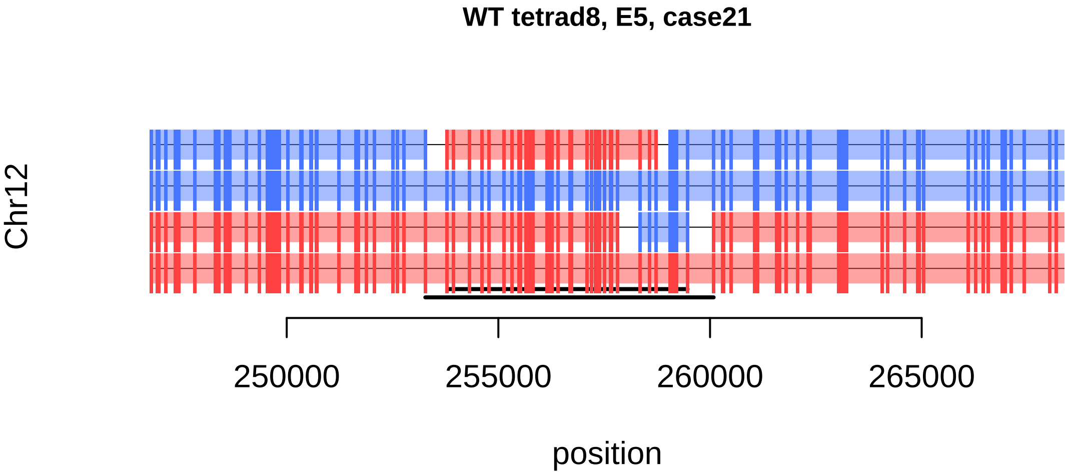

WT tetrad8, E5, case21
Chr12
250000
255000
260000
265000
position

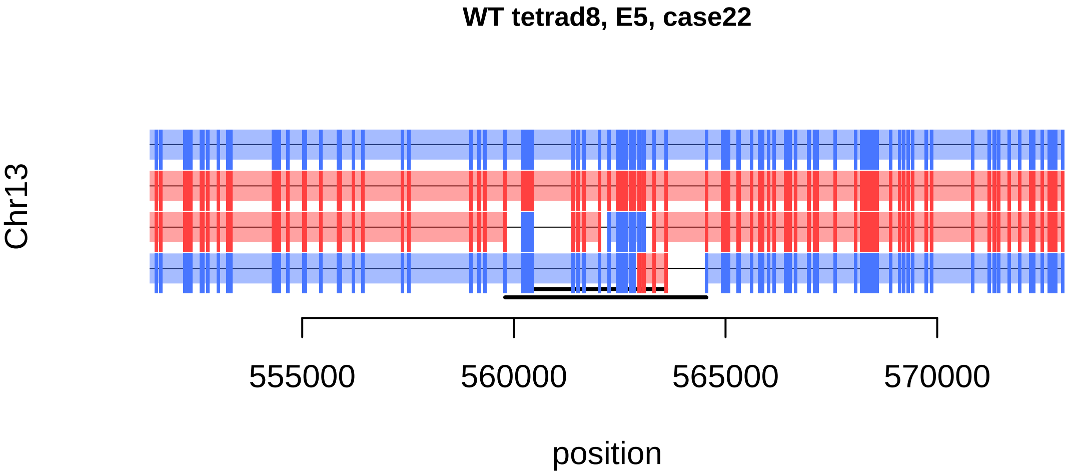

WT tetrad8, E5, case22
Chr13
555000
560000
565000
570000
position

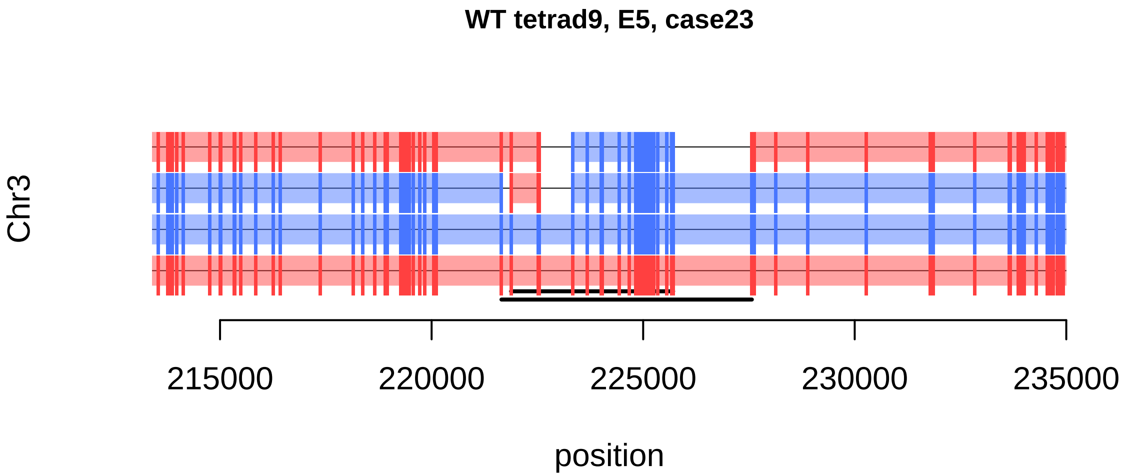
WT tetrad9, E5, case23
Chr3
215000
220000
225000
230000
235000
position

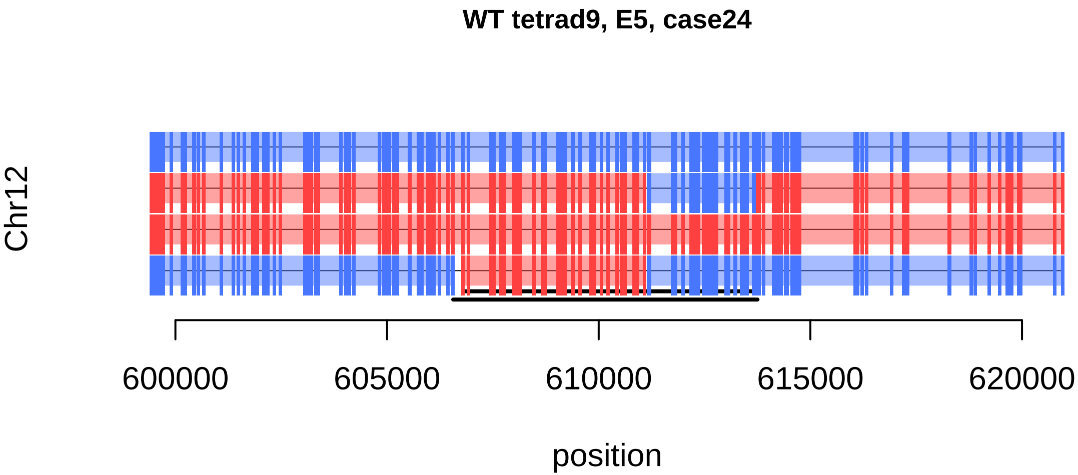

WT tetrad9, E5, case24
Chr12
600000
605000
610000
615000
620000
position

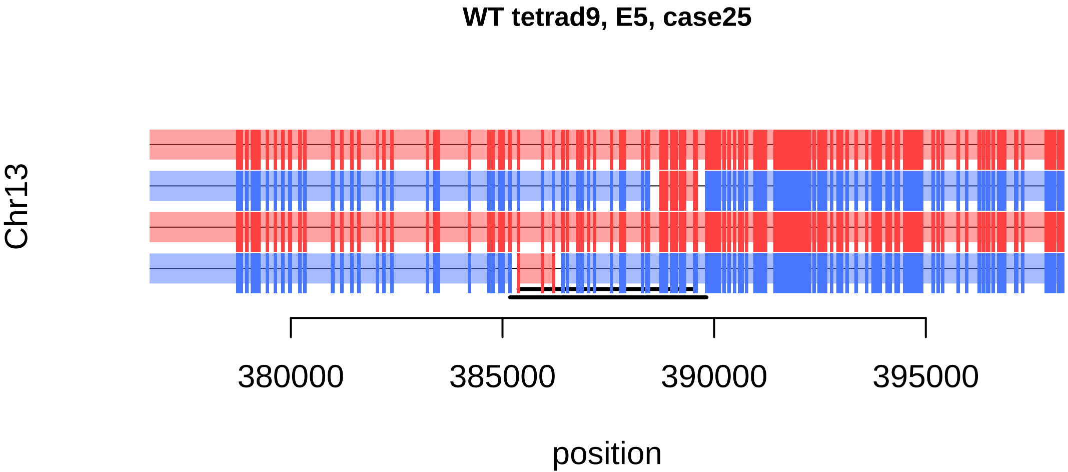

WT tetrad9, E5, case25
Chr13
380000
385000
390000
395000
position

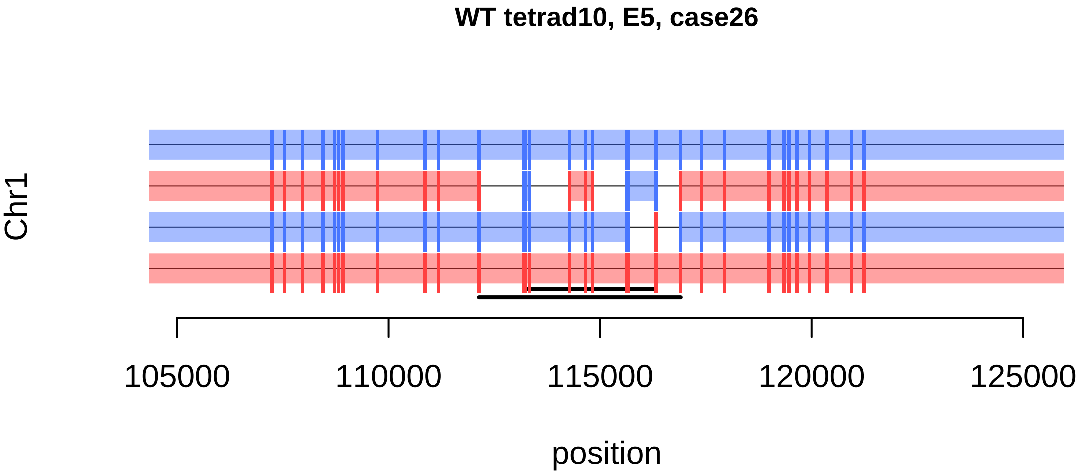
WT tetrad10, E5, case26
Chr1
105000
110000
115000
120000
125000
position

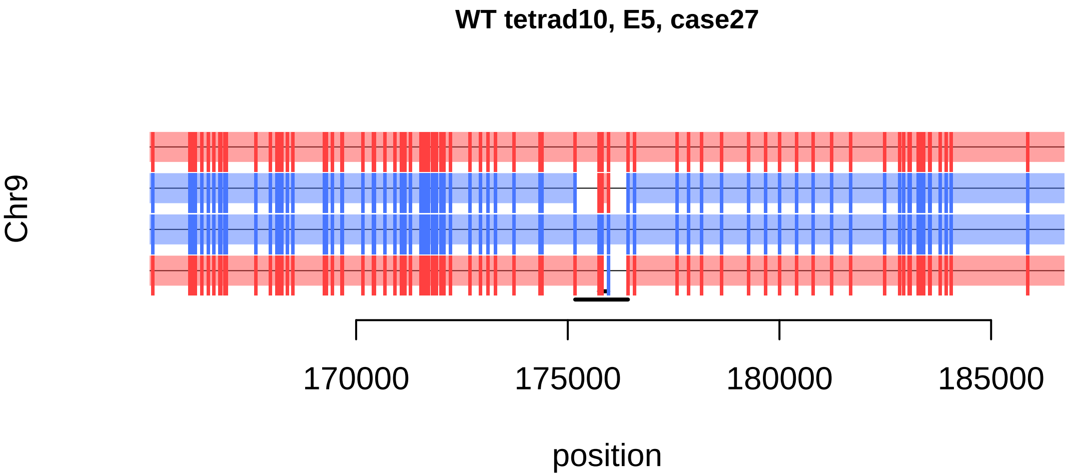
WT tetrad10, E5, case27
Chr9
170000
175000
180000
185000
position

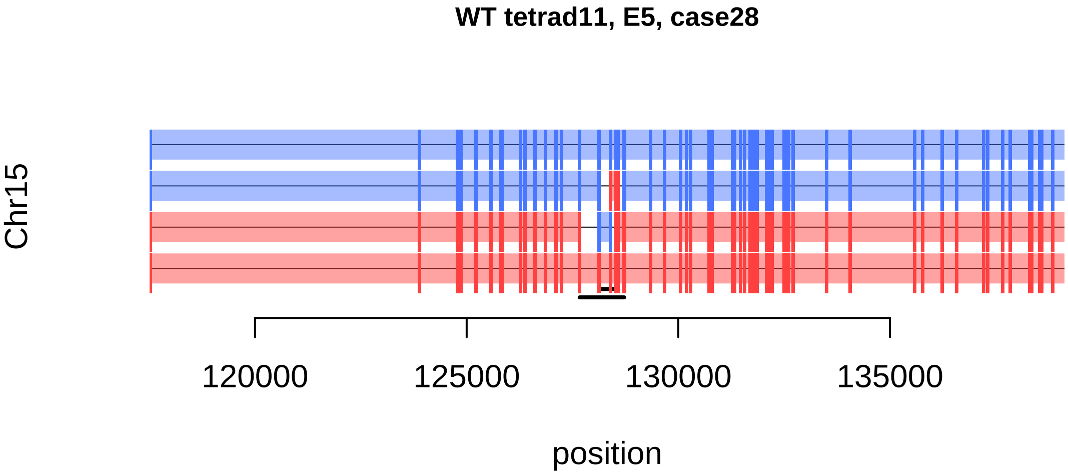

WT tetrad11, E5, case28
Chr15
120000
125000
130000
135000
position

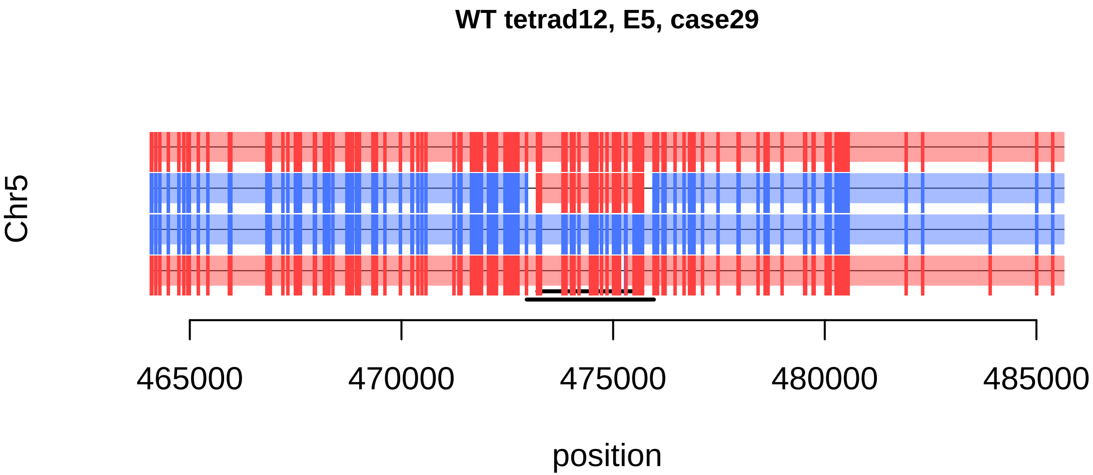
WT tetrad12, E5, case29
Chr5
465000
470000
475000
480000
485000
position

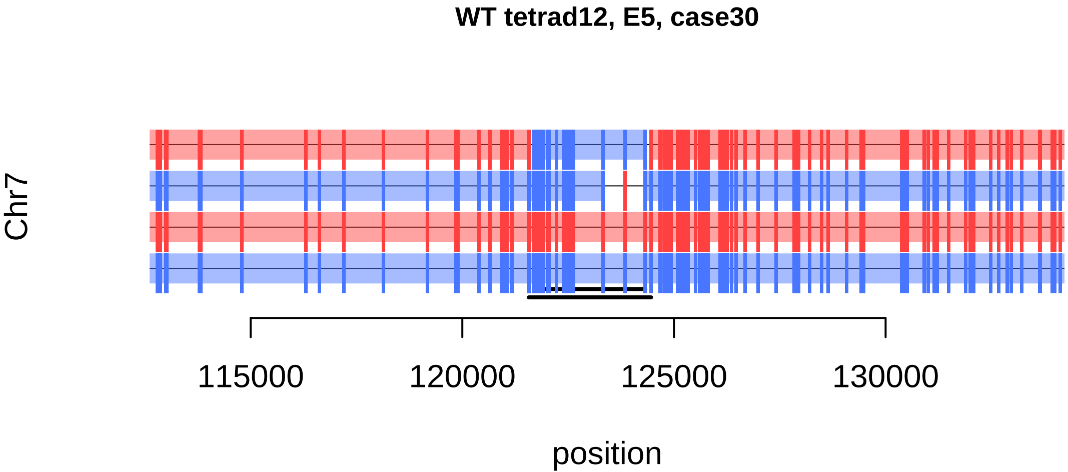
WT tetrad12, E5, case30
Chr7
115000
120000
125000
130000
position

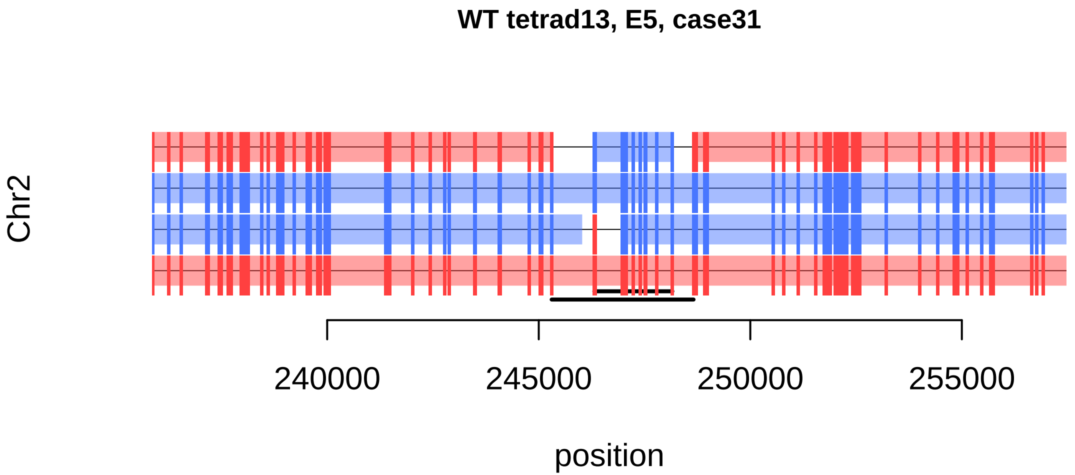
WT tetrad13, E5, case31
Chr2
240000
245000
250000
255000
position

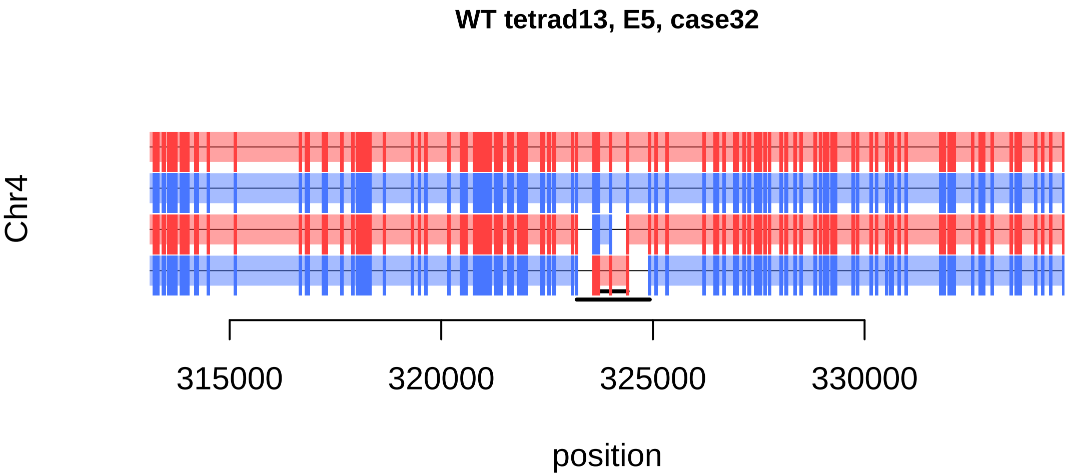
WT tetrad13, E5, case32
Chr4
315000
320000
325000
330000
position

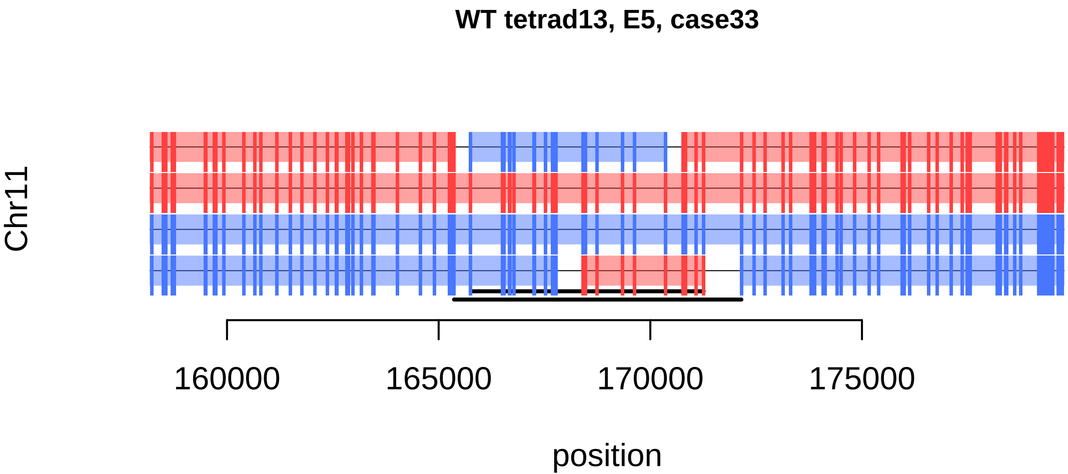
WT tetrad13, E5, case33
Chr11
160000
165000
170000
175000
position

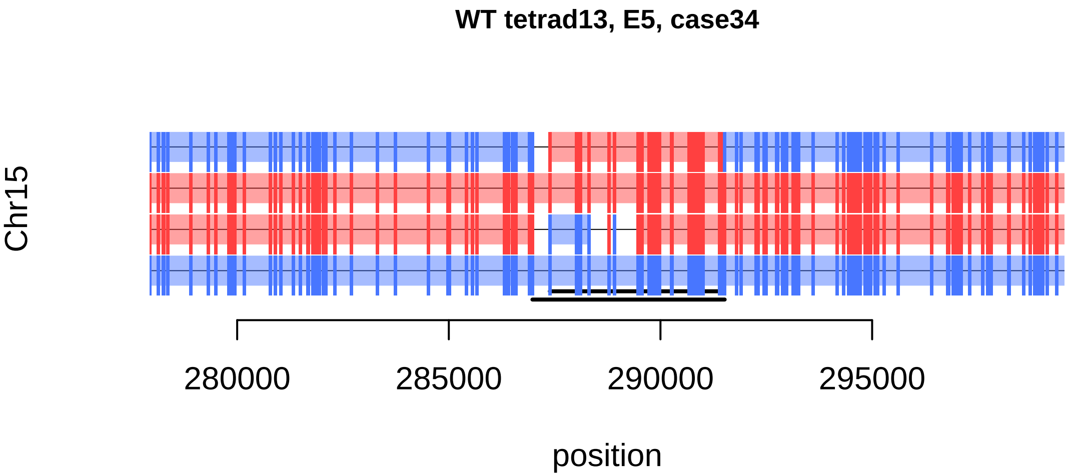
WT tetrad13, E5, case34
Chr15
280000
285000
290000
295000
position

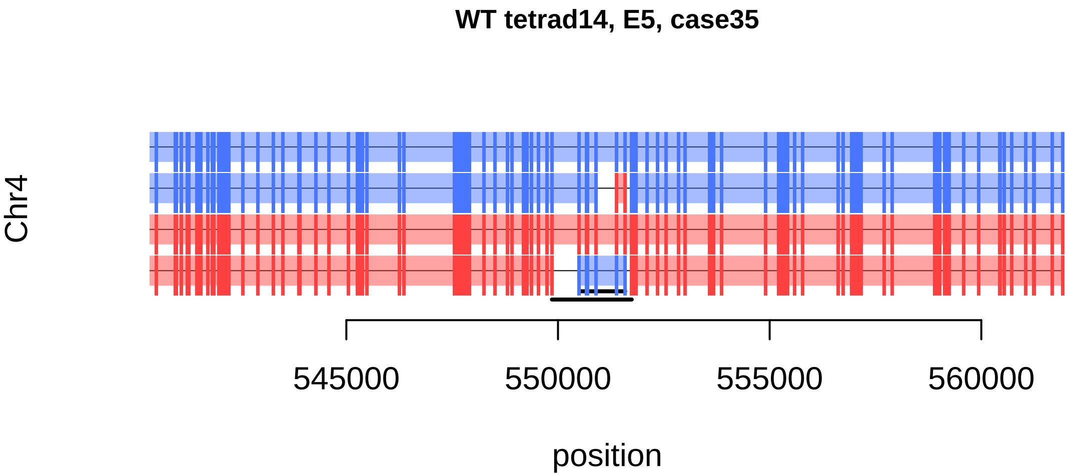
WT tetrad14, E5, case35
Chr4
545000
550000
555000
560000
position

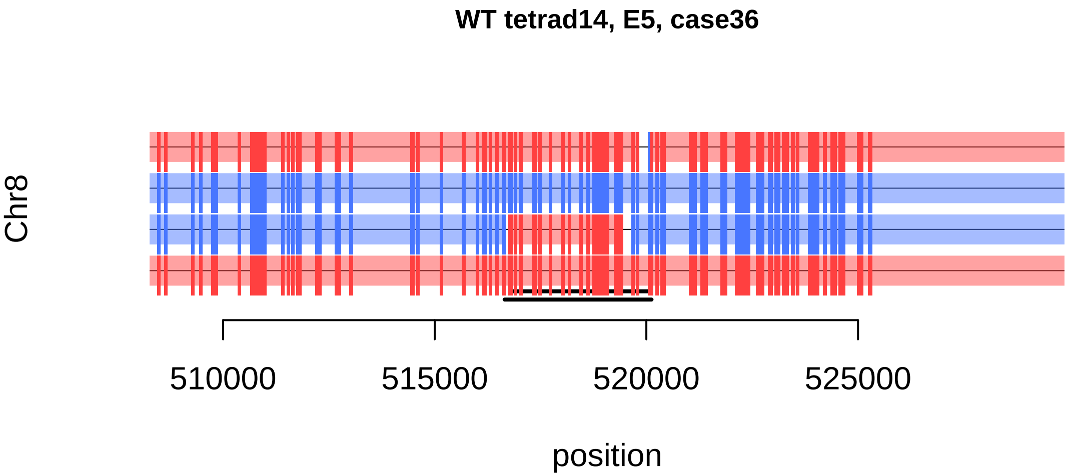
WT tetrad14, E5, case36
Chr8
510000
515000
520000
525000
position

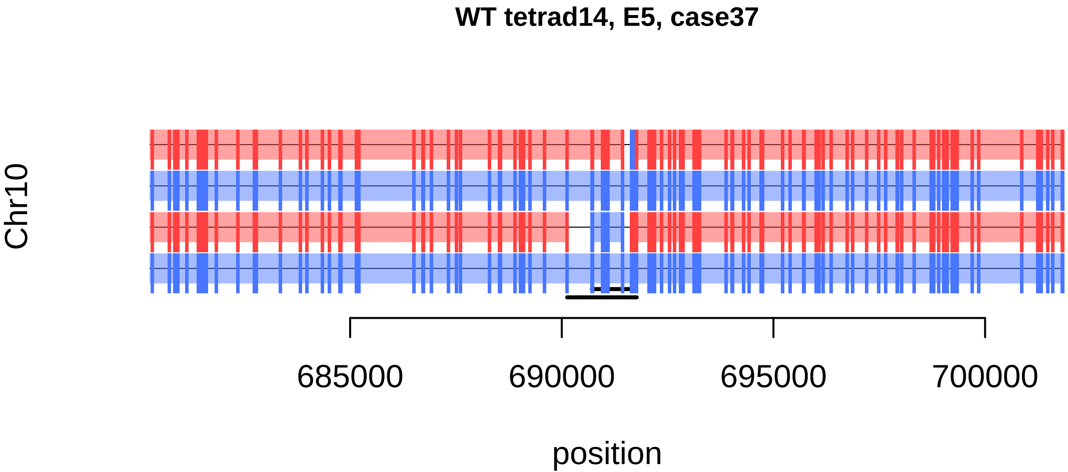
WT tetrad14, E5, case37
Chr10
685000
690000
695000
700000
position

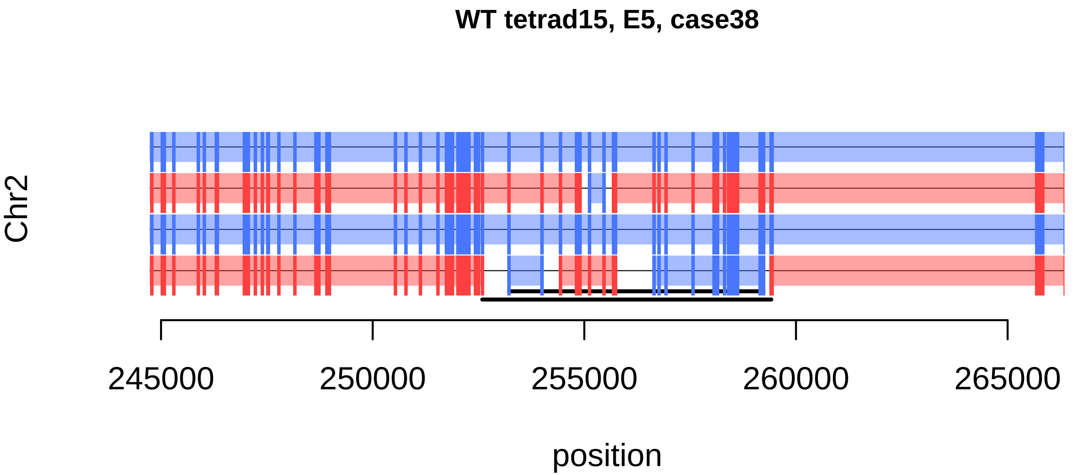
WT tetrad15, E5, case38
Chr2
245000
250000
255000
260000
265000
position

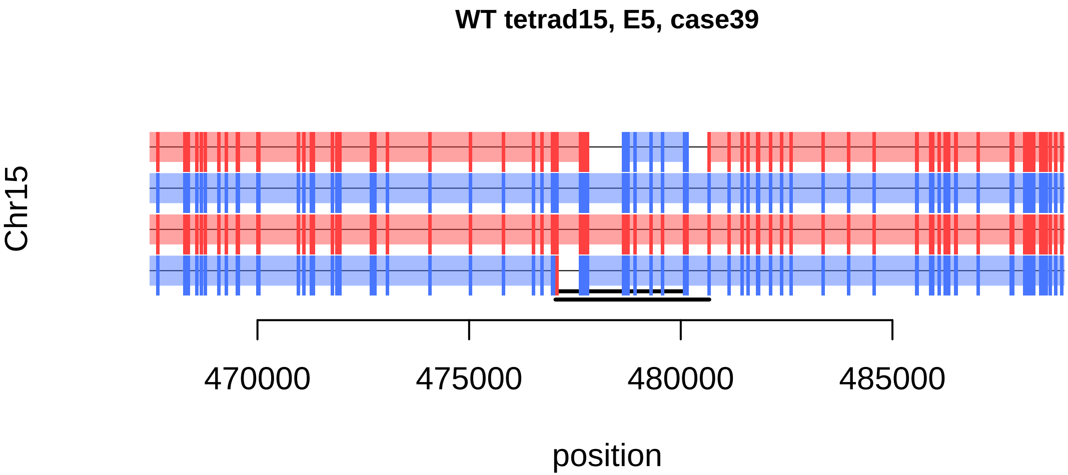
WT tetrad15, E5, case39
Chr15
470000
475000
480000
485000
position

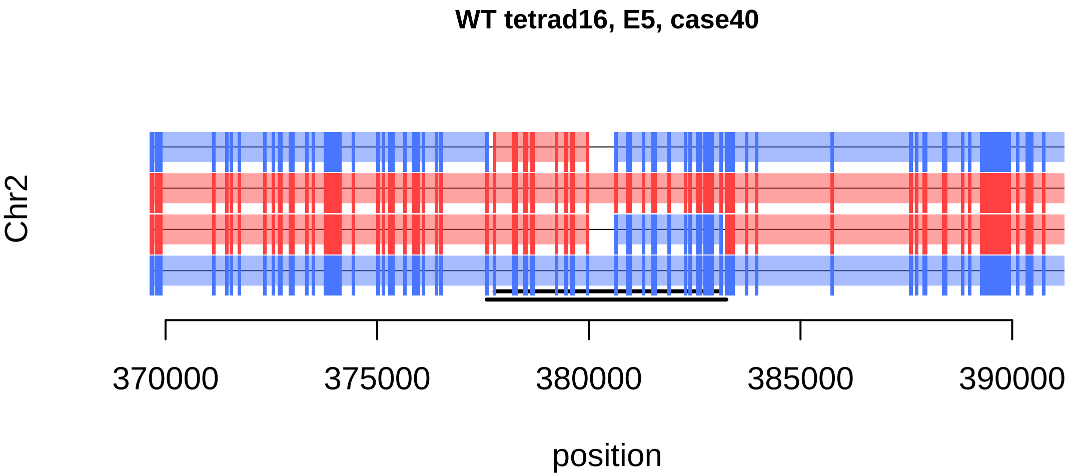
WT tetrad16, E5, case40
Chr2
370000
375000
380000
385000
390000
position

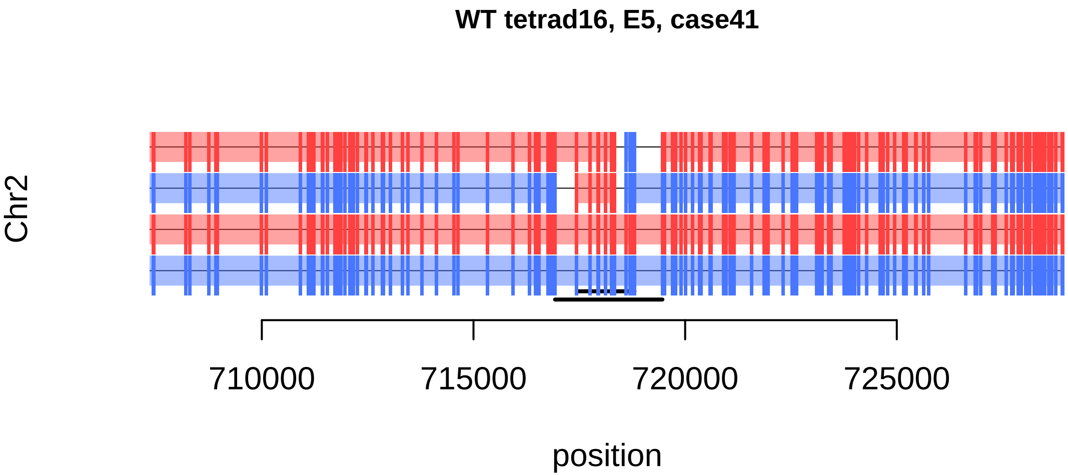
WT tetrad16, E5, case41
Chr2
710000
715000
720000
725000
position

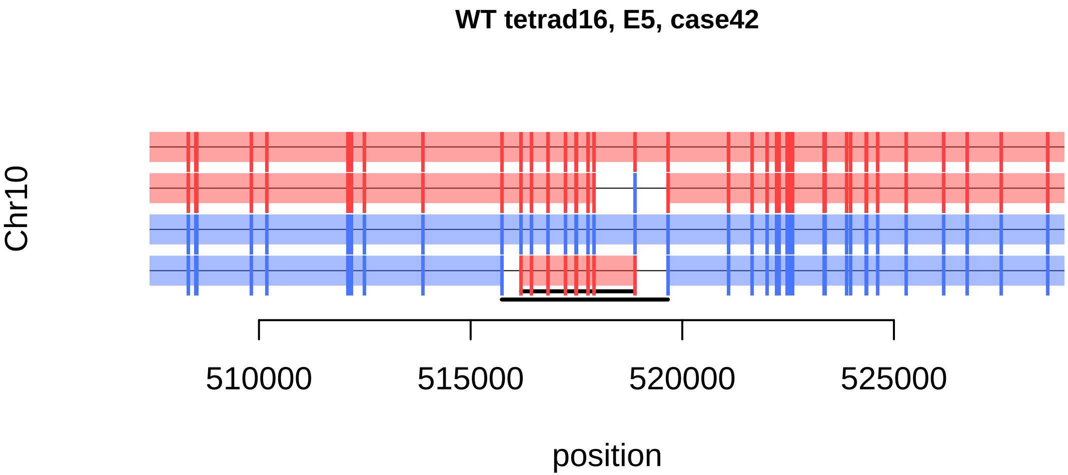

WT tetrad16, E5, case42
Chr10
510000
515000
520000
525000
position

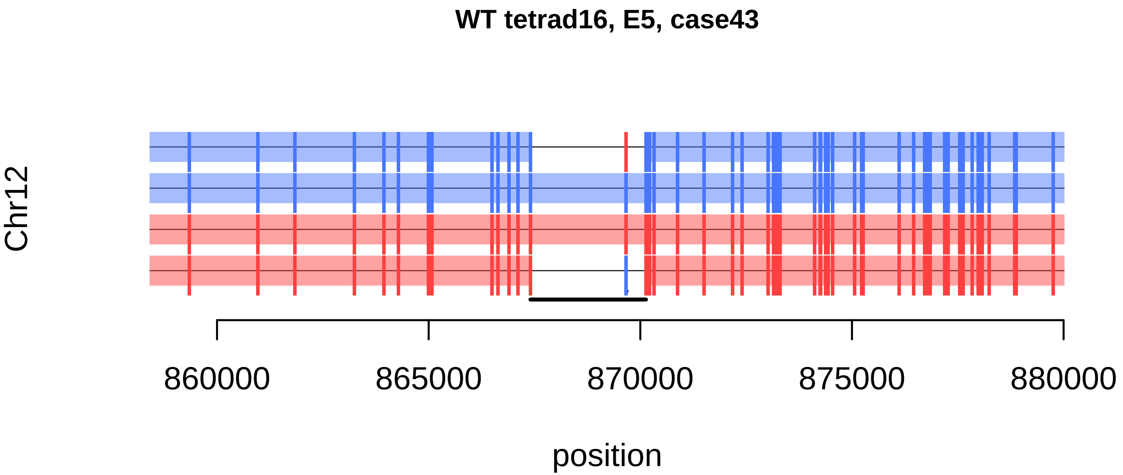
WT tetrad16, E5, case43
Chr12
860000
865000
870000
875000
880000
position

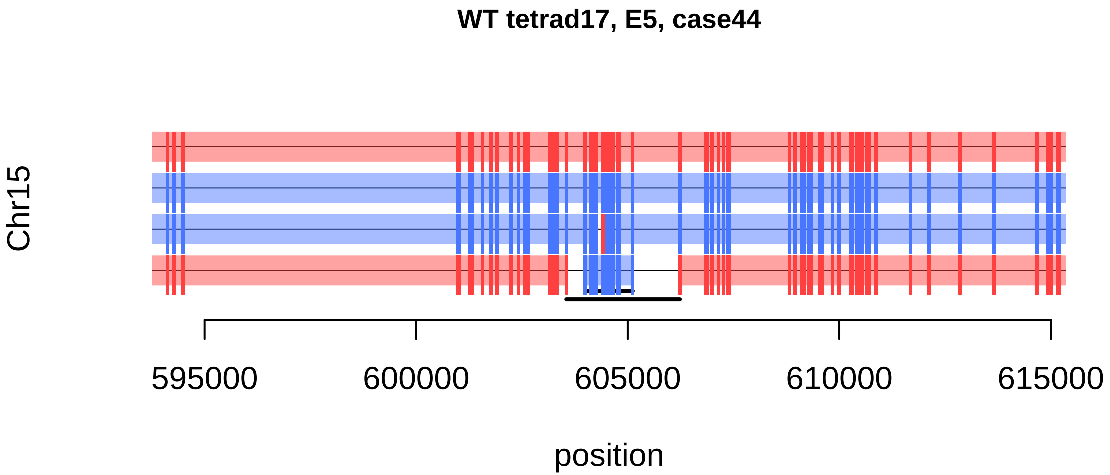

WT tetrad17, E5, case44
Chr15
595000
600000
605000
610000
615000
position

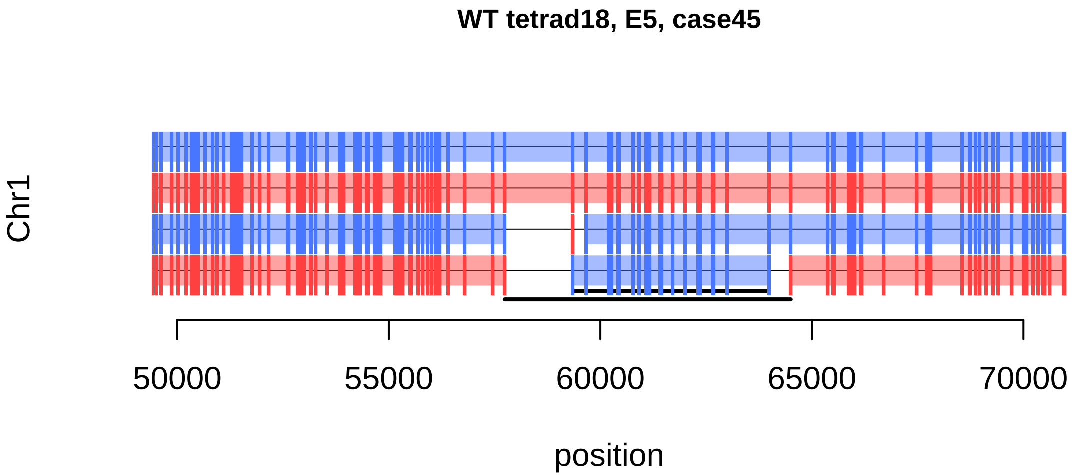

WT tetrad18, E5, case45
Chr1
50000
55000
60000
65000
70000
position

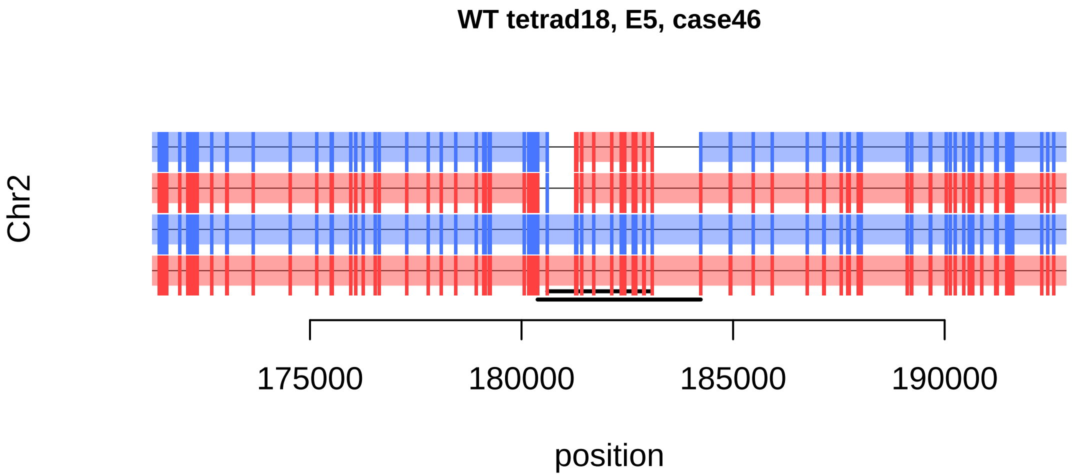

WT tetrad18, E5, case46
Chr2
175000
180000
185000
190000
position

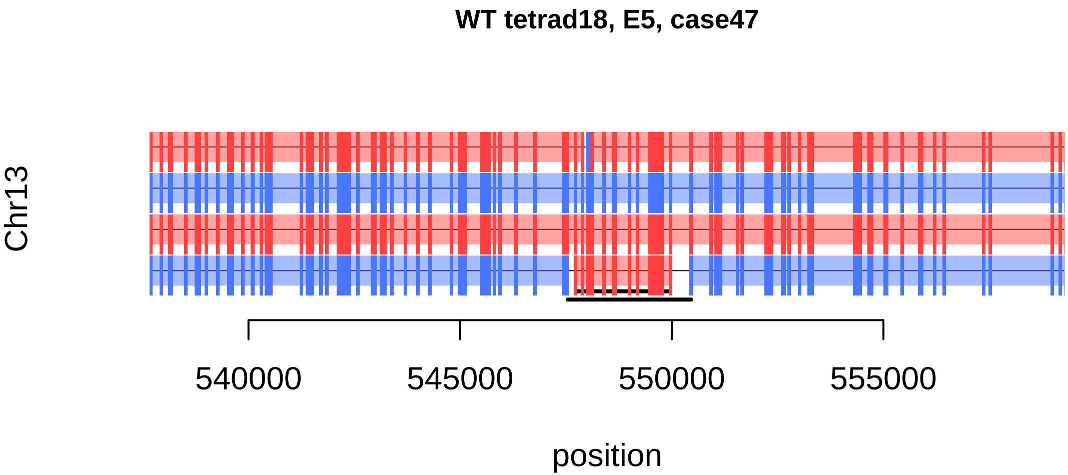
WT tetrad18, E5, case47
Chr13
540000
545000
550000
555000
position

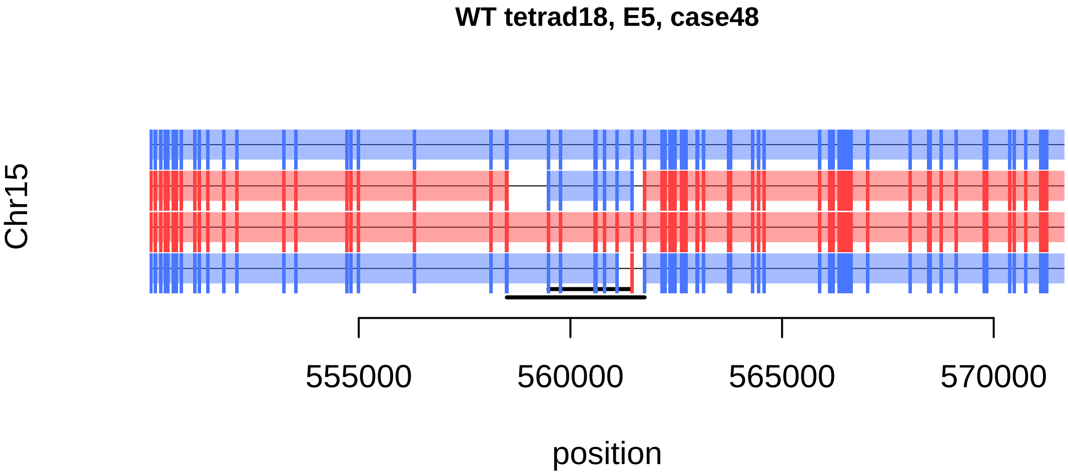
WT tetrad18, E5, case48
Chr15
555000
560000
565000
570000
position

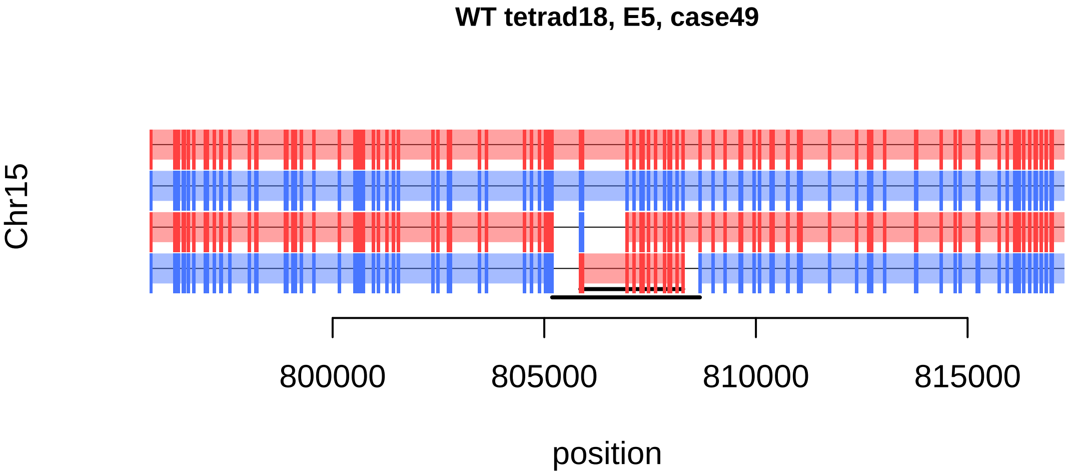
WT tetrad18, E5, case49
Chr15
800000
805000
810000
815000
position

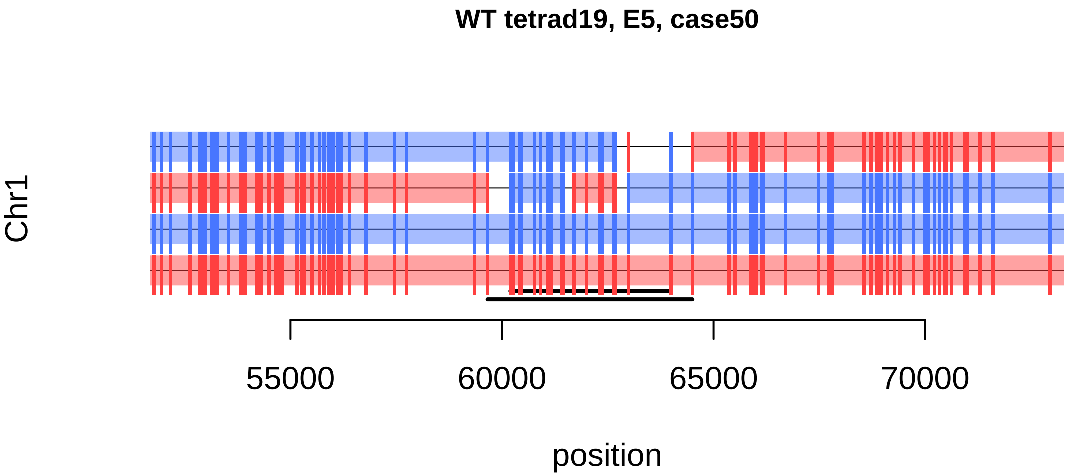

WT tetrad19, E5, case50
Chr1
55000
60000
65000
70000
position

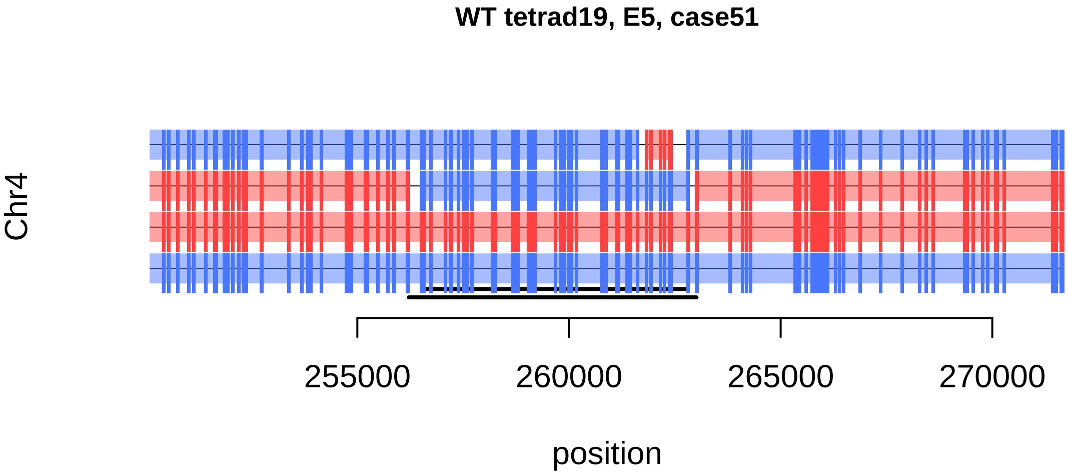
WT tetrad19, E5, case51
Chr4
255000
260000
265000
270000
position

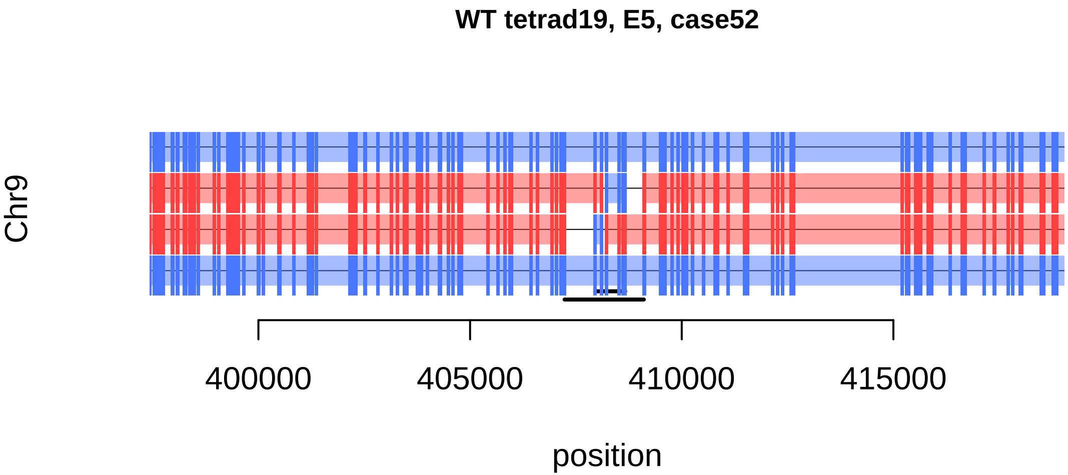
WT tetrad19, E5, case52
Chr9
400000
405000
410000
415000
position

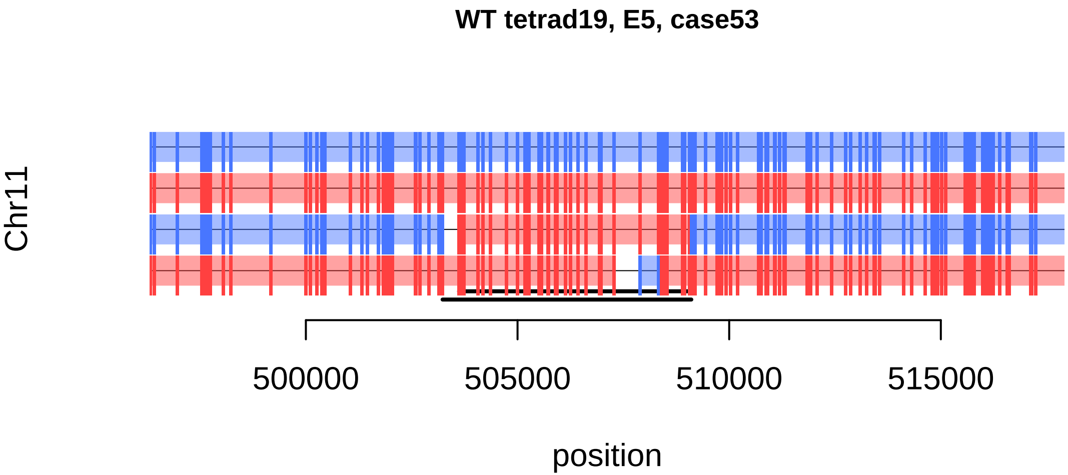

WT tetrad19, E5, case53
Chr11
500000
505000
510000
515000
position

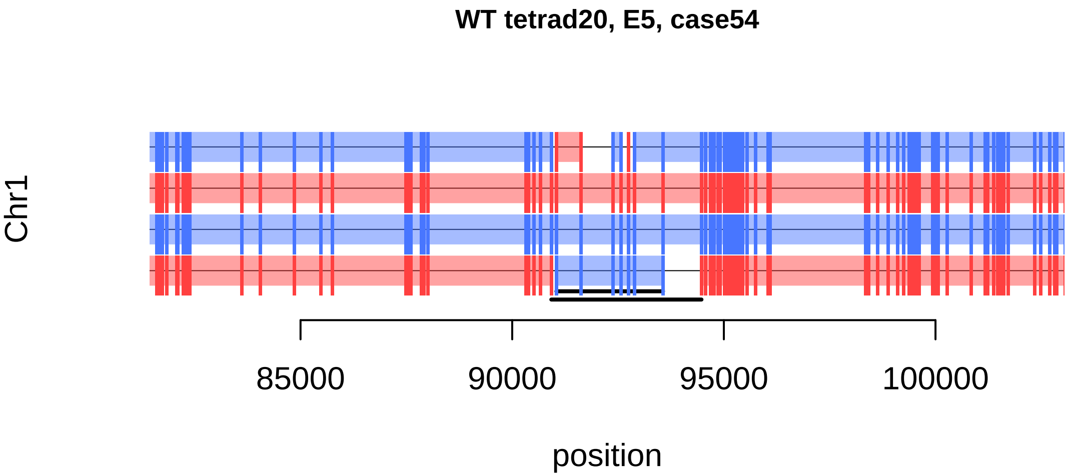

WT tetrad20, E5, case54
Chr1
85000
90000
95000
100000
position

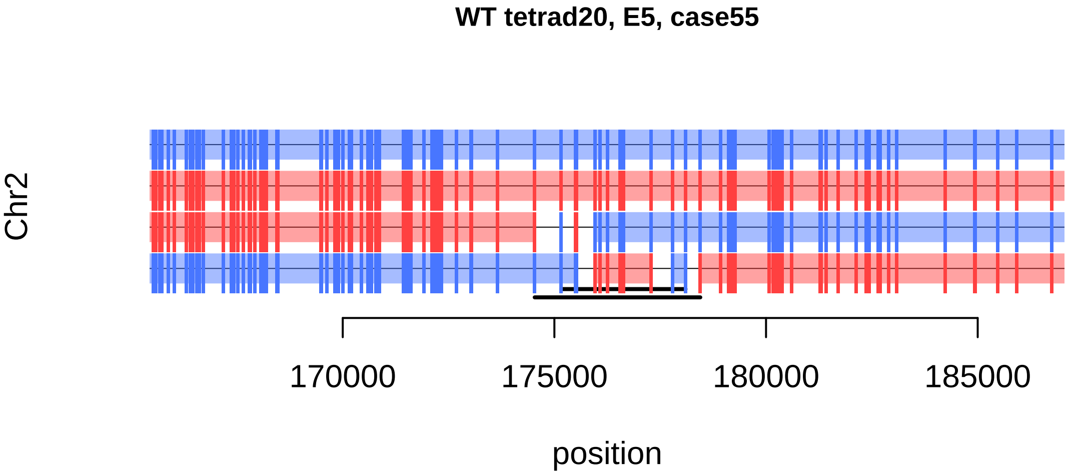
WT tetrad20, E5, case55
Chr2
170000
175000
180000
185000
position

**WT tetrad20, E5, case56**

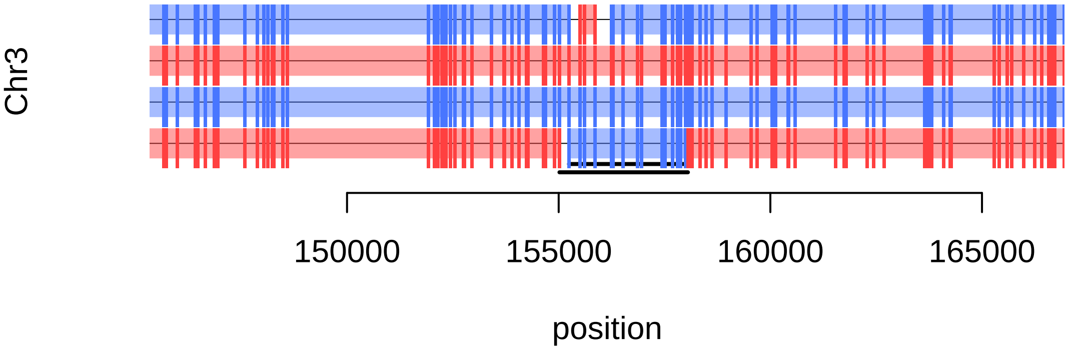

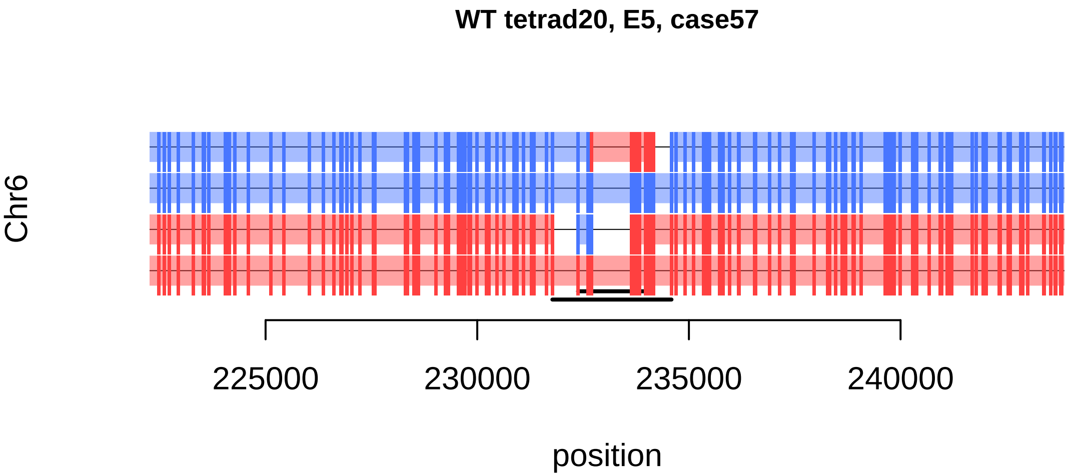

WT tetrad20, E5, case57
Chr6
225000
230000
235000
240000
position

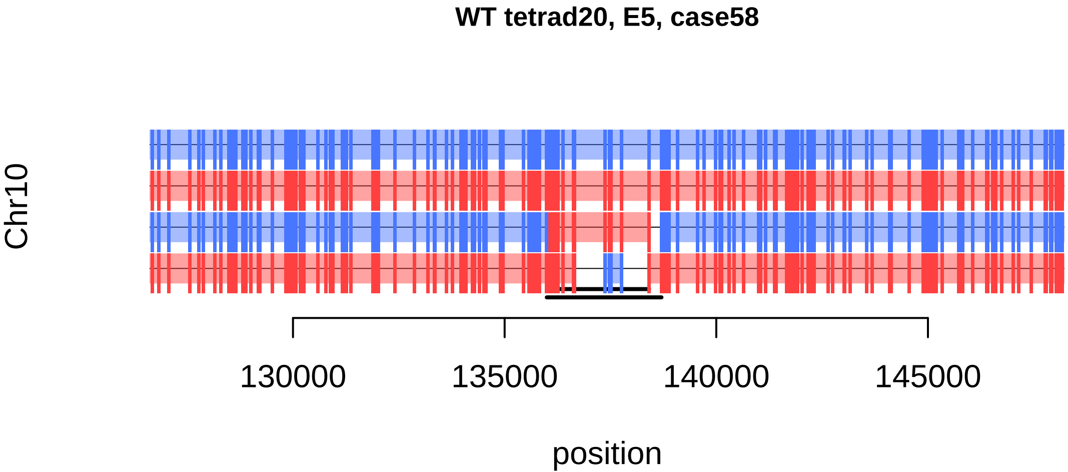
WT tetrad20, E5, case58
Chr10
130000
135000
140000
145000
position

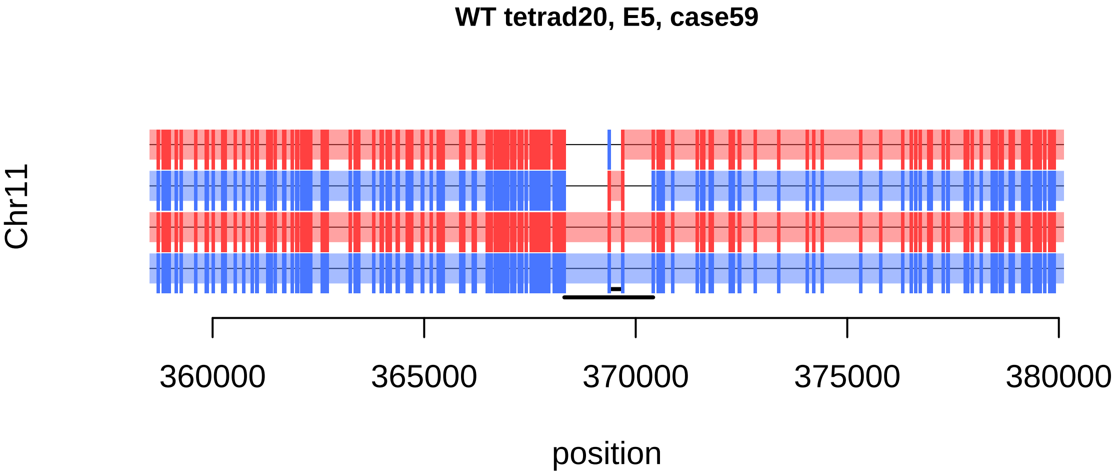
WT tetrad20, E5, case59
Chr11
360000
365000
370000
375000
380000
position

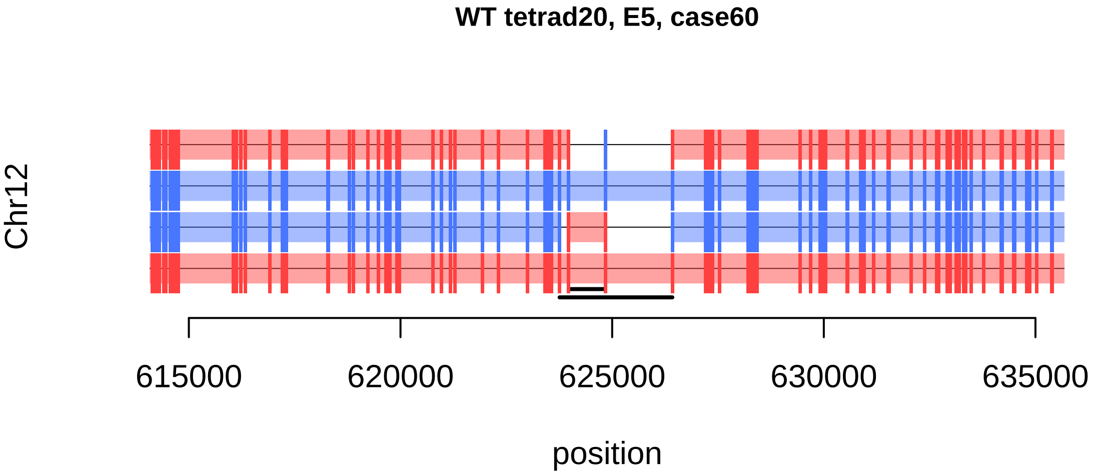
WT tetrad20, E5, case60
Chr12
615000
620000
625000
630000
635000
position

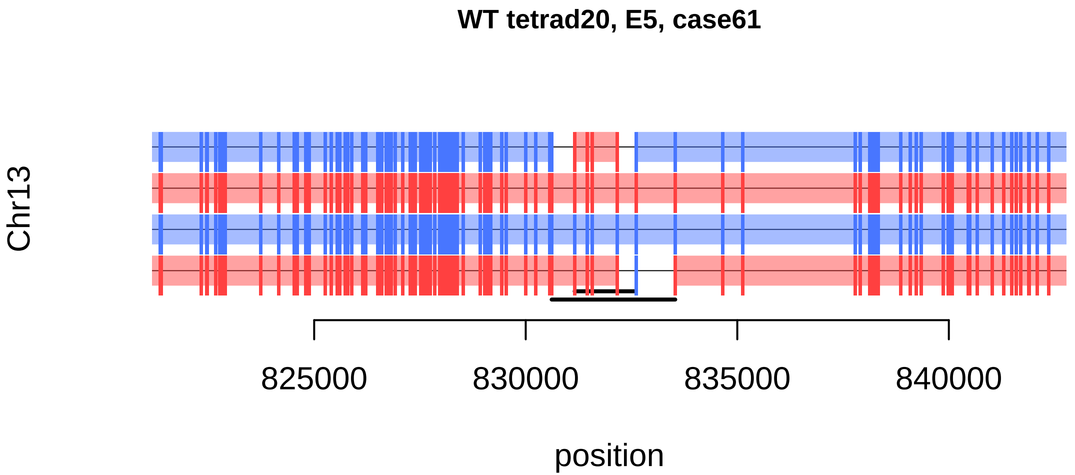
WT tetrad20, E5, case61
Chr13
825000
830000
835000
840000
position

WT tetrad20, E5, case62

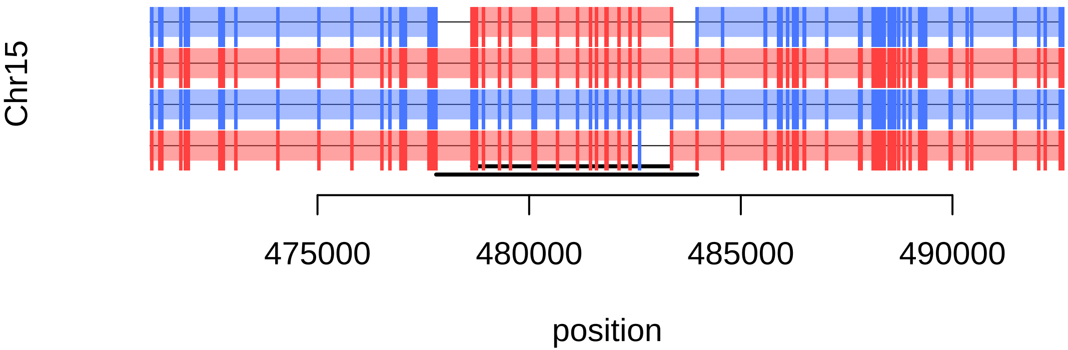

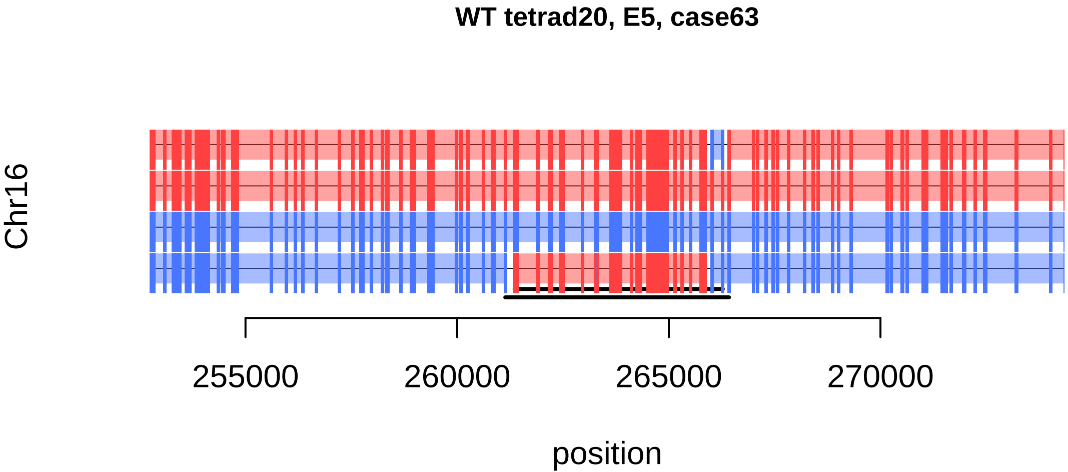
WT tetrad20, E5, case63
Chr16
255000
260000
265000
270000
position

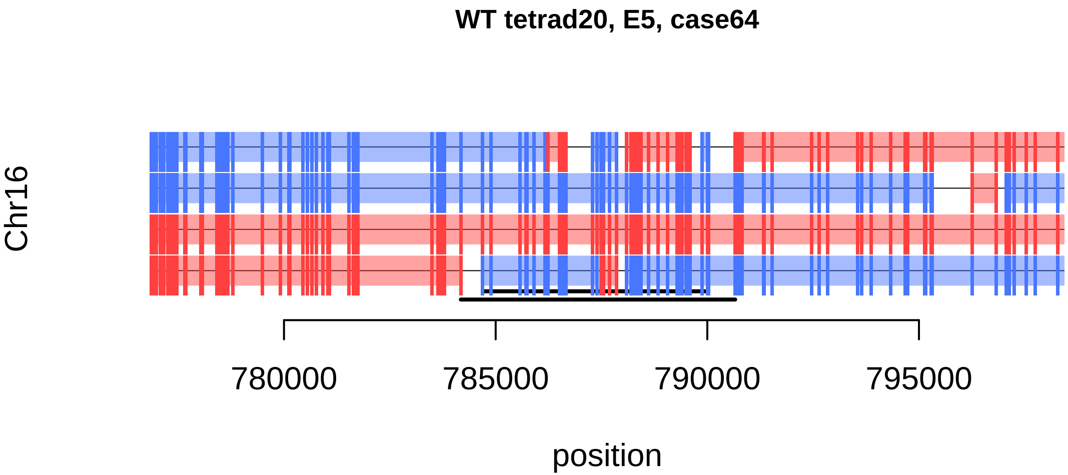

WT tetrad20, E5, case64
Chr16
780000
785000
790000
795000
position

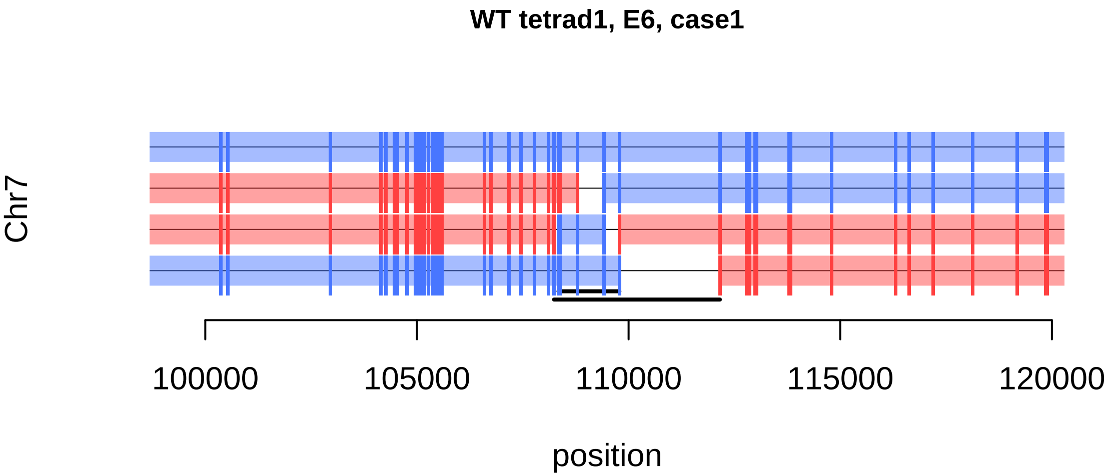

WT tetrad1, E6, case1
Chr7
100000
105000
110000
115000
120000
position

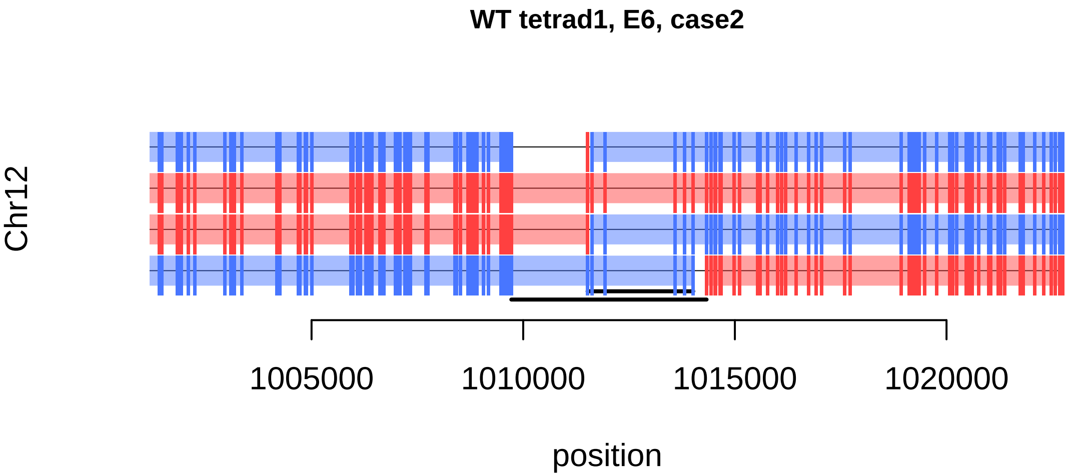

WT tetrad1, E6, case2
Chr12
1005000
1010000
1015000
1020000
position

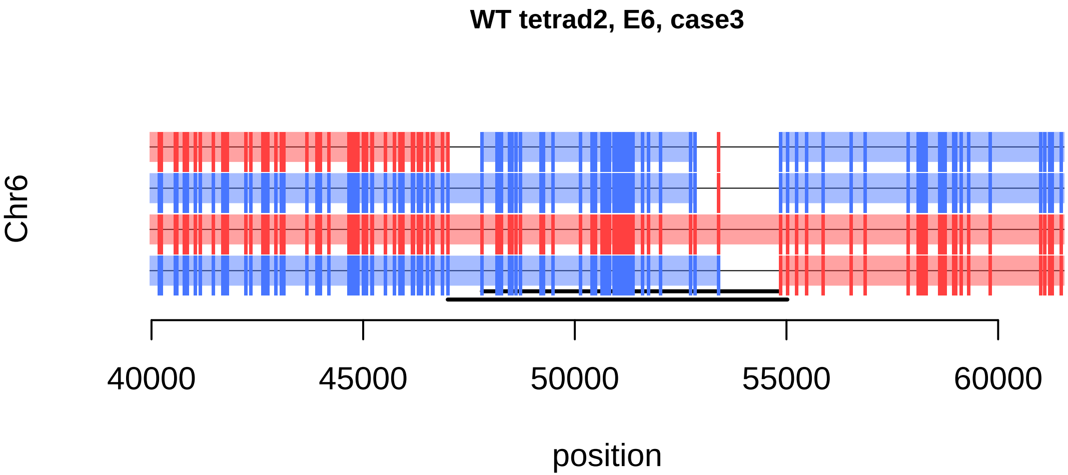
WT tetrad2, E6, case3
Chr6
40000
45000
50000
55000
60000
position

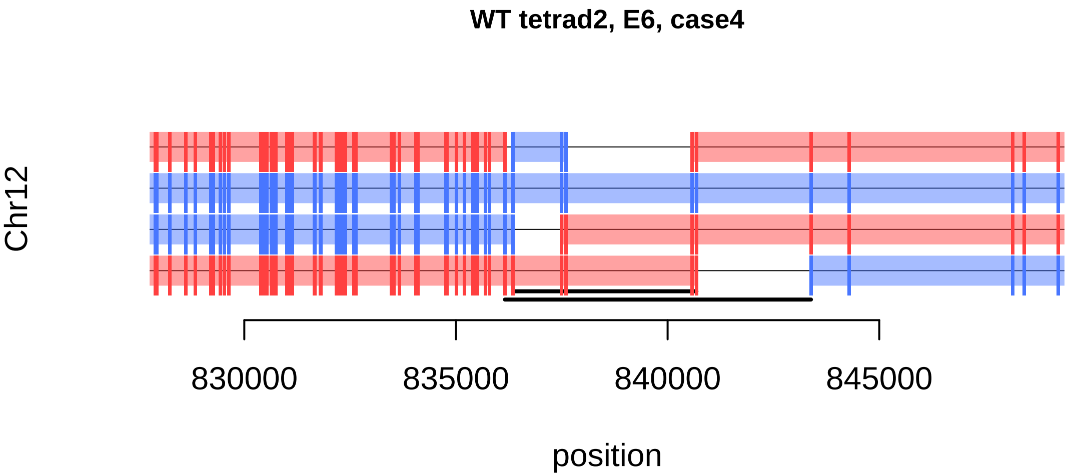

WT tetrad2, E6, case4
Chr12
830000
835000
840000
845000
position

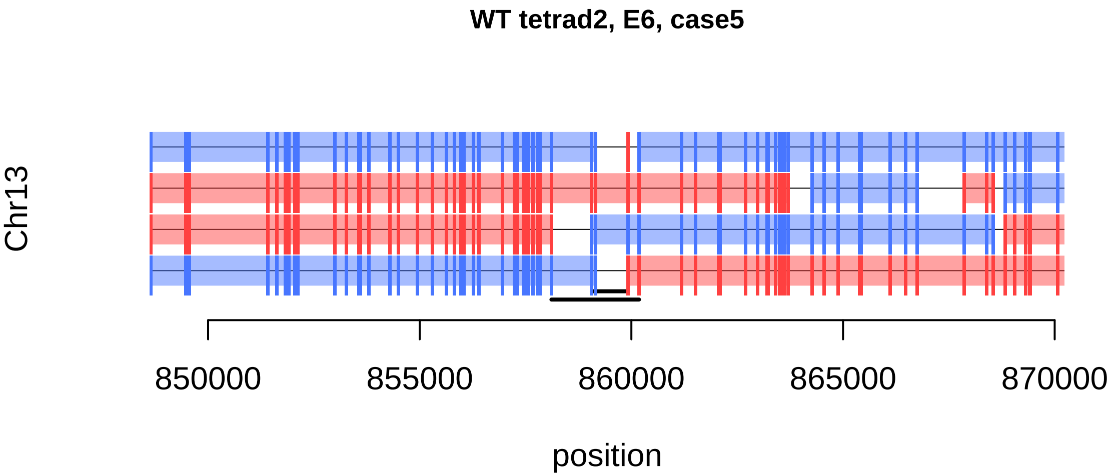
WT tetrad2, E6, case5
Chr13
850000
855000
860000
865000
870000
position

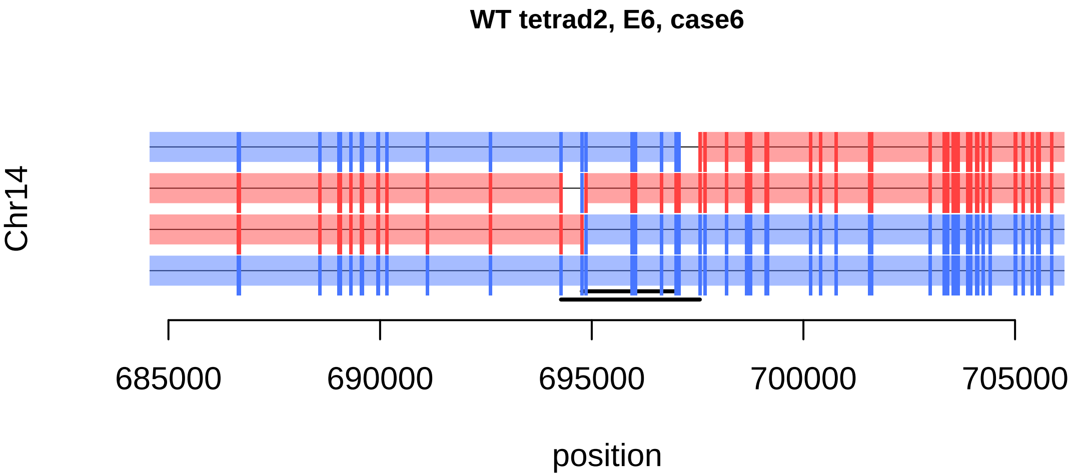
WT tetrad2, E6, case6
Chr14
685000
690000
695000
700000
705000
position

## WT tetrad2, E6, case7

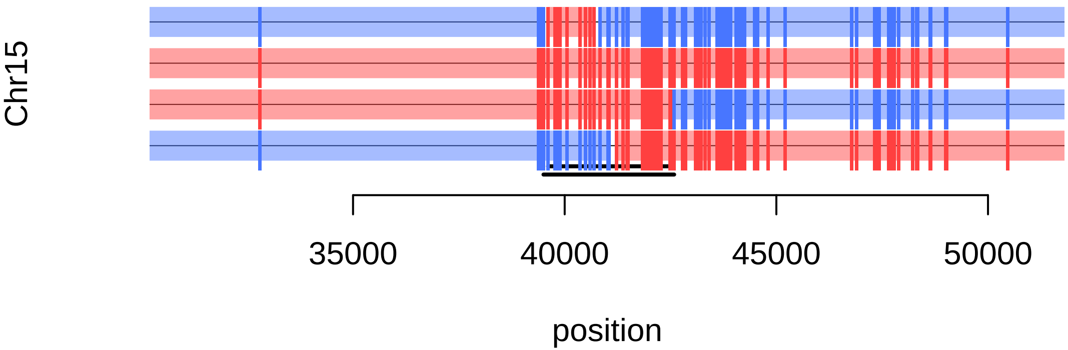

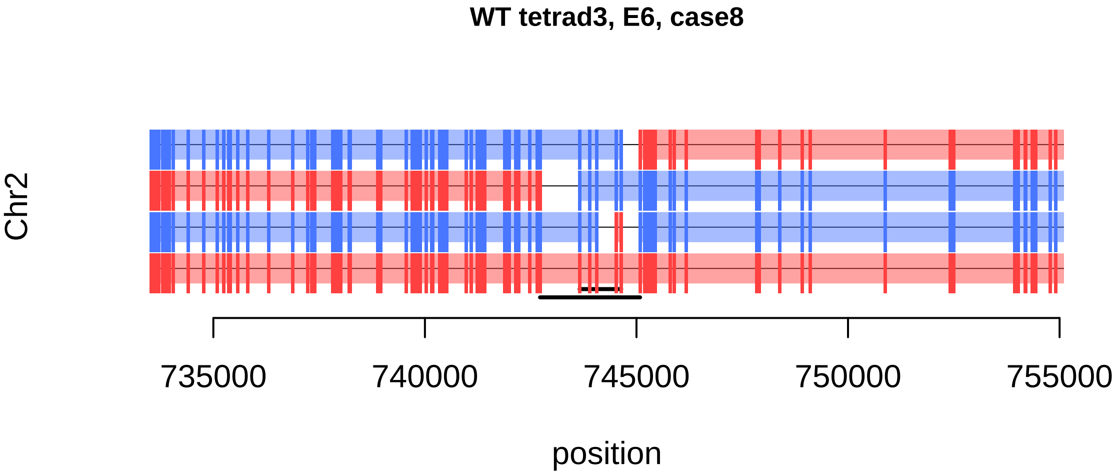
WT tetrad3, E6, case8
Chr2
735000
740000
745000
750000
755000
position

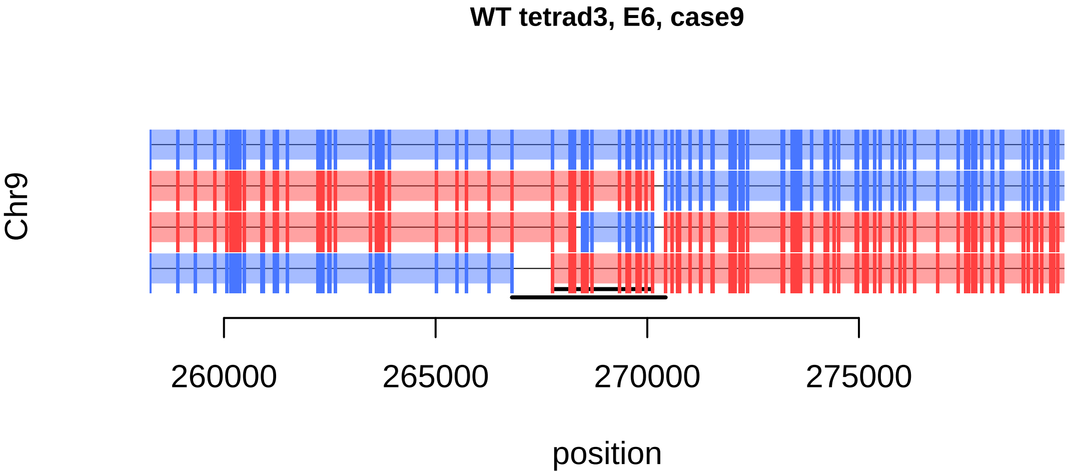
WT tetrad3, E6, case9
Chr9
260000
265000
270000
275000
position

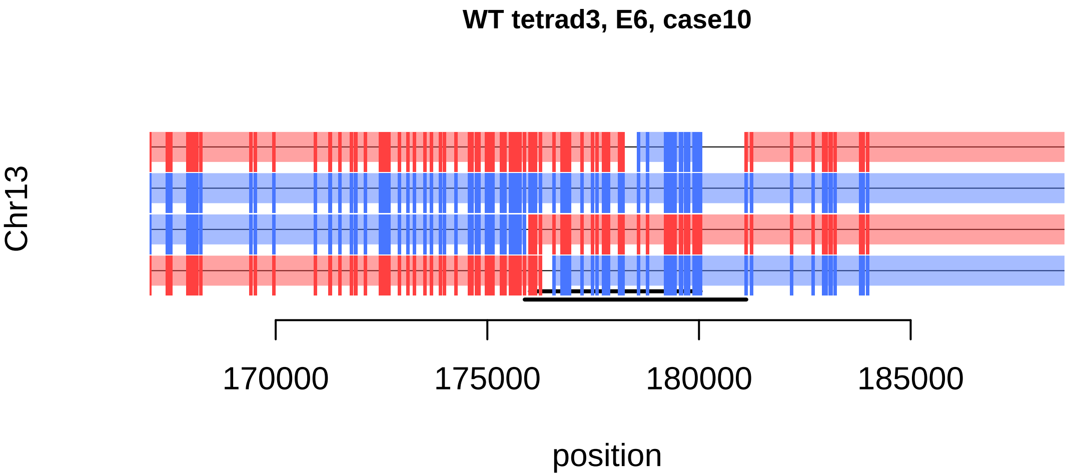

WT tetrad3, E6, case10
Chr13
170000
175000
180000
185000
position

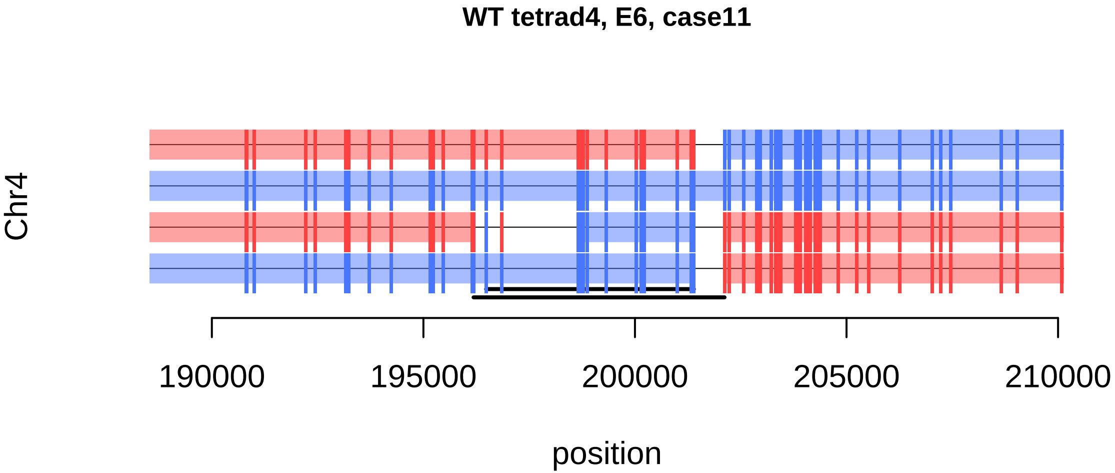

WT tetrad4, E6, case11
Chr4
190000
195000
200000
205000
210000
position

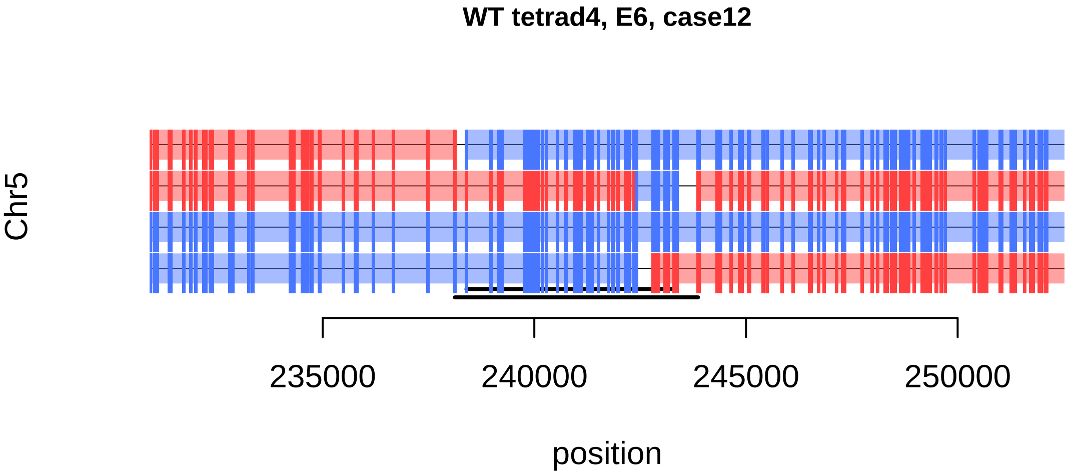
WT tetrad4, E6, case12
Chr5
235000
240000
245000
250000
position

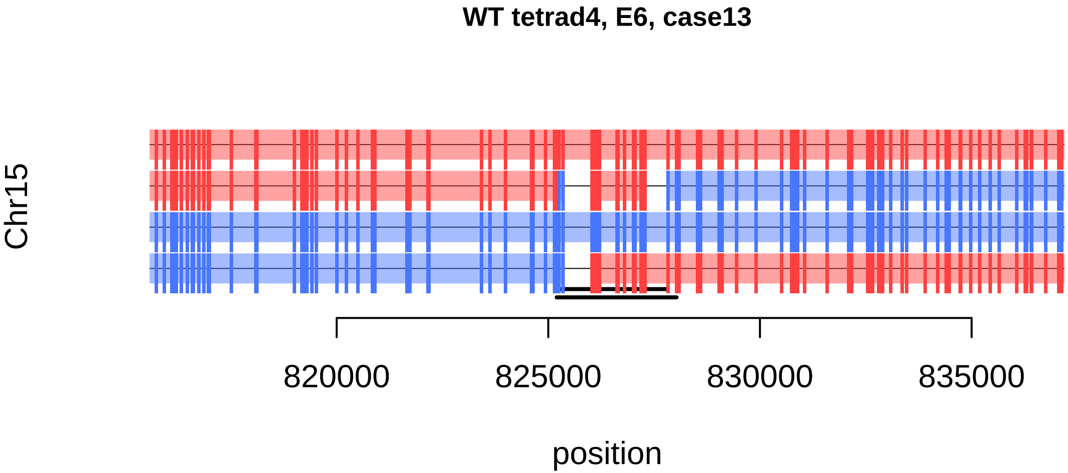
WT tetrad4, E6, case13
Chr15
820000
825000
830000
835000
position

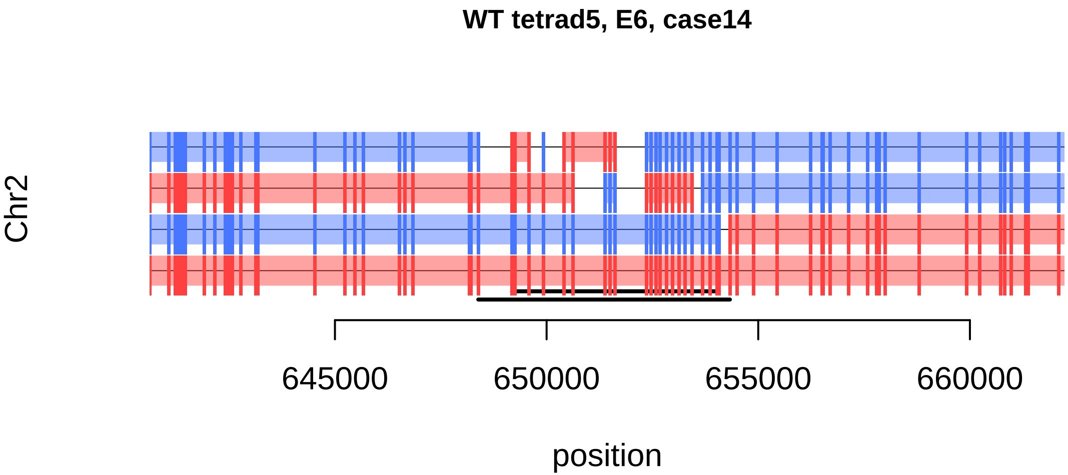

WT tetrad5, E6, case14
Chr2
645000
650000
655000
660000
position

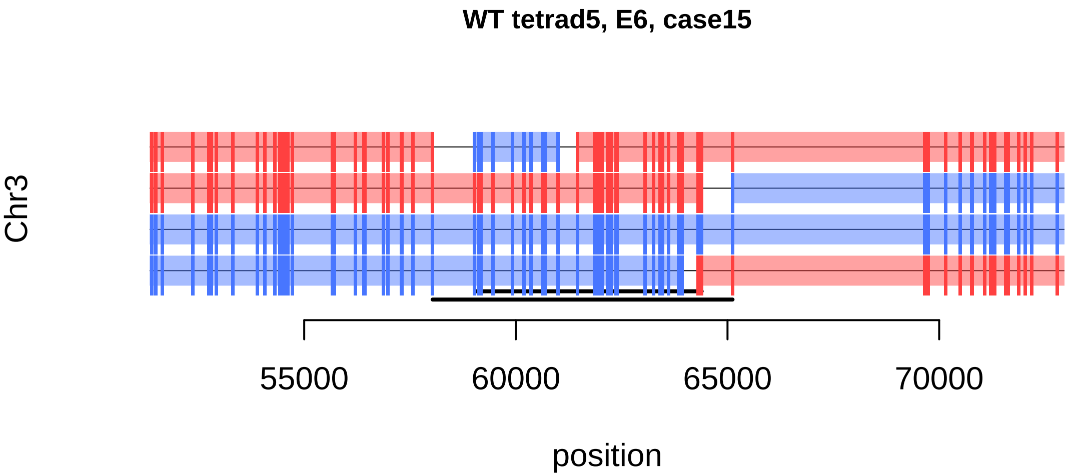
WT tetrad5, E6, case15
Chr3
55000
60000
65000
70000
position

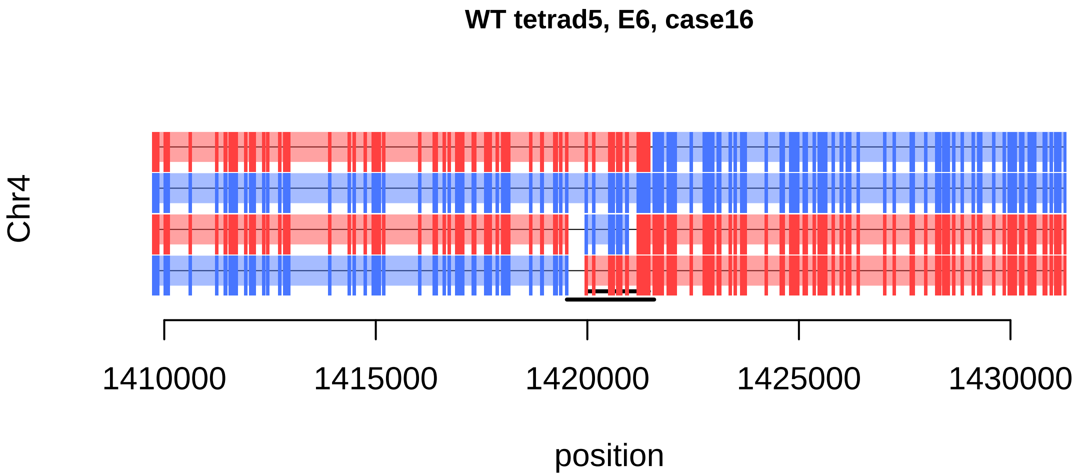
WT tetrad5, E6, case16
Chr4
1410000
1415000
1420000
1425000
1430000
position

WT tetrad5, E6, case17

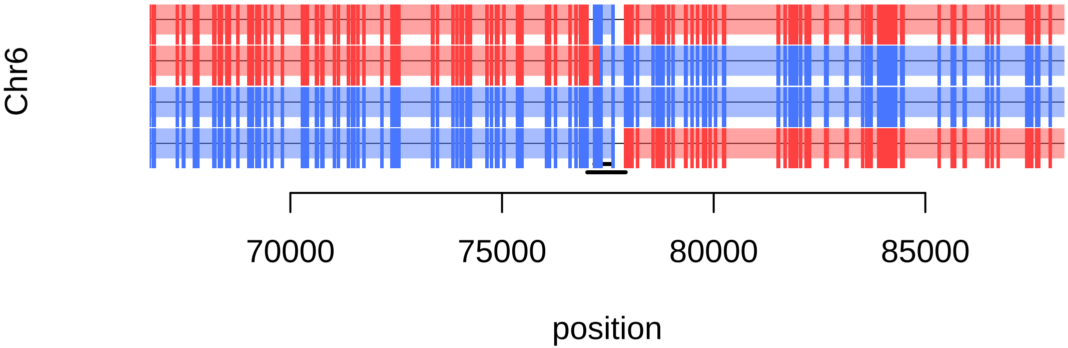

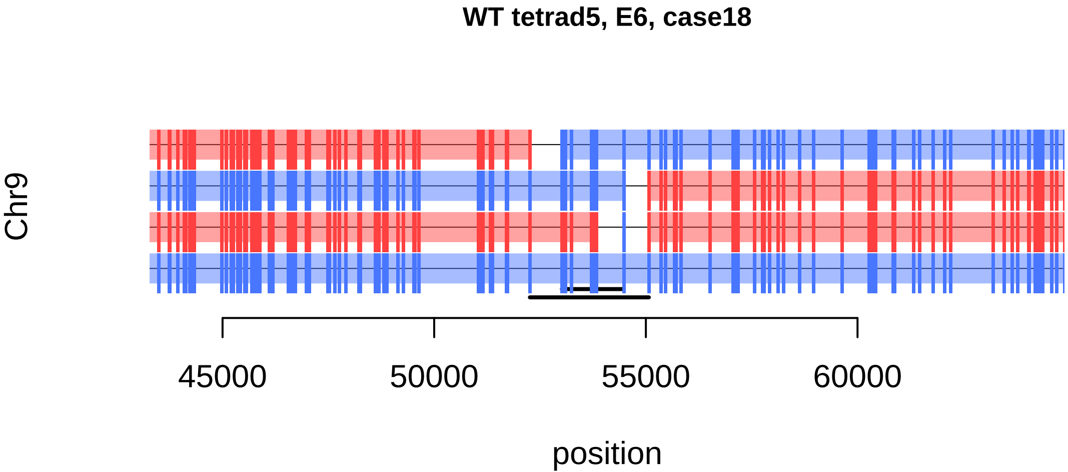

WT tetrad5, E6, case18
Chr9
45000
50000
55000
60000
position

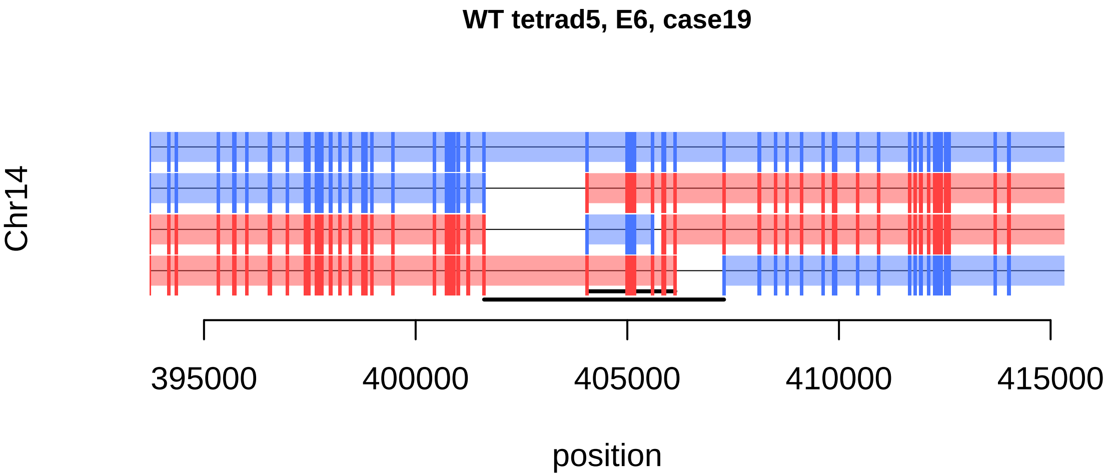
WT tetrad5, E6, case19
Chr14
395000
400000
405000
410000
415000
position

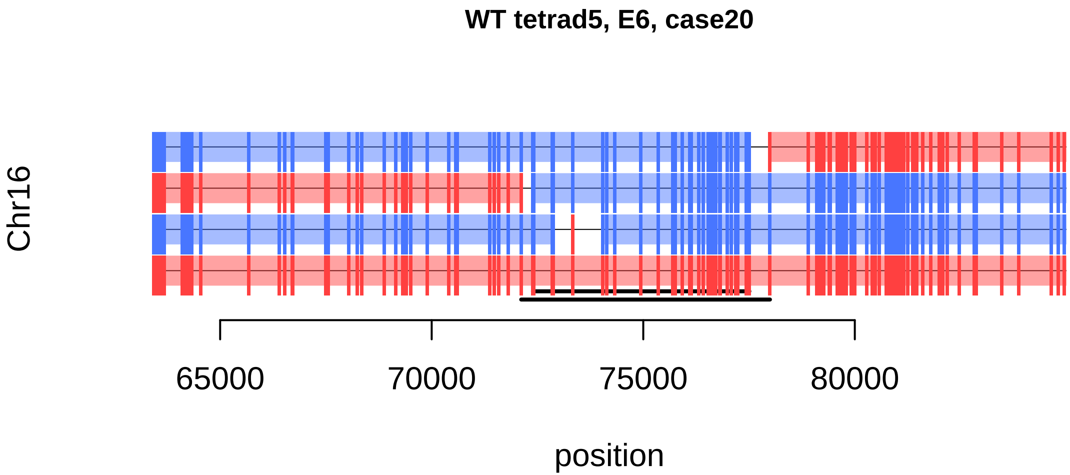

WT tetrad5, E6, case20
Chr16
65000
70000
75000
80000
position

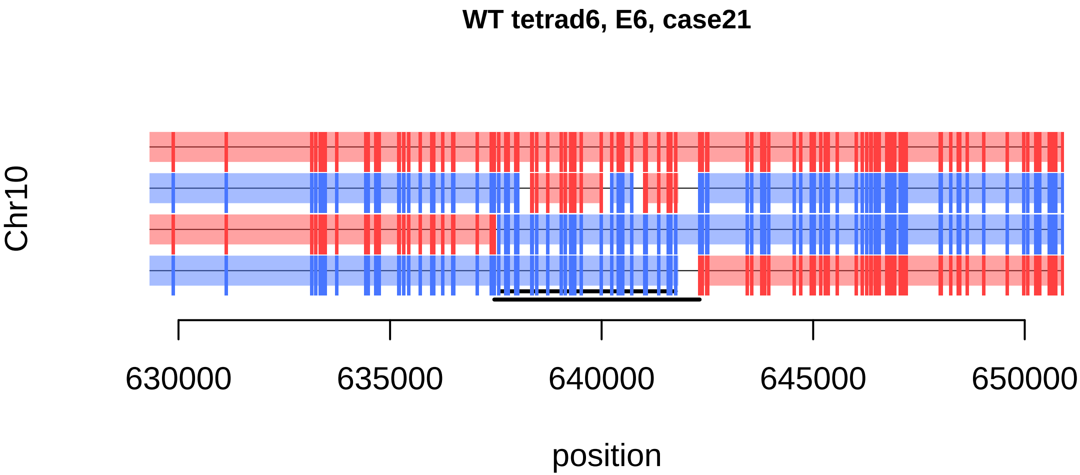
WT tetrad6, E6, case21
Chr10
630000
635000
640000
645000
650000
position

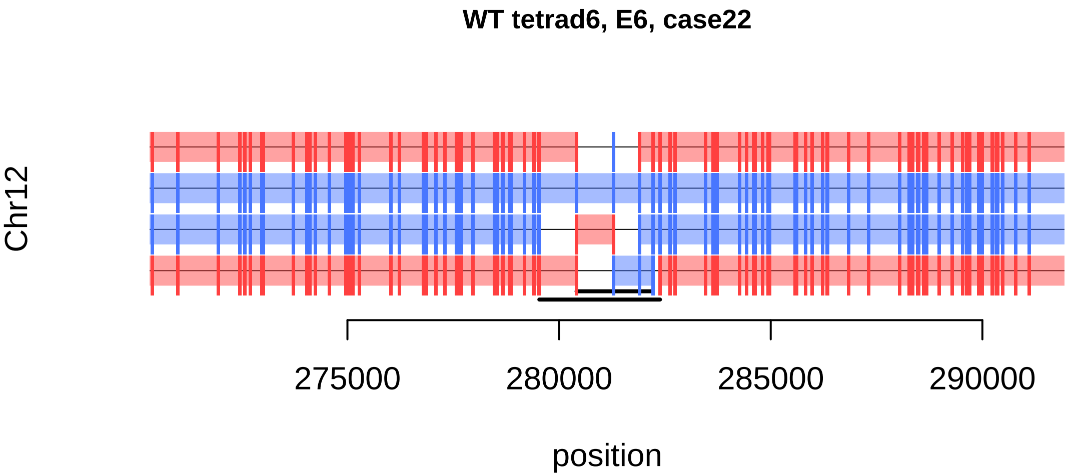

WT tetrad6, E6, case22
Chr12
275000
280000
285000
290000
position

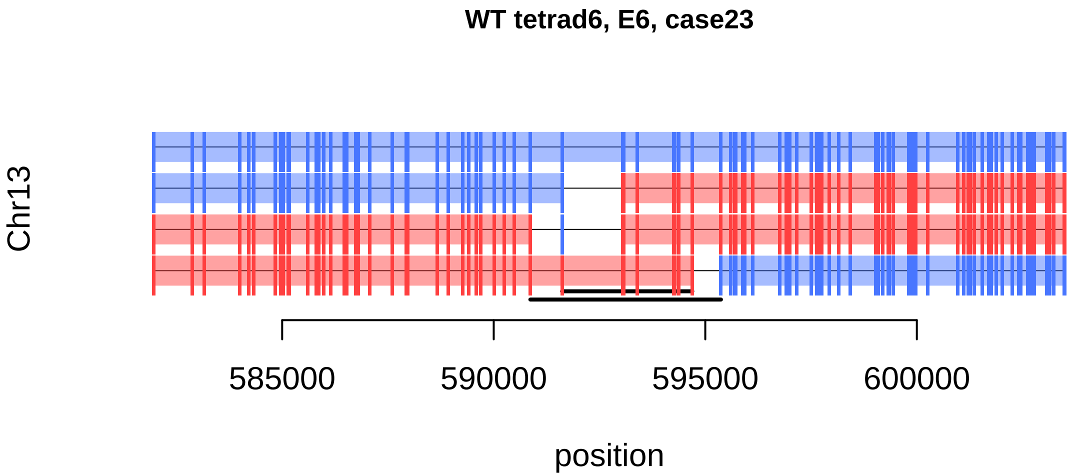
WT tetrad6, E6, case23
Chr13
585000
590000
595000
600000
position

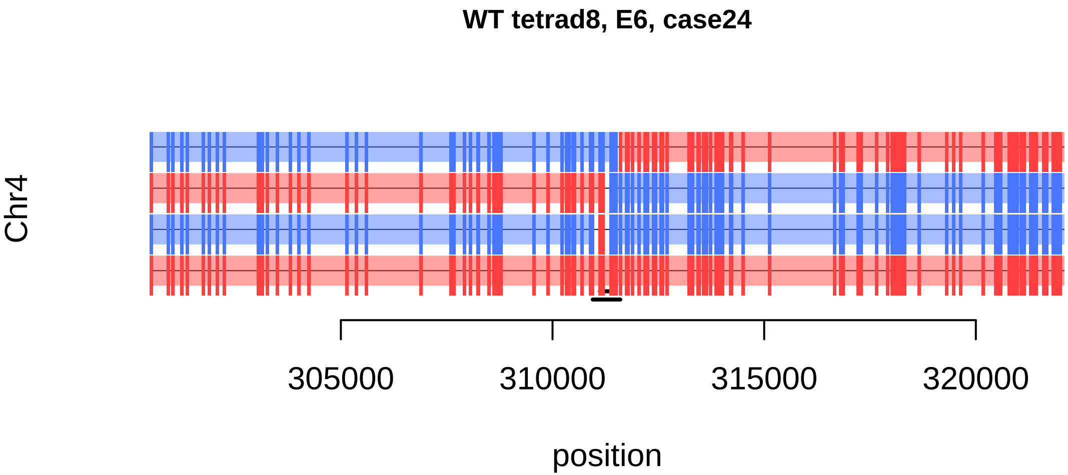
WT tetrad8, E6, case24
Chr4
305000
310000
315000
320000
position

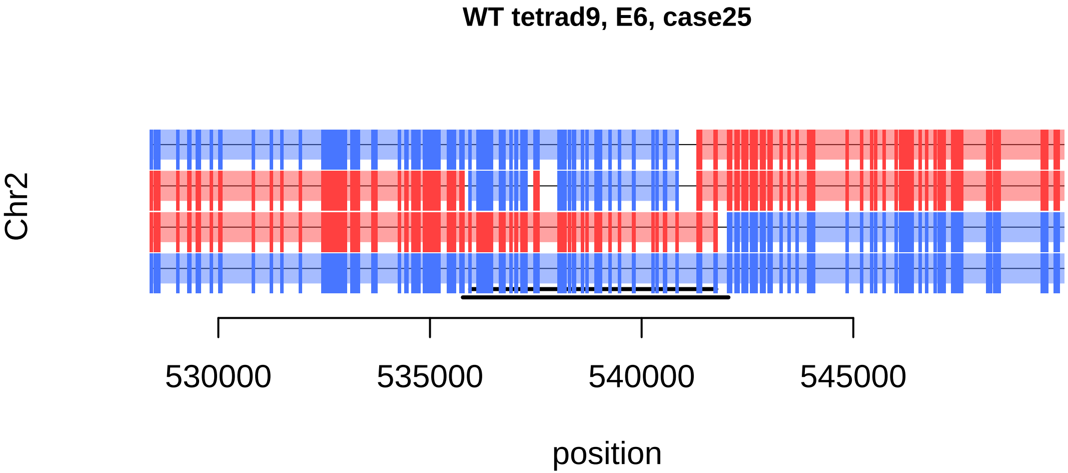
WT tetrad9, E6, case25
Chr2
530000
535000
540000
545000
position

## WT tetrad9, E6, case26

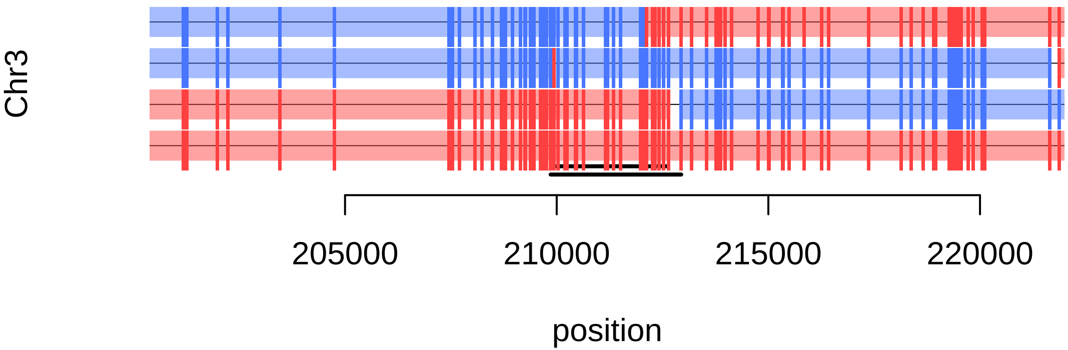

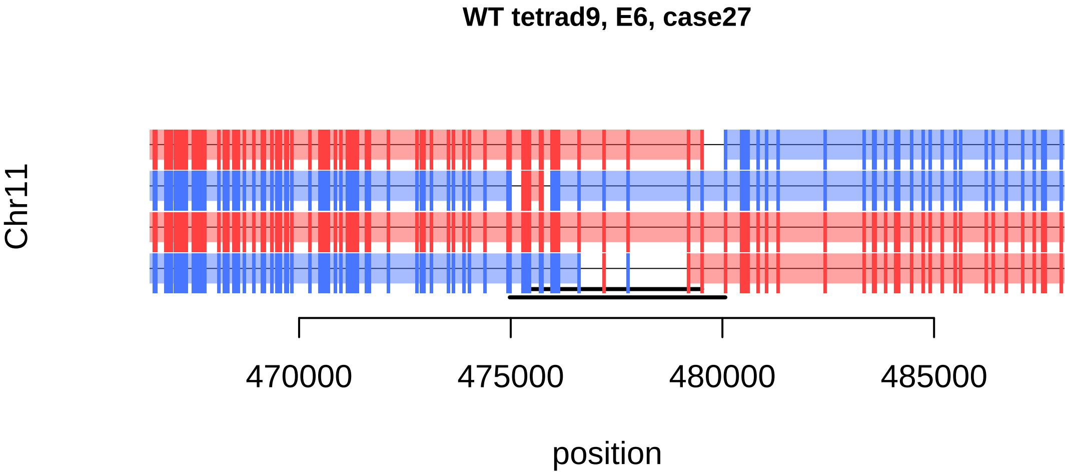
WT tetrad9, E6, case27
Chr11
470000
475000
480000
485000
position

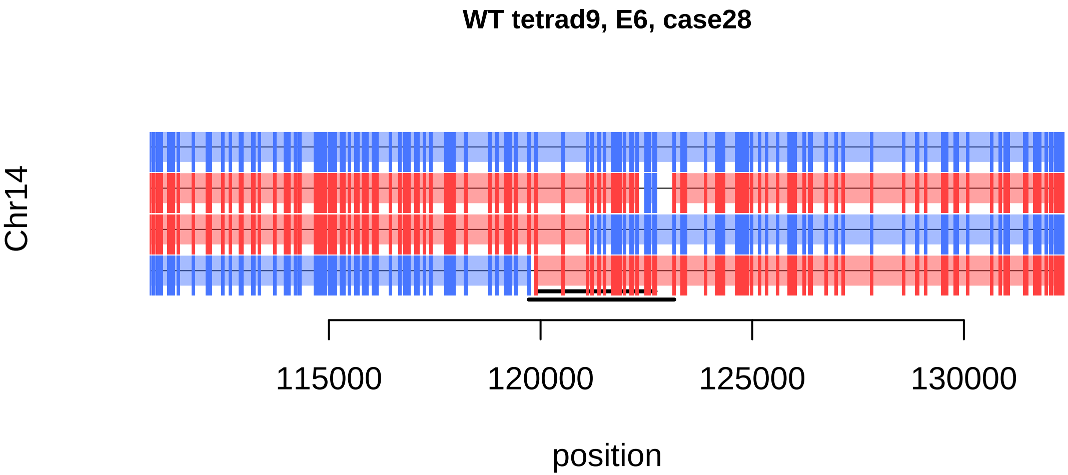
WT tetrad9, E6, case28
Chr14
115000
120000
125000
130000
position

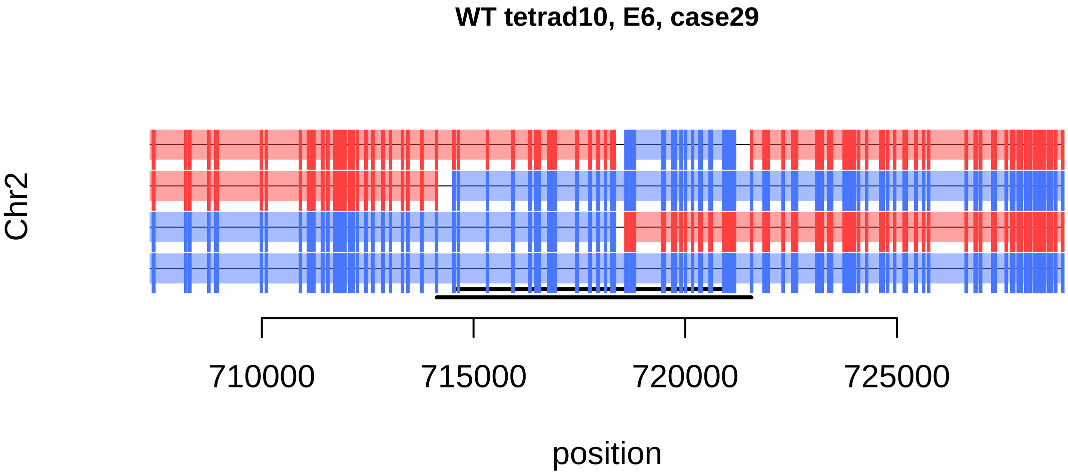
WT tetrad10, E6, case29
Chr2
710000
715000
720000
725000
position

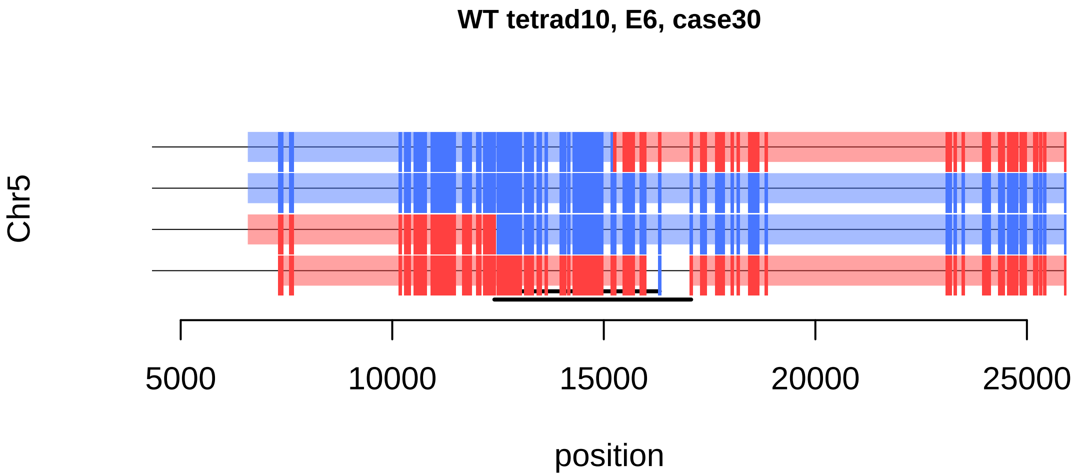
WT tetrad10, E6, case30
Chr5
5000
10000
15000
20000
25000
position

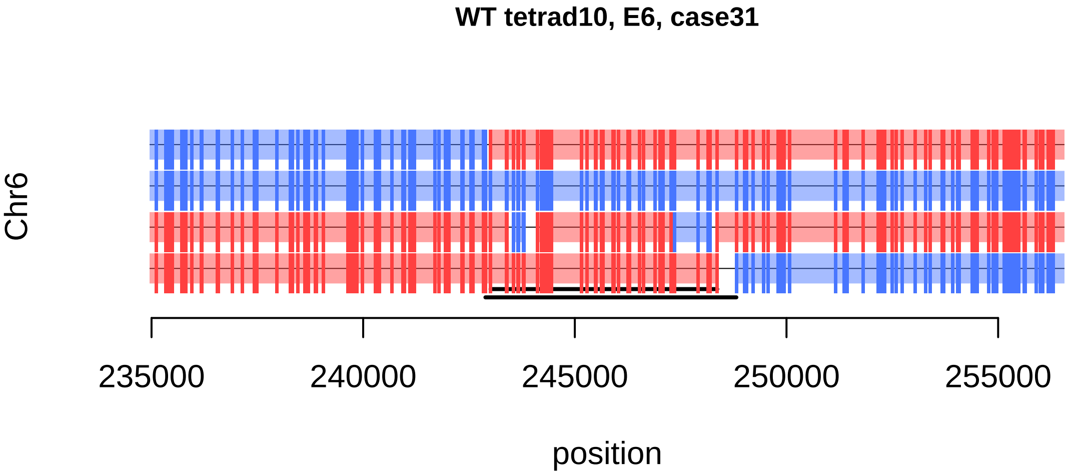

WT tetrad10, E6, case31
Chr6
235000
240000
245000
250000
255000
position

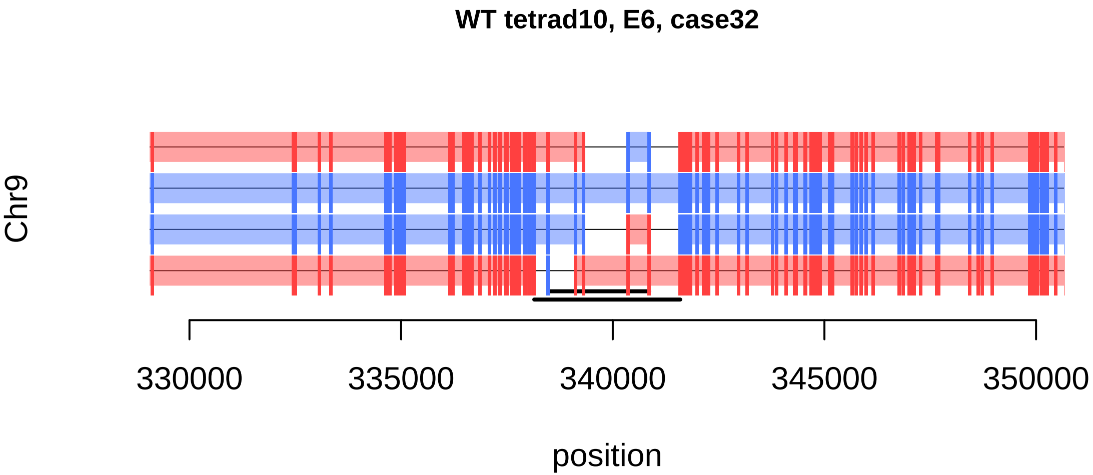
WT tetrad10, E6, case32
Chr9
330000
335000
340000
345000
350000
position

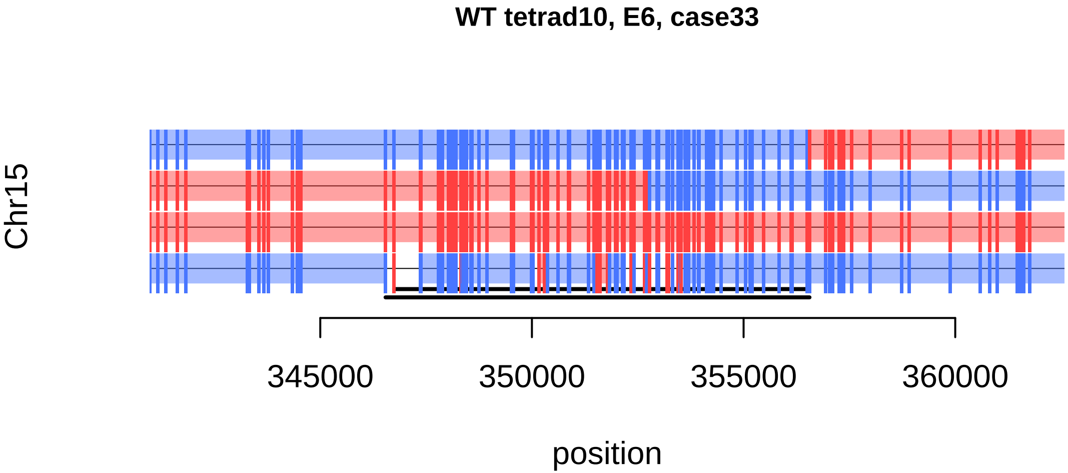
WT tetrad10, E6, case33
Chr15
345000
350000
355000
360000
position

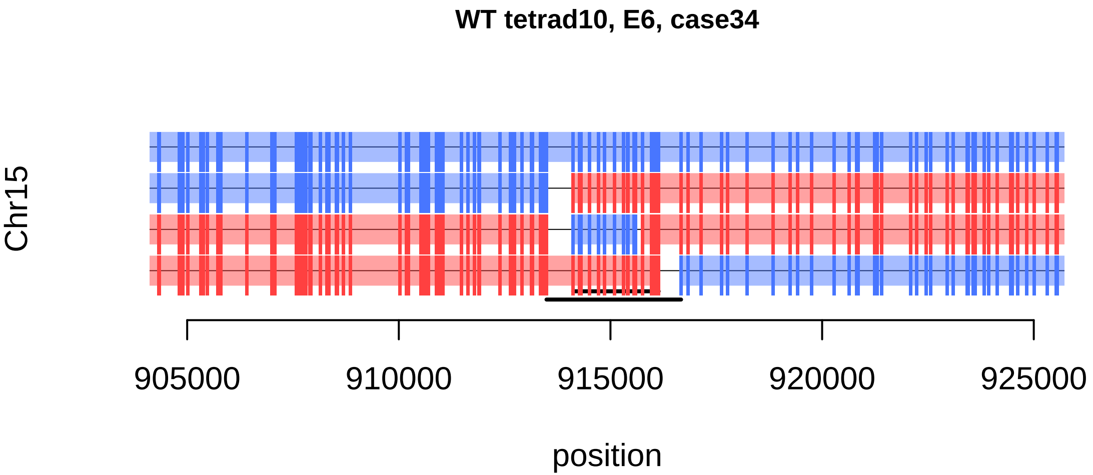

WT tetrad10, E6, case34
Chr15
905000
910000
915000
920000
925000
position

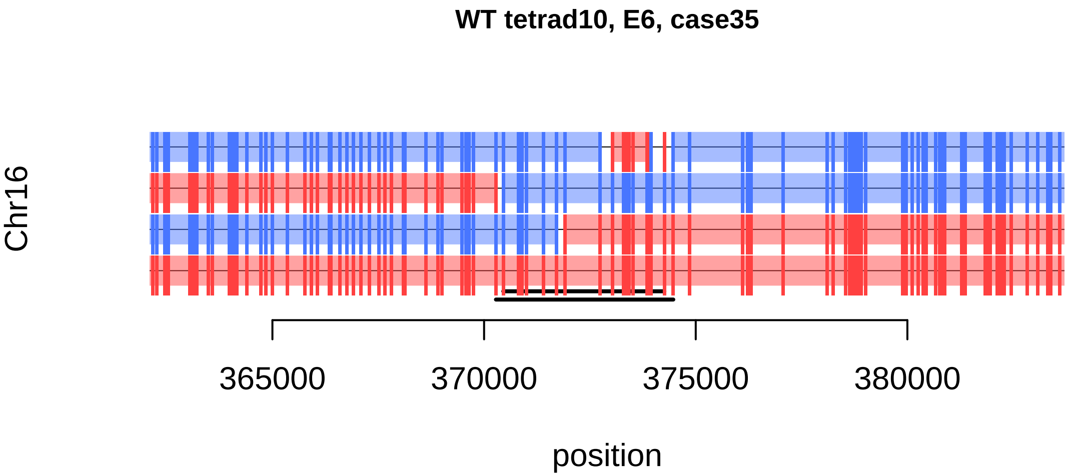

WT tetrad10, E6, case35
Chr16
365000
370000
375000
380000
position

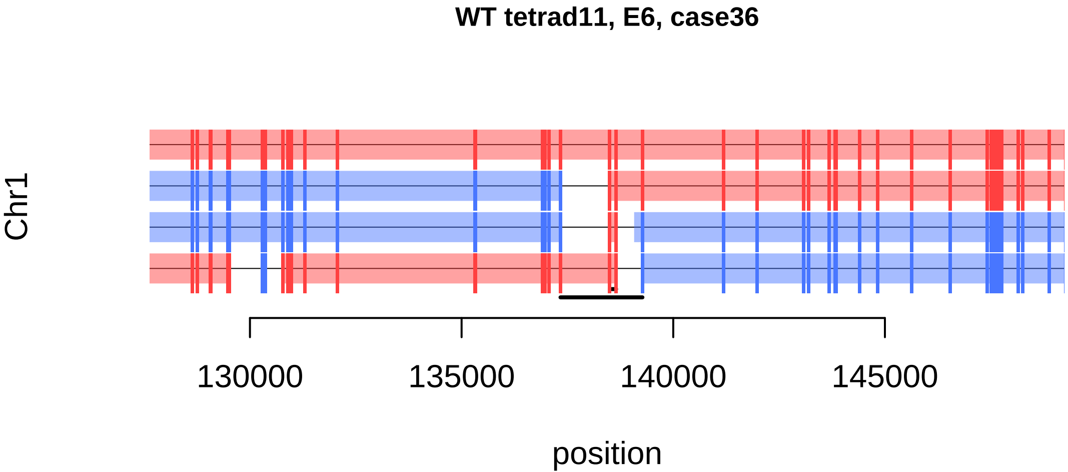
WT tetrad11, E6, case36
Chr1
130000
135000
140000
145000
position

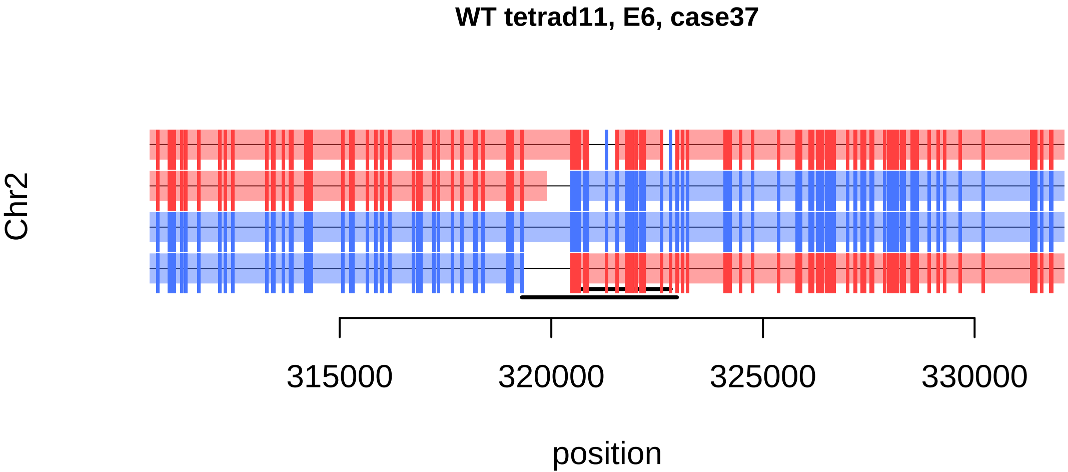
WT tetrad11, E6, case37
Chr2
315000
320000
325000
330000
position

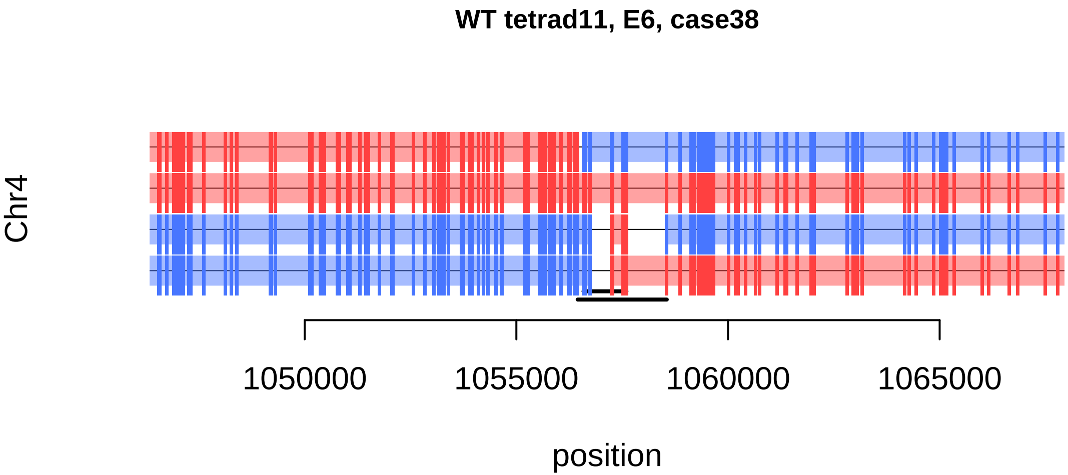
WT tetrad11, E6, case38
Chr4
1050000
1055000
1060000
1065000
position

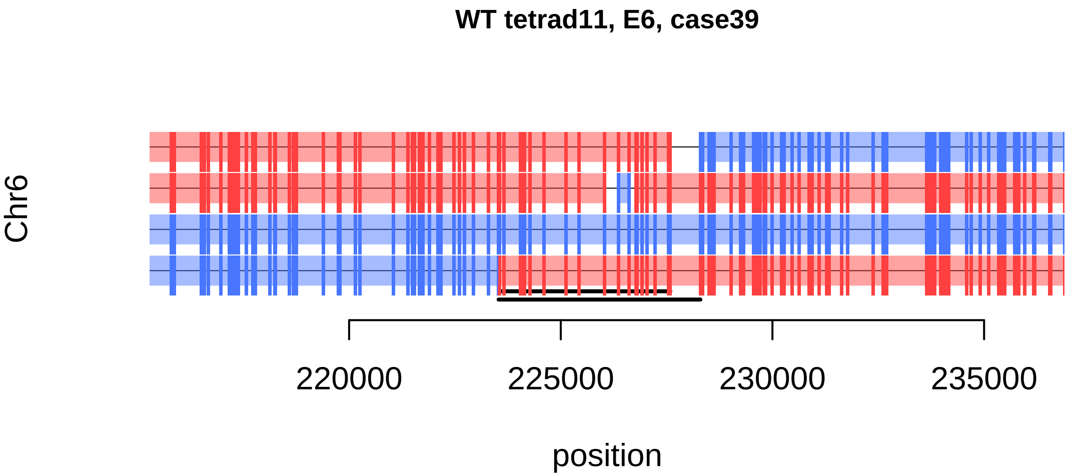
WT tetrad11, E6, case39
Chr6
220000
225000
230000
235000
position

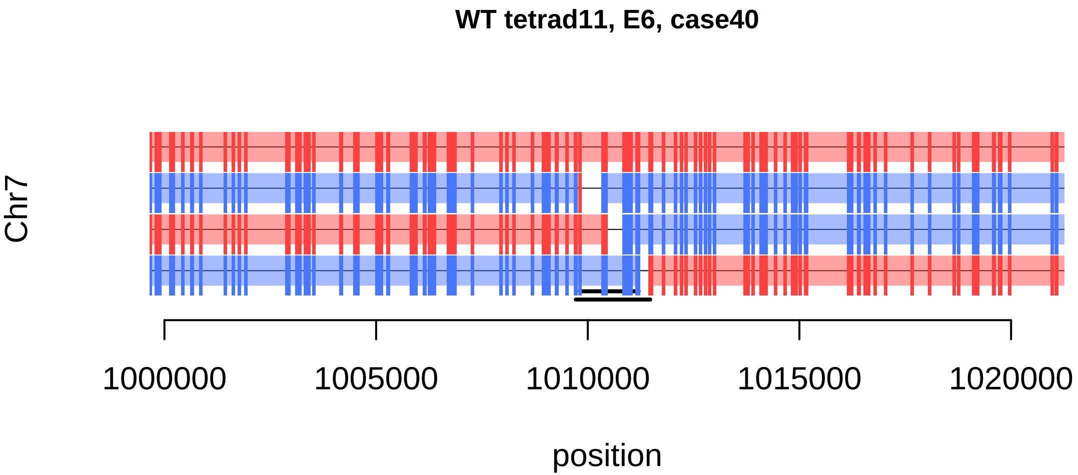
WT tetrad11, E6, case40
Chr7
1000000
1005000
1010000
1015000
1020000
position

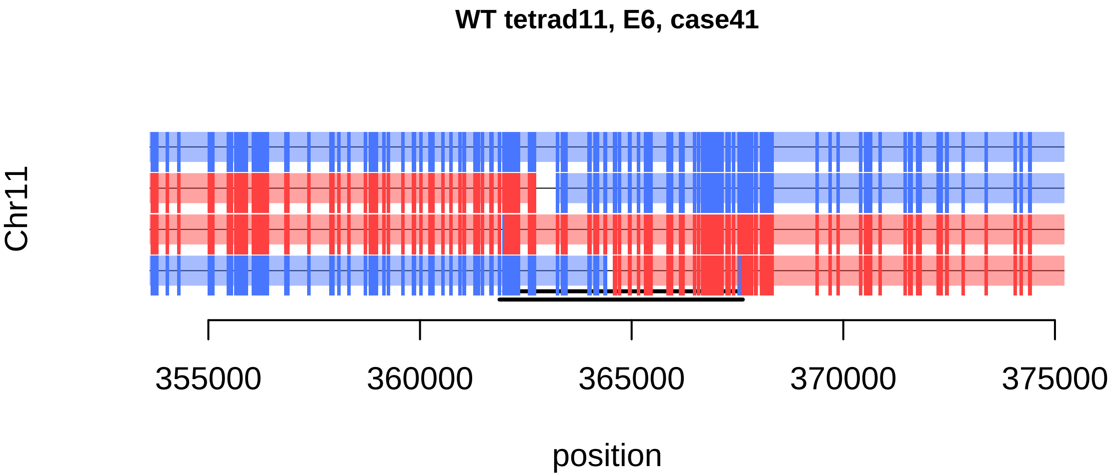

WT tetrad11, E6, case41
Chr11
355000
360000
365000
370000
375000
position

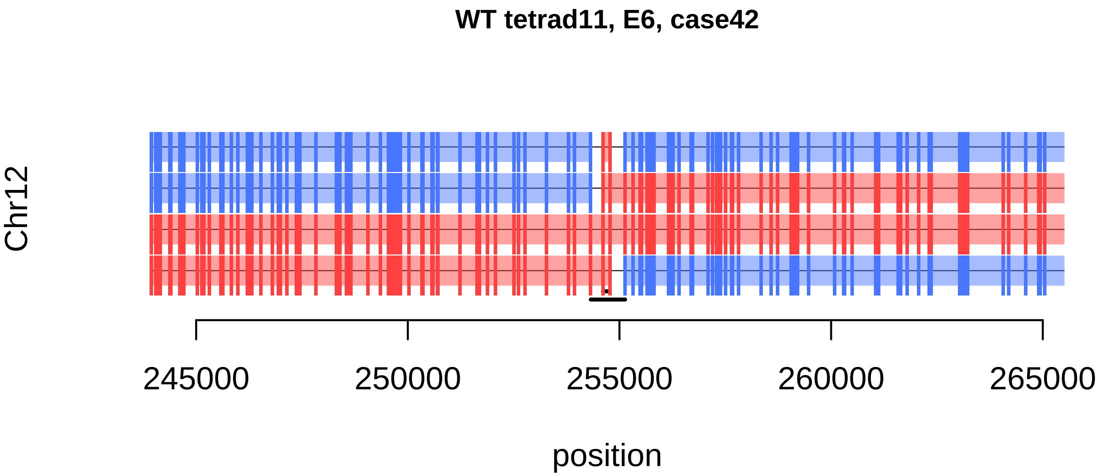
WT tetrad11, E6, case42
Chr12
245000
250000
255000
260000
265000
position

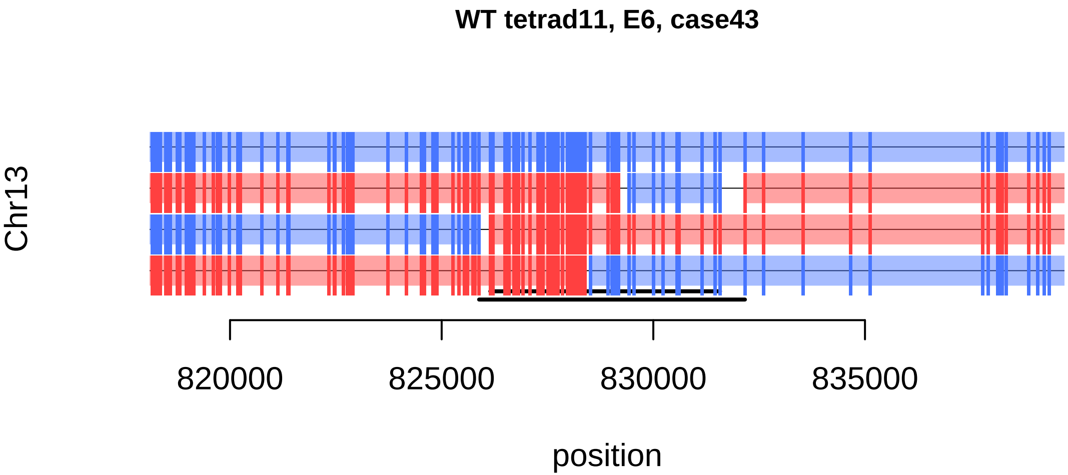

WT tetrad11, E6, case43
Chr13
820000
825000
830000
835000
position

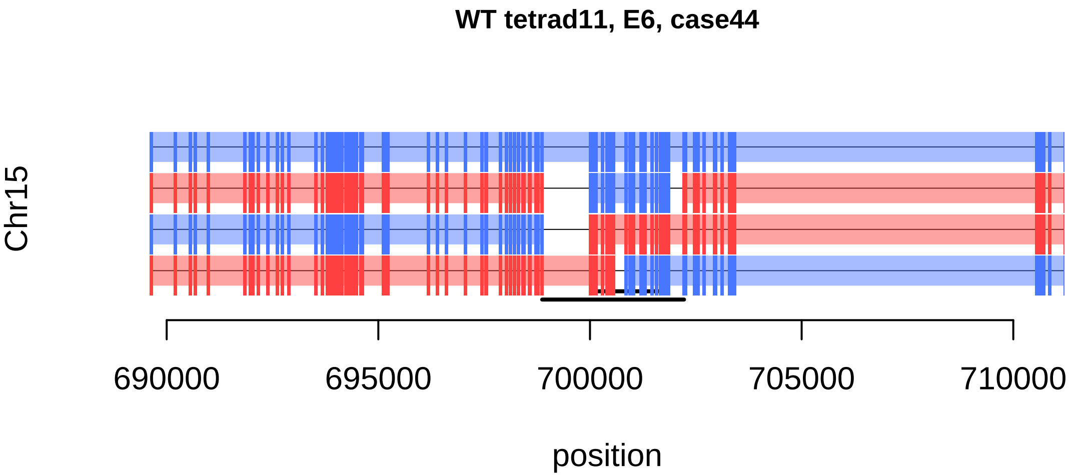
WT tetrad11, E6, case44
Chr15
690000
695000
700000
705000
710000
position

WT tetrad11, E6, case45

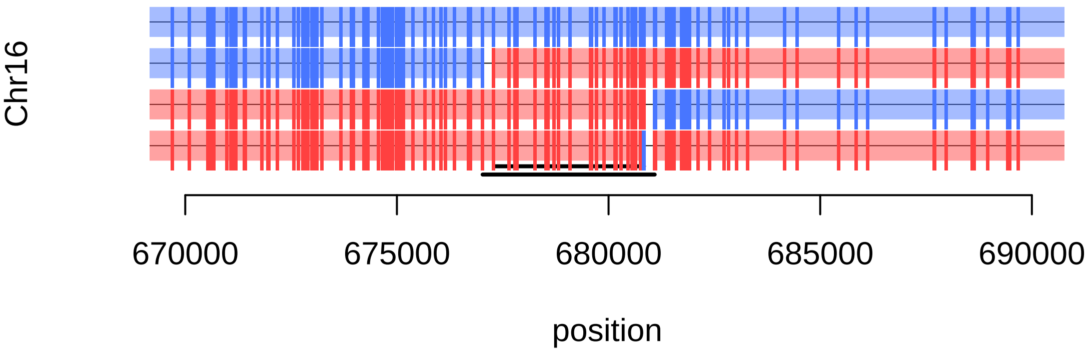

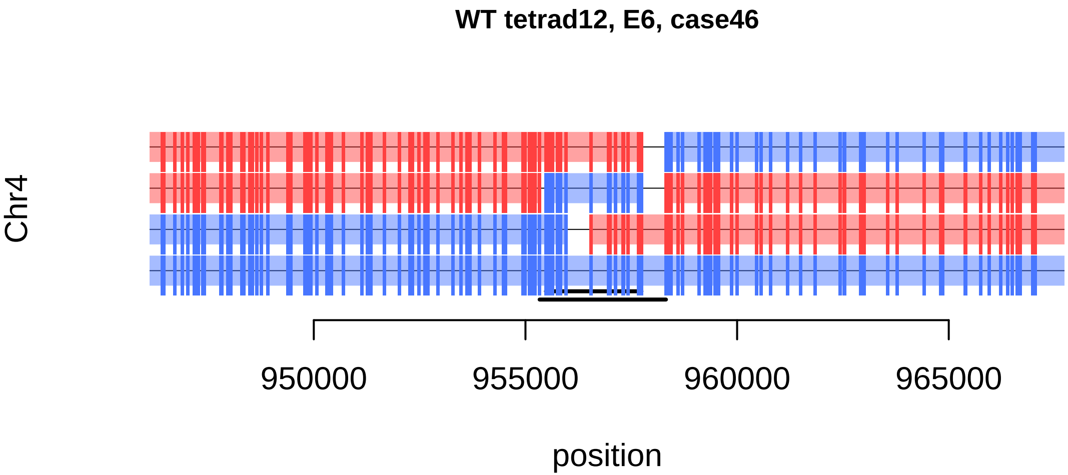
WT tetrad12, E6, case46
Chr4
950000
955000
960000
965000
position

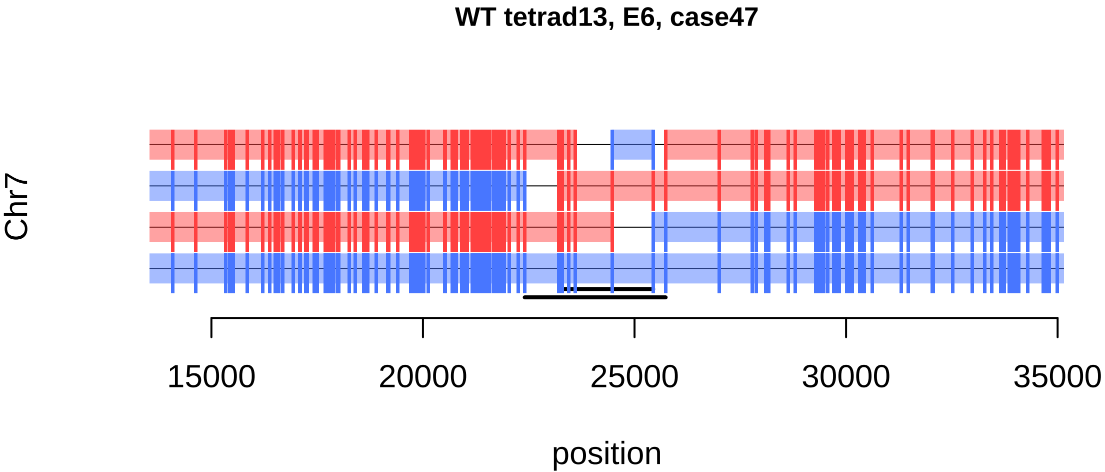
WT tetrad13, E6, case47
Chr7
15000
20000
25000
30000
35000
position

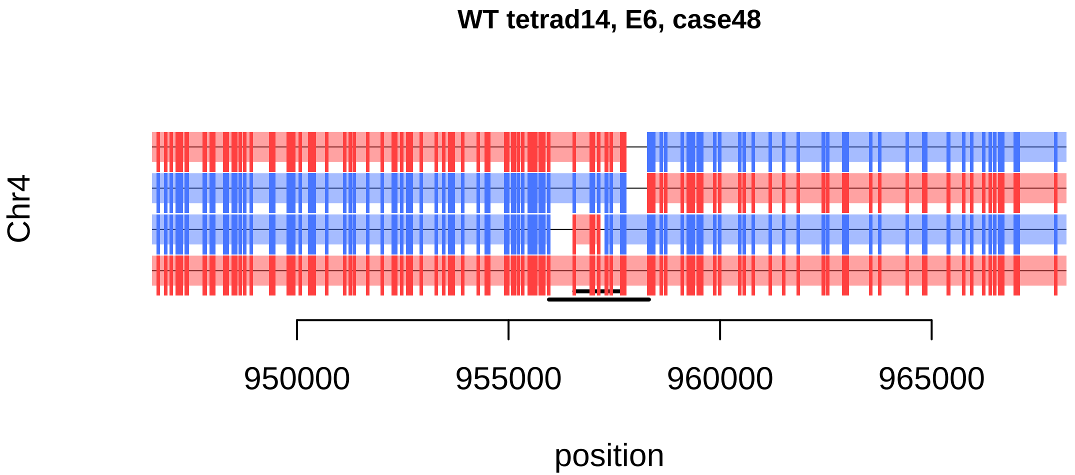
WT tetrad14, E6, case48
Chr4
950000
955000
960000
965000
position

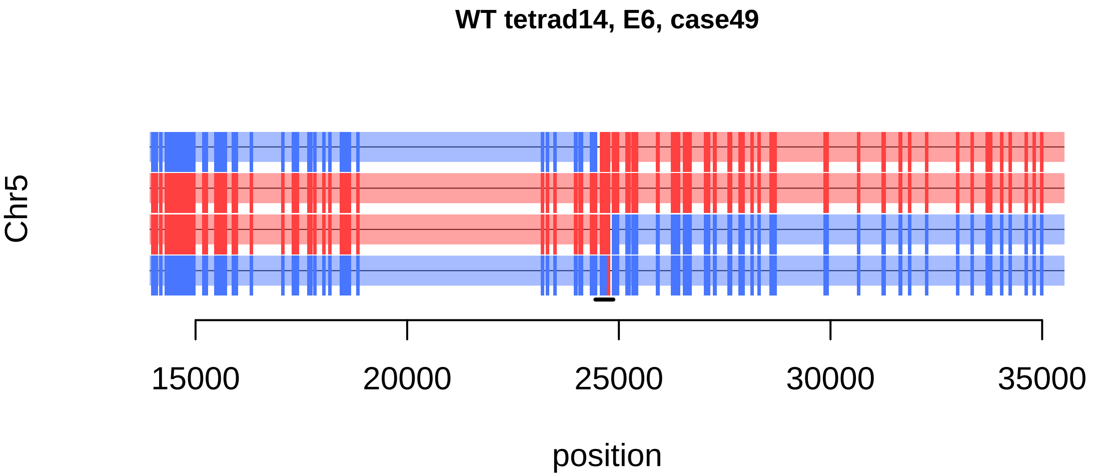
WT tetrad14, E6, case49
Chr5
15000
20000
25000
30000
35000
position

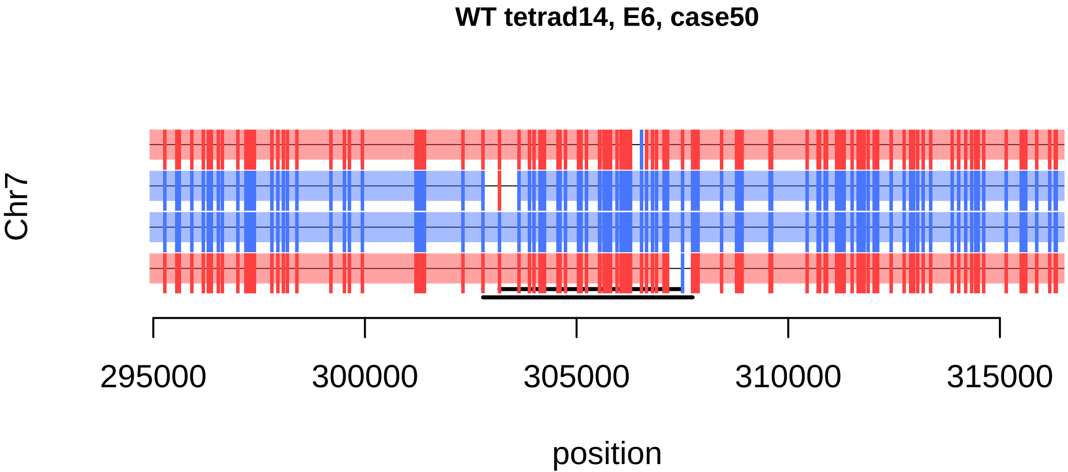
WT tetrad14, E6, case50
Chr7
295000
300000
305000
310000
315000
position

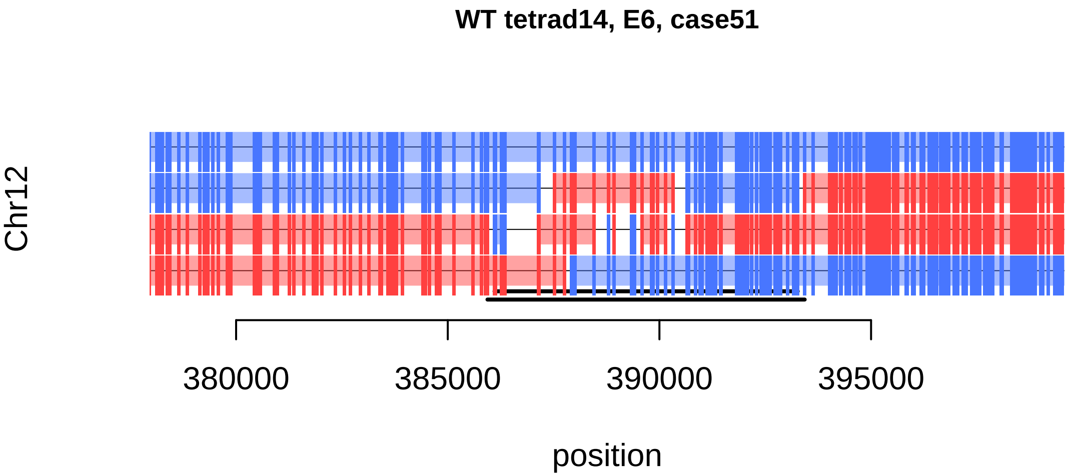

WT tetrad14, E6, case51
Chr12
380000
385000
390000
395000
position

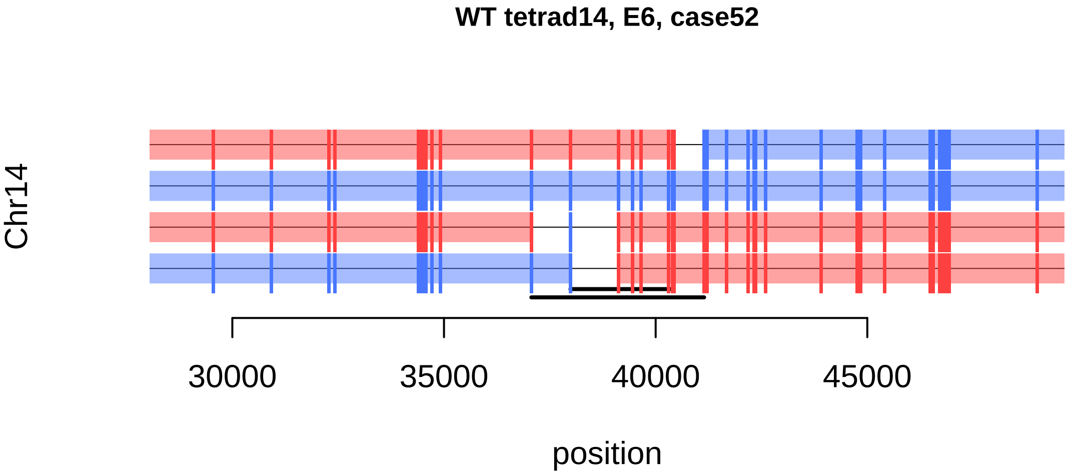
WT tetrad14, E6, case52
Chr14
30000
35000
40000
45000
position

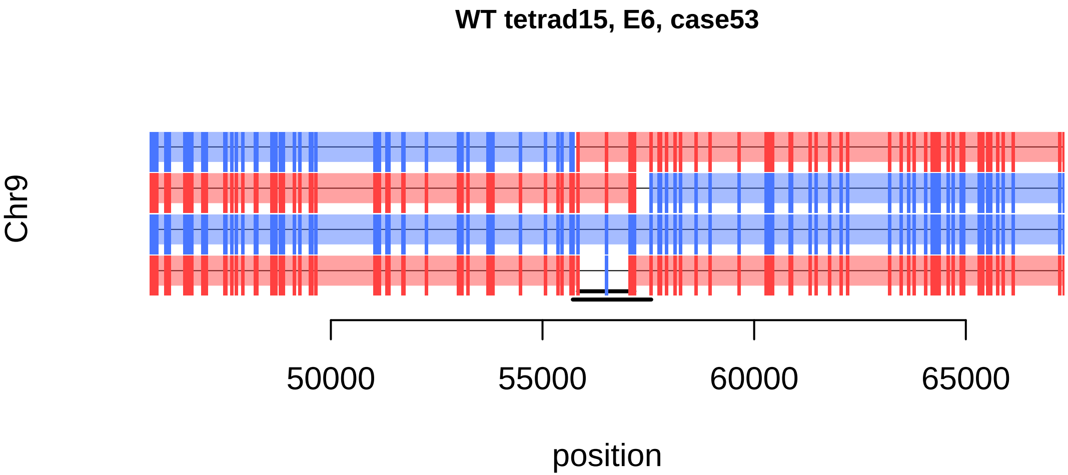
WT tetrad15, E6, case53
Chr9
50000
55000
60000
65000
position

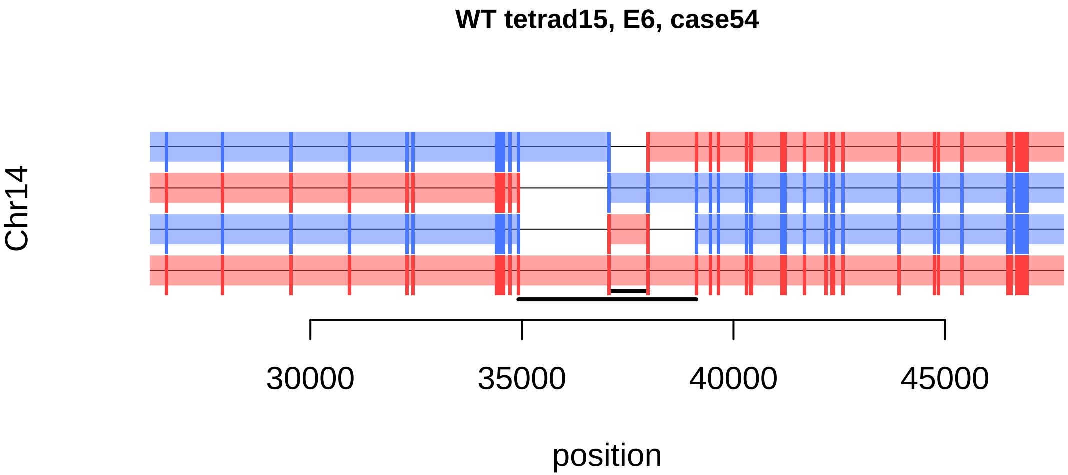
WT tetrad15, E6, case54
Chr14
30000
35000
40000
45000
position

WT tetrad16, E6, case55

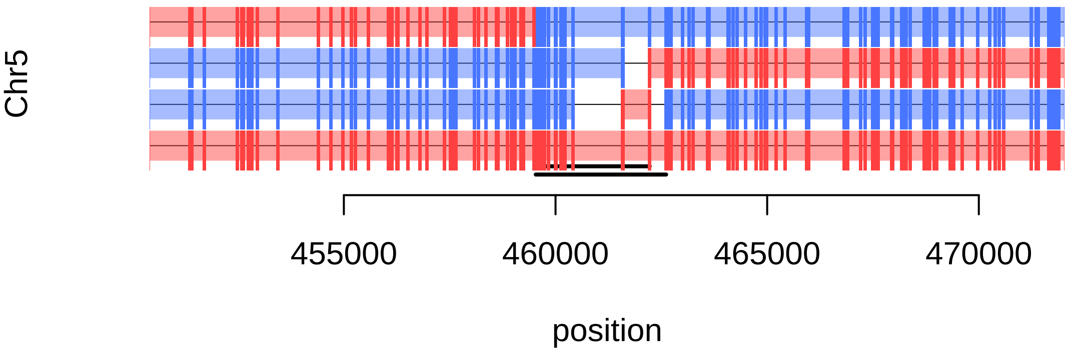

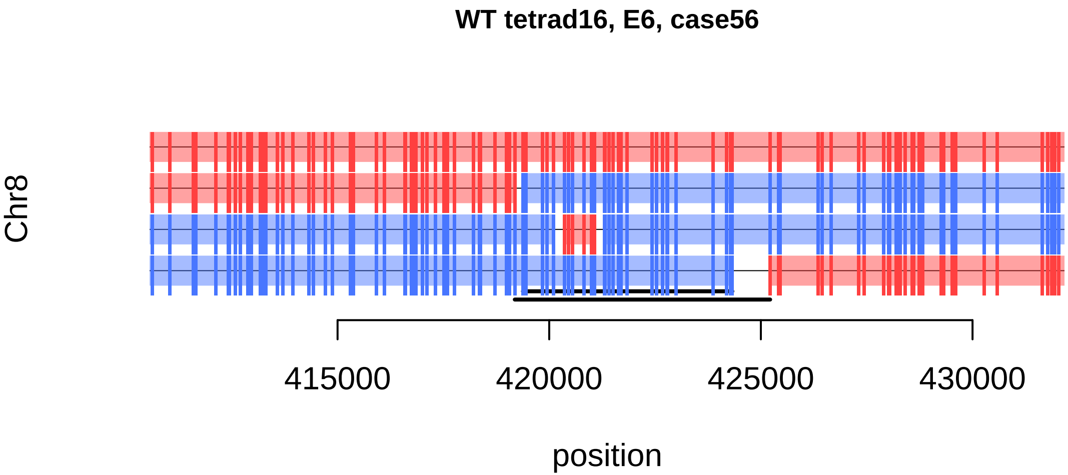
WT tetrad16, E6, case56
Chr8
415000
420000
425000
430000
position

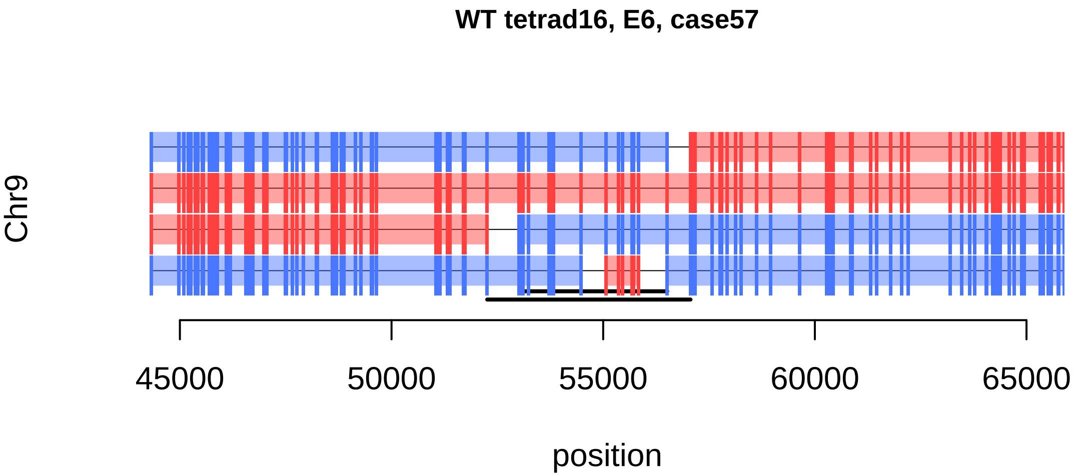
WT tetrad16, E6, case57
Chr9
45000
50000
55000
60000
65000
position

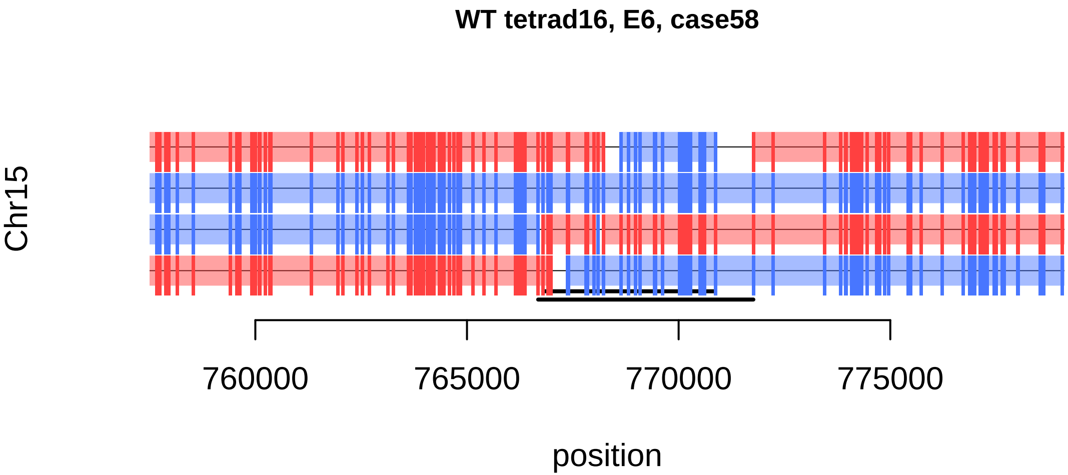

WT tetrad16, E6, case58
Chr15
760000
765000
770000
775000
position

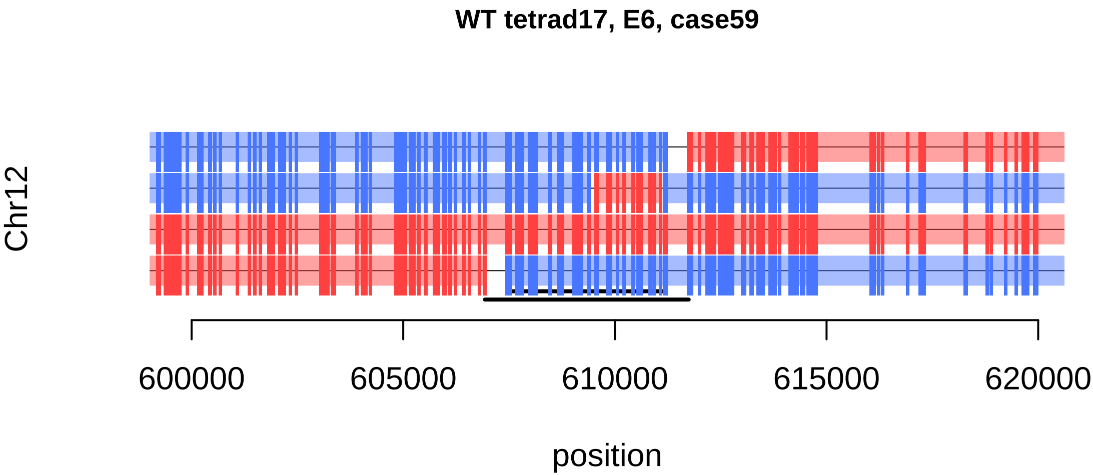
WT tetrad17, E6, case59
Chr12
600000
605000
610000
615000
620000
position

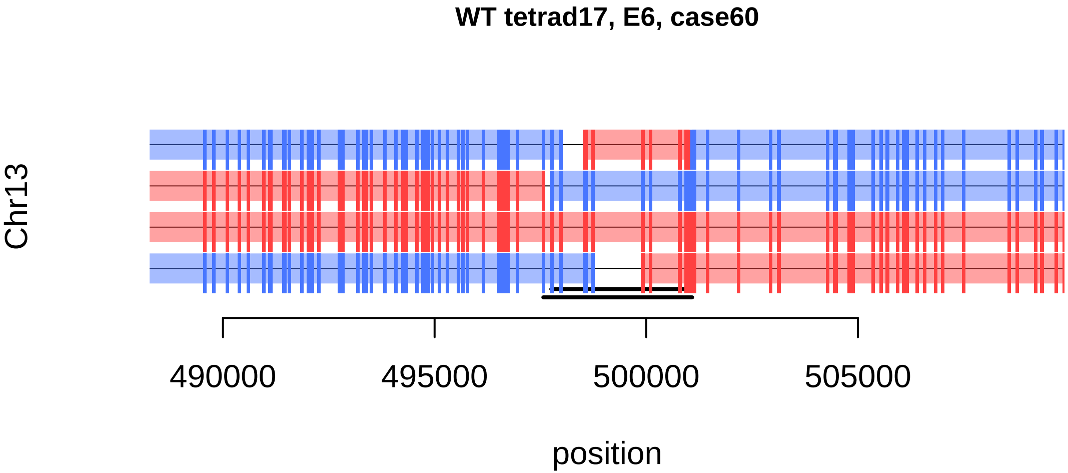
WT tetrad17, E6, case60
Chr13
490000
495000
500000
505000
position

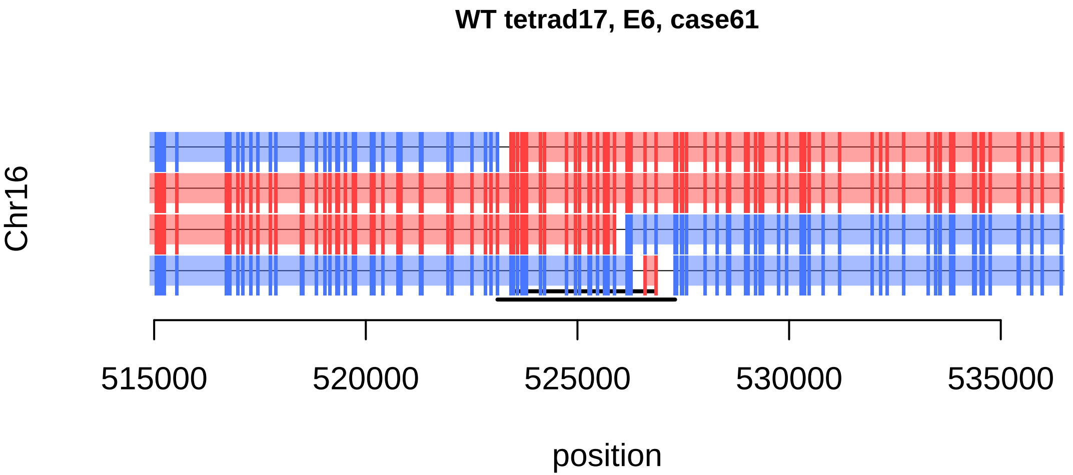
WT tetrad17, E6, case61
Chr16
515000
520000
525000
530000
535000
position

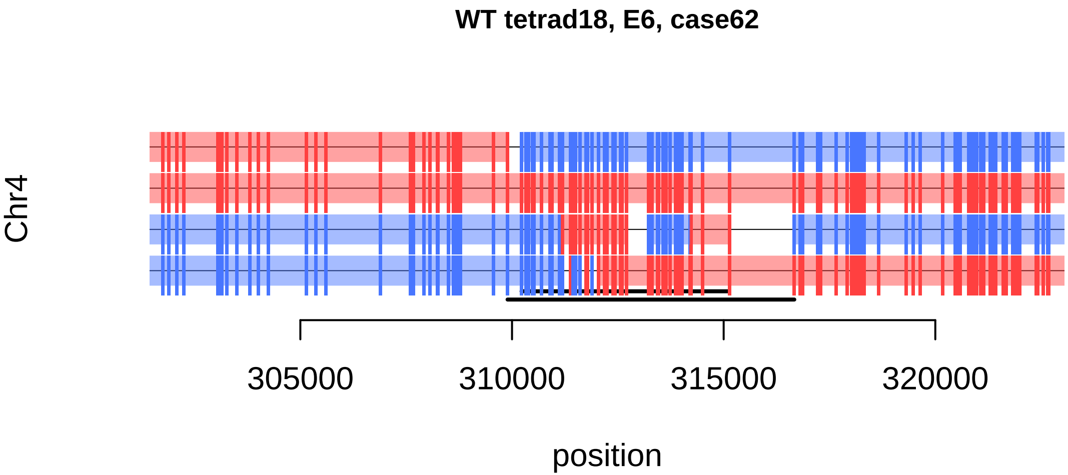
WT tetrad18, E6, case62
Chr4
305000
310000
315000
320000
position

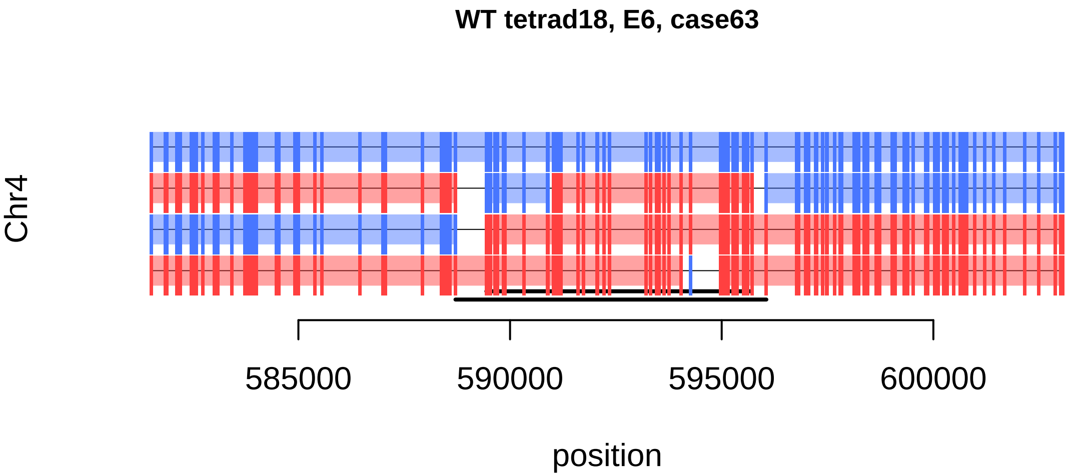
WT tetrad18, E6, case63
Chr4
585000
590000
595000
600000
position

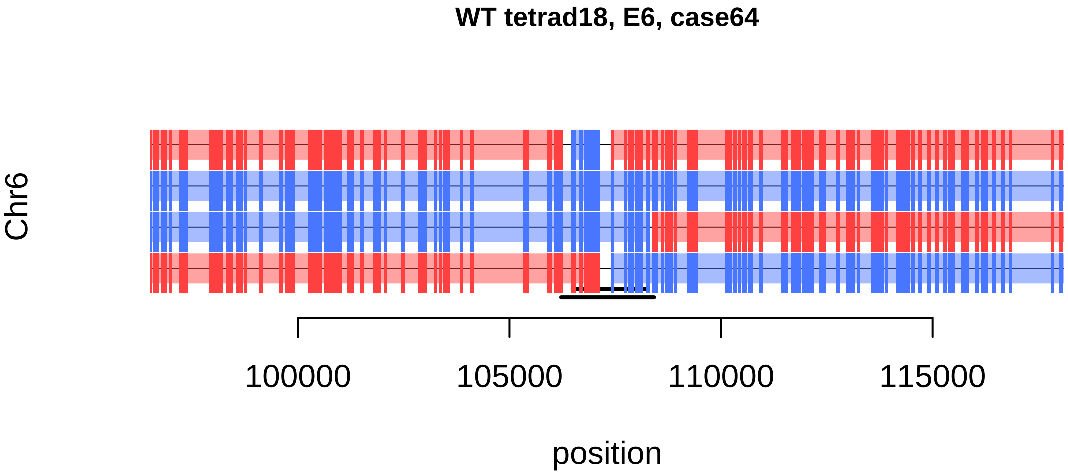
WT tetrad18, E6, case64
Chr6
100000
105000
110000
115000
position

WT tetrad18, E6, case65

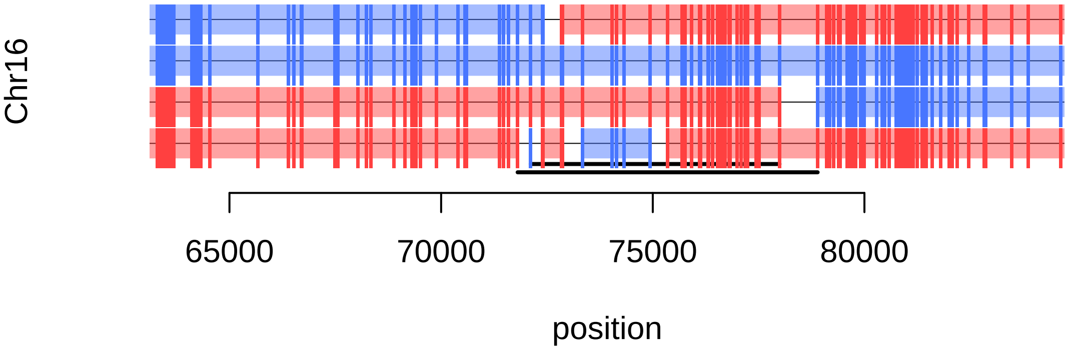

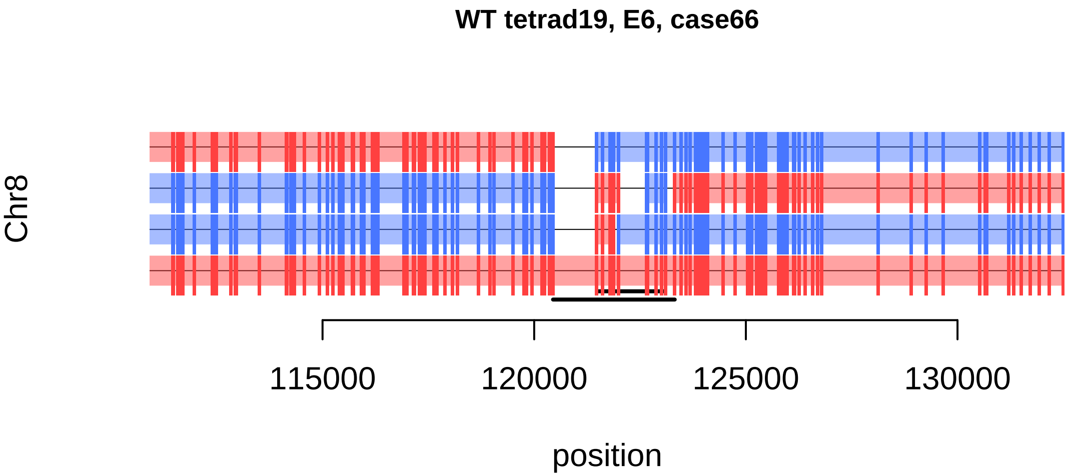
WT tetrad19, E6, case66
Chr8
115000
120000
125000
130000
position

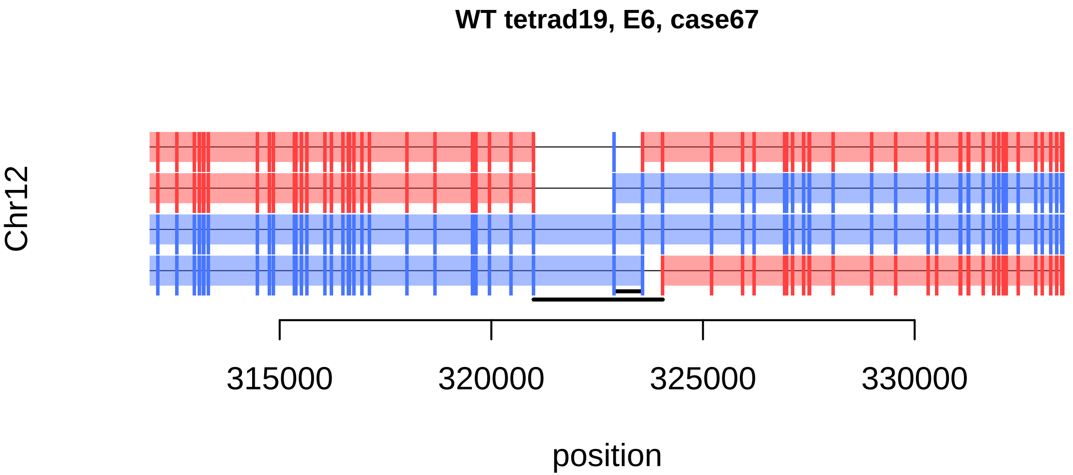
WT tetrad19, E6, case67
Chr12
315000
320000
325000
330000
position

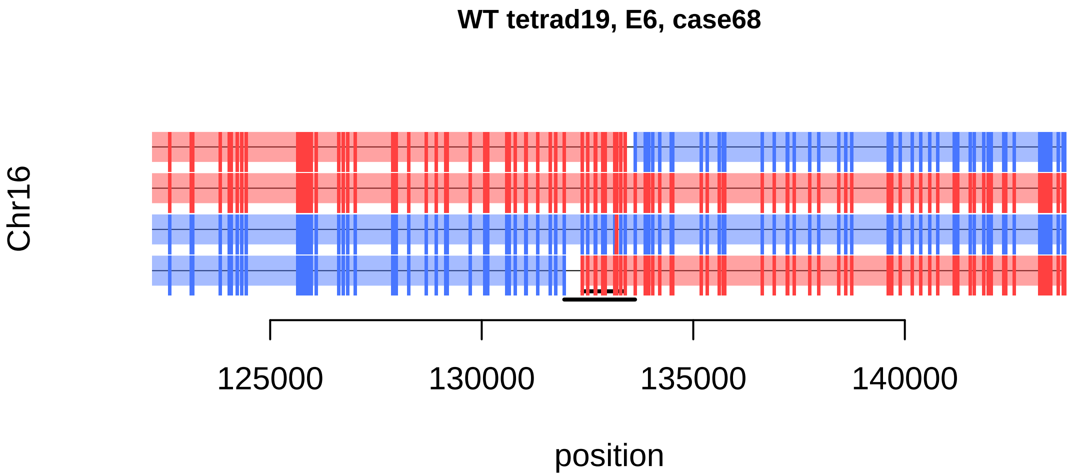
WT tetrad19, E6, case68
Chr16
125000
130000
135000
140000
position

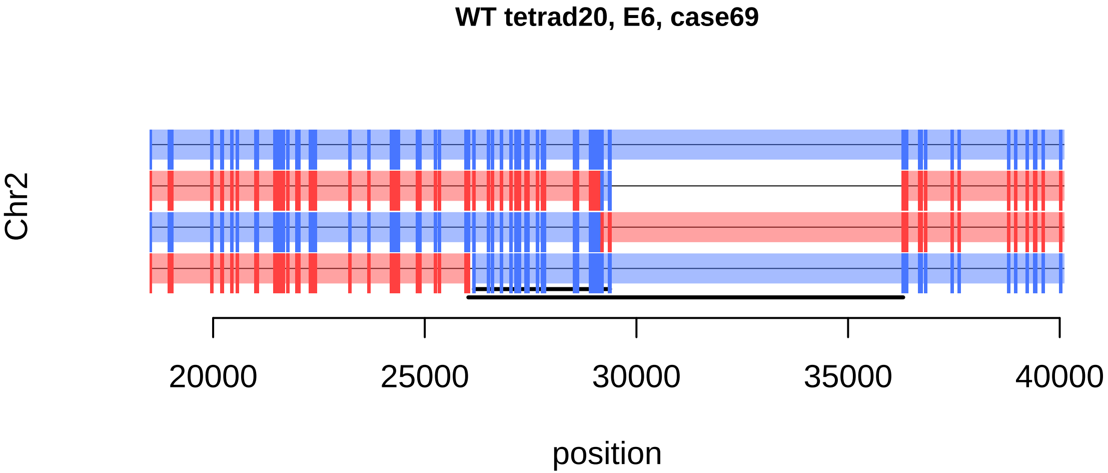
WT tetrad20, E6, case69
Chr2
20000
25000
30000
35000
40000
position

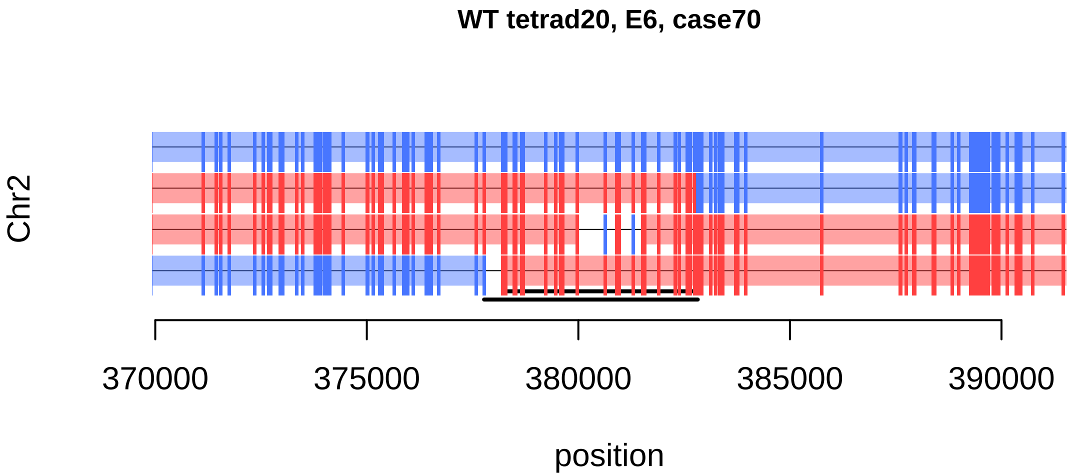

WT tetrad20, E6, case70
Chr2
370000
375000
380000
385000
390000
position

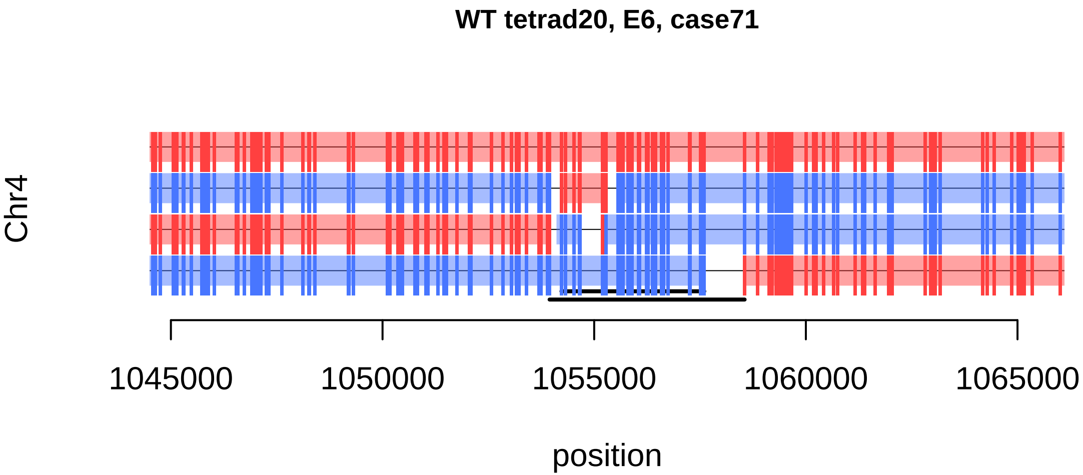

WT tetrad20, E6, case71
Chr4
1045000
1050000
1055000
1060000
1065000
position

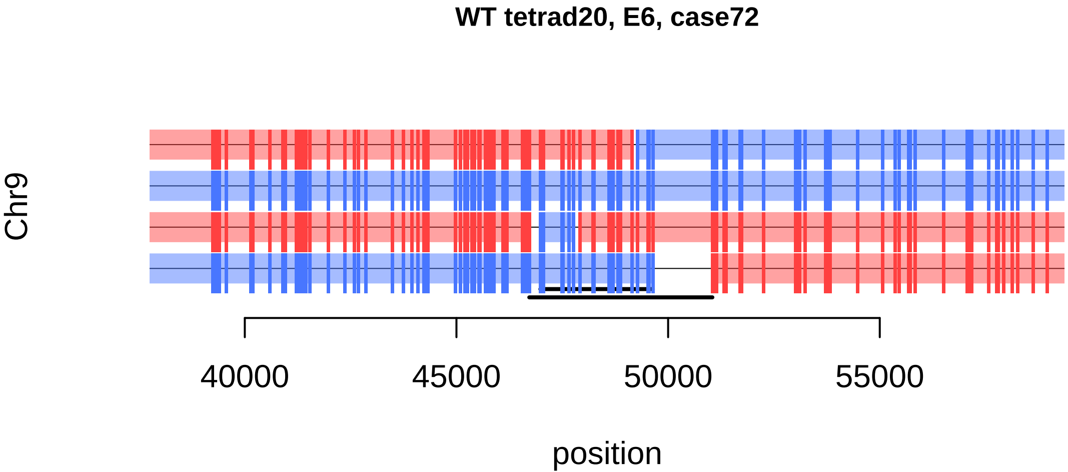

WT tetrad20, E6, case72
Chr9
40000
45000
50000
55000
position

WT tetrad20, E6, case73

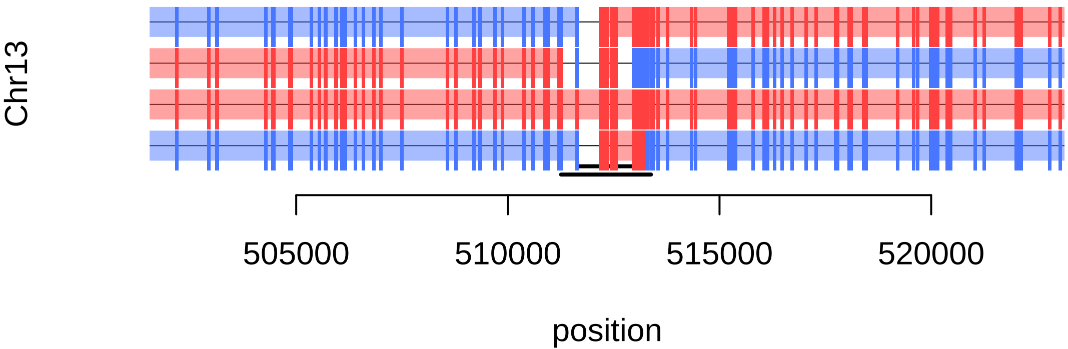

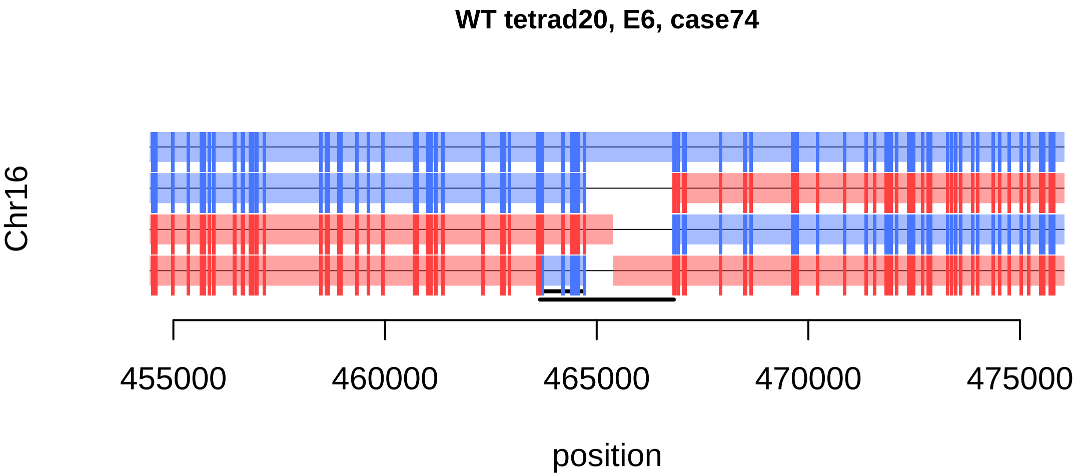
WT tetrad20, E6, case74
Chr16
455000
460000
465000
470000
475000
position

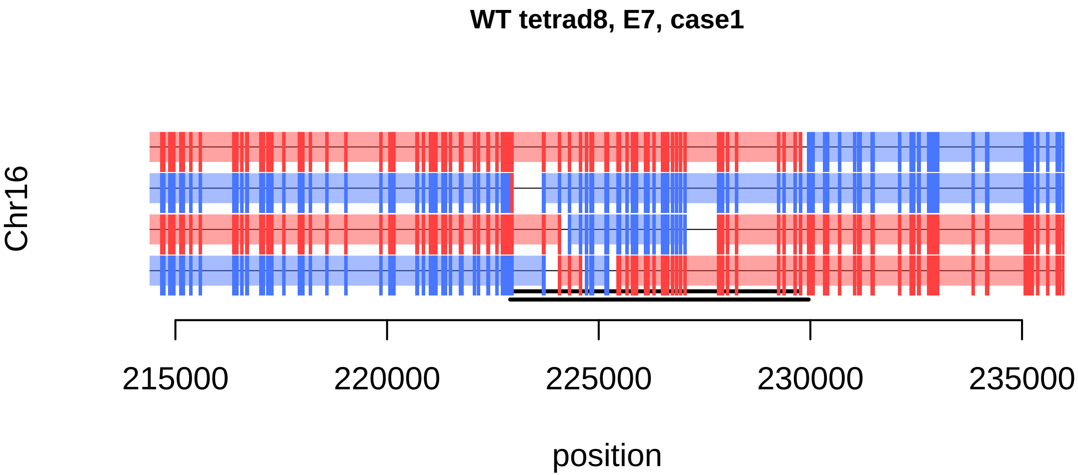
WT tetrad8, E7, case1
Chr16
215000
220000
225000
230000
235000
position

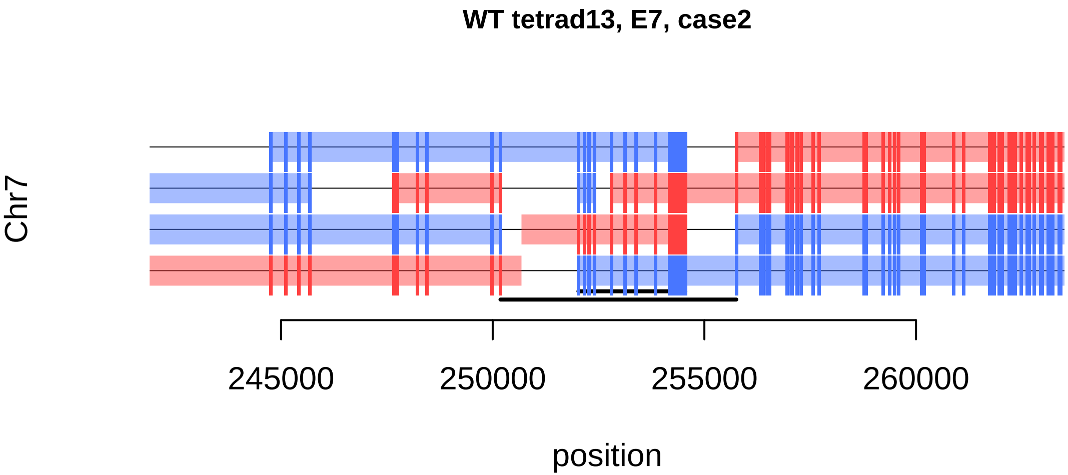
WT tetrad13, E7, case2
Chr7
245000
250000
255000
260000
position

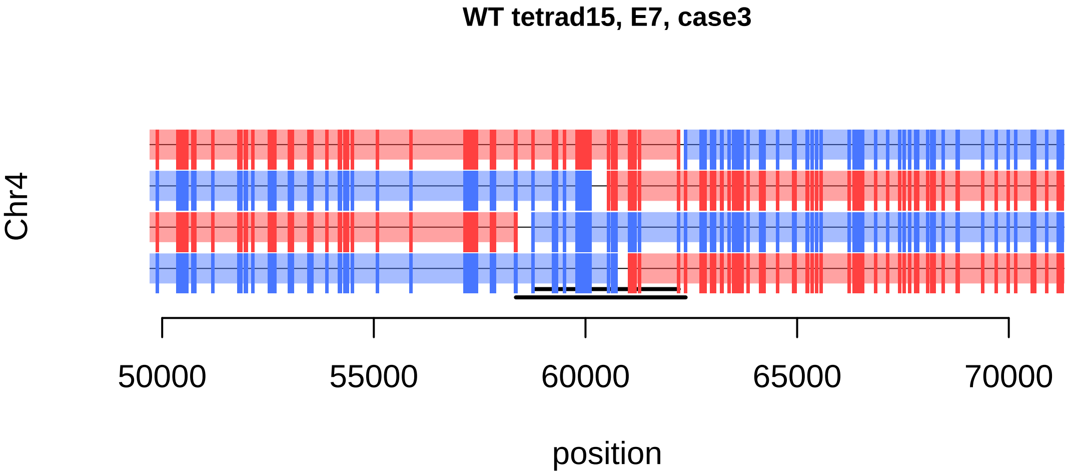

WT tetrad15, E7, case3
Chr4
50000
55000
60000
65000
70000
position
