# Supplementary material for: Mek1 Down Regulates Rad51 Activity during Yeast Meiosis by Phosphorylation of Hed1
Source: PLoS Genet. 2016 Aug 2;12(8):e1006226. doi: 10.1371/journal.pgen.1006226 (PMC4970670; doi:10.1371/journal.pgen.1006226)

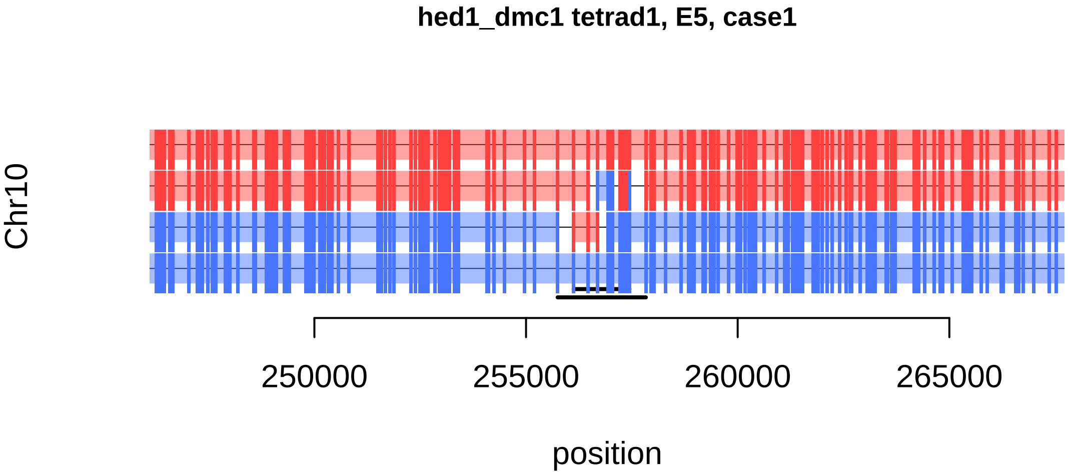
hed1_dmc1 tetrad1, E5, case1
Chr10
250000
255000
260000
265000
position

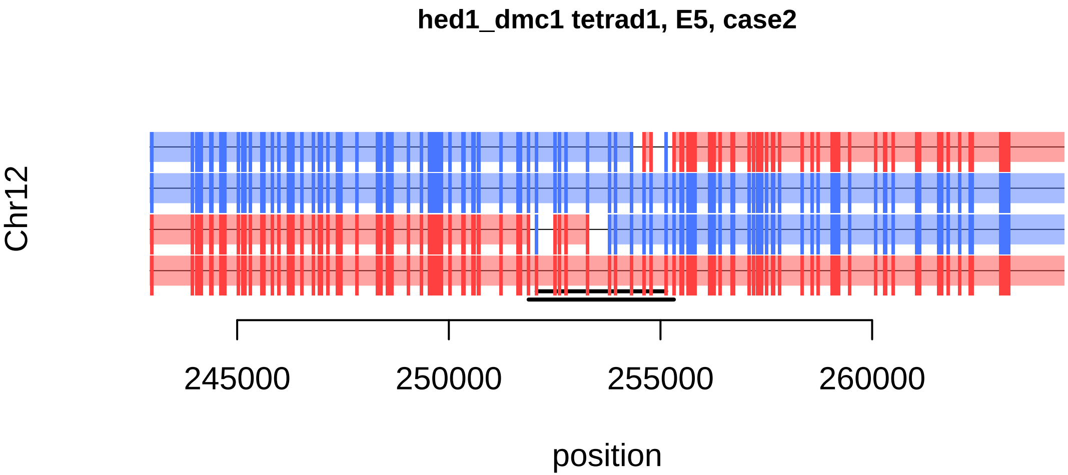
hed1_dmc1 tetrad1, E5, case2
Chr12
245000
250000
255000
260000
position

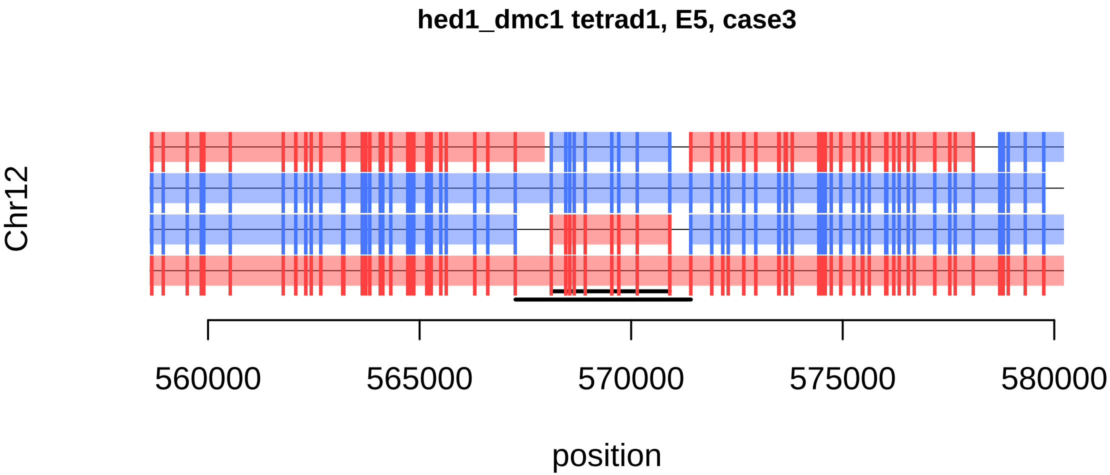
hed1_dmc1 tetrad1, E5, case3
Chr12
560000
565000
570000
575000
580000
position

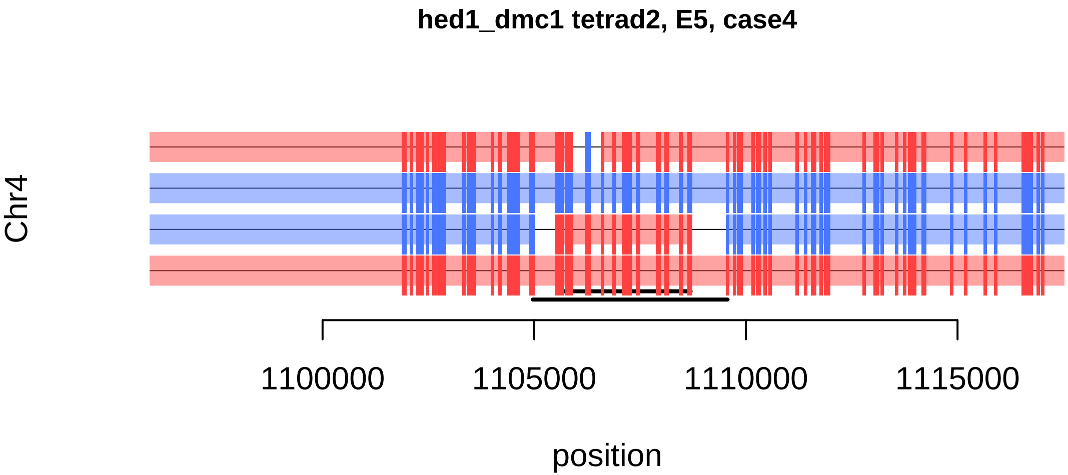
hed1_dmc1 tetrad2, E5, case4
Chr4
1100000
1105000
1110000
1115000
position

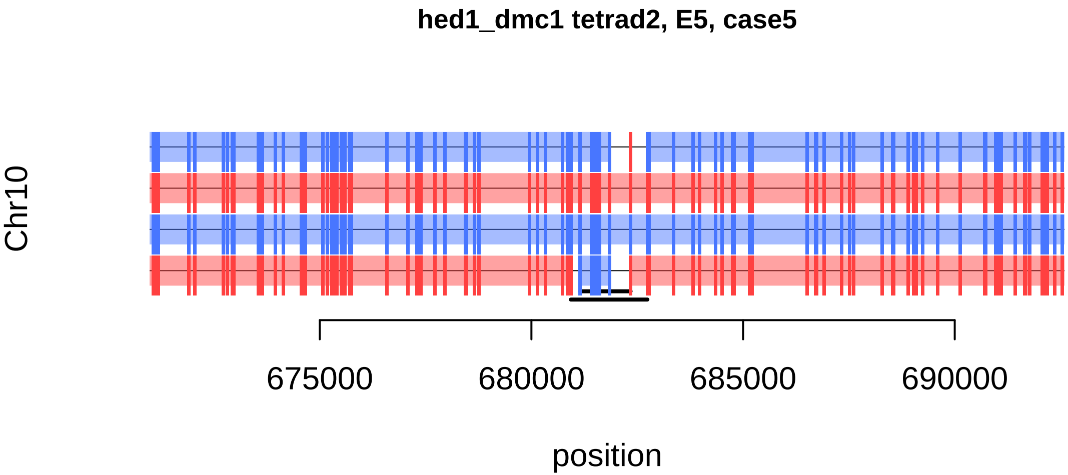
hed1_dmc1 tetrad2, E5, case5
Chr10
675000
680000
685000
690000
position

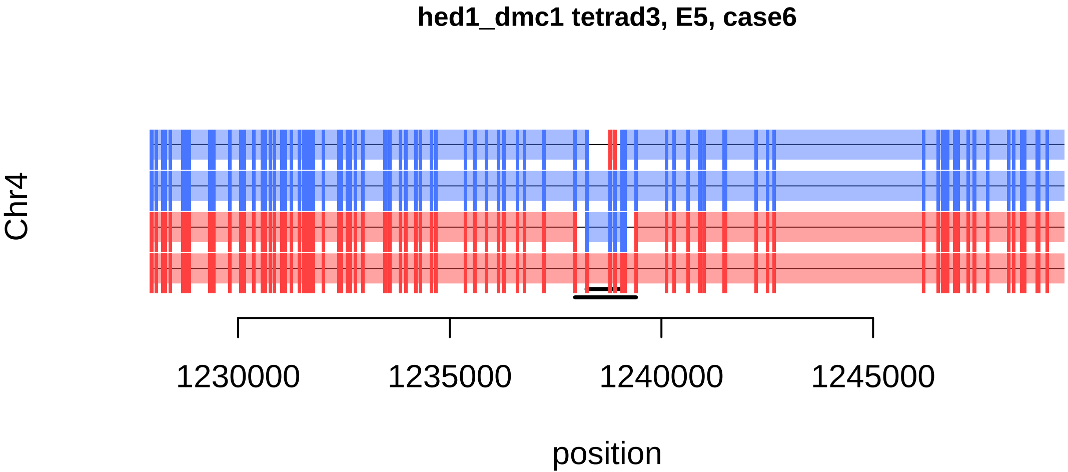
hed1_dmc1 tetrad3, E5, case6
Chr4
1230000
1235000
1240000
1245000
position

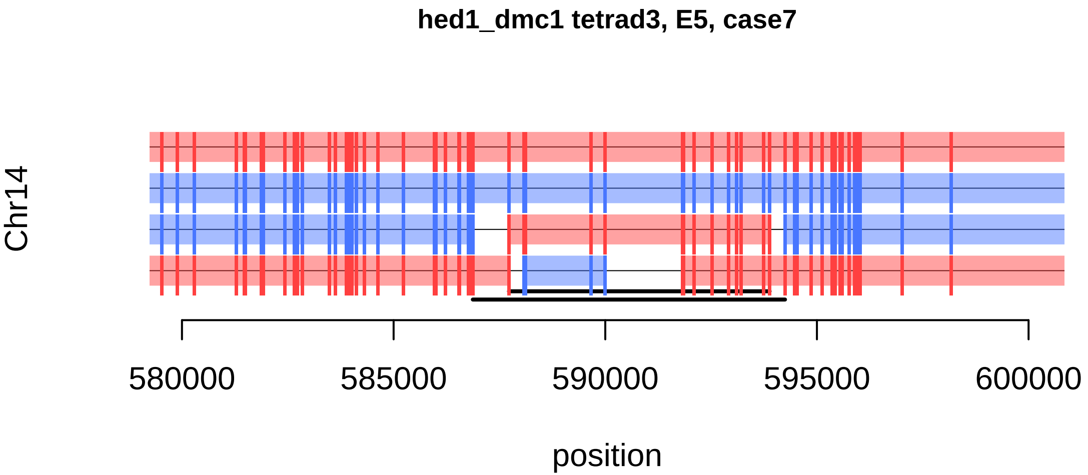
hed1_dmc1 tetrad3, E5, case7
Chr14
580000
585000
590000
595000
600000
position

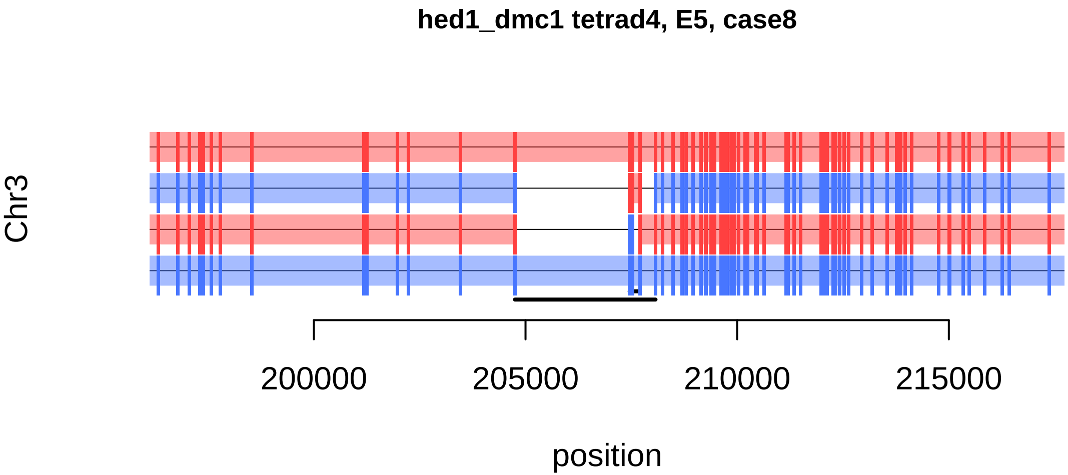
hed1_dmc1 tetrad4, E5, case8
Chr3
200000
205000
210000
215000
position

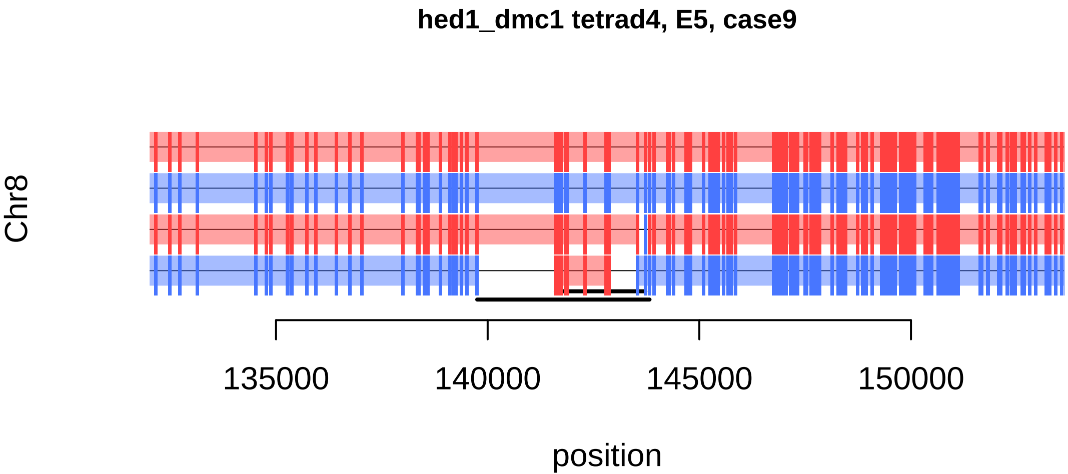
hed1_dmc1 tetrad4, E5, case9
Chr8
135000
140000
145000
150000
position

hed1_dmc1 tetrad4, E5, case10

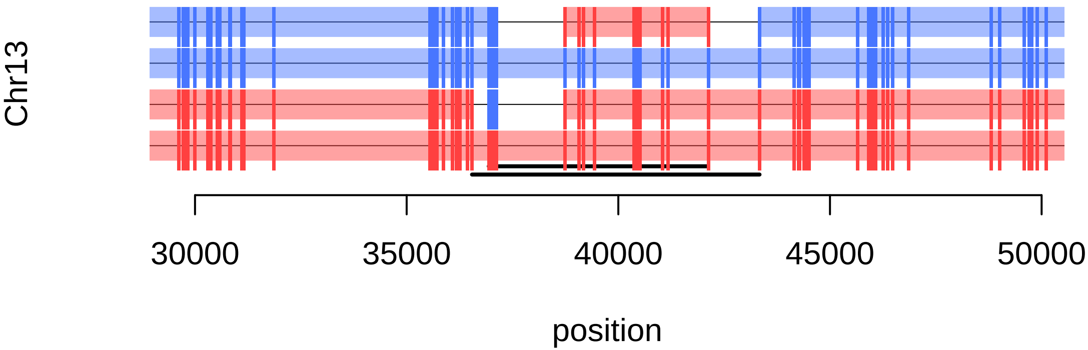

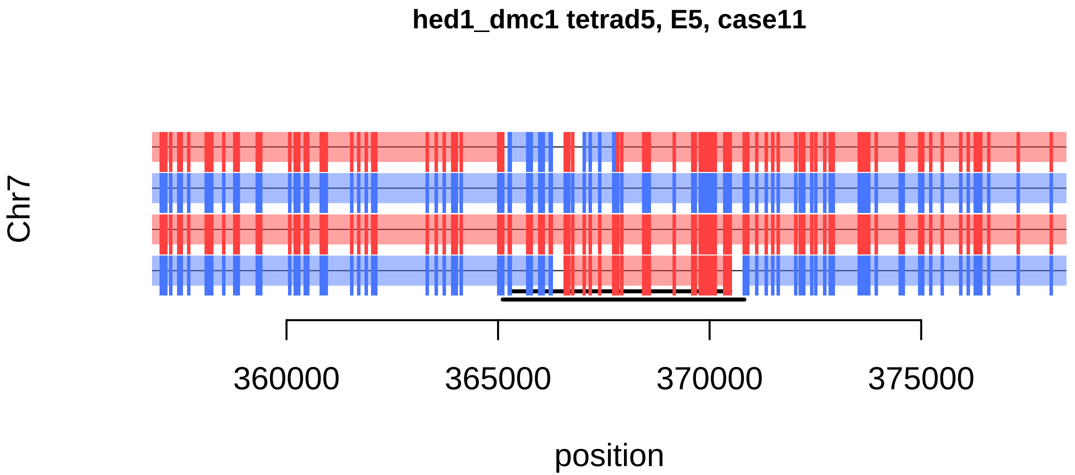
hed1_dmc1 tetrad5, E5, case11
Chr7
360000
365000
370000
375000
position

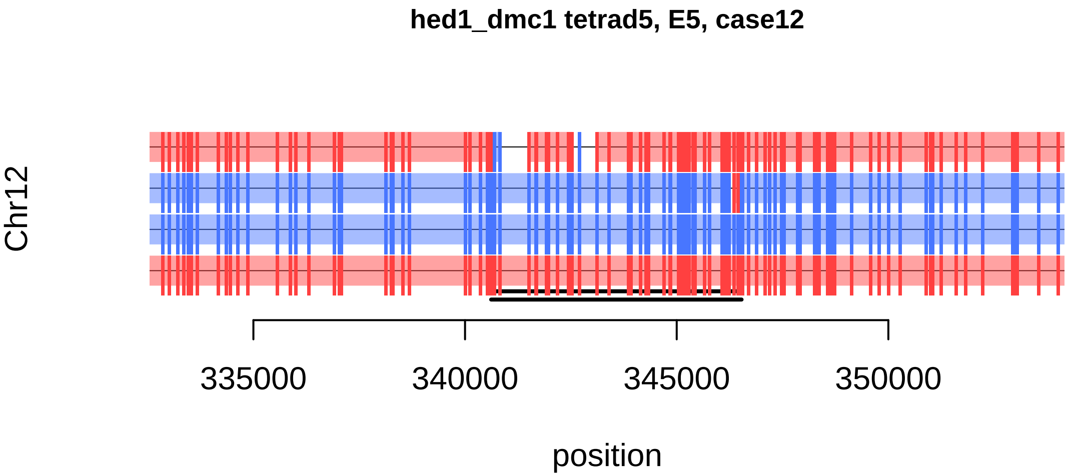
hed1_dmc1 tetrad5, E5, case12
Chr12
335000
340000
345000
350000
position

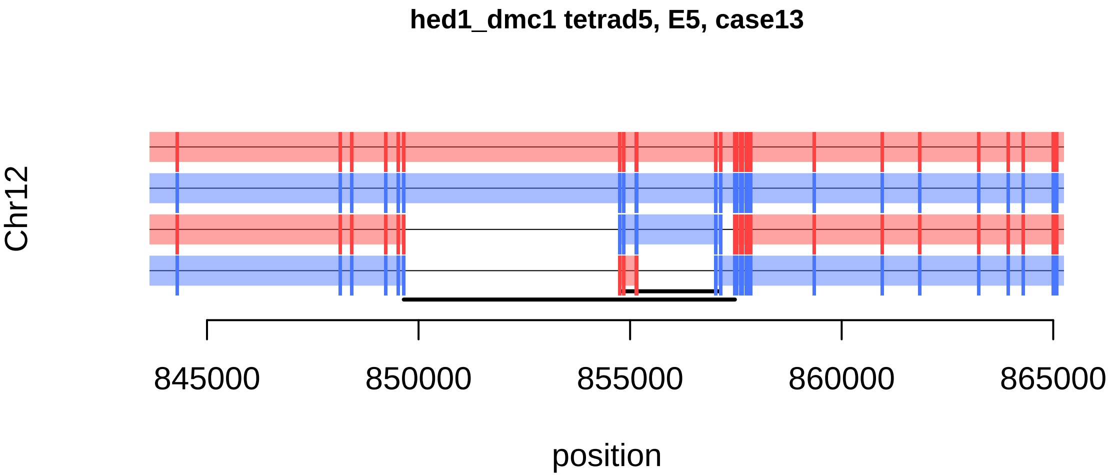

hed1_dmc1 tetrad5, E5, case13
Chr12
845000
850000
855000
860000
865000
position

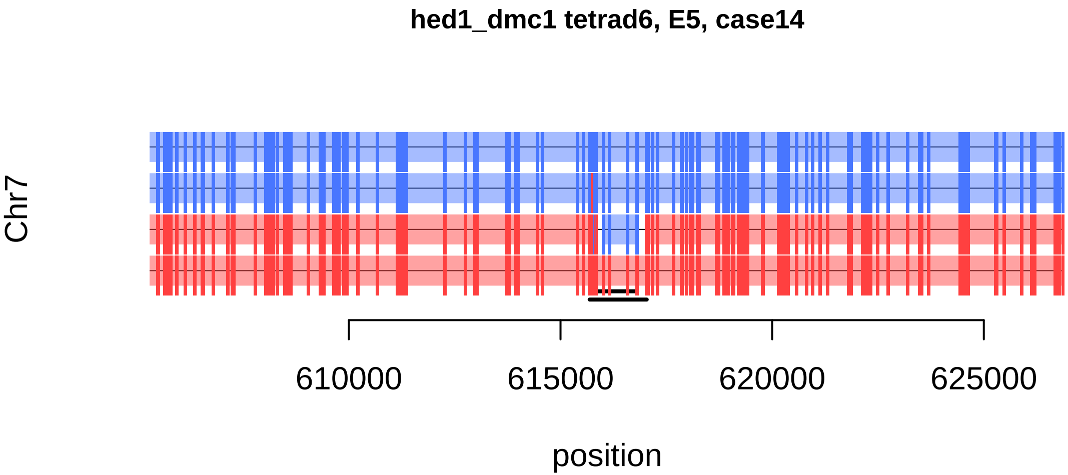

hed1_dmc1 tetrad6, E5, case14
Chr7
610000
615000
620000
625000
position

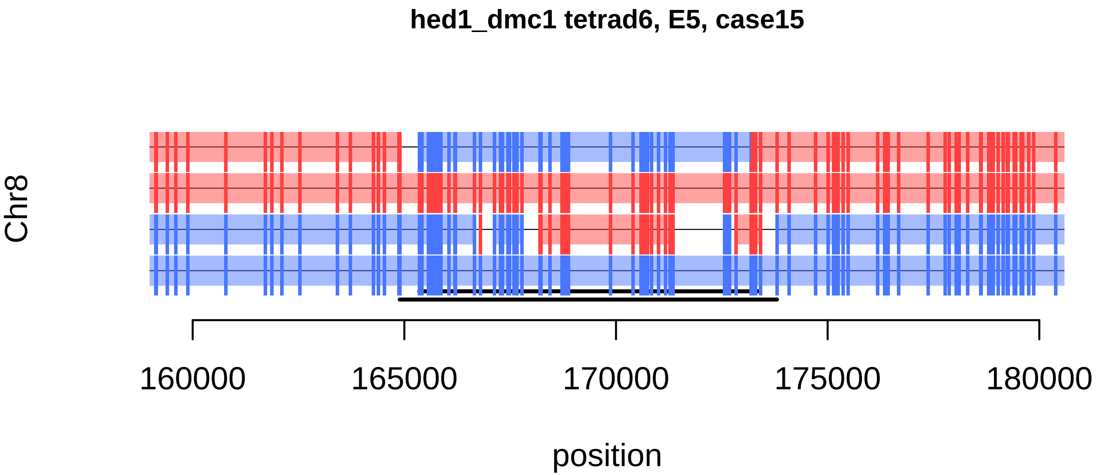
hed1_dmc1 tetrad6, E5, case15
Chr8
160000
165000
170000
175000
180000
position

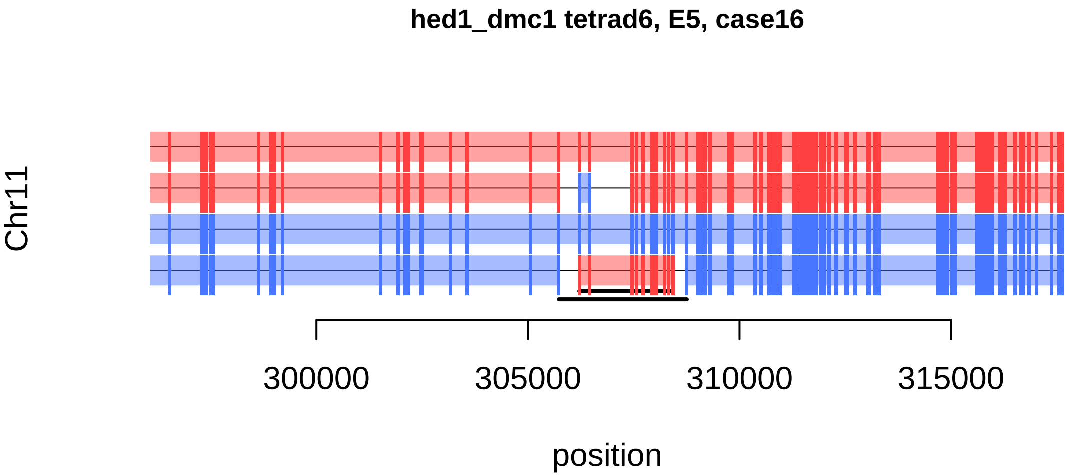
hed1_dmc1 tetrad6, E5, case16
Chr11
300000
305000
310000
315000
position

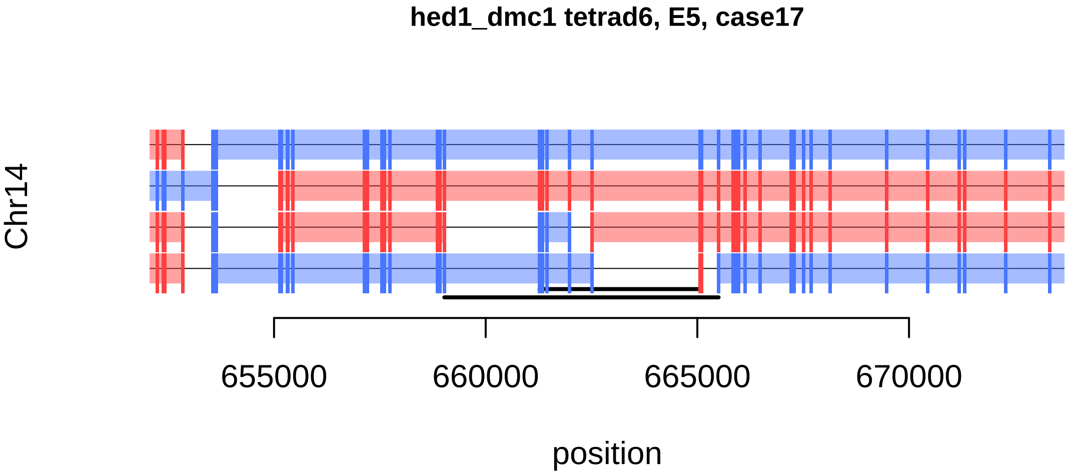
hed1_dmc1 tetrad6, E5, case17
Chr14
655000
660000
665000
670000
position

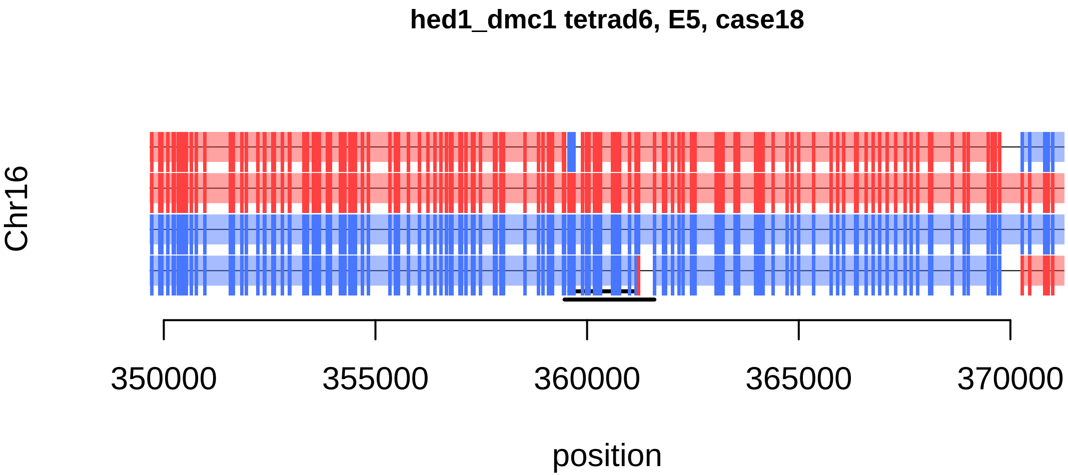
hed1_dmc1 tetrad6, E5, case18
Chr16
350000
355000
360000
365000
370000
position

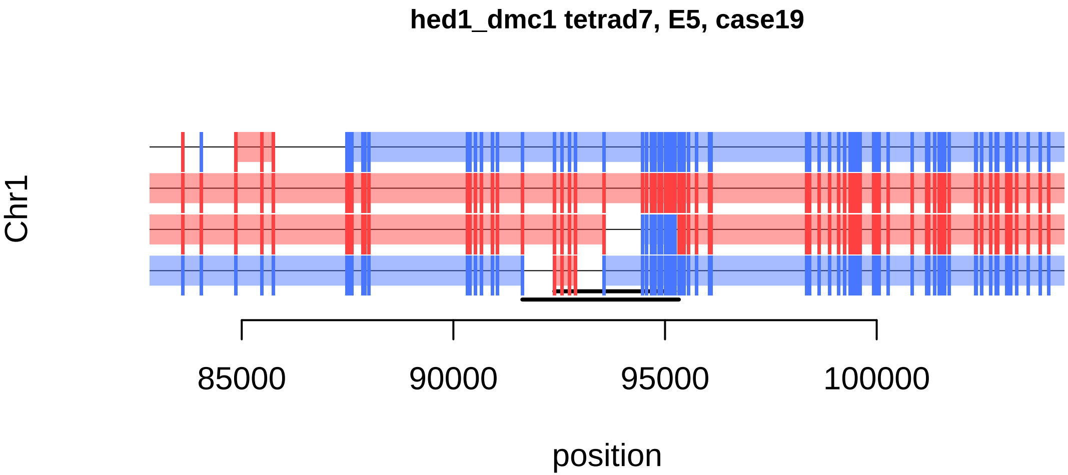
hed1_dmc1 tetrad7, E5, case19
Chr1
85000
90000
95000
100000
position

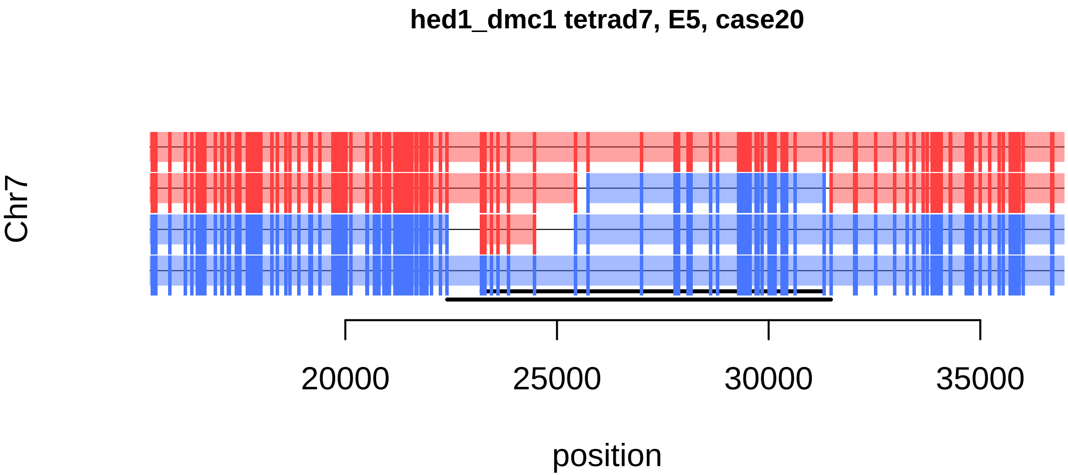
hed1_dmc1 tetrad7, E5, case20
Chr7
20000
25000
30000
35000
position

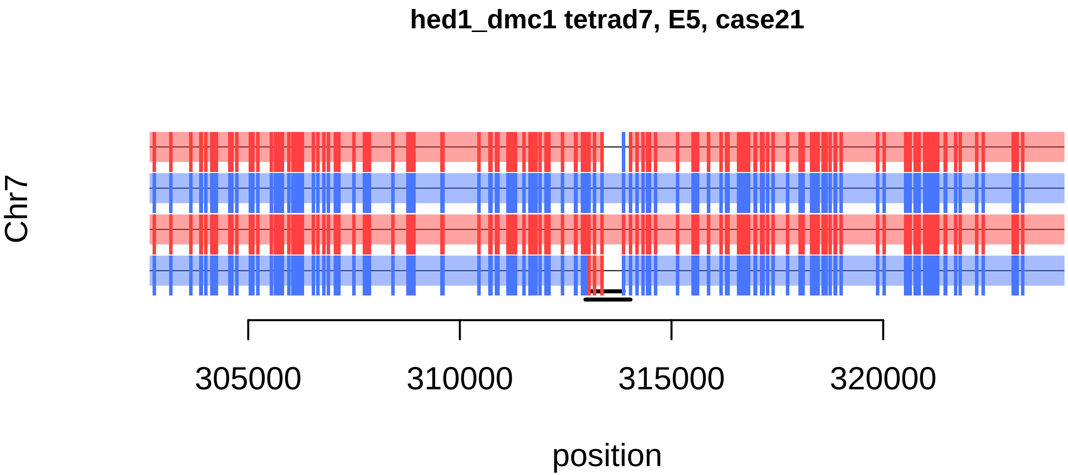
hed1_dmc1 tetrad7, E5, case21
Chr7
305000
310000
315000
320000
position

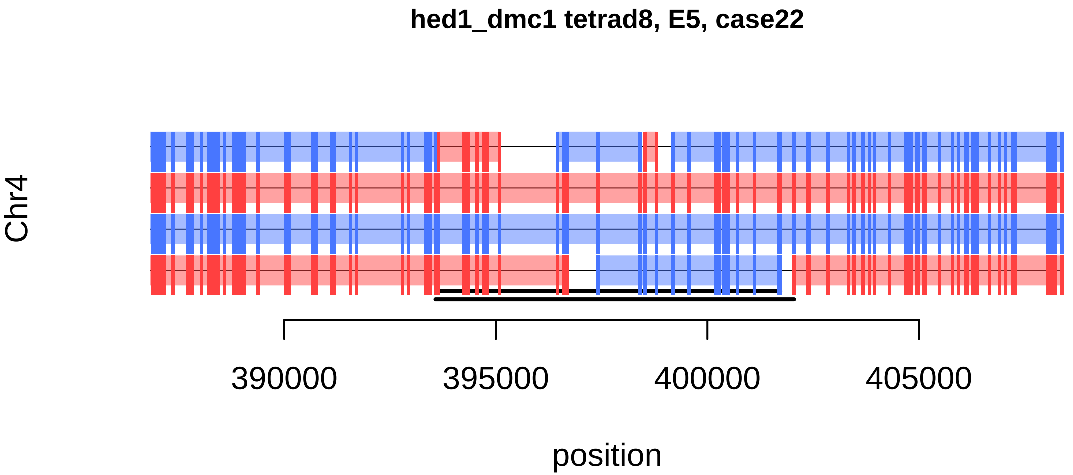
hed1_dmc1 tetrad8, E5, case22
Chr4
390000
395000
400000
405000
position

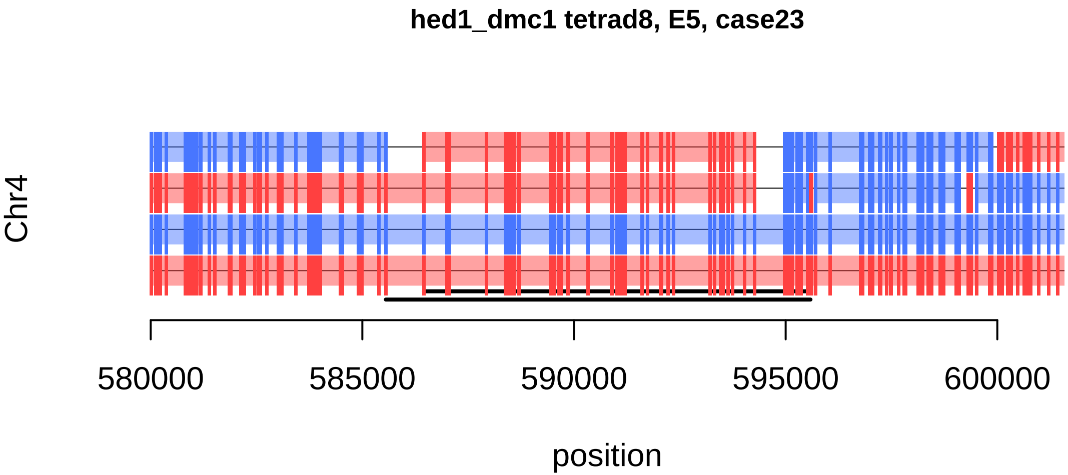
hed1_dmc1 tetrad8, E5, case23
Chr4
580000
585000
590000
595000
600000
position

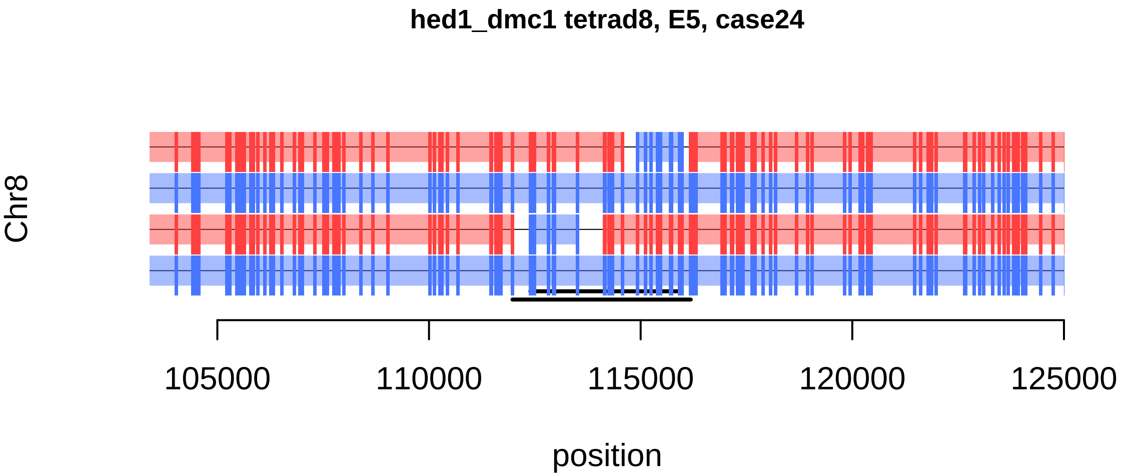
hed1_dmc1 tetrad8, E5, case24
Chr8
105000
110000
115000
120000
125000
position

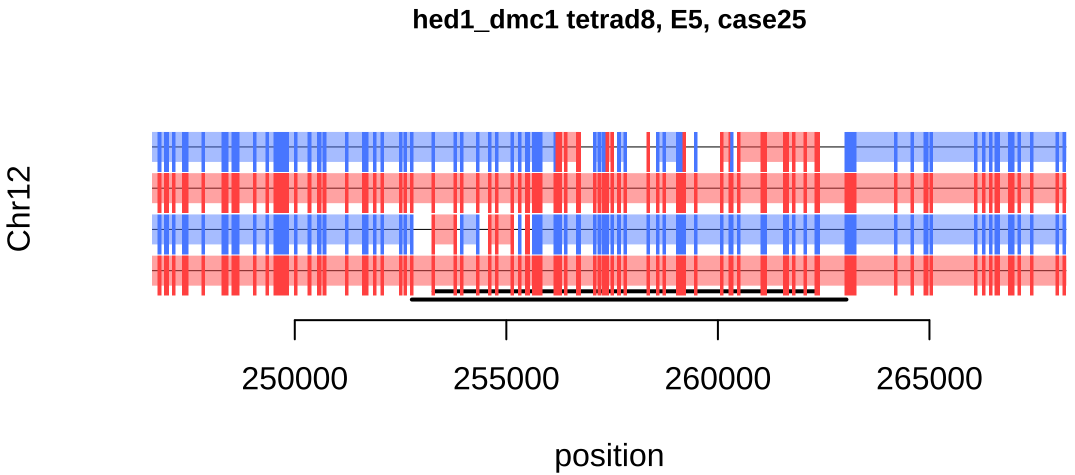
hed1_dmc1 tetrad8, E5, case25
Chr12
250000
255000
260000
265000
position

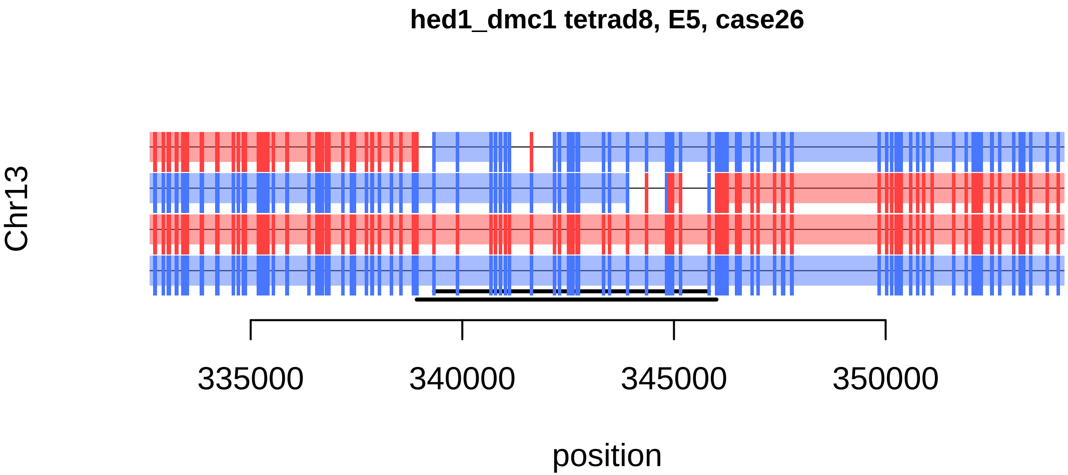

hed1_dmc1 tetrad8, E5, case26
Chr13
335000
340000
345000
350000
position

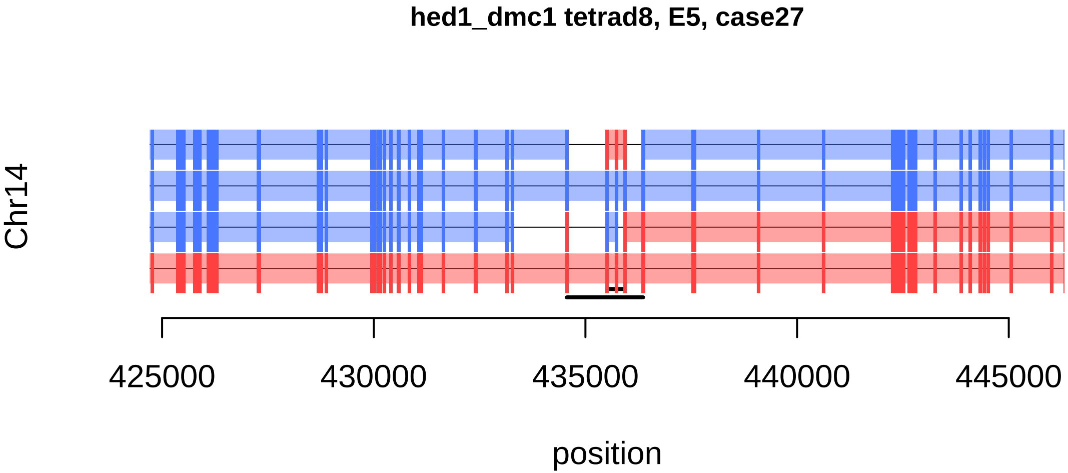
hed1_dmc1 tetrad8, E5, case27
Chr14
425000
430000
435000
440000
445000
position

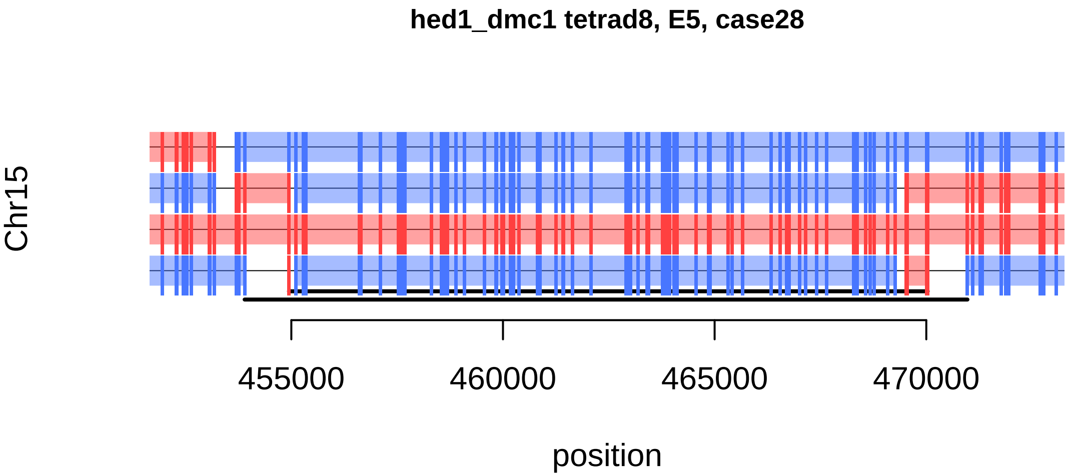
hed1_dmc1 tetrad8, E5, case28
Chr15
455000
460000
465000
470000
position

hed1_dmc1 tetrad9, E5, case29

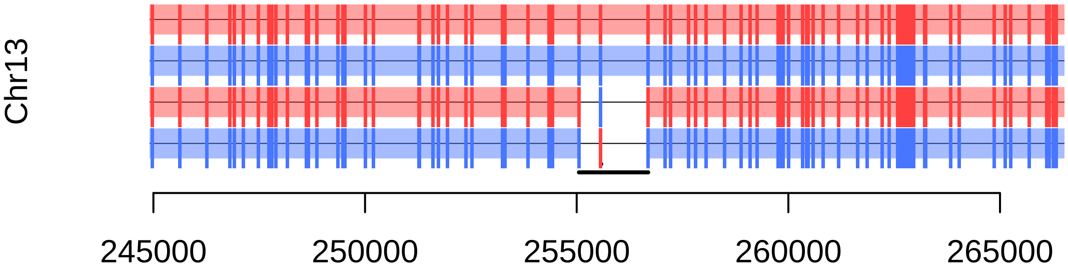

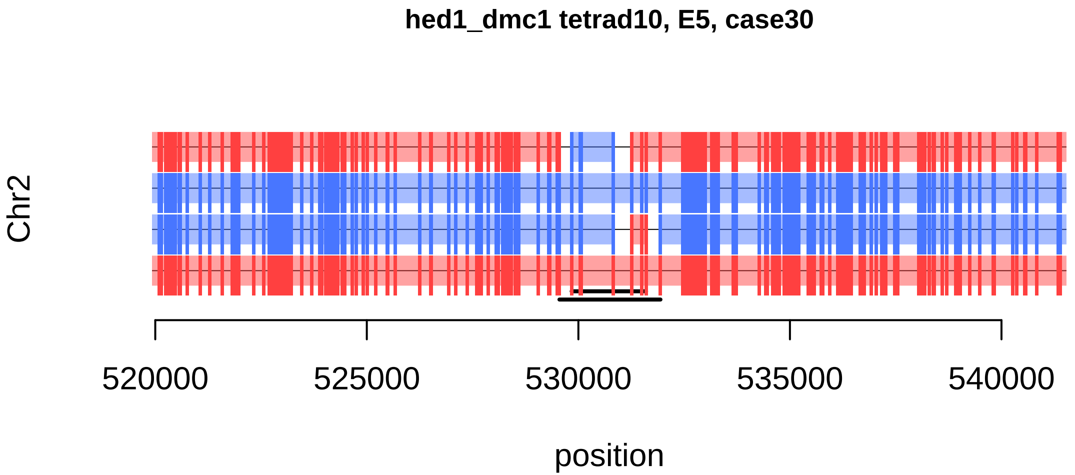
hed1_dmc1 tetrad10, E5, case30
Chr2
520000
525000
530000
535000
540000
position

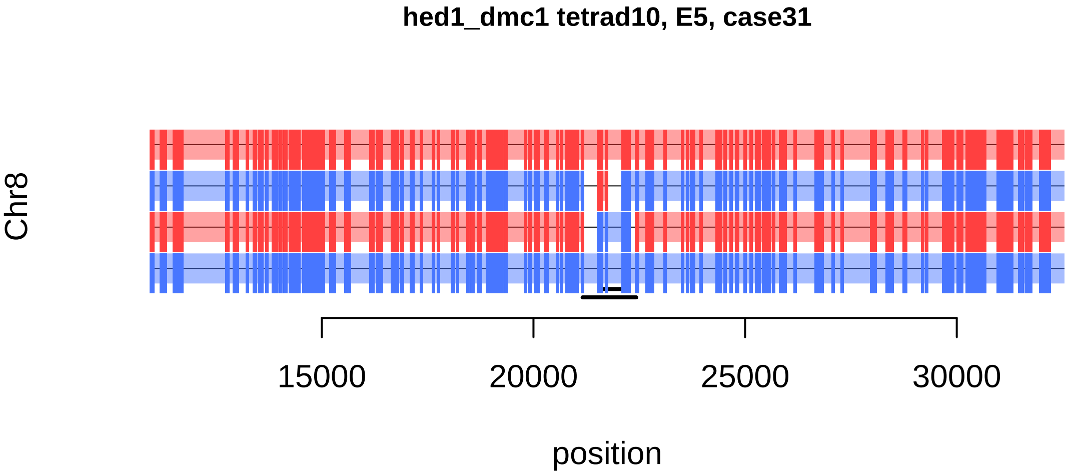
hed1_dmc1 tetrad10, E5, case31
Chr8
15000
20000
25000
30000
position

hed1_dmc1 tetrad11, E5, case32

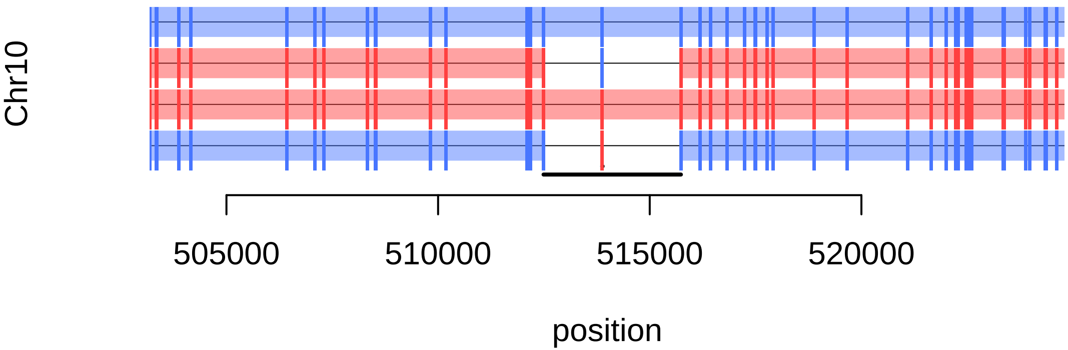

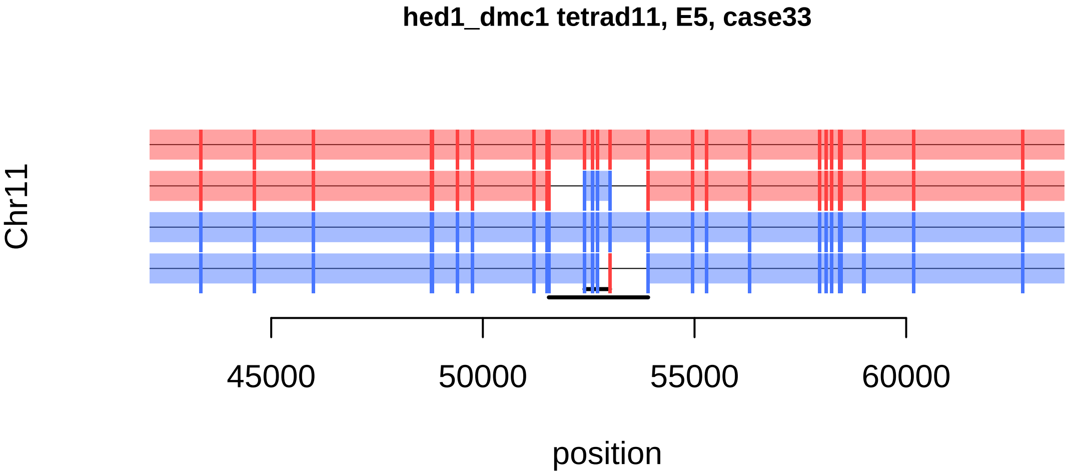
hed1_dmc1 tetrad11, E5, case33
Chr11
45000
50000
55000
60000
position

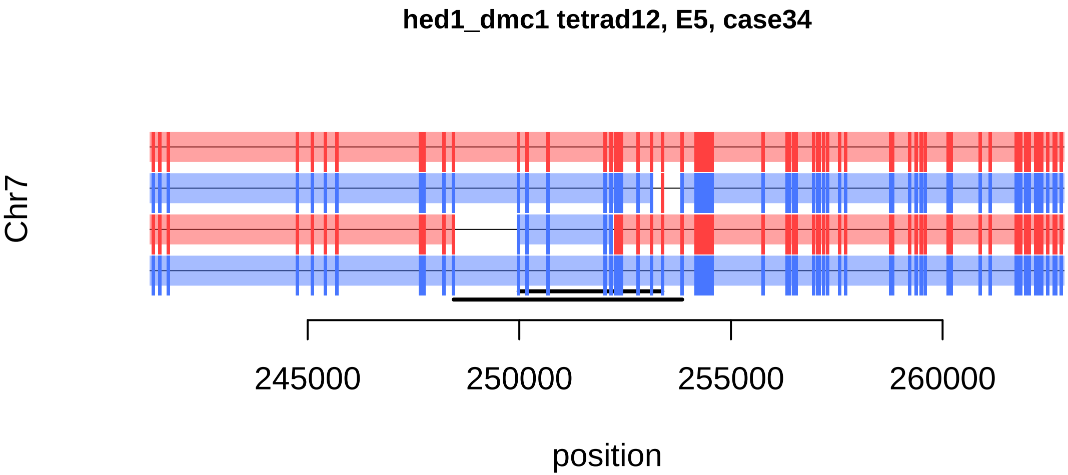
hed1_dmc1 tetrad12, E5, case34
Chr7
245000
250000
255000
260000
position

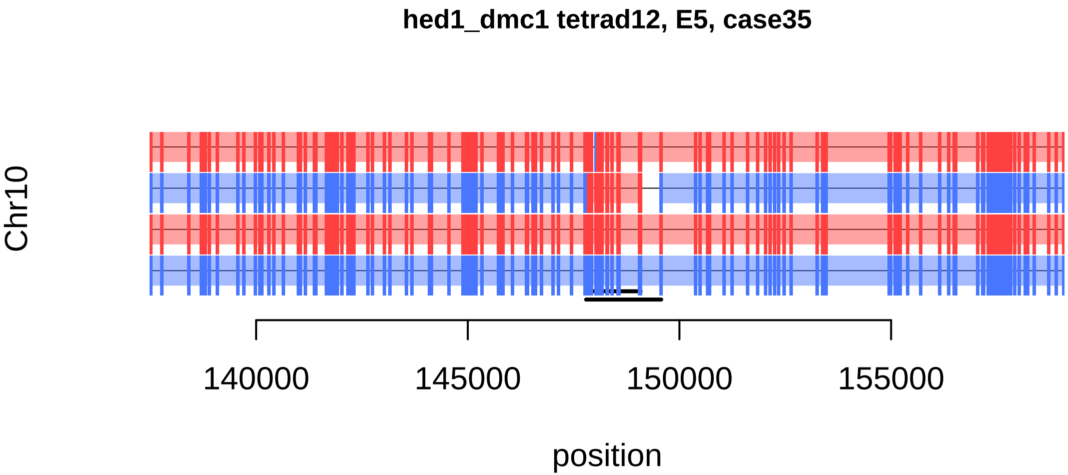
hed1_dmc1 tetrad12, E5, case35
Chr10
140000
145000
150000
155000
position

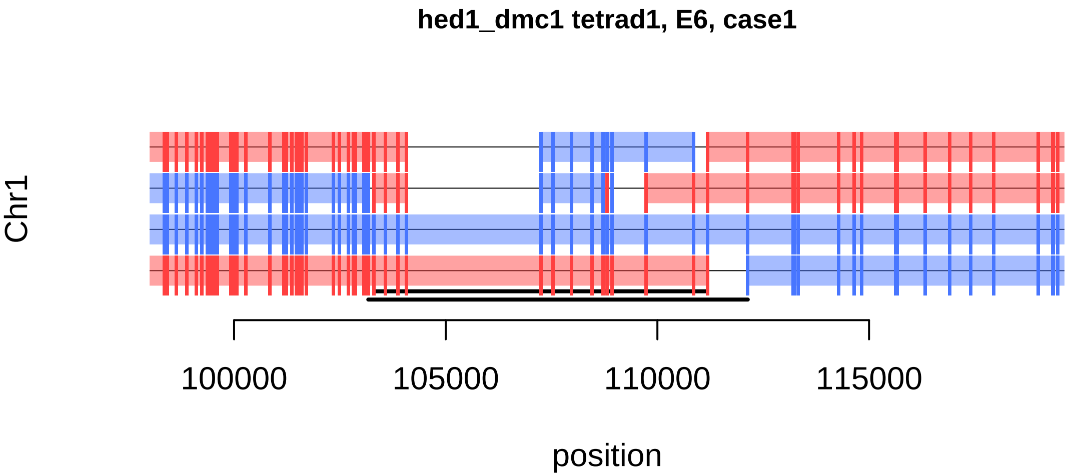

hed1_dmc1 tetrad1, E6, case1
Chr1
100000
105000
110000
115000
position

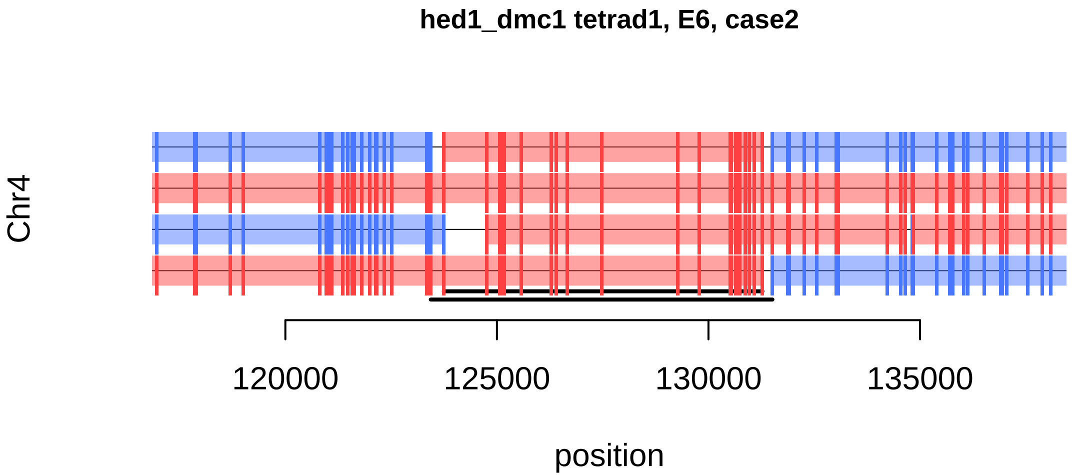
hed1_dmc1 tetrad1, E6, case2
Chr4
120000
125000
130000
135000
position

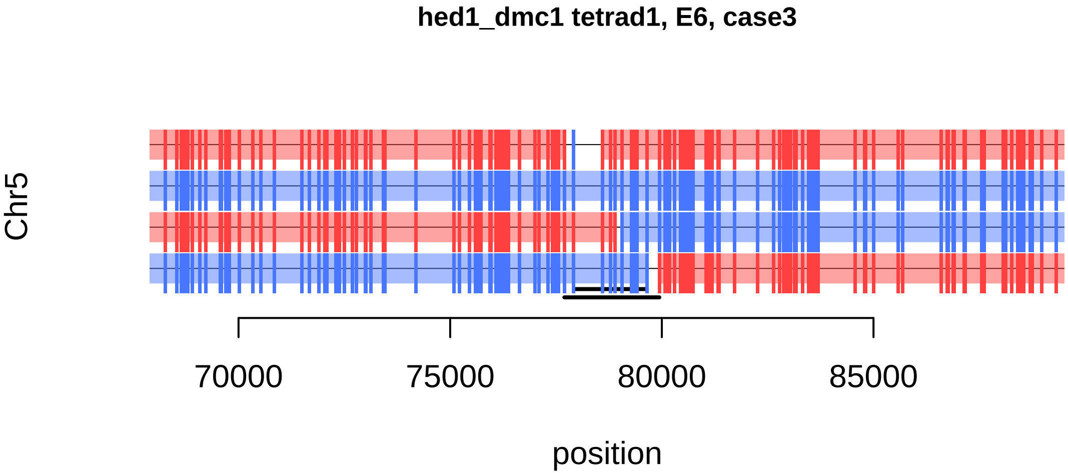

hed1_dmc1 tetrad1, E6, case3
Chr5
70000
75000
80000
85000
position

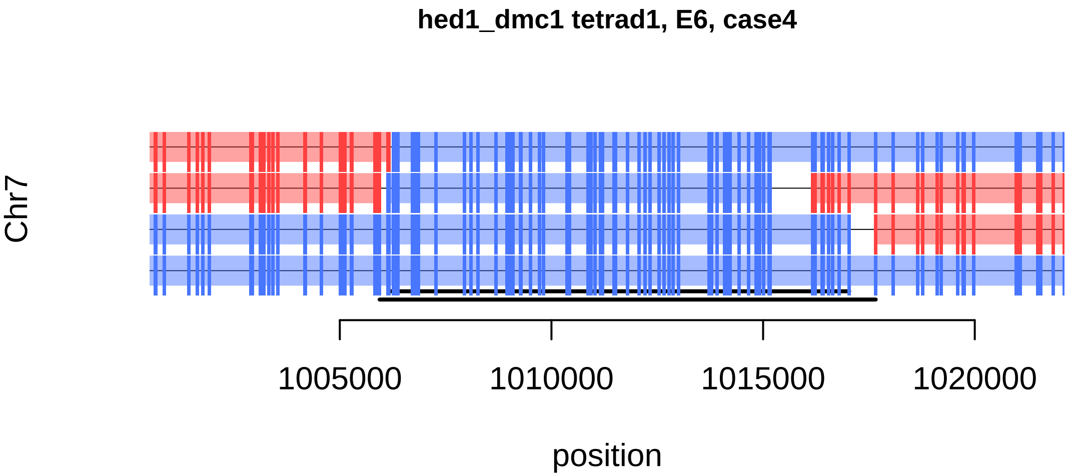
hed1_dmc1 tetrad1, E6, case4
Chr7
1005000
1010000
1015000
1020000
position

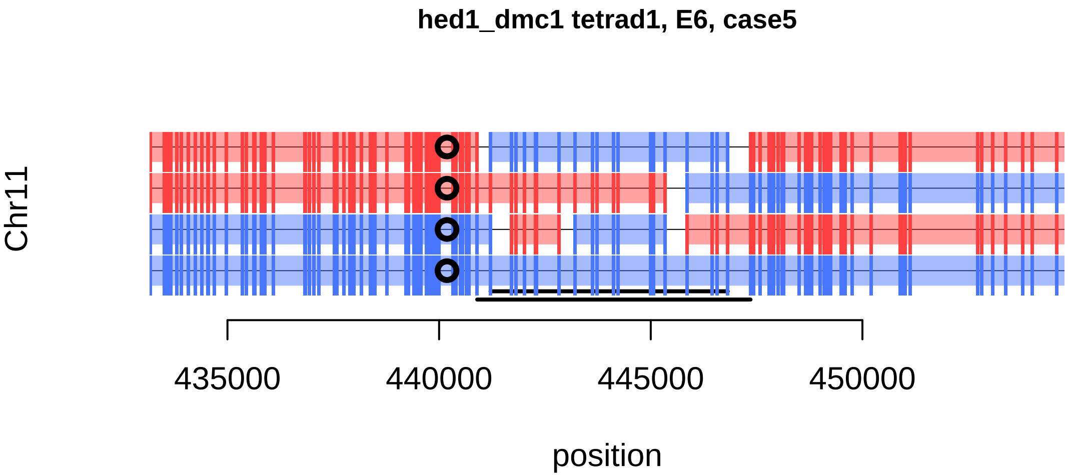
hed1_dmc1 tetrad1, E6, case5
Chr11
435000
440000
445000
450000
position

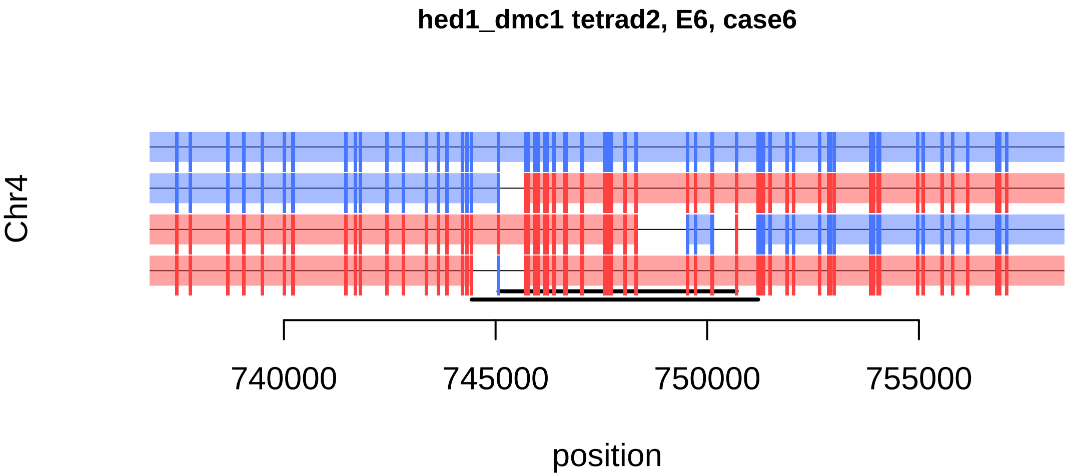
hed1_dmc1 tetrad2, E6, case6
Chr4
740000
745000
750000
755000
position

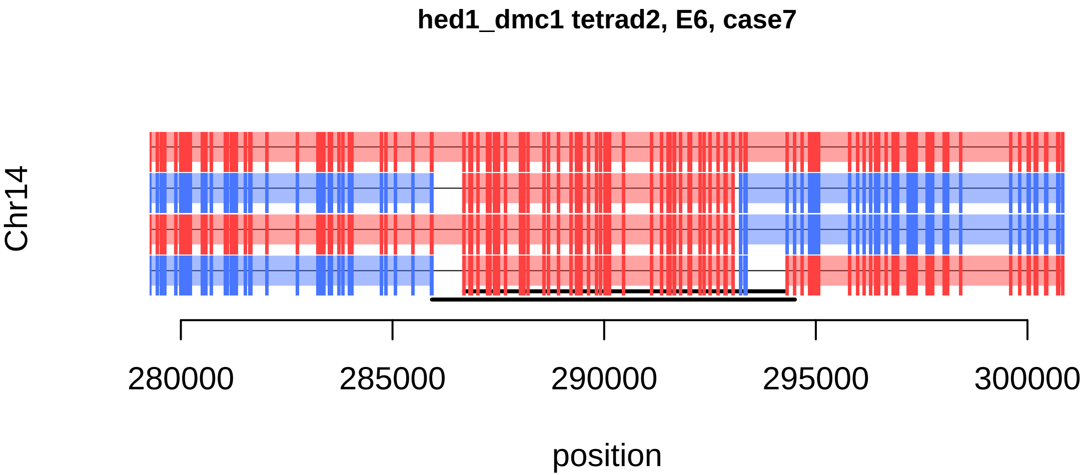
hed1_dmc1 tetrad2, E6, case7
Chr14
280000
285000
290000
295000
300000
position

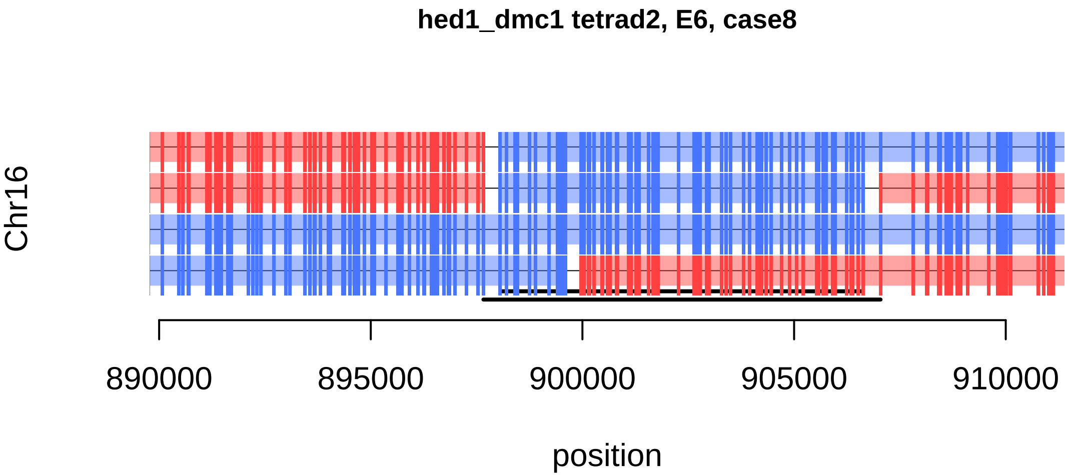
hed1_dmc1 tetrad2, E6, case8
Chr16
890000
895000
900000
905000
910000
position

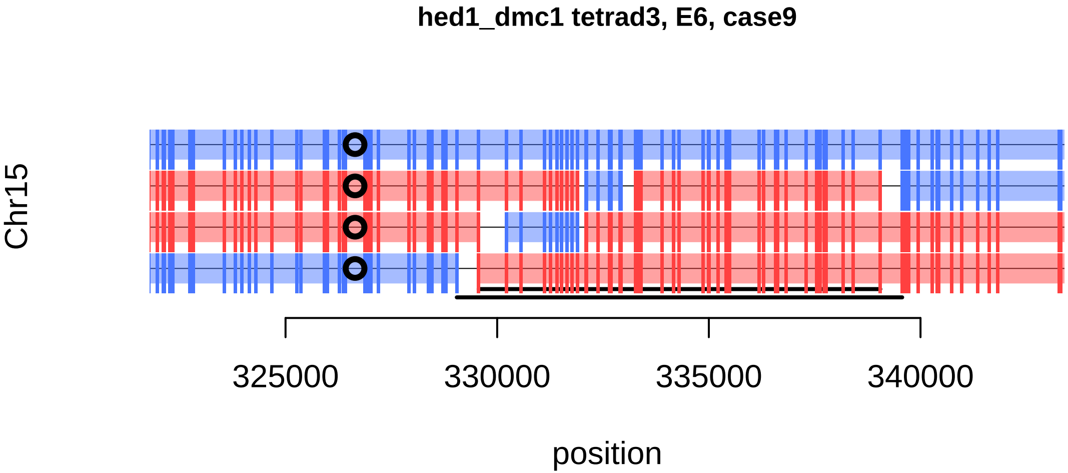

hed1_dmc1 tetrad3, E6, case9
Chr15
325000
330000
335000
340000
position

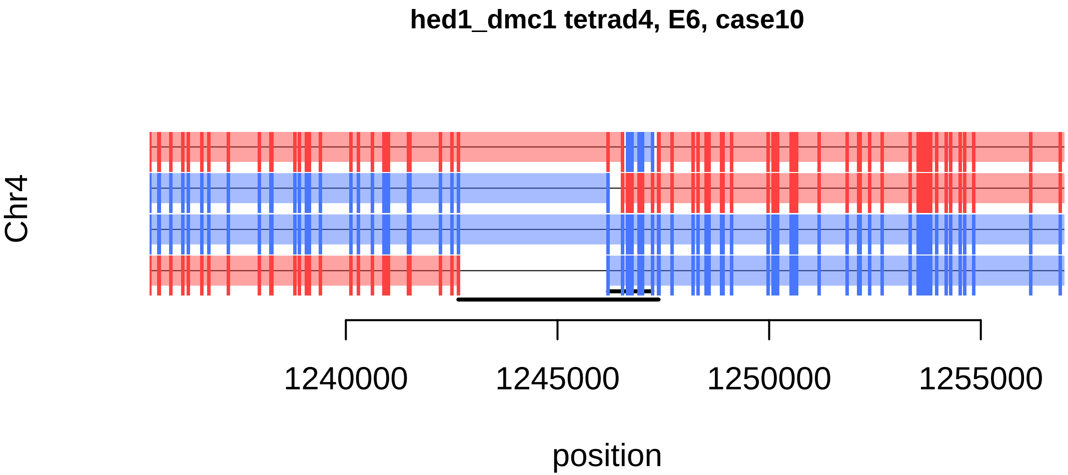
hed1_dmc1 tetrad4, E6, case10
Chr4
1240000
1245000
1250000
1255000
position

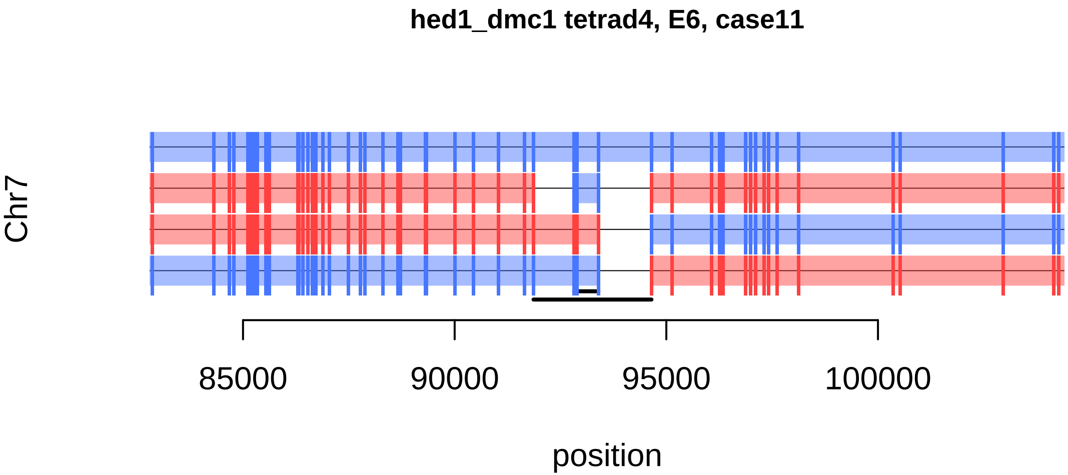

hed1_dmc1 tetrad4, E6, case11
Chr7
85000
90000
95000
100000
position

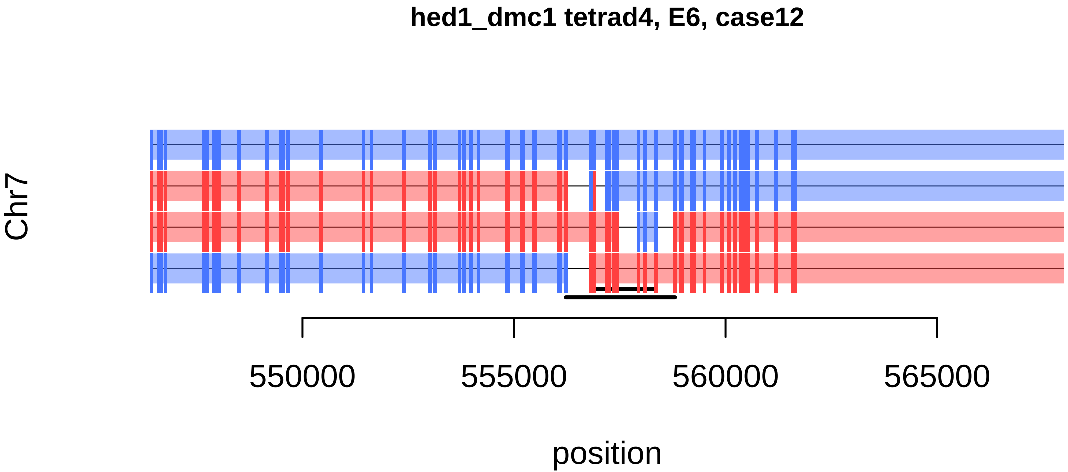
hed1_dmc1 tetrad4, E6, case12
Chr7
550000
555000
560000
565000
position

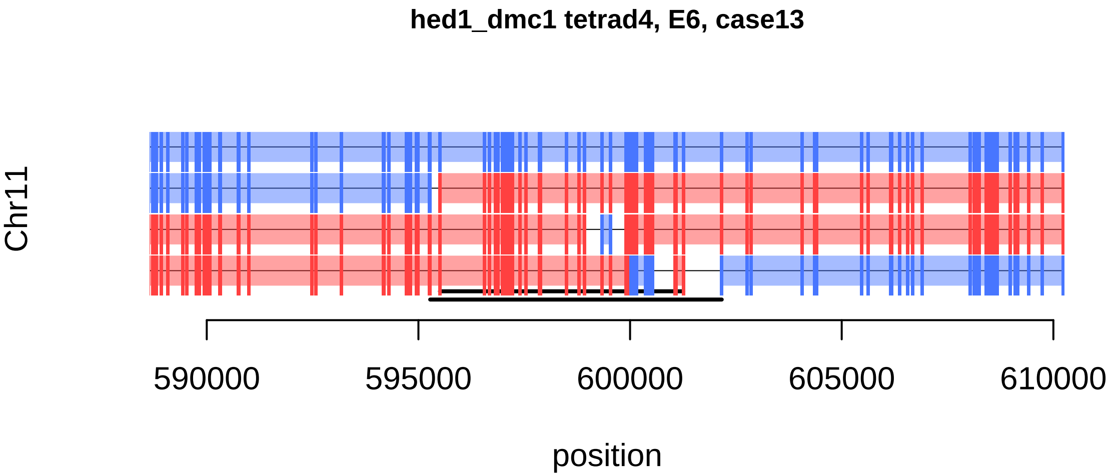

hed1_dmc1 tetrad4, E6, case13
Chr11
590000
595000
600000
605000
610000
position

hed1_dmc1 tetrad4, E6, case14

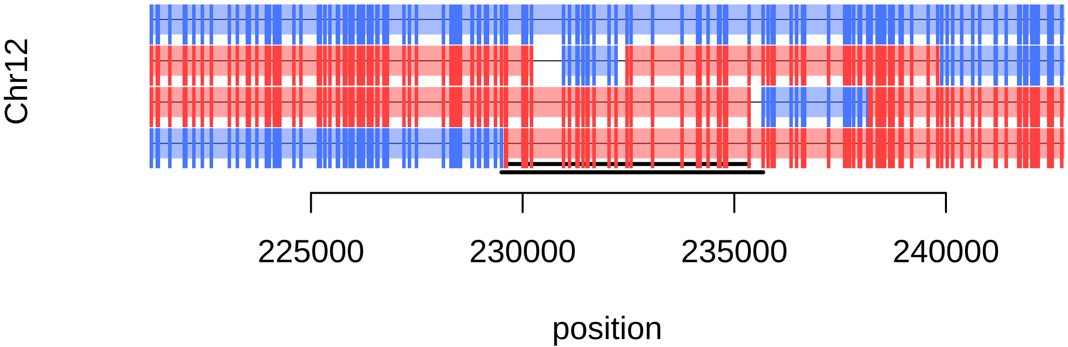

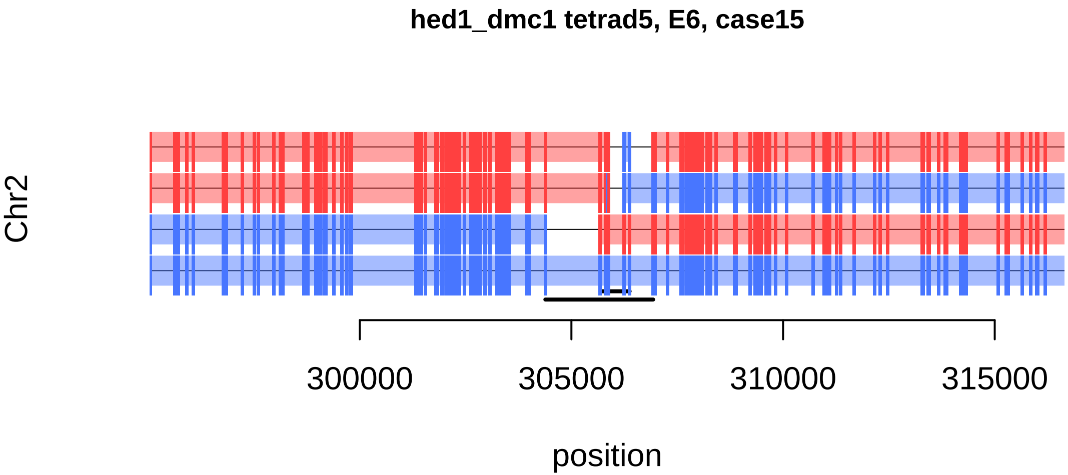
hed1_dmc1 tetrad5, E6, case15
Chr2
300000
305000
310000
315000
position

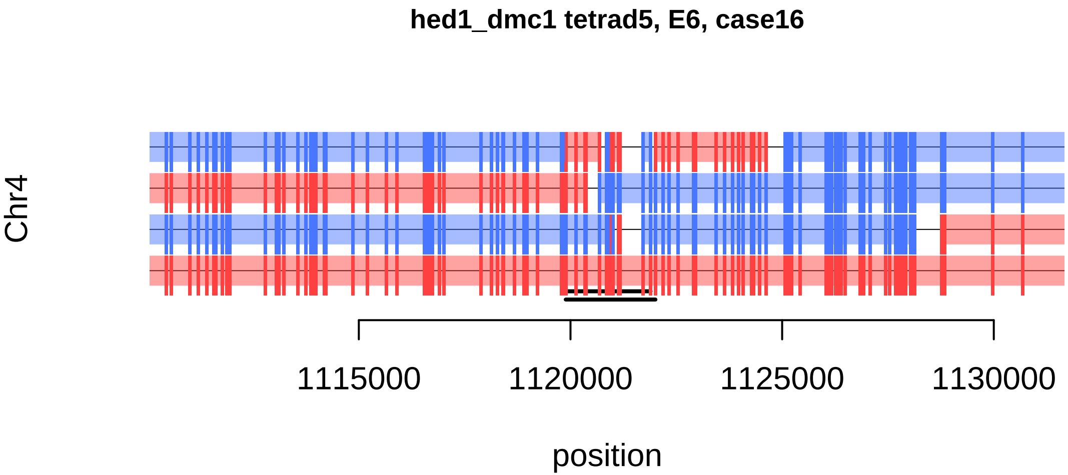

hed1_dmc1 tetrad5, E6, case16
Chr4
1115000
1120000
1125000
1130000
position

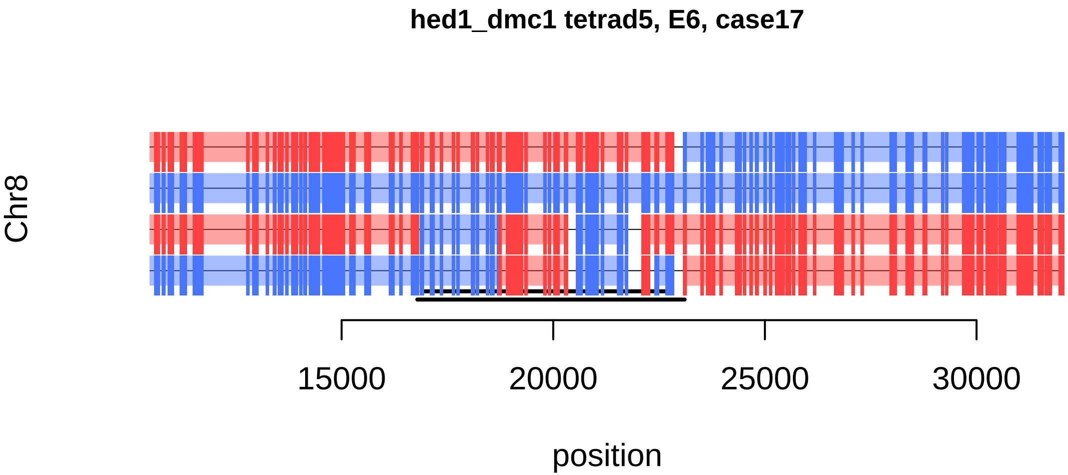

hed1_dmc1 tetrad5, E6, case17
Chr8
15000
20000
25000
30000
position

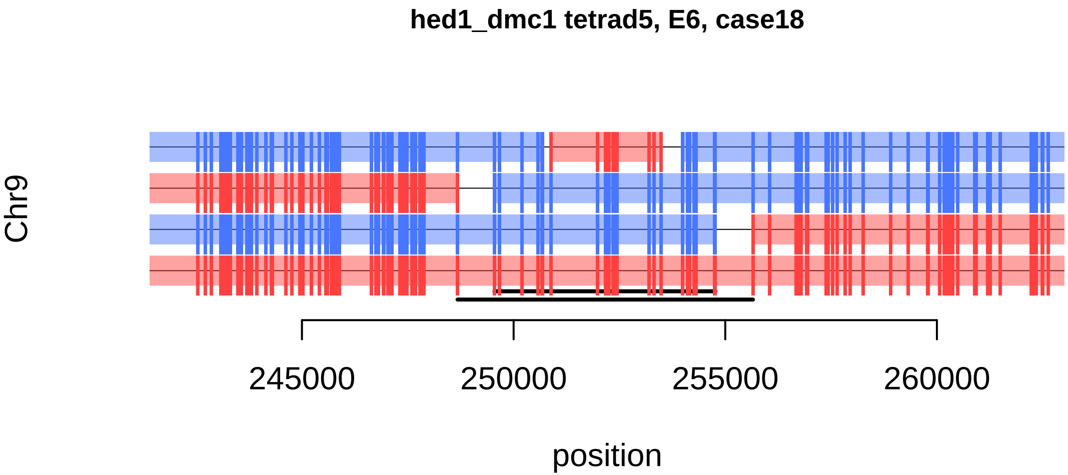
hed1_dmc1 tetrad5, E6, case18
Chr9
245000
250000
255000
260000
position

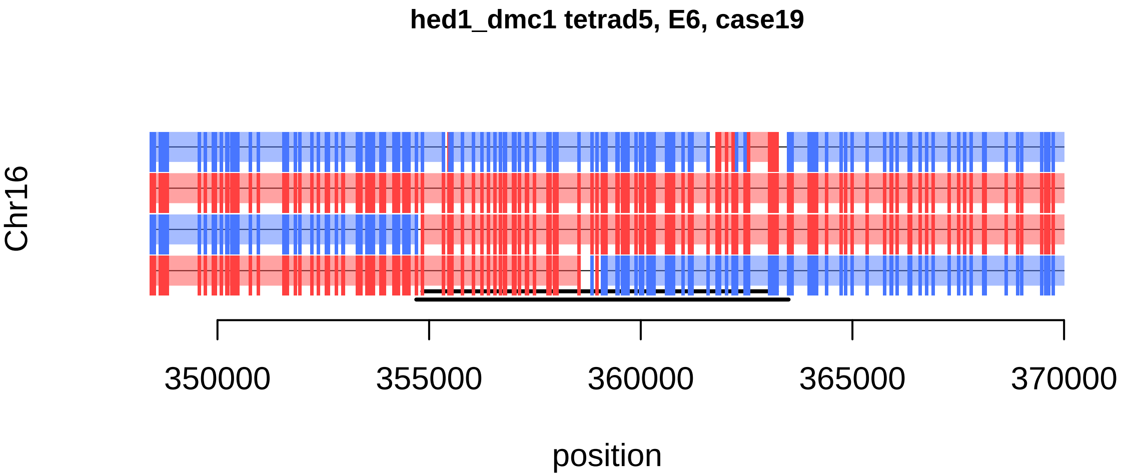
hed1_dmc1 tetrad5, E6, case19
Chr16
350000
355000
360000
365000
370000
position

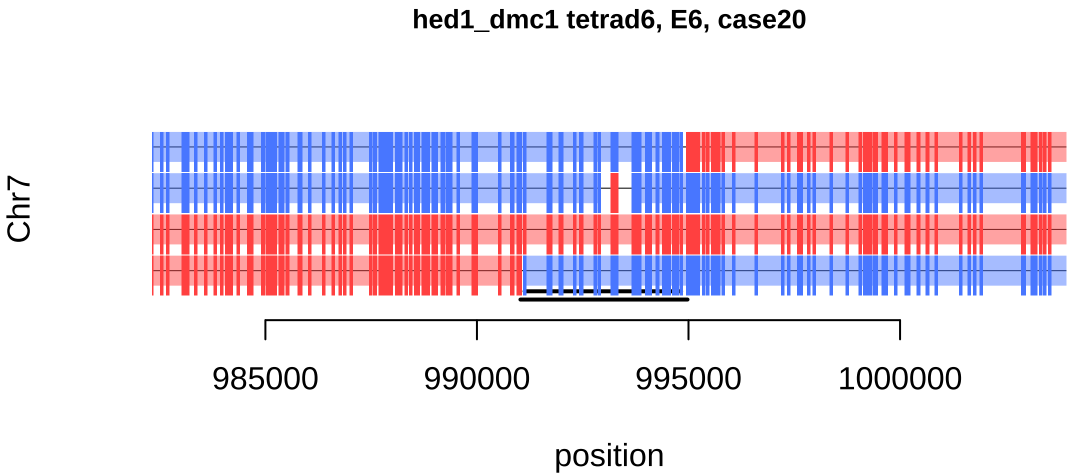
hed1_dmc1 tetrad6, E6, case20
Chr7
985000
990000
995000
1000000
position

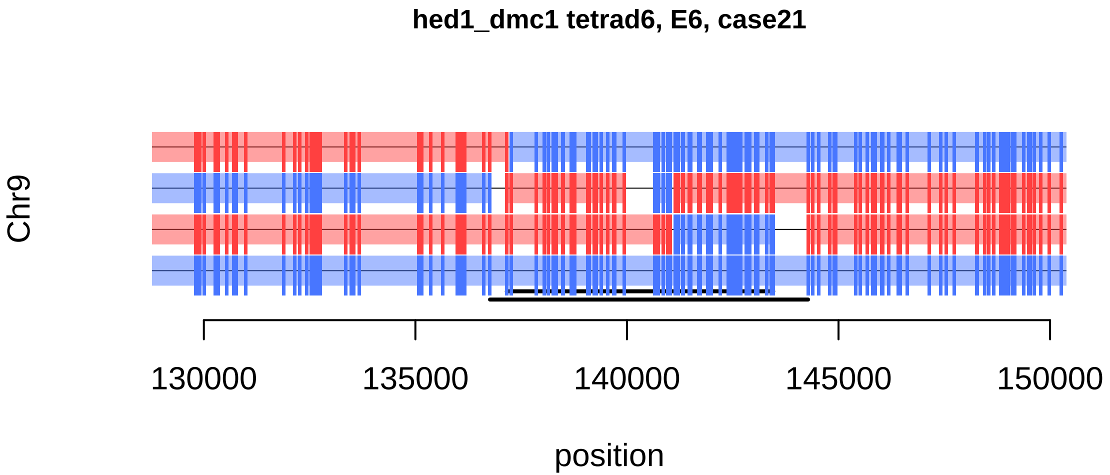
hed1_dmc1 tetrad6, E6, case21
Chr9
130000
135000
140000
145000
150000
position

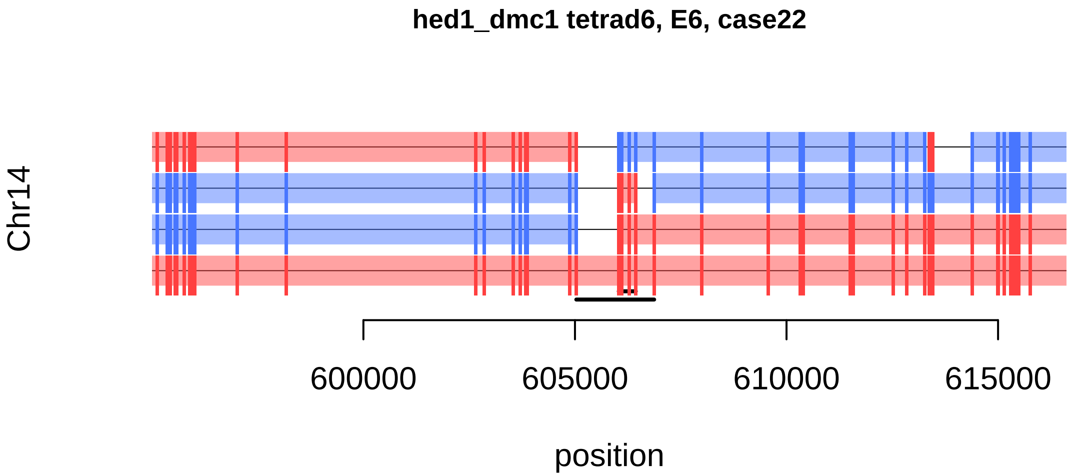
hed1_dmc1 tetrad6, E6, case22
Chr14
600000
605000
610000
615000
position

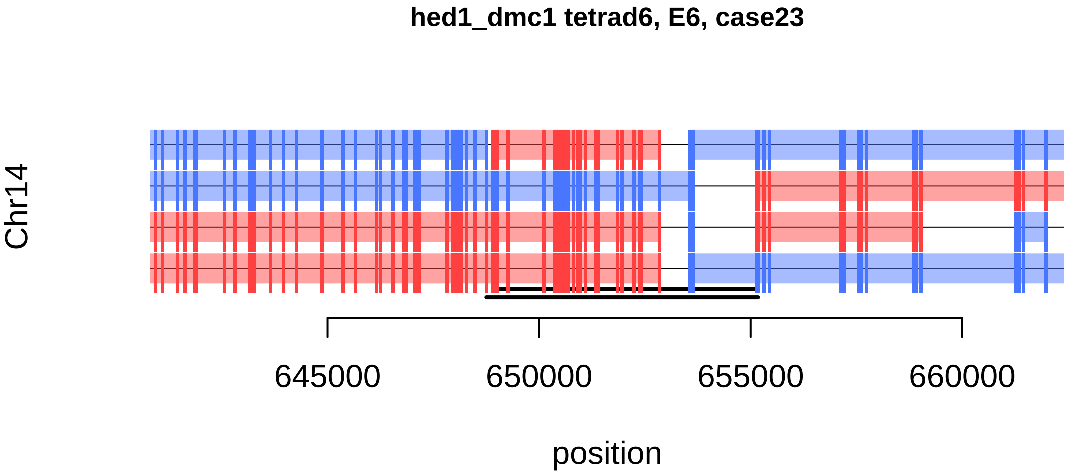
hed1_dmc1 tetrad6, E6, case23
Chr14
645000
650000
655000
660000
position

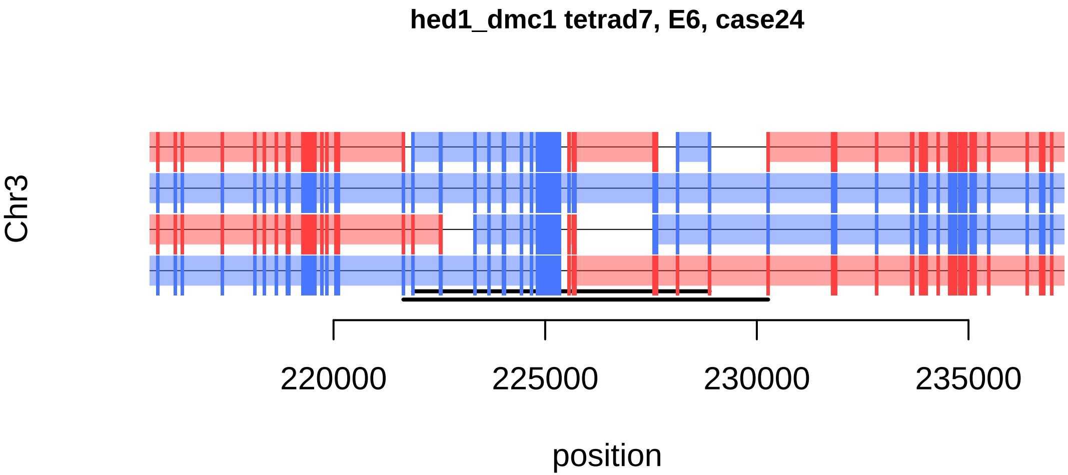
hed1_dmc1 tetrad7, E6, case24
Chr3
220000
225000
230000
235000
position

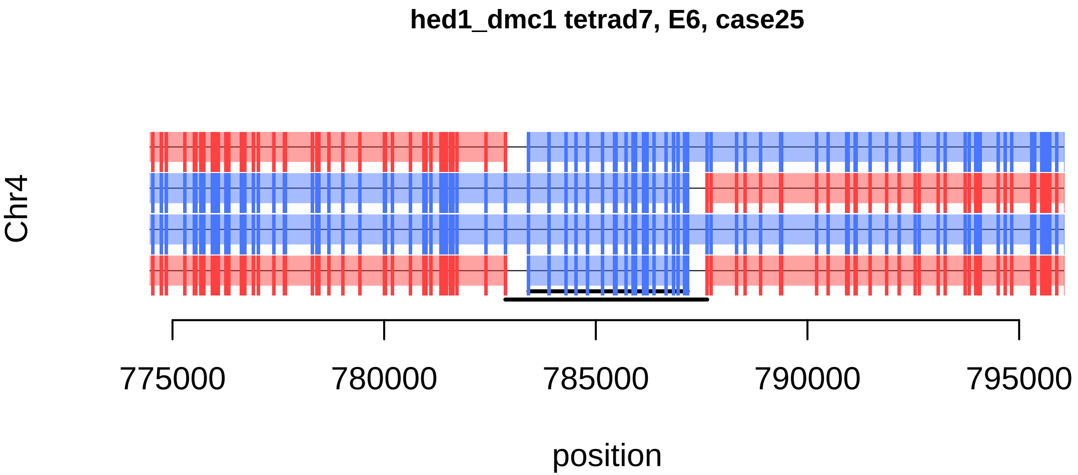

hed1_dmc1 tetrad7, E6, case25
Chr4
775000
780000
785000
790000
795000
position

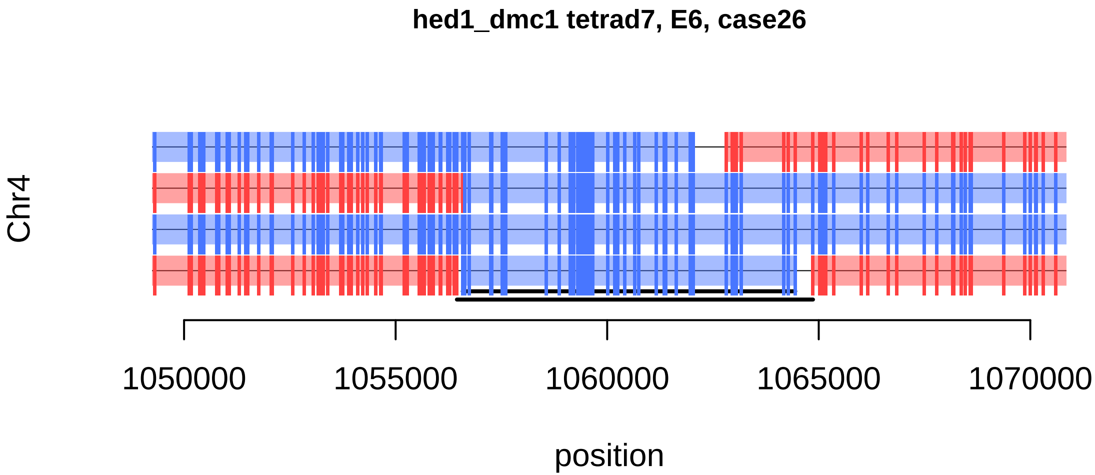
hed1_dmc1 tetrad7, E6, case26
Chr4
1050000
1055000
1060000
1065000
1070000
position

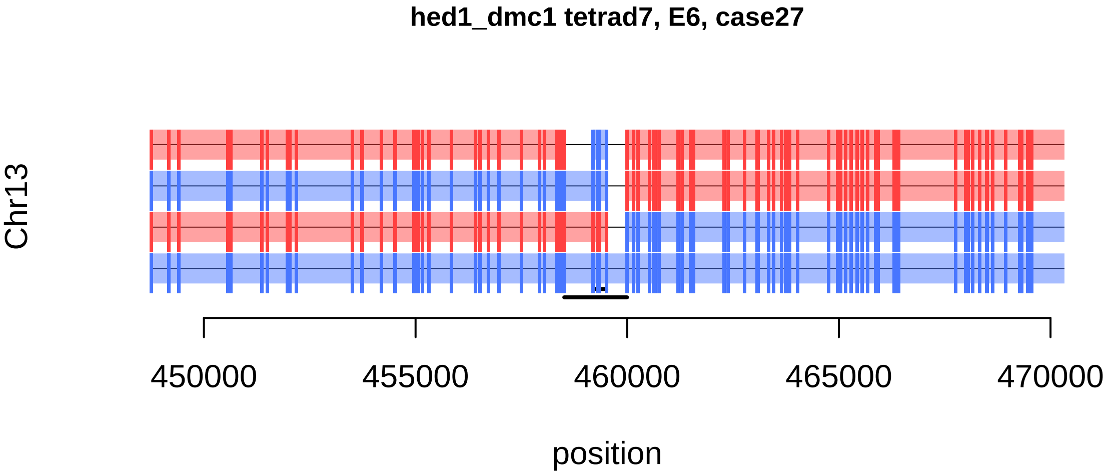

hed1_dmc1 tetrad7, E6, case27
Chr13
450000
455000
460000
465000
470000
position

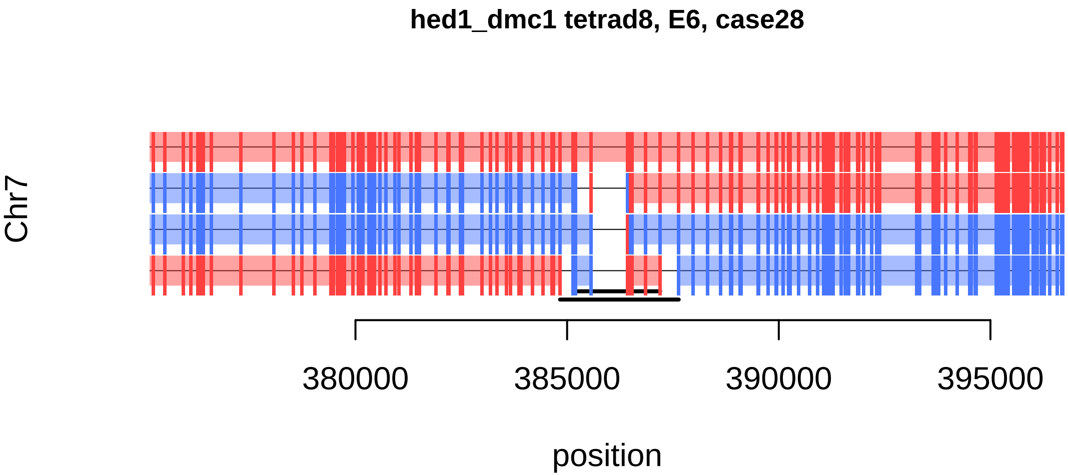
hed1_dmc1 tetrad8, E6, case28
Chr7
380000
385000
390000
395000
position

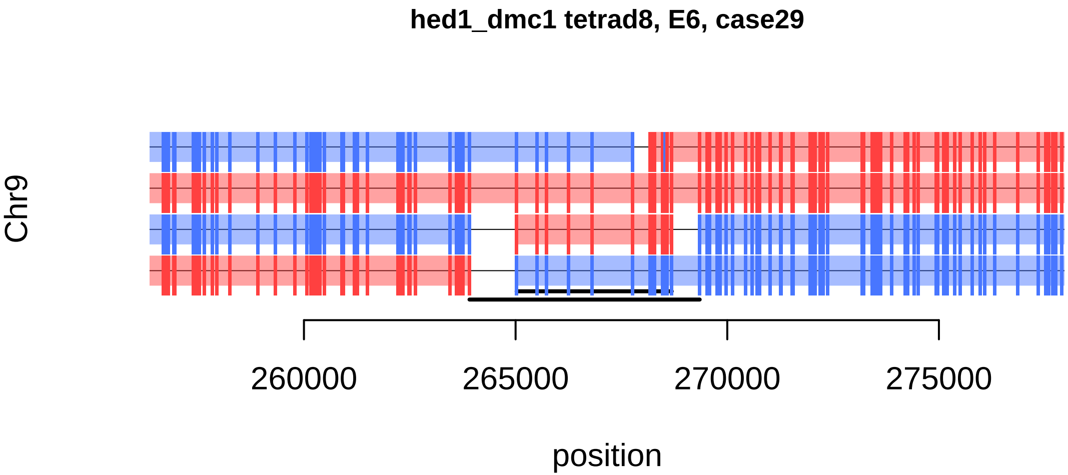
hed1_dmc1 tetrad8, E6, case29
Chr9
260000
265000
270000
275000
position

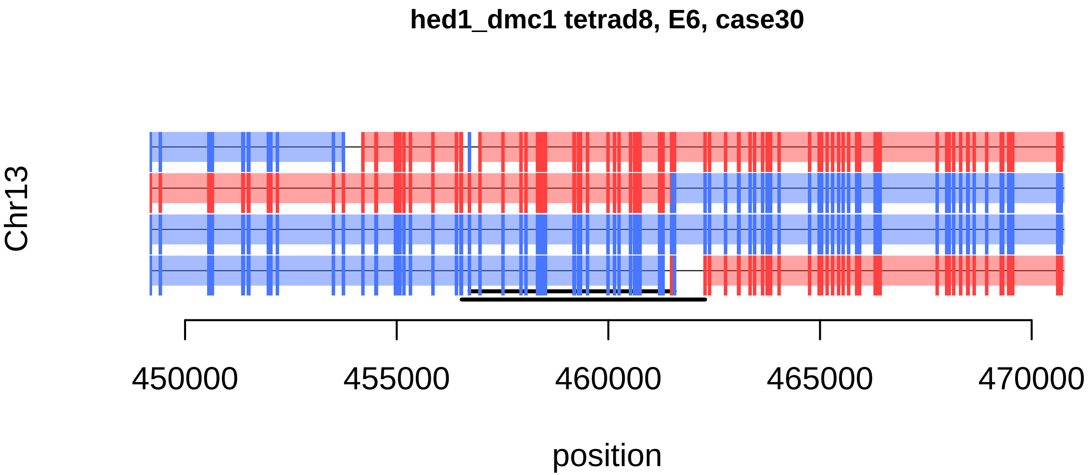

hed1_dmc1 tetrad8, E6, case30
Chr13
450000
455000
460000
465000
470000
position

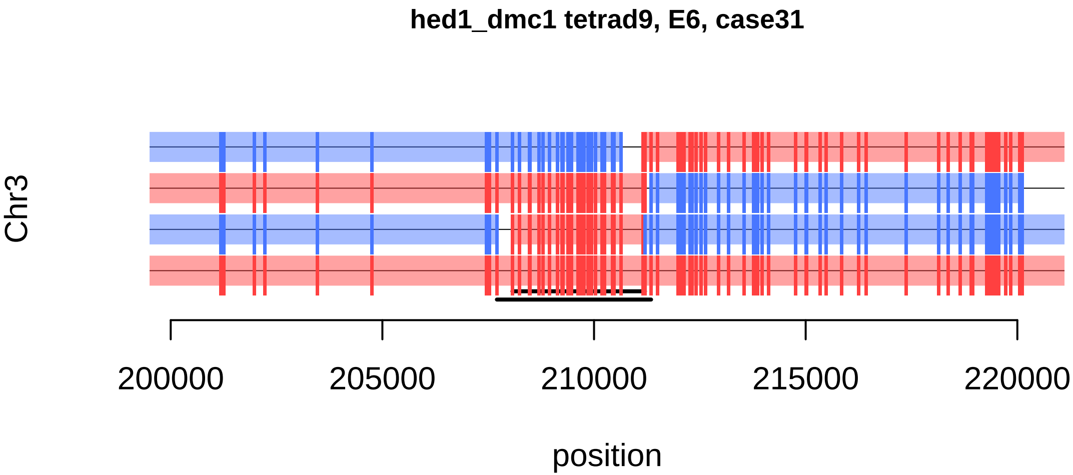
hed1_dmc1 tetrad9, E6, case31
Chr3
200000
205000
210000
215000
220000
position

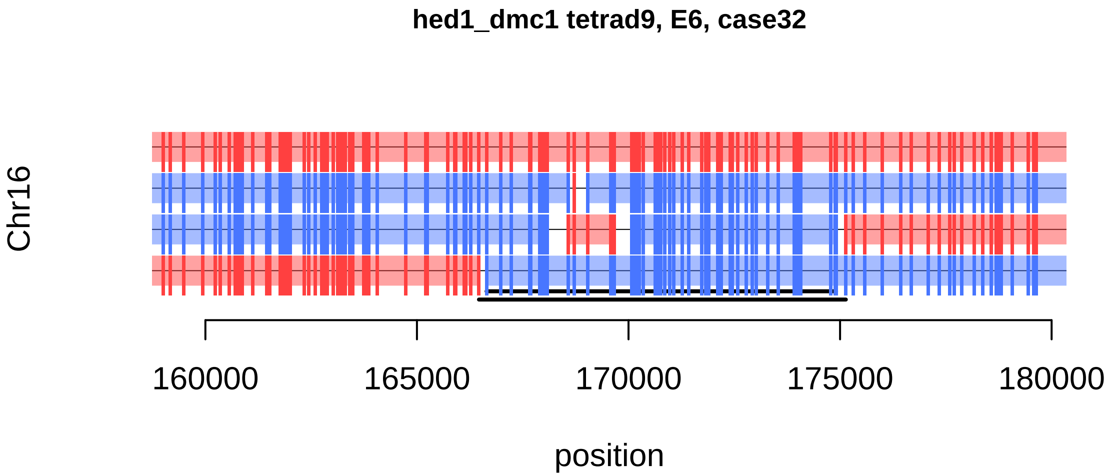
hed1_dmc1 tetrad9, E6, case32
Chr16
160000
165000
170000
175000
180000
position

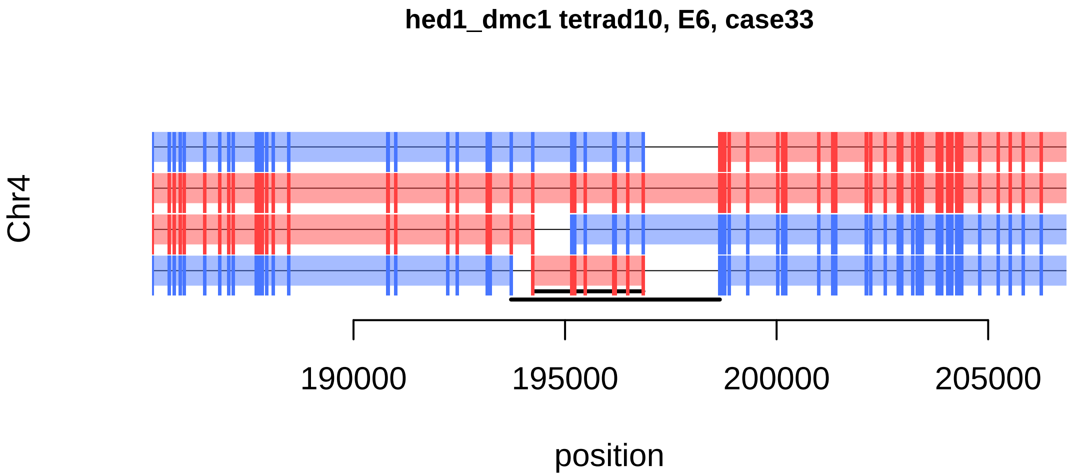
hed1_dmc1 tetrad10, E6, case33
Chr4
190000
195000
200000
205000
position

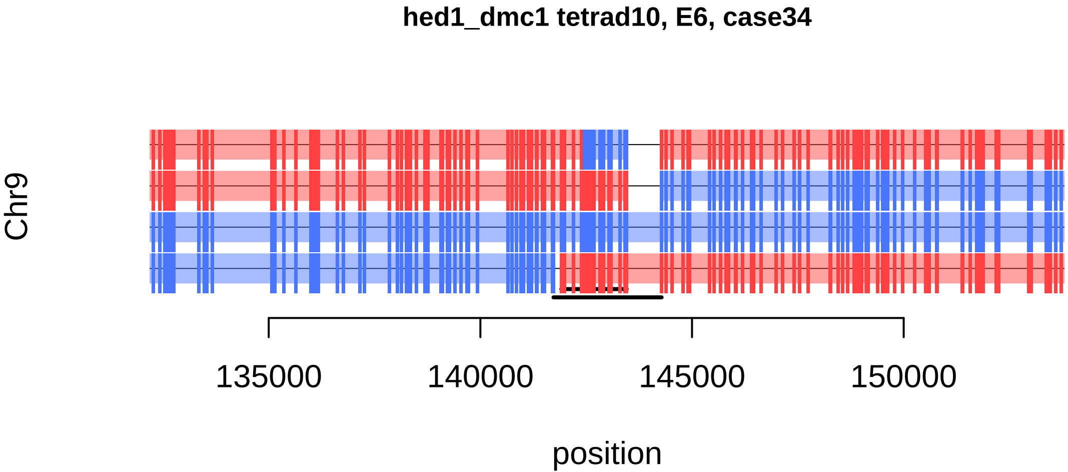
hed1_dmc1 tetrad10, E6, case34
Chr9
135000
140000
145000
150000
position

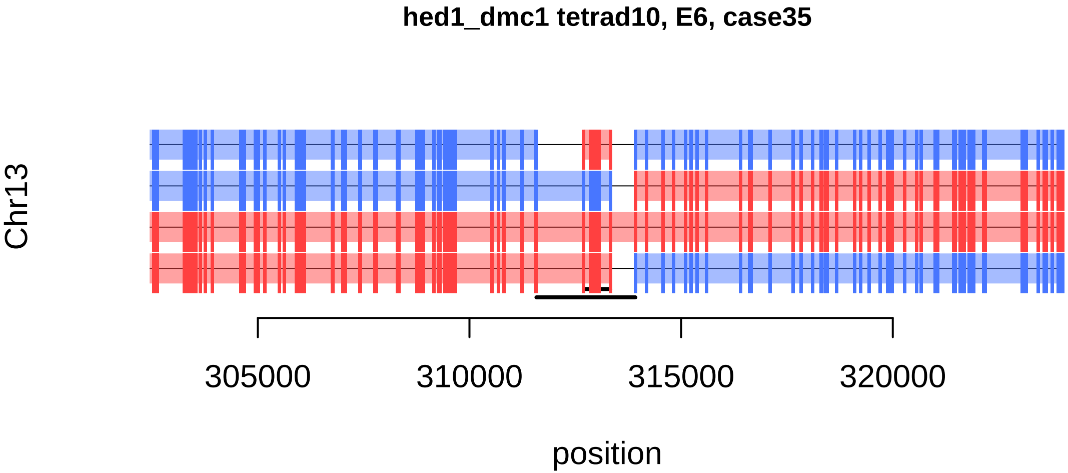
hed1_dmc1 tetrad10, E6, case35
Chr13
305000
310000
315000
320000
position

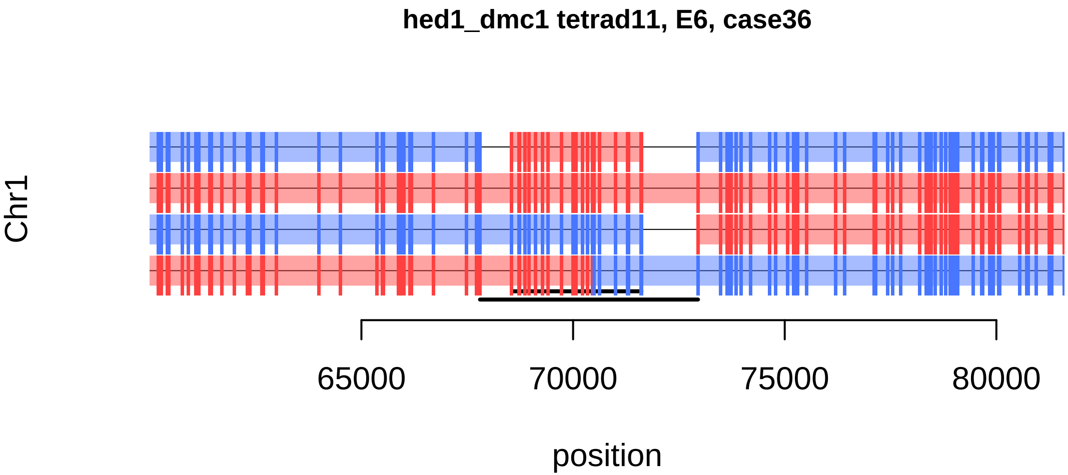

hed1_dmc1 tetrad11, E6, case36
Chr1
65000
70000
75000
80000
position

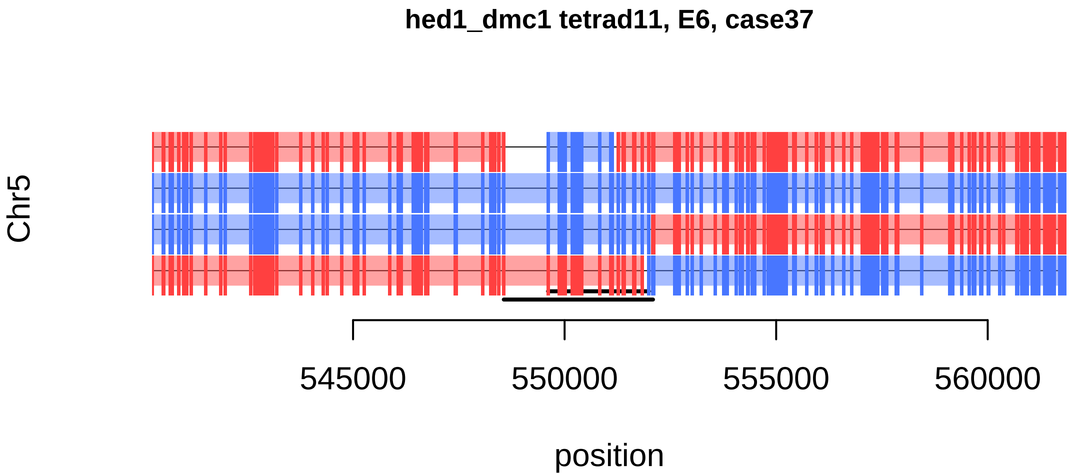
hed1_dmc1 tetrad11, E6, case37
Chr5
545000
550000
555000
560000
position

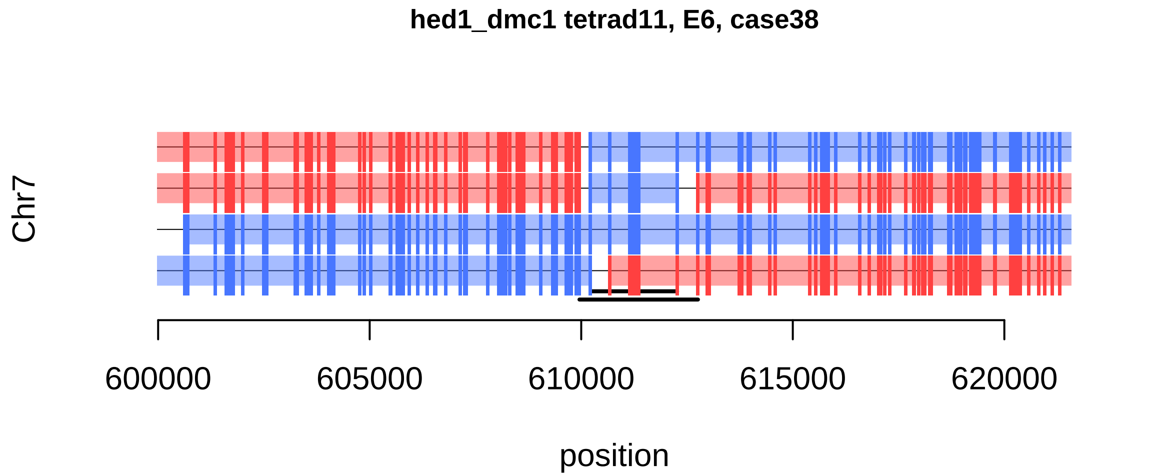

hed1_dmc1 tetrad11, E6, case38
Chr7
600000
605000
610000
615000
620000
position

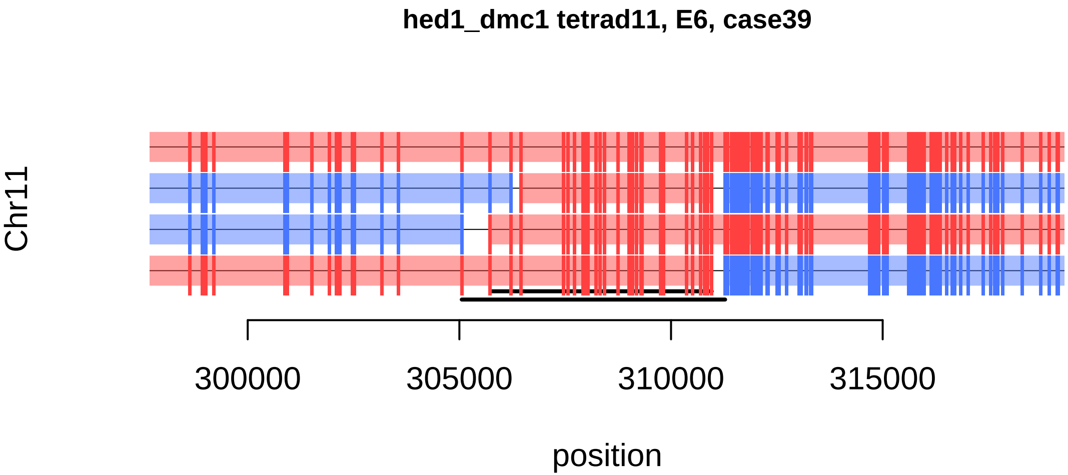
hed1_dmc1 tetrad11, E6, case39
Chr11
300000
305000
310000
315000
position

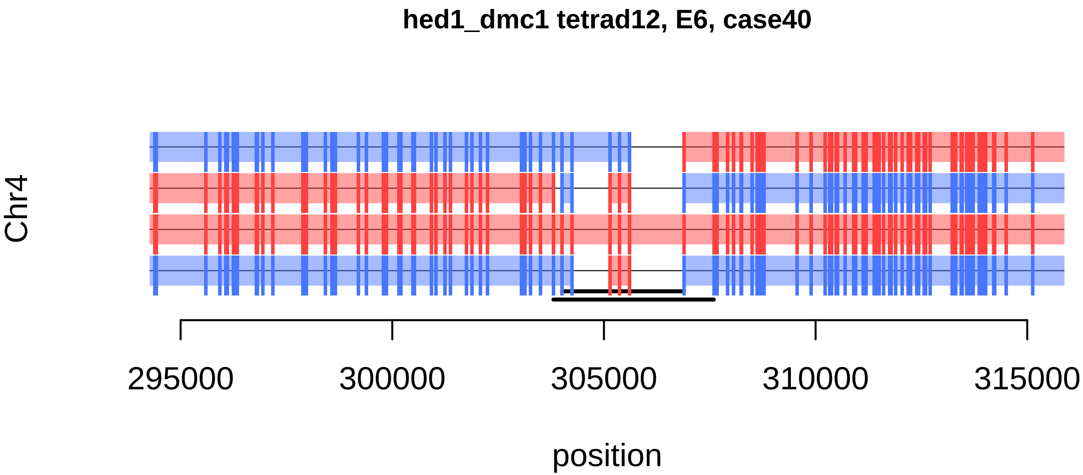

hed1_dmc1 tetrad12, E6, case40
Chr4
295000
300000
305000
310000
315000
position

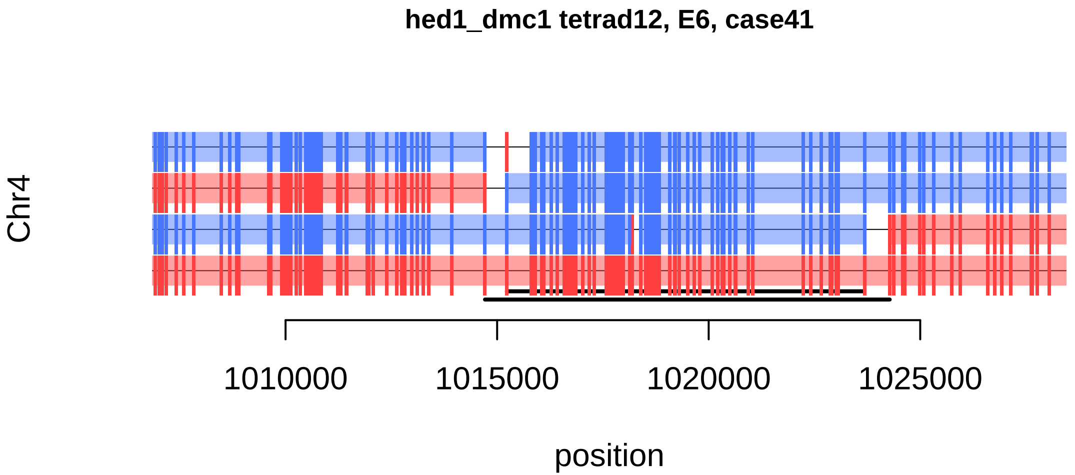
hed1_dmc1 tetrad12, E6, case41
Chr4
1010000
1015000
1020000
1025000
position

hed1_dmc1 tetrad5, E7, case1

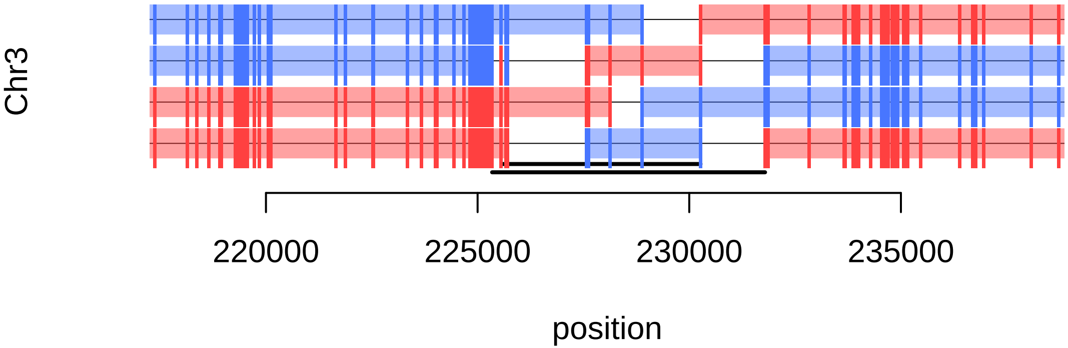

Supplement: S6 Fig — (PDF) [file pgen.1006226.s009.pdf]
