# Supplementary material for: Mek1 Down Regulates Rad51 Activity during Yeast Meiosis by Phosphorylation of Hed1
Source: PLoS Genet. 2016 Aug 2;12(8):e1006226. doi: 10.1371/journal.pgen.1006226 (PMC4970670; doi:10.1371/journal.pgen.1006226)
Supplement: S7 Fig — (PDF) [file pgen.1006226.s010.pdf]

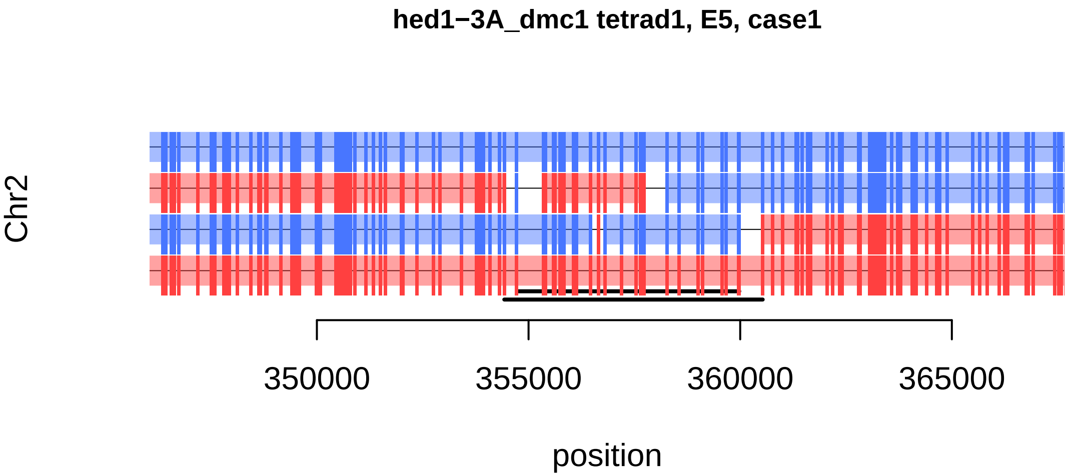
hed1−3A_dmc1 tetrad1, E5, case1
Chr2
350000
355000
360000
365000
position

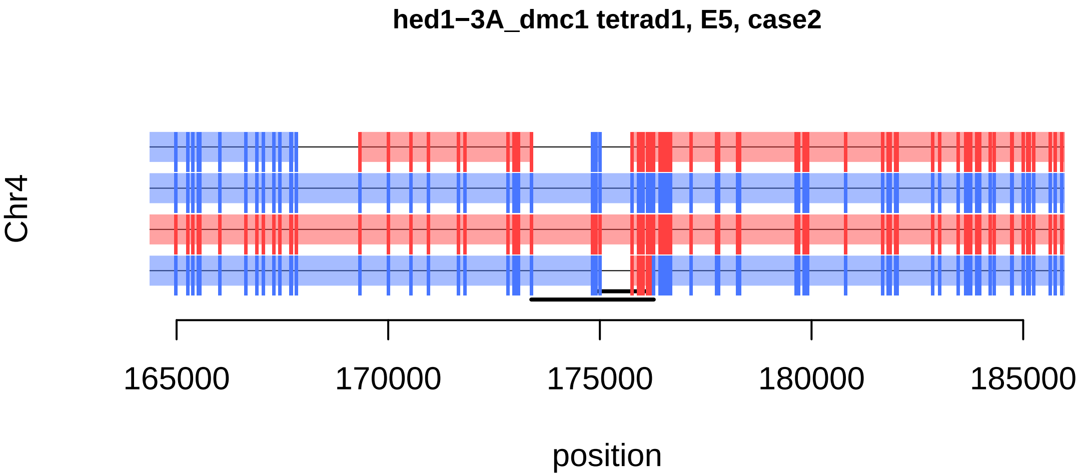
hed1−3A_dmc1 tetrad1, E5, case2
Chr4
165000
170000
175000
180000
185000
position

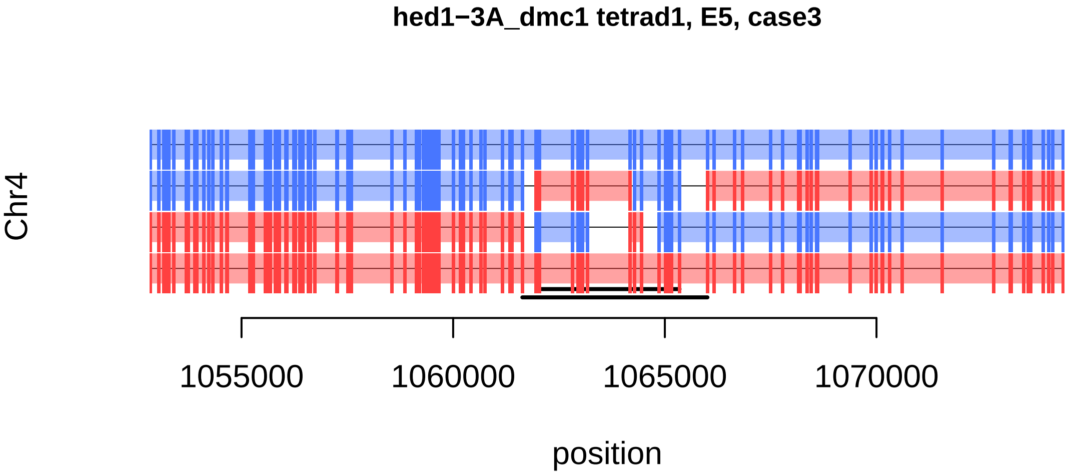
hed1−3A_dmc1 tetrad1, E5, case3
Chr4
1055000
1060000
1065000
1070000
position

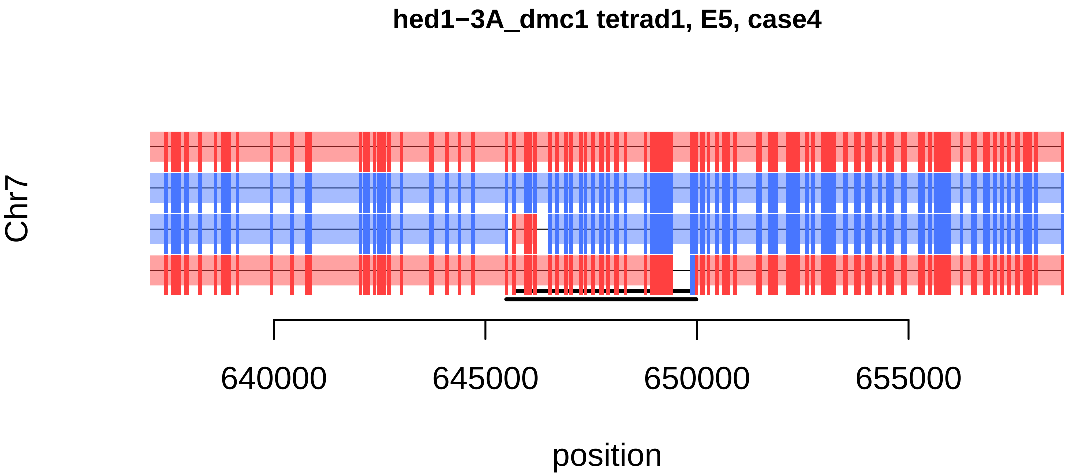
hed1−3A_dmc1 tetrad1, E5, case4
Chr7
640000
645000
650000
655000
position

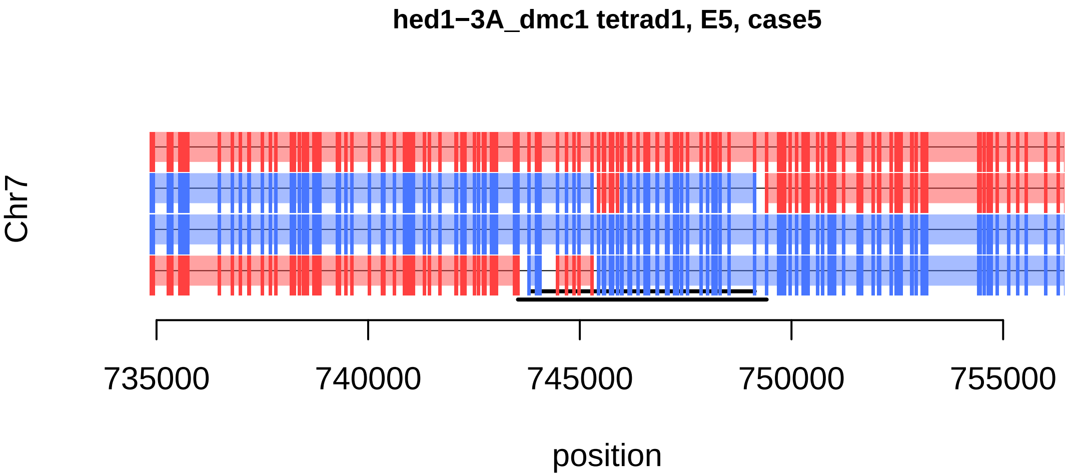
hed1−3A_dmc1 tetrad1, E5, case5
Chr7
735000
740000
745000
750000
755000
position

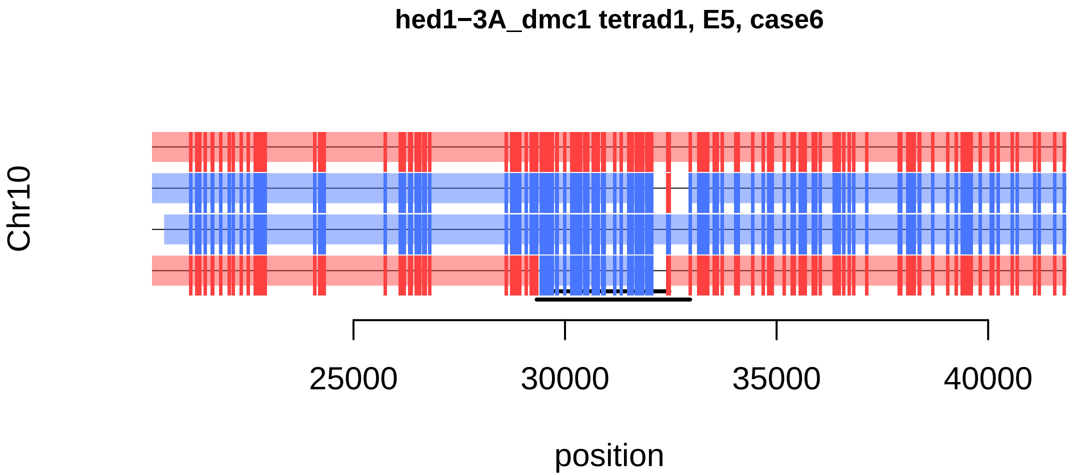
hed1−3A_dmc1 tetrad1, E5, case6
Chr10
25000
30000
35000
40000
position

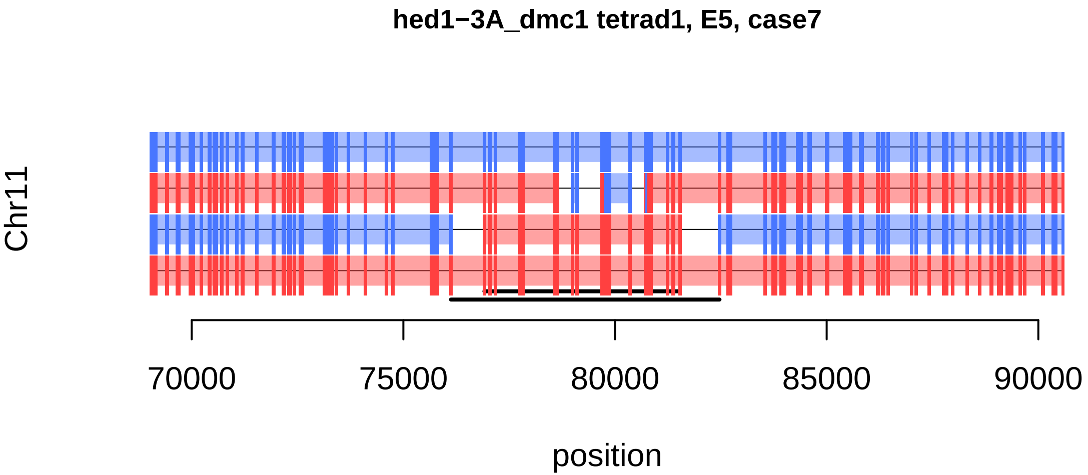
hed1−3A_dmc1 tetrad1, E5, case7
Chr11
70000
75000
80000
85000
90000
position

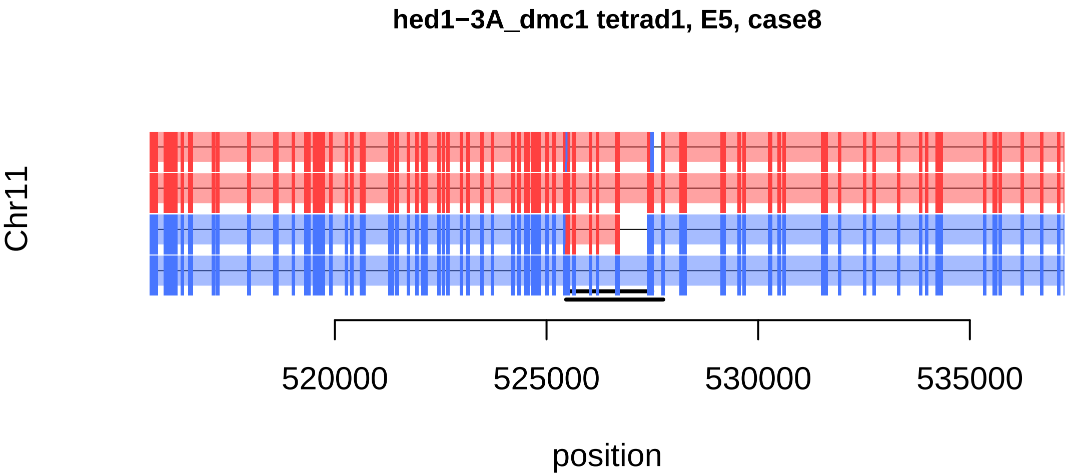
hed1−3A_dmc1 tetrad1, E5, case8
Chr11
520000
525000
530000
535000
position

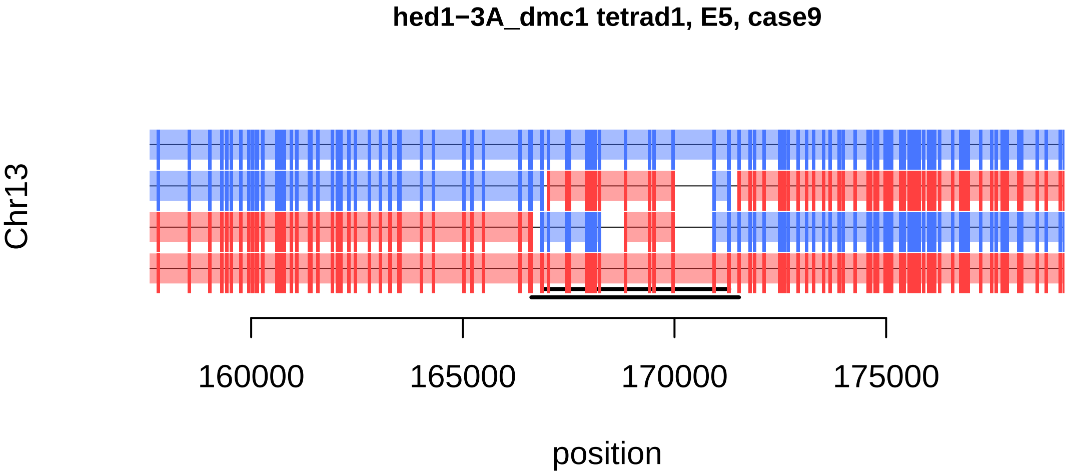

hed1−3A_dmc1 tetrad1, E5, case9
Chr13
160000
165000
170000
175000
position

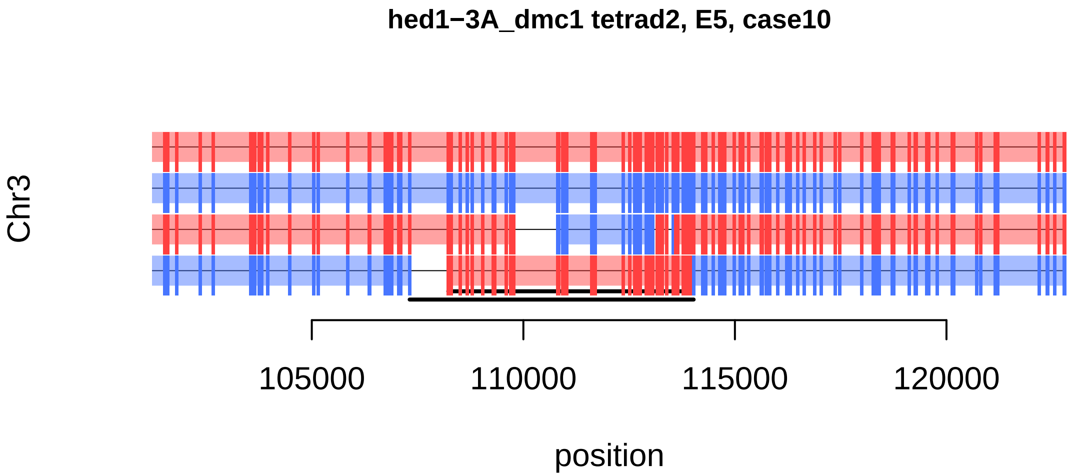
hed1−3A_dmc1 tetrad2, E5, case10
Chr3
105000
110000
115000
120000
position

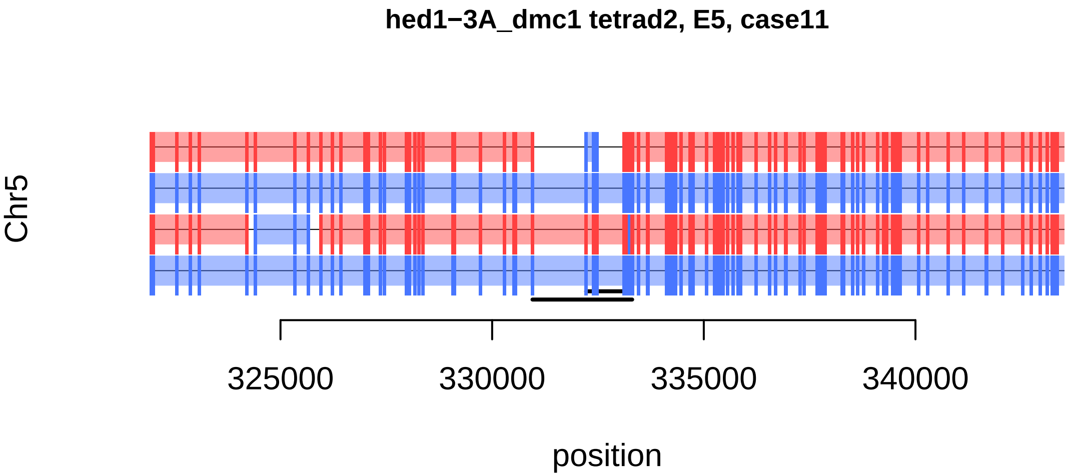
hed1−3A_dmc1 tetrad2, E5, case11
Chr5
325000
330000
335000
340000
position

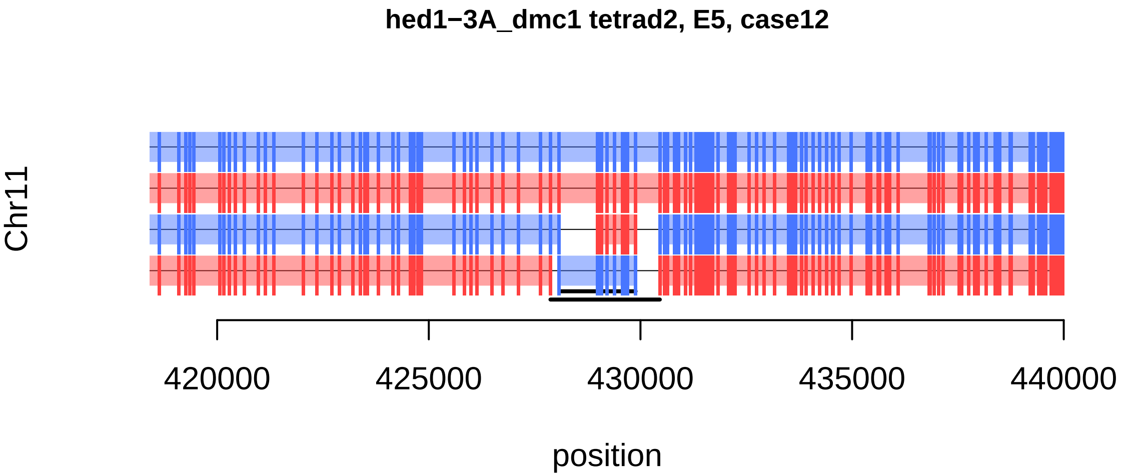
hed1−3A_dmc1 tetrad2, E5, case12
Chr11
420000
425000
430000
435000
440000
position

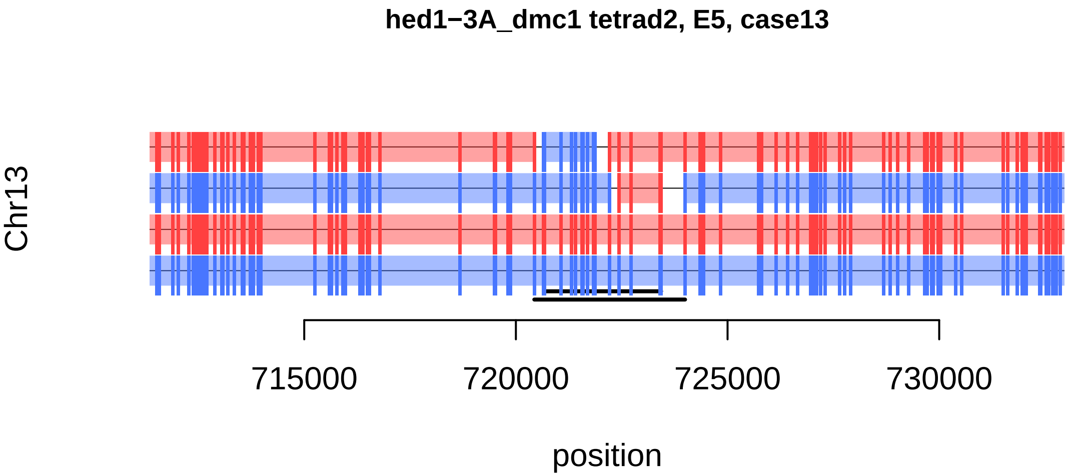
hed1−3A_dmc1 tetrad2, E5, case13
Chr13
715000
720000
725000
730000
position

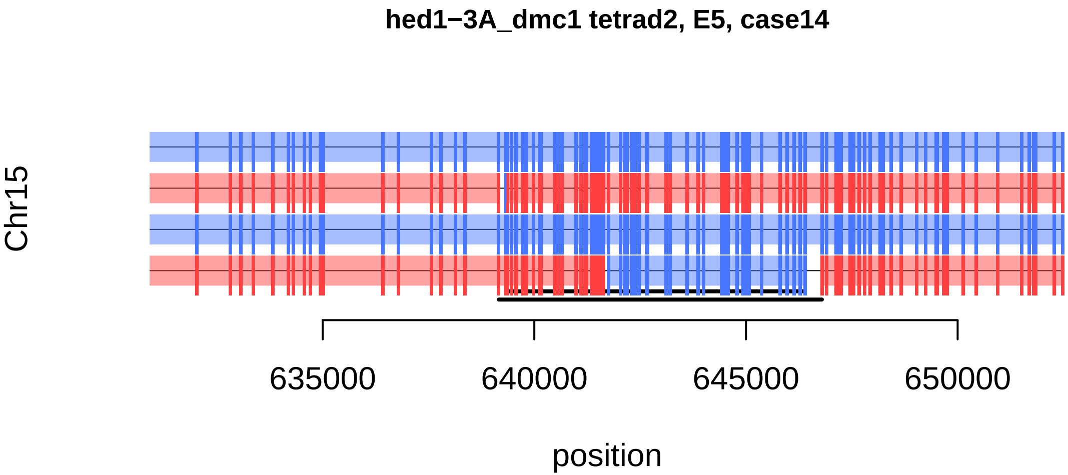

hed1−3A_dmc1 tetrad2, E5, case14
Chr15
635000
640000
645000
650000
position

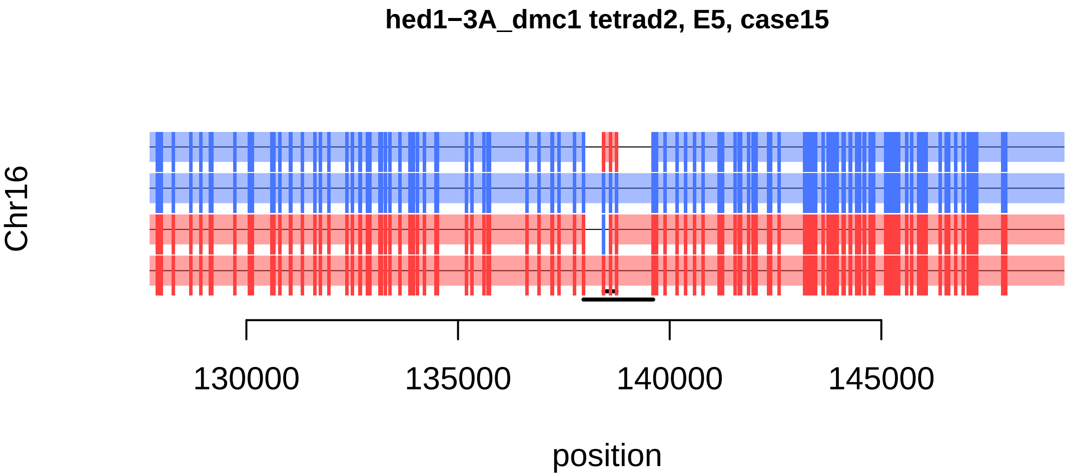
hed1−3A_dmc1 tetrad2, E5, case15
Chr16
130000
135000
140000
145000
position

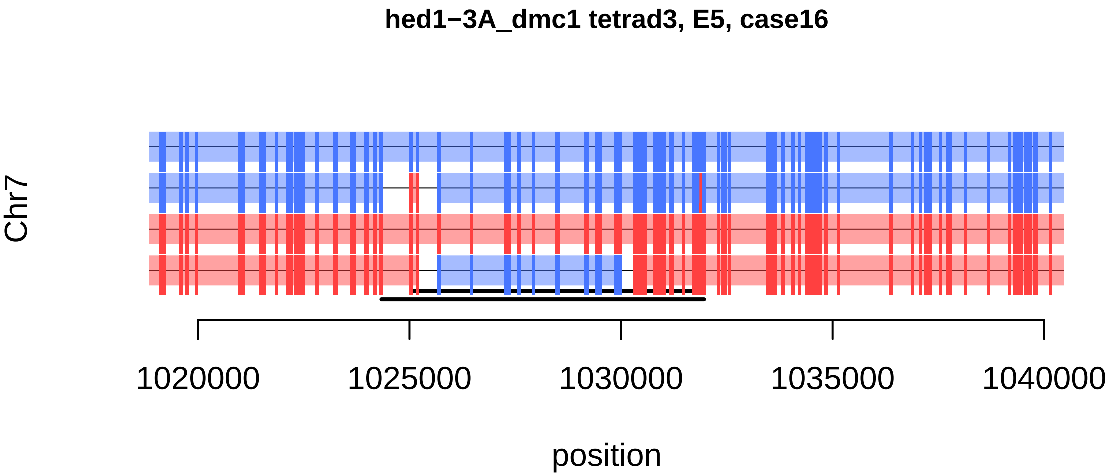

hed1−3A_dmc1 tetrad3, E5, case16
Chr7
1020000
1025000
1030000
1035000
1040000
position

hed1−3A_dmc1 tetrad3, E5, case17

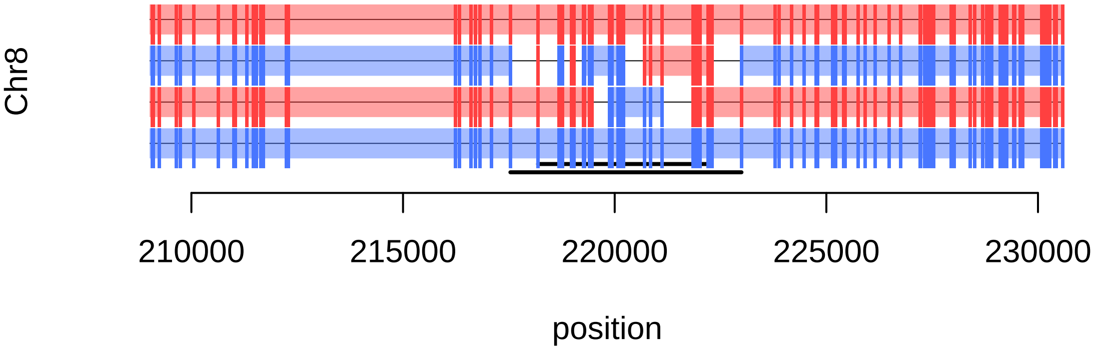

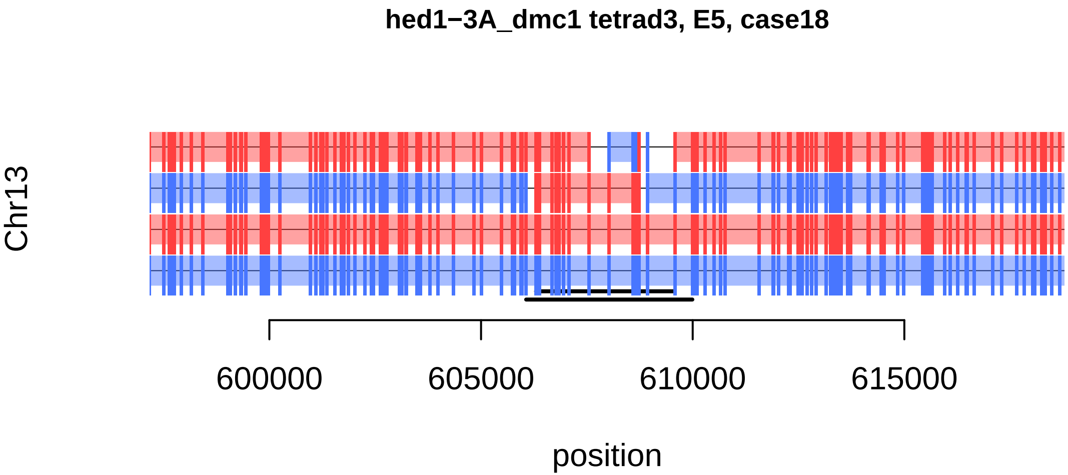
hed1−3A_dmc1 tetrad3, E5, case18
Chr13
600000
605000
610000
615000
position

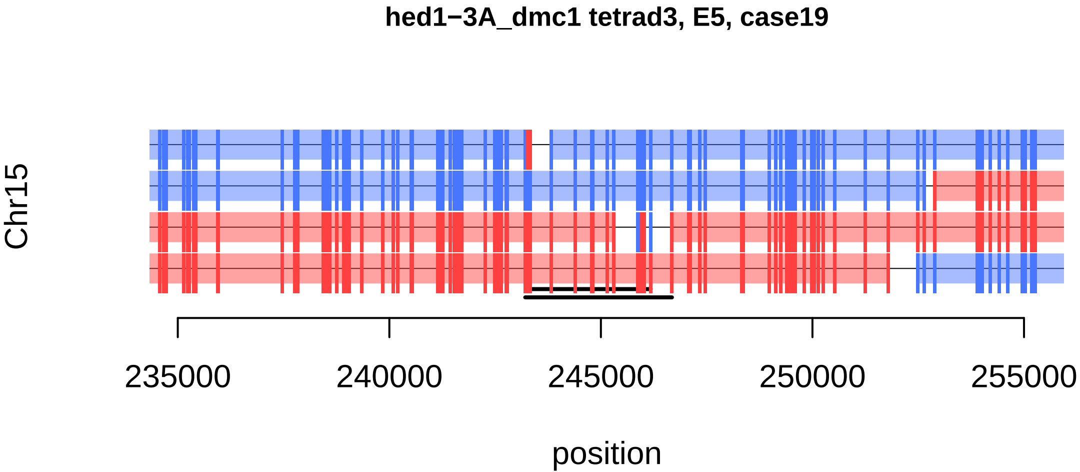
hed1−3A_dmc1 tetrad3, E5, case19
Chr15
235000
240000
245000
250000
255000
position

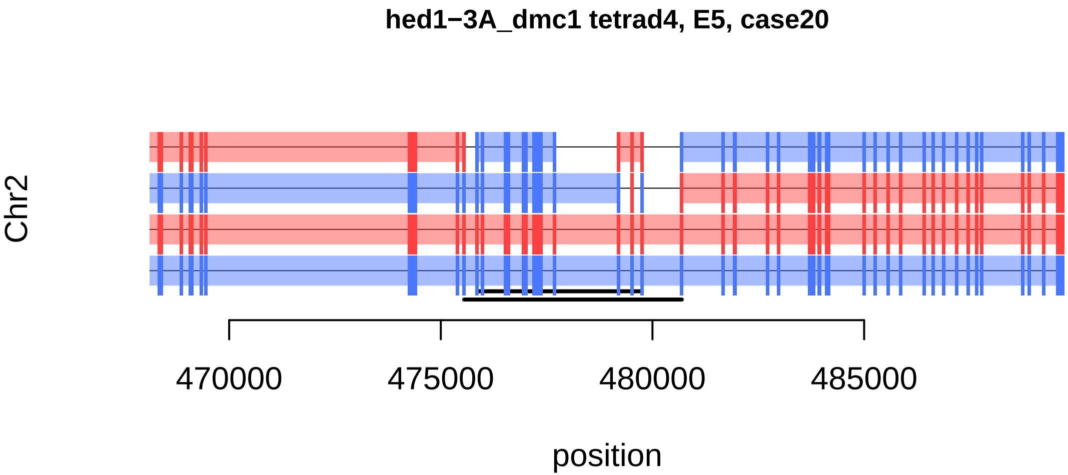
hed1−3A_dmc1 tetrad4, E5, case20
Chr2
470000
475000
480000
485000
position

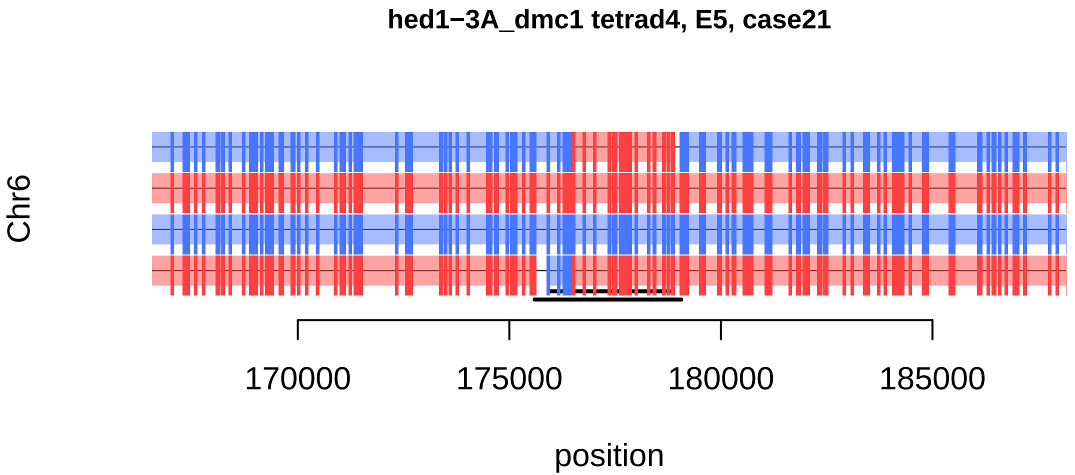
hed1−3A_dmc1 tetrad4, E5, case21
Chr6
170000
175000
180000
185000
position

hed1−3A_dmc1 tetrad4, E5, case22

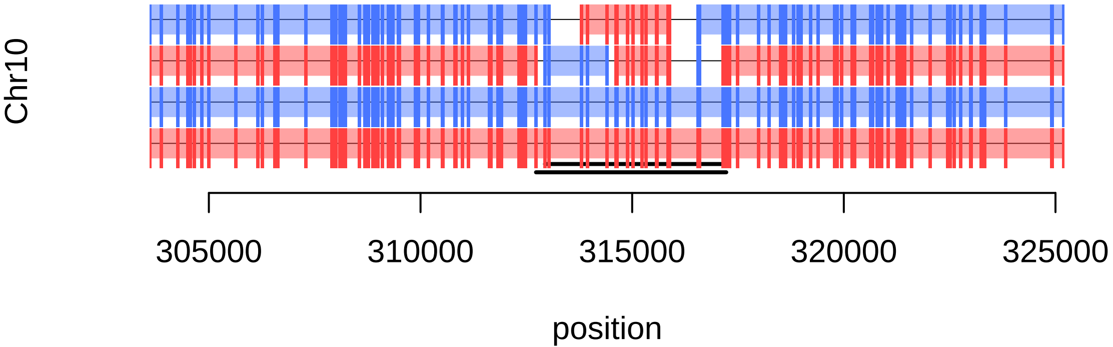

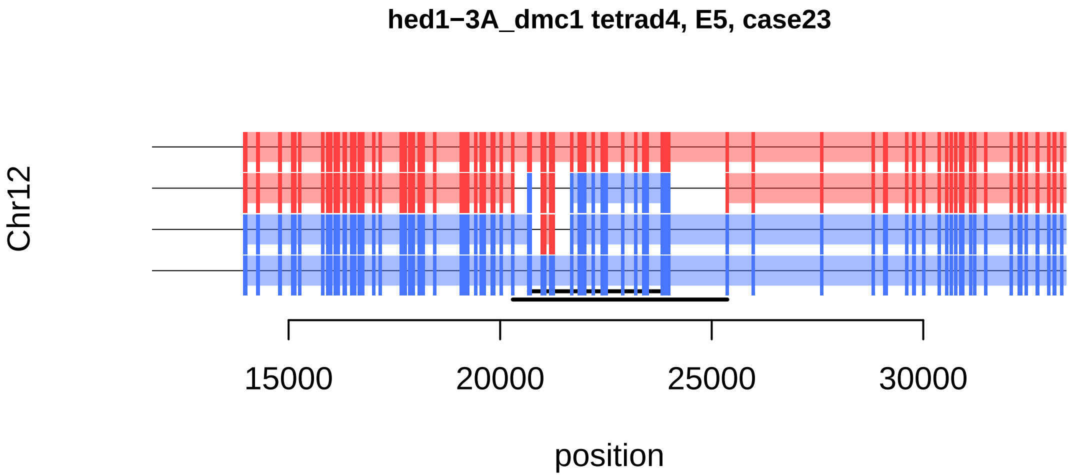
hed1−3A_dmc1 tetrad4, E5, case23
Chr12
15000
20000
25000
30000
position

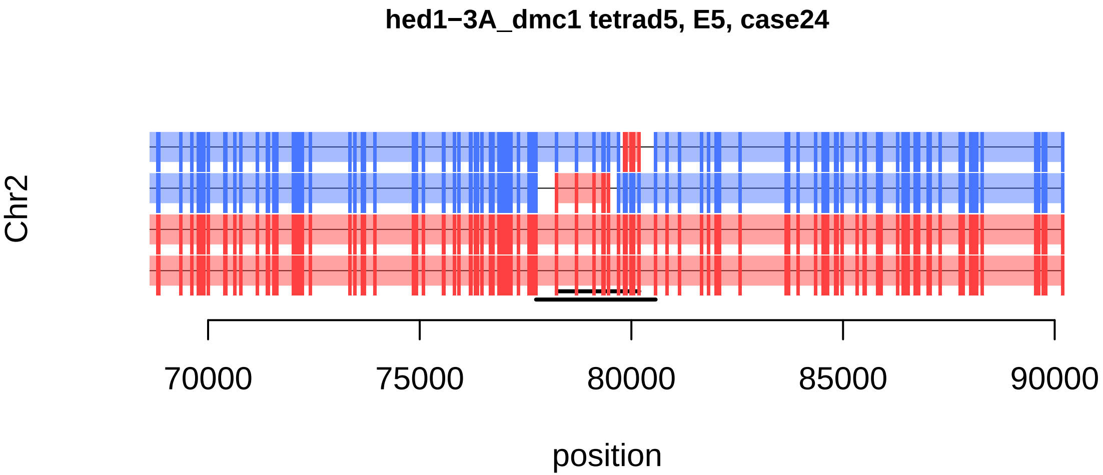

hed1−3A_dmc1 tetrad5, E5, case24
Chr2
70000
75000
80000
85000
90000
position

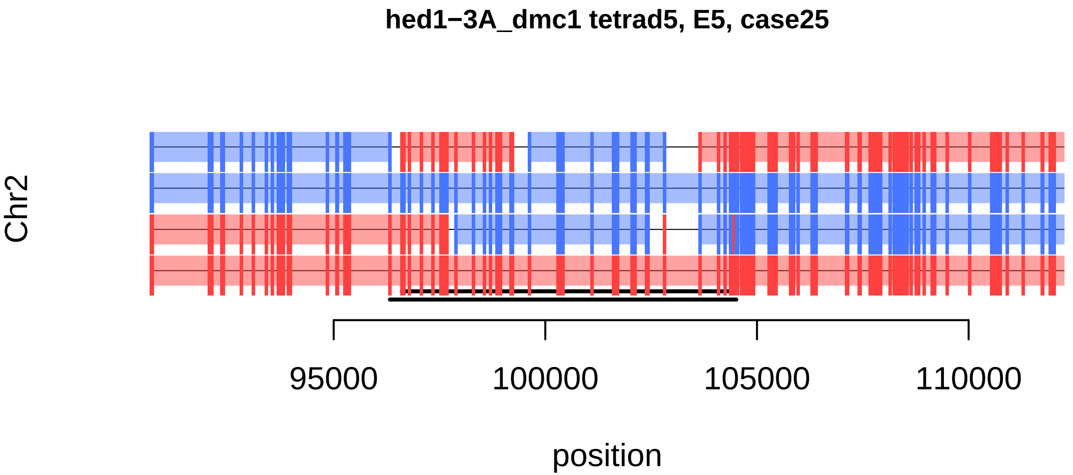
hed1−3A_dmc1 tetrad5, E5, case25
Chr2
95000
100000
105000
110000
position

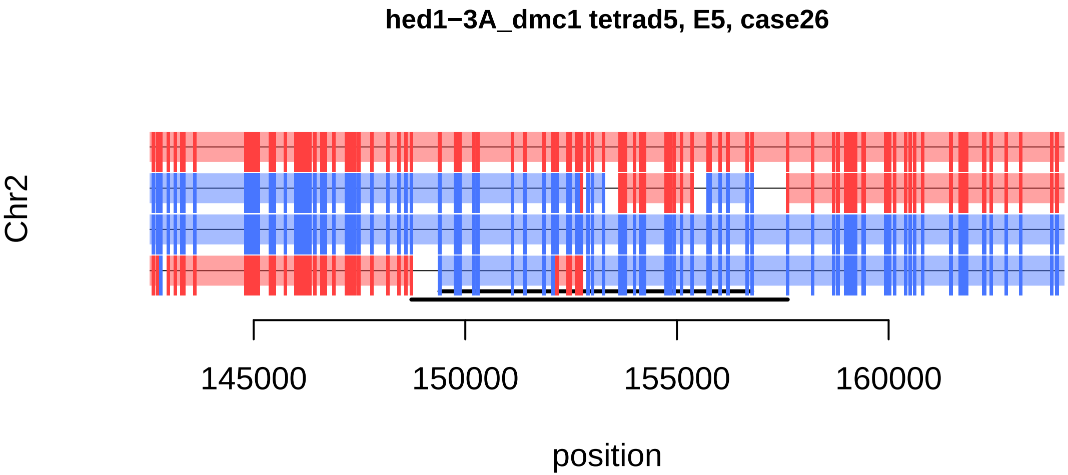
hed1−3A_dmc1 tetrad5, E5, case26
Chr2
145000
150000
155000
160000
position

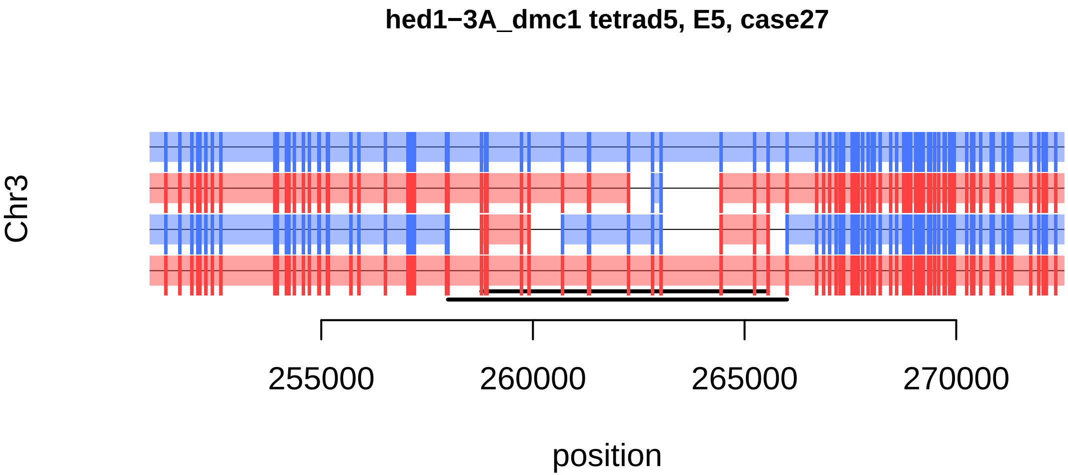
hed1−3A_dmc1 tetrad5, E5, case27
Chr3
255000
260000
265000
270000
position

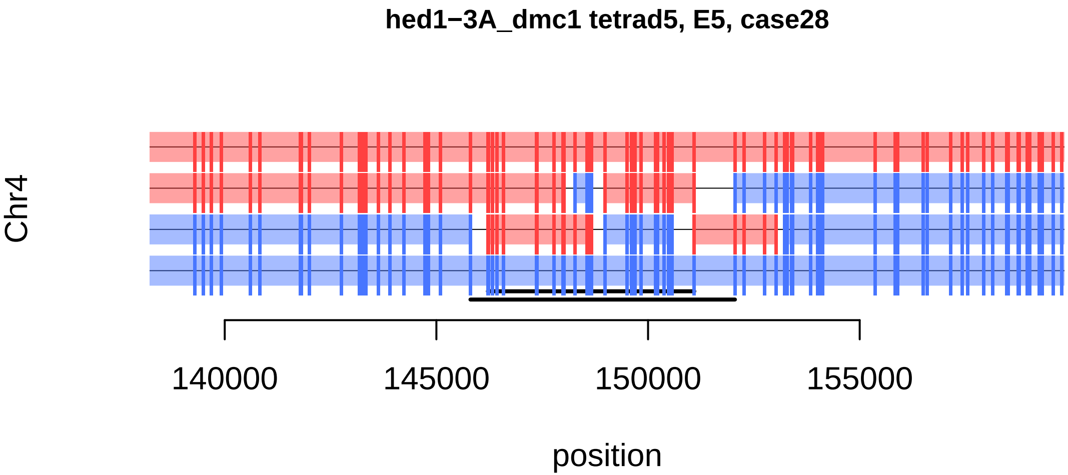
hed1−3A_dmc1 tetrad5, E5, case28
Chr4
140000
145000
150000
155000
position

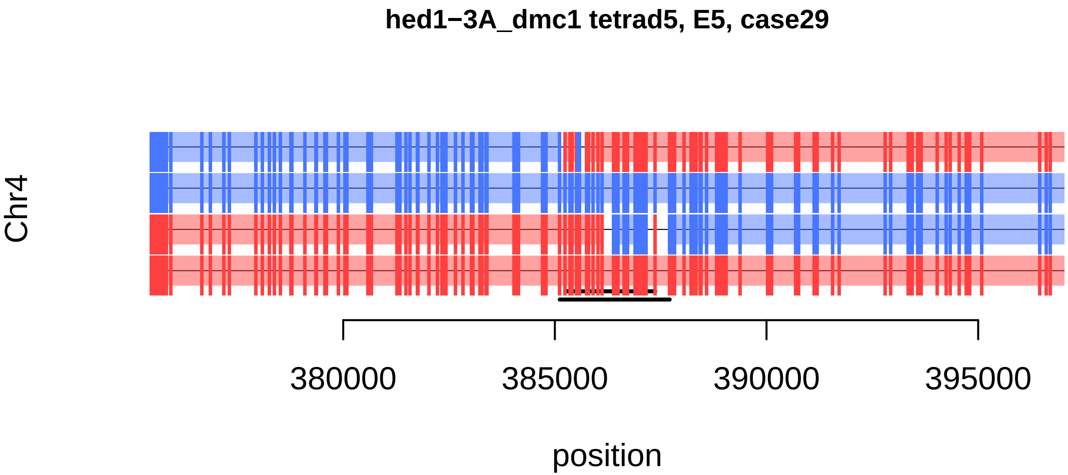

hed1−3A_dmc1 tetrad5, E5, case29
Chr4
380000
385000
390000
395000
position

hed1−3A_dmc1 tetrad5, E5, case30

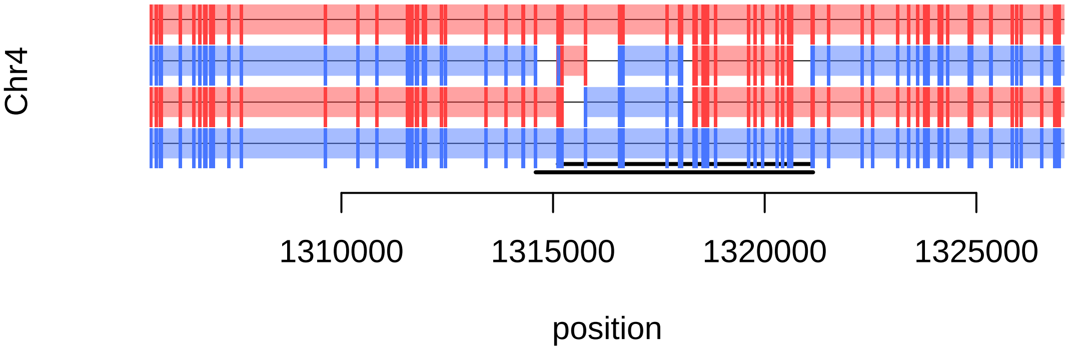

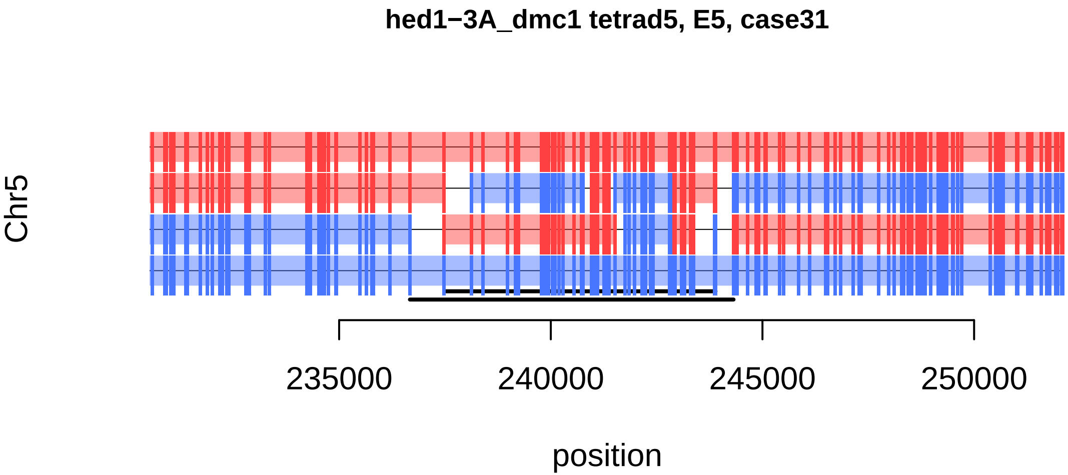

hed1−3A_dmc1 tetrad5, E5, case31
Chr5
235000
240000
245000
250000
position

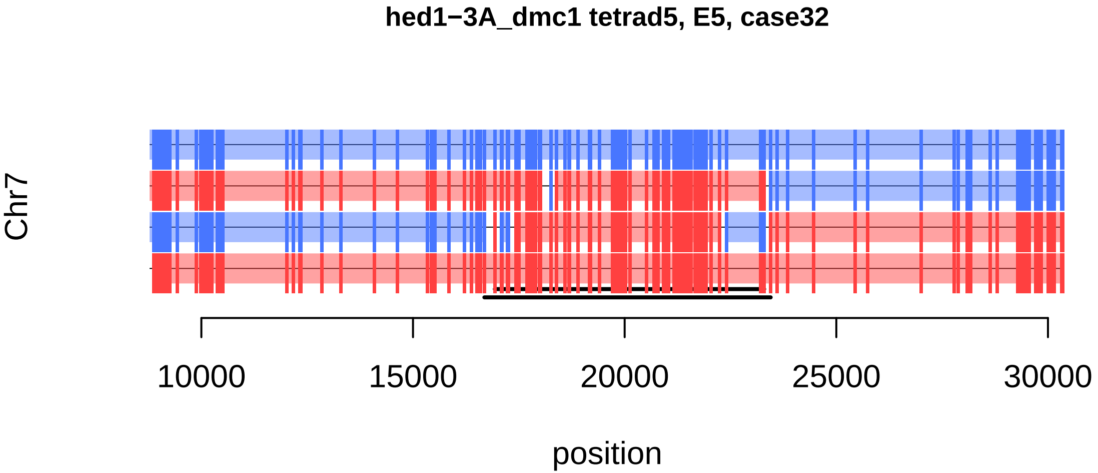

hed1−3A_dmc1 tetrad5, E5, case32
Chr7
10000
15000
20000
25000
30000
position

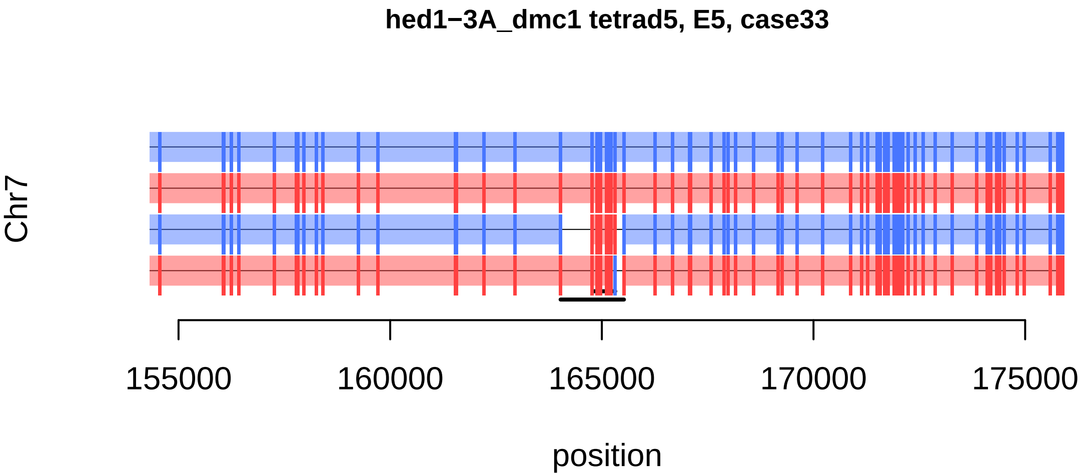
hed1−3A_dmc1 tetrad5, E5, case33
Chr7
155000
160000
165000
170000
175000
position

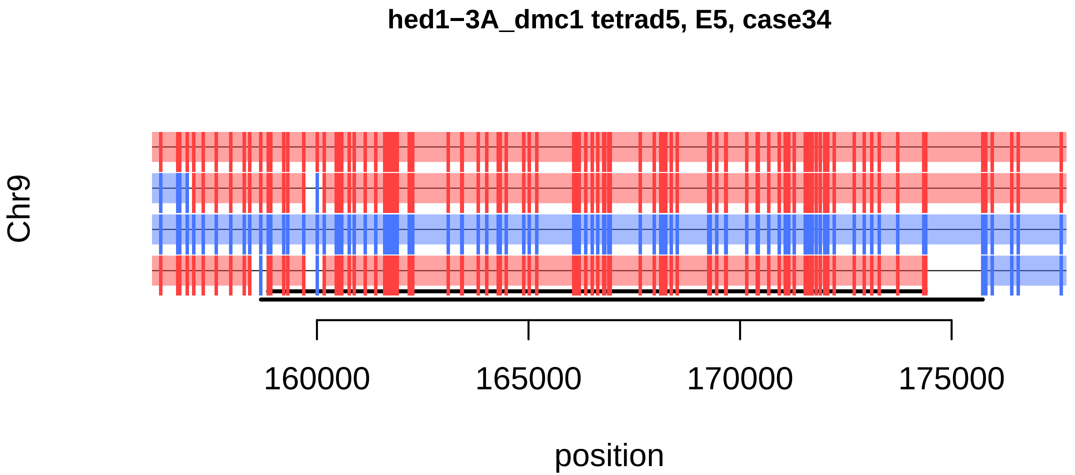
hed1−3A_dmc1 tetrad5, E5, case34
Chr9
160000
165000
170000
175000
position

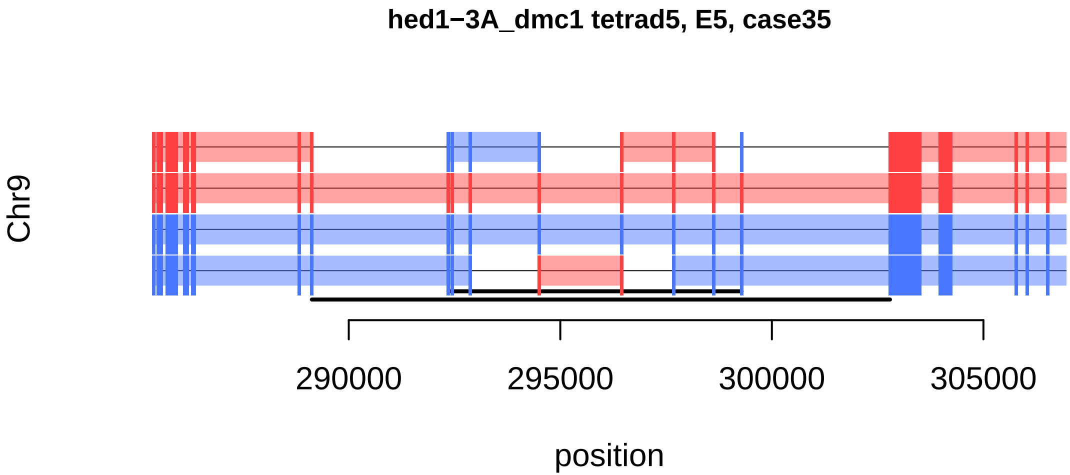

hed1−3A_dmc1 tetrad5, E5, case35
Chr9
290000
295000
300000
305000
position

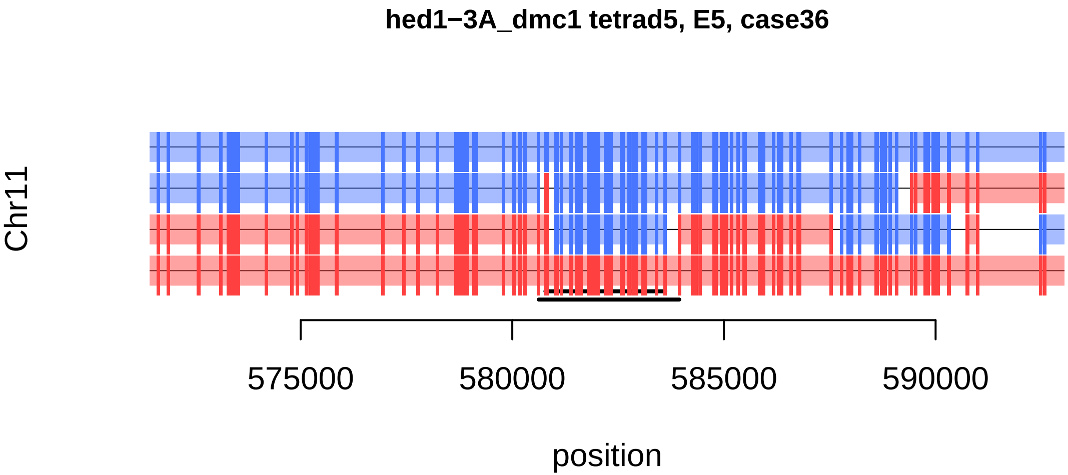
hed1−3A_dmc1 tetrad5, E5, case36
Chr11
575000
580000
585000
590000
position

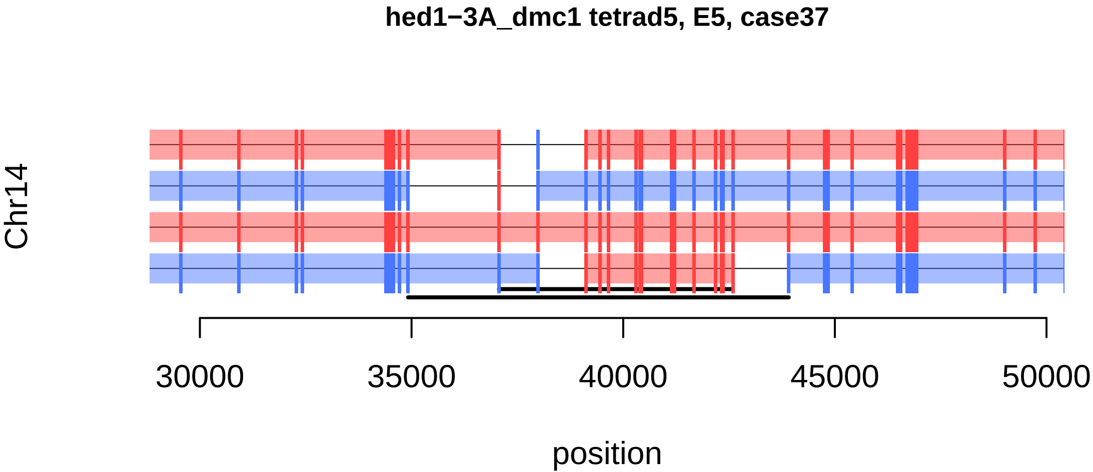
hed1−3A_dmc1 tetrad5, E5, case37
Chr14
30000
35000
40000
45000
50000
position

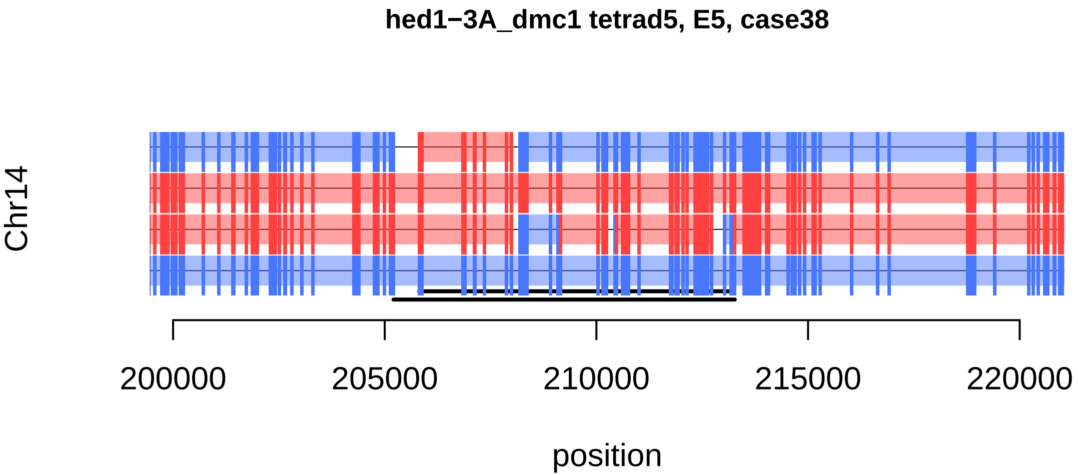
hed1−3A_dmc1 tetrad5, E5, case38
Chr14
200000
205000
210000
215000
220000
position

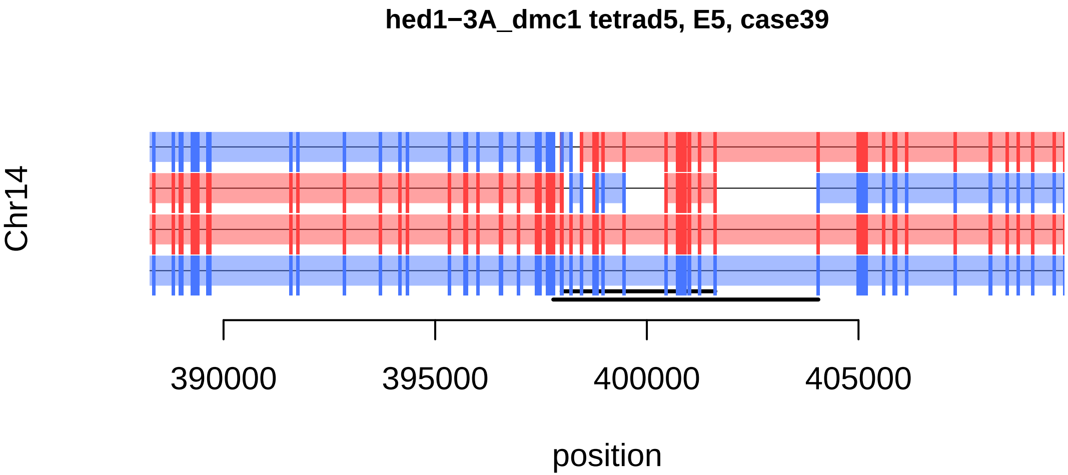

hed1−3A_dmc1 tetrad5, E5, case39
Chr14
390000
395000
400000
405000
position

hed1−3A_dmc1 tetrad5, E5, case40

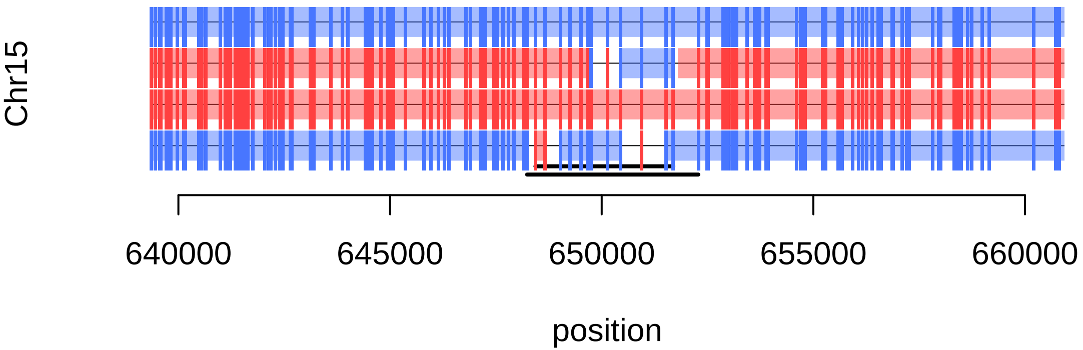

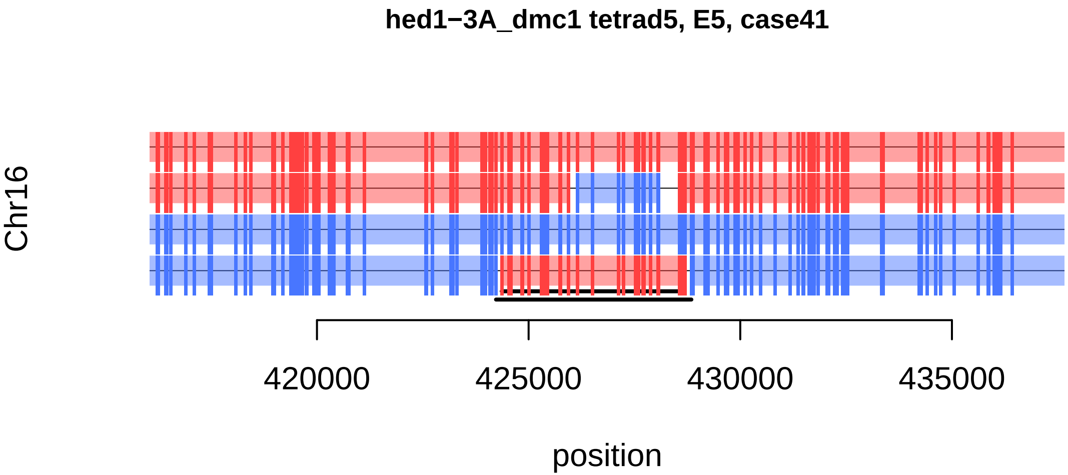
hed1−3A_dmc1 tetrad5, E5, case41
Chr16
420000
425000
430000
435000
position

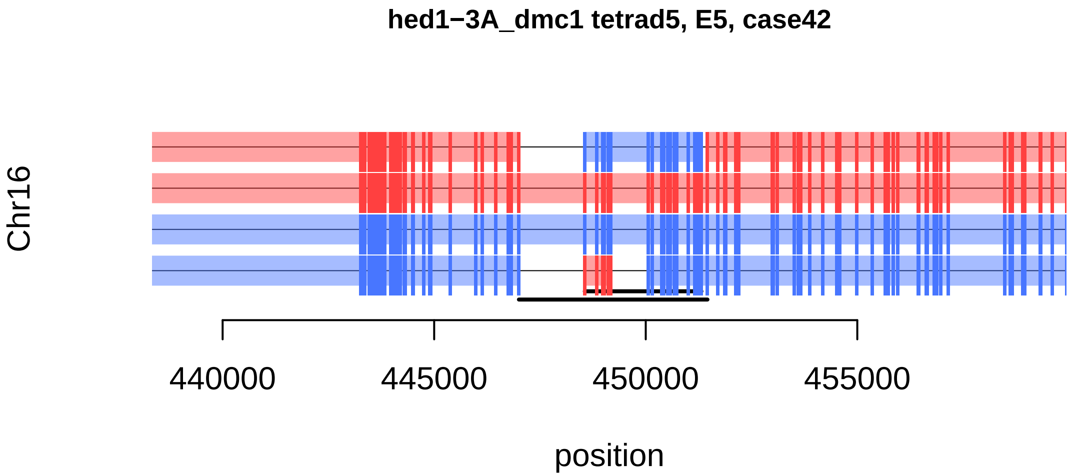
hed1−3A_dmc1 tetrad5, E5, case42
Chr16
440000
445000
450000
455000
position

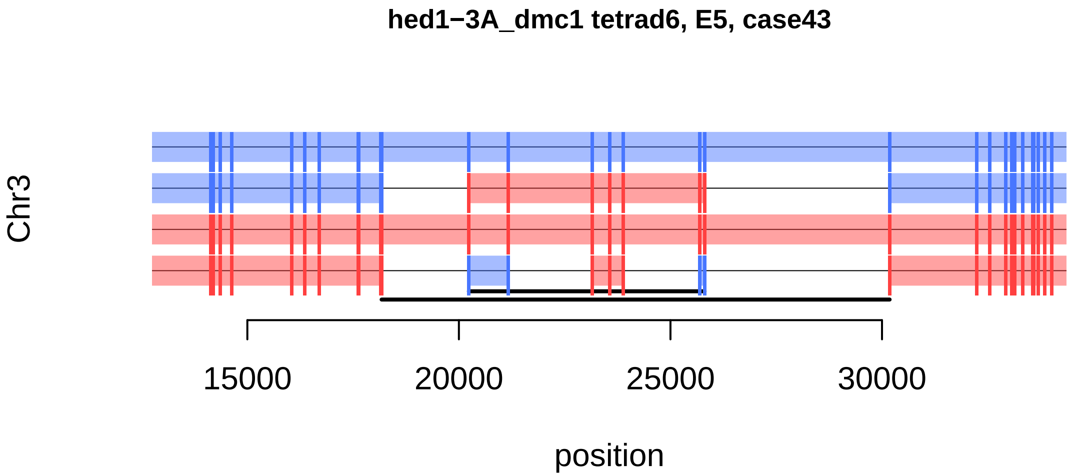
hed1−3A_dmc1 tetrad6, E5, case43
Chr3
15000
20000
25000
30000
position

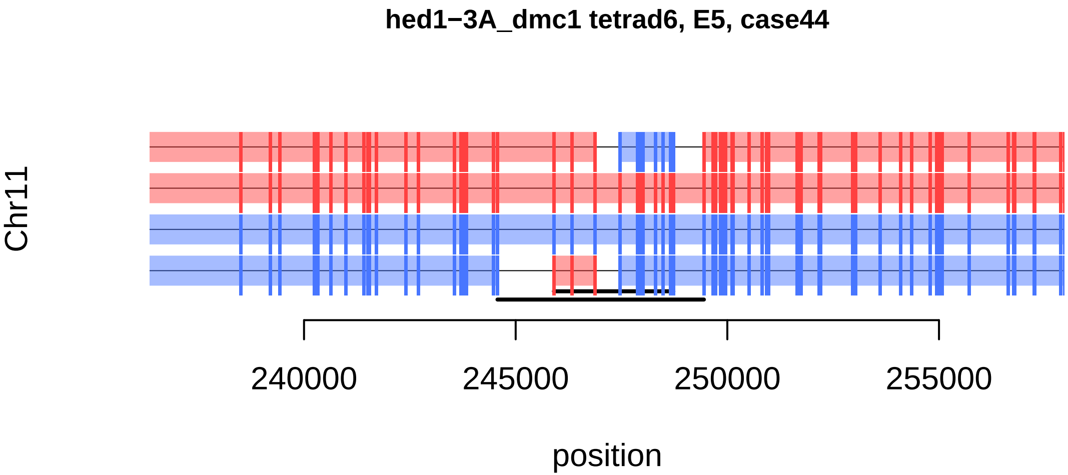

hed1−3A_dmc1 tetrad6, E5, case44
Chr11
240000
245000
250000
255000
position

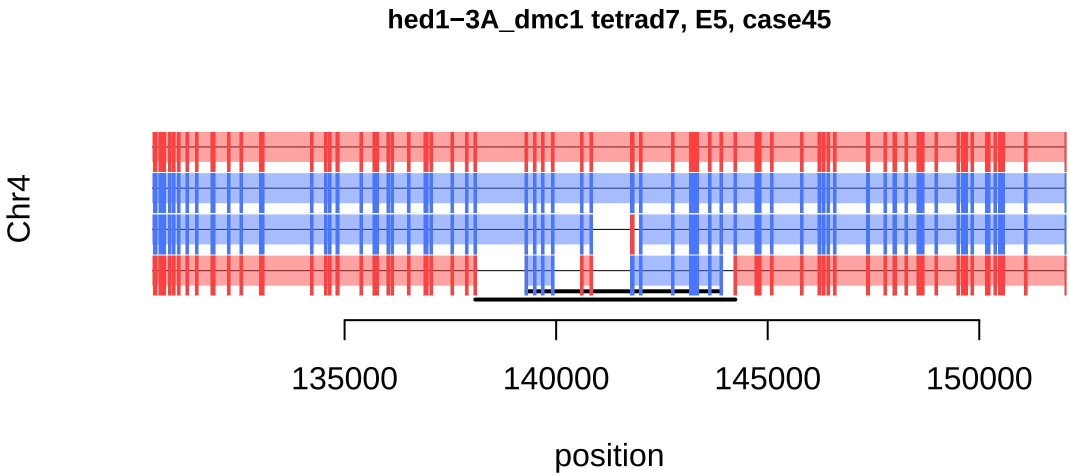
hed1−3A_dmc1 tetrad7, E5, case45
Chr4
135000
140000
145000
150000
position

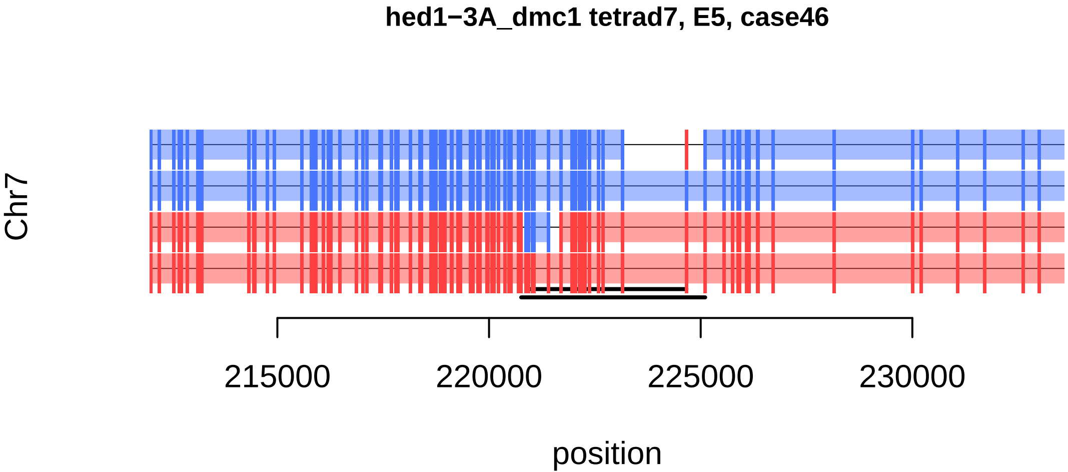
hed1−3A_dmc1 tetrad7, E5, case46
Chr7
215000
220000
225000
230000
position

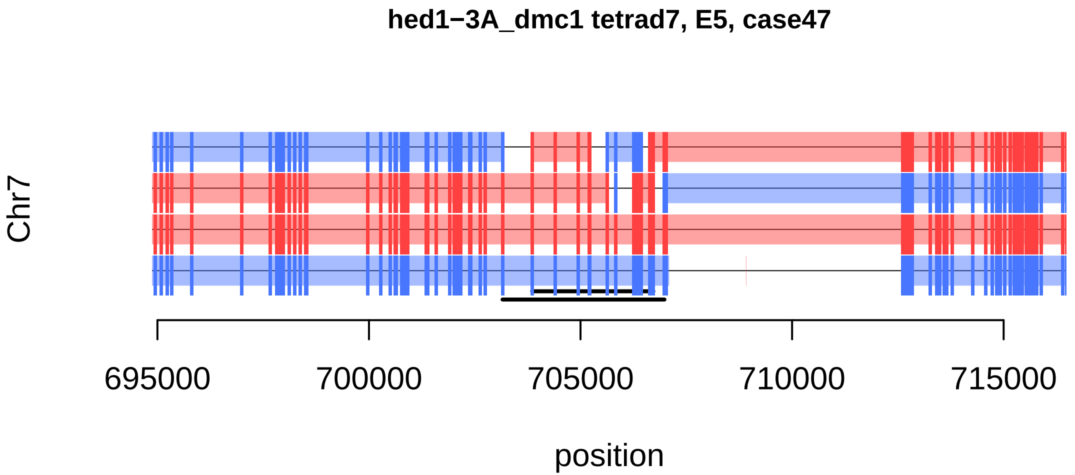
hed1−3A_dmc1 tetrad7, E5, case47
Chr7
695000
700000
705000
710000
715000
position

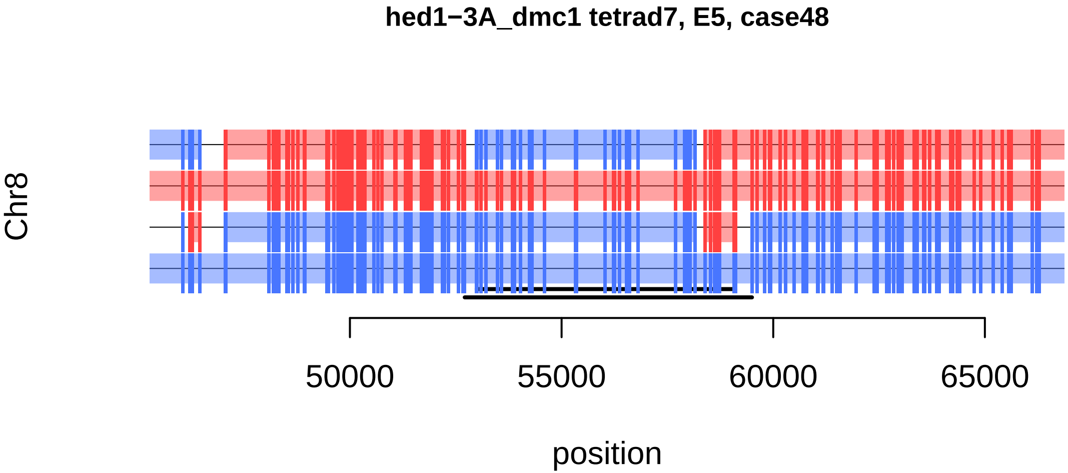
hed1−3A_dmc1 tetrad7, E5, case48
Chr8
50000
55000
60000
65000
position

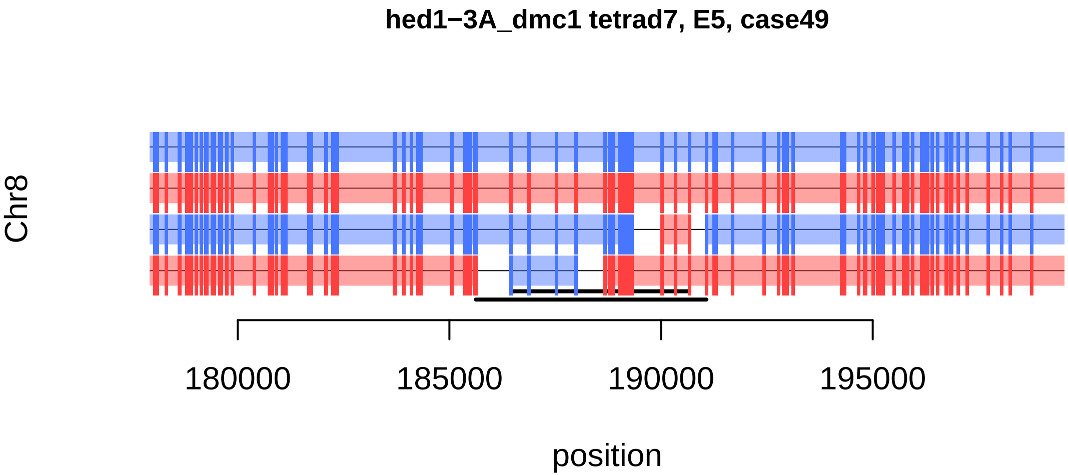
hed1−3A_dmc1 tetrad7, E5, case49
Chr8
180000
185000
190000
195000
position

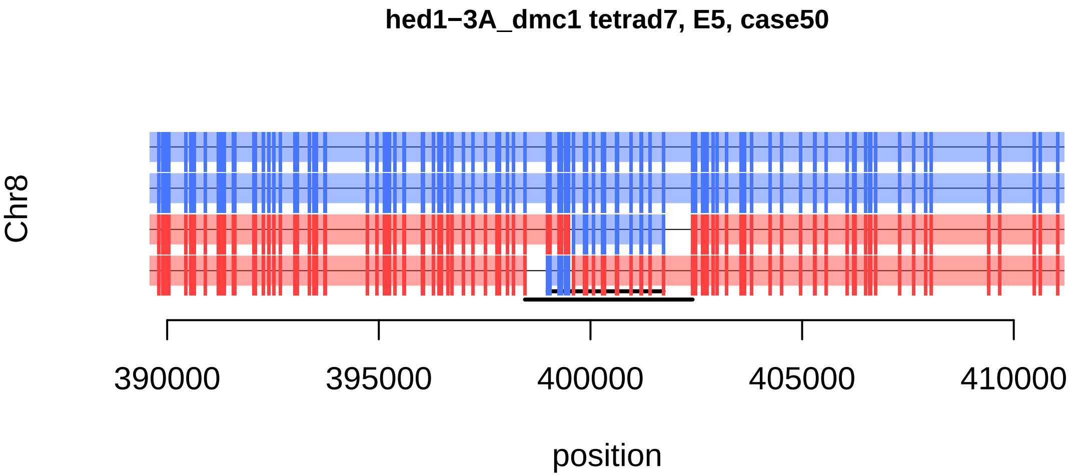
hed1−3A_dmc1 tetrad7, E5, case50
Chr8
390000
395000
400000
405000
410000
position

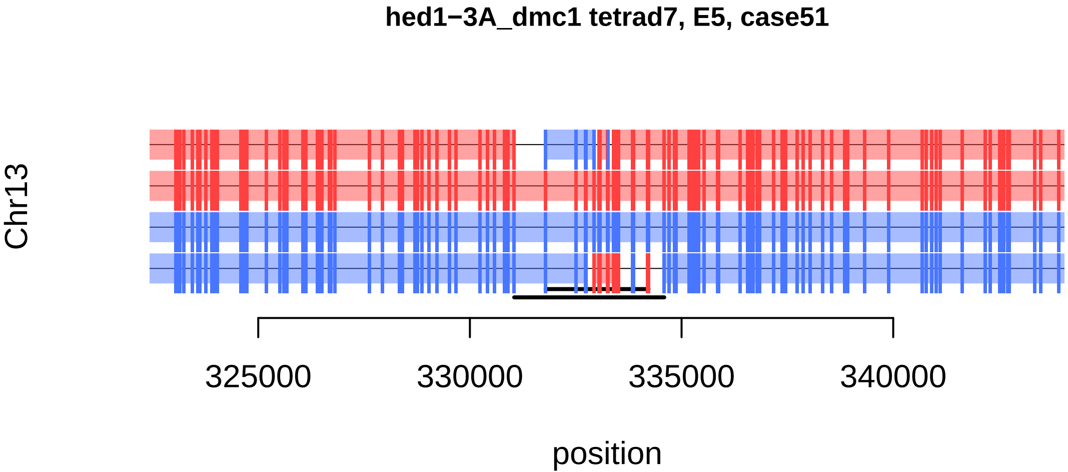
hed1−3A_dmc1 tetrad7, E5, case51
Chr13
325000
330000
335000
340000
position

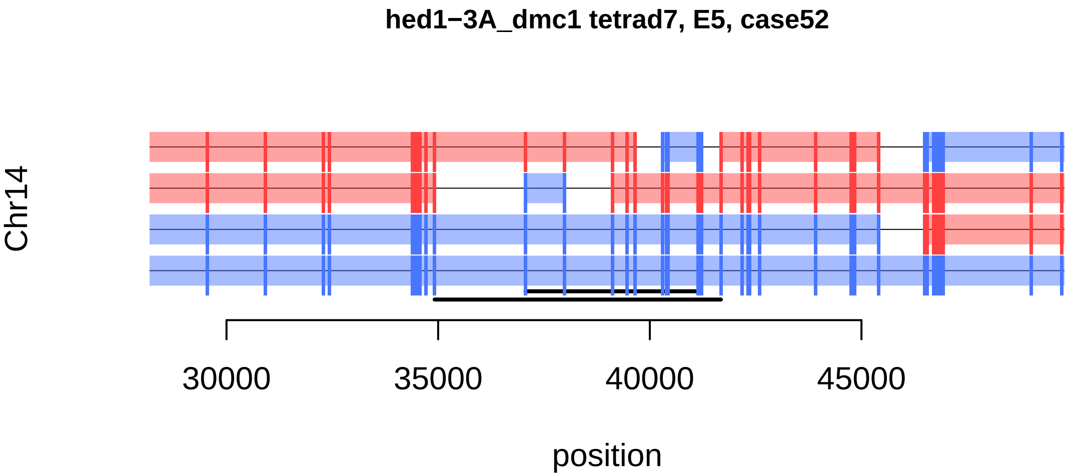
hed1−3A_dmc1 tetrad7, E5, case52
Chr14
30000
35000
40000
45000
position

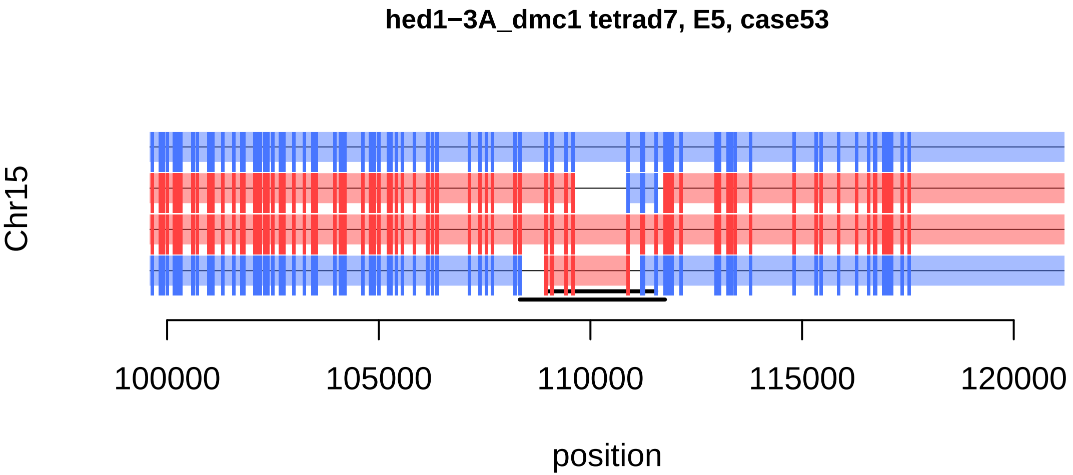
hed1−3A_dmc1 tetrad7, E5, case53
Chr15
100000
105000
110000
115000
120000
position

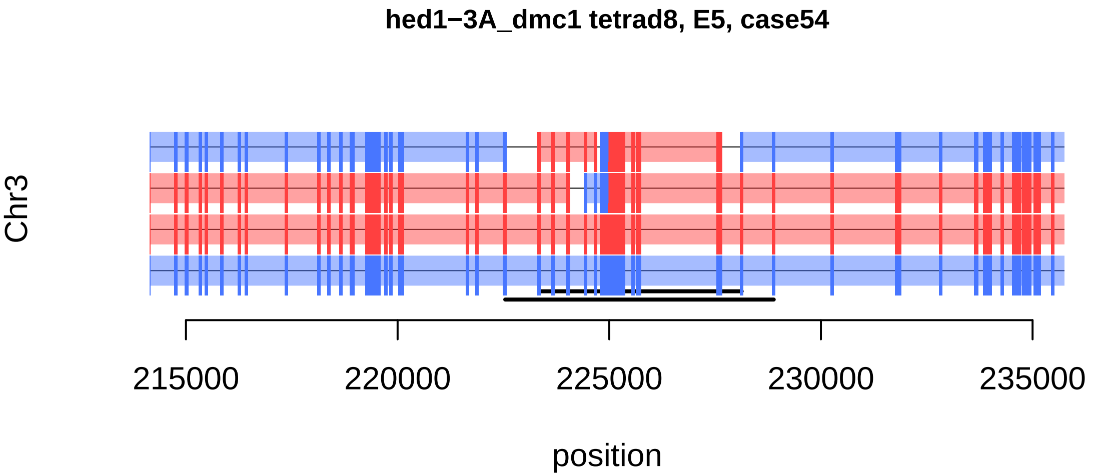
hed1−3A_dmc1 tetrad8, E5, case54
Chr3
215000
220000
225000
230000
235000
position

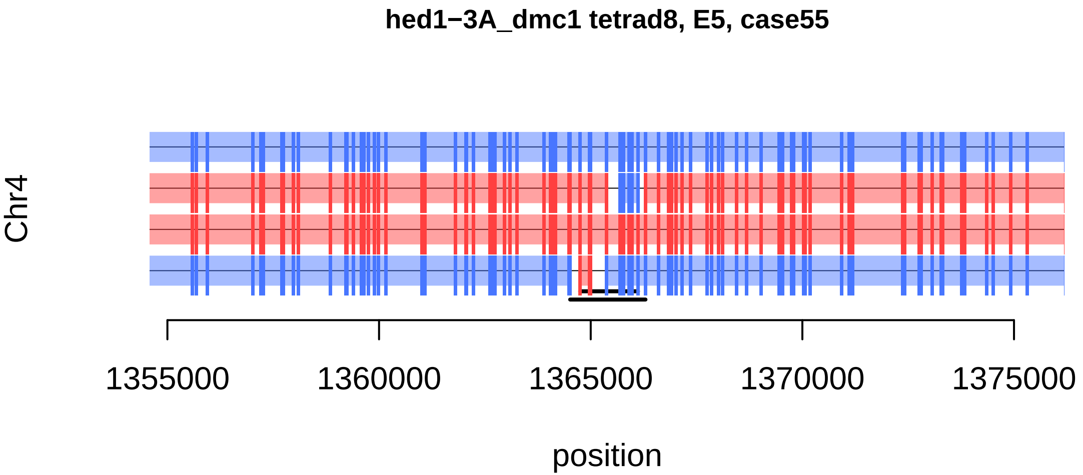
hed1−3A_dmc1 tetrad8, E5, case55
Chr4
1355000
1360000
1365000
1370000
1375000
position

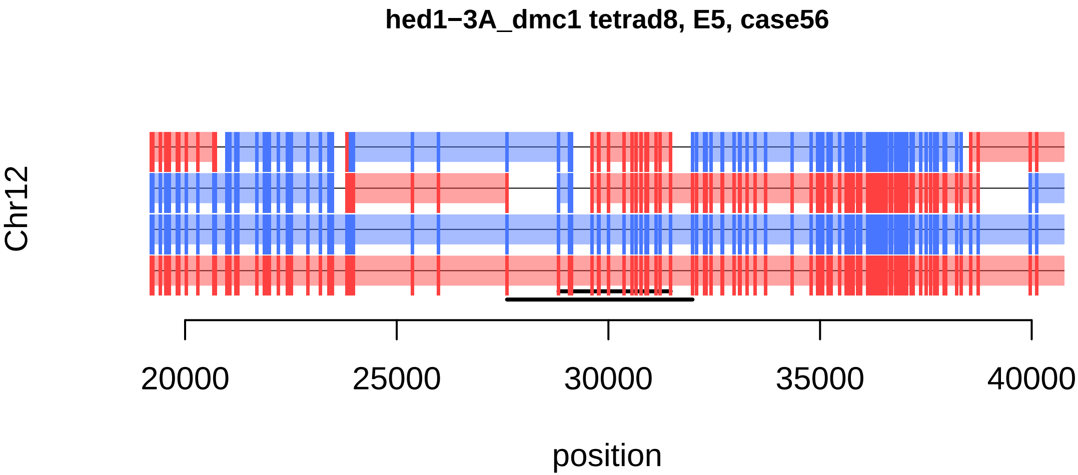

hed1−3A_dmc1 tetrad8, E5, case56
Chr12
20000
25000
30000
35000
40000
position

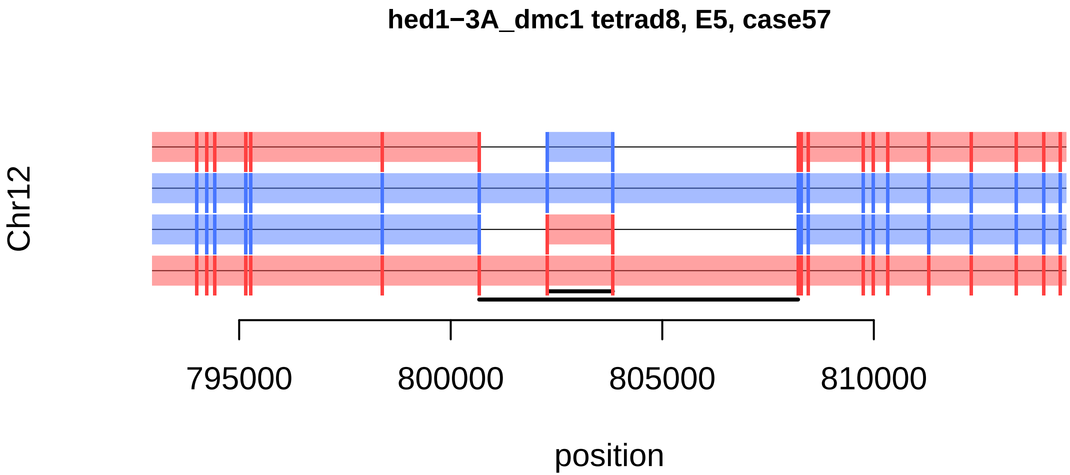

hed1−3A_dmc1 tetrad8, E5, case57
Chr12
795000
800000
805000
810000
position

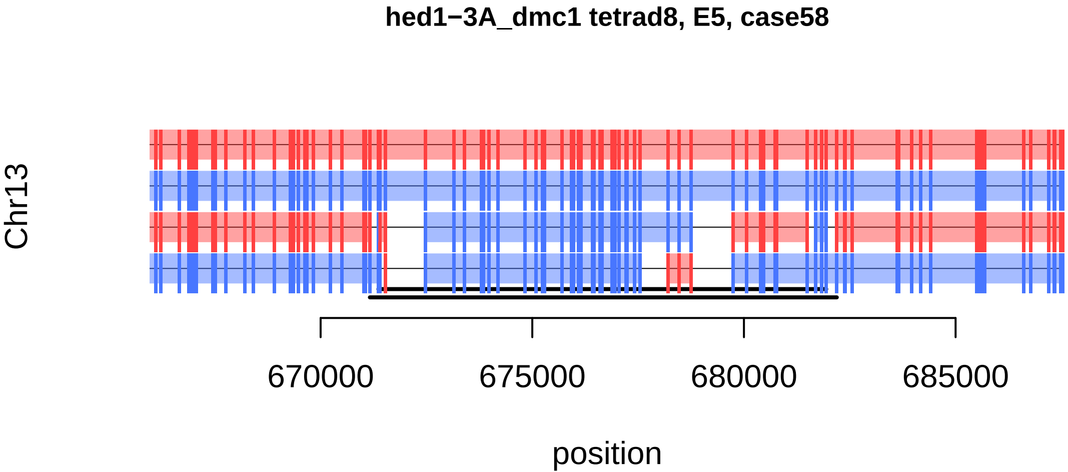
hed1−3A_dmc1 tetrad8, E5, case58
Chr13
670000
675000
680000
685000
position

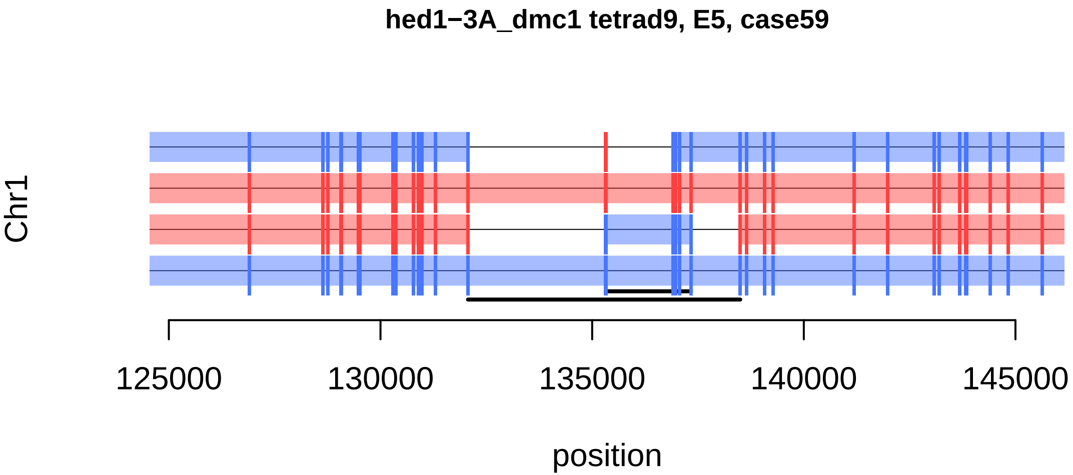
hed1−3A_dmc1 tetrad9, E5, case59
Chr1
125000
130000
135000
140000
145000
position

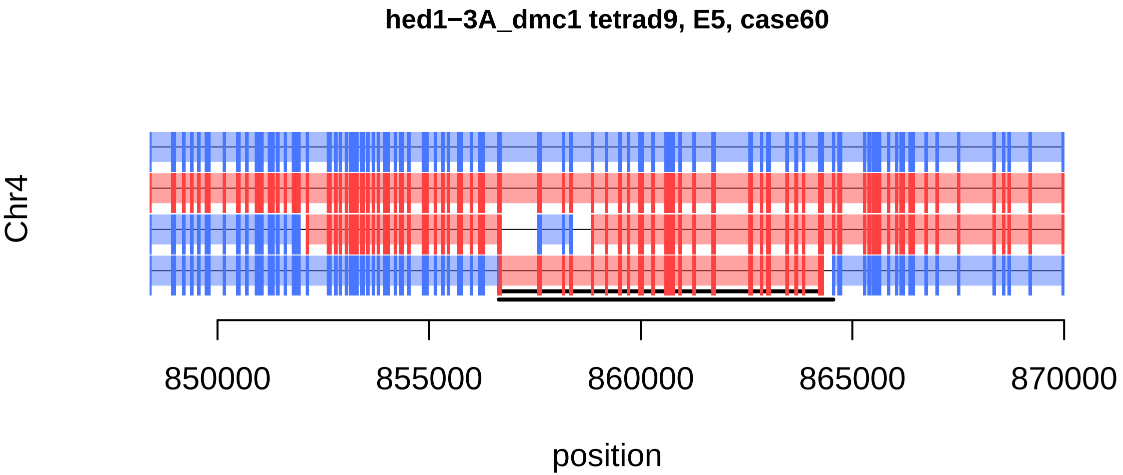
hed1−3A_dmc1 tetrad9, E5, case60
Chr4
850000
855000
860000
865000
870000
position

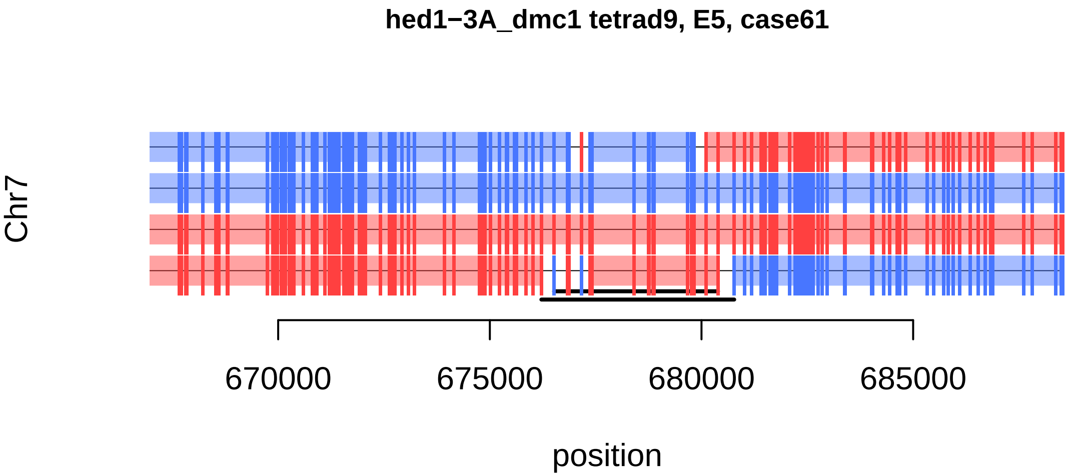
hed1−3A_dmc1 tetrad9, E5, case61
Chr7
670000
675000
680000
685000
position

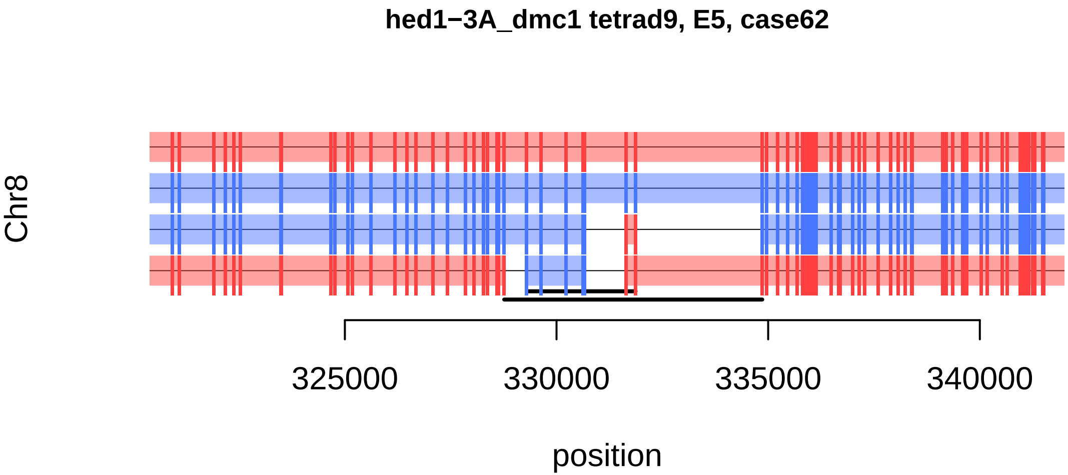

hed1−3A_dmc1 tetrad9, E5, case62
Chr8
325000
330000
335000
340000
position

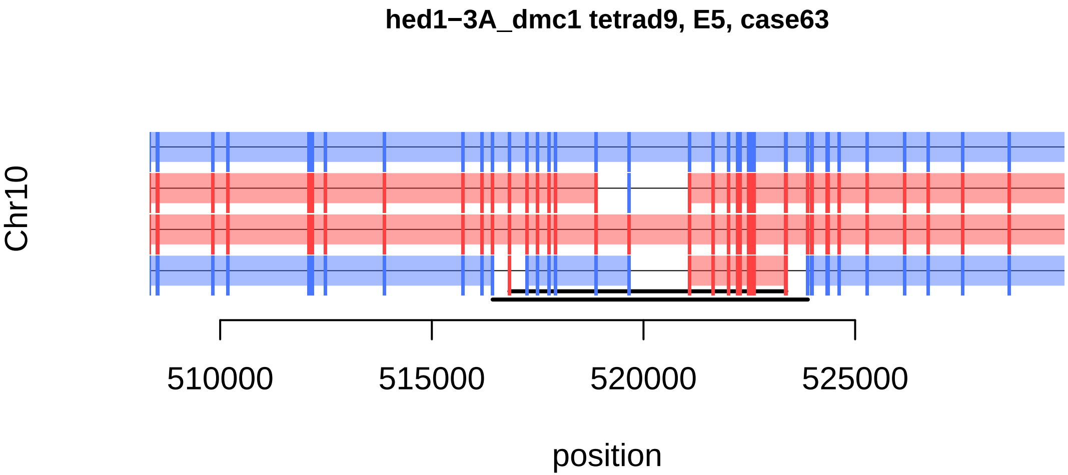
hed1−3A_dmc1 tetrad9, E5, case63
Chr10
510000
515000
520000
525000
position

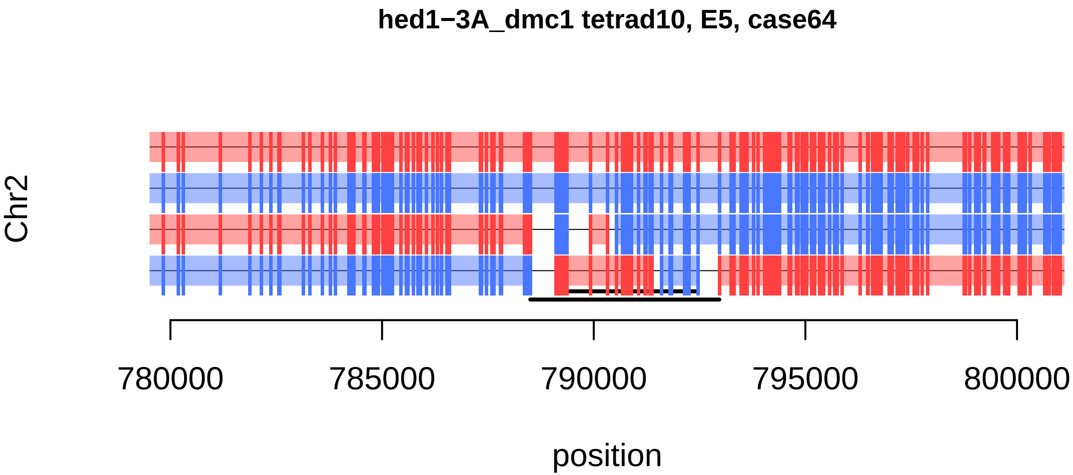

hed1−3A_dmc1 tetrad10, E5, case64
Chr2
780000
785000
790000
795000
800000
position

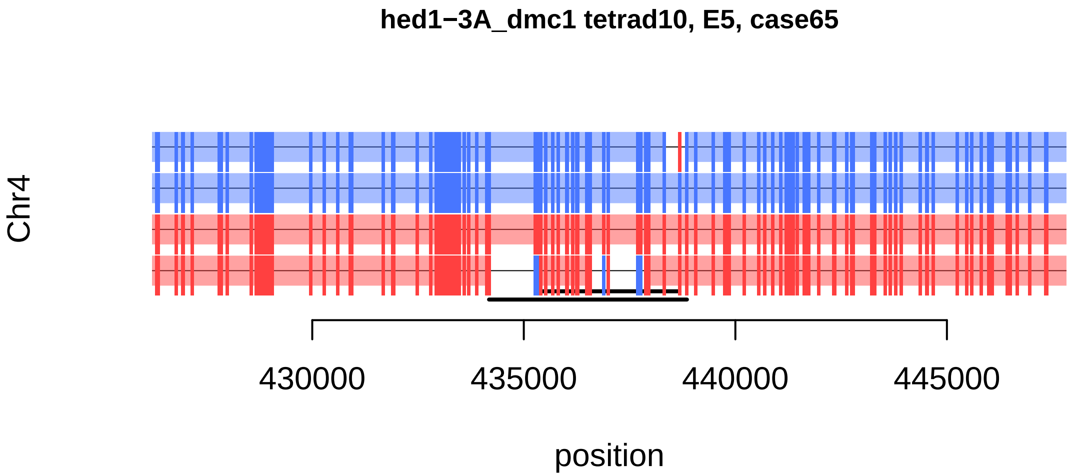
hed1−3A_dmc1 tetrad10, E5, case65
Chr4
430000
435000
440000
445000
position

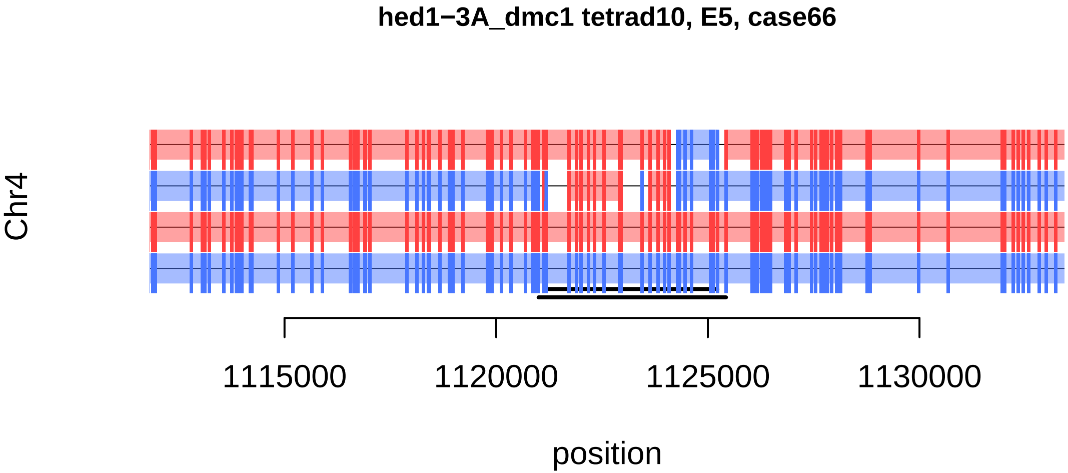
hed1−3A_dmc1 tetrad10, E5, case66
Chr4
1115000
1120000
1125000
1130000
position

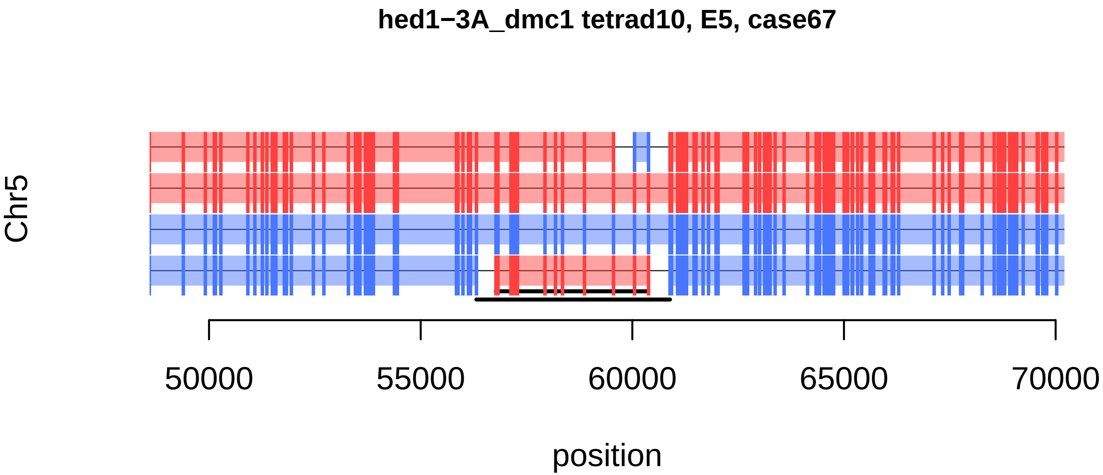

hed1−3A_dmc1 tetrad10, E5, case67
Chr5
50000
55000
60000
65000
70000
position

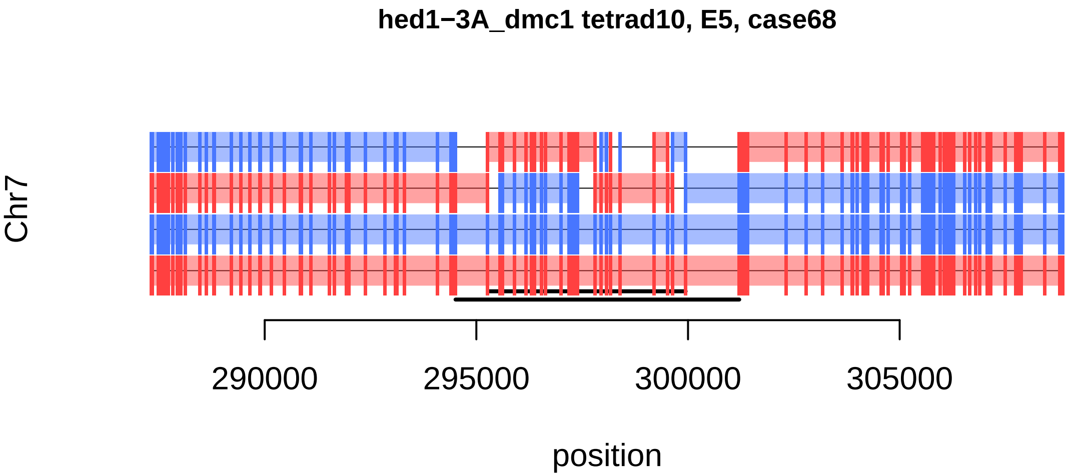
hed1−3A_dmc1 tetrad10, E5, case68
Chr7
290000
295000
300000
305000
position

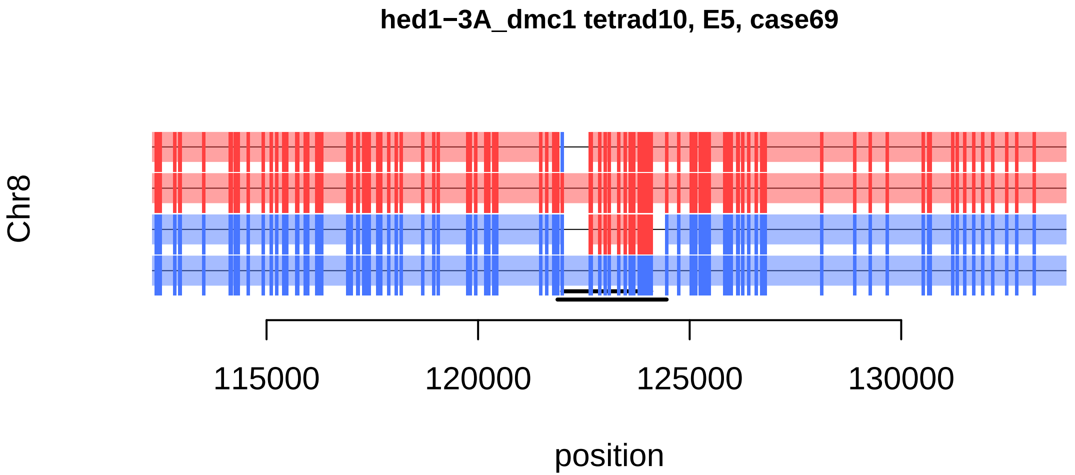
hed1−3A_dmc1 tetrad10, E5, case69
Chr8
115000
120000
125000
130000
position

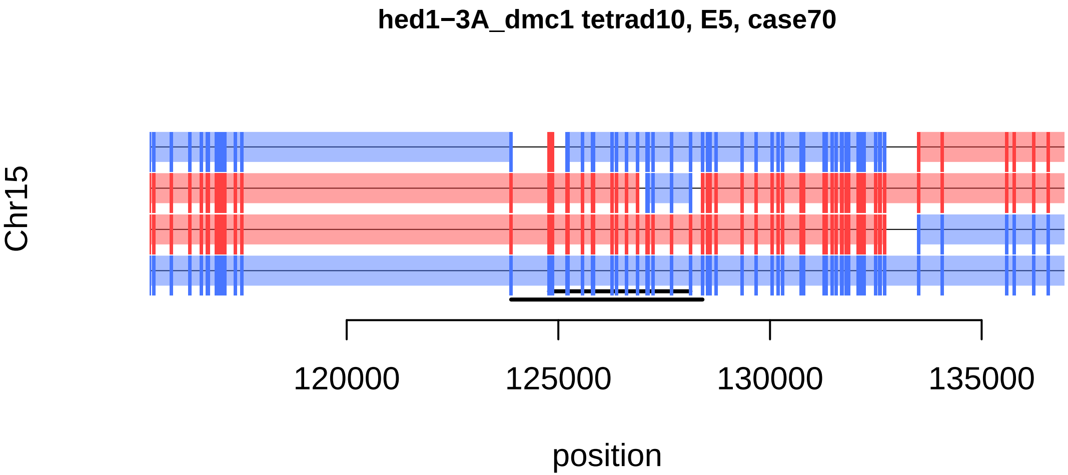
hed1−3A_dmc1 tetrad10, E5, case70
Chr15
120000
125000
130000
135000
position

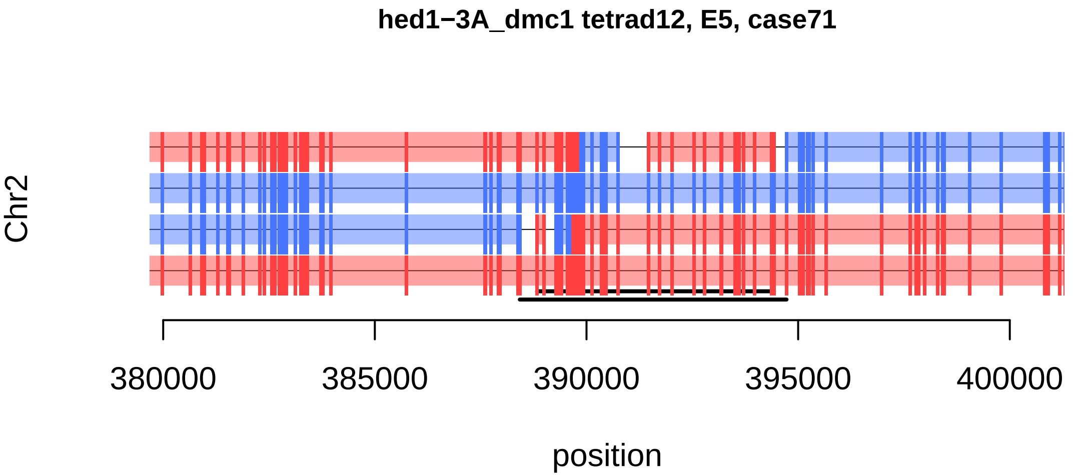
hed1−3A_dmc1 tetrad12, E5, case71
Chr2
380000
385000
390000
395000
400000
position

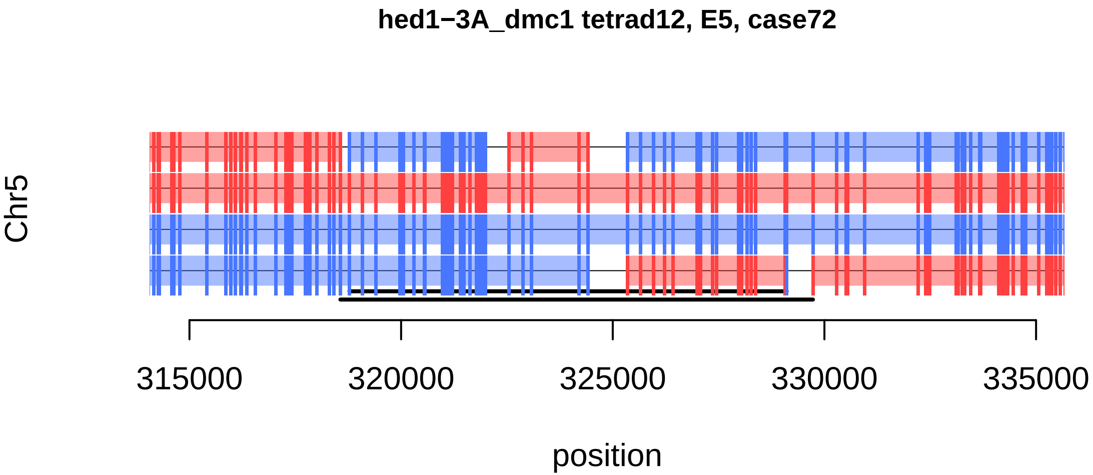
hed1−3A_dmc1 tetrad12, E5, case72
Chr5
315000
320000
325000
330000
335000
position

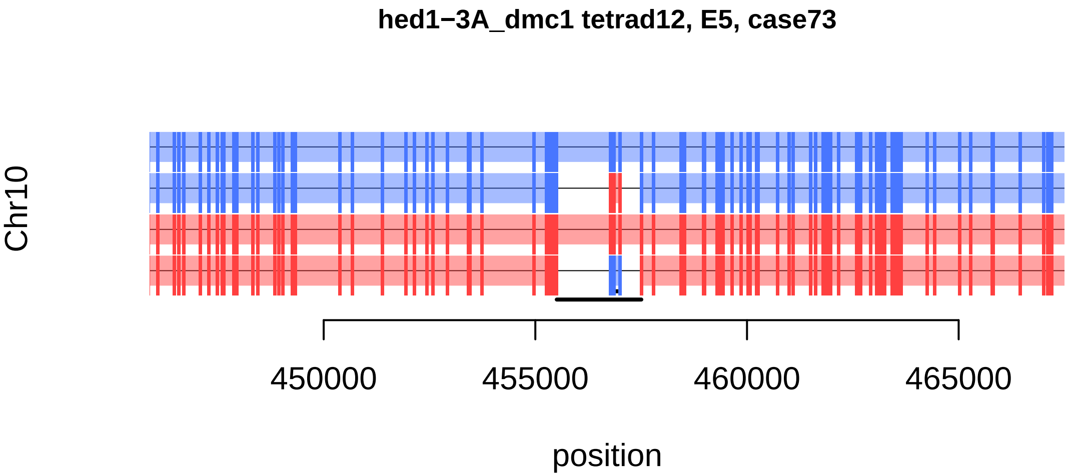
hed1−3A_dmc1 tetrad12, E5, case73
Chr10
450000
455000
460000
465000
position

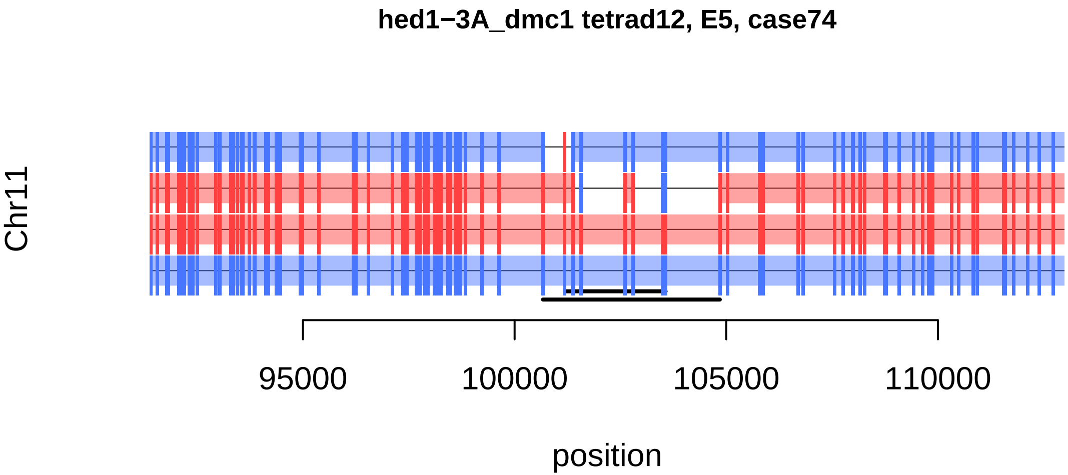
hed1−3A_dmc1 tetrad12, E5, case74
Chr11
95000
100000
105000
110000
position

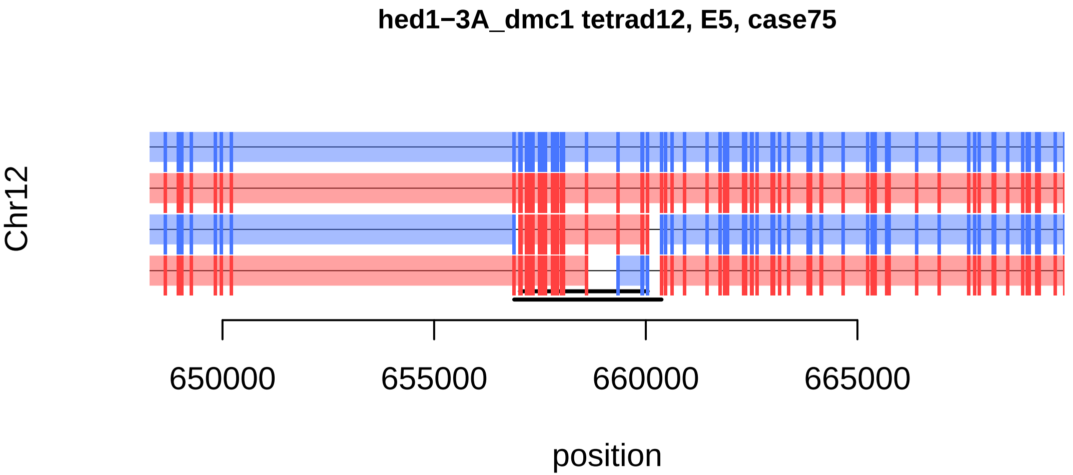
hed1−3A_dmc1 tetrad12, E5, case75
Chr12
650000
655000
660000
665000
position

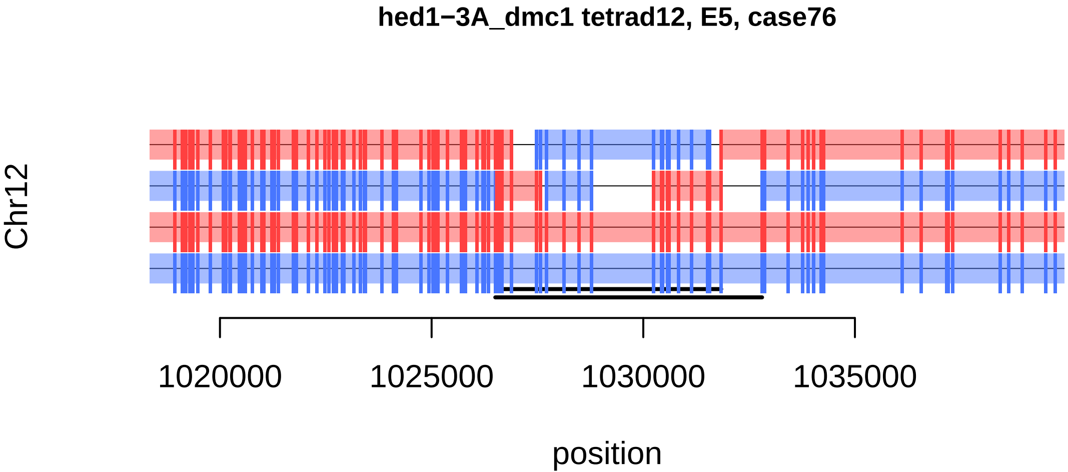

hed1−3A_dmc1 tetrad12, E5, case76
Chr12
1020000
1025000
1030000
1035000
position

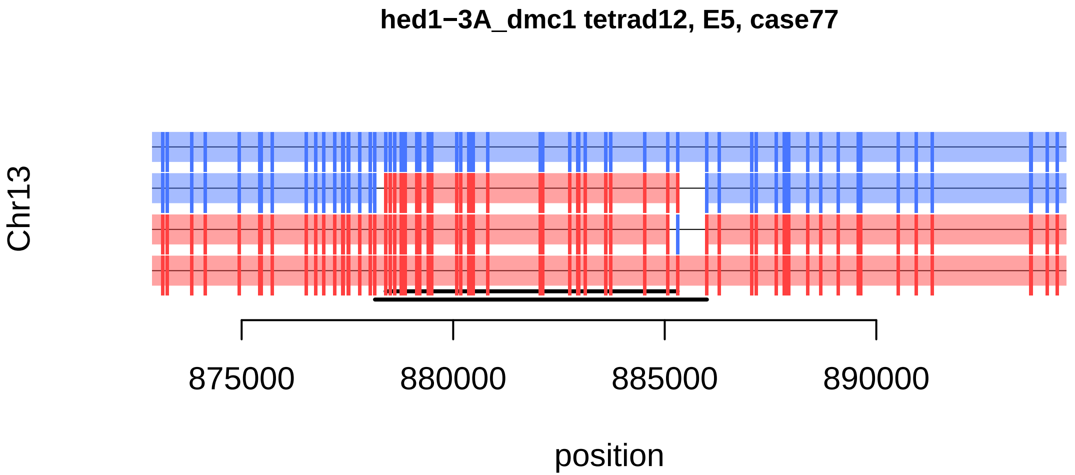
hed1−3A_dmc1 tetrad12, E5, case77
Chr13
875000
880000
885000
890000
position

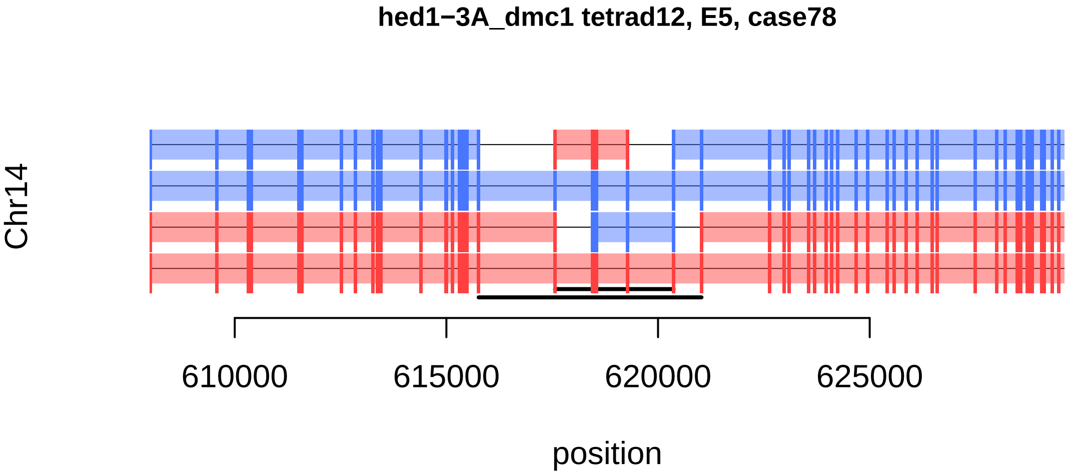
hed1−3A_dmc1 tetrad12, E5, case78
Chr14
610000
615000
620000
625000
position

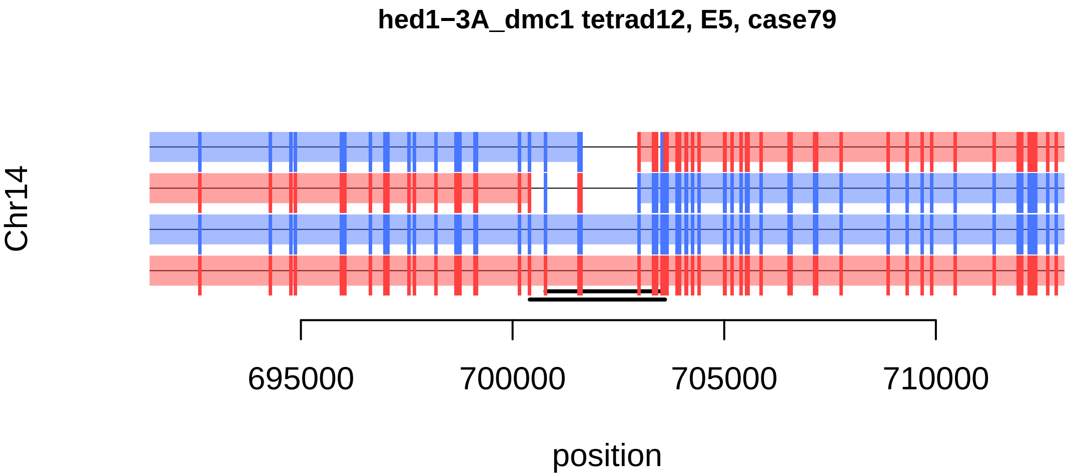
hed1−3A_dmc1 tetrad12, E5, case79
Chr14
695000
700000
705000
710000
position

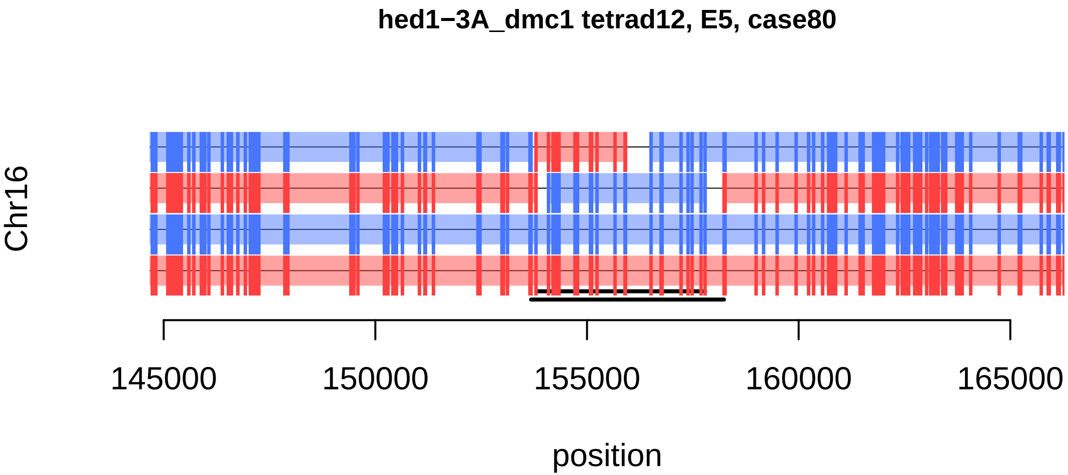
hed1−3A_dmc1 tetrad12, E5, case80
Chr16
145000
150000
155000
160000
165000
position

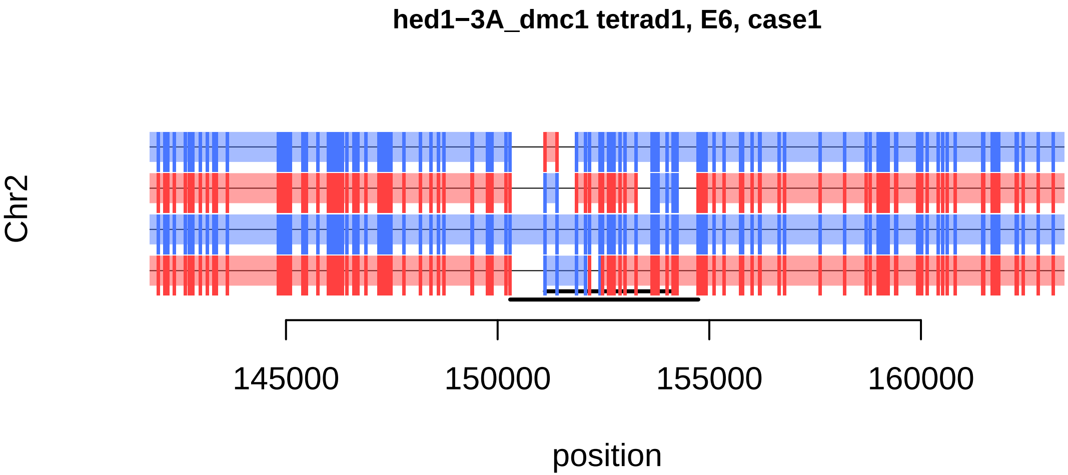

hed1−3A_dmc1 tetrad1, E6, case1
Chr2
145000
150000
155000
160000
position

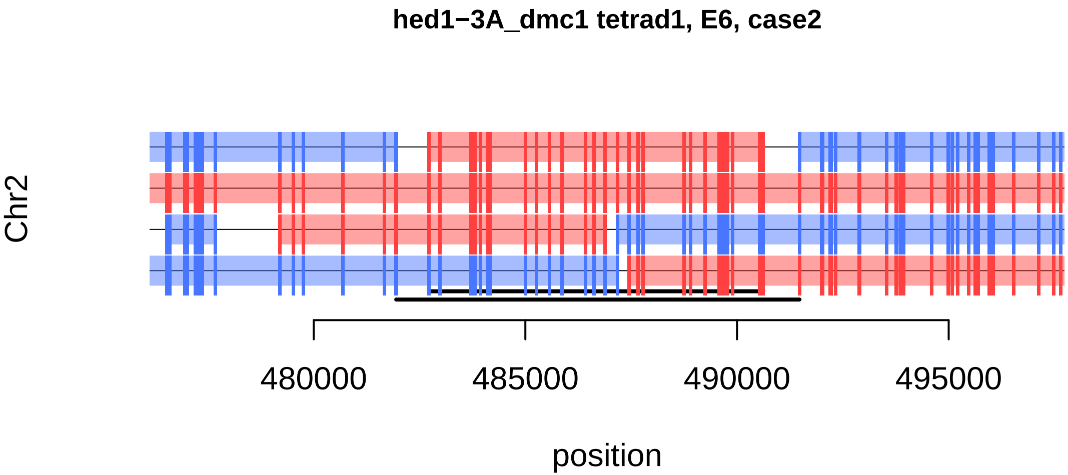

hed1−3A_dmc1 tetrad1, E6, case2
Chr2
480000
485000
490000
495000
position

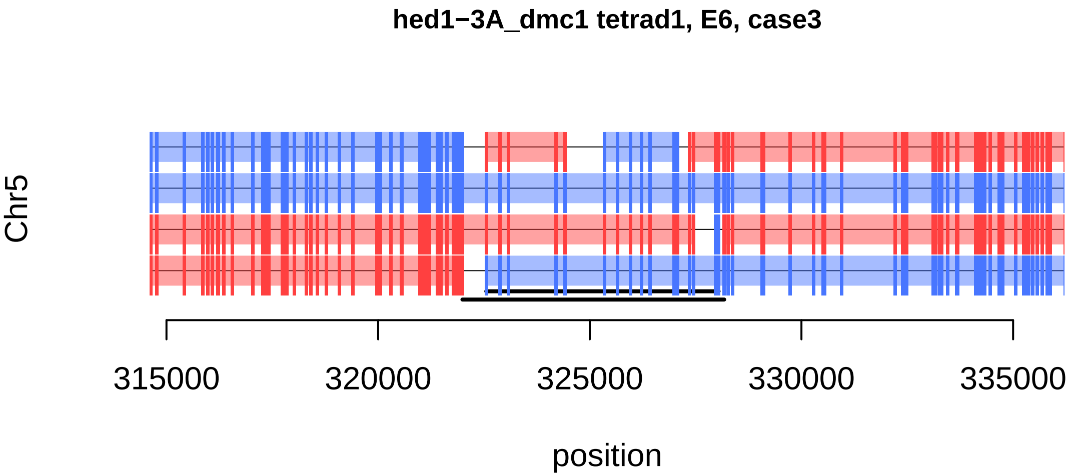
hed1−3A_dmc1 tetrad1, E6, case3
Chr5
315000
320000
325000
330000
335000
position

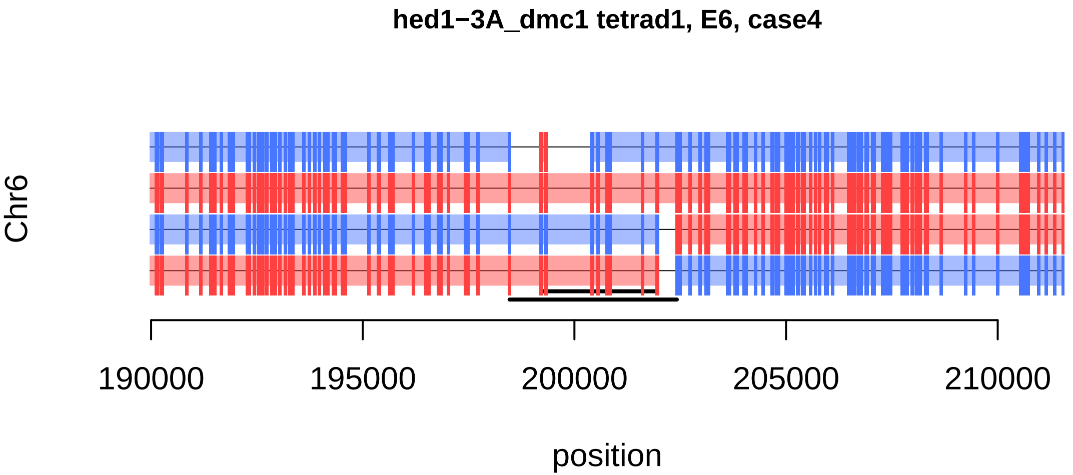
hed1−3A_dmc1 tetrad1, E6, case4
Chr6
190000
195000
200000
205000
210000
position

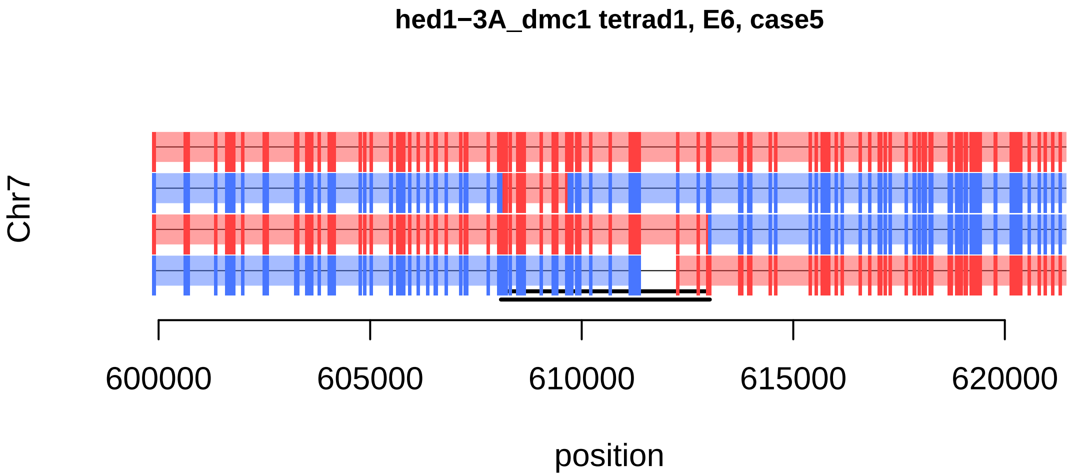
hed1−3A_dmc1 tetrad1, E6, case5
Chr7
600000
605000
610000
615000
620000
position

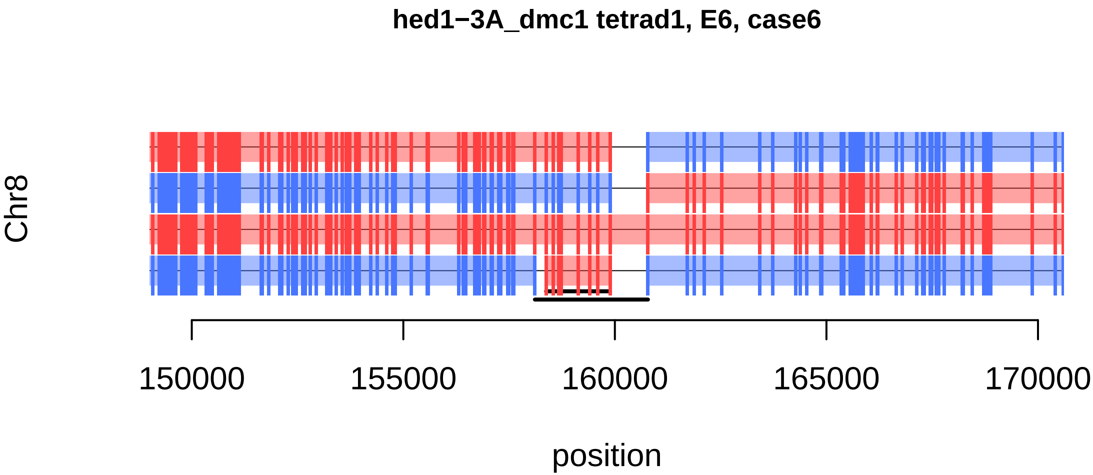
hed1−3A_dmc1 tetrad1, E6, case6
Chr8
150000
155000
160000
165000
170000
position

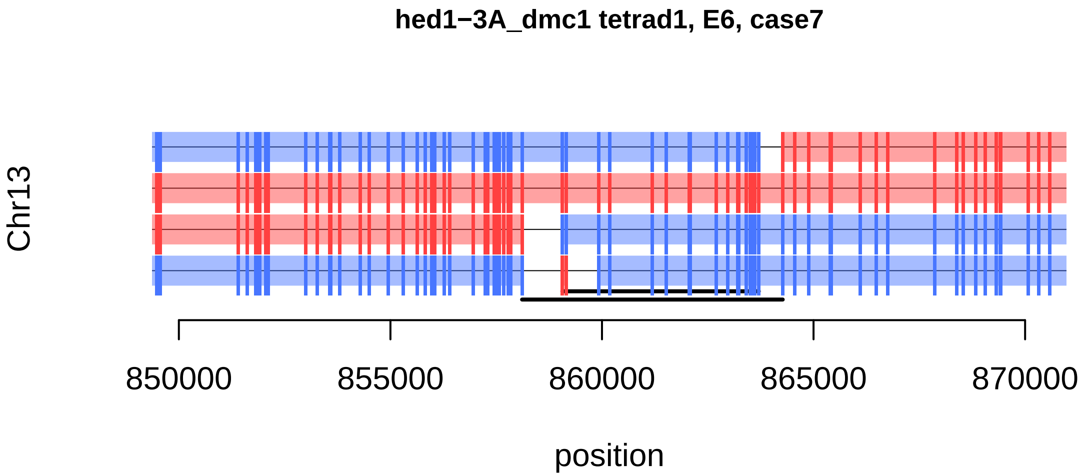
hed1−3A_dmc1 tetrad1, E6, case7
Chr13
850000
855000
860000
865000
870000
position

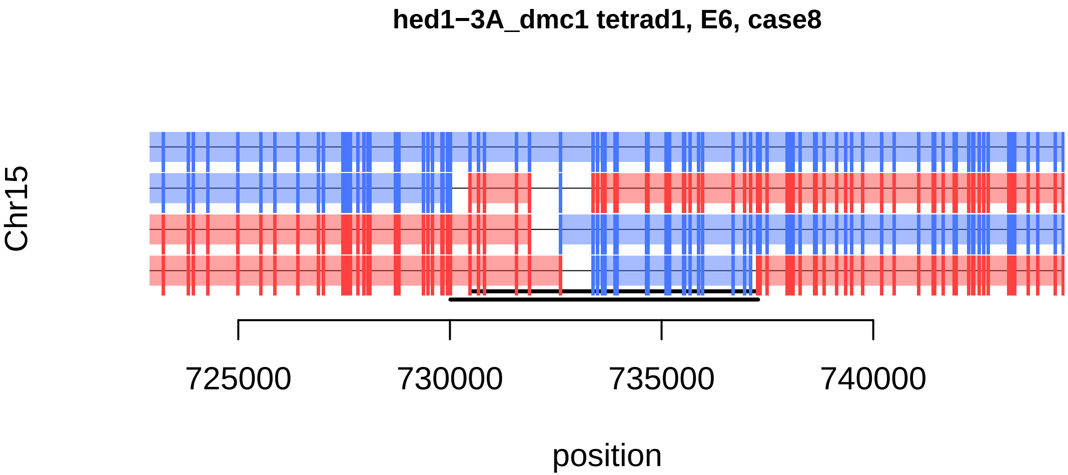
hed1−3A_dmc1 tetrad1, E6, case8
Chr15
725000
730000
735000
740000
position

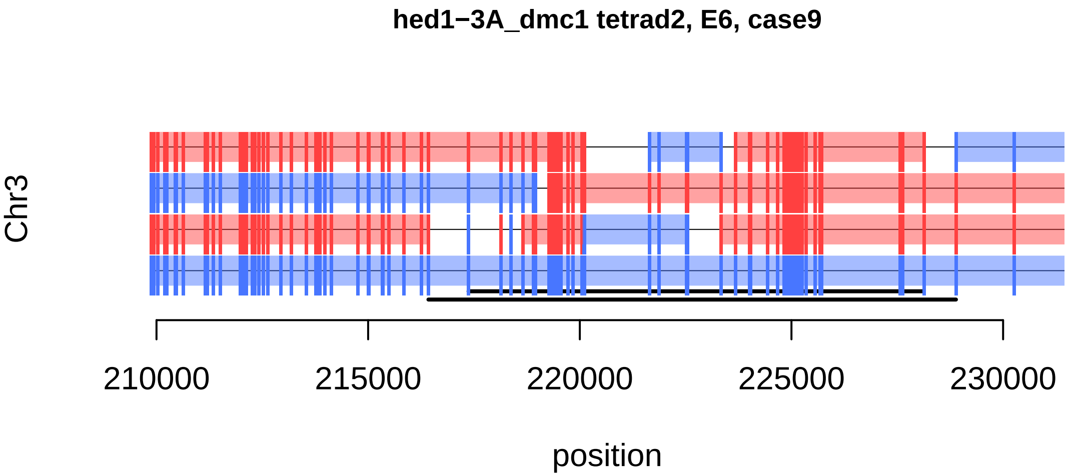

hed1−3A_dmc1 tetrad2, E6, case9
Chr3
210000
215000
220000
225000
230000
position

hed1−3A_dmc1 tetrad2, E6, case10

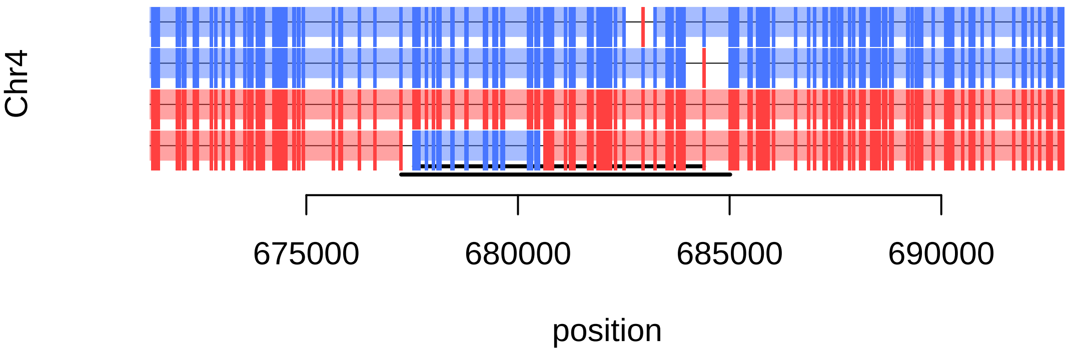

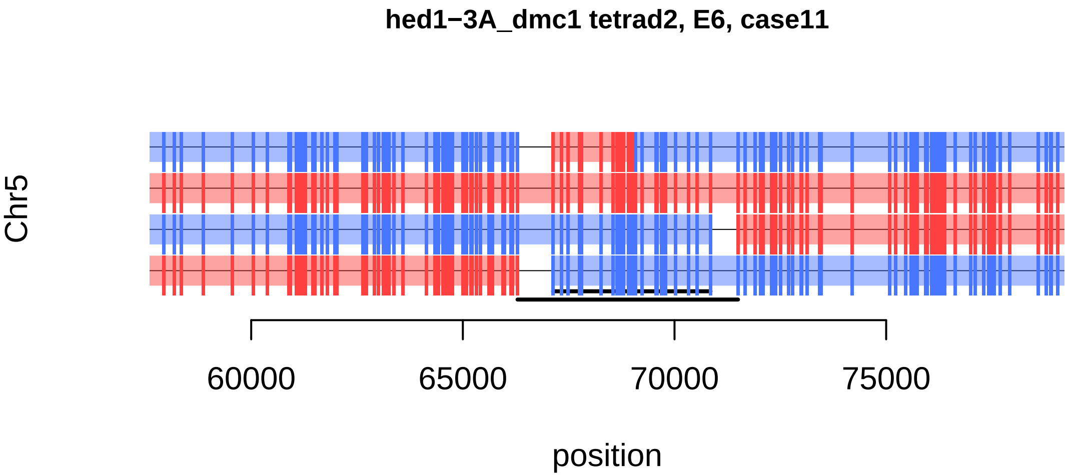
hed1−3A_dmc1 tetrad2, E6, case11
Chr5
60000
65000
70000
75000
position

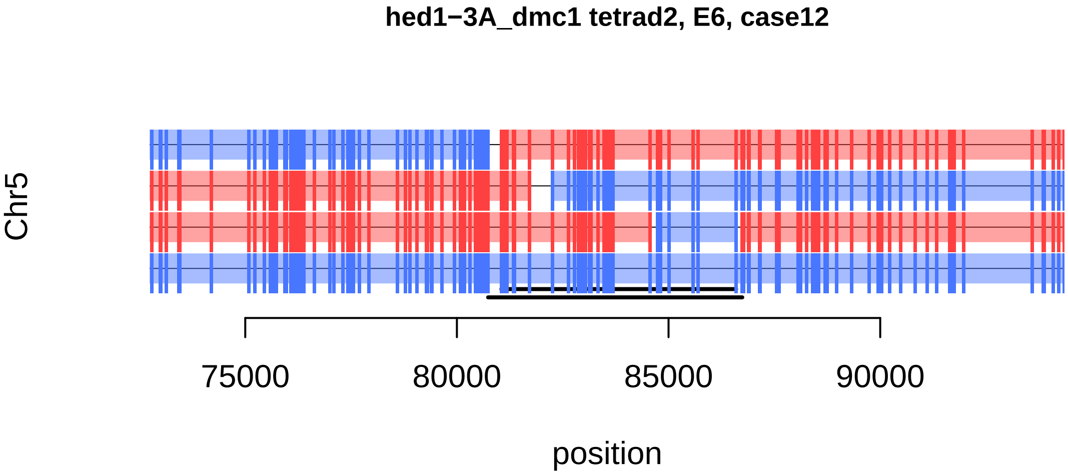

hed1−3A_dmc1 tetrad2, E6, case12
Chr5
75000
80000
85000
90000
position

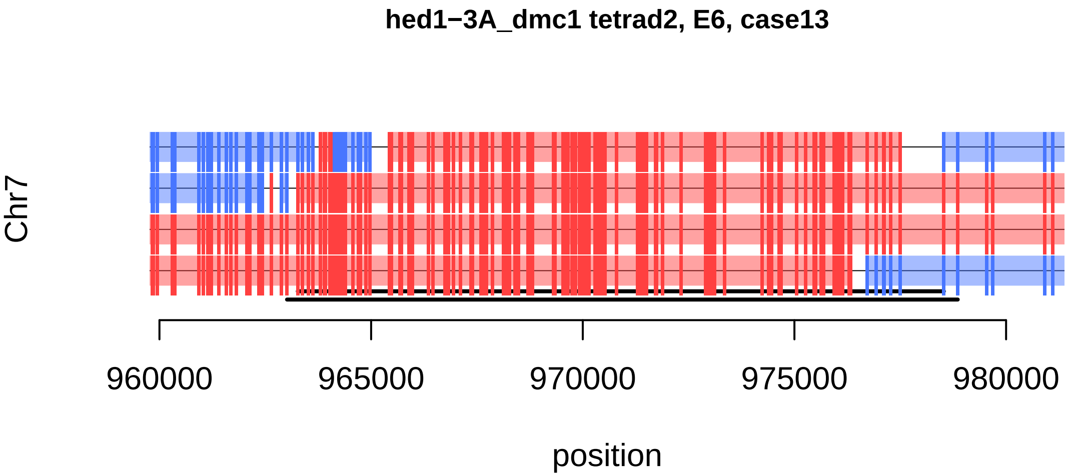
hed1−3A_dmc1 tetrad2, E6, case13
Chr7
960000
965000
970000
975000
980000
position

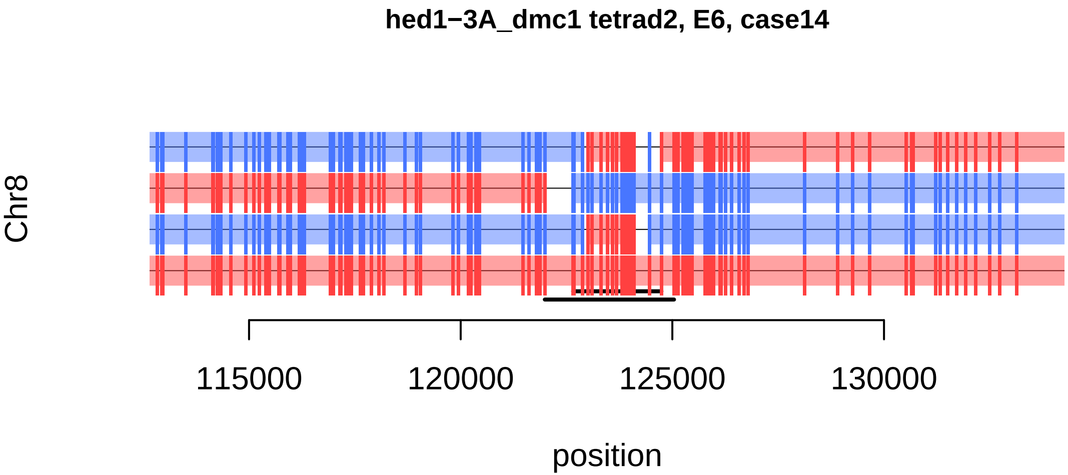

hed1−3A_dmc1 tetrad2, E6, case14
Chr8
115000
120000
125000
130000
position

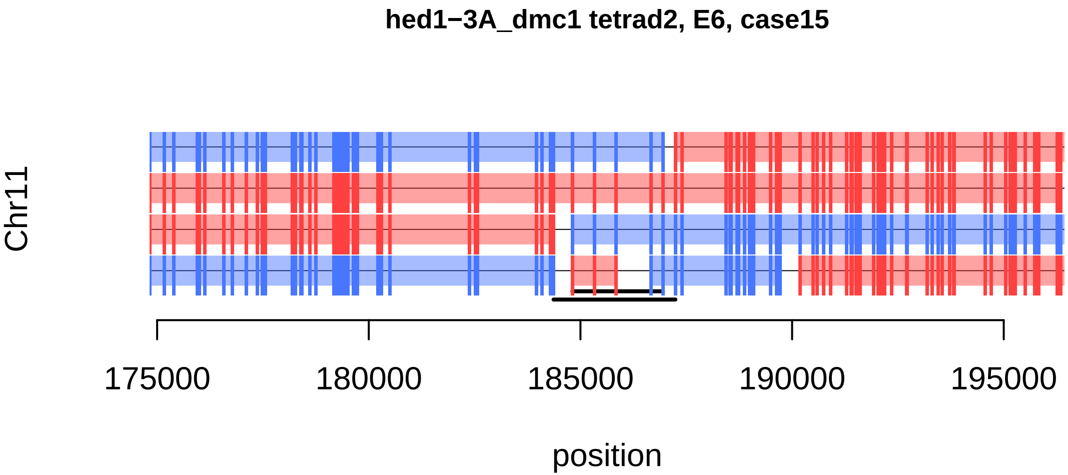
hed1−3A_dmc1 tetrad2, E6, case15
Chr11
175000
180000
185000
190000
195000
position

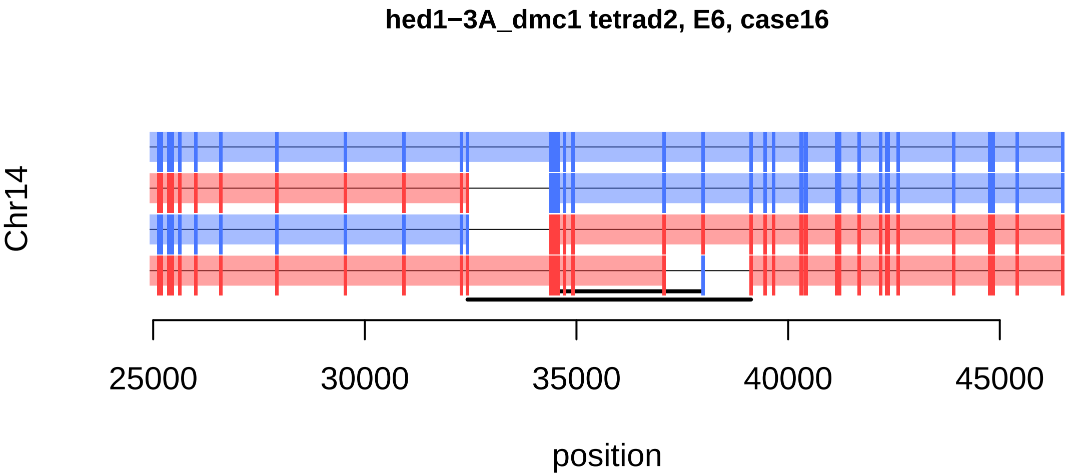
hed1−3A_dmc1 tetrad2, E6, case16
Chr14
25000
30000
35000
40000
45000
position

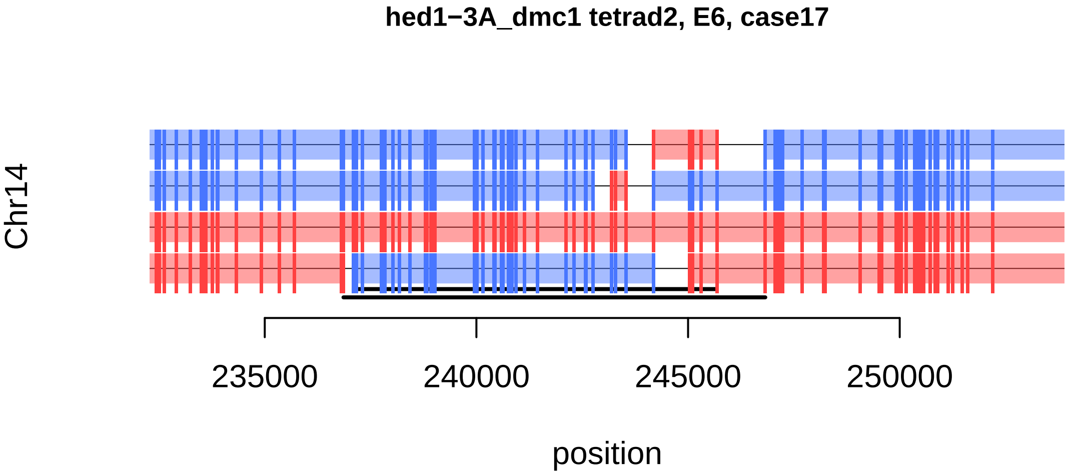
hed1−3A_dmc1 tetrad2, E6, case17
Chr14
235000
240000
245000
250000
position

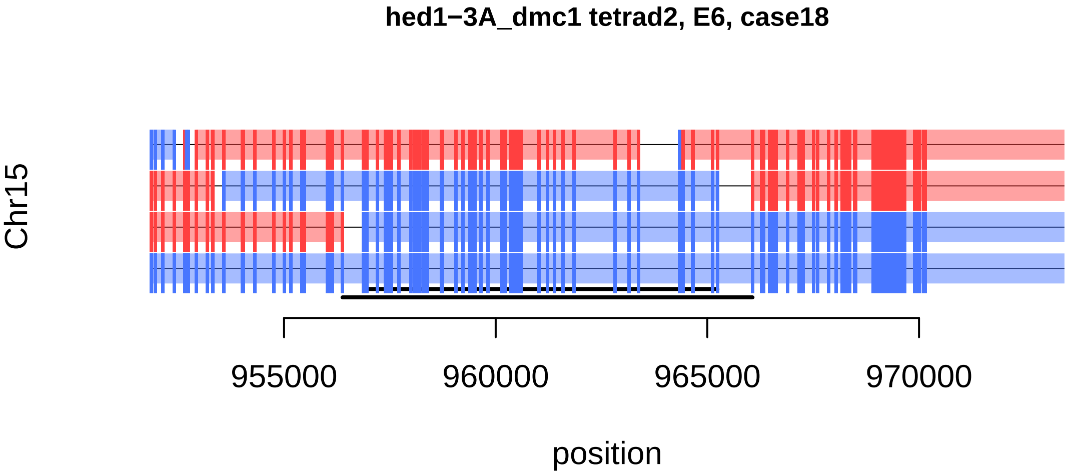
hed1−3A_dmc1 tetrad2, E6, case18
Chr15
955000
960000
965000
970000
position

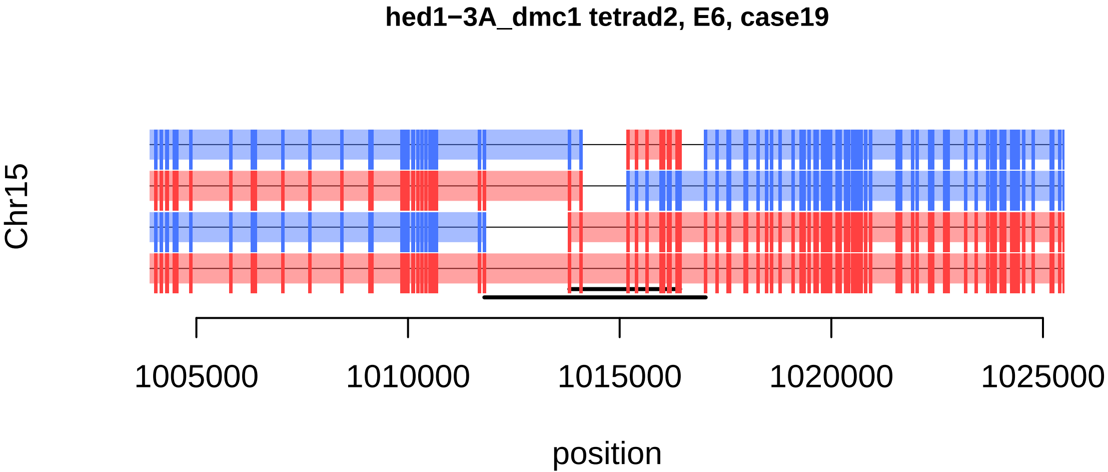
hed1−3A_dmc1 tetrad2, E6, case19
Chr15
1005000
1010000
1015000
1020000
1025000
position

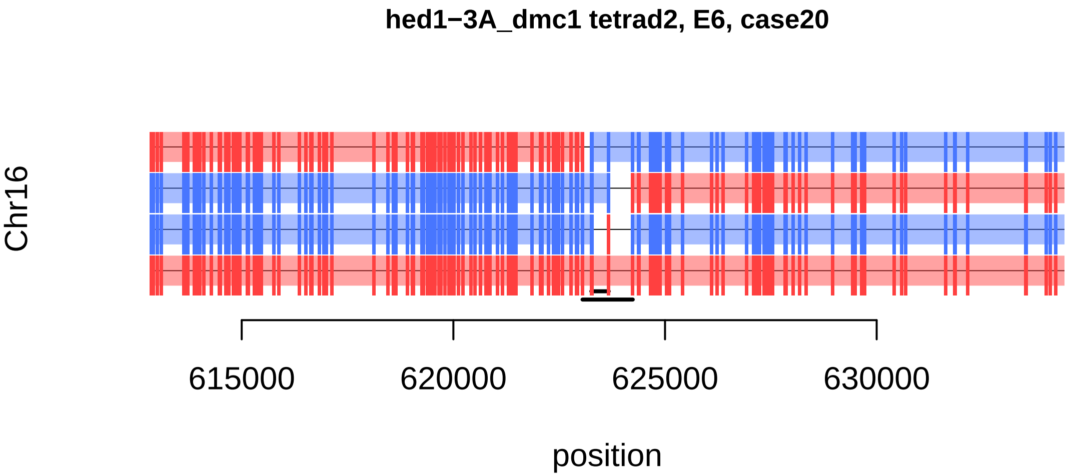

hed1−3A_dmc1 tetrad2, E6, case20
Chr16
615000
620000
625000
630000
position

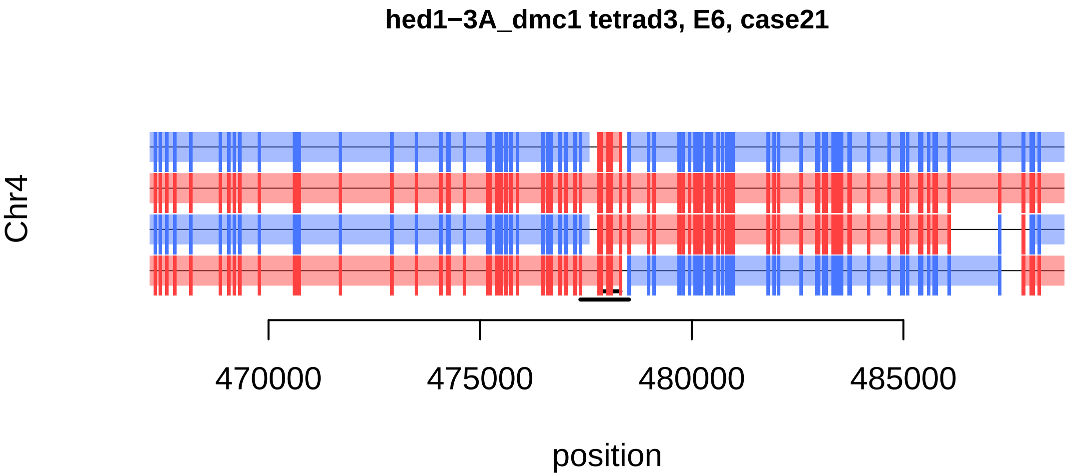
hed1−3A_dmc1 tetrad3, E6, case21
Chr4
470000
475000
480000
485000
position

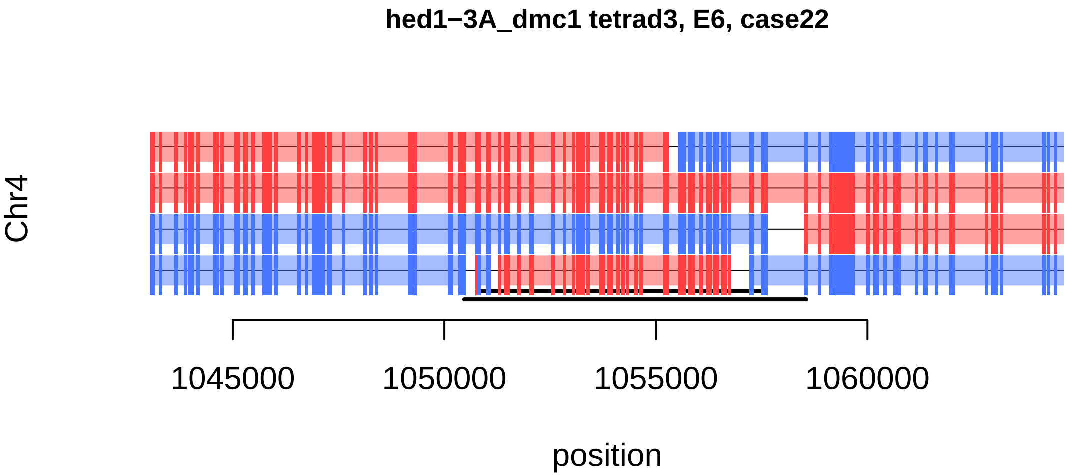
hed1−3A_dmc1 tetrad3, E6, case22
Chr4
1045000
1050000
1055000
1060000
position

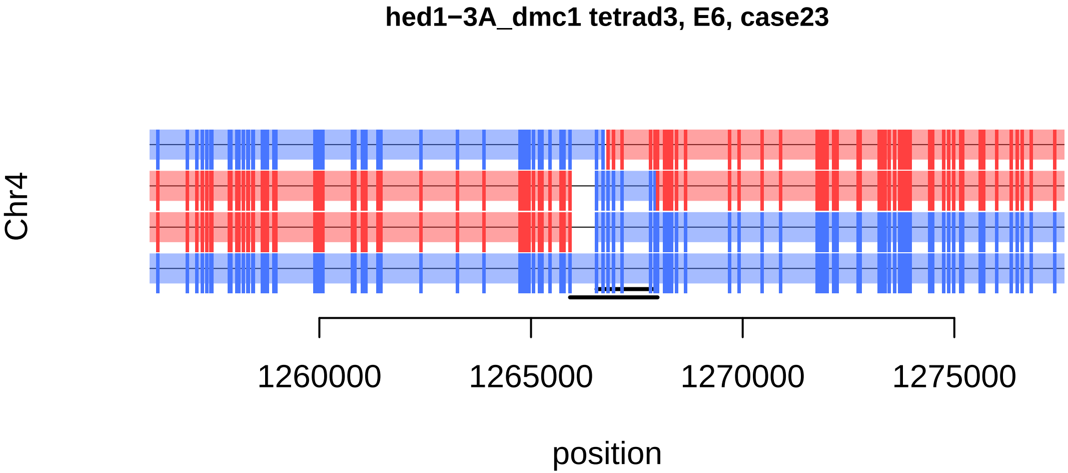
hed1−3A_dmc1 tetrad3, E6, case23
Chr4
1260000
1265000
1270000
1275000
position

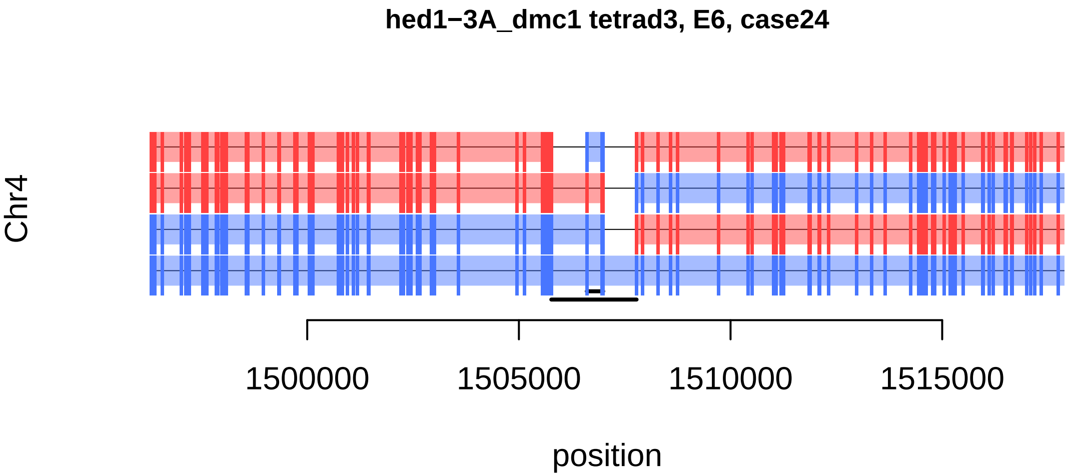

hed1−3A_dmc1 tetrad3, E6, case24
Chr4
1500000
1505000
1510000
1515000
position

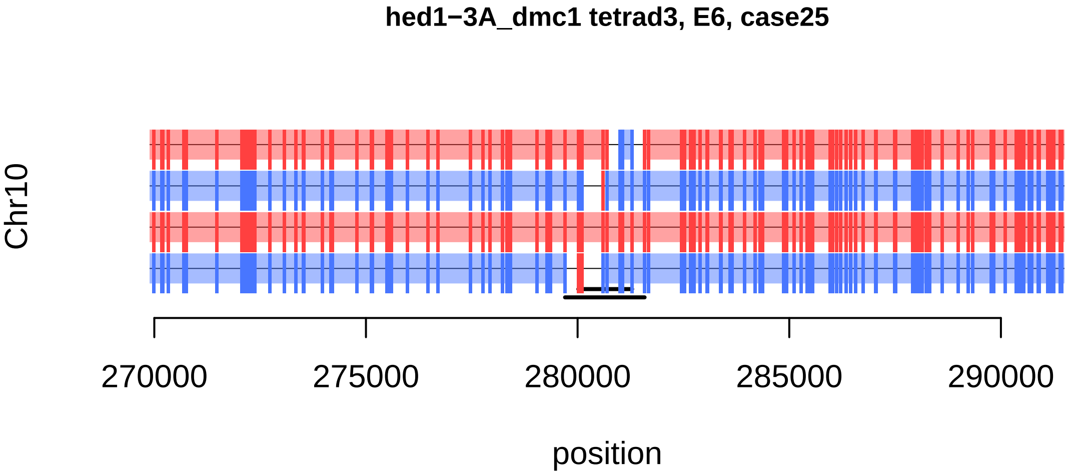
hed1−3A_dmc1 tetrad3, E6, case25
Chr10
270000
275000
280000
285000
290000
position

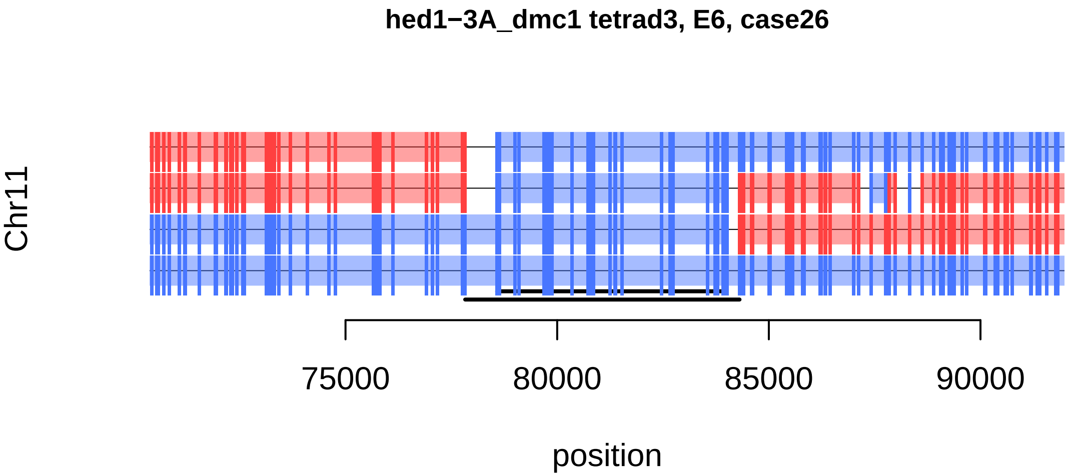
hed1−3A_dmc1 tetrad3, E6, case26
Chr11
75000
80000
85000
90000
position

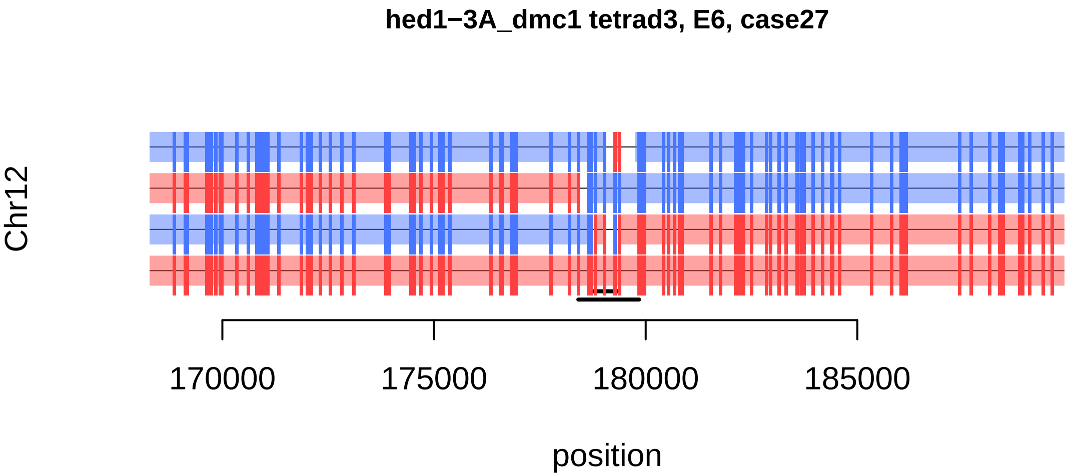
hed1−3A_dmc1 tetrad3, E6, case27
Chr12
170000
175000
180000
185000
position

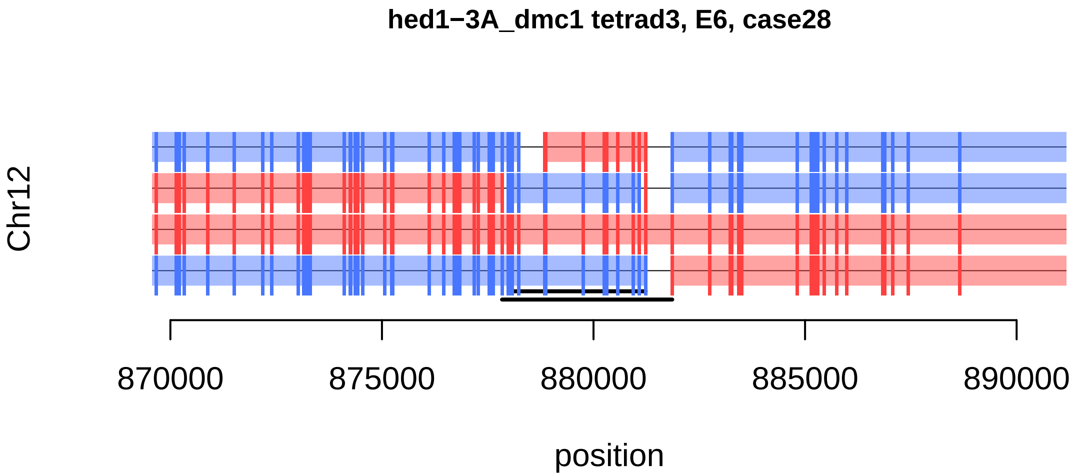
hed1−3A_dmc1 tetrad3, E6, case28
Chr12
870000
875000
880000
885000
890000
position

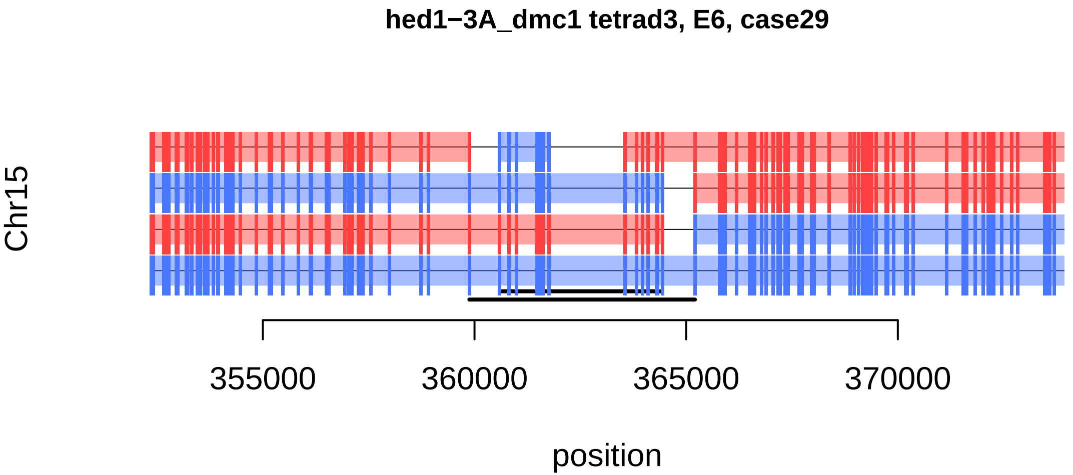
hed1−3A_dmc1 tetrad3, E6, case29
Chr15
355000
360000
365000
370000
position

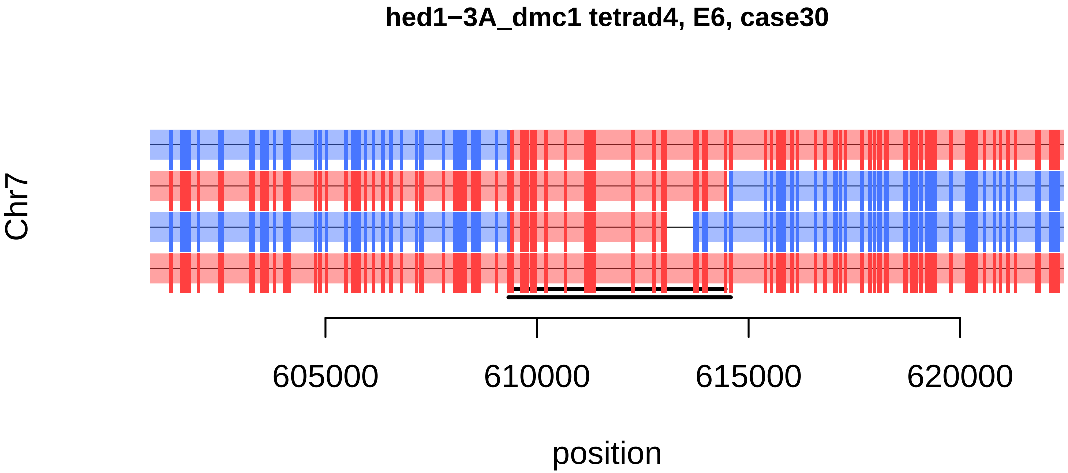
hed1−3A_dmc1 tetrad4, E6, case30
Chr7
605000
610000
615000
620000
position

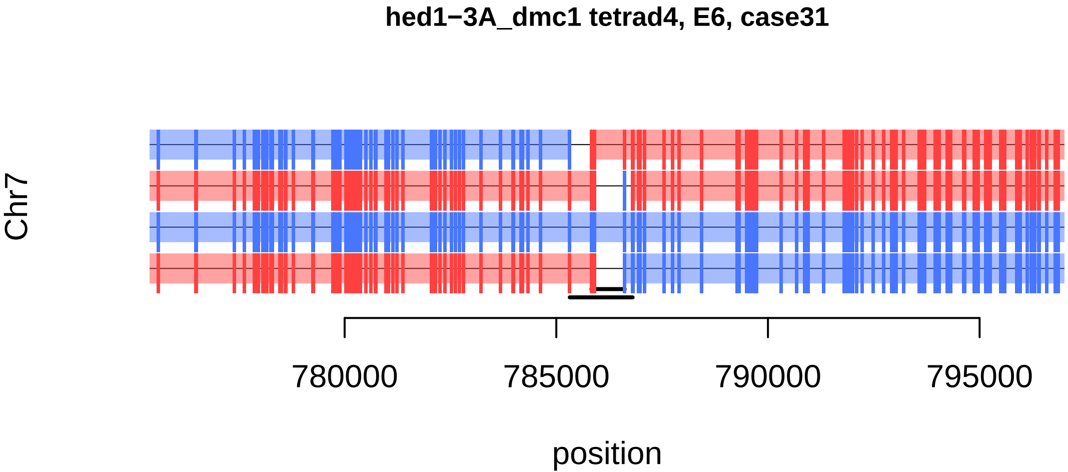
hed1−3A_dmc1 tetrad4, E6, case31
Chr7
780000
785000
790000
795000
position

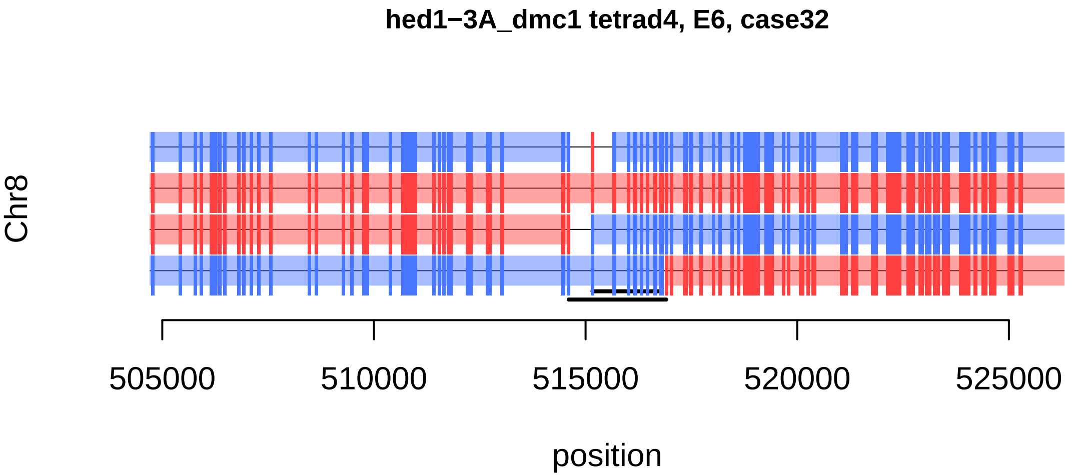
hed1−3A_dmc1 tetrad4, E6, case32
Chr8
505000
510000
515000
520000
525000
position

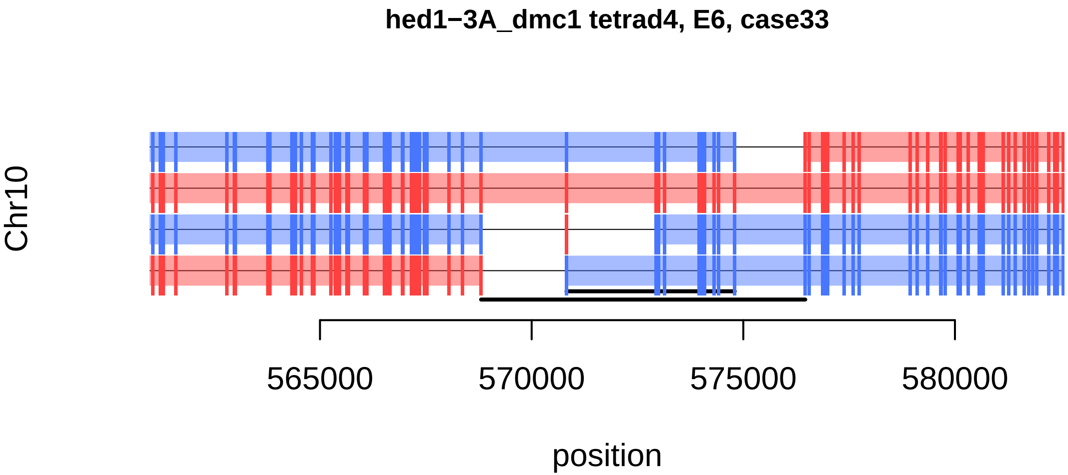
hed1−3A_dmc1 tetrad4, E6, case33
Chr10
565000
570000
575000
580000
position

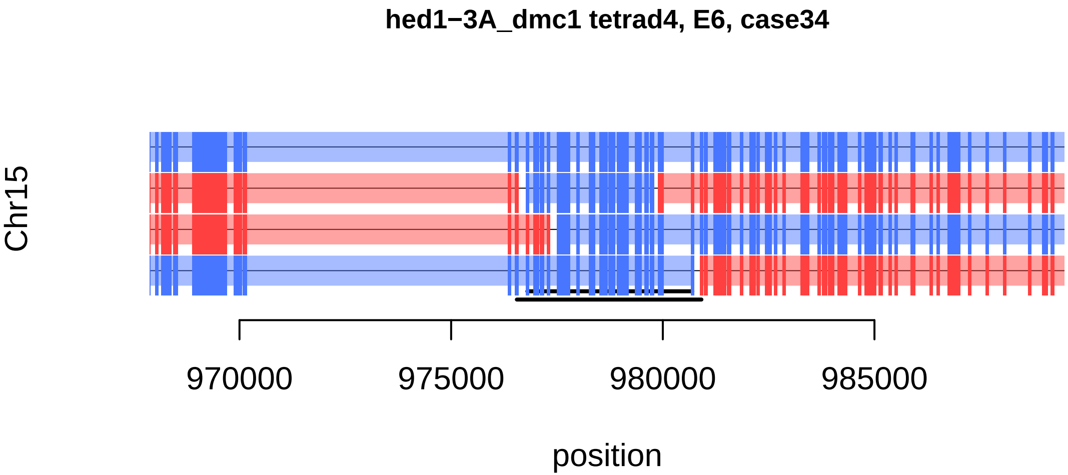
hed1−3A_dmc1 tetrad4, E6, case34
Chr15
970000
975000
980000
985000
position

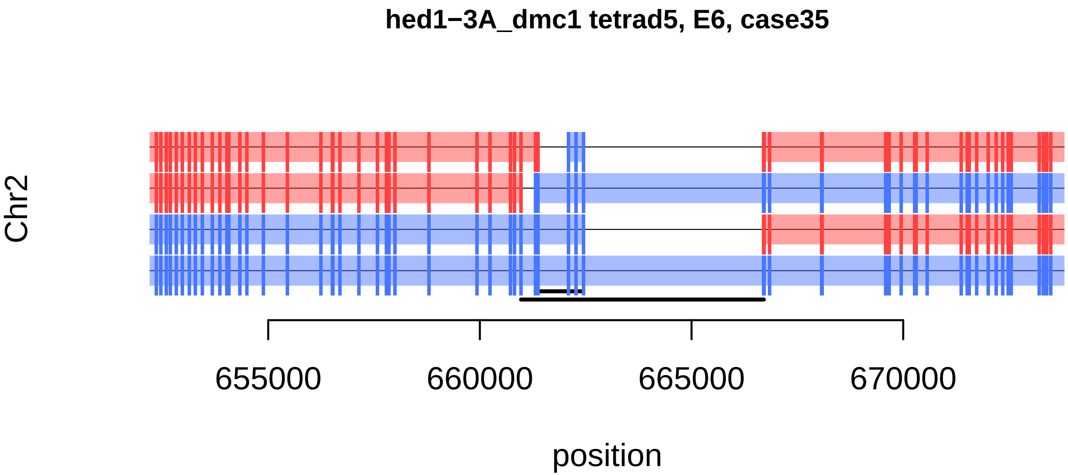
hed1−3A_dmc1 tetrad5, E6, case35
Chr2
655000
660000
665000
670000
position

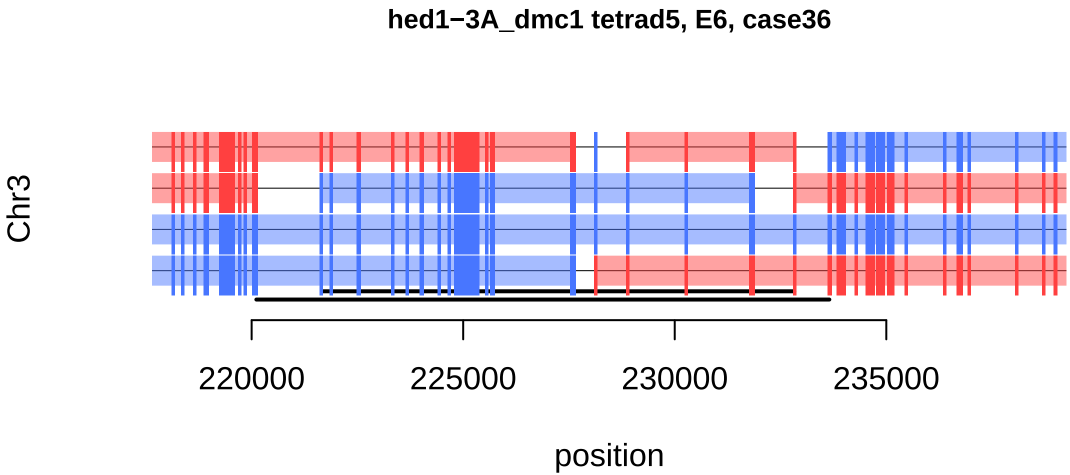

hed1−3A_dmc1 tetrad5, E6, case36
Chr3
220000
225000
230000
235000
position

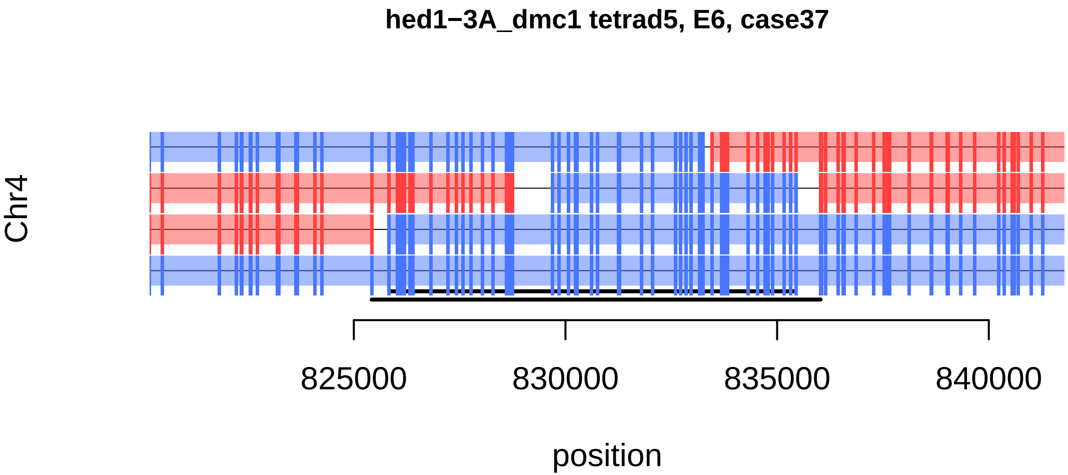
hed1−3A_dmc1 tetrad5, E6, case37
Chr4
825000
830000
835000
840000
position

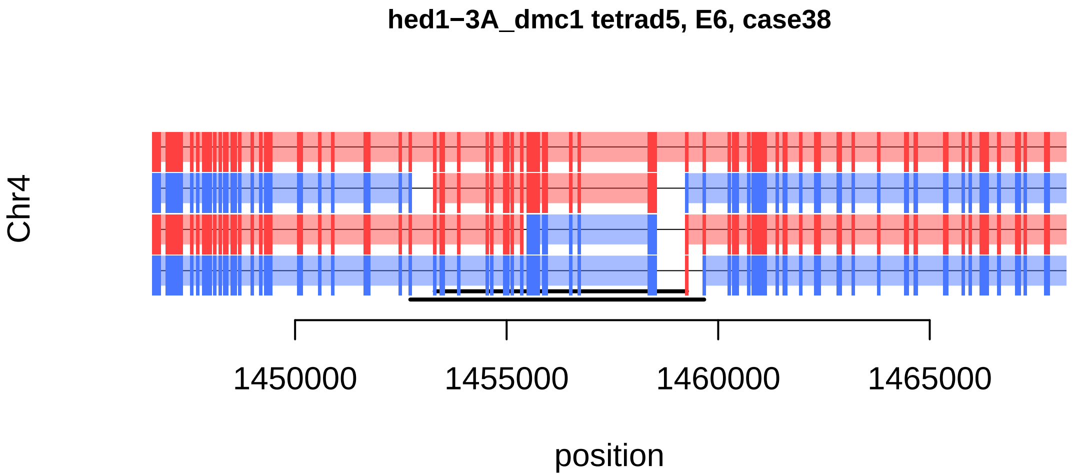
hed1−3A_dmc1 tetrad5, E6, case38
Chr4
1450000
1455000
1460000
1465000
position

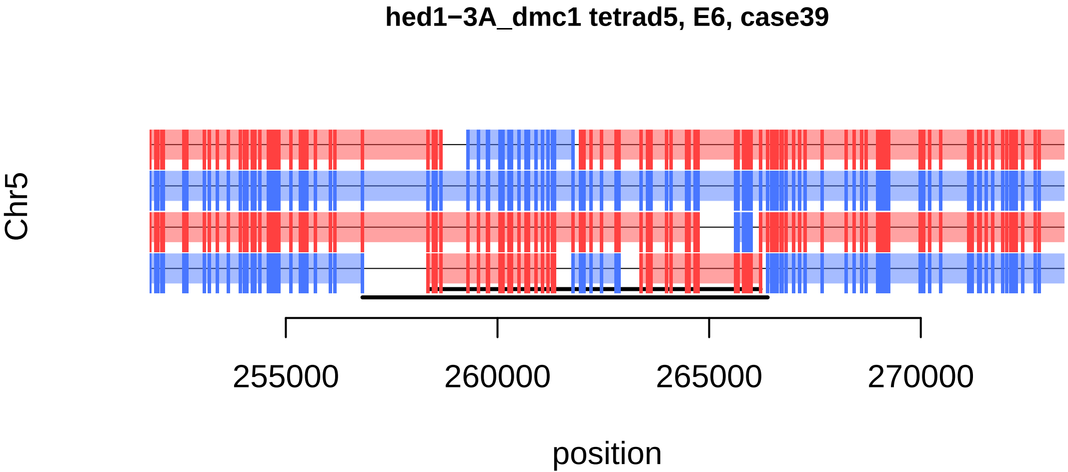
hed1−3A_dmc1 tetrad5, E6, case39
Chr5
255000
260000
265000
270000
position

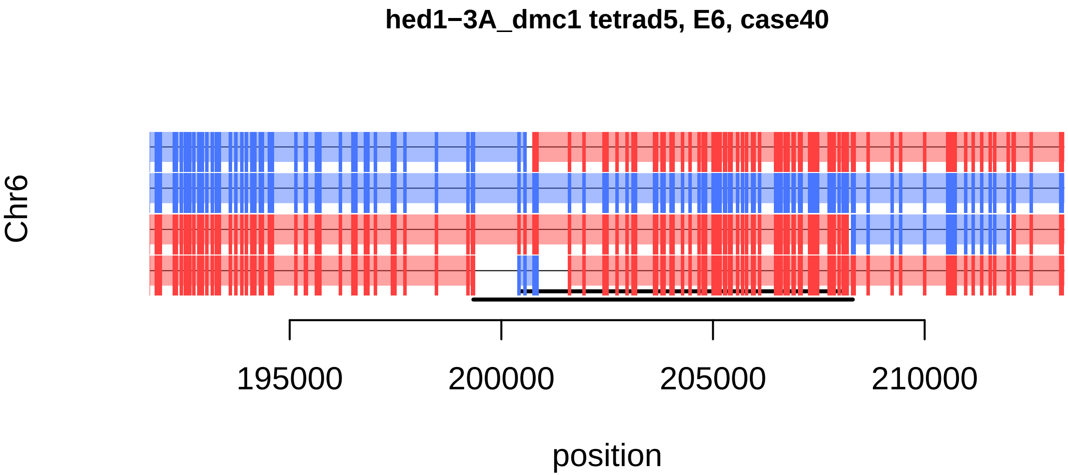
hed1−3A_dmc1 tetrad5, E6, case40
Chr6
195000
200000
205000
210000
position

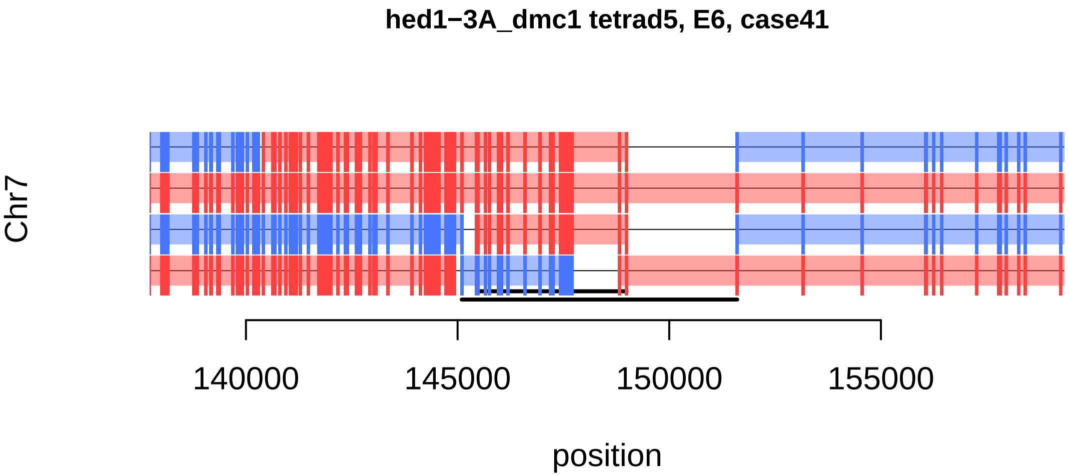
hed1−3A_dmc1 tetrad5, E6, case41
Chr7
140000
145000
150000
155000
position

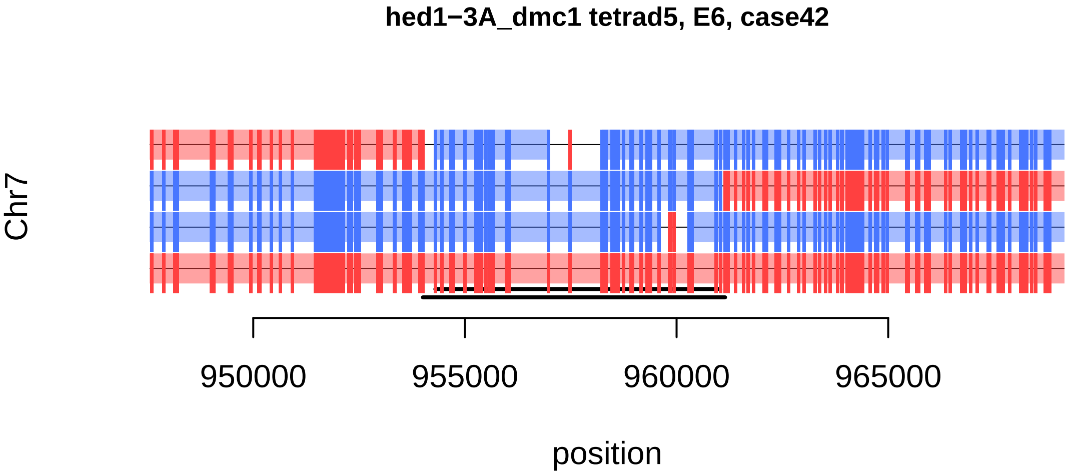
hed1−3A_dmc1 tetrad5, E6, case42
Chr7
950000
955000
960000
965000
position

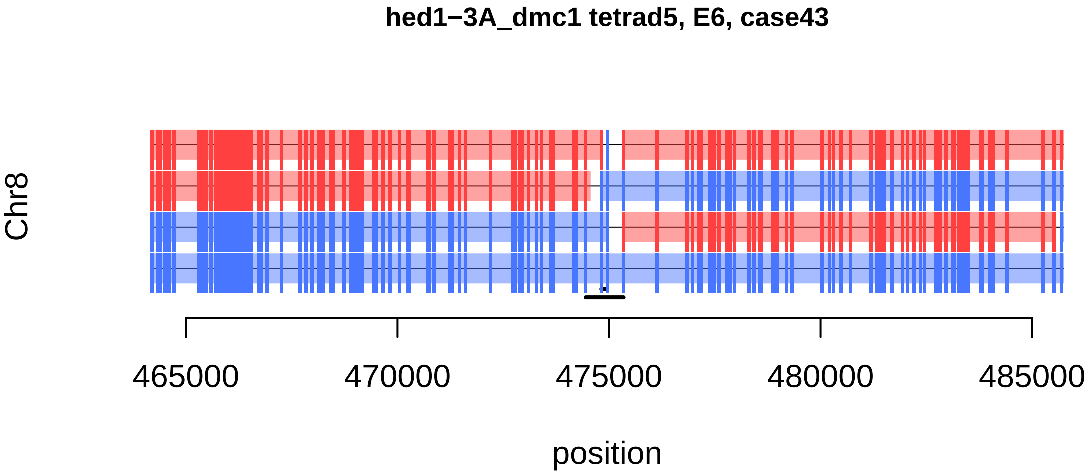

hed1−3A_dmc1 tetrad5, E6, case43
Chr8
465000
470000
475000
480000
485000
position

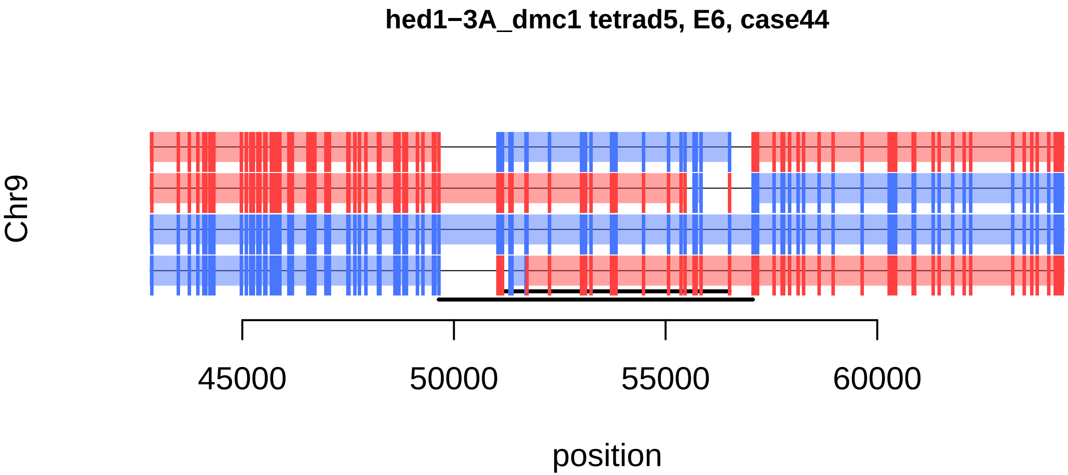
hed1−3A_dmc1 tetrad5, E6, case44
Chr9
45000
50000
55000
60000
position

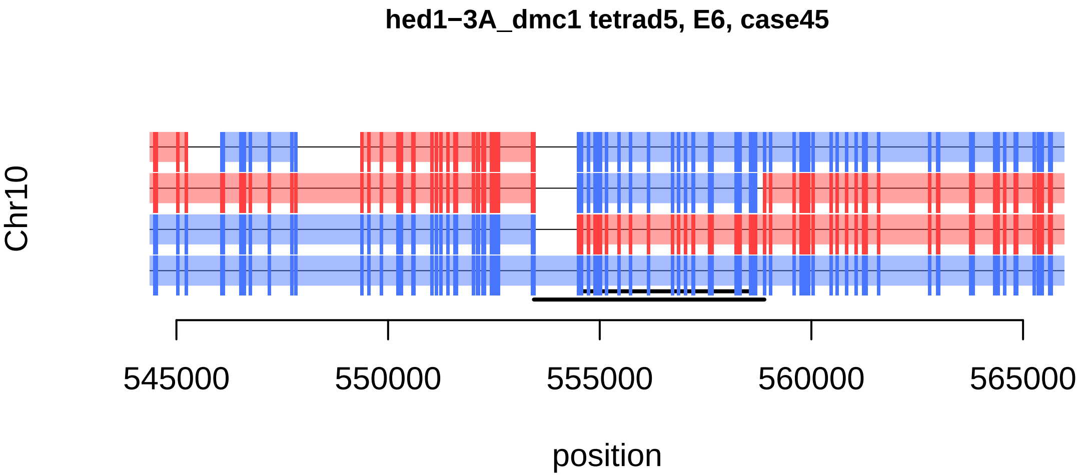

hed1−3A_dmc1 tetrad5, E6, case45
Chr10
545000
550000
555000
560000
565000
position

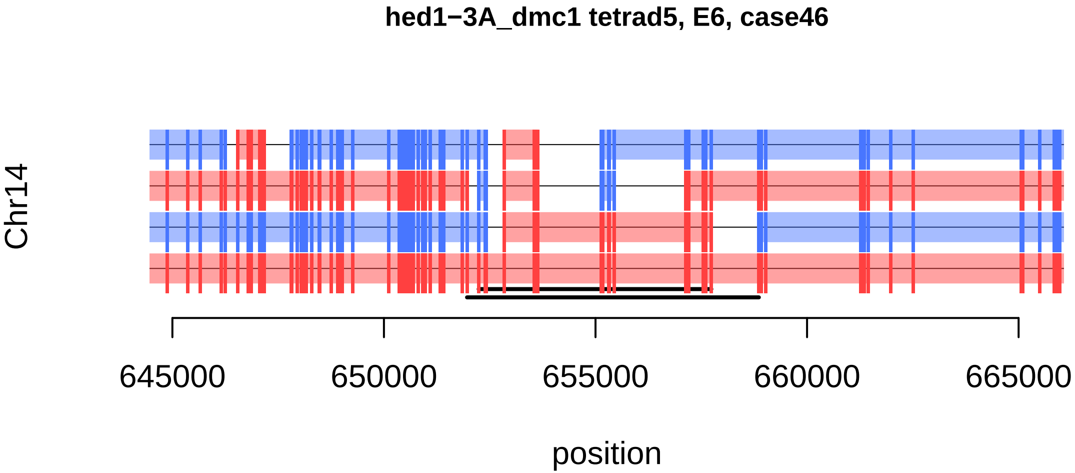
hed1−3A_dmc1 tetrad5, E6, case46
Chr14
645000
650000
655000
660000
665000
position

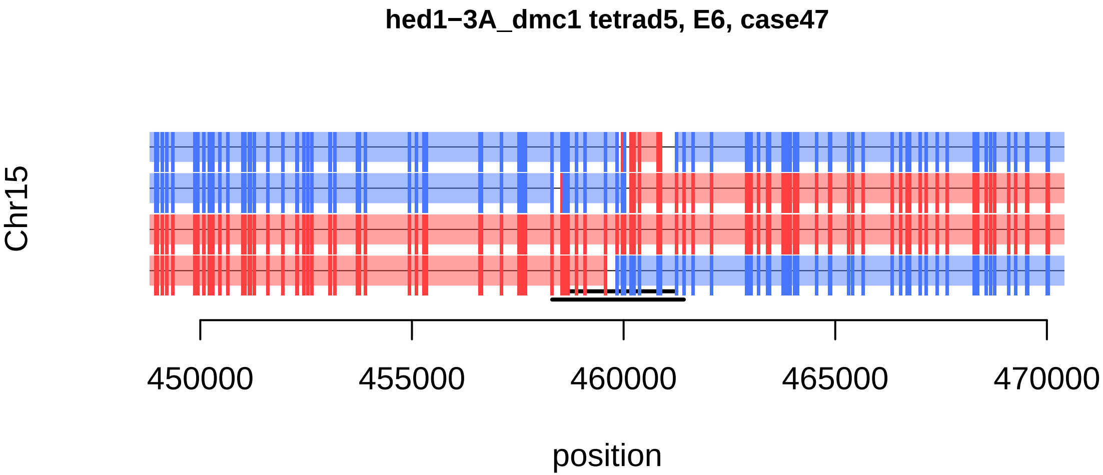
hed1−3A_dmc1 tetrad5, E6, case47
Chr15
450000
455000
460000
465000
470000
position

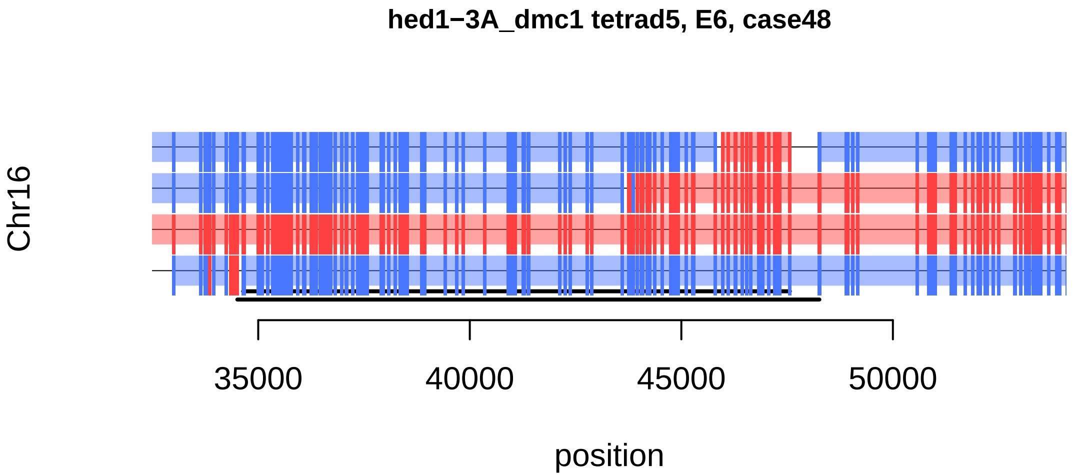
hed1−3A_dmc1 tetrad5, E6, case48
Chr16
35000
40000
45000
50000
position

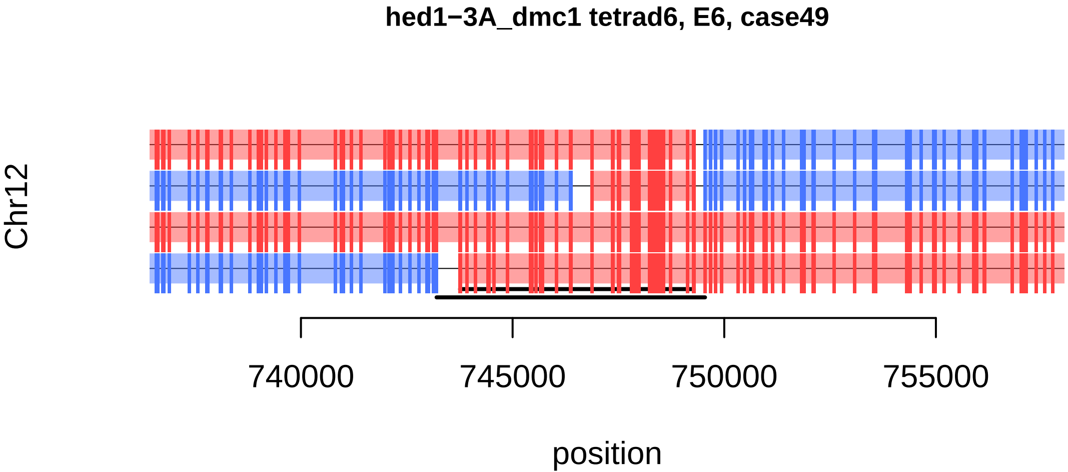
hed1−3A_dmc1 tetrad6, E6, case49
Chr12
740000
745000
750000
755000
position

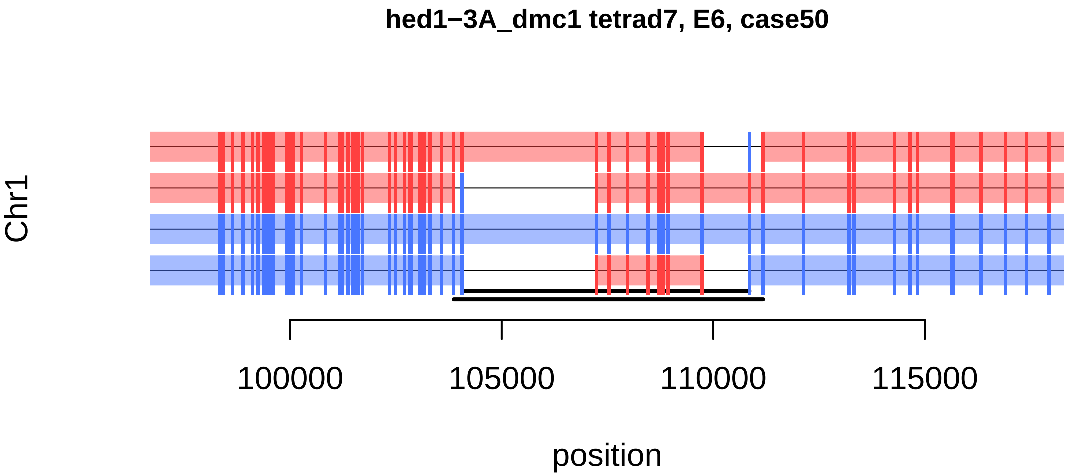
hed1−3A_dmc1 tetrad7, E6, case50
Chr1
100000
105000
110000
115000
position

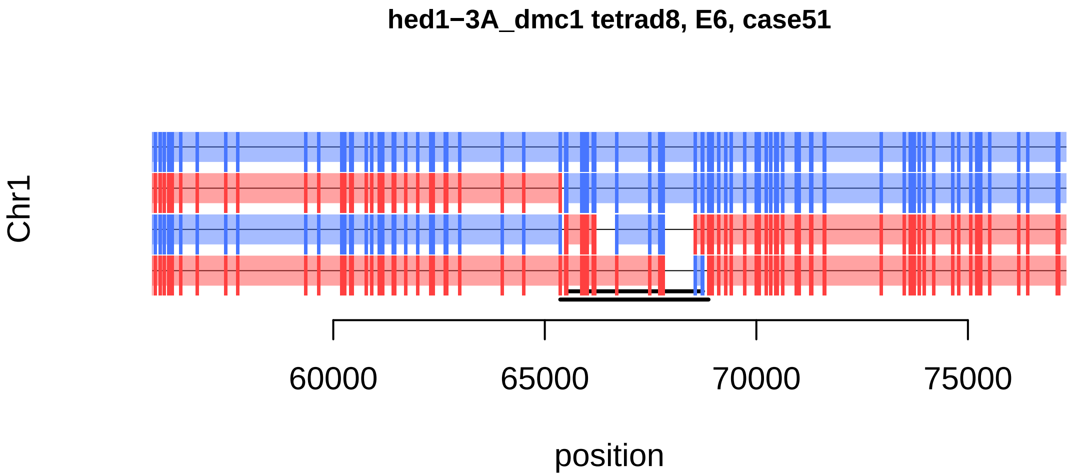
hed1−3A_dmc1 tetrad8, E6, case51
Chr1
60000
65000
70000
75000
position

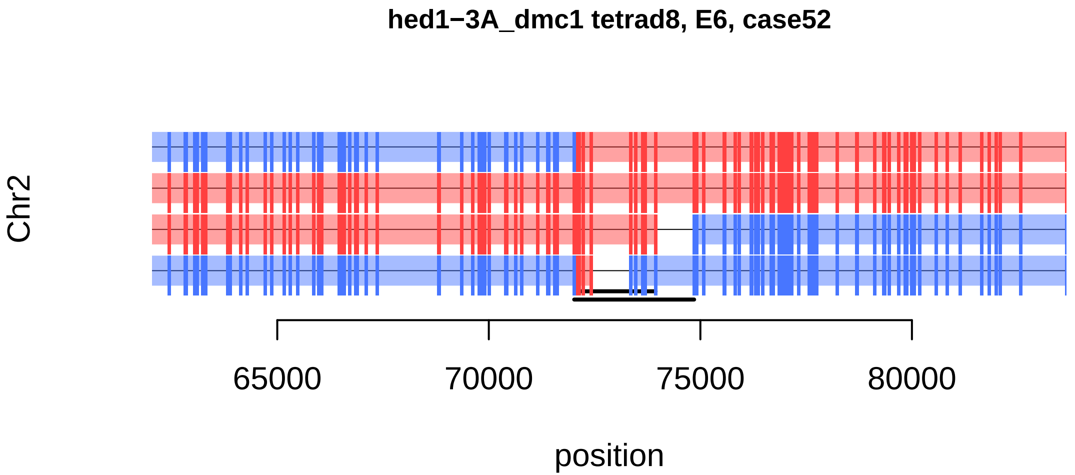
hed1−3A_dmc1 tetrad8, E6, case52
Chr2
65000
70000
75000
80000
position

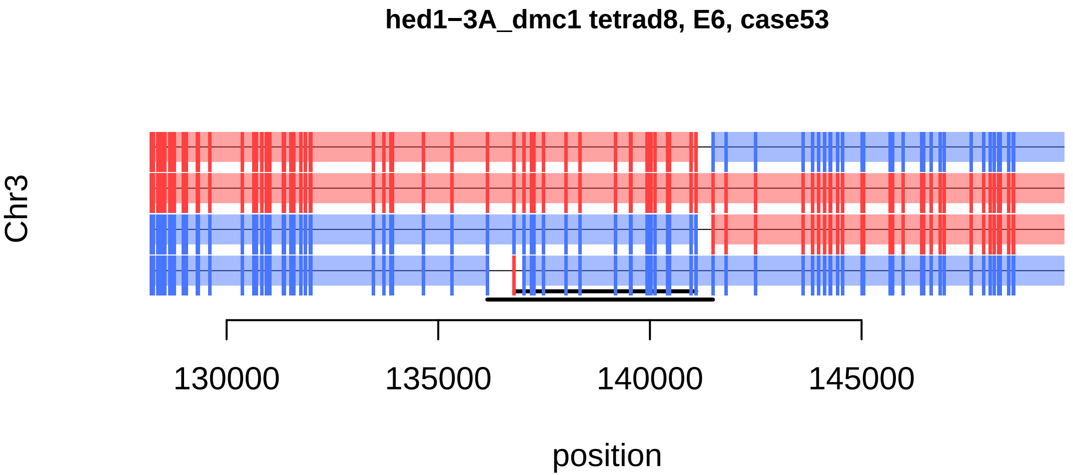
hed1−3A_dmc1 tetrad8, E6, case53
Chr3
130000
135000
140000
145000
position

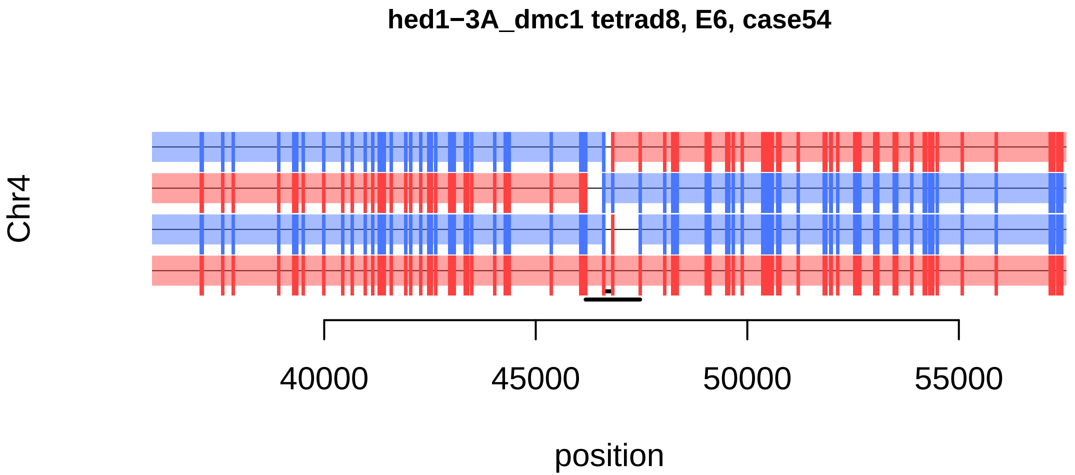

hed1−3A_dmc1 tetrad8, E6, case54
Chr4
40000
45000
50000
55000
position

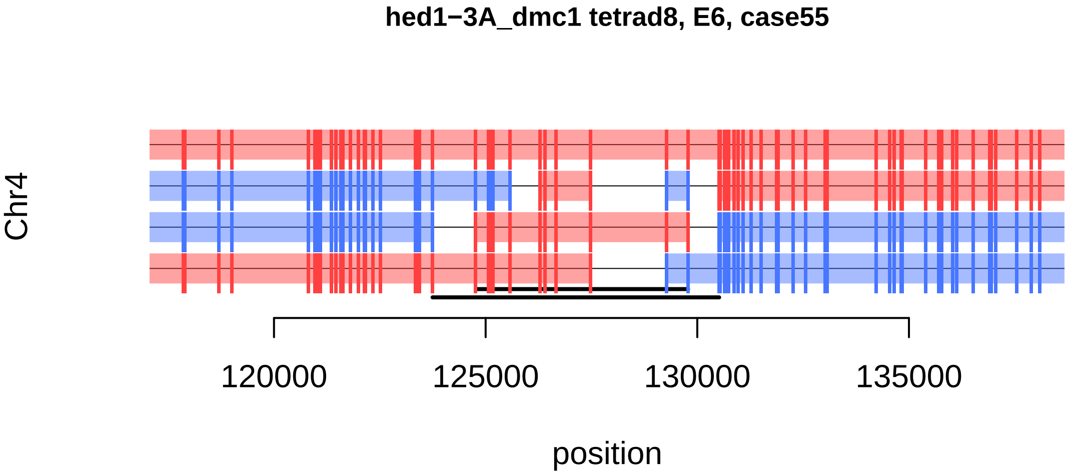
hed1−3A_dmc1 tetrad8, E6, case55
Chr4
120000
125000
130000
135000
position

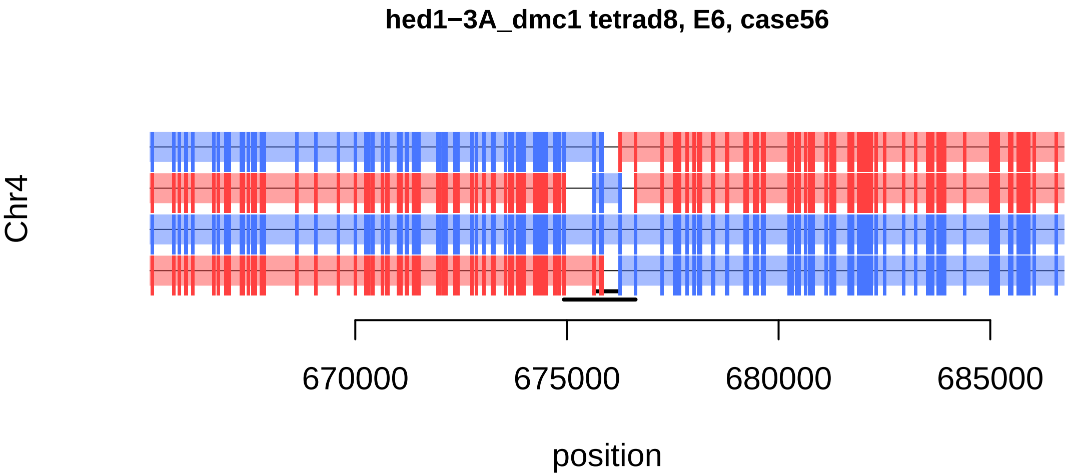
hed1−3A_dmc1 tetrad8, E6, case56
Chr4
670000
675000
680000
685000
position

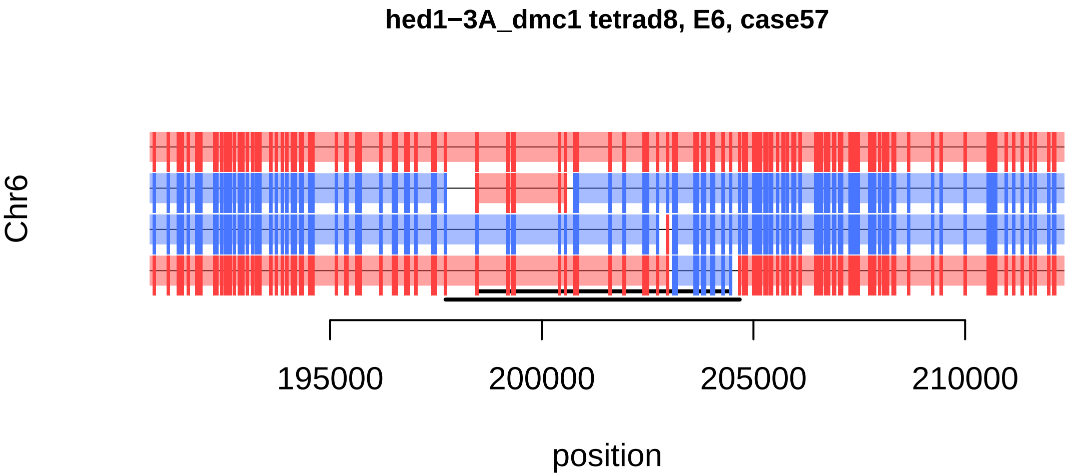
hed1−3A_dmc1 tetrad8, E6, case57
Chr6
195000
200000
205000
210000
position

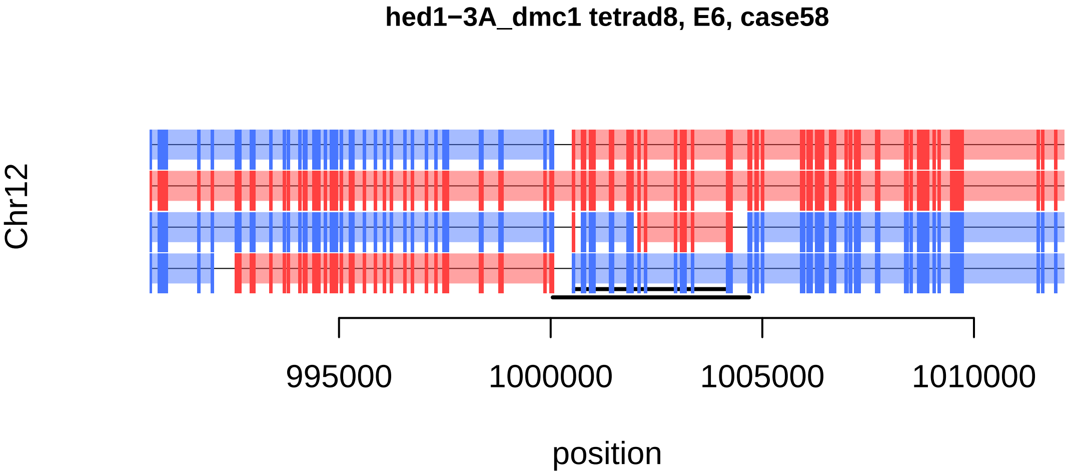
hed1−3A_dmc1 tetrad8, E6, case58
Chr12
995000
1000000
1005000
1010000
position

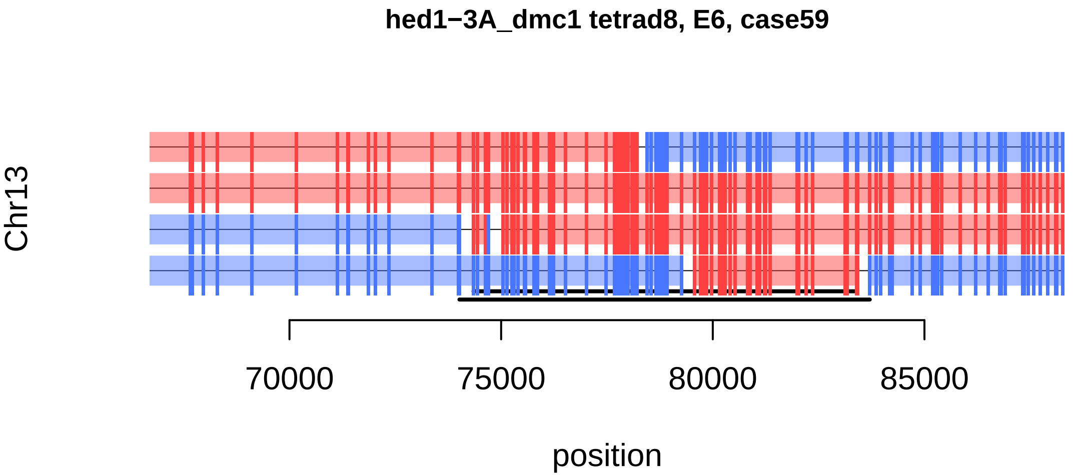

hed1−3A_dmc1 tetrad8, E6, case59
Chr13
70000
75000
80000
85000
position

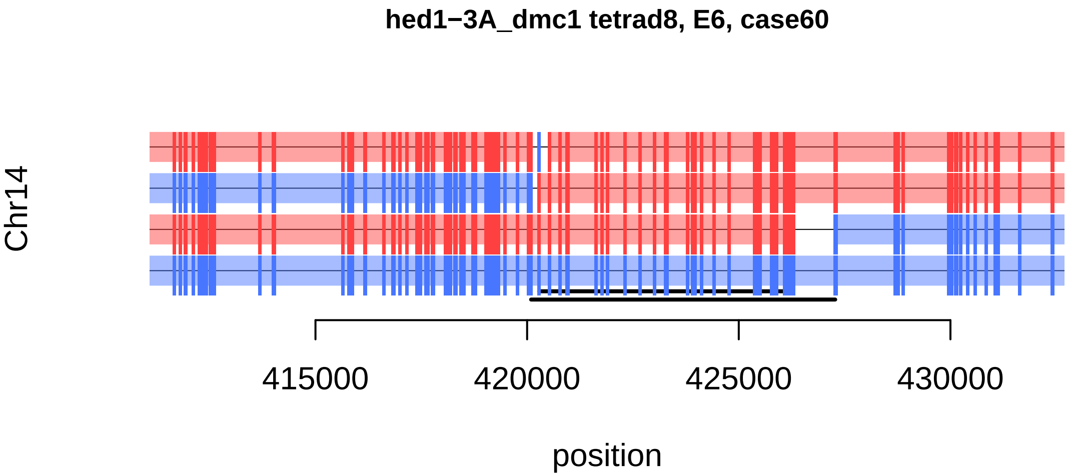
hed1−3A_dmc1 tetrad8, E6, case60
Chr14
415000
420000
425000
430000
position

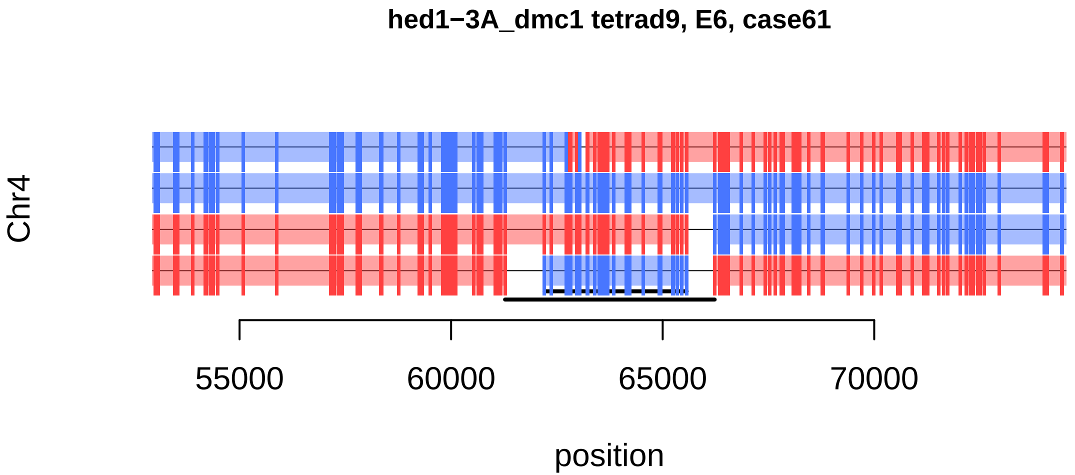
hed1−3A_dmc1 tetrad9, E6, case61
Chr4
55000
60000
65000
70000
position

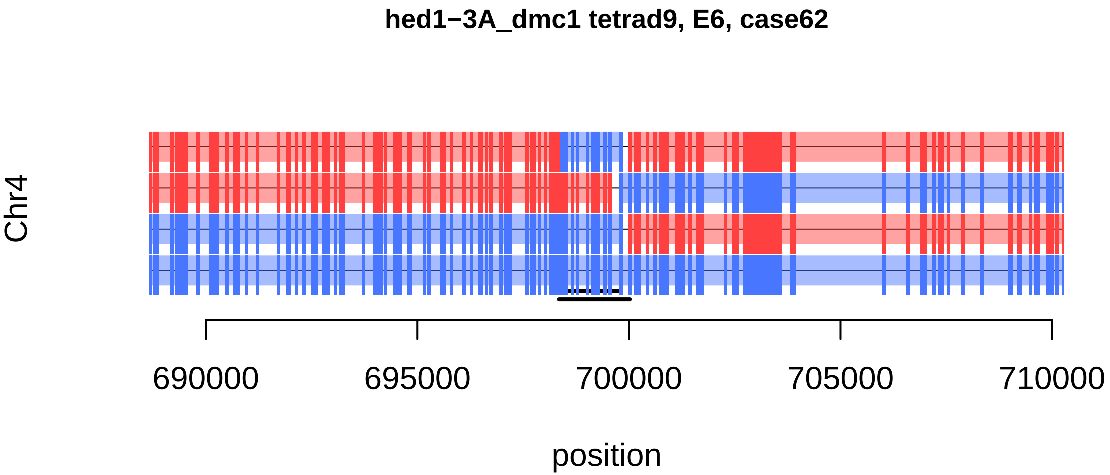
hed1−3A_dmc1 tetrad9, E6, case62
Chr4
690000
695000
700000
705000
710000
position

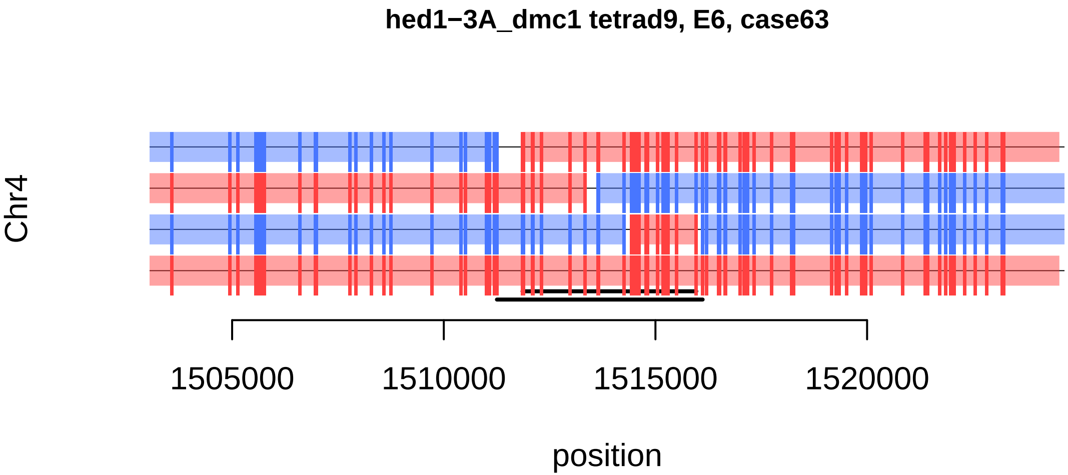
hed1−3A_dmc1 tetrad9, E6, case63
Chr4
1505000
1510000
1515000
1520000
position

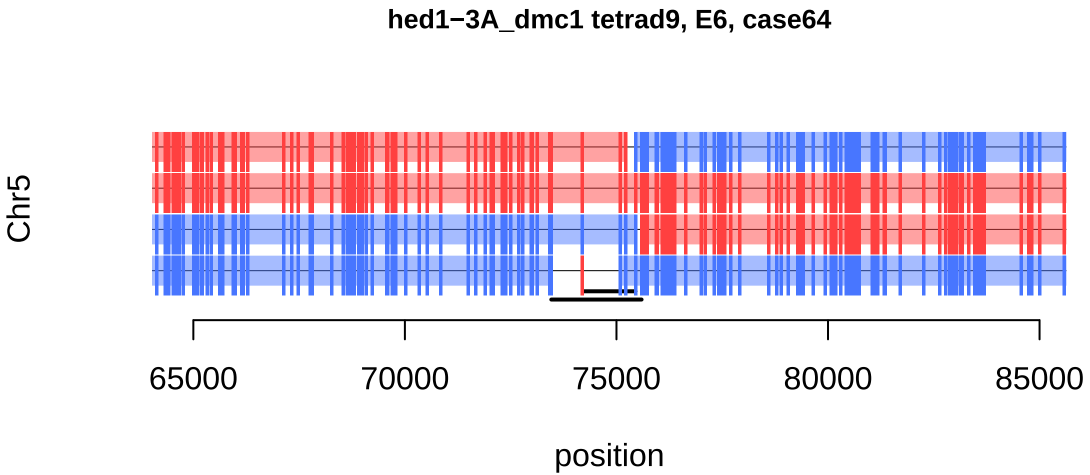
hed1−3A_dmc1 tetrad9, E6, case64
Chr5
65000
70000
75000
80000
85000
position

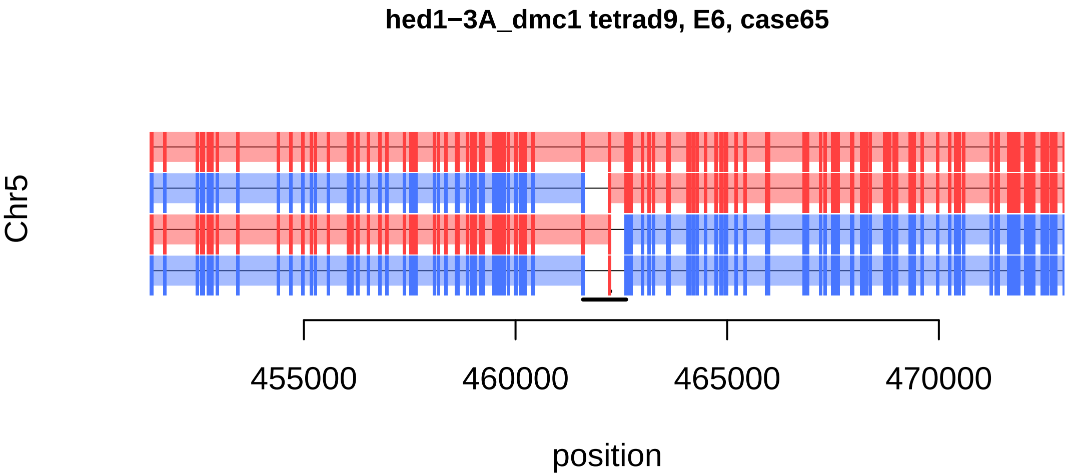
hed1−3A_dmc1 tetrad9, E6, case65
Chr5
455000
460000
465000
470000
position

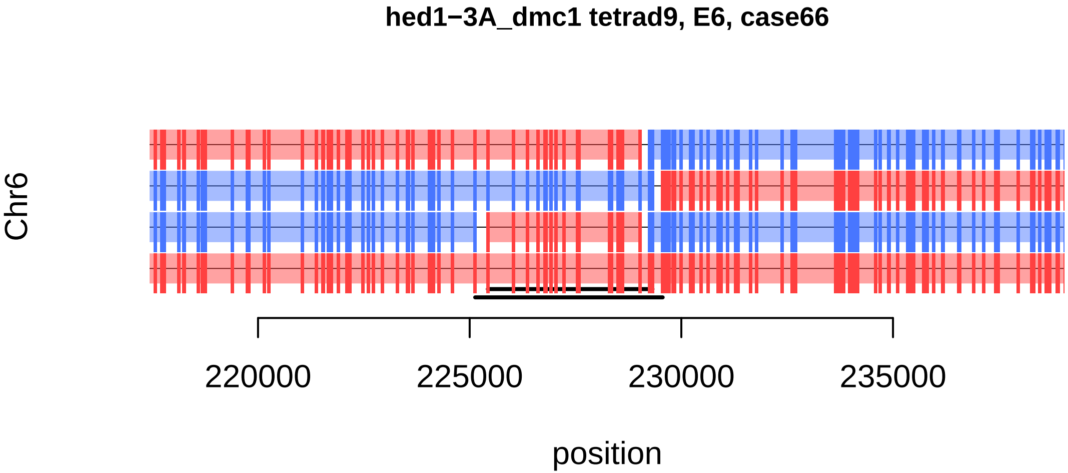
hed1−3A_dmc1 tetrad9, E6, case66
Chr6
220000
225000
230000
235000
position

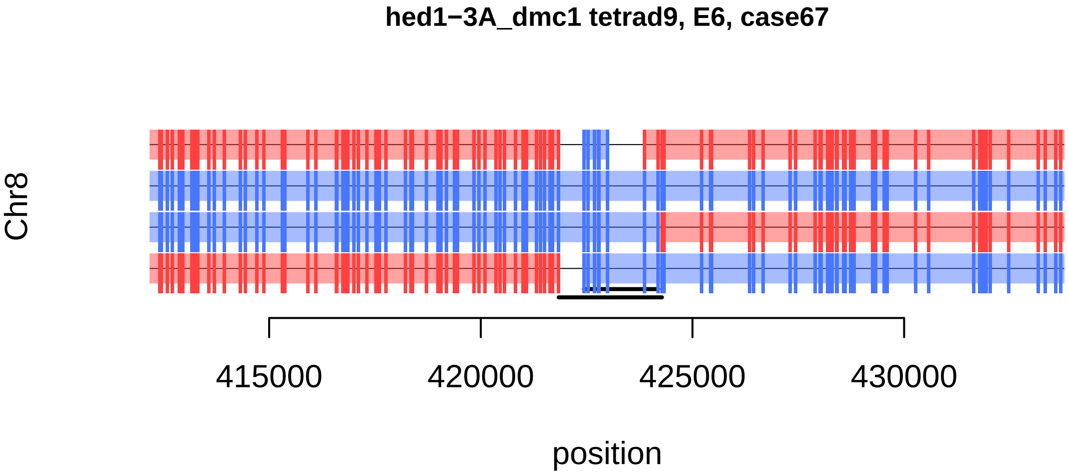

hed1−3A_dmc1 tetrad9, E6, case67
Chr8
415000
420000
425000
430000
position

hed1−3A_dmc1 tetrad9, E6, case68

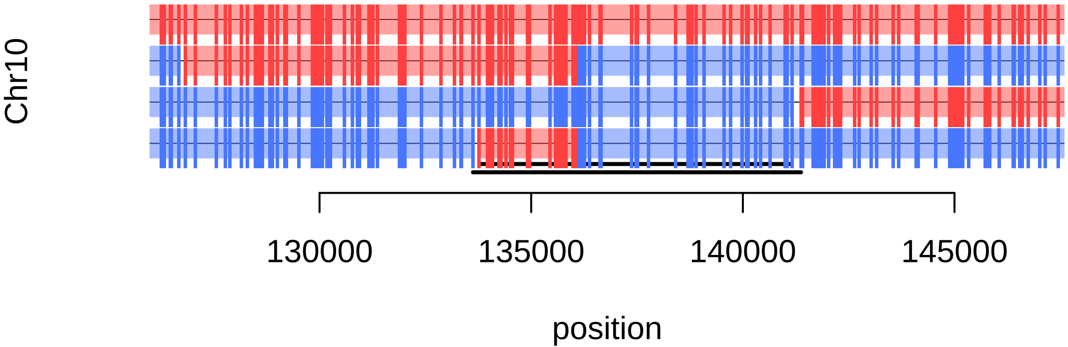

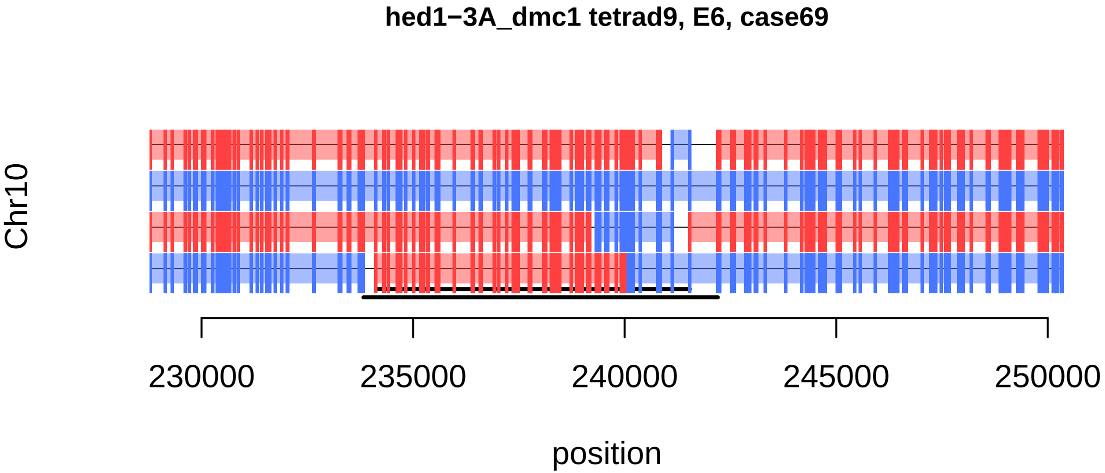
hed1−3A_dmc1 tetrad9, E6, case69
Chr10
230000
235000
240000
245000
250000
position

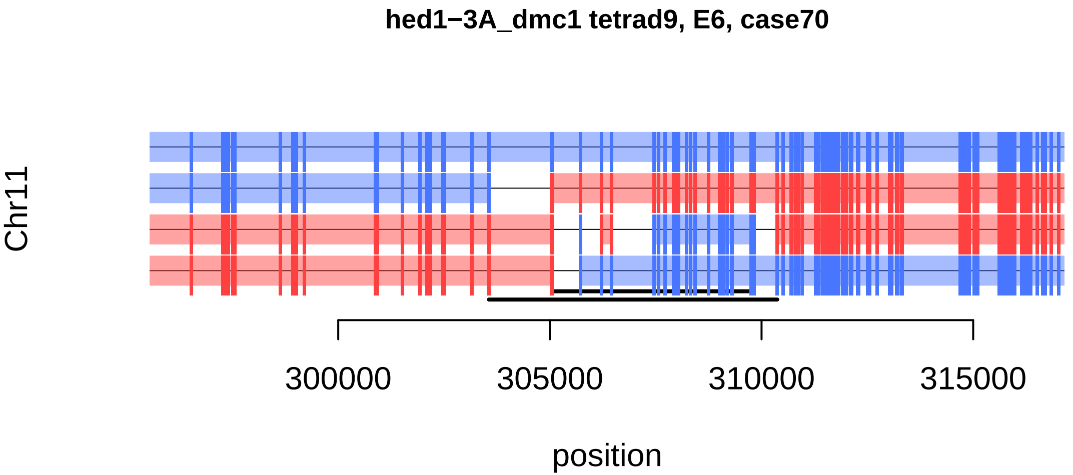
hed1−3A_dmc1 tetrad9, E6, case70
Chr11
300000
305000
310000
315000
position

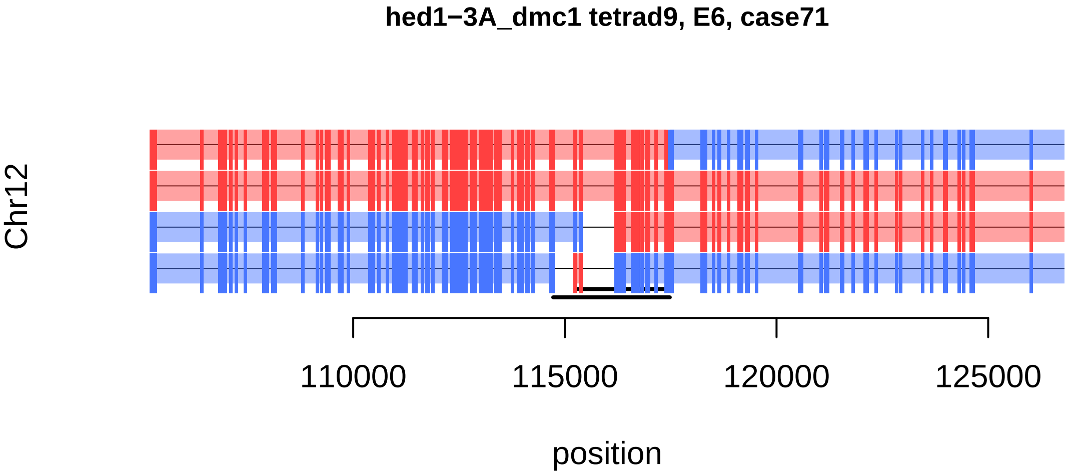

hed1−3A_dmc1 tetrad9, E6, case71
Chr12
110000
115000
120000
125000
position

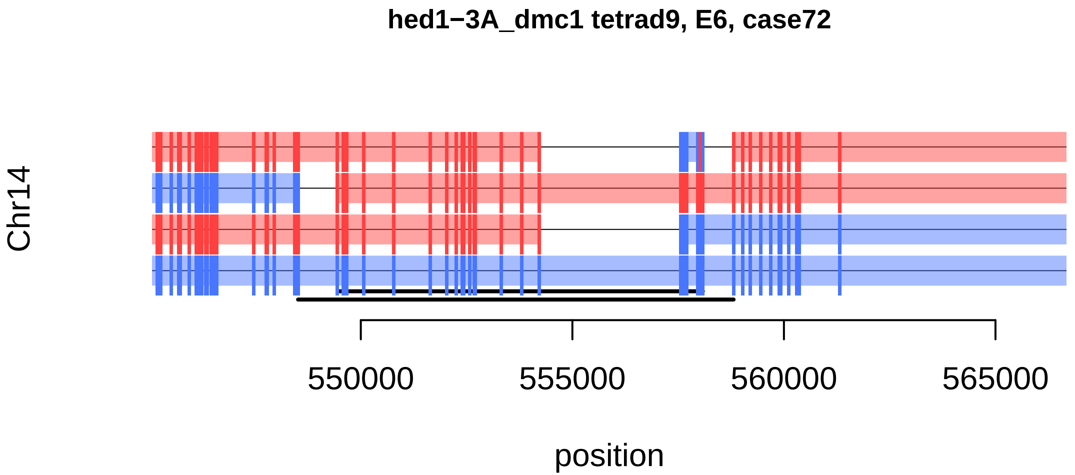
hed1−3A_dmc1 tetrad9, E6, case72
Chr14
550000
555000
560000
565000
position

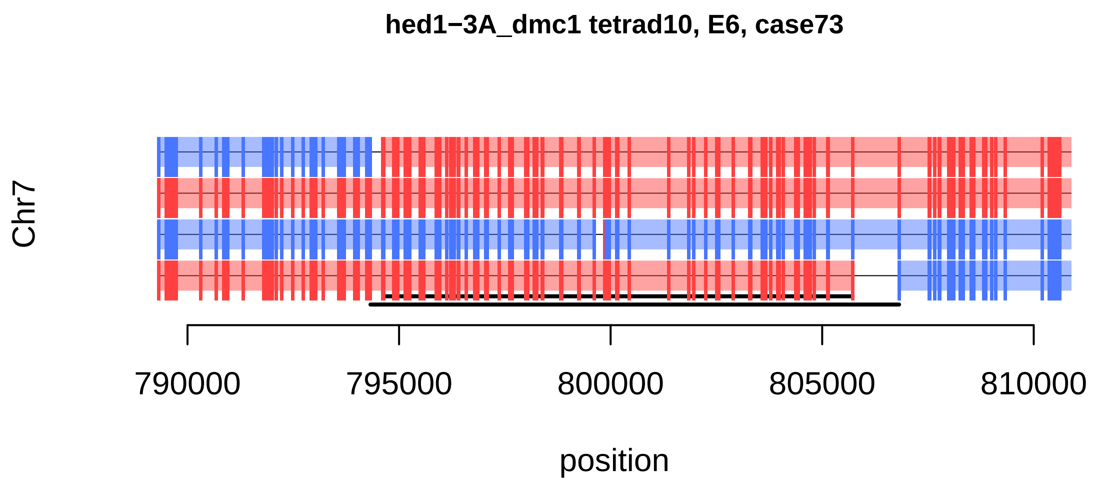
hed1−3A_dmc1 tetrad10, E6, case73
Chr7
790000
795000
800000
805000
810000
position

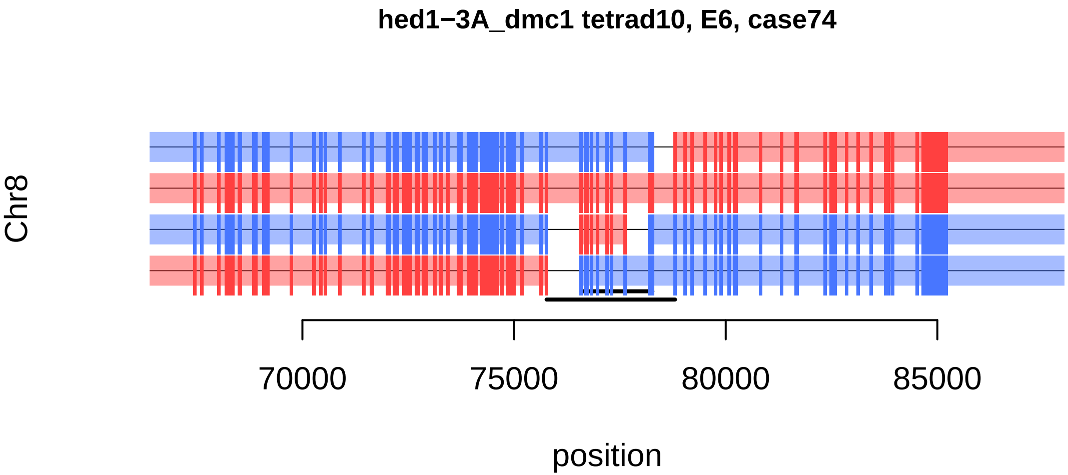
hed1−3A_dmc1 tetrad10, E6, case74
Chr8
70000
75000
80000
85000
position

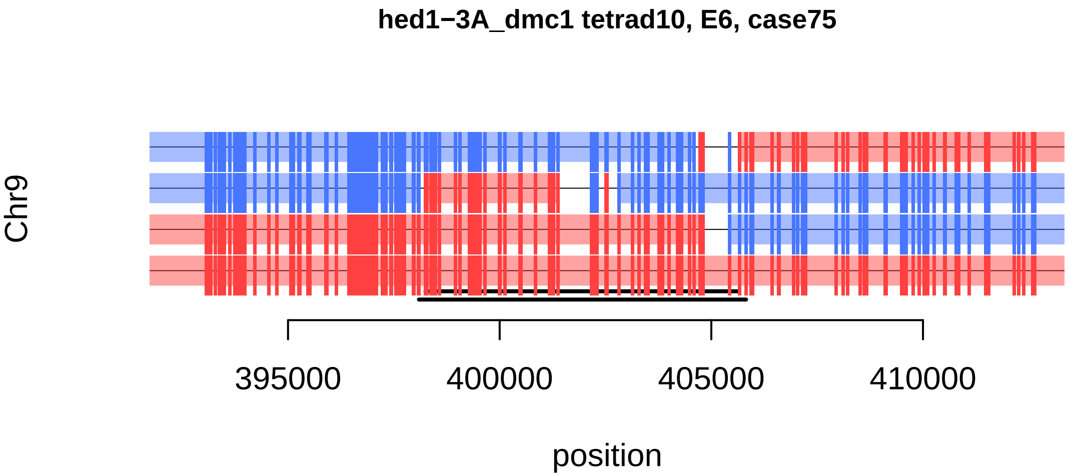
hed1−3A_dmc1 tetrad10, E6, case75
Chr9
395000
400000
405000
410000
position

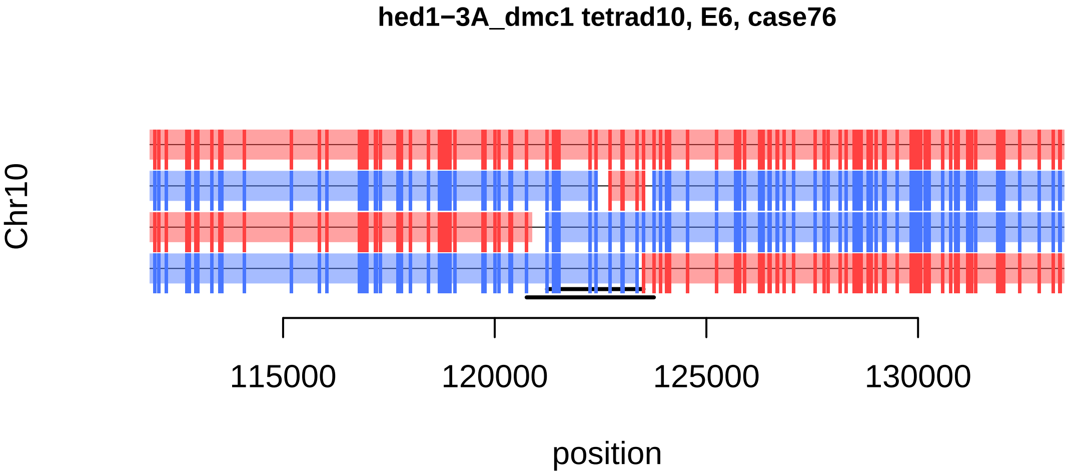
hed1−3A_dmc1 tetrad10, E6, case76
Chr10
115000
120000
125000
130000
position

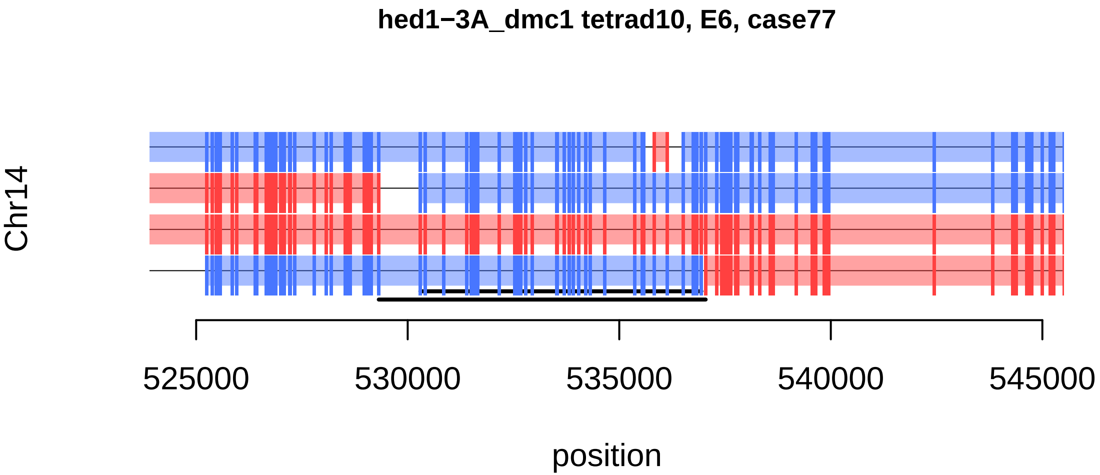
hed1−3A_dmc1 tetrad10, E6, case77
Chr14
525000
530000
535000
540000
545000
position

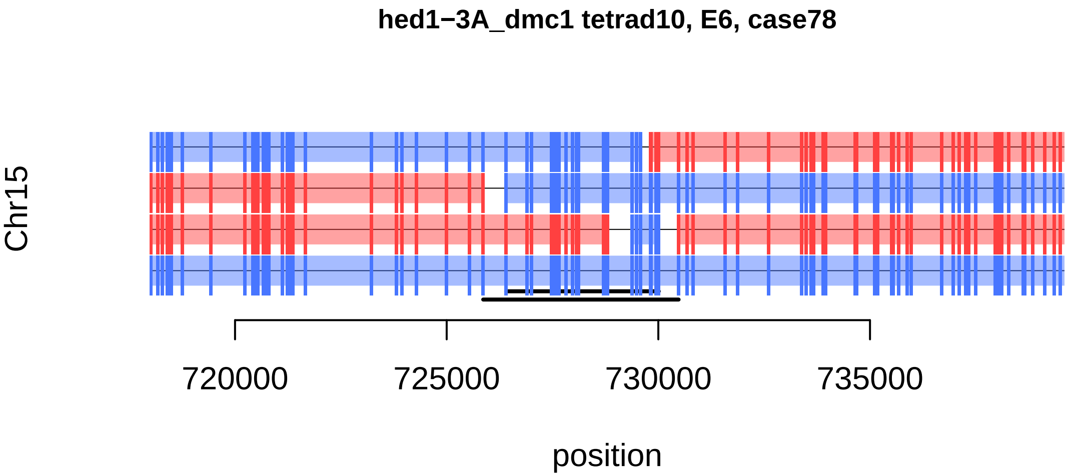

hed1−3A_dmc1 tetrad10, E6, case78
Chr15
720000
725000
730000
735000
position

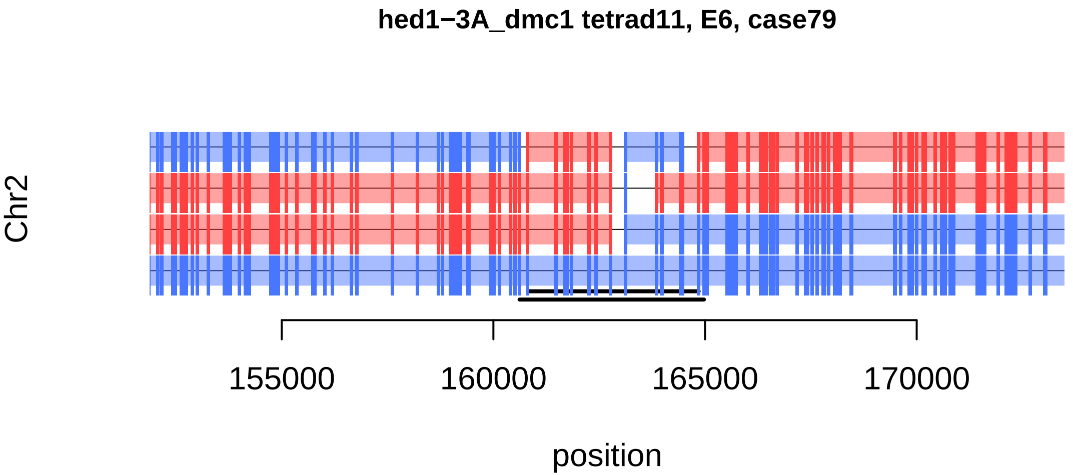
hed1−3A_dmc1 tetrad11, E6, case79
Chr2
155000
160000
165000
170000
position

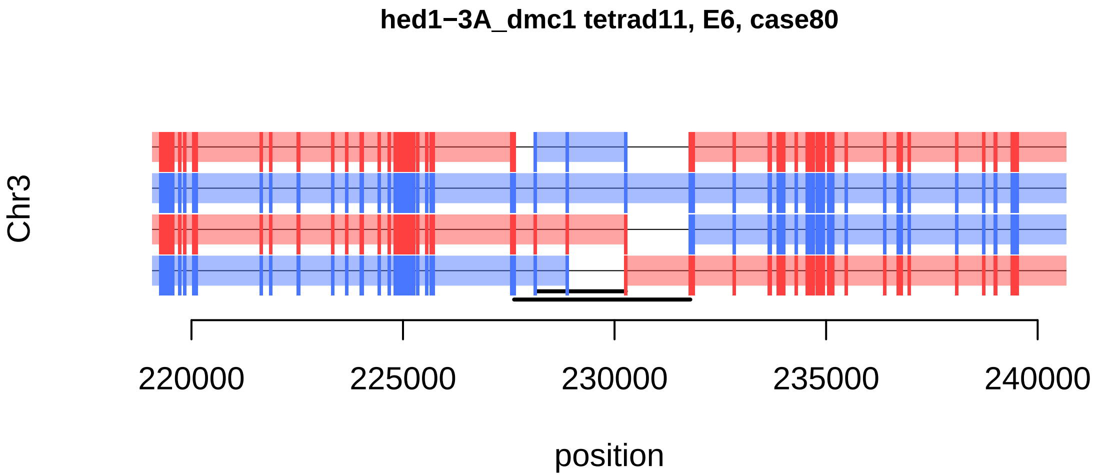

hed1−3A_dmc1 tetrad11, E6, case80
Chr3
220000
225000
230000
235000
240000
position

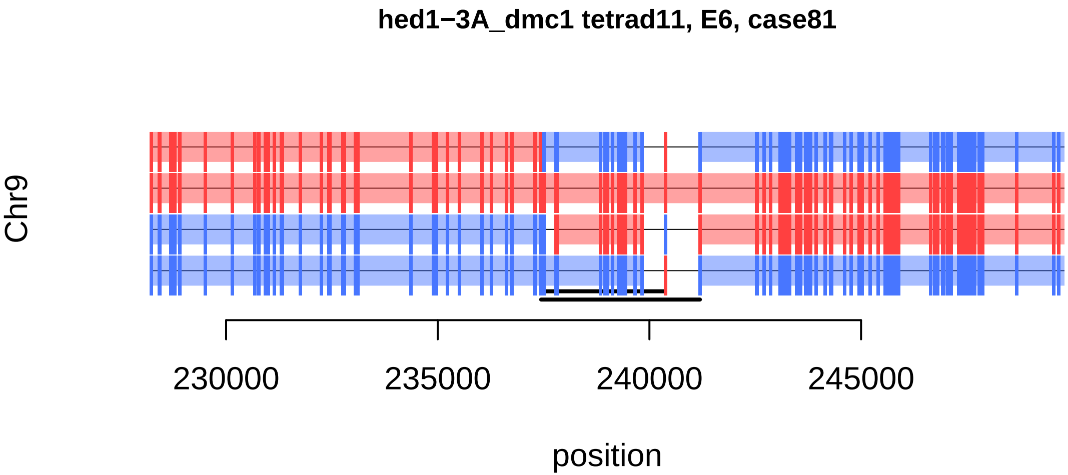
hed1−3A_dmc1 tetrad11, E6, case81
Chr9
230000
235000
240000
245000
position

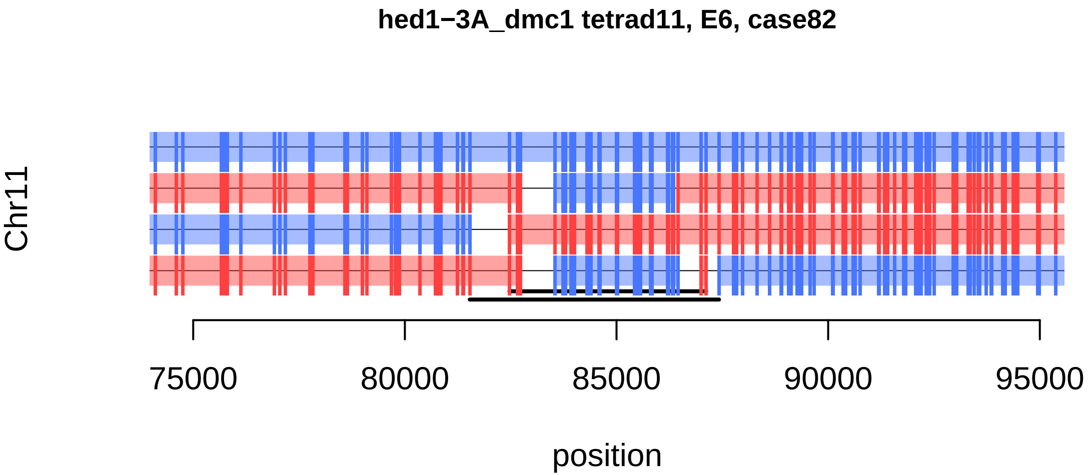
hed1−3A_dmc1 tetrad11, E6, case82
Chr11
75000
80000
85000
90000
95000
position

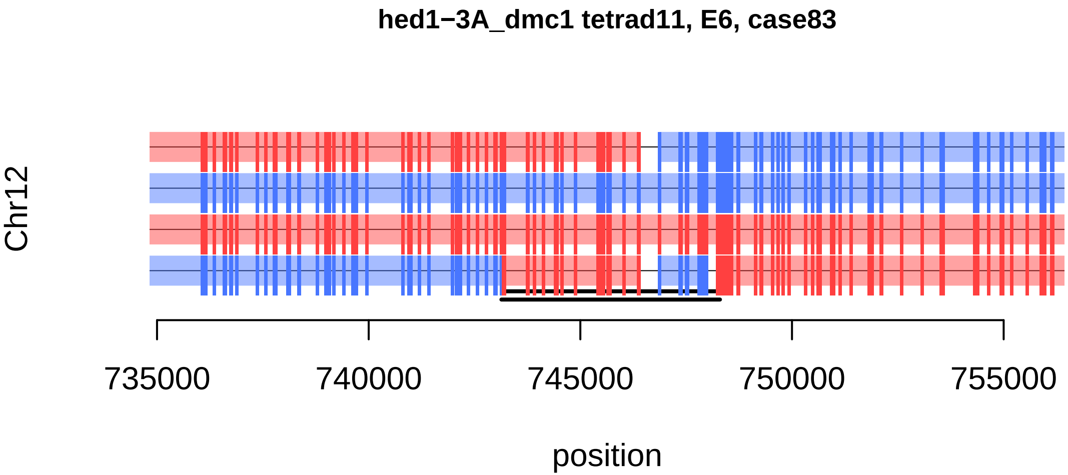

hed1−3A_dmc1 tetrad11, E6, case83
Chr12
735000
740000
745000
750000
755000
position

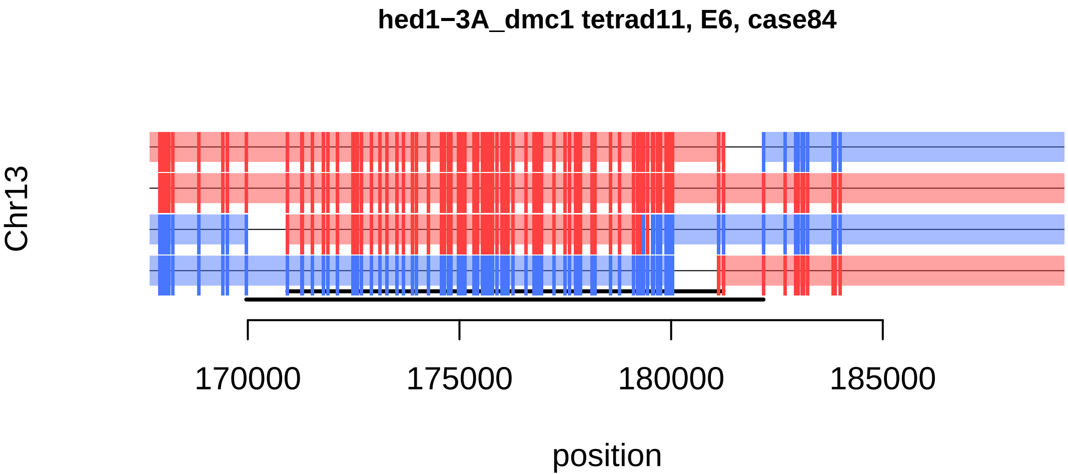
hed1−3A_dmc1 tetrad11, E6, case84
Chr13
170000
175000
180000
185000
position

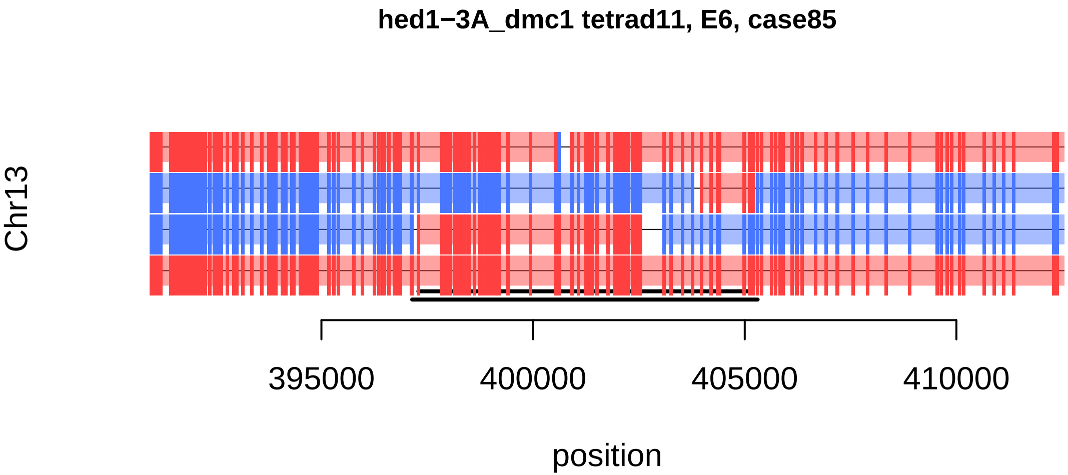
hed1−3A_dmc1 tetrad11, E6, case85
Chr13
395000
400000
405000
410000
position

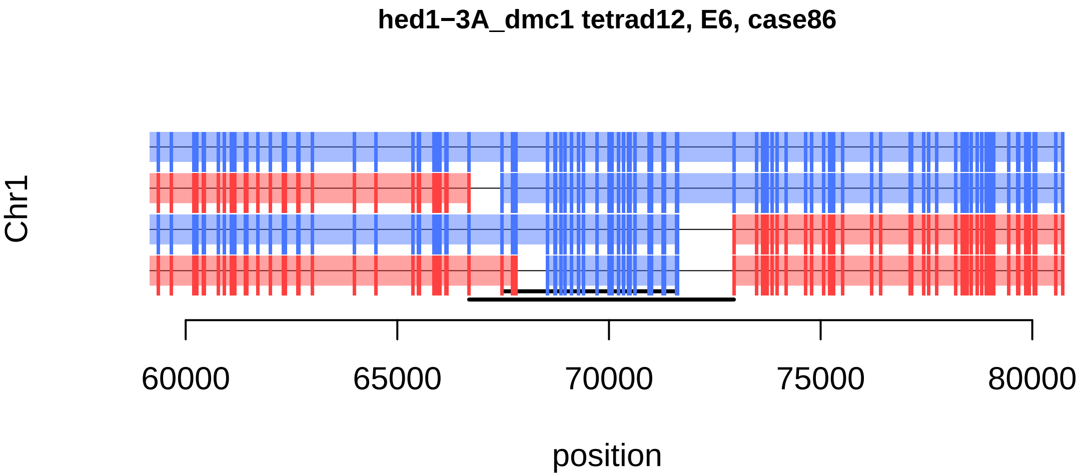
hed1−3A_dmc1 tetrad12, E6, case86
Chr1
60000
65000
70000
75000
80000
position

hed1−3A_dmc1 tetrad12, E6, case87

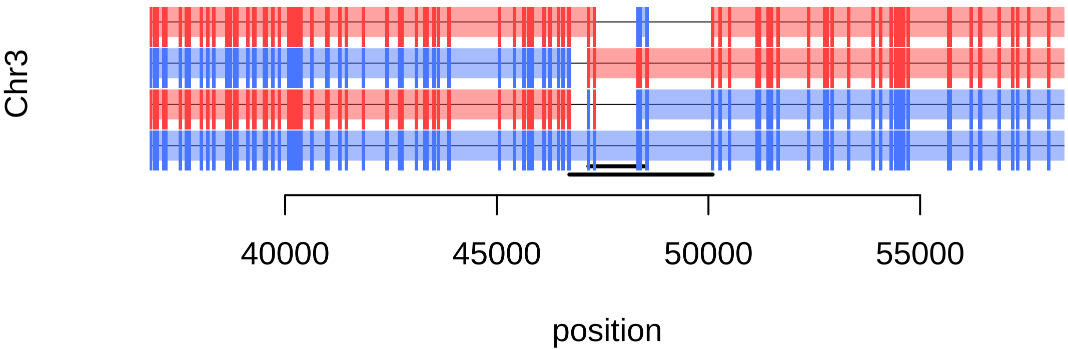

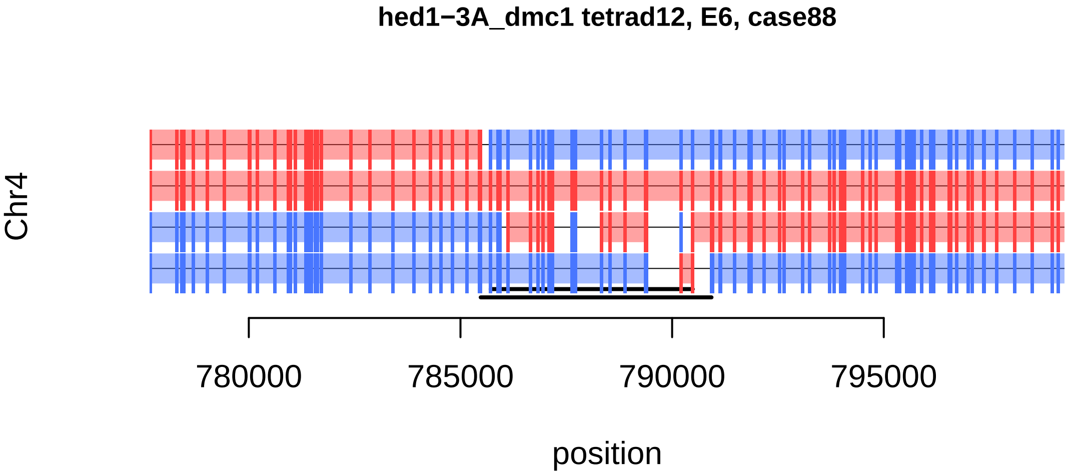
hed1−3A_dmc1 tetrad12, E6, case88
Chr4
780000
785000
790000
795000
position

hed1−3A_dmc1 tetrad12, E6, case89

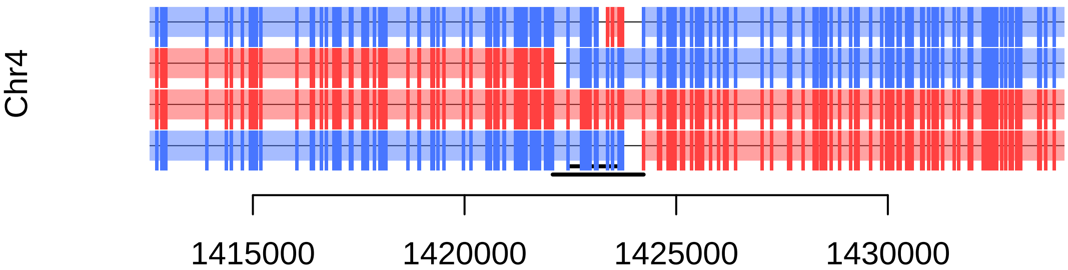

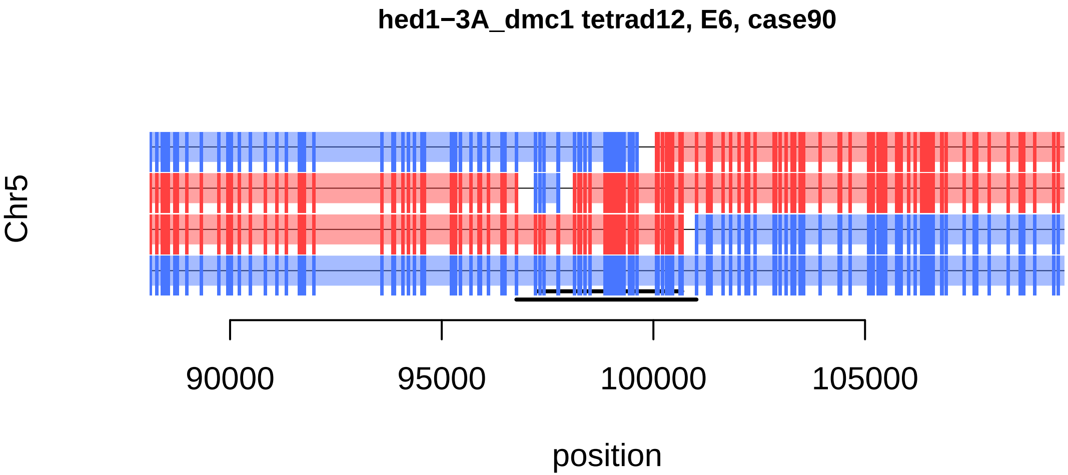
hed1−3A_dmc1 tetrad12, E6, case90
Chr5
90000
95000
100000
105000
position

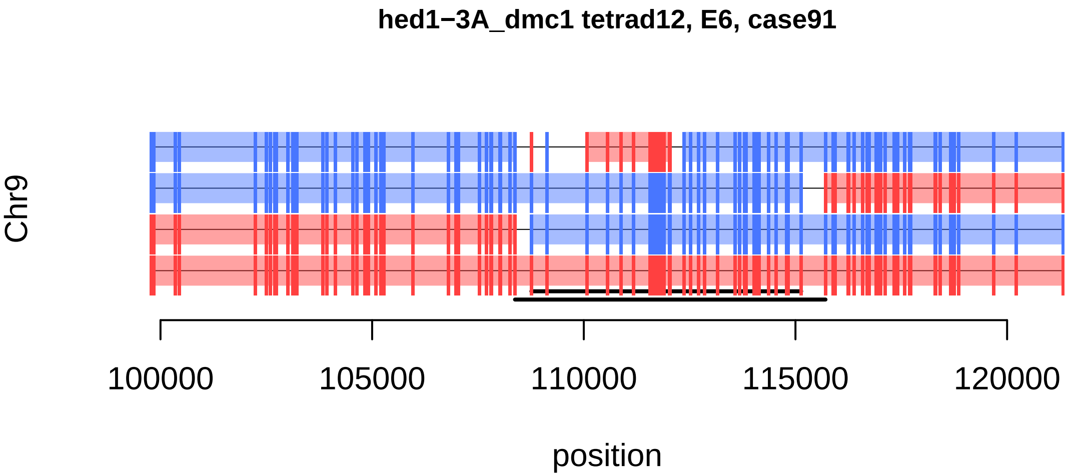
hed1−3A_dmc1 tetrad12, E6, case91
Chr9
100000
105000
110000
115000
120000
position

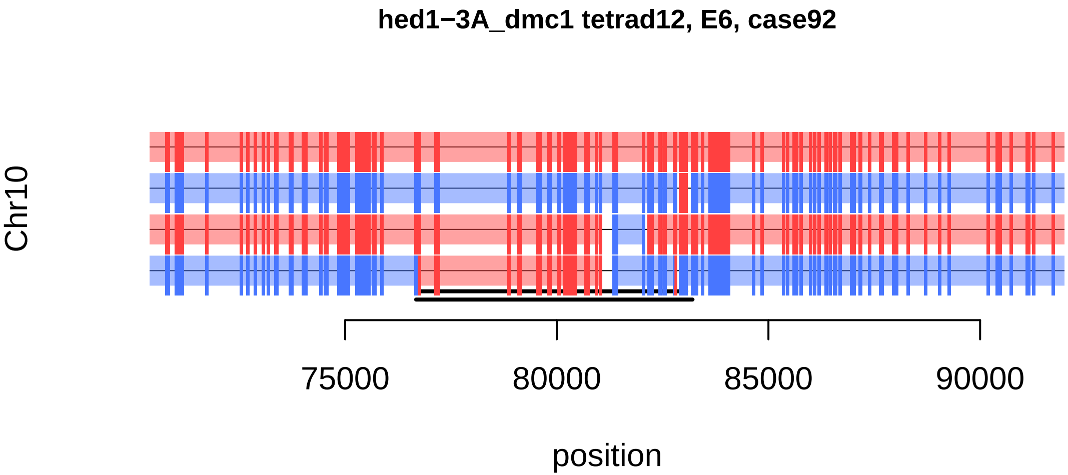
hed1−3A_dmc1 tetrad12, E6, case92
Chr10
75000
80000
85000
90000
position

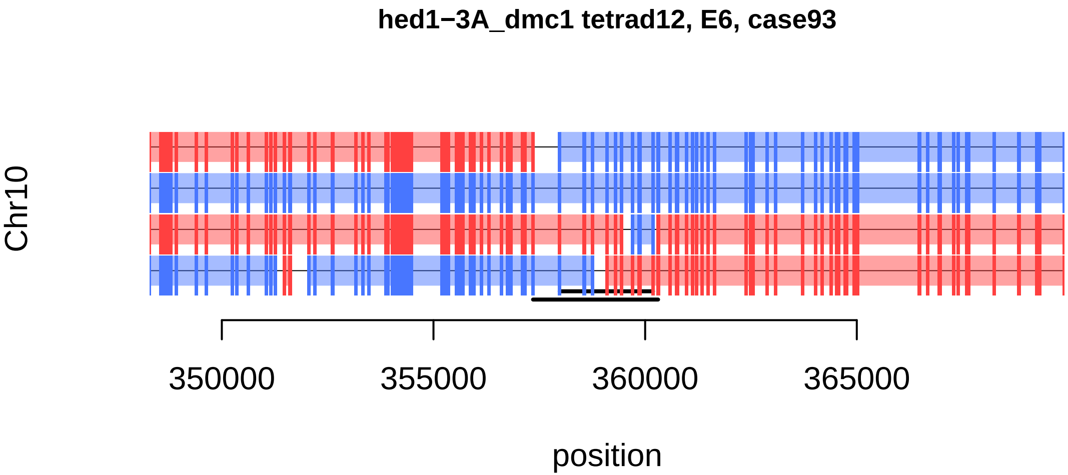
hed1−3A_dmc1 tetrad12, E6, case93
Chr10
350000
355000
360000
365000
position

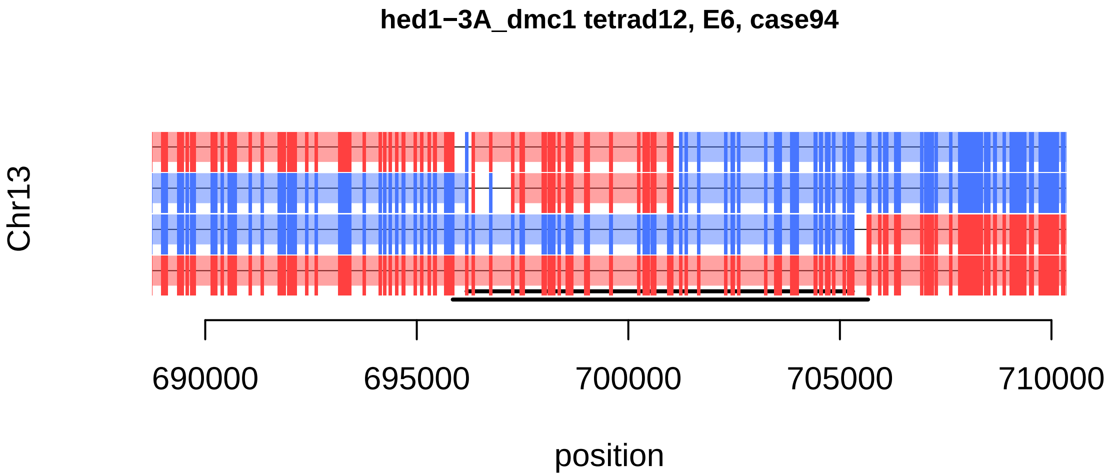
hed1−3A_dmc1 tetrad12, E6, case94
Chr13
690000
695000
700000
705000
710000
position

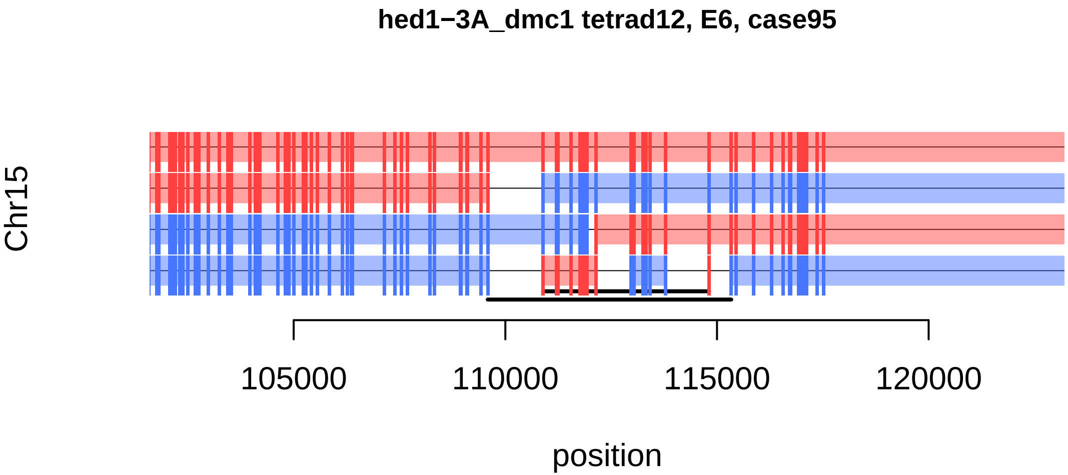
hed1−3A_dmc1 tetrad12, E6, case95
Chr15
105000
110000
115000
120000
position

hed1−3A_dmc1 tetrad12, E6, case96

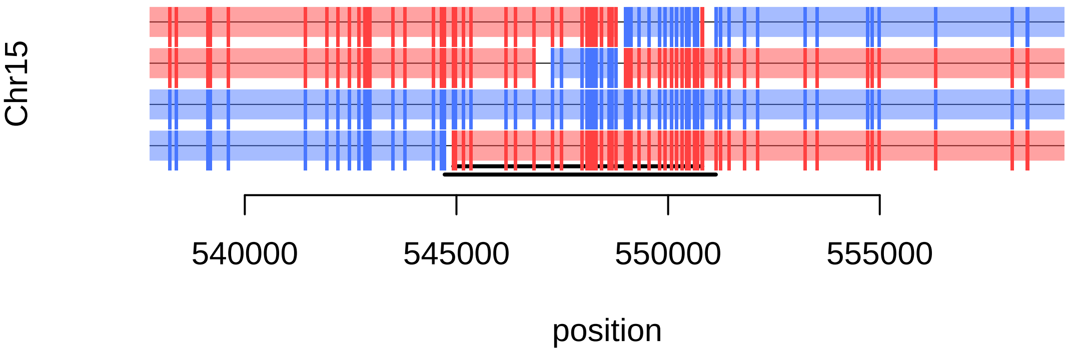

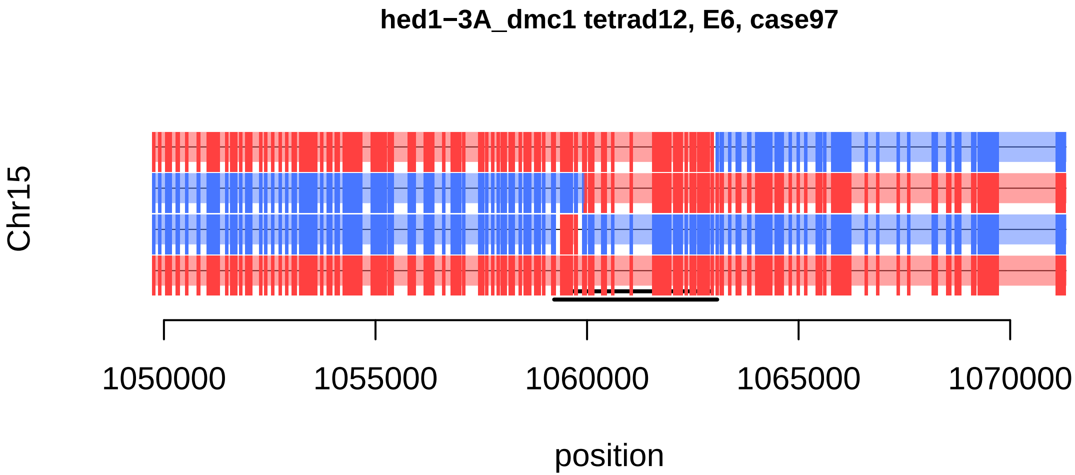
hed1−3A_dmc1 tetrad12, E6, case97
Chr15
1050000
1055000
1060000
1065000
1070000
position

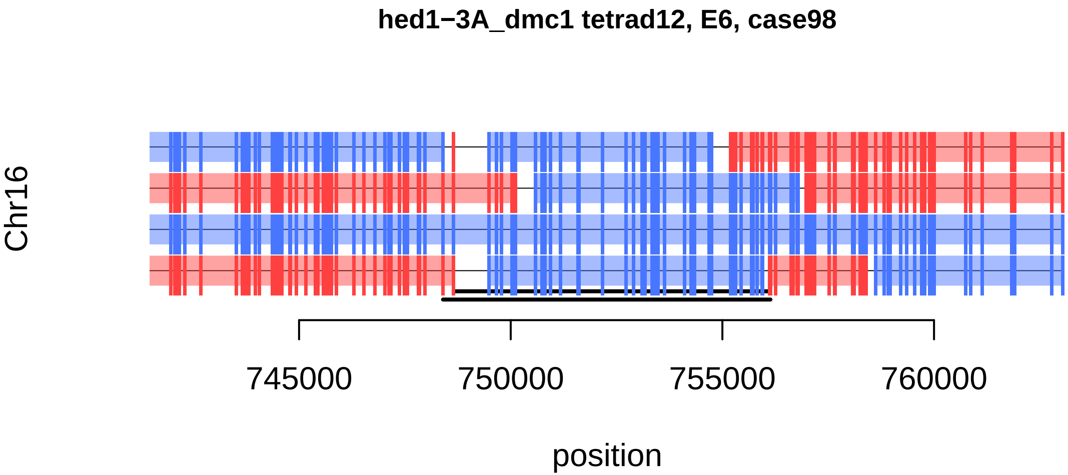
hed1−3A_dmc1 tetrad12, E6, case98
Chr16
745000
750000
755000
760000
position

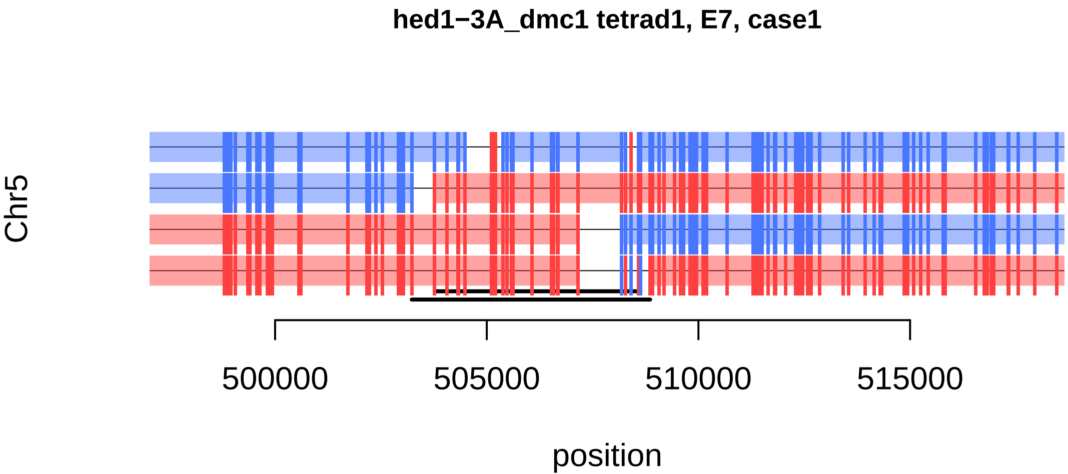

hed1−3A_dmc1 tetrad1, E7, case1
Chr5
500000
505000
510000
515000
position

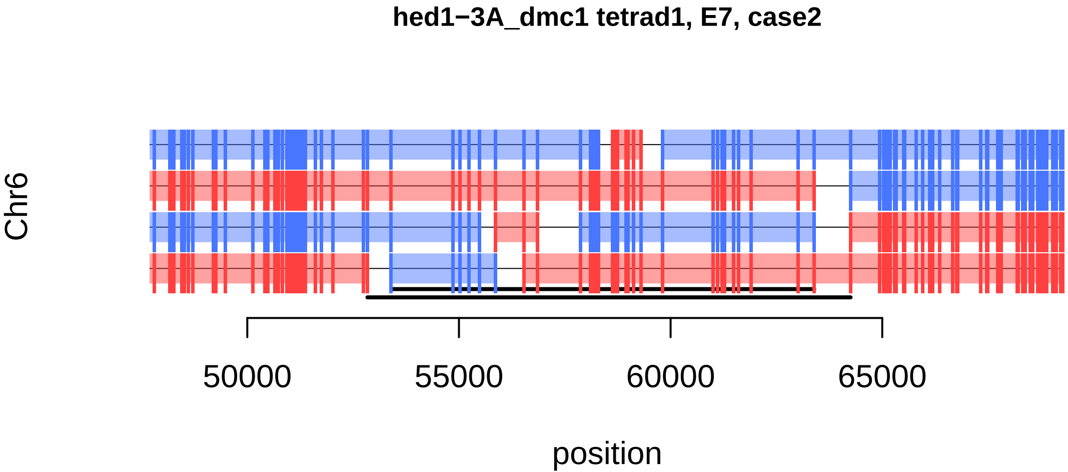
hed1−3A_dmc1 tetrad1, E7, case2
Chr6
50000
55000
60000
65000
position

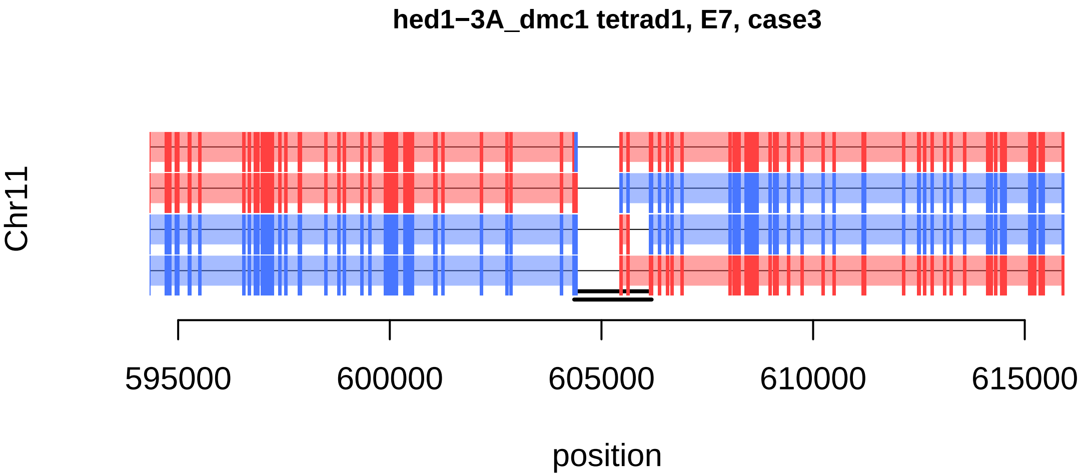
hed1−3A_dmc1 tetrad1, E7, case3
Chr11
595000
600000
605000
610000
615000
position

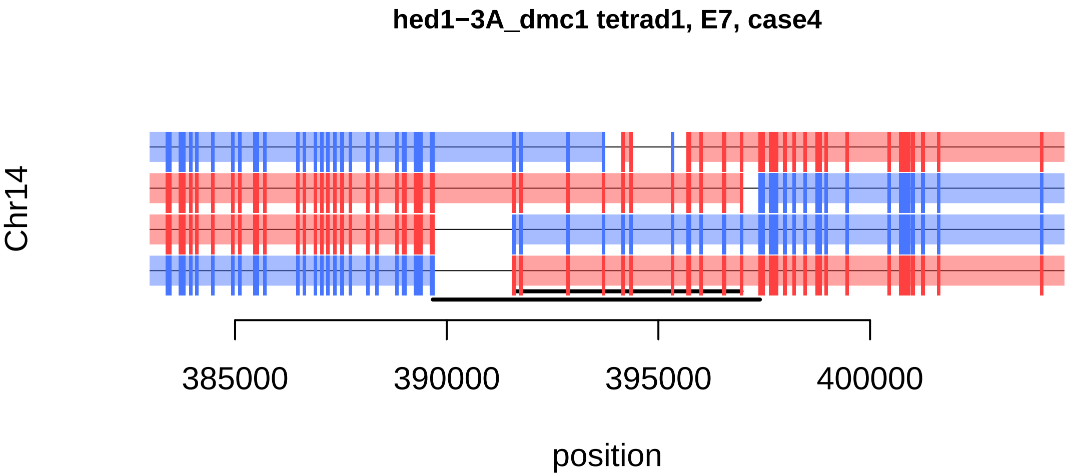
hed1−3A_dmc1 tetrad1, E7, case4
Chr14
385000
390000
395000
400000
position

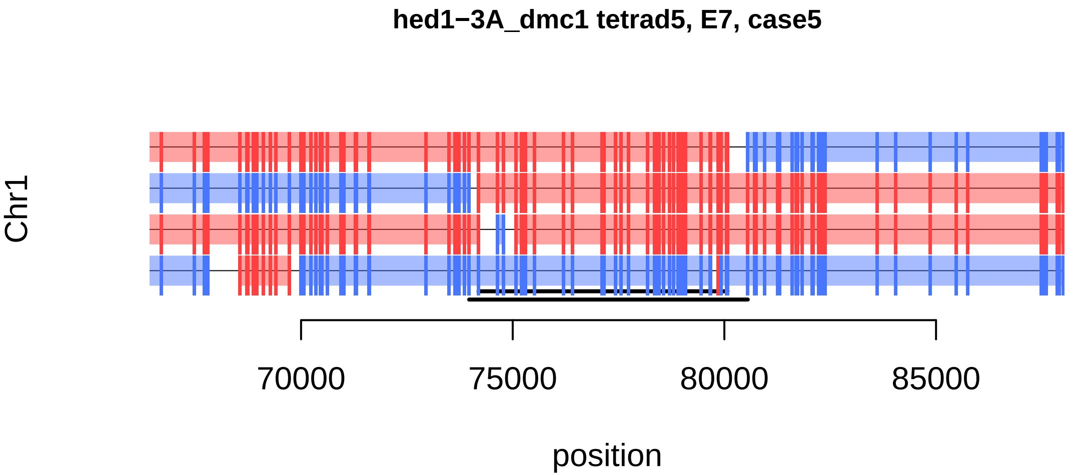

hed1−3A_dmc1 tetrad5, E7, case5
Chr1
70000
75000
80000
85000
position

hed1−3A_dmc1 tetrad5, E7, case6

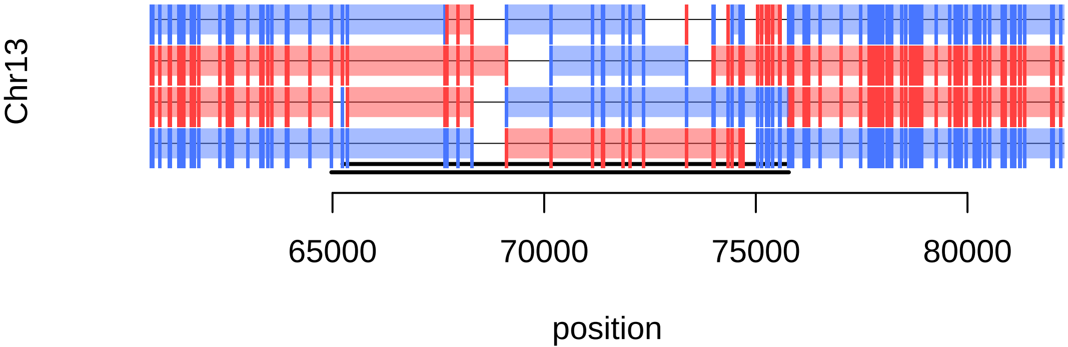

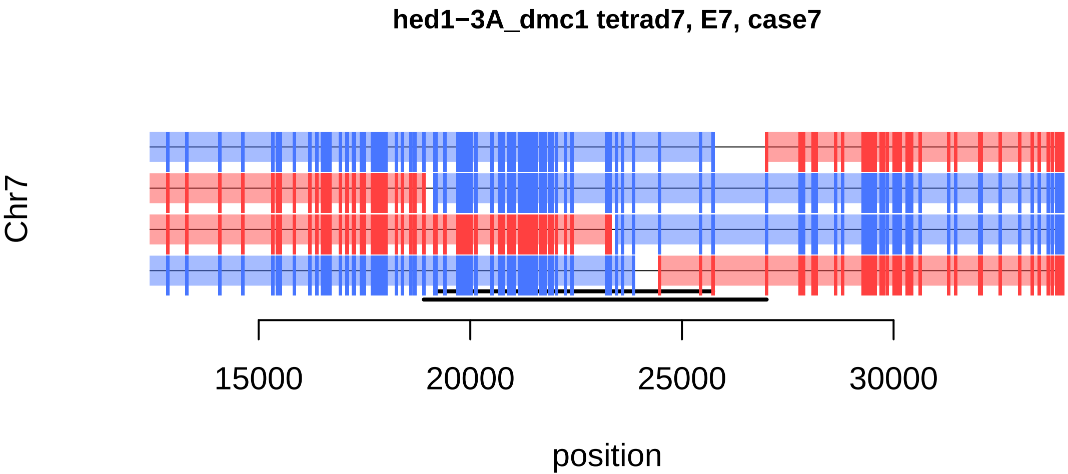

hed1−3A_dmc1 tetrad7, E7, case7
Chr7
15000
20000
25000
30000
position

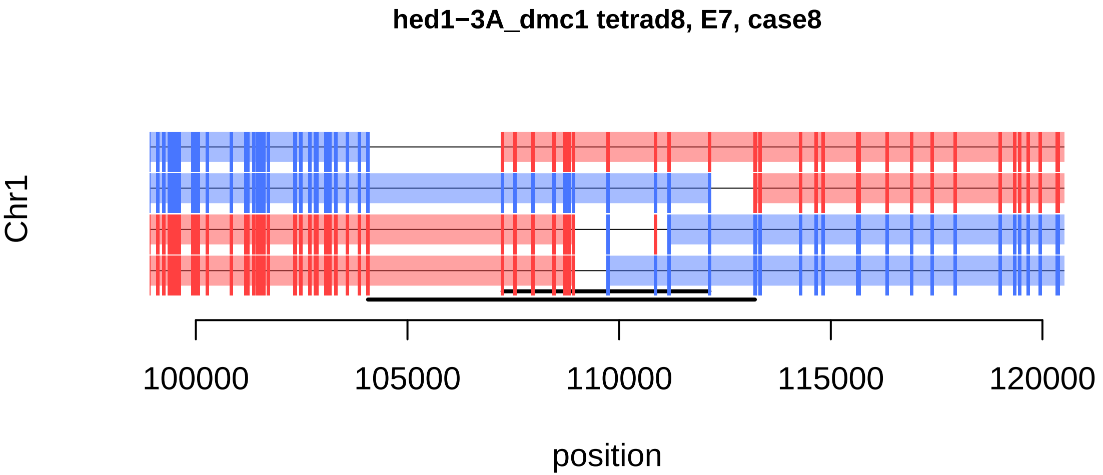

hed1−3A_dmc1 tetrad8, E7, case8
Chr1
100000
105000
110000
115000
120000
position

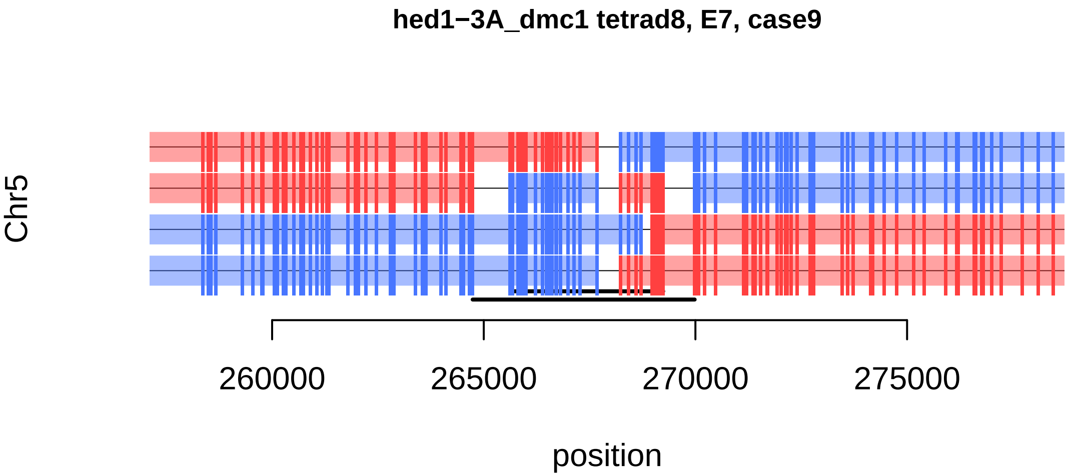
hed1−3A_dmc1 tetrad8, E7, case9
Chr5
260000
265000
270000
275000
position

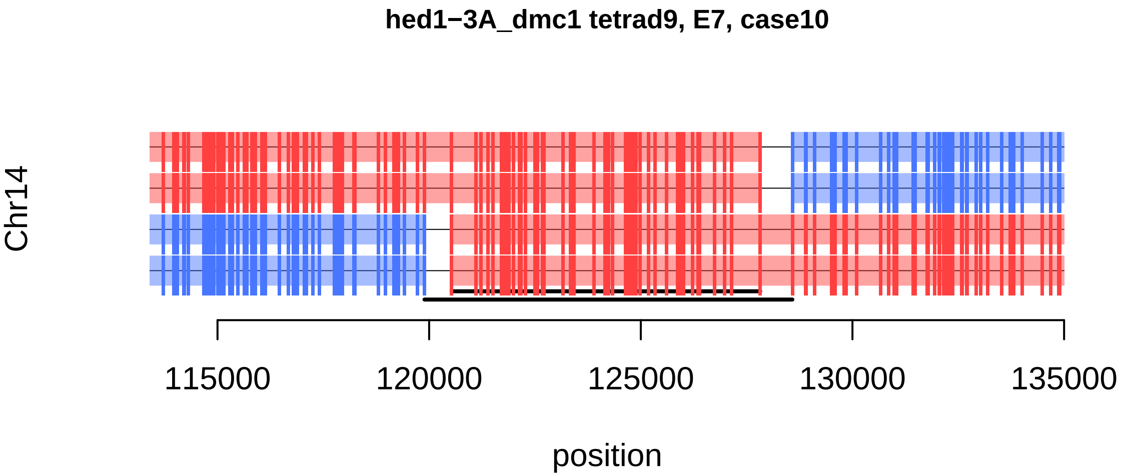
hed1−3A_dmc1 tetrad9, E7, case10
Chr14
115000
120000
125000
130000
135000
position

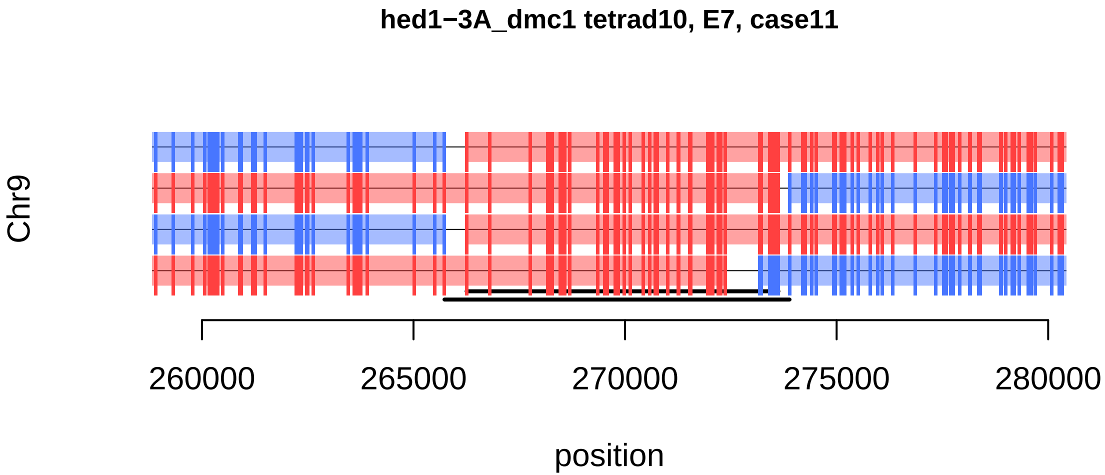

hed1−3A_dmc1 tetrad10, E7, case11
Chr9
260000
265000
270000
275000
280000
position

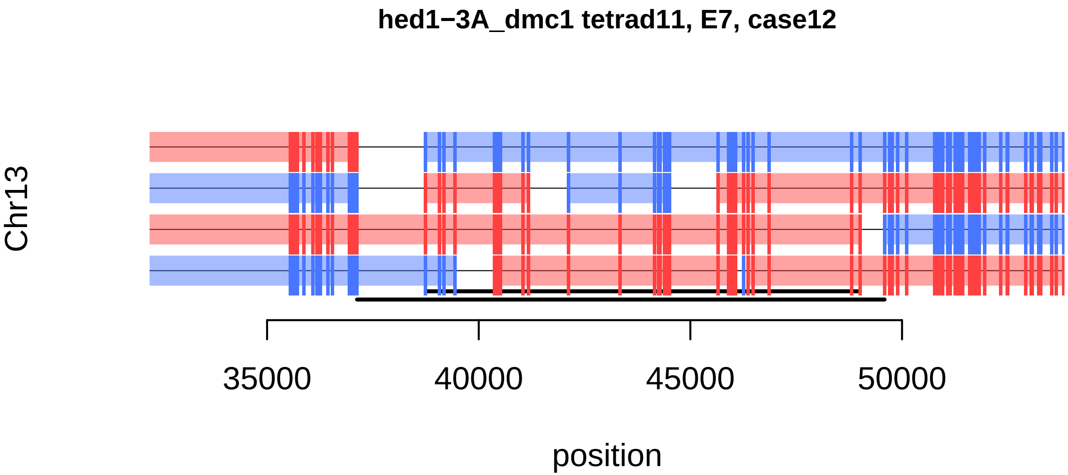

hed1−3A_dmc1 tetrad11, E7, case12
Chr13
35000
40000
45000
50000
position

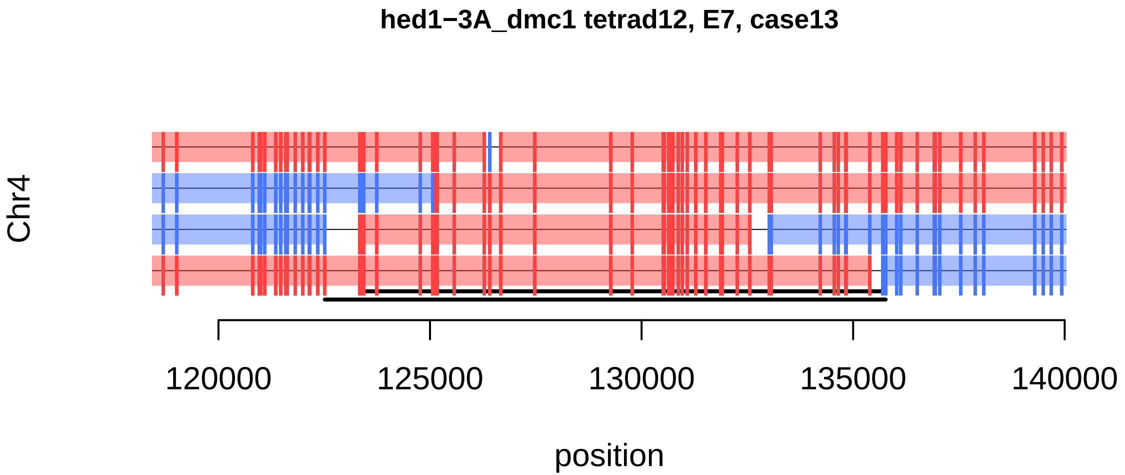
hed1−3A_dmc1 tetrad12, E7, case13
Chr4
120000
125000
130000
135000
140000
position
